# Local Genetic Correlations and Pleiotropy Reveal Shared Genetic Architecture Between COVID-19 Phenotypes and Prostate Cancer in European Populations

**DOI:** 10.7150/jca.111126

**Published:** 2025-10-10

**Authors:** Rong Xiang, Xunying Zhao, Lin Chen, Xueyao Wu, Jinyu Xiao, Xia Jiang

**Affiliations:** 1 Department of Nutrition and Food Hygiene, West China School of Public Health and West China Fourth Hospital, Sichuan University, Chengdu, China.; 2 Department of Epidemiology and Biostatistics and West China-PUMC C. C. Chen Institute of Health, West China School of Public Health and West China Fourth Hospital, Sichuan University, Chengdu, Sichuan, China.; 3 Department of Clinical Neuroscience, Center for Molecular Medicine, Karolinska Institutet, Solna, Stockholm, Sweden.

**Keywords:** COVID-19, prostate cancer, genetic correlation, genome-wide cross-trait analysis, transcriptome-wide association study, Mendelian randomization

## Abstract

**Background:** While a link between coronavirus disease 2019 (COVID-19) and prostate cancer (PrCa) has been observed in clinical settings, the shared underlying genetic influences remain unclear.

**Methods:** Leveraging summary statistics from the hitherto largest genome-wide association studies (GWASs) of European-ancestry populations, we performed the first comprehensive genome-wide cross-trait analysis to investigate the shared genetic architecture, pleiotropy, and potential causal relationships between three COVID-19 phenotypes and PrCa.

**Results:** We found no evidence of significant genome-wide genetic correlations between COVID-19 phenotypes and PrCa (*P* > 0.05). However, after partitioning the whole genome into 2353 independent regions, we observed significant local genetic correlations at chromosome 1 (chr1), chr7, and chr14 for PrCa with at least one COVID-19 phenotype (*P* < 0.05/2353). Cross-trait meta-analysis identified 22 independent single nucleotide polymorphisms (SNPs) shared between PrCa and at least one COVID-19 phenotype, totalling 25 associations, including 2 with infection, 14 with hospitalization, and 9 with critical illness. Transcriptome-wide association study (TWAS) revealed eight distinct shared genes (*CCHCR1*, *TCF19*, *ADAM15*, *HLA-C*, *CYP21A1P*, *HCP5*,* ATF6B*, and *HLA-DQB2*), predominantly enriched in tissues of the respiratory, neurological, and reproductive systems. Bidirectional Mendelian randomization (MR) demonstrated no causal association between COVID-19 phenotypes and PrCa.

**Conclusions:** Using a multi-layered analytical framework, our study provides novel insights into the shared genetic bases between COVID-19 phenotypes and PrCa, supported by significant local genetic correlations, pleiotropic SNPs, and shared genes. These findings highlight common biological mechanisms rather than direct causal relationships, suggesting the limited benefits of additional PrCa screening in COVID-19 survivors. Furthermore, the identified genes represent promising targets for future mechanistic research and clinical interventions, warranting further validation.

## Introduction

The male preponderance of coronavirus disease 2019 (COVID-19), caused by acute respiratory syndrome coronavirus 2 (SARS-CoV-2), has been well-documented in terms of both morbidity and mortality 1, 2. Men appear to be more likely to develop severe clinical outcomes 3, with nearly three times the odds of ICU admission and a 39% higher risk of death compared to women 4, despite roughly equivalent infection rates 5. In addition to respiratory damage 6, adverse impacts of COVID-19 have also been observed in the male reproductive system, with a particular focus on the testis and spermatogenesis damage 7, 8.

Prostate cancer (PrCa), on the other hand, is the most common cancer of the male reproductive system which also poses a considerable burden on global public health 9. Epidemiological studies suggest a potential phenotypic link between COVID-19 and PrCa, driven by shared risk factors such as sex, age, and comorbidities. For instance, a population-wide study in Veneto, Italy, found a higher risk of SARS-CoV-2 infection in male cancer patients compared to the male general population [odds ratio (*OR*) = 1.79, 95% confidence interval (*CI*) = 1.62-1.98, *P* < 0.0001] 10. Moreover, COVID-19 patients with cancer tend to have a higher mortality rate (*OR* = 2.34; 95% *CI* = 1.15-4.77; *P* = 0.03) and more severe outcomes when compared with the noncancer population 11. Previous evidence also suggests that COVID-19 may predispose recovered patients to the development of PrCa 12, 13. However, the observational nature of these studies precludes definitive causal conclusions due to potential biases from confounding and reverse causation.

Mendelian randomization (MR) provides a robust framework for causal inference by utilizing genetic variants as instrumental variables (IVs), thereby minimizing the limitations inherent in observational studies. To date, only four MR studies have investigated the causal relationships between COVID-19 phenotypes and PrCa, consistently reporting null associations (all *P*-values > 0.05) 14-17. However, these studies exhibit several key limitations: (i) reliance on a single COVID-19 phenotype (i.e., COVID-19 hospitalization) 14 and outdated GWAS datasets (Release 5 vs. our Release 7) 14-16, resulting in an approximately threefold smaller case sample; (ii) use of broad, non-specific outcome definitions (e.g., male genital cancers) with an approximately 12-fold smaller case sample 14; and (iii) dependence on basic MR approaches 14-16, or limited extensions to genome-wide genetic correlation and colocalization analyses 17, without incorporating a comprehensive cross-trait genetic framework. Beyond causality, shared signaling pathways involving androgen secretion, immunosuppression, and inflammation have been linked to the relationship between COVID-19 and PrCa 1, 18. Moreover, the *TMPRSS2* gene may underlie this relationship 19, 20, implying a common biological connection. Despite advances in statistical genetics and the growing availability of large-scale GWASs of European ancestry, no study has yet systematically investigated the shared genetic architecture underlying COVID-19 phenotypes and PrCa.

**Figure 1:**
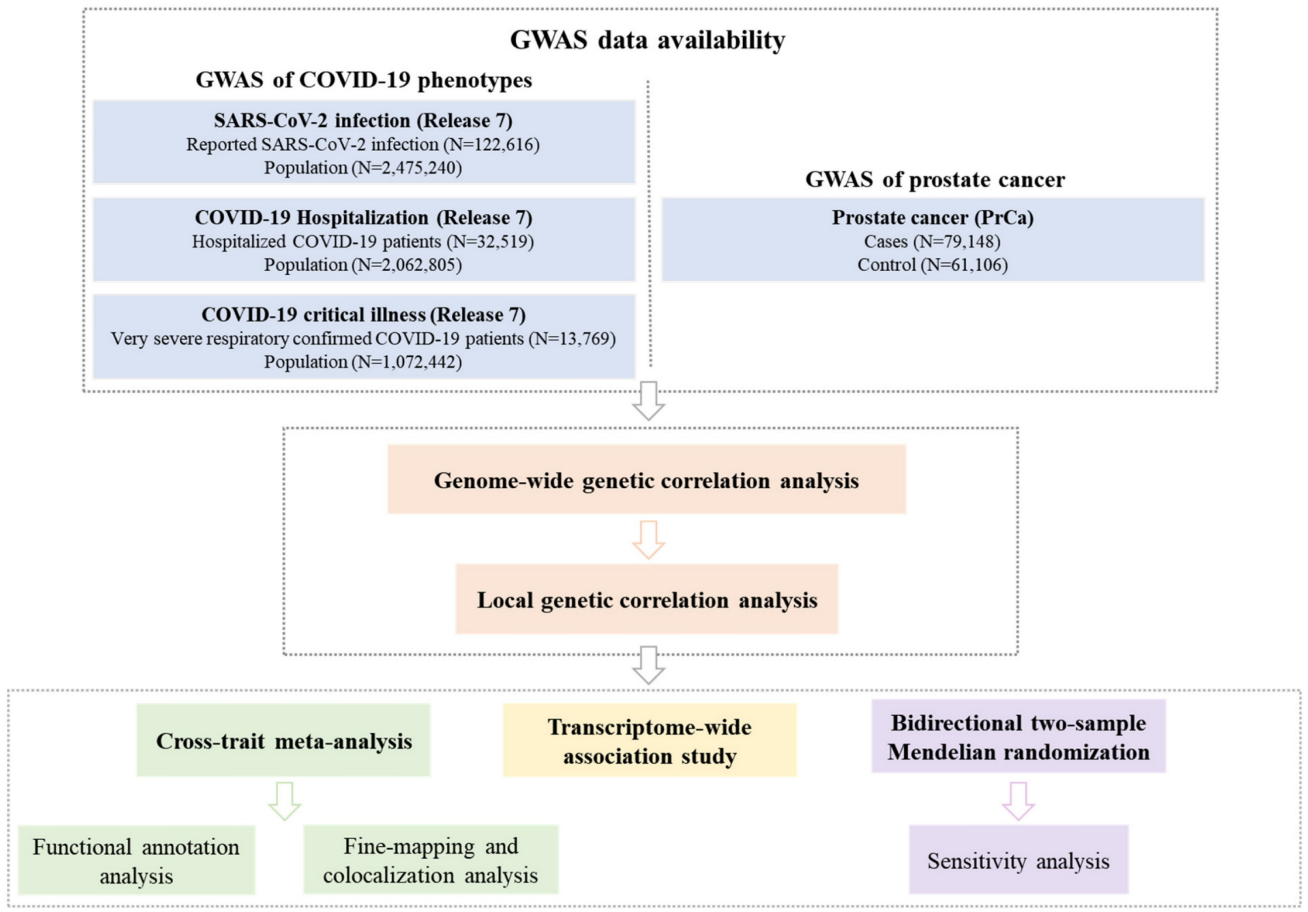
** Overall study design of the current genome-wide cross-trait analysis.** Note: Leveraging GWAS summary statistics from the hitherto largest publicly available GWAS and gene expression weights across 49 tissues from the GTEx (version 8) database, we conducted a comprehensive genome-wide cross-trait analysis to explore shared genetic architecture underlying the traits. This analysis utilizes a range of statistical genetic methods, including genetic correlation analysis to identify the shared genetic basis, cross-trait meta-analysis to detect shared loci, TWAS to identify gene-tissue pairs, and MR to infer causality. GWAS: genome-wide association study. GTEx: Genotype-Tissue Expression. TWAS: transcriptome-wide association studies. MR: Mendelian randomization.

To address this gap, we conducted a comprehensive genome-wide cross-trait analysis to investigate the genetic overlap and potential causal relationship between COVID-19 phenotypes and PrCa. Our analytical framework included: (I) global and local genetic correlation analyses to quantify shared genetic architecture; (II) cross-trait meta-analysis to identify pleiotropic loci; (III) transcriptome-wide association study (TWAS) to pinpoint shared gene-tissue pairs; and (IV) MR to evaluate causal effects. The overall study design is presented in **Figure 1**.

## Materials and Methods

### GWAS data sets

Summary statistics for PrCa and COVID-19 were retrieved from publicly available GWASs, all of European ancestry (details listed in **Supplementary Table 1**).

The GWAS summary data of PrCa was obtained from a meta-analysis of eight GWASs in the Prostate Cancer Association Group to Investigate Cancer Associated Alterations in the Genome (PRACTICAL) consortium 21, involving 79,148 cases and 61,106 controls. Quality control (QC) excluded samples with call rates < 95%, extreme heterozygosity (> 4.9 standard deviations [*SD*s]), duplicates, and first-degree relatives. SNPs with call rates < 95%, Hardy-Weinberg Equilibrium (HWE) *P*-values < 10^-7^ (controls) or < 10^-12^ (cases), minor allele frequency (MAF) < 1%, and imputation *r²* < 0.3 were excluded. The first seven principal components (PCs) and relevant covariates were adjusted to minimize bias.

For COVID-19, the hitherto largest GWAS was conducted by the COVID-19 Host Genetics Initiative (HGI, Release 7) 22 (https://www.covid19hg.org/), with the subjects of 23andMe excluded due to data restrictions. Three phenotypes were selected and further divided into two categories, representing COVID-19 susceptibility and severity. **SARS-CoV-2 infection**, cases with reported SARS-CoV-2 infection regardless of symptoms (*N* = 122,616) vs. population (*N* = 2,475,240), was used to index COVID-19 susceptibility. **COVID-19 hospitalization**, moderate or severe COVID-19 patients who were hospitalized due to COVID-19 symptoms (*N* = 32,519) vs. population (*N* = 2,062,805), and **COVID-19 critical illness**, severe COVID-19 patients who needed respiratory support or who died due to the disease (*N* = 13,769) vs. population (*N* = 1,072,442), were used to index COVID-19 severity. QC excluded samples with call rates < 98%, extreme heterozygosity (> 0.2 *SD*), and sex inconsistencies. SNPs with call rates < 95% (or < 98% for strict datasets), HWE *P*-value < 10^-6^ (controls) or < 10^-10^ (cases), and missing data differences > 2% were excluded. Confounding factors, including age, sex, PCs, and study-specific covariates were controlled to minimize bias.

### Statistical analyses

#### Genome-wide genetic correlation analysis to quantify global shared genetic basis

To assess the average genetic effects shared between PrCa and COVID-19 phenotypes, we performed a genome-wide genetic correlation analysis using linkage disequilibrium (LD) score regression (LDSC) 23. LDSC estimates global genetic correlation (*r_g_*), ranging from -1 (complete negative correlation) to +1 (complete positive correlation). We utilized pre-computed LD scores from ~1.2 million common and well-imputed SNPs in individuals of European ancestry represented in the Hapmap3 reference panel 24. Bonferroni correction was applied to account for multiple testing (*P* < 0.05/3).

#### Local genetic correlation analysis to quantify local shared genetic basis

Genome-wide genetic correlation analysis evaluates the overall genetic similarity between two traits but may overlook specific genomic regions that contribute discretely to this similarity. Therefore, we further measured the pairwise local genetic correlations between COVID-19 phenotypes and PrCa using SUPERGNOVA 25. SUPERGNOVA provides a precise quantification of genetic correlation between trait pairs by quantifying genetic variation within 2,353 independent LD blocks, each with an average length of 1.6 centimorgans. Bonferroni correction was applied to account for multiple testing (*P* < 0.05/2353).

#### Cross-trait meta-analysis to identify pleiotropic loci affecting both traits

The Cross-phenotype association analysis (CPASSOC) was performed to identify pleiotropic loci affecting both traits 26. Using GWAS summary statistics of single SNP-trait associations, CPASSOC provides two test statistics, *S*_Hom_ and *S*_Het_. *S*_Hom_, based on the fixed-effect meta-analysis method, is more powerful for homogenous genetic effect sizes, whereas *S*_Het_ is more powerful for heterogeneous effects, which are common when meta-analyzing distinct traits. Thus, we selected *S*_Het_ to combine summary statistics for COVID-19 phenotypes with PrCa.

The PLINK (version 1.9) “clumping” function was applied to obtain independent loci with the following parameters: --clump-p1 5e-8 -clump-p2 1e-5 -clump-r2 0.2 -clump-kb 500 27. Significant pleiotropic SNPs were defined as index variants satisfying *P*_single-trait_ < 1×10^-4^ (in each single trait) and *P*_CPASSOC_ < 5×10^-8^ (in cross-traits). Novel pleiotropic SNPs were defined as shared SNPs that are neither driven by a single trait (5×10^-8^ <* P*_single-trait_ < 1×10^-4^) nor in LD with index SNPs (*r^2^* < 0.2) identified in the original single-trait GWASs.

The Ensembl Variant Effect Predictor (VEP) 28 was used for detailed functional annotation of the identified pleiotropic SNPs.

#### Fine-mapping credible set analysis to identify causal variants among index SNPs

The index SNPs may not directly represent causal variants due to complex LD patterns among SNPs 29. Therefore, we conducted a fine-mapping analysis using FM-summary to identify a 99% credible set of causal variants within 250 kb of each index SNP 30. FM-summary is a Bayesian fine-mapping algorithm that focuses on the primary signal, sets a flat prior, and produces a posterior inclusion probability (PIP) of a true trait/disease association for each variant using the steepest descent approximation.

#### Colocalization analysis to determine shared or distinct variants among GWAS signals

To investigate whether the same variants or distinct nearby variants are responsible for two GWAS signals, we performed a colocalization analysis using Coloc 31. Coloc uses a Bayesian algorithm to generate posterior probabilities for five mutually exclusive hypotheses (H0: no association; H1/H2: association with one trait only; H3: association with both traits via two distinct SNPs; H4: association with both traits via a shared SNP). We extracted summary statistics for variants within 250 kb of the index SNP at each shared locus and calculated the posterior probability for H4 (*PPH4*). A locus was considered colocalized if *PPH4* exceeded 0.5.

#### Transcriptome-wide association study to identify shared gene-tissue pairs

Genetic variants influence complex traits by regulating gene expression in various tissues, thereby affecting protein abundance. To identify genes with shared expression patterns between COVID-19 and PrCa in specific tissues, we performed a transcriptome-wide association study (TWAS) using FUSION 32. We integrated GWAS summary data with expression weights across 49 tissues from the Genotype-Tissue Expression (GTEx, version 8) dataset, analyzing one tissue-trait pair at a time. Bonferroni correction was applied to account for multiple testing within each tissue.

#### Bidirectional two-sample Mendelian randomization analysis to infer causal relationships

To detect a putative causal relationship between COVID-19 phenotypes and PrCa, we performed a bidirectional two-sample MR analysis. For COVID-19, we identified independent IVs by clumping all variants that reached genome-wide significance (*P* < 5×10^-8^) using a strict criterion (*r^2^* ≤ 0.001 within a 1.0Mb window). For PrCa, we collected independent index SNPs previously reported to reach genome-wide significance (*P* < 5×10^-8^) from corresponding GWAS. The *F*-statistic was calculated to evaluate instrument strength, with a value greater than 10 indicating a strong instrument 33.

We applied the inverse-variance weighted (IVW) approach 34 as our primary approach, assuming all IVs are valid to provide reliable estimates. To further evaluate the robustness of our results and validate MR model assumptions, we performed several important sensitivity analyses, including: (I) the MR-Egger regression 35 to account for directional pleiotropy by incorporating an intercept term; (II) the weighted median approach 36 to improve robustness against invalid IVs (with fewer than half invalid); (III) the MR-Pleiotropy Residual Sum and Outlier (MR-PRESSO) approach 37 to detect and correct horizontal pleiotropy by identifying and removing outlying IVs; (IV) the leave-one-out analysis to detect outliers by removing one IV at a time and performing IVW on the remaining IVs; (V) the IVW method excluding palindromic IVs with strand ambiguity to avoid errors in effect allele identification; (VI) the IVW method excluding pleiotropic IVs associated with potential confounding phenotypes to mitigate the effects of pleiotropy based on the GWAS catalog (https://www.ebi.ac.uk/gwas/). A causal estimate was considered robustly significant if it was significant in IVW and showed consistency in all sensitivity analyses. Additionally, we performed reverse-direction MR to exclude potential reverse causality. Bonferroni correction was applied to account for multiple testing (*P* < 0.05/3).

## Results

### Genome-wide genetic correlation

We found no evidence of an overall shared genetic basis between PrCa and any of the COVID-19 phenotypes (infection: *r_g_* = -0.06, *P* = 0.20; hospitalization: *r_g_* = -0.04, *P* = 0.27; critical illness: *r_g_* = -0.04, *P* = 0.20) (**Table 1**).

### Local genetic correlation

**Figure 2:**
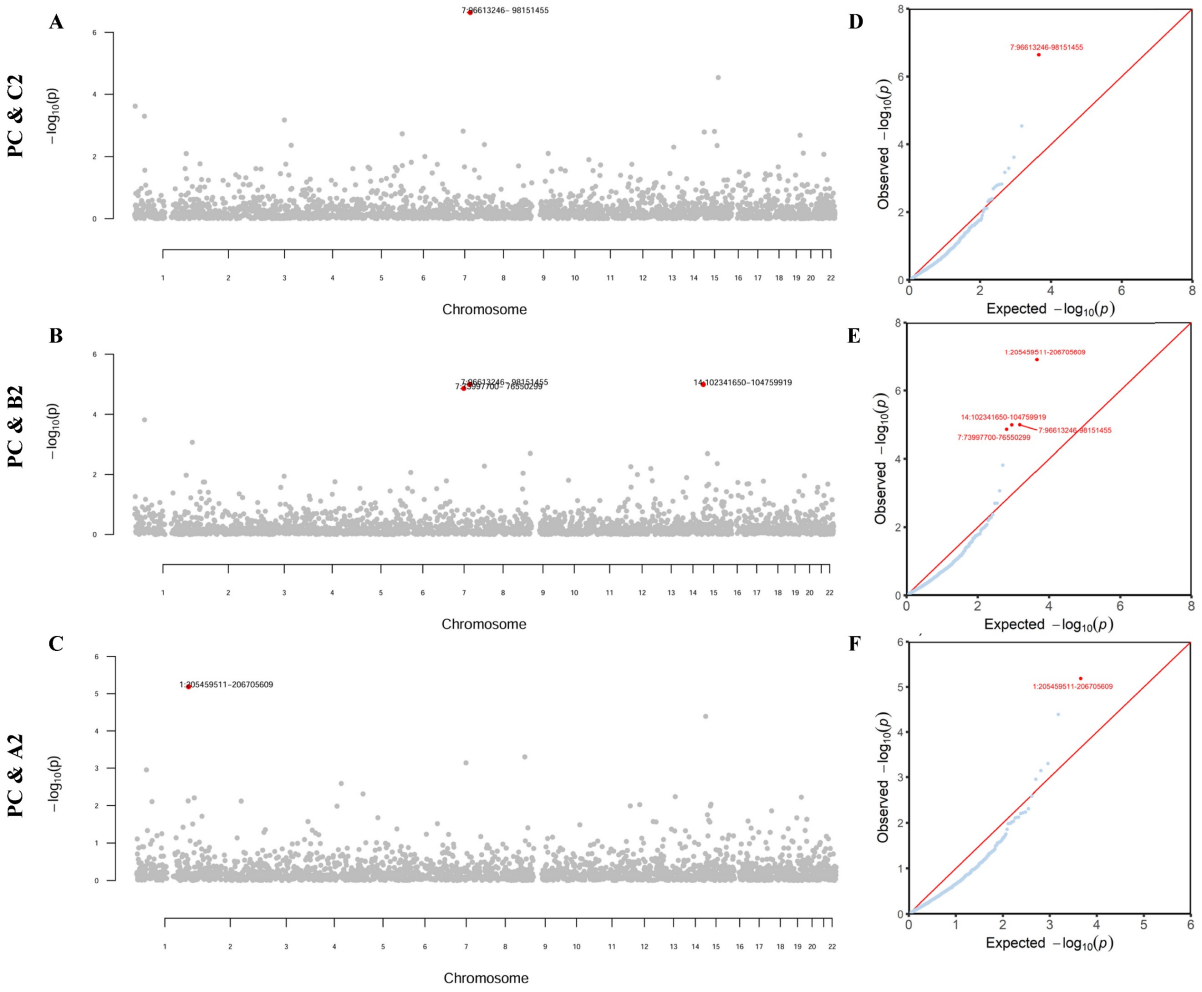
** Local genetic correlation between prostate cancer and COVID-19 phenotypes.** Note: (A-C) Manhattan plot showing the estimates of local genetic covariance between prostate cancer and COVID-19 phenotypes. The corresponding phenotype pairs are annotated at the left of each figure. For each plot, red dots represent loci with significant local genetic correlations after Bonferroni correction for multiple testing (*P* < 0.05/2353), reflecting true signals rather than random chance findings. (D-F) QQ-plot presenting region-specific *P* values from the local genetic correlation between prostate cancer and COVID-19 phenotypes. PC: prostate cancer; C2: SARS-CoV-2 infection; B2: COVID-19 hospitalization; A2: COVID-19 critical illness.

Partitioning the whole genome into 2353 independent LD blocks, we found four specific genomic regions that showed a significant local genetic correlation between PrCa and at least one COVID-19 phenotype (all *P*-values < 0.05/2353, **Figure 2**).

As detailed in **Supplementary Table 2**, one region (**7q21.3-7q22.1**) was shared by PrCa and infection *r_g_* = -4.79×10^-4^, *P* = 2.30×10^-7^). Additionally, four regions (**1q32.1**, 7q11.23, **7q21.3-7q22.1**, and 14q32.31-14q32.33) were shared by PrCa and hospitalization (*r_g_* = -4.00×10^-4^~3.68×10^-4^, *P* = 1.01×10^-5^~1.38×10^-5^). Furthermore, one region (**1q32.1**) was shared by PrCa and critical illness (*r_g_* = 5.36×10^-4^, *P* = 6.51×10^-6^). Notably, two genomic regions, 7q21.3-7q22.1 and 1q32.1, were identified repeatedly as significant regions.

### Cross-trait meta-analysis

**Figure 3:**
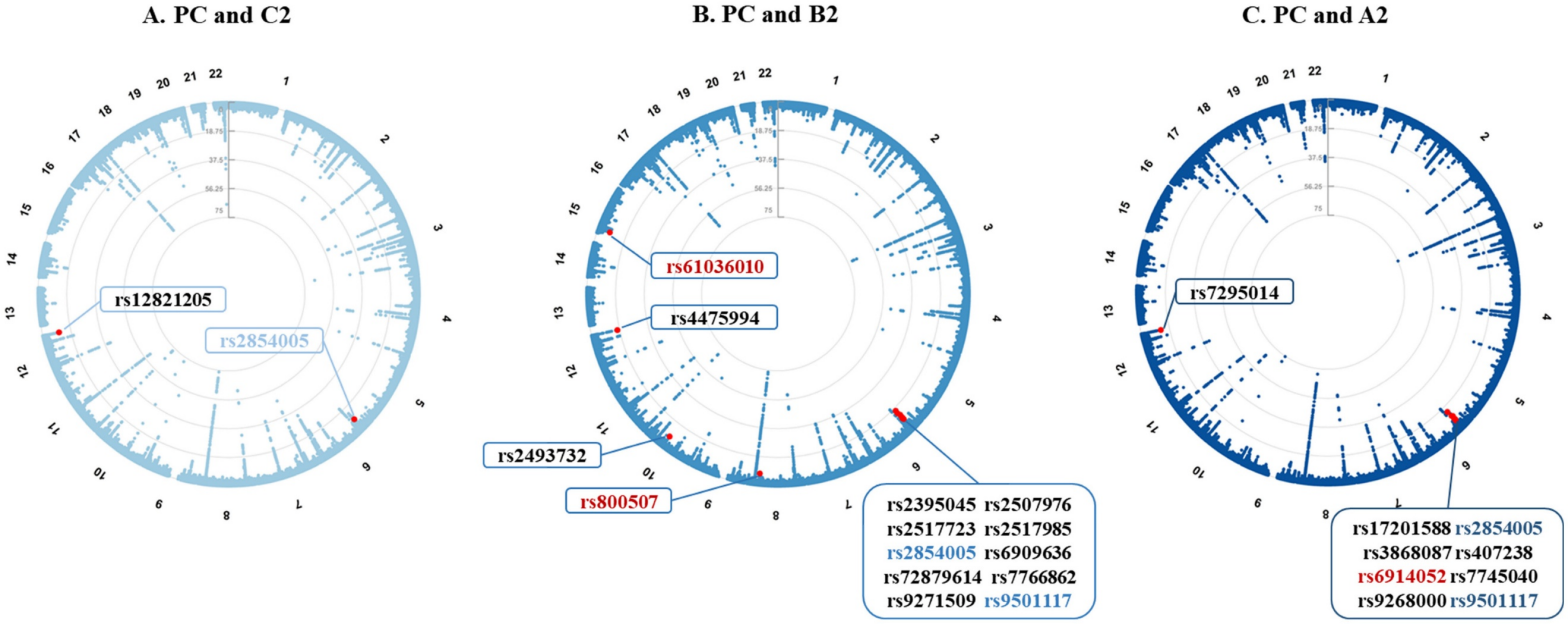
** Pleiotropic loci between prostate cancer and COVID-19 phenotypes identified from the cross-trait meta-analysis.** Note: (A-C) Circular Manhattan plots displaying pleiotropic loci identified for prostate cancer and COVID-19 phenotypes. The corresponding phenotype pairs are annotated at the top of each figure. For each plot, the outermost numbers represent chromosomes 1-22, and the red dots represent significant pleiotropic SNPs in the cross-trait meta-analysis (*P*_CPASSOC_ < 5×10^-8^ and *P*_single-trait_ < 1×10^-4^ in both traits). All 22 identified SNPs are listed in the textboxes with font colors denoting their classification. The fonts marked in red represent novel shared pleiotropic SNPs, and the fonts marked in (light/dark) blue represent shared pleiotropic SNPs for prostate cancer with at least two COVID-19 phenotypes. PC: prostate cancer; C2: SRAS-CoV-2 infection; B2: COVID-19 hospitalization; A2: COVID-19 critical illness; SNP: single nucleotide polymorphism.

To identify individual SNPs affecting both COVID-19 and PrCa, we next conducted a cross-trait meta-analysis. We identified a total of 22 independent pleiotropic SNPs shared between PrCa and at least one COVID-19 phenotype, totaling 25 associations, including 2 for infection, 14 for hospitalization, and 9 for critical illness (all fulfilled *P*_single-trait_ < 1×10^-4^ and *P*_CPASSOC_ < 5×10^-8^, **Figure 3**).

Additional details on the shared pleiotropic SNPs are provided in **Supplementary Table 3,** with their annotations included in **Supplementary Table 4**. Among these shared SNPs, the most significant was SNP rs4475994 (*P*_CPASSOC_ = 4.89×10^-18^) located at 12q24.33 near ***FBRSL1***. SNP rs9501117 located at 6q27 was found as a shared SNP between PrCa and the two COVID-19 severity phenotypes (hospitalization and critical illness), while SNP rs2854005 (harboring ***HLA-B***) located at 6q27 was found as a shared SNP between PrCa and all three COVID-19 phenotypes.

After excluding SNPs that were in LD (*r^2^* ≥ 0.2) with any of the previously reported single-trait-associated significant SNPs, we identified three novel shared SNPs for PrCa and two COVID-19 severity phenotypes. SNP rs6914052 (*P*_CPASSOC_ = 4.96×10^-8^) located at 6q27 was shared between PrCa and critical illness, while the other two SNPs (rs61036010 and rs800507) were shared between PrCa and hospitalization. SNP rs61036010 (*P*_CPASSOC_ = 1.05×10^-8^) located at 15q11.1-26.3 was near ***KLF13***, while SNP rs800507 (*P*_CPASSOC_ = 2.34×10^-8^) located at 8q24.3 was near ***TRPS1***.

### Fine-mapping credible set analysis

For all shared SNPs identified by the cross-trait meta-analysis, we identified a 99% credible set of causal variants. In total, 627 candidate SNPs were identified in the 99% credible set for PrCa and COVID-19 phenotypes, including 32 candidate SNPs for PrCa and infection, 395 candidate SNPs for PrCa and hospitalization, and 200 candidate SNPs for PrCa and critical illness. Lists of credible set SNPs in each shared pleiotropic SNP for PrCa and COVID-19 are shown in **Supplementary Table 5**.

### Colocalization analysis

Colocalization analysis was further performed to determine whether genetic variants driving the association in two traits are the same. As shown in **Supplementary Table 6**, we found four shared loci to colocalize at the same candidate SNPs (*PPH* > 0.5), including one shared locus (rs12821205) for PrCa and infection, two shared loci (rs4475994 and rs800507) for PrCa and hospitalization, and one shared locus (rs6914052) for PrCa and critical illness.

### Transcriptome-wide association study

We identified multiple TWAS-significant gene-tissue pairs shared between PrCa and COVID-19 phenotypes, suggesting gene-level genetic correlation across traits (**Table 2**). A total of eight genes were TWAS-significant for PrCa with at least one COVID-19 phenotype, including one for PrCa and infection, seven for PrCa and hospitalization, and five for PrCa and critical illness, mainly enriched in tissues of kidney cortex, adrenal gland, lung, brain, artery, whole blood, testis, uterus, and adipose. Among them, *HLA-C*, *TCF19*, and *ADAM15* were shared by PrCa and the two COVID-19 severity phenotypes, while *CCHCR1* was shared by PrCa and all three COVID-19 phenotypes. Notably, *CCHCR1* was also close to the pleiotropic locus (lead SNP: rs2517985) identified by cross-trait meta-analysis.

### Bidirectional two-sample Mendelian randomization

**Figure 4:**
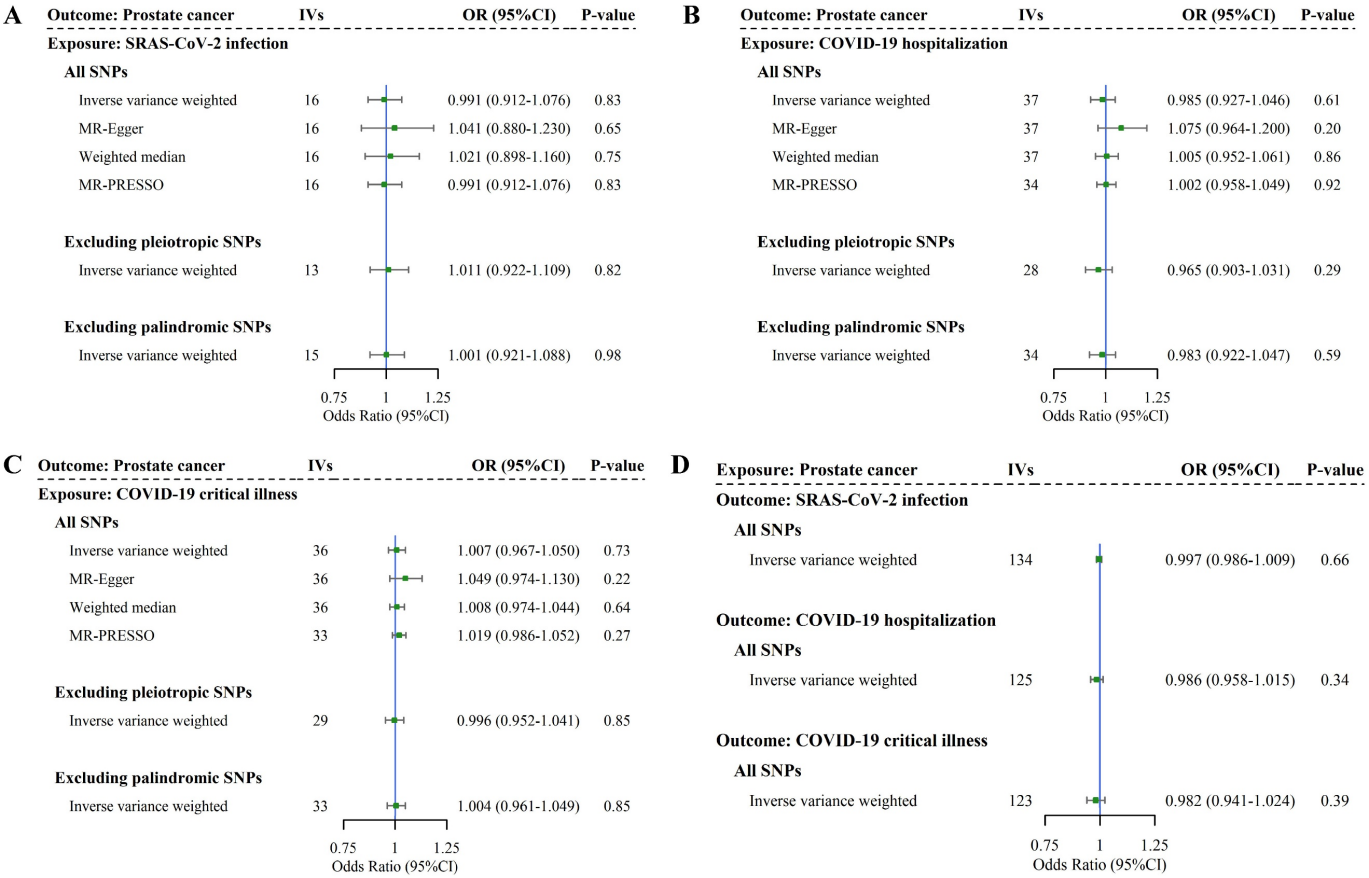
** Bidirectional causal relationship underlying COVID-19 phenotypes and prostate cancer.** Note: (A-C) Estimates of causal effect for genetic liability to COVID-19 phenotypes with prostate cancer. (D) Estimates of causal effect for genetic liability to prostate cancer with COVID-19 phenotypes. Boxes represent the point estimates of causal effects, and error bars represent 95% confidence intervals. IVs: instrumental variables; *OR*: odds ratio; *CI*: confidence interval; SNP: single nucleotide polymorphism.

We finally conducted a bidirectional two-sample MR analysis to examine the causal association. We identified 16, 38, and 37 SNPs as IVs for SAS-CoV-2 infection, COVID-19 hospitalization, and COVID-19 critical illness, respectively. The *F*-statistics for these IVs were in the order of 105.06, 73.84, and 77.49, suggesting strong instruments. More details of IVs selected for COVID-19 phenotypes are listed in **Supplementary Table 7**. As shown in **Figure 4** and **Supplementary Table 8**, no association between genetically predicted COVID-19 and PrCa was identified in IVW (infection: *OR* = 0.99, 95%*CI* = 0.91-1.08, *P* = 0.83; hospitalization: *OR* = 0.99, 95%*CI* = 0.93-1.05, *P* = 0.61; critical illness: *OR* = 1.01, 95%*CI* = 0.97-1.05, *P* = 0.73). Consistent with these findings, we did not observe any changes in the significance of estimates in MR-Egger (infection: *P* = 0.65; hospitalization: *P* = 0.20; critical illness: *P* = 0.22), weighted median (infection: *P* = 0.75; hospitalization: *P* = 0.86; critical illness: *P* = 0.64). Other sensitivity analyses (i.e., MR-PRESSO, IVW excluding palindromic or pleiotropic SNPs, and leave-one-out) yielded similar findings, with specific results presented in **Supplementary Tables 8**-**9**. The consistency in significance of estimates between IVW and all sensitivity analyses indicates the robustness of our findings.

For reverse MR analysis, we identified 139 SNPs as IVs for PrCa, with an *F*-statistic of 91.18, suggesting strong instruments (more details of IVs selection for PrCa are listed in **Supplementary Table 10**). As shown in **Figure 4** and **Supplementary Tables 11-12**, genetic predisposition to PrCa did not appear to affect any phenotype of COVID-19 in the reverse-direction MR (infection: *OR* = 1.00, 95%*CI* = 0.99-1.01, *P* = 0.66; hospitalization: *OR* = 0.99, 95%*CI* = 0.96-1.02, *P* = 0.34; critical illness: *OR* = 0.98, 95%*CI* = 0.94-1.02, *P* = 0.39). These results exclude reverse causality.

## Discussion

To the best of our knowledge, this is the first large-scale genome-wide cross-trait analysis that systematically investigates the shared genetic basis underlying COVID-19 and PrCa. Although we found no evidence of genome-wide genetic correlations, after partitioning the whole genome, we found local genetic correlations at chr1, chr7, and chr14. By using cross-trait meta-analysis, we identified 22 SNPs shared between COVID-19 and PrCa. TWAS revealed eight shared genes, mostly enriched in tissues of the adrenal gland, lung, brain, and testis. Finally, bidirectional MR identified no causal association between COVID-19 and PrCa. The findings of our study further demonstrated biological links underlying these two complex traits, highlighting shared mechanisms rather than potential causal associations.

In our study, while no overall genetic correlation between COVID-19 and PrCa was found, local genetic correlations were observed after partitioning the whole genome into independent regions, indicating a non-trivial shared biology underlying these two traits at the regional rather than genome-wide level. Specifically, we found four significant shared genomic regions, with two regions at 1q32.1 and 7q21.3-7q22.1 shared between PrCa and two COVID-19 phenotypes. 1q32.1 harbors *NFASC* 38, a previously reported risk gene for COVID-19, and* MDM4* 39, a previously reported risk gene for PrCa. 7q21.3-7q22.1 harbors *SGCE* 40 and *LMTK2* 41 previously reported as risk genes for COVID-19 and PrCa, respectively. Besides, the region at 7q11.23 was previously found to map several risk genes independently associated with COVID-19 38 and testosterone levels 42, while another region at 14q32.31-q32.33 was previously found to map several risk genes only associated with androgen-related male-pattern baldness 43. Altogether, these local genetic correlations further validate the notion that the genome-wide genomic approach may fail to detect association signals at the local level. This phenomenon is not uncommon in complex trait studies and may reflect the effects of epigenetic regulation, gene-gene, or gene-environment interactions that are not captured by global analysis. Moreover, the findings suggest a shared genetic basis underlying COVID-19 and PrCa, which is either directly through variants affecting both traits (horizontal pleiotropy) or through the causal effect of one trait on the other (vertical pleiotropy) 44.

In the downstream analyses that were conducted to explore these alternatives, cross-trait meta-analysis identified 22 shared SNPs, indicating horizontal pleiotropy and shared biological mechanisms underlying COVID-19 and PrCa. Among shared genetic variants, SNP rs2854005 was shared between PrCa and all three COVID-19 phenotypes. The loci harbor ***HLA-B***, a gene previously found to be associated with the loss of protective genes and reduced cell-mediated immunity in elderly PrCa patients 45, as well as with the modulation of the clinical severity of COVID-19 46. Our results, supported by previous research, indicate that gene ***HLA-B*** may serve as a potential target for mechanistic studies or interventions. Additionally, SNP rs800507 was novel and mapped to *TRPS1* with strong evidence of colocalization (*PPH4* > 0.5), demonstrating etiological connections. ***TRPS1*** was found to be highly expressed in androgen-dependent prostate cancer (LNCaP-FGC) cells compared with androgen-independent prostate cancer (LNCaP-LNO) cells 47. Chang et al. suggested its expression was associated with the apoptotic process in the rat ventral prostate 48 and was repressed by androgen in LNCaP-FGC cells, while was hardly detectable in LNCaP-LNO cells 49. In addition, its overexpression in LNCaP-FGC cells also decreased androgen-induced prostate-specific antigen expression 50. All these studies suggest that *TRPS1* is androgen-repressible and involved in apoptosis, thus a crucial regulator in the development of PrCa. However, its role in COVID-19 has not been clarified, making it a potential key gene for future mechanistic studies on COVID-19 sequelae. Notably, androgens can influence the breadth of immune response by altering the activity of specific immune subsets (e.g. myeloid cells, dendritic cells) directly involved in viral clearance 51. In addition, an androgen-responsive gene (i.e., *TMPRSS2*) was reported to play an important role in the initiation and progression of PrCa 19, as well as in the entry of human coronaviruses (e.g., SARS-CoV-2) into host cells 20.

Integrating GWAS and GTEx tissue-specific expression data, our TWAS analysis further revealed biological pleiotropy at a gene-tissue pair level. TWAS revealed eight genes shared by PrCa with at least one COVID-19 phenotype, largely enriched in the kidney cortex, adrenal gland, lung, brain, and testis. Among them, *CCHCR1* was shared by PrCa with all three COVID-19 phenotypes, highlighting its significance in our study. ***CCHCR1*** is a centrosome and processing body-localized protein regulating various cellular functions, including steroidogenesis that occurs in the adrenal cortex and testis 52. Steroid exerts a significant effect on the T cell-mediated immune response, especially CD4+ T cells responsible for infectious immunity, making patients more susceptible to opportunistic infections 53. *CCHCR1* was found to be a testis-specific expression 21 and associated with androgen-related alopecia areata 54. Androgen signaling usually targets the lung to enhance the expression of *TMPRSS2* 55. Although the specific role of *CCHCR1* between COVID-19 and PrCa has not been reported, this evidence suggests possible mechanisms involving the combination of immunosuppression and androgens.

In contrast, a two-sample MR analysis was performed to investigate vertical pleiotropy, but no significant association between genetically predicted COVID-19 and PrCa was found, consistent with a previous MR study 16. The COVID-19 pandemic has affected PrCa screening 56. Our findings may suggest that transient infection with SARS-CoV-2 is very unlikely to increase the risk of PrCa development, further implying that extra PrCa screening of COVID-19 patients may have a minimal public health benefit. Similarly, reverse MR analysis showed that genetically predicted PrCa did not affect any phenotypes of COVID-19, aligning with results from previous MR studies 15, 16. This finding may suggest that PrCa does not causally increase the risk of COVID-19, excluding potential reverse causality and indicating that androgen deprivation therapy (ADT), a typical PrCa treatment, may not be beneficial in reducing COVID-19 risk. For instance, one study found PrCa patients receiving ADT were significantly less vulnerable to COVID-19 than those not receiving ADT (*OR* = 4.05, 95%*CI* = 1.55-10.59) 10; however, other studies reported no significant differences 57, 58. Overall, these negative findings may suggest that COVID-19 has no long-term effect on PrCa; nevertheless, caution is warranted and further validation is needed when larger GWASs become available.

We acknowledge several potential limitations of our study. **First**, our analyses were restricted to European populations due to limited sample sizes and power in other ethnic groups. For example, standard clumping applied to East Asian GWASs yielded only one or no valid IVs, rendering MR analysis infeasible. While this limits generalizability, it helps reduce population stratification, and future studies in more diverse populations are needed for validation. **Second,** sex-combined GWAS summary data for COVID-19 phenotypes were used due to the unavailability of sex-specific data, which may introduce sex heterogeneity and potentially distort true genetic associations. Furthermore, the relatively recent emergence of COVID-19 may limit the GWAS summary data in capturing different SARS-CoV-2 variants and the full spectrum of genetic variations. **Third,** as most of the analytical software adopted in our study does not support the management and analysis of sex chromosomes, only autosomal data were included (except for MR analysis). **Fourth,** the power of our MR analysis may still be limited by sample size, case proportion, and heritability of IVs, leading to overall negative findings. Future larger GWASs with more powerful IVs are needed to validate our findings. **Fifth**, while our study identified multiple genes potentially relevant to COVID-19 and PrCa, their functional implications remain speculative. More experimental research is needed to understand the underlying pathophysiological mechanisms. **Finally,** the lack of individual-level data limits the exploration of gene-environment interactions, which should be an important focus for future research once such data become available. Despite the current limitations, our findings suggest promising avenues for further exploration.

Taken together, our study extends beyond conventional MR by implementing a comprehensive cross-trait analytical framework - including LDSC, SUPERGNOVA, cross-trait meta-analysis, TWAS, and MR - based on the most recent and well-powered GWAS data. We found no evidence supporting a direct causal effect of COVID-19 on PrCa, suggesting limited benefit from additional PrCa screening among COVID-19 survivors. Moreover, we identified several shared genes, such as *HLA-B* and *CCHCR1*, previously implicated in either COVID-19 or PrCa, which support the robustness of our findings and point to potential targets for diagnosis and therapy. These findings deepen our understanding of the genetic overlap and causal relationship between COVID-19 and PrCa, providing a foundation for future mechanistic studies. Further validation using large, sex-stratified, and multi-ethnic datasets is warranted.

## Conclusions

In conclusion, our study demonstrates shared genetic bases between COVID-19 phenotypes and PrCa, driven by significant local genetic correlations, pleiotropic SNPs, and shared genes. These findings highlight common biological mechanisms rather than direct causal relationships, suggesting the limited benefits of additional PrCa screening for COVID-19 survivors. Furthermore, the identified shared genes may serve as promising candidates for future mechanistic research and clinical interventions. Validation in larger and more diverse datasets is required to confirm these findings.

## Supplementary Material

Supplementary tables.

## Figures and Tables

**Table 1:** Genome-wide genetic correlation between prostate cancer and COVID-19 phenotypes

Trait 1	Trait 2	*r_g_*	*r_g_*_*SE*	*P*-value
Prostate cancer	SARS-CoV-2 infection	-0.06	0.05	0.20
Prostate cancer	COVID-19 hospitalization	-0.04	0.04	0.27
Prostate cancer	COVID-19 critical illness	-0.04	0.03	0.20

Note: *r_g_*: genetic correlation; *SE*: standard error; Infection: reported SARS-CoV-2 infection vs. population; Hospitalization: hospitalized COVID-19 patients vs. population; Critical illness: very severe respiratory confirmed COVID-19 patients vs. population.

**Table 2:** TWAS-identified shared gene-tissue pairs between COVID-19 and prostate cancer.

Gene	ID	Tissue Type	CHR	No. SNPs	Prostate cancer			COVID-19		
					BEST.GWAS.ID	TWAS.*Z*	*P*_Bonferroni_	BEST.GWAS.ID	TWAS.*Z*	*P*_Bonferroni_
**Prostate cancer and infection**										
CCHCR1	ENSG00000204536.13	Kidney Cortex	6	171	rs1265087	-4.19	2.75E-05	rs3130981	4.23	2.37E-05
**Prostate cancer and hospitalization**										
TCF19	ENSG00000137310.11	Artery Tibial	6	174	rs1265087	-6.44	1.16E-10	rs2844623	4.96	7.03E-07
TCF19	ENSG00000137310.11	Brain Hippocampus	6	172	rs1265087	-6.41	1.46E-10	rs2844623	5.16	2.46E-07
TCF19	ENSG00000137310.11	Brain Substantia nigra	6	173	rs1265087	-6.95	3.70E-12	rs2844623	4.85	1.21E-06
TCF19	ENSG00000137310.11	Testis	6	174	rs1265087	-5.68	1.37E-08	rs2844623	4.79	1.67E-06
TCF19	ENSG00000137310.11	Whole Blood	6	174	rs1265087	-4.92	8.81E-07	rs2844623	4.87	1.12E-06
ADAM15	ENSG00000143537.13	Adipose Subcutaneous	1	379	rs3753639	4.72	2.34E-06	rs35154152	4.85	1.24E-06
CYP21A1P	ENSG00000204338.8	Brain Putamen basal ganglia	6	197	rs1144708	5.82	6.06E-09	rs2515919	-4.45	8.77E-06
HLA-C	ENSG00000204525.16	Minor Salivary Gland	6	204	rs1265087	-5.48	4.17E-08	rs2844623	5.09	3.52E-07
HLA-C	ENSG00000204525.16	Small Intestine Terminal Ileum	6	204	rs1265087	-4.80	1.55E-06	rs2844623	5.47	4.60E-08
CCHCR1	ENSG00000204536.13	Adrenal Gland	6	174	rs1265087	-5.76	8.34E-09	rs2844623	5.82	6.00E-09
CCHCR1	ENSG00000204536.13	Brain Cortex	6	173	rs1265087	-6.60	4.18E-11	rs2844623	4.95	7.60E-07
CCHCR1	ENSG00000204536.13	Brain Putamen basal ganglia	6	174	rs1265087	-7.30	2.86E-13	rs2844623	4.66	3.24E-06
CCHCR1	ENSG00000204536.13	Breast Mammary Tissue	6	173	rs1265087	-6.50	7.86E-11	rs2844623	4.63	3.74E-06
CCHCR1	ENSG00000204536.13	Colon Sigmoid	6	174	rs1265087	-6.57	4.99E-11	rs2844623	4.92	8.72E-07
CCHCR1	ENSG00000204536.13	Esophagus Gastroesophageal Junction	6	174	rs1265087	-4.54	5.70E-06	rs2844623	5.05	4.46E-07
CCHCR1	ENSG00000204536.13	Esophagus Muscularis	6	174	rs1265087	-4.63	3.73E-06	rs2844623	4.62	3.87E-06
CCHCR1	ENSG00000204536.13	Heart Atrial Appendage	6	172	rs1265087	-6.79	1.11E-11	rs2844623	5.10	3.40E-07
CCHCR1	ENSG00000204536.13	Kidney Cortex	6	171	rs1265087	-4.19	2.75E-05	rs2844623	4.19	2.77E-05
CCHCR1	ENSG00000204536.13	Liver	6	174	rs1265087	-7.06	1.64E-12	rs2844623	4.99	5.92E-07
CCHCR1	ENSG00000204536.13	Lung	6	172	rs1265087	-6.38	1.76E-10	rs2844623	5.63	1.80E-08
CCHCR1	ENSG00000204536.13	Minor Salivary Gland	6	174	rs1265087	-7.30	2.86E-13	rs2844623	4.66	3.24E-06
CCHCR1	ENSG00000204536.13	Pancreas	6	173	rs1265087	-7.30	2.86E-13	rs2844623	4.66	3.24E-06
CCHCR1	ENSG00000204536.13	Pituitary	6	174	rs1265087	-7.28	3.38E-13	rs2844623	4.67	3.02E-06
CCHCR1	ENSG00000204536.13	Prostate	6	173	rs1265087	-7.30	2.86E-13	rs2844623	4.66	3.24E-06
CCHCR1	ENSG00000204536.13	Spleen	6	175	rs1265087	-6.85	7.19E-12	rs2844623	4.96	6.93E-07
CCHCR1	ENSG00000204536.13	Stomach	6	174	rs1265087	-7.30	2.86E-13	rs2844623	4.66	3.24E-06
CCHCR1	ENSG00000204536.13	Uterus	6	173	rs1265087	-7.30	2.86E-13	rs2844623	4.66	3.24E-06
CCHCR1	ENSG00000204536.13	Vagina	6	173	rs1265087	-7.30	2.86E-13	rs2844623	4.66	3.24E-06
HCP5	ENSG00000206337.10	Spleen	6	275	rs1265087	7.30	2.86E-13	rs2844623	-4.66	3.24E-06
ATF6B	ENSG00000213676.10	Heart Atrial Appendage	6	163	rs1144708	4.51	6.56E-06	rs660895	-4.70	2.64E-06
ATF6B	ENSG00000213676.10	Skin Sun Exposed Lower leg	6	163	rs1144708	4.63	3.63E-06	rs660895	-4.63	3.57E-06
-	ENSG00000237285.1	Testis	6	171	rs3891175	5.72	1.06E-08	rs660895	-4.63	3.64E-06
-	ENSG00000272501.1	Brain Putamen basal ganglia	6	174	rs1265087	-4.73	2.24E-06	rs2844623	6.47	9.55E-11
-	ENSG00000272501.1	Kidney Cortex	6	171	rs1265087	-6.56	5.29E-11	rs2844623	4.68	2.94E-06
-	ENSG00000280287.1	Cells EBV-transformed lymphocytes	12	299	rs11146929	4.57	4.90E-06	rs12821205	-5.11	3.15E-07
**Prostate cancer and critical illness**										
TCF19	ENSG00000137310.11	Artery Tibial	6	174	rs1265087	-6.44	1.16E-10	rs3130981	4.90	9.76E-07
TCF19	ENSG00000137310.11	Brain Hippocampus	6	172	rs1265087	-6.41	1.46E-10	rs3130981	5.06	4.27E-07
TCF19	ENSG00000137310.11	Whole Blood	6	174	rs1265087	-4.92	8.81E-07	rs3130981	4.80	1.55E-06
ADAM15	ENSG00000143537.13	Testis	1	379	rs3753639	5.10	3.35E-07	rs35154152	5.09	3.63E-07
HLA-C	ENSG00000204525.16	Minor Salivary Gland	6	204	rs1265087	-5.48	4.17E-08	rs3130981	5.05	4.53E-07
HLA-C	ENSG00000204525.16	Small Intestine Terminal Ileum	6	204	rs1265087	-4.80	1.55E-06	rs3130981	4.92	8.85E-07
CCHCR1	ENSG00000204536.13	Adrenal Gland	6	174	rs1265087	-5.76	8.34E-09	rs3130981	5.58	2.44E-08
CCHCR1	ENSG00000204536.13	Breast Mammary Tissue	6	173	rs1265087	-6.50	7.86E-11	rs3130981	4.52	6.17E-06
CCHCR1	ENSG00000204536.13	Colon Sigmoid	6	174	rs1265087	-6.57	4.99E-11	rs3130981	4.98	6.24E-07
CCHCR1	ENSG00000204536.13	Esophagus Gastroesophageal Junction	6	174	rs1265087	-4.54	5.70E-06	rs3130981	5.27	1.35E-07
CCHCR1	ENSG00000204536.13	Esophagus Muscularis	6	174	rs1265087	-4.63	3.73E-06	rs3130981	5.28	1.30E-07
CCHCR1	ENSG00000204536.13	Heart Atrial Appendage	6	172	rs1265087	-6.79	1.11E-11	rs3130981	5.08	3.68E-07
CCHCR1	ENSG00000204536.13	Heart Left Ventricle	6	174	rs1265087	-4.70	2.63E-06	rs3130981	4.54	5.60E-06
CCHCR1	ENSG00000204536.13	Kidney Cortex	6	171	rs1265087	-4.19	2.75E-05	rs3130981	4.72	2.37E-06
CCHCR1	ENSG00000204536.13	Liver	6	174	rs1265087	-7.06	1.64E-12	rs3130981	4.56	5.05E-06
CCHCR1	ENSG00000204536.13	Lung	6	172	rs1265087	-6.38	1.76E-10	rs3130981	5.42	6.11E-08
CCHCR1	ENSG00000204536.13	Spleen	6	175	rs1265087	-6.85	7.19E-12	rs3130981	4.56	5.11E-06
CCHCR1	ENSG00000204536.13	Uterus	6	173	rs1265087	-7.30	2.86E-13	rs3130981	4.29	1.78E-05
CCHCR1	ENSG00000204536.13	Vagina	6	173	rs1265087	-7.30	2.86E-13	rs3130981	4.29	1.78E-05
HLA-DQB2	ENSG00000232629.8	Brain Cortex	6	259	rs3891175	4.71	2.51E-06	rs660895	-4.49	7.21E-06
-	ENSG00000272501.1	Brain Putamen basal ganglia	6	174	rs1265087	-4.73	2.24E-06	rs3130981	6.77	1.25E-11
-	ENSG00000272501.1	Kidney Cortex	6	171	rs1265087	-6.56	5.29E-11	rs3130981	4.85	1.23E-06
-	ENSG00000280287.1	Cells EBV-transformed lymphocytes	12	299	rs11146929	4.57	4.90E-06	rs10870551	-6.39	1.70E-10

Infection: reported SARS-CoV-2 infection vs. population; Hospitalization: hospitalized COVID-19 patients vs. population; Critical illness: very severe respiratory confirmed COVID-19 patients vs. population; TWAS: transcriptome-wide association study; ID: identifier; CHR: Chromosome; SNP: single nucleotide polymorphism; No. SNPs: number of SNPs in the locus; GWAS: genome-wide association study; *Z*: Z value for TWAS.
