# Supplementary material for: Local Genetic Correlations and Pleiotropy Reveal Shared Genetic Architecture Between COVID-19 Phenotypes and Prostate Cancer in European Populations
Source: J Cancer. 2025 Oct 10;16(14):4245–56. doi: 10.7150/jca.111126 (PMC12595267; doi:10.7150/jca.111126)
Supplement: Supplementary file 1 — Supplementary tables. [file jcav16p4245s1.pdf]

Supplementary Table 1. Details of GWAS summary data.

Phenotype	N_{cases}	$N_{controls}$	N_{total}	Heritibility (standard error)	Ethnicity	Consortium	Year	Data source
Prostate cancer	79148	61106	140254	0.1663 (0.0198)	European	PRACTICAL	2018	PMID: 29892016
**COVID-19 phenotypes**								
Infection	122616	2475240	2597856	0.0111 (0.0016)	European	HGI	2022	https://www.covid19hg.org
Hospitalization	32519	2062805	2095324	0.0578 (0.0089)	European	HGI	2022	https://www.covid19hg.org
Critical illness	13769	1072442	1086211	0.1247 (0.0233)	European	HGI	2022	https://www.covid19hg.org

Infection: reported SARS-CoV-2 infection vs. population; Hospitalization: hospitalized COVID-19 patients vs. population; Critical illness: very severe respiratory confirmed COVID-19 patients vs. population; N: sample size; PRACTICAL: Prostate Cancer Association Group to Investigate Cancer Associated Alterations in the Genome consortium; HGI: COVID-19 Host Genetics Initiative.

Supplementary Table 2. Results from local genetic correlation analysis conducted for prostate cancer and COVID-19 phenotypes.

Chromosome	Start position	End position	Number of SNPs	Local genetic covariance	Variance	P-value
**Prostate cancer and SARS-CoV-2 infection**						
1	1103150	2320702	361	-9.51E-06	8.21E-10	0.740
1	2321099	3065568	234	4.20E-06	5.42E-10	0.857
1	3066761	3679461	290	-1.62E-04	1.95E-09	0.000
1	3679775	4749076	633	-3.65E-05	1.75E-09	0.383
1	4750013	5000086	168	-9.40E-06	4.71E-10	0.665
1	5000970	5471517	301	2.62E-05	8.33E-10	0.364
1	5472626	6133700	338	4.16E-05	8.49E-10	0.154
1	6134990	7245669	447	-2.21E-07	9.35E-10	0.994
1	7245922	7604778	205	-1.15E-05	5.20E-10	0.615
1	7604831	9067142	513	2.29E-05	1.11E-09	0.491
1	9067673	9959051	319	2.66E-05	1.03E-09	0.406
1	9959748	11774956	584	2.01E-05	1.45E-09	0.598
1	11776196	12147753	192	6.05E-06	3.79E-10	0.756
1	12150385	14625524	731	1.49E-05	2.05E-09	0.743
1	14626896	15423481	421	-4.78E-05	1.28E-09	0.182
1	15424441	17123862	613	-5.83E-05	1.52E-09	0.134
1	17176635	17872659	337	-4.41E-06	6.81E-10	0.866
1	17874843	18484945	341	-8.65E-06	9.56E-10	0.780
1	18486272	20022063	799	-1.24E-05	2.03E-09	0.783
1	20023833	20763899	370	-1.90E-05	1.16E-09	0.577
1	20765245	22634131	796	2.56E-05	1.82E-09	0.549
1	22636103	23082667	208	-2.05E-06	5.54E-10	0.931
1	23085227	24527501	491	-2.07E-05	1.13E-09	0.537
1	24528210	25305673	317	-1.42E-06	7.40E-10	0.958
1	25305783	26976424	612	3.31E-05	1.18E-09	0.335
1	26981479	28160199	205	3.98E-05	6.06E-10	0.106
1	28168872	29181525	242	2.23E-05	3.93E-10	0.261
1	29184254	30792409	735	-1.89E-05	1.55E-09	0.631
1	30792984	32042421	610	3.30E-05	1.02E-09	0.301
1	32042540	34016219	486	8.23E-06	8.12E-10	0.773
1	34017349	34797348	442	-2.23E-05	1.03E-09	0.486
1	34798014	35364965	274	-2.68E-05	7.79E-10	0.337
1	35366056	38196841	762	3.45E-05	1.64E-09	0.393
1	38459210	39535259	436	-4.31E-07	1.20E-09	0.990

1	39537291	40933221	445	1.03E-04	8.73E-10	0.001
1	40934284	42166782	487	-6.85E-05	9.67E-10	0.028
1	42168539	43365094	396	-2.17E-05	6.67E-10	0.400
1	43365565	44482312	462	7.24E-06	7.64E-10	0.793
1	44483704	46895641	764	5.63E-05	1.06E-09	0.083
1	46897698	47999344	386	1.04E-05	6.59E-10	0.686
1	48000156	50640662	790	1.21E-05	1.33E-09	0.741
1	50641944	53116453	445	3.88E-05	7.37E-10	0.153
1	53117066	54197141	419	1.49E-05	8.10E-10	0.600
1	54197688	55012825	295	-2.95E-05	7.50E-10	0.281
1	55012881	55479198	250	1.18E-05	6.50E-10	0.643
1	55479967	56413577	485	2.18E-05	1.03E-09	0.497
1	56414730	57585670	599	1.50E-05	1.23E-09	0.669
1	57585980	58535140	555	2.98E-05	1.02E-09	0.350
1	58536417	59306823	412	-5.48E-06	7.96E-10	0.846
1	59307290	59736866	187	-8.15E-06	4.67E-10	0.706
1	59739179	61257170	537	-4.87E-05	1.10E-09	0.141
1	61258378	62107806	382	5.00E-06	1.26E-09	0.888
1	62108681	63376404	518	-8.69E-06	1.05E-09	0.788
1	63379031	65008206	795	2.66E-05	1.75E-09	0.525
1	65010679	66773349	698	-1.16E-05	1.39E-09	0.756
1	66774350	67760291	537	2.23E-05	9.77E-10	0.475
1	67761365	69441414	724	-4.56E-06	1.62E-09	0.910
1	69441920	70486660	328	3.28E-05	8.30E-10	0.255
1	70490925	71818029	502	6.49E-06	1.00E-09	0.838
1	71822765	74326378	643	9.37E-06	1.04E-09	0.771
1	74326484	75992462	563	-2.14E-05	9.74E-10	0.493
1	75992778	76476360	183	-6.83E-06	2.81E-10	0.684
1	76477207	77139859	372	-2.70E-05	8.81E-10	0.364
1	77141039	79111862	686	2.57E-06	1.37E-09	0.945
1	79113455	80341320	483	-1.37E-05	8.95E-10	0.646
1	80341732	81056946	241	-1.34E-05	4.71E-10	0.536
1	81058295	82883310	839	-5.19E-06	1.92E-09	0.906
1	82884721	83990906	441	1.88E-07	8.59E-10	0.995
1	83991748	84728665	328	8.18E-06	5.79E-10	0.734
1	84729134	85940249	595	-4.22E-06	1.35E-09	0.908
1	85940875	86549498	270	1.53E-06	4.93E-10	0.945

1	86549569	87652502	417	-1.21E-05	8.34E-10	0.676
1	87654606	89447978	634	-2.79E-06	1.49E-09	0.942
1	89451664	91866856	883	1.46E-05	1.79E-09	0.729
1	91867046	93351477	560	9.74E-06	1.01E-09	0.759
1	93352491	94451007	318	-9.02E-08	4.91E-10	0.997
1	94452062	95199048	406	-7.27E-06	8.63E-10	0.804
1	95199771	96149941	435	4.03E-06	9.53E-10	0.896
1	96150318	97457893	467	5.00E-06	8.18E-10	0.861
1	97458722	98562260	517	-3.17E-05	7.76E-10	0.255
1	98564736	100064599	575	1.14E-05	1.24E-09	0.746
1	100065413	101682573	614	-5.62E-06	9.76E-10	0.857
1	101683165	102039858	221	6.85E-06	4.94E-10	0.758
1	102040268	102893372	341	-1.06E-05	7.02E-10	0.689
1	102894567	104369301	288	2.18E-06	5.80E-10	0.928
1	104373157	105192916	270	1.12E-06	5.81E-10	0.963
1	105193022	106641877	398	3.75E-05	7.74E-10	0.178
1	106642705	107432265	291	3.87E-06	5.63E-10	0.871
1	107432576	109075191	693	2.22E-05	1.63E-09	0.582
1	109075561	110301260	464	-2.14E-06	9.07E-10	0.943
1	110302505	111738116	679	-2.21E-05	1.56E-09	0.577
1	111739749	112560278	566	-3.77E-05	1.33E-09	0.302
1	112561693	114451307	682	3.85E-07	1.09E-09	0.991
1	114451386	115878242	635	-3.91E-05	1.30E-09	0.278
1	115879821	116884431	399	8.26E-06	8.34E-10	0.775
1	116884519	118837474	650	-3.21E-05	1.37E-09	0.386
1	118837888	119792751	365	-2.15E-06	6.61E-10	0.933
1	119793605	120347239	234	1.13E-05	5.60E-10	0.634
1	144998563	147347713	453	-4.35E-06	8.42E-10	0.881
1	147348339	151830844	601	-4.24E-06	2.11E-09	0.926
1	151832901	152535021	216	7.60E-06	3.10E-10	0.666
1	152535586	153163377	278	-6.69E-06	3.77E-10	0.731
1	153165450	154544651	506	2.55E-05	7.73E-10	0.359
1	154547292	156334116	551	-1.83E-05	3.97E-09	0.772
1	156335111	157154186	313	-4.52E-06	6.45E-10	0.859
1	157154324	158867538	820	2.16E-05	1.73E-09	0.603
1	158870085	160648549	764	1.02E-05	1.87E-09	0.813
1	160649926	161973976	510	5.19E-05	1.05E-09	0.109

1	161974584	162995355	563	-3.35E-05	1.20E-09	0.333
1	163000394	164410254	676	3.13E-06	1.22E-09	0.929
1	164412847	165190876	436	2.77E-05	1.20E-09	0.424
1	165191702	166363258	605	2.68E-05	1.38E-09	0.470
1	166364063	167158927	484	2.63E-06	9.12E-10	0.931
1	167159608	168168773	462	1.39E-05	1.02E-09	0.664
1	168169452	169077127	489	6.47E-06	1.23E-09	0.854
1	169078572	169521853	193	-4.68E-07	2.91E-10	0.978
1	169522554	170372503	446	-7.73E-06	7.82E-10	0.782
1	170375663	170914261	181	7.56E-06	3.73E-10	0.696
1	170915133	172647523	864	3.29E-05	1.46E-09	0.390
1	172648956	175384090	909	4.47E-07	1.36E-09	0.990
1	175385076	176416712	369	9.46E-06	6.81E-10	0.717
1	176418578	177431255	438	-1.09E-05	8.09E-10	0.701
1	177432237	178940717	621	-1.53E-05	1.17E-09	0.654
1	178943012	179676274	338	6.48E-07	5.36E-10	0.978
1	179678188	180567596	397	-2.80E-05	7.66E-10	0.311
1	180568720	181311800	309	-8.85E-07	5.60E-10	0.970
1	181313404	182293001	421	2.21E-05	8.73E-10	0.454
1	182294372	183796074	577	-3.50E-05	9.52E-10	0.256
1	183796618	185511423	737	5.32E-05	1.52E-09	0.173
1	185512838	187847742	807	-2.49E-05	1.43E-09	0.510
1	187848898	189756187	599	8.06E-06	8.78E-10	0.786
1	189758772	190107648	158	5.30E-06	2.64E-10	0.744
1	190107883	191432168	473	2.99E-05	7.56E-10	0.276
1	191433917	192444256	331	-8.97E-06	5.60E-10	0.705
1	192452631	193504005	346	1.73E-05	5.47E-10	0.460
1	193505535	194000243	197	-6.27E-07	3.33E-10	0.973
1	194000953	194733444	212	-1.20E-05	3.49E-10	0.521
1	194734415	195155161	176	4.51E-06	2.85E-10	0.789
1	195155293	195995510	312	3.37E-07	6.20E-10	0.989
1	195995948	197309603	347	2.13E-06	5.90E-10	0.930
1	197310096	198540028	417	-1.74E-05	6.91E-10	0.507
1	198540418	199578700	369	-9.05E-06	7.33E-10	0.738
1	199580285	201070422	658	-8.19E-05	1.32E-09	0.024
1	201070740	201365889	257	2.40E-05	6.02E-10	0.327
1	201366509	202820077	608	-1.68E-05	1.38E-09	0.651

1	202820815	203979519	488	-4.94E-05	1.32E-09	0.173
1	203980019	205458538	791	-1.26E-04	2.26E-09	0.008
1	205459511	206705609	336	7.76E-05	1.58E-09	0.051
1	206706419	208158787	548	2.76E-05	1.08E-09	0.400
1	210741816	211763053	432	5.35E-06	1.00E-09	0.866
1	208160220	210738604	1288	1.17E-05	2.84E-09	0.826
1	211763754	213578006	641	-5.38E-05	1.39E-09	0.149
1	213578592	215128675	756	1.80E-05	1.71E-09	0.663
1	215129087	216672805	623	1.84E-05	1.20E-09	0.597
1	216673326	217575665	684	-4.30E-08	1.59E-09	0.999
1	217577346	218860068	520	-1.84E-06	1.24E-09	0.958
1	218860456	220068681	530	-1.49E-05	9.61E-10	0.630
1	220069681	221856899	640	4.13E-05	1.25E-09	0.243
1	221857359	223630391	645	-1.93E-05	1.54E-09	0.623
1	223630619	224434769	280	-5.47E-06	5.89E-10	0.822
1	224435934	226143348	570	-7.30E-06	1.09E-09	0.825
1	226146348	226808294	242	-1.91E-05	5.23E-10	0.403
1	226808697	228100891	558	1.03E-05	1.00E-09	0.744
1	228101119	229075210	320	-4.60E-05	7.46E-10	0.092
1	229076157	230477970	634	3.83E-06	1.35E-09	0.917
1	230479324	231318678	503	2.03E-05	1.25E-09	0.565
1	231321215	232382234	476	2.53E-05	9.72E-10	0.418
1	232384356	232949914	349	-2.28E-05	8.14E-10	0.424
1	232950888	233750725	429	-3.76E-05	1.02E-09	0.239
1	233753991	234206593	224	2.48E-05	6.44E-10	0.330
1	234209087	235772624	702	2.34E-05	1.78E-09	0.579
1	235773790	236536723	314	2.53E-06	6.17E-10	0.919
1	236537770	237493854	566	2.89E-06	1.17E-09	0.933
1	237495080	239297872	909	-2.33E-05	2.21E-09	0.621
1	239299272	240562764	672	-2.68E-05	1.65E-09	0.509
1	240563367	241178287	352	-3.10E-05	1.14E-09	0.360
1	241178944	241766551	357	-8.02E-06	1.01E-09	0.800
1	241768617	242697031	497	-1.24E-05	1.37E-09	0.738
1	242700803	244105053	454	1.97E-05	7.69E-10	0.477
1	244106113	244927751	383	-2.84E-05	1.08E-09	0.387
1	244929262	246606531	851	5.38E-05	2.08E-09	0.238
1	246606926	246989171	161	-1.30E-05	5.36E-10	0.575

1	246991094	247838628	369	-1.79E-05	9.01E-10	0.552
1	247838870	248585259	281	1.14E-05	5.18E-10	0.616
2	11320	1158981	538	2.30E-05	1.20E-09	0.508
2	1159982	1681720	220	-1.56E-05	5.97E-10	0.523
2	1683187	3003071	698	7.08E-05	2.02E-09	0.115
2	3003472	4148069	589	-1.62E-05	1.54E-09	0.681
2	4148784	5017172	423	-8.02E-06	1.08E-09	0.807
2	5118816	6361343	633	-5.04E-05	1.95E-09	0.254
2	6363082	7322437	569	-3.84E-05	1.52E-09	0.324
2	7323039	8980346	703	-1.96E-06	1.92E-09	0.964
2	8980472	10440987	658	-7.80E-05	2.57E-09	0.124
2	10442541	10879340	236	6.03E-05	6.40E-10	0.017
2	10881867	11652586	377	6.36E-05	1.46E-09	0.096
2	11652795	12720977	565	-1.85E-05	1.81E-09	0.663
2	12723749	14648908	706	3.65E-05	1.62E-09	0.364
2	14649571	15720775	452	-2.42E-05	8.70E-10	0.412
2	15721423	16549199	386	4.63E-06	1.11E-09	0.890
2	16549538	18344242	768	7.97E-05	1.72E-09	0.055
2	18344821	18646822	144	-1.17E-05	3.38E-10	0.526
2	18647868	20059542	690	1.70E-05	1.64E-09	0.674
2	20062043	22427190	825	5.09E-05	2.63E-09	0.321
2	22428680	23855977	652	2.27E-05	1.58E-09	0.569
2	23857184	25485735	455	-2.48E-05	8.13E-10	0.384
2	25486770	26894217	438	-8.25E-06	9.71E-10	0.791
2	26894589	28597305	482	-2.33E-06	7.76E-10	0.933
2	28597624	29484922	455	5.42E-05	1.06E-09	0.095
2	29486078	29987735	338	-2.44E-06	7.69E-10	0.930
2	29988753	31009977	499	-1.35E-05	1.22E-09	0.700
2	31010212	32489851	591	-1.34E-05	1.22E-09	0.700
2	32493210	33361425	336	4.23E-06	7.98E-10	0.881
2	33362643	34279720	532	8.10E-06	1.33E-09	0.824
2	34280335	35607183	529	-3.11E-05	1.15E-09	0.360
2	35608923	36121342	278	-4.41E-07	5.78E-10	0.985
2	36122006	37004185	554	-2.20E-06	1.48E-09	0.954
2	37008066	38882278	873	-5.81E-05	2.22E-09	0.218
2	38882577	40077971	314	-9.31E-07	5.41E-10	0.968
2	40080343	40625847	349	-2.24E-05	7.91E-10	0.425

2	40626336	41381901	294	-9.59E-06	5.87E-10	0.692
2	41382889	42087411	359	1.53E-06	7.40E-10	0.955
2	42088016	42947335	404	-2.73E-05	8.01E-10	0.335
2	42949616	43307776	218	-1.83E-05	2.01E-09	0.683
2	43309247	44048346	326	-8.88E-05	2.19E-09	0.058
2	44049120	45194289	539	-1.50E-05	1.14E-09	0.656
2	45195022	45562814	268	-6.08E-06	9.38E-10	0.843
2	45563613	47052067	882	-6.36E-06	2.41E-09	0.897
2	47052788	47782756	370	-2.82E-05	1.16E-09	0.407
2	47782836	49112501	508	-2.13E-05	1.34E-09	0.559
2	49113132	50757671	842	-1.62E-06	1.86E-09	0.970
2	50758657	51667413	400	-8.80E-06	8.63E-10	0.765
2	51668142	53351493	835	-4.01E-06	1.82E-09	0.925
2	53352175	54599930	554	-2.03E-05	1.26E-09	0.567
2	54600556	56158646	724	-8.66E-06	1.64E-09	0.830
2	56160647	56599354	240	-4.15E-06	7.25E-10	0.877
2	56600739	57938354	492	-3.69E-05	1.02E-09	0.249
2	57939866	60449069	1014	-2.27E-05	2.38E-09	0.642
2	60449710	62420656	658	4.55E-05	1.29E-09	0.206
2	62425639	64623935	687	2.52E-06	4.73E-09	0.971
2	64626895	65226463	256	1.24E-05	6.97E-10	0.640
2	65228236	66374803	559	-4.02E-06	1.27E-09	0.910
2	66375016	67978287	798	-1.44E-05	1.94E-09	0.743
2	67981487	68871573	395	-3.38E-06	8.54E-10	0.908
2	68871710	70753882	812	3.89E-05	1.64E-09	0.337
2	70756057	71704491	467	-2.08E-05	1.19E-09	0.545
2	71704844	73172972	469	3.07E-06	1.07E-09	0.925
2	73174848	74121000	254	2.50E-06	4.09E-10	0.901
2	74121278	76615798	1069	-2.84E-05	2.45E-09	0.566
2	76616819	77384991	368	-1.26E-05	8.54E-10	0.667
2	77386522	79007966	562	-1.13E-05	1.35E-09	0.758
2	80804991	81588311	231	6.88E-06	4.46E-10	0.745
2	79008155	80804284	1066	2.14E-05	2.67E-09	0.678
2	81588482	83604057	599	-4.90E-07	9.25E-10	0.987
2	83604796	85283713	481	7.72E-06	8.71E-10	0.794
2	85284353	86559358	572	-1.79E-05	1.22E-09	0.608
2	86562902	89135764	464	-1.79E-05	9.63E-10	0.565

2	92324281	98790763	461	1.10E-05	7.94E-10	0.696
2	98791433	100195879	407	-3.13E-05	6.65E-10	0.225
2	100197638	101338509	453	-8.70E-07	8.35E-10	0.976
2	101338644	102688272	529	7.03E-05	1.38E-09	0.058
2	102688495	103262415	357	2.51E-05	6.43E-10	0.322
2	103264434	104481488	294	-1.20E-05	6.12E-10	0.626
2	104485456	105871610	462	-1.46E-05	1.09E-09	0.658
2	105872982	107406122	724	-1.94E-05	1.92E-09	0.657
2	107406885	108427822	407	-2.04E-05	8.56E-10	0.486
2	108427959	110109210	583	4.18E-06	1.21E-09	0.905
2	110109222	112924907	587	5.74E-05	3.14E-09	0.305
2	112924973	114846282	718	-1.65E-06	1.49E-09	0.966
2	114848115	116770829	607	7.89E-06	1.30E-09	0.827
2	116772945	117727045	304	2.41E-06	5.22E-10	0.916
2	117728904	119581280	716	-1.69E-05	1.64E-09	0.676
2	119581439	121070811	505	-8.50E-06	1.08E-09	0.796
2	121073253	123241656	763	7.62E-05	1.91E-09	0.082
2	123244233	124730999	532	-9.11E-06	1.04E-09	0.777
2	124733246	125735879	428	-3.21E-06	9.95E-10	0.919
2	125741434	127372889	518	-2.24E-06	9.65E-10	0.943
2	127374341	129310292	756	-3.03E-05	1.78E-09	0.473
2	129312188	129864416	215	-3.02E-07	5.33E-10	0.990
2	129864730	131463038	407	8.06E-06	8.72E-10	0.785
2	131466136	133030349	226	-1.62E-05	5.60E-10	0.492
2	133034031	133950661	431	-2.48E-06	1.10E-09	0.941
2	133951544	136822267	1074	-5.83E-06	2.12E-09	0.899
2	136825272	138619715	738	-2.38E-05	1.60E-09	0.553
2	138620371	139412389	267	8.31E-06	5.22E-10	0.716
2	139413312	140902261	570	1.27E-05	1.27E-09	0.722
2	140902383	141803496	400	-1.24E-05	8.95E-10	0.679
2	141803602	143254981	699	2.95E-05	1.65E-09	0.468
2	143255262	145301314	618	-2.73E-05	1.66E-09	0.502
2	145303576	147252762	524	-4.20E-05	1.10E-09	0.205
2	147255216	149268931	497	1.98E-05	9.44E-10	0.519
2	149270710	151184680	609	-9.29E-06	1.37E-09	0.802
2	151191481	152490219	540	-7.09E-05	1.17E-09	0.038
2	152491269	153369892	311	-5.85E-06	5.74E-10	0.807

2	153371042	154414456	449	1.17E-05	1.14E-09	0.730
2	154415112	155208173	297	1.85E-08	6.78E-10	0.999
2	155209736	156568446	482	-1.71E-05	1.00E-09	0.590
2	156568806	158165818	441	-1.20E-06	1.07E-09	0.971
2	158173710	159574883	463	-1.89E-05	1.13E-09	0.574
2	159575554	161077823	603	-5.53E-06	1.23E-09	0.875
2	161078891	163358537	678	2.70E-05	1.18E-09	0.431
2	163359084	165200324	484	-6.71E-05	1.13E-09	0.046
2	165202539	167120956	737	6.30E-05	1.75E-09	0.132
2	167123247	168427789	400	1.46E-06	7.92E-10	0.959
2	168431583	169176766	322	-2.44E-05	8.68E-10	0.408
2	169178092	170973262	789	-2.85E-05	1.79E-09	0.500
2	170973461	172086127	479	-7.68E-06	1.21E-09	0.825
2	172088943	173586201	589	7.24E-05	3.26E-09	0.205
2	173588663	175585616	785	3.62E-05	2.38E-09	0.458
2	175586510	177326682	629	3.19E-05	1.47E-09	0.405
2	177327202	178551958	558	-8.07E-06	1.15E-09	0.812
2	178552788	180138313	565	-4.34E-05	1.18E-09	0.206
2	180139219	180661233	324	-7.65E-06	9.70E-10	0.806
2	180662102	181627195	413	-5.46E-05	9.62E-10	0.079
2	181628139	182840114	361	-2.75E-05	7.53E-10	0.316
2	182845657	184442530	601	1.36E-05	1.22E-09	0.698
2	184443166	185277576	257	6.83E-06	4.88E-10	0.757
2	185279087	186731282	380	-1.77E-05	6.77E-10	0.495
2	186731309	188540877	478	1.38E-05	8.21E-10	0.631
2	188542361	190794689	527	3.11E-05	1.04E-09	0.334
2	190797341	191870624	350	-5.01E-05	6.42E-10	0.048
2	191873553	192695107	283	-2.26E-05	7.95E-10	0.422
2	192696255	195109845	444	-2.77E-05	8.00E-10	0.328
2	195117932	197307918	619	-9.94E-06	1.28E-09	0.781
2	197308707	198140953	270	-1.30E-05	4.74E-10	0.550
2	198141746	200498970	621	9.35E-06	1.04E-09	0.772
2	200504209	201571624	452	-5.34E-06	9.09E-10	0.859
2	201572564	202829668	382	-6.51E-05	1.41E-09	0.083
2	202830132	204814896	525	2.33E-05	1.07E-09	0.475
2	204815902	206043539	517	8.98E-06	1.32E-09	0.805
2	206045927	207721890	728	4.23E-05	1.70E-09	0.305

2	207722425	208639154	389	6.15E-05	1.22E-09	0.079
2	208641174	209940909	408	3.76E-06	8.92E-10	0.900
2	209941332	210832981	269	-1.65E-06	5.01E-10	0.941
2	210838781	212377672	605	-6.38E-07	1.32E-09	0.986
2	212377759	212845716	216	-2.11E-05	6.25E-10	0.398
2	212845994	215005050	818	-2.42E-05	1.71E-09	0.557
2	215006557	216448007	604	-2.08E-05	1.35E-09	0.572
2	216449570	217184275	343	-2.12E-06	9.08E-10	0.944
2	217184715	218615634	620	-2.99E-05	1.50E-09	0.439
2	218616633	219671910	401	-1.62E-05	7.13E-10	0.543
2	219675067	220774229	409	2.26E-05	8.41E-10	0.436
2	220775971	221445218	359	-3.84E-05	8.93E-10	0.199
2	221445953	222985126	638	4.08E-05	1.58E-09	0.305
2	222985611	224002780	434	3.66E-05	1.05E-09	0.258
2	224003069	225832409	736	-8.25E-07	1.63E-09	0.984
2	225833758	227531180	618	-1.90E-05	1.23E-09	0.587
2	227532464	228456451	380	-5.65E-06	8.00E-10	0.842
2	228457117	229368530	433	-2.55E-06	1.09E-09	0.938
2	229368812	230282624	396	3.16E-05	9.65E-10	0.309
2	230283310	231322125	471	7.96E-05	1.25E-09	0.024
2	231322831	232790491	543	3.12E-05	1.46E-09	0.414
2	232791948	233964632	468	-3.98E-05	1.04E-09	0.217
2	233968153	234699046	368	1.71E-05	6.48E-10	0.503
2	234699366	235626316	630	-2.96E-05	1.77E-09	0.481
2	235627087	236619385	515	-8.00E-06	1.66E-09	0.845
2	236619713	238209382	608	1.77E-05	1.51E-09	0.649
2	238209871	239122917	445	-5.63E-05	1.31E-09	0.120
2	239123082	239801883	289	-1.72E-05	6.86E-10	0.512
2	239832097	240325991	242	1.21E-05	7.81E-10	0.664
2	240327154	240961852	336	-2.93E-05	1.19E-09	0.395
2	240962194	241907076	460	1.77E-05	1.55E-09	0.653
2	241909927	243185680	317	5.32E-05	1.77E-09	0.206
3	60197	1128944	484	3.35E-05	1.55E-09	0.394
3	1129028	1527748	339	2.49E-05	1.17E-09	0.467
3	1528147	2423237	601	-1.66E-05	1.73E-09	0.689
3	2423999	2769230	241	-1.52E-05	8.62E-10	0.604
3	2769392	3364279	476	1.09E-05	1.97E-09	0.806

3	3364442	4430024	630	1.57E-05	1.72E-09	0.706
3	4430848	5564422	653	5.35E-05	2.23E-09	0.257
3	5565384	6421659	513	2.74E-06	1.56E-09	0.945
3	6422814	7509288	575	8.91E-06	1.57E-09	0.822
3	7509715	7991133	371	9.26E-07	9.81E-10	0.976
3	7991981	9194649	780	-1.07E-04	2.26E-09	0.025
3	9195331	10526438	649	-1.53E-05	1.78E-09	0.716
3	10526803	11220278	388	1.61E-06	1.35E-09	0.965
3	11221721	12858028	673	5.10E-05	1.65E-09	0.210
3	12859004	13523338	332	4.05E-05	1.01E-09	0.202
3	13524741	14309204	367	-4.92E-07	1.11E-09	0.988
3	14309548	14816364	329	1.39E-05	1.08E-09	0.673
3	14816745	16661587	815	-7.64E-05	2.08E-09	0.094
3	16662488	18454062	540	-6.21E-06	1.29E-09	0.863
3	18457304	18886128	167	8.74E-07	3.95E-10	0.965
3	18886158	20752492	602	2.54E-05	1.61E-09	0.527
3	20753368	21522703	363	3.18E-05	1.10E-09	0.338
3	21523116	22102707	449	-1.41E-06	1.35E-09	0.969
3	22103146	23122359	464	-1.58E-05	1.25E-09	0.656
3	23123318	23802647	259	5.73E-06	7.11E-10	0.830
3	25460109	26392563	317	4.95E-06	7.15E-10	0.853
3	23803541	25459497	887	-2.95E-05	2.49E-09	0.554
3	26393305	27606695	467	1.29E-06	1.07E-09	0.969
3	27609447	29409547	794	6.22E-06	2.31E-09	0.897
3	29409589	30716602	620	-3.88E-06	1.85E-09	0.928
3	30717661	31720982	438	-5.47E-06	9.84E-10	0.862
3	31721774	33011510	533	6.54E-06	1.78E-09	0.877
3	33012981	33986974	341	1.55E-05	7.69E-10	0.575
3	33988115	35241960	454	5.23E-06	1.12E-09	0.875
3	35243764	36481385	482	-4.41E-05	1.13E-09	0.190
3	36484375	38078255	627	-6.57E-06	1.48E-09	0.864
3	38080101	38723967	263	2.75E-06	6.40E-10	0.913
3	38724849	40649760	701	-1.55E-05	1.54E-09	0.694
3	40660283	42184173	434	1.73E-05	9.52E-10	0.574
3	42184361	45162316	1057	5.85E-05	2.42E-09	0.234
3	45165430	46657162	653	2.46E-05	4.23E-09	0.705
3	46657428	48525955	411	1.82E-05	7.78E-10	0.515

3	48531227	52214640	856	1.71E-05	1.60E-09	0.670
3	52215002	54078568	705	9.46E-08	1.47E-09	0.998
3	54080241	55731145	920	3.32E-05	2.72E-09	0.525
3	55733390	58063819	873	-3.89E-05	1.92E-09	0.376
3	58067795	58700675	307	3.47E-05	7.01E-10	0.190
3	58700802	60347135	936	-2.96E-05	2.86E-09	0.580
3	60347701	61277928	614	4.94E-06	1.67E-09	0.904
3	61279726	62881664	855	1.54E-06	2.70E-09	0.976
3	62882619	63504979	310	2.59E-05	8.09E-10	0.363
3	63505191	64473972	483	-2.25E-05	1.41E-09	0.549
3	64475164	65337581	550	-1.66E-05	1.64E-09	0.683
3	65340365	66169323	368	-2.95E-05	1.20E-09	0.395
3	66170253	67294968	419	8.66E-06	1.13E-09	0.797
3	67299401	69197507	756	-4.74E-05	1.85E-09	0.271
3	69205190	70288126	413	9.59E-07	1.09E-09	0.977
3	70289919	71418327	409	-2.73E-06	1.73E-09	0.948
3	71421152	73238935	813	-5.32E-05	2.64E-09	0.301
3	73239448	75376291	877	-7.46E-06	2.39E-09	0.879
3	75377075	76854466	345	2.44E-06	8.25E-10	0.932
3	76855676	77507524	288	-1.86E-05	7.80E-10	0.506
3	77508835	79022673	488	1.13E-05	1.24E-09	0.749
3	79024541	81187867	525	-1.21E-06	1.07E-09	0.970
3	81190039	82446513	356	-4.22E-06	6.85E-10	0.872
3	82446746	83946780	382	1.91E-05	8.33E-10	0.509
3	83947451	85090935	310	-5.73E-07	6.89E-10	0.983
3	85093629	86734415	435	6.15E-05	8.54E-10	0.035
3	86736368	87407921	210	-5.96E-05	2.95E-09	0.273
3	87408634	88725583	376	-2.36E-05	8.00E-10	0.404
3	88725754	94202010	445	8.03E-07	9.74E-10	0.979
3	94202771	96056259	472	-7.27E-06	9.03E-10	0.809
3	96679063	98299365	463	8.55E-07	9.95E-10	0.978
3	98300731	100591006	762	1.08E-05	1.55E-09	0.784
3	100591848	101808006	473	1.68E-04	2.45E-09	0.001
3	101808379	103025517	322	-8.29E-07	7.34E-10	0.976
3	103025715	103698105	210	1.79E-05	4.84E-10	0.417
3	103700909	104579408	324	1.87E-05	7.76E-10	0.502
3	104580063	104878270	154	-1.14E-06	3.63E-10	0.952

3	104879008	106330349	517	3.27E-05	1.34E-09	0.373
3	106332831	107729547	480	1.10E-04	2.14E-09	0.018
3	107730020	108635884	340	-3.92E-06	8.20E-10	0.891
3	108636466	109961316	401	2.29E-05	8.65E-10	0.437
3	109962369	111416310	404	1.43E-05	8.81E-10	0.631
3	111416451	112182995	420	6.48E-06	9.91E-10	0.837
3	112183360	113323793	432	1.71E-06	1.35E-09	0.963
3	113324119	115280312	582	2.39E-05	1.52E-09	0.540
3	115282058	116450487	509	-1.79E-05	1.31E-09	0.621
3	116452274	117998840	678	3.73E-05	1.96E-09	0.399
3	120521733	121335461	214	-1.95E-06	4.24E-10	0.925
3	118000136	120520317	1012	-1.01E-04	2.39E-09	0.039
3	121336367	122692039	596	4.19E-05	1.35E-09	0.254
3	122693533	123196819	219	-6.75E-06	5.38E-10	0.771
3	123200440	124677278	597	-1.61E-06	1.46E-09	0.966
3	124678230	125875831	442	-4.28E-06	1.16E-09	0.900
3	125876862	126213162	202	3.52E-05	4.95E-10	0.114
3	126213467	127471230	536	-1.12E-05	1.33E-09	0.757
3	127471641	130243259	811	-2.14E-04	5.61E-09	0.004
3	130244120	131373405	333	-4.19E-05	6.70E-10	0.106
3	131373740	132115911	443	7.67E-06	1.48E-09	0.842
3	132117285	133128449	407	2.99E-06	9.22E-10	0.922
3	133129272	134383242	550	-2.26E-05	1.52E-09	0.562
3	134385789	137369181	1106	5.50E-05	2.46E-09	0.267
3	137370076	138662229	384	3.78E-05	1.42E-09	0.315
3	138665796	140275652	774	4.96E-05	2.00E-09	0.267
3	140276484	142863564	912	-4.34E-06	2.09E-09	0.924
3	142864473	143494546	387	-1.08E-05	1.32E-09	0.767
3	143495679	145663879	729	1.98E-05	1.65E-09	0.627
3	145664135	146722271	402	1.80E-05	9.85E-10	0.566
3	146722383	148316359	475	-2.40E-05	1.25E-09	0.497
3	148316766	149988630	750	3.73E-05	2.05E-09	0.410
3	149994553	151178708	481	-1.07E-05	1.27E-09	0.765
3	151178836	151904190	289	1.14E-05	8.84E-10	0.701
3	151905386	153054901	368	7.08E-06	1.03E-09	0.825
3	153056839	154181836	468	1.85E-05	1.34E-09	0.613
3	154183562	156005200	508	5.78E-06	1.30E-09	0.872

3	156005905	157311596	529	2.40E-05	1.27E-09	0.499
3	157312028	159272972	716	-1.46E-06	1.57E-09	0.971
3	159274785	161523859	700	1.66E-05	1.51E-09	0.669
3	161524489	162065286	249	7.80E-07	7.39E-10	0.977
3	162067622	163250087	376	9.82E-06	7.75E-10	0.724
3	163251056	164816939	494	-3.50E-07	9.56E-10	0.991
3	164820183	167116849	615	1.77E-05	1.41E-09	0.638
3	167117143	167912878	293	-3.49E-05	6.47E-10	0.170
3	167913564	170158128	714	4.30E-06	4.59E-09	0.949
3	170159134	171311936	486	1.18E-05	1.54E-09	0.764
3	171312880	172965694	658	2.03E-05	1.94E-09	0.644
3	172966405	174274308	526	-5.69E-05	1.34E-09	0.121
3	174275336	175049294	365	1.05E-05	1.03E-09	0.745
3	175050262	176268375	462	-1.03E-05	1.21E-09	0.768
3	176269091	177530606	501	4.69E-06	1.42E-09	0.901
3	177531847	179359242	627	5.68E-05	1.58E-09	0.153
3	179366819	181426554	525	-2.09E-06	1.27E-09	0.953
3	181431571	182975975	457	5.72E-05	1.34E-09	0.118
3	182976382	183762614	363	-3.65E-05	7.91E-10	0.194
3	183762644	185262137	590	1.89E-05	1.77E-09	0.653
3	185263467	186780913	684	5.67E-06	2.24E-09	0.905
3	186782999	187220633	227	-7.39E-06	6.61E-10	0.774
3	187220777	188201380	508	-3.15E-05	1.62E-09	0.434
3	188202550	189531496	585	-8.12E-05	1.95E-09	0.066
3	189531911	190303805	414	-4.91E-05	1.22E-09	0.159
3	190304172	190992002	286	-7.36E-06	7.36E-10	0.786
3	190993386	191840443	362	1.60E-05	9.22E-10	0.598
3	191840983	192753870	381	-2.69E-05	1.01E-09	0.397
3	192754177	193885973	516	2.54E-05	1.48E-09	0.508
3	193886289	194464791	287	6.00E-06	9.18E-10	0.843
3	194465233	194756339	146	6.22E-06	5.79E-10	0.796
3	194756597	195854787	325	-7.23E-06	1.41E-09	0.848
3	195855401	196414159	209	-1.60E-05	6.62E-10	0.534
3	196415355	197962382	529	4.02E-07	1.27E-09	0.991
4	10642	685993	149	1.30E-07	2.40E-10	0.993
4	686563	1422863	301	-4.33E-05	7.22E-10	0.107
4	1478711	3612028	639	4.08E-05	1.27E-09	0.252

4	3613164	4263579	147	1.96E-05	5.90E-10	0.419
4	4264705	5044744	440	4.76E-06	1.06E-09	0.884
4	5047695	5971152	587	1.36E-05	1.59E-09	0.733
4	5971634	7135912	602	-8.45E-05	1.91E-09	0.053
4	7136158	8054472	690	-5.53E-05	2.16E-09	0.234
4	8055323	9408136	371	-4.99E-05	1.03E-09	0.119
4	9408158	10696229	611	2.44E-06	9.27E-10	0.936
4	10696913	12198579	733	9.85E-06	1.45E-09	0.796
4	12201002	13002341	347	-9.07E-06	5.77E-10	0.706
4	13002905	14394452	629	-3.40E-05	1.44E-09	0.369
4	14394902	15849986	606	-7.77E-06	1.19E-09	0.822
4	15850685	16520118	382	2.31E-05	8.52E-10	0.428
4	16521003	18752293	861	-6.44E-07	1.64E-09	0.987
4	18752722	20084049	441	3.91E-05	8.65E-10	0.184
4	20084563	21292213	550	3.61E-05	1.13E-09	0.284
4	21292651	22393873	439	1.24E-05	8.26E-10	0.665
4	22395570	23563570	369	2.38E-05	7.72E-10	0.392
4	23563931	24992827	684	-1.41E-05	1.56E-09	0.721
4	24993167	26468997	679	3.76E-05	2.42E-09	0.445
4	26473220	27903383	509	6.74E-06	1.12E-09	0.840
4	27904556	28871862	388	-2.09E-06	7.04E-10	0.937
4	28872833	30453156	508	1.22E-05	1.00E-09	0.701
4	30453619	31739254	482	2.93E-06	9.26E-10	0.923
4	31740009	32592949	294	-1.91E-05	5.45E-10	0.414
4	32593453	33640100	205	1.21E-05	3.47E-10	0.515
4	33640864	35150284	380	-8.70E-06	4.66E-10	0.687
4	35151237	36301997	434	-1.18E-05	8.02E-10	0.676
4	36304377	37534798	577	1.86E-05	1.27E-09	0.602
4	37536119	37886455	212	-2.80E-05	5.82E-10	0.245
4	37887735	38974458	602	-3.67E-05	1.55E-09	0.351
4	38976533	40199852	440	-3.14E-05	9.55E-10	0.309
4	40200304	40869208	304	-2.56E-05	9.72E-10	0.412
4	40870402	42662283	725	-6.03E-05	1.49E-09	0.119
4	42662718	43962569	451	1.05E-05	7.87E-10	0.707
4	43963518	45187835	414	-2.33E-05	6.75E-10	0.370
4	45188855	47543891	621	8.24E-07	1.00E-09	0.979
4	47544012	48226267	277	1.21E-05	4.33E-10	0.561

4	48227642	53412129	409	2.28E-05	4.47E-10	0.281
4	53413674	55202613	631	3.57E-06	1.07E-09	0.913
4	55202697	57418400	884	4.45E-05	1.79E-09	0.293
4	57420107	58696072	630	-2.38E-05	1.44E-09	0.530
4	58697912	59358251	234	-2.92E-06	4.57E-10	0.892
4	59358729	60145179	251	-2.95E-06	4.82E-10	0.893
4	60145181	61371992	375	7.89E-06	6.70E-10	0.761
4	61372415	61872517	167	-2.49E-06	3.14E-10	0.888
4	61872656	64117718	644	-4.59E-06	1.29E-09	0.898
4	64118963	65300308	243	-1.16E-05	3.59E-10	0.539
4	65562213	66598615	369	1.24E-05	6.75E-10	0.633
4	66599449	68058793	478	-1.61E-05	8.78E-10	0.588
4	68059850	68455531	160	1.40E-05	2.78E-10	0.402
4	68456074	69954151	270	1.16E-05	6.33E-10	0.645
4	69954297	70517143	270	4.02E-06	3.73E-10	0.835
4	70517792	71516586	418	1.44E-05	6.49E-10	0.572
4	72416258	75196731	712	-1.50E-06	1.86E-09	0.972
4	75555658	76283179	293	1.69E-05	6.45E-10	0.505
4	76284248	77128724	364	3.48E-06	7.46E-10	0.899
4	77129568	78333381	607	8.94E-06	1.13E-09	0.790
4	78333798	79469226	505	2.48E-05	8.59E-10	0.398
4	79470550	80994124	436	6.73E-06	8.47E-10	0.817
4	80995881	81978151	261	1.45E-06	4.49E-10	0.945
4	81979993	83012967	403	2.34E-05	6.80E-10	0.369
4	83017789	83994716	385	2.13E-05	6.54E-10	0.404
4	83998014	85080933	430	4.09E-05	1.13E-09	0.224
4	85081980	87980070	902	8.19E-06	1.41E-09	0.827
4	87980748	89236855	568	5.03E-06	1.21E-09	0.885
4	89238369	90126190	323	-5.06E-05	7.13E-10	0.058
4	90126331	91453993	447	-2.99E-05	8.73E-10	0.311
4	91456722	94229509	751	1.30E-05	1.45E-09	0.732
4	94230511	95596188	496	-2.16E-05	1.08E-09	0.511
4	95596744	96181408	236	3.96E-06	4.86E-10	0.858
4	96182669	96464931	212	-6.00E-06	5.29E-10	0.794
4	96466095	97911191	514	2.25E-05	9.07E-10	0.455
4	97911459	99422081	372	4.36E-06	4.02E-10	0.828
4	99423280	100322786	399	7.04E-06	6.63E-10	0.785

4	100323029	101897750	598	-6.63E-06	9.94E-10	0.833
4	101898276	103387944	551	-3.13E-05	1.40E-09	0.403
4	103388441	104802530	435	-1.64E-05	5.63E-10	0.491
4	104803329	106479425	508	1.15E-04	4.30E-09	0.079
4	106482185	108468632	628	-2.70E-05	1.09E-09	0.414
4	108469756	109979131	575	-1.98E-06	9.84E-10	0.950
4	109980374	112204254	780	4.94E-05	1.61E-09	0.219
4	112204259	114902479	1024	-8.66E-06	1.88E-09	0.842
4	117275542	118219755	308	1.34E-05	5.82E-10	0.577
4	114904730	117275534	678	1.75E-05	1.02E-09	0.583
4	118220292	119945314	632	-1.44E-05	1.13E-09	0.667
4	119946706	121391843	519	3.68E-06	8.13E-10	0.897
4	121393020	121973864	244	1.36E-05	5.06E-10	0.546
4	121976226	123561869	551	2.14E-05	1.01E-09	0.501
4	123563078	125863762	773	-3.57E-05	1.48E-09	0.354
4	125863974	127236805	494	-4.55E-06	1.35E-09	0.902
4	127237363	129514427	625	-2.64E-06	9.21E-10	0.931
4	129514448	130364383	286	-4.99E-06	5.54E-10	0.832
4	130364741	130949530	194	1.09E-06	3.29E-10	0.952
4	130950008	131948861	379	-1.05E-05	7.66E-10	0.705
4	131951466	133741365	442	-3.60E-06	7.36E-10	0.894
4	133741753	135744132	532	-3.34E-05	9.13E-10	0.269
4	135749591	137247266	391	8.02E-06	8.18E-10	0.779
4	137247703	137987679	298	1.45E-05	7.07E-10	0.585
4	137988838	139114529	404	2.43E-05	7.94E-10	0.388
4	139115093	139552965	239	6.26E-06	5.49E-10	0.789
4	139553359	140546206	342	-1.68E-05	6.51E-10	0.509
4	140548713	141751418	489	-1.65E-05	1.35E-09	0.653
4	141752960	142807957	391	-4.58E-05	8.62E-10	0.119
4	142808503	145021868	627	1.42E-06	8.40E-10	0.961
4	145024452	148047972	852	2.63E-05	1.50E-09	0.498
4	148053679	150632409	780	1.33E-05	1.35E-09	0.719
4	150634191	153226998	838	-3.61E-06	1.25E-09	0.919
4	153233476	154947363	615	-2.97E-05	1.17E-09	0.386
4	154949091	156604003	632	-1.99E-05	1.38E-09	0.592
4	156604173	158112985	581	-5.90E-06	1.24E-09	0.867
4	158114105	160356254	644	-4.95E-06	1.08E-09	0.880

4	160357877	160822830	147	5.45E-06	2.34E-10	0.721
4	161056173	161796696	321	8.05E-06	6.48E-10	0.752
4	162151565	162629423	187	-5.18E-06	5.39E-10	0.823
4	162629512	163181531	216	7.94E-06	4.10E-10	0.695
4	163183341	163726178	186	-1.16E-05	4.25E-10	0.574
4	163728264	164910818	398	-5.90E-06	7.89E-10	0.834
4	164913270	165817608	368	-5.95E-06	7.67E-10	0.830
4	165820093	166609852	327	1.70E-05	7.58E-10	0.536
4	166611193	167643023	297	-9.86E-07	6.78E-10	0.970
4	167643601	169568082	733	-2.71E-05	1.49E-09	0.484
4	169569516	170676328	421	3.15E-05	7.70E-10	0.257
4	170678993	172735897	573	-3.64E-05	1.01E-09	0.252
4	172737462	173760563	310	-2.66E-05	5.36E-10	0.250
4	173761916	174832248	301	6.59E-05	8.57E-10	0.024
4	174833988	176193705	541	-1.83E-05	1.42E-09	0.628
4	176199091	177290105	362	-3.47E-05	8.65E-10	0.238
4	177290945	177972278	282	4.37E-06	5.74E-10	0.855
4	177974062	178758792	336	-1.41E-05	9.52E-10	0.647
4	178758930	180119509	494	3.90E-05	1.10E-09	0.240
4	180120693	181732608	834	3.03E-05	2.26E-09	0.523
4	181732746	182420390	387	2.23E-05	1.27E-09	0.532
4	184283411	185477454	594	-7.69E-05	1.94E-09	0.081
4	182420863	184283167	1001	4.68E-06	3.02E-09	0.932
4	185477964	185932497	229	-7.92E-06	4.72E-10	0.716
4	185934003	187113041	594	-3.18E-05	1.65E-09	0.434
4	187114073	188687052	716	-1.20E-05	1.85E-09	0.780
4	188687155	189524815	363	3.34E-05	1.28E-09	0.351
4	189525752	190088167	270	1.71E-05	7.11E-10	0.521
4	190088768	191043878	201	1.06E-05	5.29E-10	0.646
5	10056	1267356	414	3.39E-05	9.09E-10	0.261
5	1270983	1762678	242	3.50E-06	1.38E-09	0.925
5	1763566	2369337	388	3.47E-05	2.39E-09	0.478
5	2369710	2979000	434	4.26E-05	1.30E-09	0.237
5	2979710	3600334	481	-9.17E-06	1.20E-09	0.792
5	3601924	4222111	306	2.53E-05	7.97E-10	0.371
5	4223577	5137474	433	-1.71E-05	1.01E-09	0.591
5	5137788	5718773	375	6.87E-06	9.38E-10	0.822

5	5719162	6858229	683	3.68E-06	1.59E-09	0.926
5	6859297	7852978	495	-3.61E-06	1.09E-09	0.913
5	7853304	9084076	627	3.43E-05	1.22E-09	0.327
5	9084701	10533767	857	1.18E-05	1.94E-09	0.788
5	10666804	11170506	324	-2.92E-05	6.52E-10	0.253
5	11170703	13603734	759	2.42E-05	1.32E-09	0.506
5	13603883	14987807	739	-3.74E-05	1.40E-09	0.318
5	14988284	16034608	301	-1.69E-05	4.01E-10	0.399
5	16035369	17161451	494	-4.00E-05	1.19E-09	0.245
5	17162704	18048683	259	9.14E-06	9.30E-10	0.764
5	18050107	18791978	252	-8.73E-06	6.80E-10	0.738
5	18792568	19486153	258	5.24E-06	4.89E-10	0.812
5	19487359	20414139	285	1.53E-06	4.03E-10	0.939
5	20415799	21573200	239	6.90E-06	4.60E-10	0.748
5	21574347	23150924	573	-4.39E-07	8.34E-10	0.988
5	23153562	24794957	518	1.92E-06	9.31E-10	0.950
5	24796795	26183141	454	2.20E-05	9.60E-10	0.478
5	26185302	26849079	203	1.45E-05	4.65E-10	0.501
5	26849808	28303480	392	2.74E-05	7.58E-10	0.321
5	28310093	30129786	583	2.60E-05	1.03E-09	0.419
5	30130713	31748529	717	-2.08E-06	1.60E-09	0.959
5	31748611	33944217	987	9.19E-06	2.40E-09	0.851
5	33945758	35798682	686	2.74E-05	1.41E-09	0.466
5	35799275	37888797	710	-2.80E-05	1.34E-09	0.443
5	37889231	39438373	674	-1.32E-05	1.42E-09	0.727
5	39438575	40954879	626	-8.76E-06	9.44E-10	0.776
5	40955561	42620153	556	-9.93E-06	7.75E-10	0.721
5	42623245	43859513	307	2.71E-05	4.80E-10	0.215
5	43862567	45286358	281	-5.10E-05	4.95E-10	0.022
5	45286977	50161698	290	-4.52E-06	2.78E-10	0.786
5	50162186	52133670	654	-7.63E-05	1.15E-09	0.024
5	52134410	52661197	301	-2.03E-05	5.42E-10	0.384
5	52662483	53513531	324	1.07E-05	6.78E-10	0.681
5	53515888	55412347	790	-6.74E-05	1.31E-09	0.063
5	55413961	55932471	299	-2.68E-05	8.59E-10	0.361
5	55933701	57337744	583	-4.69E-05	1.02E-09	0.142
5	57338357	58834736	805	4.87E-06	1.56E-09	0.902

5	58834852	60880207	583	9.64E-06	8.47E-10	0.740
5	60883078	62531300	658	4.95E-05	1.05E-09	0.127
5	62531952	63837838	373	1.58E-05	6.06E-10	0.520
5	63840208	65083148	459	1.56E-05	7.00E-10	0.554
5	65083209	65909616	334	-1.50E-05	7.31E-10	0.578
5	65910316	67096208	520	-1.25E-05	9.94E-10	0.691
5	67096780	70682458	690	3.44E-05	1.64E-09	0.397
5	70683839	72420748	629	-8.35E-07	9.15E-10	0.978
5	72420784	73507955	557	-3.00E-05	1.20E-09	0.386
5	73508509	75240469	728	1.44E-05	1.30E-09	0.689
5	75242395	75740199	233	2.37E-06	5.05E-10	0.916
5	75743532	77203135	737	6.35E-05	1.96E-09	0.151
5	77203718	79068089	685	4.41E-06	1.28E-09	0.902
5	79068300	80480761	705	6.45E-08	1.57E-09	0.999
5	80481996	81712715	434	-4.39E-05	8.18E-10	0.125
5	81714078	82658571	362	1.14E-05	7.75E-10	0.681
5	82660901	84397685	586	-2.90E-05	1.42E-09	0.441
5	84399219	85315500	310	6.04E-06	4.66E-10	0.780
5	85316429	86490293	328	1.77E-05	6.40E-10	0.484
5	86492986	88065637	305	1.43E-05	4.26E-10	0.487
5	88069593	90500398	712	9.40E-06	1.30E-09	0.794
5	90504842	93602613	721	1.87E-05	1.14E-09	0.579
5	93607443	95822590	793	4.78E-05	1.51E-09	0.218
5	95822732	96867936	466	-5.46E-05	8.31E-10	0.058
5	96870861	97435808	143	-1.30E-05	2.62E-10	0.421
5	97436504	99258325	558	-2.35E-05	9.10E-10	0.437
5	99259751	101101103	483	-3.29E-06	6.98E-10	0.901
5	101101325	102681586	443	2.07E-06	5.60E-10	0.930
5	102686157	104542488	700	-8.10E-06	1.46E-09	0.832
5	104543131	105885913	312	9.23E-06	5.52E-10	0.694
5	105887333	106800282	343	-3.78E-05	7.16E-10	0.158
5	106800765	108633176	758	3.89E-05	1.62E-09	0.333
5	108633347	109231597	246	-3.13E-06	3.55E-10	0.868
5	109232816	110819753	510	-1.39E-05	8.93E-10	0.642
5	110821786	111962676	471	1.21E-05	1.03E-09	0.707
5	111963025	112396750	156	2.05E-06	2.32E-10	0.893
5	112397011	113603768	561	1.06E-05	9.85E-10	0.736

5	113604579	114448326	333	-6.50E-06	6.08E-10	0.792
5	114448597	115828291	681	-5.09E-05	1.37E-09	0.170
5	115829425	117003674	543	-6.61E-05	1.14E-09	0.051
5	117004531	117712427	268	-8.31E-06	5.31E-10	0.718
5	117714517	119668423	688	-2.48E-05	1.34E-09	0.497
5	119669457	120962383	495	1.30E-06	1.01E-09	0.967
5	120963248	121481183	148	6.70E-06	2.71E-10	0.684
5	121485609	122603725	451	-1.94E-05	8.16E-10	0.498
5	122604501	123236124	257	1.80E-05	4.61E-10	0.403
5	123238611	123797013	229	-7.72E-06	4.90E-10	0.727
5	123798436	124746813	465	-2.35E-05	1.16E-09	0.491
5	124748624	126938920	874	7.58E-06	1.72E-09	0.855
5	126940058	128727903	666	-1.39E-05	1.11E-09	0.676
5	128730275	130376511	412	-5.84E-06	5.02E-10	0.794
5	130376936	131873073	450	4.98E-06	5.08E-10	0.825
5	131874022	132603137	224	1.17E-05	3.72E-10	0.544
5	132609517	134321546	718	-6.86E-05	1.58E-09	0.084
5	134329463	135339464	501	-1.58E-05	9.64E-10	0.611
5	135340951	137948424	905	4.83E-05	1.34E-09	0.187
5	137950183	140644484	766	-7.21E-06	9.43E-10	0.814
5	140644957	141640050	475	-1.31E-05	1.08E-09	0.691
5	141640419	142230251	276	-2.02E-05	7.10E-10	0.449
5	144584265	145803004	431	2.63E-06	6.04E-10	0.915
5	142230949	144583138	963	1.66E-05	1.61E-09	0.679
5	145803849	147651230	762	-7.78E-05	1.46E-09	0.042
5	150395351	150917796	368	-3.81E-05	7.31E-10	0.159
5	147665223	150393107	1253	9.56E-06	2.29E-09	0.842
5	150918158	152360494	594	8.48E-05	1.38E-09	0.022
5	152361546	152864614	177	5.04E-06	2.90E-10	0.767
5	152867348	153494463	316	-5.40E-06	4.40E-10	0.797
5	153495193	154448863	413	-1.90E-06	8.56E-10	0.948
5	154449856	155290211	358	-3.24E-06	6.42E-10	0.898
5	155290613	156628287	483	-1.99E-06	9.11E-10	0.947
5	156629754	157197141	306	-5.09E-05	5.41E-10	0.029
5	157199547	158491655	551	-6.14E-06	9.58E-10	0.843
5	158492279	159135376	279	5.50E-05	6.13E-10	0.026
5	159136348	160908680	774	-1.41E-06	1.62E-09	0.972

5	160910101	161688620	224	-4.27E-07	3.99E-10	0.983
5	161688913	162741298	358	8.44E-06	6.75E-10	0.745
5	162742614	163769136	431	5.89E-06	8.85E-10	0.843
5	163769738	164944949	399	-1.02E-05	7.52E-10	0.710
5	164947504	165638940	255	-2.33E-06	7.66E-10	0.933
5	165642395	166432731	347	6.92E-06	8.92E-10	0.817
5	166433367	167760730	549	-6.57E-06	1.27E-09	0.854
5	167761922	169124478	717	-2.89E-05	1.71E-09	0.486
5	169125063	170889081	954	6.57E-05	1.99E-09	0.141
5	170891834	171772889	429	-1.25E-05	1.02E-09	0.696
5	171775715	172376903	328	2.29E-05	8.92E-10	0.444
5	172377031	173611384	635	-5.50E-05	1.51E-09	0.157
5	173613085	174468408	481	7.37E-05	1.88E-09	0.089
5	174468624	176128648	690	1.00E-05	1.75E-09	0.811
5	176129392	177991305	618	1.52E-05	1.45E-09	0.691
5	177992368	178410668	199	7.14E-05	5.26E-10	0.002
5	178410839	179245749	391	-7.28E-05	9.70E-10	0.019
5	179245856	180007693	316	4.71E-05	9.08E-10	0.118
5	180008456	180790950	242	1.37E-06	7.10E-10	0.959
6	63979	824529	335	-2.68E-05	1.09E-09	0.417
6	826312	1582647	523	-1.11E-06	1.73E-09	0.979
6	1582926	2890836	718	3.02E-05	1.89E-09	0.488
6	2891016	4315210	772	-7.10E-05	2.15E-09	0.125
6	4317398	5056146	426	3.69E-05	1.05E-09	0.254
6	5057399	6246639	735	-1.40E-06	1.95E-09	0.975
6	6246984	7328580	685	2.51E-05	2.27E-09	0.598
6	7329417	8791321	741	-2.11E-05	2.40E-09	0.666
6	8791421	9760323	374	2.29E-06	9.59E-10	0.941
6	9764413	11234164	726	-3.55E-05	2.16E-09	0.445
6	11235682	12183952	544	1.62E-05	1.29E-09	0.652
6	12186102	13388480	617	2.08E-05	1.39E-09	0.578
6	13389521	14618107	554	4.66E-05	1.35E-09	0.205
6	14619148	15998368	596	-4.89E-05	2.67E-09	0.344
6	15998406	17067160	545	-4.56E-05	1.73E-09	0.273
6	17069966	19203108	880	-1.16E-05	1.95E-09	0.792
6	19203782	20228741	524	-1.28E-05	1.52E-09	0.742
6	20231516	22049758	824	1.40E-05	2.63E-09	0.784

6	22052425	23495209	748	-4.30E-06	3.00E-09	0.937
6	23495260	23935020	233	6.28E-06	5.45E-10	0.788
6	23935352	24799440	438	4.01E-06	1.28E-09	0.911
6	24803038	25483400	411	1.79E-05	1.02E-09	0.576
6	25484295	26774948	730	8.13E-06	2.49E-09	0.870
6	26778827	28669698	609	-1.93E-05	1.39E-09	0.604
6	32900018	33863002	401	-5.70E-05	1.81E-09	0.181
6	33863662	35451187	514	7.71E-05	1.01E-09	0.015
6	35452346	36608267	395	1.01E-05	8.28E-10	0.725
6	38687185	39021712	205	-1.54E-07	4.54E-10	0.994
6	36611164	38686222	997	2.67E-05	2.48E-09	0.591
6	39022698	40344526	675	1.92E-05	1.59E-09	0.630
6	40345542	42105449	887	-4.60E-05	2.62E-09	0.369
6	42105555	43957473	633	-2.90E-05	1.68E-09	0.479
6	43959116	45787468	702	1.86E-05	1.75E-09	0.658
6	45788111	46556325	327	-3.52E-06	6.77E-10	0.892
6	46560003	47885216	681	-1.21E-05	1.37E-09	0.744
6	47887063	49494241	468	-1.01E-06	8.19E-10	0.972
6	49494933	50406476	309	-1.43E-05	6.74E-10	0.581
6	50406575	51703012	475	2.87E-06	9.83E-10	0.927
6	51704668	53402679	834	1.10E-05	1.98E-09	0.804
6	53405127	54291407	454	-2.04E-06	9.10E-10	0.946
6	54293261	56103747	744	-1.01E-05	1.48E-09	0.793
6	56104522	57082026	352	-7.02E-06	6.48E-10	0.783
6	57085542	58084365	151	-2.38E-06	3.11E-10	0.892
6	58139505	62691733	306	2.91E-06	5.33E-10	0.900
6	62692409	63712779	264	3.16E-06	4.18E-10	0.877
6	63713620	65760585	508	-1.08E-05	8.16E-10	0.706
6	65762553	67028107	493	-1.58E-05	9.84E-10	0.614
6	67029501	67556729	242	-4.61E-07	5.61E-10	0.984
6	67557245	68546601	376	3.56E-05	8.21E-10	0.214
6	68546995	69295414	301	-1.51E-06	8.12E-10	0.958
6	69295526	70359936	399	-1.79E-05	7.80E-10	0.522
6	70360869	72448598	882	2.85E-05	1.85E-09	0.508
6	72449421	73170712	296	5.75E-06	6.67E-10	0.824
6	73171594	75064563	683	5.84E-05	1.46E-09	0.127
6	75065279	75843360	172	-3.44E-06	3.95E-10	0.863

6	75843845	77257094	415	-2.06E-05	7.15E-10	0.442
6	77257275	78957547	680	1.27E-05	1.30E-09	0.724
6	78958134	79861169	296	-6.15E-06	5.46E-10	0.793
6	79862483	80556359	340	1.43E-05	8.31E-10	0.619
6	80557170	82387384	660	5.59E-05	1.19E-09	0.105
6	82387920	83904869	519	-9.15E-06	1.11E-09	0.783
6	83908232	86040334	810	-8.15E-07	1.68E-09	0.984
6	86040497	87138115	340	-1.24E-05	6.25E-10	0.619
6	87140364	88532072	486	-7.55E-05	8.58E-10	0.010
6	88533324	89668547	467	3.90E-05	1.27E-09	0.274
6	89673861	91400644	815	5.64E-05	1.98E-09	0.205
6	91401234	93426186	759	-9.20E-06	1.82E-09	0.829
6	93429797	94919277	573	4.60E-05	1.30E-09	0.203
6	94920611	97093037	660	-2.67E-05	1.41E-09	0.478
6	97093295	98893182	514	-8.79E-07	1.04E-09	0.978
6	98893830	100286683	585	2.11E-05	1.32E-09	0.562
6	100287245	101862923	465	6.93E-06	9.28E-10	0.820
6	101863563	102887537	348	6.88E-06	8.45E-10	0.813
6	102888281	104211089	445	-2.28E-05	8.40E-10	0.431
6	104211475	105752435	549	-1.97E-05	1.15E-09	0.560
6	105754306	106921804	537	-1.59E-05	1.58E-09	0.690
6	106922281	108322638	656	-1.03E-05	1.74E-09	0.805
6	108325624	110654066	761	-3.62E-05	1.40E-09	0.332
6	110654605	111390344	274	5.45E-06	6.64E-10	0.833
6	114220361	116130955	594	-7.86E-06	1.25E-09	0.824
6	111391537	114218733	1067	2.06E-05	2.27E-09	0.666
6	116130983	117566682	496	-7.52E-05	1.25E-09	0.034
6	117567468	119071713	561	2.41E-06	1.18E-09	0.944
6	119075856	120628075	540	1.48E-05	1.20E-09	0.668
6	120628214	121673627	339	1.49E-05	6.25E-10	0.552
6	121674337	123104703	522	-1.64E-05	1.04E-09	0.610
6	123105676	123889346	320	3.38E-05	8.16E-10	0.236
6	123890282	125333797	658	-2.05E-05	1.72E-09	0.621
6	125336799	128759251	900	-1.54E-05	1.84E-09	0.719
6	128759755	130538497	787	-4.18E-06	1.94E-09	0.924
6	130540383	131972731	564	3.94E-05	1.26E-09	0.268
6	131973932	133867802	873	2.94E-05	2.20E-09	0.531

6	133869809	136186007	802	-1.14E-05	1.95E-09	0.796
6	136186368	137811295	508	7.97E-05	1.13E-09	0.018
6	137813095	139532424	748	2.50E-05	2.03E-09	0.579
6	139533572	142036032	709	3.74E-06	1.54E-09	0.924
6	142036912	143719278	602	-1.85E-06	1.32E-09	0.959
6	143720467	146663272	1047	1.70E-05	2.20E-09	0.718
6	146665424	148179283	592	-3.38E-05	1.29E-09	0.347
6	148180191	148917941	479	-3.19E-05	1.59E-09	0.423
6	148919158	150298842	639	5.88E-06	1.28E-09	0.870
6	150299299	151097980	538	7.85E-06	1.67E-09	0.847
6	151100484	151929942	491	1.93E-05	1.50E-09	0.619
6	151930249	153550147	880	-4.25E-05	2.80E-09	0.422
6	153551177	155228511	745	-1.08E-05	2.00E-09	0.810
6	155229566	156756457	768	6.89E-06	2.13E-09	0.881
6	156761338	157398853	256	4.47E-05	6.35E-10	0.076
6	157402041	158192951	273	-1.57E-05	7.96E-10	0.579
6	158193244	159318774	547	-2.64E-05	1.10E-09	0.426
6	159319812	159792458	251	-1.88E-05	4.46E-10	0.374
6	159793334	161123451	690	2.12E-05	7.00E-09	0.800
6	161123771	162298351	679	-1.70E-05	4.19E-09	0.793
6	162300151	163277414	510	-5.00E-07	1.20E-09	0.988
6	163277858	164003180	378	5.03E-05	1.00E-09	0.112
6	164006012	165242243	674	-1.90E-05	1.67E-09	0.642
6	165246062	166191910	572	1.17E-05	1.56E-09	0.767
6	166193932	167177733	678	3.08E-05	1.90E-09	0.480
6	167178790	168548525	628	6.44E-06	1.45E-09	0.866
6	168549335	169231503	390	-2.25E-05	1.06E-09	0.490
6	169231937	171049558	874	8.00E-06	1.98E-09	0.857
7	20608	1490775	419	3.74E-05	1.24E-09	0.289
7	1493090	2479029	317	1.20E-06	7.48E-10	0.965
7	2480729	2930941	207	1.61E-05	5.01E-10	0.472
7	2931676	3736942	499	-3.98E-06	1.22E-09	0.910
7	3738087	4783224	512	1.38E-07	1.26E-09	0.997
7	4783723	5412932	213	7.57E-06	7.06E-10	0.776
7	5414602	6912527	401	2.44E-05	1.04E-09	0.450
7	7168493	7679885	293	-3.11E-05	7.00E-10	0.239
7	7680697	8475147	543	5.92E-05	1.61E-09	0.140

7	8476787	9223721	526	-1.79E-05	1.36E-09	0.627
7	9223762	9893744	341	-2.06E-05	9.41E-10	0.502
7	9894421	11298815	623	-6.94E-06	1.38E-09	0.852
7	11299552	12687871	880	-4.28E-05	2.19E-09	0.360
7	12689322	13674908	508	-3.39E-07	1.22E-09	0.992
7	13675094	14941781	684	3.39E-05	1.75E-09	0.417
7	14942134	16480989	759	1.33E-05	1.70E-09	0.747
7	16481863	17277084	484	-5.64E-06	1.47E-09	0.883
7	17279294	18861958	710	5.79E-06	1.69E-09	0.888
7	18863387	19975992	510	2.30E-05	1.09E-09	0.486
7	19978609	21712233	880	-5.74E-05	3.36E-09	0.322
7	21712615	22811384	708	4.77E-05	1.96E-09	0.281
7	22811921	24389035	702	-2.75E-05	1.36E-09	0.456
7	24389147	24980408	323	-2.09E-06	8.15E-10	0.942
7	24982494	26300992	674	-3.65E-05	2.77E-09	0.487
7	26302668	28038375	768	7.19E-05	8.90E-09	0.446
7	28039590	28710596	382	-2.65E-05	1.46E-09	0.487
7	28710857	30171413	827	3.65E-05	2.02E-09	0.417
7	30172195	31608495	659	4.35E-06	1.60E-09	0.913
7	31609317	33150311	750	-9.72E-06	1.60E-09	0.808
7	33151181	34939884	766	1.95E-05	1.67E-09	0.633
7	34940039	35614036	301	-7.69E-06	6.28E-10	0.759
7	35615611	37353302	858	5.72E-05	2.03E-09	0.204
7	37354091	38159855	466	-7.22E-06	1.08E-09	0.826
7	38160558	39534540	677	8.46E-06	1.62E-09	0.833
7	39535570	40925323	373	-2.91E-05	7.23E-10	0.279
7	40926906	42494824	654	9.05E-06	1.56E-09	0.819
7	46667873	47279128	336	-2.30E-06	7.29E-10	0.932
7	42495555	44971837	906	1.87E-05	1.67E-09	0.647
7	44972152	46665658	694	-3.61E-05	1.47E-09	0.346
7	47281095	49130572	861	6.05E-05	2.15E-09	0.192
7	49131824	49821750	300	3.31E-05	6.07E-10	0.179
7	49822437	51674880	774	-7.22E-06	1.51E-09	0.853
7	51675218	52274523	293	-8.46E-06	7.75E-10	0.761
7	52275656	53175065	478	2.91E-06	1.05E-09	0.928
7	53348777	54707110	519	3.72E-06	1.00E-09	0.906
7	54707337	55641707	451	1.30E-06	1.04E-09	0.968

7	55641798	57382254	387	-2.89E-06	7.77E-10	0.917
7	57382271	62556935	175	-1.19E-05	2.70E-10	0.467
7	62557202	64193303	281	1.92E-06	4.97E-10	0.931
7	64699988	65570171	153	-2.50E-06	2.14E-10	0.865
7	65570310	66453460	219	4.88E-06	2.15E-10	0.739
7	66454071	67369810	339	-1.00E-05	7.56E-10	0.716
7	67370861	68125403	317	1.33E-05	8.56E-10	0.649
7	68126378	68561421	188	1.24E-05	4.20E-10	0.544
7	68562932	69806895	289	-6.77E-05	4.55E-10	0.002
7	69808153	71204524	594	3.41E-06	1.69E-09	0.934
7	71205507	71873571	249	9.61E-07	4.84E-10	0.965
7	71874997	73996533	397	1.54E-06	7.71E-10	0.956
7	73997700	76550299	364	-6.67E-05	8.41E-10	0.021
7	76551983	77101532	181	3.50E-06	4.13E-10	0.863
7	77102731	77953248	411	3.97E-06	8.33E-10	0.891
7	77954163	78839317	553	-1.79E-05	1.37E-09	0.628
7	78843289	80546745	614	1.44E-05	1.22E-09	0.681
7	80547047	82758529	882	-1.09E-05	2.21E-09	0.816
7	82758644	83959105	533	2.21E-05	1.14E-09	0.513
7	83959751	85570789	448	-2.97E-05	7.52E-10	0.278
7	85571372	86341446	218	-7.72E-06	3.77E-10	0.691
7	88732470	89270556	217	-1.32E-05	4.48E-10	0.533
7	86345013	88732142	836	1.67E-05	1.45E-09	0.662
7	89271899	91173749	774	-3.95E-05	1.55E-09	0.316
7	91174778	93339775	617	2.45E-07	1.06E-09	0.994
7	93340137	95171731	630	1.39E-05	1.41E-09	0.711
7	95419256	96611864	460	-3.92E-05	9.03E-10	0.192
**7**	**96613246**	**98151455**	**496**	**-4.79E-04**	**8.58E-09**	**0.000**
7	98152191	100555361	666	2.40E-05	1.25E-09	0.498
7	100606141	101730309	387	3.30E-05	1.30E-09	0.360
7	101731567	103193331	319	5.56E-06	6.32E-10	0.825
7	103193651	104157576	550	-3.89E-06	1.22E-09	0.911
7	104158491	105425027	481	-3.67E-05	8.44E-10	0.206
7	105427565	106052506	320	1.57E-07	9.15E-10	0.996
7	106052873	107305328	457	2.51E-05	9.66E-10	0.420
7	107306421	108375939	543	-2.26E-05	1.04E-09	0.484
7	108376173	109584769	351	4.01E-06	6.70E-10	0.877

7	109587777	111447153	593	-2.31E-05	1.12E-09	0.490
7	111450919	113336970	590	-6.47E-06	1.29E-09	0.857
7	113337327	116315205	824	-8.76E-05	1.57E-09	0.027
7	116317043	118347634	570	3.03E-05	9.56E-10	0.328
7	118348359	121094386	573	5.50E-06	9.05E-10	0.855
7	121094460	122368663	413	-1.54E-05	9.06E-10	0.609
7	122369853	124154451	639	4.94E-06	1.09E-09	0.881
7	124155319	125386718	379	2.31E-05	5.90E-10	0.342
7	125387168	126516683	442	-1.02E-05	7.94E-10	0.717
7	126517818	127905983	588	-3.90E-05	9.83E-10	0.214
7	127907054	128554075	209	-3.40E-06	4.74E-10	0.876
7	128555262	130074230	546	-2.39E-05	1.14E-09	0.478
7	130075370	130797123	249	-1.25E-05	7.15E-10	0.641
7	130797776	131856855	498	-1.75E-05	1.30E-09	0.628
7	131857419	134306672	895	8.06E-05	1.65E-09	0.048
7	134307114	135464701	504	1.85E-05	1.07E-09	0.572
7	135466022	137137511	701	1.15E-05	1.54E-09	0.770
7	137138736	138742981	587	-4.44E-05	1.72E-09	0.285
7	138744100	141106552	845	-4.81E-05	2.30E-09	0.316
7	141109797	142655113	482	2.25E-05	8.54E-10	0.440
7	142655717	144274735	430	-1.20E-05	8.40E-10	0.678
7	144276424	146018520	552	-7.03E-06	1.16E-09	0.837
7	146023023	147204086	503	1.38E-05	1.02E-09	0.666
7	147204549	148619685	685	1.04E-05	1.52E-09	0.791
7	148620581	150491084	657	1.77E-05	1.39E-09	0.635
7	150491851	151390799	461	-2.23E-05	1.27E-09	0.532
7	151391044	152409155	288	3.62E-05	1.26E-09	0.308
7	152410248	153080056	288	-8.68E-05	9.13E-10	0.004
7	153081698	153934166	322	6.78E-06	8.63E-10	0.818
7	153934210	154963787	621	-7.72E-06	1.61E-09	0.847
7	154964316	155379409	239	1.53E-06	1.01E-09	0.962
7	155380353	156814410	651	1.68E-06	1.74E-09	0.968
7	156815867	158029340	505	4.79E-05	1.32E-09	0.188
7	158030463	158759939	286	4.77E-06	5.53E-10	0.839
7	158760677	159127587	134	-4.79E-06	3.04E-10	0.783
8	246934	1162157	489	1.51E-05	1.53E-09	0.698
8	1162454	1625424	273	-6.25E-06	8.71E-10	0.832

8	1625732	2042364	303	1.10E-05	1.14E-09	0.745
8	2043187	2680255	317	1.39E-05	1.12E-09	0.678
8	2681051	3358423	619	-6.93E-06	2.00E-09	0.877
8	3358433	3584832	297	-2.30E-06	8.64E-10	0.938
8	3585058	4071438	630	-2.62E-05	1.77E-09	0.533
8	4071516	4274957	255	7.15E-06	7.44E-10	0.793
8	4275286	4479596	220	1.44E-05	8.73E-10	0.626
8	4479836	4754328	302	-2.01E-06	8.30E-10	0.944
8	4755088	5146808	311	-1.52E-05	8.97E-10	0.611
8	5147734	5781592	596	2.00E-05	1.67E-09	0.624
8	5781947	6017670	226	-7.85E-06	5.61E-10	0.740
8	6018292	6350873	272	-3.66E-07	6.69E-10	0.989
8	6352358	7793703	490	-2.86E-05	1.46E-09	0.455
8	7793837	8735213	412	1.34E-05	1.01E-09	0.673
8	8736386	10117293	800	-1.16E-05	2.14E-09	0.803
8	10117411	10535826	286	-1.06E-05	8.91E-10	0.724
8	10537940	11249261	410	-1.92E-05	9.16E-10	0.525
8	11250848	11836622	364	-1.85E-05	1.19E-09	0.591
8	11838355	12975295	284	-3.65E-05	8.19E-10	0.202
8	12975578	13940652	686	3.53E-05	1.82E-09	0.408
8	13942500	14876660	523	-2.71E-05	1.27E-09	0.446
8	14877288	15273777	321	-9.11E-06	7.30E-10	0.736
8	15274608	16087631	534	-7.77E-06	1.44E-09	0.838
8	16087729	17579518	803	-2.46E-05	2.25E-09	0.604
8	17581106	18670645	722	-4.84E-05	1.77E-09	0.250
8	18670908	19490180	559	-2.96E-05	1.56E-09	0.453
8	19491428	21021401	887	5.73E-05	2.56E-09	0.257
8	21022075	21402658	257	-2.57E-06	6.58E-10	0.920
8	21404381	22539316	482	-2.89E-07	1.08E-09	0.993
8	22539641	23548146	588	-8.04E-05	5.60E-09	0.282
8	23548749	24456913	317	-1.26E-06	7.46E-10	0.963
8	24457122	25461315	425	3.66E-05	8.68E-10	0.214
8	25461344	26681450	716	-6.28E-05	2.75E-09	0.231
8	26681799	27404354	420	-1.33E-05	9.37E-10	0.663
8	27405207	27759126	214	-2.54E-06	4.25E-10	0.902
8	27759839	29277590	622	-4.76E-07	1.46E-09	0.990
8	31947880	32448740	282	-1.08E-05	7.12E-10	0.685

8	29277652	31947675	956	6.56E-05	2.41E-09	0.181
8	32449533	33980486	355	1.45E-05	6.74E-10	0.578
8	33981742	35313077	399	1.78E-05	6.56E-10	0.488
8	35314837	37375458	435	-4.72E-06	8.86E-10	0.874
8	37376914	39779989	596	-2.25E-05	1.45E-09	0.554
8	39781444	40415799	340	2.36E-05	8.69E-10	0.423
8	40417433	41718356	592	1.24E-05	1.54E-09	0.751
8	41719750	42501174	186	8.45E-06	3.78E-10	0.664
8	42501717	49028667	390	3.27E-07	5.52E-10	0.989
8	49038732	52156842	816	2.05E-05	1.36E-09	0.578
8	52157415	54115712	718	-1.59E-05	1.49E-09	0.680
8	54116775	55914038	660	-1.28E-06	1.79E-09	0.976
8	55914314	57653028	655	1.52E-05	1.52E-09	0.696
8	57655067	58674153	403	1.63E-05	8.64E-10	0.580
8	58675131	59568410	383	-3.63E-06	9.50E-10	0.906
8	59570176	61809929	767	-6.53E-05	1.70E-09	0.113
8	61810694	63710298	771	3.50E-06	1.70E-09	0.932
8	63711348	65193342	460	-2.44E-06	9.99E-10	0.938
8	65195953	66024947	260	-1.05E-05	5.50E-10	0.653
8	66025862	67192642	387	-2.86E-05	8.03E-10	0.313
8	67193708	69108839	501	-2.86E-05	1.17E-09	0.403
8	69111244	70003448	401	1.96E-05	1.10E-09	0.554
8	70003607	72013005	727	1.34E-05	1.70E-09	0.746
8	72014069	72749564	427	1.17E-05	1.25E-09	0.740
8	72751469	73423214	352	-1.27E-05	9.26E-10	0.677
8	73423786	74996061	687	1.34E-07	1.62E-09	0.997
8	74998868	76294909	508	3.07E-06	1.18E-09	0.929
8	76295292	78015335	544	-1.89E-05	1.14E-09	0.575
8	78015611	78632215	197	1.20E-05	3.90E-10	0.544
8	78632893	79831888	374	9.20E-06	6.83E-10	0.725
8	79832211	80654655	273	-1.06E-05	7.35E-10	0.697
8	80656206	81330540	223	-8.64E-06	5.75E-10	0.719
8	81331077	83726996	809	-1.38E-05	1.89E-09	0.751
8	83727741	85093121	385	-1.37E-05	8.84E-10	0.644
8	85094979	86979097	352	1.18E-05	6.11E-10	0.634
8	86990451	87562786	212	1.80E-05	3.76E-10	0.354
8	87563878	89083319	540	1.50E-05	1.18E-09	0.662

8	89083979	90605445	456	-7.47E-06	8.74E-10	0.801
8	90607207	92342283	523	2.89E-06	9.26E-10	0.924
8	92344138	94269630	544	4.68E-06	1.32E-09	0.898
8	94272436	95810190	578	-1.16E-05	1.29E-09	0.747
8	95810772	96533604	390	2.50E-05	9.95E-10	0.428
8	96534326	97318660	396	-1.39E-05	1.05E-09	0.666
8	97320605	98867870	622	6.48E-06	1.40E-09	0.862
8	98869940	101450242	830	-5.46E-06	1.53E-09	0.889
8	101451169	102937063	656	-3.28E-05	1.73E-09	0.431
8	102944509	103730879	375	-1.37E-06	1.10E-09	0.967
8	103970185	105389708	566	-1.02E-05	1.15E-09	0.763
8	105391381	107409808	782	1.36E-05	1.84E-09	0.752
8	107410067	108108114	325	1.49E-05	7.05E-10	0.575
8	108115161	108646193	244	4.82E-06	6.42E-10	0.849
8	108646968	110761074	744	-2.04E-05	1.39E-09	0.584
8	110761776	112611637	402	1.83E-05	7.73E-10	0.510
8	112612842	114393054	481	-9.85E-06	8.09E-10	0.729
8	114394256	116095059	617	9.54E-07	1.19E-09	0.978
8	116095815	117130004	271	1.99E-05	5.50E-10	0.395
8	117131947	118622352	563	2.68E-05	1.36E-09	0.469
8	118623844	120216957	719	2.12E-05	1.77E-09	0.614
8	120217356	121767384	601	-1.48E-05	1.19E-09	0.668
8	121770585	123545992	691	1.85E-05	1.76E-09	0.659
8	123547148	124483938	403	8.00E-06	1.12E-09	0.811
8	124486753	125247268	404	6.43E-06	1.11E-09	0.847
8	125248576	126268046	461	2.60E-06	1.23E-09	0.941
8	126269921	127434945	507	-4.43E-06	1.35E-09	0.904
8	127436003	128162974	338	-5.29E-05	1.50E-08	0.666
8	128166556	128542444	193	-3.15E-04	1.83E-08	0.020
8	128543956	129297518	432	-3.83E-05	1.90E-09	0.379
8	129297807	130379330	411	1.84E-05	9.21E-10	0.543
8	130380171	130812339	231	-1.60E-05	5.89E-10	0.510
8	130814803	131553214	285	-2.40E-05	6.30E-10	0.340
8	131553327	132358990	430	1.54E-05	1.08E-09	0.639
8	132359241	133454722	553	-1.13E-05	1.25E-09	0.750
8	133455530	134566686	734	-2.55E-05	1.78E-09	0.546
8	134567348	135269827	424	6.31E-07	1.25E-09	0.986

8	135270868	136209239	515	1.73E-05	1.32E-09	0.633
8	136209325	137306730	483	3.25E-06	1.02E-09	0.919
8	137307361	138223632	347	-6.58E-06	7.39E-10	0.809
8	138224181	139194661	572	3.34E-05	1.31E-09	0.356
8	139195331	140093896	520	4.28E-05	1.51E-09	0.271
8	140094627	141355502	731	-1.10E-05	1.87E-09	0.800
8	141358681	143750205	924	-1.71E-05	2.38E-09	0.725
8	143750619	146303789	716	-1.15E-06	1.51E-09	0.976
9	342841	1006606	533	2.63E-05	1.39E-09	0.480
9	1007923	1792147	678	2.06E-05	1.65E-09	0.612
9	1792277	2665240	685	-4.02E-05	1.99E-09	0.368
9	2666964	3348816	358	-2.96E-05	8.45E-10	0.309
9	3349487	4378727	699	1.86E-05	2.67E-09	0.719
9	4380096	4590305	142	2.37E-05	4.24E-10	0.250
9	4591655	5273194	342	4.68E-05	7.45E-10	0.086
9	5273896	6144065	384	-1.79E-05	8.25E-10	0.533
9	6144333	7232230	569	4.00E-06	1.35E-09	0.913
9	7232510	7757891	465	4.40E-06	1.19E-09	0.898
9	7758579	8235898	325	6.59E-06	8.03E-10	0.816
9	8236122	9374664	803	4.57E-05	2.24E-09	0.335
9	9375194	9793342	253	-1.33E-05	6.32E-10	0.596
9	9793846	10358286	350	6.21E-06	8.70E-10	0.833
9	10358672	10879071	263	2.51E-05	7.27E-10	0.352
9	10879188	11616822	307	-1.59E-06	6.47E-10	0.950
9	11884425	12274220	180	-7.52E-06	3.96E-10	0.705
9	12274774	12778224	274	-2.91E-05	6.40E-10	0.251
9	12780295	13797055	477	-2.00E-05	1.22E-09	0.566
9	13797496	14712257	604	-8.97E-06	2.09E-09	0.844
9	14714681	16054406	644	-3.52E-05	1.45E-09	0.354
9	16056127	16833797	538	2.33E-05	1.45E-09	0.540
9	16836011	17627988	363	1.07E-05	7.53E-10	0.696
9	17628726	18659920	605	-6.86E-06	1.40E-09	0.854
9	18660695	19129349	353	3.09E-06	9.41E-10	0.920
9	19456098	20324812	482	-2.04E-05	1.20E-09	0.556
9	20324848	21549638	502	1.46E-05	1.05E-09	0.653
9	21550959	22968575	596	1.37E-05	1.32E-09	0.706
9	22968789	24154728	439	2.19E-05	1.31E-09	0.545

9	24155142	25425160	409	3.83E-05	1.36E-09	0.298
9	25425511	26317104	404	1.63E-05	1.21E-09	0.639
9	26317973	27335346	535	-1.93E-05	1.35E-09	0.598
9	27336207	28252383	497	-7.75E-06	1.38E-09	0.834
9	28253887	29622939	660	-7.70E-06	1.38E-09	0.836
9	29623053	30819576	414	-8.82E-06	7.16E-10	0.742
9	30821676	31836697	373	-1.99E-05	7.34E-10	0.463
9	31837040	32249649	172	1.41E-07	3.24E-10	0.994
9	32250247	33186788	464	-2.99E-05	9.72E-10	0.338
9	33187897	34769004	522	-2.61E-05	1.07E-09	0.424
9	34771534	36712124	646	-7.88E-06	1.34E-09	0.829
9	36714252	37678932	456	6.02E-05	1.10E-09	0.070
9	37679713	42220384	545	3.16E-05	1.41E-09	0.400
9	69576527	71968825	445	9.97E-06	9.95E-10	0.752
9	71969482	73398448	622	-3.33E-05	1.16E-09	0.329
9	73399750	73916072	309	-1.35E-05	6.03E-10	0.583
9	73916583	74600739	231	1.47E-05	5.71E-10	0.539
9	74600822	75517582	356	-2.75E-05	6.62E-10	0.285
9	75519251	76785168	379	-2.26E-05	7.78E-10	0.417
9	76786297	78214833	562	-2.65E-05	1.30E-09	0.462
9	78215299	78900183	469	-1.43E-05	1.62E-09	0.722
9	81066078	81860861	384	-1.25E-05	9.29E-10	0.681
9	81863722	82348771	246	1.47E-05	5.35E-10	0.525
9	78901112	81064897	968	-4.23E-05	2.27E-09	0.375
9	82349606	83041982	353	1.43E-05	9.01E-10	0.633
9	83043376	83455568	219	9.83E-06	4.84E-10	0.655
9	83455867	84388566	421	1.83E-05	1.03E-09	0.569
9	84389168	86180165	683	-1.38E-06	1.60E-09	0.973
9	86181461	87571563	629	-2.46E-05	1.40E-09	0.511
9	87571959	87811640	150	-7.14E-06	3.19E-10	0.690
9	87813612	88867151	355	1.46E-05	7.25E-10	0.588
9	88869630	89948499	515	-2.65E-05	1.18E-09	0.441
9	89949778	90198251	198	-3.70E-05	3.94E-10	0.062
9	90198375	90893610	338	4.99E-06	8.11E-10	0.861
9	90893774	91608628	247	-1.24E-05	5.69E-10	0.604
9	91610543	92342927	327	9.01E-06	8.11E-10	0.752
9	92443570	93441674	461	-2.55E-05	1.08E-09	0.437

9	93442205	94163515	361	3.24E-05	9.17E-10	0.284
9	94164373	95854872	626	2.68E-05	1.10E-09	0.418
9	95855100	97719592	731	9.70E-06	1.41E-09	0.796
9	97720818	98889276	456	5.23E-06	9.05E-10	0.862
9	98891743	100917602	625	-5.36E-06	1.14E-09	0.874
9	100919318	101473404	376	-1.90E-05	9.75E-10	0.542
9	103544757	103969076	179	3.25E-06	2.73E-10	0.844
9	101474837	103543692	679	-9.12E-05	1.18E-09	0.008
9	103969339	104552711	293	1.60E-05	6.50E-10	0.531
9	104553938	105041375	303	1.50E-05	6.92E-10	0.568
9	105041756	105743445	320	-1.75E-05	6.39E-10	0.489
9	105744075	106487777	333	-2.41E-05	7.41E-10	0.376
9	106488291	107545903	496	-1.45E-06	7.86E-10	0.959
9	107546901	108751162	555	-4.34E-06	1.22E-09	0.901
9	108754828	109972524	499	-3.11E-05	1.18E-09	0.364
9	109974206	111137001	674	3.50E-05	1.67E-09	0.392
9	111138232	111997222	549	1.26E-05	1.12E-09	0.708
9	112001473	112593531	326	-2.91E-05	7.11E-10	0.275
9	112594858	113255402	401	2.19E-05	1.08E-09	0.504
9	113255544	114077164	462	4.95E-05	1.07E-09	0.130
9	114078103	114828332	330	-7.23E-06	7.16E-10	0.787
9	114830330	115391053	230	1.96E-05	3.62E-10	0.303
9	115393806	116148630	399	-1.99E-05	9.31E-10	0.514
9	116149967	117306807	689	-9.02E-05	1.72E-09	0.030
9	117307880	118569429	682	-1.99E-05	1.46E-09	0.602
9	118570426	120030166	743	-3.96E-05	1.74E-09	0.342
9	120030269	121008844	499	7.53E-06	1.07E-09	0.818
9	121009165	121318886	151	-5.03E-06	3.23E-10	0.780
9	121319662	122258204	433	-1.86E-05	8.87E-10	0.532
9	122258577	124870612	937	5.61E-05	1.79E-09	0.185
9	124871322	125542861	336	-1.36E-05	8.19E-10	0.634
9	125545194	126926376	449	-3.37E-05	9.35E-10	0.270
9	126927204	128926989	624	3.48E-05	1.10E-09	0.294
9	128927088	129477470	292	-1.75E-05	8.50E-10	0.549
9	129477886	131429495	582	-2.74E-05	1.04E-09	0.395
9	131430602	132891863	507	4.19E-06	1.21E-09	0.904
9	132895131	133640777	305	4.28E-06	1.03E-09	0.894

9	133642918	134639655	432	-1.07E-05	9.57E-10	0.731
9	134640928	135401279	345	2.34E-06	9.39E-10	0.939
9	135401721	136417159	502	4.93E-05	4.35E-09	0.455
9	136429816	136979539	317	2.54E-05	1.01E-09	0.423
9	136979744	137928846	496	3.44E-05	1.62E-09	0.393
9	137929853	138817142	515	-4.60E-05	1.74E-09	0.269
9	138817178	141146684	725	2.13E-05	1.71E-09	0.606
10	60969	747115	154	-2.56E-08	3.65E-10	0.999
10	747772	1330771	280	-9.20E-06	7.02E-10	0.728
10	1331955	1609370	172	-1.55E-06	4.71E-10	0.943
10	1610839	2104472	389	-1.30E-06	9.97E-10	0.967
10	2106418	3143283	689	-2.37E-05	1.74E-09	0.570
10	3144251	3785605	442	2.09E-05	1.09E-09	0.526
10	3786476	5358542	956	1.99E-05	2.25E-09	0.674
10	5359010	6316910	615	4.74E-05	1.70E-09	0.250
10	6318458	7639684	883	5.58E-05	2.48E-09	0.263
10	9433090	10540465	440	4.93E-05	1.30E-09	0.171
10	7639932	9432277	1108	-2.55E-05	2.57E-09	0.615
10	10542217	12092029	828	-4.15E-05	2.09E-09	0.364
10	12092208	12798954	371	-8.16E-05	1.33E-09	0.025
10	12799286	13718384	597	-7.40E-06	1.55E-09	0.851
10	13719970	14411956	499	-1.75E-05	1.44E-09	0.644
10	14412084	15303527	489	-7.73E-06	1.42E-09	0.837
10	15303983	16443442	476	3.49E-05	9.46E-10	0.257
10	16444693	17591626	664	-1.78E-05	1.60E-09	0.657
10	17591919	19202731	694	-1.44E-05	1.45E-09	0.705
10	19203300	20131614	450	1.61E-05	8.93E-10	0.591
10	20132516	21243558	680	-1.71E-06	1.53E-09	0.965
10	21244087	23696890	631	-2.44E-05	1.42E-09	0.518
10	23698033	25414215	686	-9.11E-06	1.37E-09	0.805
10	25416598	26631987	555	-8.91E-06	1.07E-09	0.785
10	26633775	27667904	411	-3.29E-06	8.24E-10	0.909
10	27668052	28734655	508	-1.25E-05	1.01E-09	0.696
10	28735045	29174963	234	-1.58E-05	4.68E-10	0.466
10	29175101	29961838	535	-1.78E-05	1.39E-09	0.634
10	29962511	30653567	388	6.86E-05	9.81E-10	0.029
10	30654528	31479103	433	1.46E-05	9.50E-10	0.636

10	31479422	33656047	779	1.44E-05	1.34E-09	0.695
10	36020445	36884906	379	8.16E-06	8.85E-10	0.784
10	33656119	36017592	829	-5.57E-06	1.52E-09	0.886
10	36885921	37845308	313	8.69E-07	6.20E-10	0.972
10	38367233	43220539	218	4.03E-07	3.16E-10	0.982
10	43222372	43605902	162	-5.65E-07	4.26E-10	0.978
10	43606687	45247184	692	-1.97E-05	1.30E-09	0.584
10	45247418	46769302	372	5.58E-05	1.91E-09	0.202
10	47768664	49812917	361	-6.10E-06	8.36E-10	0.833
10	49816189	50839403	568	-2.19E-05	1.18E-09	0.524
10	50840736	52086621	209	-1.08E-04	5.86E-09	0.157
10	52086939	52707026	238	-9.58E-06	4.21E-10	0.641
10	52708822	53362540	343	-2.78E-05	7.81E-10	0.320
10	53362932	54413554	512	1.17E-05	1.14E-09	0.729
10	54413909	55334166	523	3.00E-05	1.17E-09	0.381
10	55334740	56204677	453	-9.85E-06	7.70E-10	0.723
10	56207098	56883966	279	-2.27E-05	1.25E-09	0.521
10	56885639	58806290	704	-2.08E-05	1.14E-09	0.538
10	59158617	59822195	312	-1.41E-05	5.33E-10	0.542
10	59822974	60375613	206	-1.21E-05	3.10E-10	0.493
10	60375857	62558737	972	-5.93E-05	1.97E-09	0.182
10	62558842	64263322	626	-1.94E-05	1.27E-09	0.586
10	64263402	65469133	435	1.73E-05	7.70E-10	0.533
10	65735028	66980708	395	-2.42E-05	7.27E-10	0.369
10	66980980	67545544	227	-8.45E-06	4.77E-10	0.699
10	67546331	68429563	514	-3.83E-06	1.11E-09	0.909
10	68430236	69893580	549	8.61E-06	9.70E-10	0.782
10	69896336	71052508	440	1.50E-05	9.29E-10	0.624
10	71052888	72479853	929	5.61E-05	2.73E-09	0.283
10	72480072	73612902	784	-5.19E-05	2.22E-09	0.271
10	73614288	75696752	550	3.81E-06	6.27E-10	0.879
10	75700056	78638957	888	5.42E-06	1.42E-09	0.886
10	78642246	79740262	594	-8.55E-06	1.29E-09	0.812
10	79742617	80874092	594	-2.54E-05	1.69E-09	0.537
10	80874523	81881954	307	4.81E-05	1.35E-09	0.190
10	81882370	82378624	279	1.90E-05	9.08E-10	0.527
10	82379403	82881996	243	-2.02E-05	6.11E-10	0.414

10	82883949	83597042	269	1.10E-05	5.28E-10	0.633
10	83597818	84383108	342	6.68E-06	6.44E-10	0.792
10	84383277	85424102	487	2.32E-05	8.73E-10	0.433
10	85424493	86459792	527	-2.75E-05	1.32E-09	0.448
10	86460169	87285511	356	6.37E-07	6.27E-10	0.980
10	87285907	88450872	497	5.95E-06	1.17E-09	0.862
10	88451869	89845801	371	-1.33E-05	7.97E-10	0.637
10	89846944	90734906	410	-4.03E-05	8.91E-10	0.178
10	90735832	91959588	592	1.68E-05	1.19E-09	0.627
10	91960142	92682235	320	1.58E-05	5.58E-10	0.505
10	92682369	94196248	469	1.23E-05	8.68E-10	0.676
10	94196690	95064828	355	7.17E-06	5.70E-10	0.764
10	95065655	95946938	409	1.38E-06	9.35E-10	0.964
10	95949432	97984754	853	-2.50E-05	1.37E-09	0.499
10	99554895	100240681	333	-2.15E-05	7.05E-10	0.418
10	97985690	99554180	665	-5.54E-05	1.32E-09	0.127
10	100241204	102089663	714	9.52E-06	1.20E-09	0.784
10	102090924	104207799	690	-1.56E-05	1.18E-09	0.650
10	104208761	106139081	683	9.24E-06	1.30E-09	0.798
10	106139294	107874146	733	1.25E-05	1.31E-09	0.731
10	107875182	109577686	742	2.09E-05	1.60E-09	0.601
10	109578567	110311094	318	-1.01E-05	5.85E-10	0.677
10	110311425	112559414	764	-3.89E-05	1.38E-09	0.295
10	112560012	113839866	516	-4.91E-06	8.99E-10	0.870
10	113845990	115698315	912	-4.41E-05	2.22E-09	0.349
10	117705146	118416795	358	2.50E-05	7.14E-10	0.349
10	115700005	117702645	680	3.66E-05	1.23E-09	0.298
10	118417453	119697663	621	2.80E-07	1.58E-09	0.994
10	119698700	120994671	529	-2.20E-05	1.24E-09	0.533
10	120997666	121874991	393	-7.42E-06	8.81E-10	0.803
10	121875531	122805770	507	-2.61E-05	1.86E-09	0.545
10	122807254	123853223	521	1.29E-04	2.69E-09	0.013
10	123855124	124894743	545	-4.72E-07	1.22E-09	0.989
10	124894909	125868860	560	5.71E-06	1.53E-09	0.884
10	125919795	126739513	451	4.67E-05	1.43E-09	0.216
10	126739798	127820978	517	1.23E-05	1.09E-09	0.711
10	127821105	128409952	405	-2.36E-05	1.08E-09	0.472

10	128410393	129482709	634	-2.26E-06	1.67E-09	0.956
10	129483173	130282731	491	3.44E-05	1.33E-09	0.346
10	130282817	131192968	537	-2.45E-05	1.63E-09	0.544
10	131195759	132251820	596	4.96E-05	1.45E-09	0.194
10	132252499	132964299	471	-5.56E-06	1.27E-09	0.876
10	132966372	133355925	285	9.41E-06	9.48E-10	0.760
10	133356417	134858046	638	3.19E-05	1.55E-09	0.419
10	134858720	135524335	243	-6.31E-06	4.92E-10	0.776
11	333347	1057948	321	1.32E-05	6.66E-10	0.608
11	1058583	1861912	309	-1.17E-06	8.38E-10	0.968
11	1862168	2422034	247	1.29E-04	6.33E-09	0.106
11	2423545	3660412	527	-3.68E-05	1.95E-09	0.404
11	3661873	4745931	519	4.58E-06	1.64E-09	0.910
11	4746391	5529152	655	-7.89E-05	1.29E-09	0.028
11	5532222	6010718	357	-8.51E-06	7.56E-10	0.757
11	6011761	6917273	555	-2.37E-05	1.25E-09	0.502
11	6917772	7715871	483	-2.81E-05	1.82E-09	0.509
11	7717884	9068716	668	5.40E-05	1.46E-09	0.158
11	9069046	10370675	440	-2.01E-05	8.32E-10	0.485
11	10371614	11361034	681	-1.42E-05	1.74E-09	0.733
11	11361458	12018942	492	-6.31E-08	1.35E-09	0.999
11	12019010	13133902	717	-3.82E-05	2.15E-09	0.410
11	13134600	14934686	720	3.28E-05	1.29E-09	0.362
11	14936943	16789155	642	-5.62E-05	1.10E-09	0.091
11	16790799	18788126	918	-3.03E-05	1.90E-09	0.486
11	18789820	20113591	792	-3.87E-05	2.10E-09	0.399
11	21773680	23111273	502	3.90E-06	9.95E-10	0.902
11	20115277	21773251	1017	-2.87E-05	2.69E-09	0.580
11	24750113	25106557	163	2.32E-06	3.31E-10	0.899
11	23112584	24749839	709	-4.07E-05	1.67E-09	0.319
11	25109191	26046095	403	-1.92E-05	7.73E-10	0.491
11	26046627	27018778	419	-1.97E-05	8.06E-10	0.487
11	27019873	28741185	545	1.39E-05	9.53E-10	0.652
11	28742220	30056918	404	3.16E-07	8.40E-10	0.991
11	30057440	31853968	577	1.36E-05	8.59E-10	0.644
11	31856654	33383448	567	-7.08E-05	1.19E-09	0.041
11	33384046	34321466	429	-1.44E-06	1.07E-09	0.965

11	34321876	35034307	445	-8.84E-05	1.41E-09	0.019
11	35036029	36117340	598	3.37E-05	1.50E-09	0.383
11	36118027	37838625	746	1.84E-06	1.73E-09	0.965
11	37839347	40357803	640	1.53E-05	1.20E-09	0.658
11	40359663	40716222	154	2.18E-05	3.16E-10	0.220
11	40716823	42243196	591	-6.57E-07	1.19E-09	0.985
11	42244050	42854352	257	-3.21E-06	4.80E-10	0.884
11	42854878	43952796	470	1.32E-05	9.74E-10	0.672
11	46252956	47893120	417	3.58E-07	6.38E-10	0.989
11	43953774	46245900	1084	3.47E-05	2.96E-09	0.523
11	48351331	49659131	215	-2.37E-07	3.00E-10	0.989
11	49660004	55116367	305	-4.33E-06	4.12E-10	0.831
11	55116451	56160712	429	2.51E-06	5.90E-10	0.918
11	56625775	58455947	714	-2.29E-05	1.10E-09	0.490
11	58457495	59615953	298	1.76E-05	4.31E-10	0.396
11	59620206	61870732	816	-2.79E-05	1.74E-09	0.503
11	61873755	63153602	405	-4.56E-07	8.14E-10	0.987
11	63154309	66835194	1068	1.89E-05	2.67E-09	0.715
11	66835372	68515239	414	-1.80E-05	1.11E-09	0.589
11	68521072	69508567	391	1.59E-04	1.52E-08	0.198
11	69509343	70613763	469	-2.44E-05	1.34E-09	0.505
11	70616465	71842303	364	1.20E-05	6.84E-10	0.648
11	71842976	74158869	681	-1.81E-05	1.36E-09	0.625
11	74159542	76431100	889	-2.86E-05	1.98E-09	0.520
11	76432156	77903706	543	4.43E-05	8.68E-10	0.133
11	79723675	80720242	465	-2.69E-05	1.26E-09	0.449
11	77904339	79723318	879	2.78E-05	1.84E-09	0.517
11	80721384	81653514	551	-1.04E-05	1.24E-09	0.768
11	81654617	82844918	481	-1.35E-05	9.97E-10	0.669
11	82847086	83575703	267	3.09E-06	4.37E-10	0.882
11	83576460	84208616	304	2.30E-05	6.58E-10	0.369
11	84212522	84618654	169	-2.71E-06	4.22E-10	0.895
11	84618788	86623913	833	-4.94E-06	1.68E-09	0.904
11	86625137	87429545	370	1.20E-05	6.92E-10	0.648
11	87431647	87827395	167	1.34E-05	4.27E-10	0.517
11	87827514	89208590	526	1.73E-05	1.15E-09	0.609
11	89441680	91115475	483	4.84E-06	8.01E-10	0.864

11	91118661	91676282	198	-1.07E-06	4.25E-10	0.958
11	91677374	92626428	367	-8.67E-06	6.03E-10	0.724
11	92627643	93270431	278	-8.88E-06	5.77E-10	0.711
11	93272186	94389331	421	1.48E-05	7.93E-10	0.600
11	94391845	95329108	415	-7.73E-06	1.10E-09	0.816
11	95332310	96848965	669	-5.66E-05	1.50E-09	0.144
11	96849530	98703542	778	1.24E-05	1.82E-09	0.770
11	98703733	99613901	508	-7.37E-06	1.23E-09	0.834
11	99614560	101156501	814	2.29E-06	1.87E-09	0.958
11	101158517	101892922	344	-1.46E-05	5.65E-10	0.540
11	101894471	102668702	379	-2.21E-05	1.04E-09	0.493
11	102668882	103491310	383	7.05E-07	7.80E-10	0.980
11	103491586	104871322	551	-3.07E-06	1.01E-09	0.923
11	104872103	106251514	419	8.72E-06	6.87E-10	0.739
11	106252171	107489662	537	-4.43E-06	1.28E-09	0.901
11	107491606	108495975	327	-3.33E-05	8.90E-10	0.265
11	108496357	110701645	740	-1.58E-06	1.34E-09	0.966
11	110702636	111981853	469	2.21E-05	8.38E-10	0.446
11	111985737	113103996	469	-2.12E-05	9.11E-10	0.482
11	113105405	113958177	414	-8.63E-05	1.76E-09	0.040
11	113959061	114742322	448	-2.53E-05	1.06E-09	0.439
11	114743956	115627217	414	2.85E-05	9.67E-10	0.360
11	115652723	117097952	689	-1.14E-05	1.77E-09	0.788
11	117098436	118898319	898	2.96E-05	2.04E-09	0.512
11	118899218	120171371	574	8.00E-07	1.40E-09	0.983
11	120173354	121660636	713	-1.16E-05	1.51E-09	0.765
11	121661507	123289851	721	-2.90E-05	1.97E-09	0.513
11	123291301	124403522	618	1.55E-05	1.37E-09	0.675
11	124404297	125984562	770	4.58E-05	1.91E-09	0.295
11	125984970	126772347	528	8.66E-06	1.34E-09	0.813
11	126772905	127715860	459	-6.35E-06	1.07E-09	0.846
11	127716140	129186210	718	-8.12E-07	1.74E-09	0.984
11	129187995	129838375	321	-2.43E-05	9.03E-10	0.418
11	129839528	130892029	437	-3.74E-05	1.10E-09	0.260
11	130894131	131870763	676	2.85E-05	1.84E-09	0.507
11	131871467	132386355	340	-7.73E-06	6.95E-10	0.769
11	132386834	133679495	783	5.19E-05	2.34E-09	0.283

11	133679758	134946382	719	1.76E-05	3.34E-09	0.761
12	68657	665200	216	4.59E-06	5.66E-10	0.847
12	665655	1602602	326	2.78E-05	7.34E-10	0.304
12	1602652	2176046	281	2.13E-05	8.45E-10	0.464
12	2176265	2886299	325	-1.94E-05	8.85E-10	0.515
12	2886946	3558488	354	1.76E-05	1.01E-09	0.578
12	3559429	4693778	656	-3.83E-05	2.02E-09	0.393
12	4695425	5877653	808	2.47E-05	2.04E-09	0.584
12	5878686	6879279	441	2.96E-05	1.08E-09	0.368
12	8658644	10477423	257	4.12E-05	6.12E-10	0.096
12	10477712	11684725	448	-7.12E-06	8.04E-10	0.802
12	11687997	12530718	424	-7.62E-05	1.84E-09	0.076
12	12533494	13362446	381	-2.07E-05	1.15E-09	0.541
12	13362818	14658341	699	1.42E-05	2.14E-09	0.759
12	14659441	16423287	588	2.34E-05	1.15E-09	0.490
12	16423998	18466533	744	-9.83E-06	1.61E-09	0.807
12	18467618	19924288	582	-7.64E-05	1.47E-09	0.046
12	19925541	21568051	878	-1.12E-05	1.71E-09	0.787
12	21569730	22325878	305	-2.77E-05	5.81E-10	0.250
12	22327634	22943718	204	1.48E-05	4.56E-10	0.489
12	22944771	23819079	359	-4.99E-05	1.08E-09	0.128
12	23819364	24545631	412	8.28E-05	1.22E-09	0.018
12	24545834	25991825	716	2.89E-05	1.58E-09	0.468
12	25992543	27529660	775	-2.01E-06	1.86E-09	0.963
12	29111480	30354932	709	-2.65E-05	1.36E-09	0.472
12	27529712	29111136	700	1.18E-05	1.36E-09	0.750
12	30355547	31067009	415	-1.59E-05	7.43E-10	0.559
12	31067490	32081072	408	1.18E-05	1.05E-09	0.717
12	32082695	32645755	311	-7.85E-06	7.74E-10	0.778
12	32645848	33435901	369	-1.14E-05	7.09E-10	0.669
12	33436298	38231063	326	1.30E-06	4.89E-10	0.953
12	38233264	40017827	410	-3.22E-06	5.96E-10	0.895
12	40022029	41015211	475	2.78E-06	8.98E-10	0.926
12	41015333	42346846	665	1.21E-05	1.24E-09	0.732
12	42347363	43074061	261	5.99E-06	4.84E-10	0.785
12	43074434	43982146	422	5.82E-06	7.57E-10	0.832
12	43982342	44922863	220	6.82E-06	4.12E-10	0.737

12	44923334	46022099	343	-5.37E-06	6.23E-10	0.830
12	46023426	47504173	481	-4.71E-05	8.69E-10	0.110
12	47505424	48267360	361	1.93E-05	1.24E-09	0.584
12	48267635	48965461	365	-3.97E-05	1.56E-09	0.315
12	48967935	50160195	315	2.70E-05	8.05E-10	0.341
12	50160662	51582048	414	-5.18E-05	7.52E-10	0.059
12	51582188	52913730	700	3.20E-05	1.34E-09	0.382
12	52914211	54379670	717	4.14E-05	3.06E-09	0.454
12	54380016	56792337	763	-5.27E-05	1.55E-09	0.181
12	56795415	57534912	235	4.04E-05	4.40E-10	0.054
12	57535266	58748272	385	1.42E-06	6.18E-10	0.955
12	58748846	60859665	699	-4.12E-06	1.19E-09	0.905
12	60859809	61738896	266	7.53E-06	3.95E-10	0.705
12	61740561	63158569	602	1.90E-05	1.34E-09	0.603
12	63158712	63908647	359	-3.23E-05	8.09E-10	0.256
12	63910665	65383507	408	-4.40E-05	9.06E-10	0.143
12	65926411	67039427	476	-3.12E-05	8.93E-10	0.297
12	67039600	67817612	375	3.58E-05	9.00E-10	0.232
12	67818111	68740414	516	-2.15E-05	1.23E-09	0.540
12	68742112	69826093	486	-5.27E-05	1.32E-09	0.147
12	69826237	70815755	526	-1.96E-06	1.03E-09	0.951
12	70817279	71688642	413	-4.11E-05	1.01E-09	0.195
12	71690933	72513196	316	-8.88E-07	5.88E-10	0.971
12	72525849	74846997	634	1.11E-05	1.16E-09	0.743
12	75193041	76058346	327	-1.25E-05	6.20E-10	0.617
12	76060283	77098899	451	3.14E-05	9.71E-10	0.314
12	77099081	78398357	624	9.99E-06	1.34E-09	0.785
12	78400884	78925445	243	4.09E-06	6.44E-10	0.872
12	78926240	80944201	511	-1.45E-05	8.99E-10	0.630
12	80945774	83137872	721	-3.32E-06	1.53E-09	0.932
12	83143660	83854442	270	8.33E-06	6.66E-10	0.747
12	83856540	85014953	417	5.12E-06	6.62E-10	0.842
12	85016898	87296535	569	-4.38E-05	8.83E-10	0.141
12	87297692	87905878	166	1.86E-06	3.02E-10	0.915
12	87909726	88848006	218	-4.49E-06	3.88E-10	0.820
12	88851543	91827908	896	6.66E-05	1.82E-09	0.119
12	91827930	93792073	768	-5.39E-05	1.86E-09	0.212

12	93792655	95717265	1015	7.08E-06	2.51E-09	0.888
12	95717497	97439396	838	4.34E-05	1.86E-09	0.314
12	99224060	99816442	206	-3.41E-06	4.37E-10	0.870
12	97439589	99220284	912	-4.68E-05	2.17E-09	0.315
12	99816842	101857521	803	1.92E-05	1.81E-09	0.651
12	101857796	102961329	417	3.12E-05	8.76E-10	0.292
12	102961450	104011319	433	3.64E-05	9.44E-10	0.236
12	105817813	106155478	204	6.38E-06	5.50E-10	0.786
12	104012667	105816376	1163	3.15E-05	2.30E-09	0.512
12	106156001	107521770	612	9.61E-06	1.10E-09	0.772
12	107527013	108504440	424	4.01E-05	8.81E-10	0.176
12	108506696	110111462	700	-6.90E-05	1.80E-09	0.104
12	110112932	113027651	567	8.98E-06	1.06E-09	0.783
12	113030227	113877766	334	4.87E-05	7.36E-10	0.073
12	113885696	114882476	596	6.52E-05	1.45E-09	0.087
12	114883085	115326972	186	1.39E-05	7.00E-10	0.599
12	117087697	117644288	184	5.58E-06	3.99E-10	0.780
12	115329869	117087376	969	1.02E-05	2.54E-09	0.840
12	117644436	118888131	675	7.32E-06	1.64E-09	0.856
12	118888731	119301273	203	1.05E-05	5.43E-10	0.654
12	119303044	120007172	394	-5.78E-05	1.01E-09	0.069
12	120008093	121720040	644	-1.42E-05	1.18E-09	0.679
12	124710996	125416169	363	2.09E-05	1.38E-09	0.573
12	121721831	124710880	880	-2.97E-05	1.39E-09	0.427
12	125417493	126461057	519	-4.92E-06	1.41E-09	0.896
12	126463562	127382559	515	-5.02E-06	1.10E-09	0.880
12	127383648	128141516	402	-1.04E-06	1.24E-09	0.977
12	128141610	128810520	447	-1.24E-06	1.27E-09	0.972
12	128814615	129688509	572	5.71E-05	2.05E-09	0.207
12	129689639	130157939	346	2.14E-06	1.09E-09	0.948
12	130158997	130859973	457	1.80E-05	1.38E-09	0.628
12	130861322	131407836	344	6.15E-06	1.09E-09	0.852
12	131408716	132012266	376	-2.64E-05	9.32E-10	0.387
12	132014206	132706778	276	1.13E-06	6.47E-10	0.965
12	132807034	133841558	235	-8.21E-05	3.65E-09	0.174
13	19020095	20682304	395	2.11E-06	5.81E-10	0.930
13	20684769	21688499	462	1.25E-05	8.99E-10	0.678

13	21688974	22209012	221	7.78E-06	4.48E-10	0.713
13	22211354	23590611	815	-1.67E-05	2.49E-09	0.739
13	23590702	23827019	193	-1.89E-05	6.87E-10	0.471
13	23827815	24477568	384	1.32E-05	8.20E-10	0.645
13	24477971	25210959	503	-4.29E-06	1.23E-09	0.903
13	25212359	25639070	222	-2.43E-05	4.76E-10	0.265
13	25640695	27282973	736	-4.85E-05	1.43E-09	0.199
13	27283115	28283059	562	1.45E-07	1.35E-09	0.997
13	28284459	28876214	285	-1.58E-05	5.59E-10	0.503
13	28879332	29872965	453	-1.67E-05	9.83E-10	0.595
13	29873521	31379027	787	2.62E-05	1.93E-09	0.551
13	31384910	32983250	798	9.97E-06	1.62E-09	0.804
13	32984197	34681078	709	-5.60E-05	1.13E-09	0.096
13	34683262	36042839	439	-1.04E-05	9.88E-10	0.740
13	36043978	37027089	549	5.42E-06	1.06E-09	0.868
13	37029009	38290705	619	3.73E-05	1.07E-09	0.253
13	38291011	38877678	287	1.77E-06	4.89E-10	0.936
13	38887155	40179288	575	-3.03E-05	9.66E-10	0.330
13	40181792	41773356	639	-2.52E-05	1.24E-09	0.474
13	41774489	43099692	548	-1.73E-05	9.47E-10	0.573
13	43099889	44637493	786	2.45E-05	1.43E-09	0.517
13	46493234	46866562	161	-9.15E-06	2.62E-10	0.572
13	44638337	46491101	755	-3.42E-05	1.48E-09	0.374
13	48440893	49987145	462	6.89E-07	6.14E-10	0.978
13	46869621	48439005	725	-8.65E-06	1.34E-09	0.813
13	49987663	51254196	496	-2.41E-05	1.53E-09	0.539
13	51254712	53336663	702	-7.82E-06	1.15E-09	0.818
13	53337952	54681048	611	1.29E-05	1.19E-09	0.709
13	54681363	56433642	544	-1.59E-05	8.09E-10	0.577
13	56434161	58247964	413	-3.61E-06	5.17E-10	0.874
13	60087834	60854106	241	-3.84E-05	3.31E-10	0.035
13	58248124	60085069	714	-9.15E-06	1.32E-09	0.801
13	60855021	61589772	348	2.30E-06	5.79E-10	0.924
13	61591949	62912481	486	-7.75E-06	8.05E-10	0.785
13	62913542	64201284	415	-2.26E-06	7.38E-10	0.934
13	64202212	65339575	366	-1.26E-06	5.24E-10	0.956
13	65340421	66197786	243	3.73E-06	2.94E-10	0.828

13	66200191	67713633	592	2.66E-05	9.69E-10	0.393
13	67714255	69561090	645	1.69E-05	1.12E-09	0.613
13	69561894	70642326	429	3.63E-07	8.56E-10	0.990
13	70643712	71095217	239	5.32E-06	4.81E-10	0.808
13	71095483	71759743	313	-5.99E-06	7.60E-10	0.828
13	71763633	72958993	473	1.08E-05	9.05E-10	0.719
13	72959394	73795534	346	2.52E-07	1.45E-09	0.995
13	73796728	75084163	678	-3.78E-05	1.62E-09	0.348
13	75084473	76504672	666	1.29E-04	2.10E-09	0.005
13	78032193	78807439	318	1.88E-06	5.17E-10	0.934
13	76505282	78030962	671	2.24E-05	1.25E-09	0.527
13	78807836	80252742	553	-1.48E-05	8.60E-10	0.614
13	80257200	82580321	856	2.28E-05	1.52E-09	0.558
13	82580994	84132765	494	1.49E-06	6.33E-10	0.953
13	84133082	85035423	269	-1.45E-05	4.60E-10	0.499
13	85037313	85609664	262	2.35E-07	4.31E-10	0.991
13	85609760	87039195	528	1.54E-05	9.47E-10	0.617
13	87039360	88190520	314	1.04E-05	4.19E-10	0.613
13	88191656	89057861	239	8.44E-06	3.90E-10	0.669
13	89059635	90177618	305	1.36E-05	3.87E-10	0.490
13	90179364	90867683	318	-8.21E-06	6.09E-10	0.740
13	90869873	91967960	405	-4.50E-05	7.38E-10	0.098
13	91970313	92912119	365	5.22E-06	6.36E-10	0.836
13	92912423	93918144	412	-2.31E-05	7.35E-10	0.394
13	93918533	94562549	322	-5.89E-06	5.08E-10	0.794
13	94563601	95540024	474	2.14E-05	1.09E-09	0.518
13	97521207	98589842	367	-3.32E-05	6.58E-10	0.196
13	95540709	97520182	918	-1.60E-05	1.61E-09	0.689
13	98594975	100043997	777	-3.86E-05	1.45E-09	0.311
13	100044673	101392748	563	-1.07E-05	1.12E-09	0.750
13	101394726	102299385	584	1.88E-06	1.10E-09	0.955
13	102299498	103339911	558	2.78E-05	1.06E-09	0.393
13	103340536	104065375	505	1.74E-05	1.05E-09	0.591
13	104065534	104842764	367	-2.60E-06	8.42E-10	0.928
13	104843186	105990071	584	5.05E-06	1.49E-09	0.896
13	105990812	106439396	245	4.94E-06	5.62E-10	0.835
13	106442608	107785431	712	-1.74E-05	1.75E-09	0.678

13	107788025	108518444	439	4.99E-05	1.12E-09	0.136
13	108519987	109359792	437	-7.22E-06	1.10E-09	0.827
13	109359834	110338482	539	4.78E-05	1.49E-09	0.216
13	110338913	111432821	729	-2.86E-05	1.96E-09	0.519
13	111434814	113266084	883	-1.20E-05	2.02E-09	0.790
13	113269127	115109853	671	-9.50E-06	1.24E-09	0.788
14	20544512	20877114	188	-2.06E-05	4.79E-10	0.347
14	20878890	21267077	308	2.27E-05	1.34E-09	0.536
14	21271573	22378835	523	-5.26E-05	1.46E-09	0.169
14	22379132	22738105	229	3.60E-05	6.19E-10	0.148
14	22738161	23607730	506	-2.56E-05	1.42E-09	0.496
14	23607866	24904154	516	-5.82E-06	1.59E-09	0.884
14	26135167	26679549	244	6.75E-06	5.94E-10	0.782
14	24904240	26134815	654	3.87E-05	1.25E-09	0.275
14	26680794	28466225	589	-1.94E-05	1.19E-09	0.575
14	28466533	29196856	255	1.27E-05	5.10E-10	0.575
14	29198059	32014712	797	3.28E-06	1.81E-09	0.939
14	32016504	33033695	420	-5.04E-06	1.01E-09	0.874
14	36345767	36682357	143	1.03E-06	2.49E-10	0.948
14	34769883	36344816	498	1.78E-05	9.58E-10	0.566
14	33036081	34769821	1119	-1.13E-04	3.39E-09	0.051
14	36683516	38481516	689	4.40E-05	1.95E-09	0.318
14	38486187	39370633	361	-2.31E-05	7.01E-10	0.384
14	39370726	40336296	361	-4.89E-06	6.62E-10	0.849
14	40336990	41615299	437	-1.95E-06	7.11E-10	0.942
14	41615331	43065019	495	1.78E-05	8.17E-10	0.533
14	43068948	44541699	471	5.82E-06	1.12E-09	0.862
14	44542368	45247021	304	1.53E-05	6.25E-10	0.540
14	45250418	46311209	221	-9.63E-06	2.74E-10	0.561
14	46312678	47456738	355	1.26E-06	5.34E-10	0.957
14	47457846	48351664	389	7.01E-07	7.55E-10	0.980
14	48352461	49001226	256	-9.55E-06	5.95E-10	0.695
14	49004194	49955015	359	6.48E-06	6.54E-10	0.800
14	52078316	52761693	415	-1.47E-06	9.65E-10	0.962
14	49955381	52077965	934	3.12E-06	2.01E-09	0.944
14	52762622	54162101	504	2.19E-05	2.05E-09	0.629
14	54162655	55043520	312	1.73E-05	7.23E-10	0.520

14	55044905	56215082	474	-3.41E-05	1.11E-09	0.307
14	56215477	56949485	467	1.14E-05	8.90E-10	0.703
14	58449526	59135770	213	-9.86E-06	3.89E-10	0.617
14	56949644	58448681	677	2.01E-05	1.54E-09	0.608
14	63166807	63890839	304	8.79E-06	5.59E-10	0.710
14	61651278	63164649	666	3.41E-05	1.56E-09	0.388
14	63897095	65218457	418	-2.00E-05	6.70E-10	0.440
14	59135989	61649612	944	-2.94E-05	1.57E-09	0.458
14	65220775	66353557	440	7.77E-08	7.80E-10	0.998
14	66354477	67990648	525	1.17E-05	6.46E-10	0.645
14	67991028	69355606	630	-4.41E-05	2.01E-09	0.326
14	71132759	72213258	357	-1.95E-05	5.30E-10	0.398
14	69356353	71131097	787	7.26E-05	2.15E-09	0.117
14	72214790	72888979	421	2.90E-06	8.14E-10	0.919
14	72889376	74250730	550	1.30E-05	1.22E-09	0.710
14	74252993	75747118	538	-1.65E-05	7.76E-10	0.554
14	75747258	76583135	371	6.18E-06	8.20E-10	0.829
14	76583775	77332160	386	6.05E-06	1.02E-09	0.850
14	77332408	78421884	573	9.13E-06	1.17E-09	0.790
14	78422260	79026816	371	-9.54E-06	7.94E-10	0.735
14	80999361	81787255	327	9.83E-06	6.03E-10	0.689
14	79027504	80998547	757	4.74E-05	1.55E-09	0.229
14	81787481	82459297	259	1.35E-05	5.89E-10	0.579
14	84087979	85035235	352	-2.63E-05	6.70E-10	0.309
14	82461794	84087895	607	-2.43E-05	1.39E-09	0.514
14	85037974	86051751	438	-1.07E-05	1.06E-09	0.743
14	86053777	87334624	501	2.07E-05	9.97E-10	0.512
14	87335256	88641174	451	1.39E-05	8.28E-10	0.628
14	88641335	90069965	638	2.28E-05	1.31E-09	0.530
14	90075441	92098486	749	-1.80E-05	1.82E-09	0.673
14	92099719	93378838	646	4.89E-05	1.52E-09	0.209
14	93379151	94420996	456	-4.90E-05	1.07E-09	0.133
14	94421488	95347359	703	3.19E-05	1.62E-09	0.428
14	95347761	96250900	641	-7.30E-05	1.66E-09	0.073
14	96251418	97570260	662	2.11E-05	1.64E-09	0.603
14	97571437	98987472	651	-1.92E-05	1.46E-09	0.615
14	98989483	99980087	544	-6.06E-05	1.56E-09	0.125

14	99981105	101244250	481	-1.08E-05	1.02E-09	0.736
14	101244293	102339561	444	2.78E-05	1.48E-09	0.470
14	104761778	105440784	241	2.36E-05	6.14E-10	0.340
14	102341650	104759919	714	1.43E-04	2.06E-09	0.002
14	105443695	106552839	140	8.42E-07	2.76E-10	0.960
15	22362658	23288776	160	-1.18E-05	4.62E-10	0.582
15	23292175	24320772	301	-1.48E-05	8.59E-10	0.613
15	24321414	24976269	148	-3.77E-06	3.49E-10	0.840
15	25107905	25750941	208	6.25E-06	4.79E-10	0.775
15	25752114	26185554	310	-4.62E-05	9.49E-10	0.134
15	26186113	26675188	259	3.66E-05	7.86E-10	0.192
15	26676258	27265824	242	-1.21E-06	7.05E-10	0.964
15	27266835	27819408	254	-3.47E-06	6.28E-10	0.890
15	27820352	28325439	266	-5.82E-06	6.17E-10	0.815
15	28326536	29617158	230	-4.37E-06	4.69E-10	0.840
15	29617517	31314317	411	4.23E-05	1.14E-09	0.210
15	31315570	32177326	354	5.08E-05	1.38E-09	0.172
15	32180607	33251727	348	1.00E-05	9.33E-10	0.742
15	33253484	33792116	378	-2.39E-05	1.09E-09	0.469
15	33793179	34273308	356	1.37E-06	9.89E-10	0.965
15	34273346	34970673	239	-1.55E-05	5.19E-10	0.495
15	34971983	35941864	367	-4.03E-06	6.94E-10	0.878
15	35943377	36424581	299	-2.32E-06	6.57E-10	0.928
15	36425448	36968033	285	-9.91E-06	7.99E-10	0.726
15	36968291	37930544	442	1.28E-05	9.94E-10	0.684
15	37931874	38804104	337	-1.39E-05	7.29E-10	0.607
15	38805185	39518563	386	1.40E-05	8.83E-10	0.638
15	39519092	40382441	407	-1.63E-05	8.77E-10	0.583
15	40383779	41211827	277	-3.87E-06	7.85E-10	0.890
15	41212265	42222063	300	1.21E-05	4.19E-10	0.556
15	42222390	43473907	378	-9.29E-06	5.35E-10	0.688
15	43474655	44988610	261	7.61E-06	2.78E-10	0.648
15	44992475	46292382	424	-3.11E-05	6.81E-10	0.234
15	46294385	47000090	290	1.80E-05	4.85E-10	0.414
15	47000857	47977097	382	2.39E-06	6.42E-10	0.925
15	47979127	49075918	359	1.52E-05	5.66E-10	0.522
15	49078966	50004817	260	1.79E-05	3.55E-10	0.342

15	51682775	52589995	273	1.52E-05	3.17E-10	0.394
15	50006184	51681073	716	3.77E-05	1.12E-09	0.258
15	52590615	53014125	170	1.07E-05	2.49E-10	0.497
15	53015101	53766542	361	-8.01E-06	7.55E-10	0.771
15	53767876	54336662	313	-5.62E-06	6.36E-10	0.824
15	54337243	55134743	392	1.39E-05	7.99E-10	0.624
15	55135408	55469163	140	1.13E-05	2.52E-10	0.476
15	55470187	55877891	151	2.21E-05	3.16E-10	0.213
15	55879453	56784345	319	2.92E-05	5.63E-10	0.218
15	56784506	57637678	235	3.56E-06	3.16E-10	0.841
15	57638075	58438535	510	-1.24E-05	1.06E-09	0.704
15	58439935	59505191	502	-3.03E-06	1.11E-09	0.928
15	59507391	60018662	244	6.99E-05	4.88E-10	0.002
15	60019136	60670149	285	1.85E-05	6.17E-10	0.455
15	60670287	61803306	669	-4.44E-06	1.82E-09	0.917
15	61803892	62735671	460	-7.09E-06	8.73E-10	0.810
15	62737452	64335240	693	-3.67E-05	1.35E-09	0.318
15	65104668	66358474	354	3.09E-05	4.82E-10	0.159
15	66359486	66948204	296	3.19E-06	7.24E-10	0.906
15	68642047	69936117	406	1.93E-05	1.13E-09	0.565
15	66948951	68640917	759	-3.71E-05	1.27E-09	0.297
15	69938058	70772135	450	9.97E-05	1.22E-09	0.004
15	70772414	71919256	463	-2.24E-05	9.03E-10	0.457
15	74453873	75516403	321	-1.17E-04	7.85E-10	0.000
15	71920065	74447996	707	-4.76E-05	1.14E-09	0.160
15	75520945	76397358	160	-4.64E-06	2.51E-10	0.769
15	76397390	77910873	428	-1.25E-05	5.50E-10	0.594
15	77912408	78637877	276	-8.77E-07	6.88E-10	0.973
15	78640760	79259009	192	-9.74E-06	3.67E-10	0.611
15	79261077	79991408	437	1.43E-05	8.77E-10	0.629
15	79992629	80635045	302	-1.78E-05	7.50E-10	0.517
15	80637749	81604871	477	-2.28E-05	1.16E-09	0.504
15	81607249	84062334	626	1.17E-05	1.17E-09	0.733
15	84063245	85517022	507	-7.20E-07	1.05E-09	0.982
15	85518062	86341812	361	-1.96E-05	5.25E-10	0.392
15	86342119	86650301	159	1.65E-05	3.45E-10	0.375
15	86650331	87113409	248	-8.55E-07	5.24E-10	0.970

15	87114392	87693557	314	-7.64E-06	8.02E-10	0.787
15	87693885	88032107	160	-9.17E-06	3.51E-10	0.625
15	88032736	88742960	322	-2.95E-06	6.86E-10	0.910
15	88743108	89561223	336	-1.21E-05	8.71E-10	0.682
15	89562631	90452882	361	8.10E-06	6.55E-10	0.752
15	90453183	91221307	299	1.32E-05	5.68E-10	0.579
15	91226586	91745246	251	-1.83E-05	6.87E-10	0.484
15	91749626	92164207	203	3.80E-05	6.59E-10	0.139
15	92165068	92667377	253	-5.07E-06	6.74E-10	0.845
15	92669184	93590376	566	2.94E-05	1.36E-09	0.425
15	93591893	93908402	232	-3.88E-07	9.74E-10	0.990
15	93909052	94547933	368	1.30E-06	9.53E-10	0.966
15	94548924	95576904	643	3.82E-06	1.85E-09	0.929
15	95577096	96497729	429	-1.88E-05	1.21E-09	0.589
15	96500358	97091511	333	2.07E-06	9.24E-10	0.946
15	97092654	98496698	629	8.38E-05	1.94E-09	0.057
15	98497881	99128454	384	-8.46E-06	1.14E-09	0.802
15	100078196	100456641	152	-1.30E-05	3.42E-10	0.483
15	99128932	100077720	485	-3.10E-06	1.25E-09	0.930
15	100457692	101403655	489	3.10E-06	1.29E-09	0.931
15	101408616	101918220	304	2.20E-05	7.39E-10	0.419
15	101918830	102516259	287	3.49E-05	7.90E-10	0.215
16	60291	596605	208	-7.65E-06	5.89E-10	0.753
16	598838	945806	136	-6.35E-06	3.63E-10	0.739
16	946205	1547999	261	-6.65E-06	7.47E-10	0.808
16	1551082	2293296	333	2.82E-05	6.92E-10	0.284
16	2294890	3960458	489	-1.53E-05	1.26E-09	0.666
16	3961770	4689471	275	-4.54E-06	8.77E-10	0.878
16	4690187	5114499	203	3.78E-05	4.95E-10	0.090
16	5115228	5854496	426	-2.21E-05	1.21E-09	0.525
16	5855169	6264138	318	1.60E-05	1.08E-09	0.626
16	6264139	7045506	647	2.82E-05	1.80E-09	0.506
16	7046430	7524343	442	1.10E-05	1.21E-09	0.752
16	7525825	8236887	555	-2.37E-06	1.55E-09	0.952
16	8237157	8572350	256	2.80E-05	8.41E-10	0.334
16	8572557	8960864	188	9.16E-06	6.31E-10	0.715
16	8962907	9658861	335	-7.01E-05	1.35E-09	0.056

16	9659584	9997178	212	1.60E-05	5.02E-10	0.475
16	10002419	10430046	325	8.79E-06	8.14E-10	0.758
16	10431221	11493266	531	-2.35E-05	1.43E-09	0.535
16	11612436	12151685	190	1.63E-05	5.27E-10	0.479
16	12151797	12775407	451	1.38E-06	1.01E-09	0.965
16	12775897	13119544	140	-3.89E-06	3.97E-10	0.845
16	13120367	13978116	506	8.68E-07	1.28E-09	0.981
16	13979558	15885158	356	-8.37E-06	8.35E-10	0.772
16	15885866	16220858	240	6.04E-06	5.27E-10	0.792
16	16222763	17334499	272	2.55E-06	7.69E-10	0.927
16	17335780	18062164	300	3.88E-05	8.11E-10	0.174
16	18065944	20054371	508	-1.99E-05	1.20E-09	0.564
16	20055884	21235343	435	-8.95E-06	1.02E-09	0.779
16	21237217	23369433	478	3.10E-05	1.24E-09	0.379
16	23371110	24308825	377	-6.77E-06	8.81E-10	0.820
16	24309108	25589623	527	1.42E-05	1.28E-09	0.691
16	26683251	26943239	138	-3.14E-05	6.38E-10	0.213
16	25590147	26681997	620	-3.20E-05	1.87E-09	0.459
16	26944850	27445360	274	3.06E-05	9.81E-10	0.329
16	27446054	29023966	445	2.22E-05	8.53E-10	0.447
16	29694822	31380596	353	-4.46E-05	8.42E-10	0.124
16	31384554	47981754	267	-3.65E-05	5.48E-10	0.119
16	47982405	49006844	259	1.16E-06	7.14E-10	0.965
16	49007408	50694011	658	3.92E-05	1.87E-09	0.365
16	50698071	51700822	458	1.29E-05	1.27E-09	0.718
16	53848448	54671719	496	-3.71E-05	2.28E-09	0.437
16	51703888	53845487	830	7.48E-05	1.85E-09	0.082
16	54673195	55505040	496	-1.44E-05	1.38E-09	0.699
16	55506137	56906006	604	-7.94E-05	1.36E-09	0.031
16	56908045	57663112	346	-2.95E-05	1.12E-09	0.379
16	57663619	58508278	391	-3.16E-05	2.00E-09	0.479
16	59889269	60744157	301	-3.74E-06	7.72E-10	0.893
16	58510034	59888170	534	2.87E-05	1.12E-09	0.391
16	60745208	61991704	352	-1.65E-05	7.54E-10	0.548
16	61993462	63395786	493	-6.36E-05	1.38E-09	0.087
16	63396006	64714759	477	-5.48E-05	1.02E-09	0.087
16	64716851	65538017	376	-1.42E-05	7.57E-10	0.605

16	65538484	68429077	907	-1.09E-06	1.89E-09	0.980
16	72915753	73542956	354	2.34E-05	1.22E-09	0.504
16	68429894	70885818	619	-3.52E-05	1.12E-09	0.293
16	70887506	72915425	541	-5.72E-05	9.99E-10	0.070
16	73543220	74724933	470	2.05E-05	1.17E-09	0.550
16	74730819	75517115	291	6.16E-07	7.22E-10	0.982
16	75518129	76056374	169	-9.45E-06	3.82E-10	0.629
16	76058697	76987262	437	-7.85E-06	9.53E-10	0.799
16	76988130	77455781	322	1.65E-05	8.49E-10	0.572
16	77456244	77688760	199	-4.16E-06	4.90E-10	0.851
16	77689074	78446696	584	-6.39E-07	1.71E-09	0.988
16	78452348	78709939	217	-4.06E-05	6.78E-10	0.119
16	78710328	79368760	703	6.70E-06	2.18E-09	0.886
16	79369779	80184442	562	-6.01E-05	1.41E-09	0.110
16	80184495	81209234	657	-4.92E-05	1.54E-09	0.211
16	82482458	83078581	571	2.50E-05	1.75E-09	0.551
16	81209631	82481987	785	1.03E-04	2.57E-09	0.041
16	84646612	85146709	319	-1.64E-05	1.07E-09	0.616
16	83078959	83971446	826	1.73E-05	2.39E-09	0.724
16	83972415	84645588	626	1.08E-05	2.31E-09	0.822
16	85146935	85663943	211	-2.98E-05	1.01E-09	0.347
16	85664453	86221644	412	3.79E-05	1.44E-09	0.319
16	86745955	87173290	254	1.00E-06	1.03E-09	0.975
16	86222417	86745679	508	-5.26E-06	1.87E-09	0.903
16	87175053	88389287	532	-2.99E-06	1.88E-09	0.945
16	88439429	88964934	242	-1.62E-05	8.68E-10	0.583
16	88966667	89604610	217	1.56E-05	6.32E-10	0.536
16	89605495	90292767	240	-9.64E-07	6.38E-10	0.970
17	828	296442	133	2.00E-05	1.10E-09	0.546
17	396660	820493	179	8.80E-05	1.78E-09	0.037
17	820511	1480393	260	-1.98E-05	1.26E-09	0.577
17	1481897	2613925	472	-6.75E-06	1.64E-09	0.868
17	2616352	3425788	387	-8.32E-06	1.58E-09	0.834
17	3426133	4321755	384	-6.94E-06	1.31E-09	0.848
17	4322053	4650487	176	2.85E-05	8.93E-10	0.340
17	4652156	5573766	371	8.21E-06	1.19E-09	0.812
17	5574433	6570522	608	8.51E-06	2.49E-09	0.865

17	6572170	6975193	181	1.93E-05	7.78E-10	0.489
17	6976284	7723513	318	5.25E-05	1.47E-09	0.171
17	8705612	9229793	253	-3.63E-05	1.44E-09	0.338
17	7725660	8704551	399	1.70E-05	2.71E-09	0.743
17	9233113	9714549	223	-4.01E-06	8.79E-10	0.893
17	9714995	10573151	522	7.67E-06	2.00E-09	0.864
17	10573457	11667346	523	3.08E-05	2.01E-09	0.492
17	11668104	13000883	588	-1.75E-05	3.32E-09	0.761
17	13001700	13625425	351	2.15E-05	1.54E-09	0.584
17	13626102	14209988	323	-1.10E-05	1.28E-09	0.759
17	14210015	14499605	185	-1.11E-05	7.93E-10	0.694
17	14500054	14859339	269	1.39E-05	1.00E-09	0.660
17	14860784	16319512	496	6.35E-05	1.73E-09	0.127
17	16321959	17292491	308	1.11E-05	1.09E-09	0.737
17	17299591	18446092	382	-2.34E-05	1.06E-09	0.472
17	18456679	19666001	259	8.48E-06	8.25E-10	0.768
17	20679485	21284673	159	-1.52E-06	7.59E-10	0.956
17	21285166	25677370	211	-6.08E-06	6.18E-10	0.807
17	25679294	26272308	203	-1.52E-06	6.21E-10	0.952
17	26273607	27884779	447	-2.27E-05	1.27E-09	0.523
17	27885675	29259899	376	-7.27E-06	1.16E-09	0.831
17	29262773	30878805	457	2.38E-05	1.49E-09	0.538
17	30879433	31989867	616	-4.68E-05	2.19E-09	0.317
17	31990385	32677709	410	1.04E-05	1.40E-09	0.781
17	32677947	33614452	507	-1.16E-05	1.83E-09	0.785
17	33616080	34393028	274	-2.68E-05	8.73E-10	0.364
17	34393296	35725997	364	-1.50E-06	1.21E-09	0.966
17	37344586	38649203	332	-1.80E-05	1.28E-09	0.614
17	35731855	37341235	493	9.68E-05	1.42E-08	0.416
17	38652220	39657179	455	8.42E-06	1.26E-09	0.813
17	39657337	40231221	206	-2.13E-06	5.92E-10	0.930
17	40232810	41769732	392	-5.07E-06	1.07E-09	0.877
17	41771040	43317574	552	-1.58E-05	1.71E-09	0.703
17	44863413	45874715	302	2.33E-06	8.99E-10	0.938
17	45876022	46828302	406	-1.12E-05	1.48E-09	0.770
17	46828412	48027295	402	2.49E-05	2.09E-09	0.586
17	48027688	48832008	360	1.42E-05	1.58E-09	0.721

17	48832437	49892805	428	-3.78E-05	1.31E-09	0.296
17	49894599	51282380	527	-2.02E-06	1.43E-09	0.957
17	52863321	53338298	227	1.14E-05	7.39E-10	0.675
17	51284441	52861905	537	-4.74E-05	1.72E-09	0.254
17	53346718	54164464	385	-9.08E-06	1.20E-09	0.793
17	54164935	55278525	429	-2.13E-05	1.53E-09	0.586
17	57487538	59307733	303	-1.65E-05	8.19E-10	0.565
17	55279508	57486793	880	4.72E-05	3.09E-09	0.396
17	59309085	60883279	464	-1.34E-05	1.47E-09	0.726
17	60885287	62035655	261	-1.01E-05	6.86E-10	0.701
17	62037685	64434272	754	9.32E-06	2.45E-09	0.851
17	64434524	65013985	328	1.34E-05	1.22E-09	0.702
17	65016683	66315468	382	-7.22E-05	1.41E-09	0.054
17	66318466	66903293	310	2.97E-05	1.11E-09	0.374
17	66906217	68199133	460	7.12E-06	1.48E-09	0.853
17	68199783	68875761	304	-5.28E-06	1.02E-09	0.869
17	68877746	69242135	206	-1.02E-04	2.37E-09	0.036
17	69242932	70147289	397	-5.64E-05	1.92E-09	0.198
17	70148198	70947580	477	2.70E-06	2.07E-09	0.953
17	70948535	71825523	480	1.78E-05	1.94E-09	0.687
17	71829519	72740027	438	-7.08E-07	1.74E-09	0.986
17	72742484	74016834	403	-2.92E-05	1.28E-09	0.413
17	74018640	74909405	368	2.93E-05	1.21E-09	0.400
17	74910943	75471247	314	7.72E-05	1.43E-09	0.041
17	75476450	75842792	220	4.87E-07	1.09E-09	0.988
17	75844316	76445231	219	1.98E-05	1.01E-09	0.534
17	76446689	77093768	322	-1.81E-05	1.33E-09	0.620
17	77094717	77468875	306	6.16E-05	1.57E-09	0.119
17	78780686	79683249	363	-3.57E-05	1.38E-09	0.336
17	77471383	78780080	510	3.93E-05	1.94E-09	0.373
17	79683698	80969550	416	-2.56E-05	1.26E-09	0.470
18	349293	906343	394	-3.53E-06	1.04E-09	0.913
18	906673	1940581	435	-9.02E-06	9.95E-10	0.775
18	1940863	2206099	166	-4.45E-05	1.07E-09	0.173
18	2206891	2889826	315	2.18E-05	7.65E-10	0.430
18	2891847	3357120	219	-2.39E-05	6.53E-10	0.350
18	3359237	3861997	218	-3.01E-07	8.77E-10	0.992

18	3862461	4597021	433	-9.84E-06	1.24E-09	0.780
18	4601573	5197395	229	2.93E-05	6.13E-10	0.237
18	5198562	5852964	327	-3.04E-05	7.92E-10	0.281
18	5855598	6630394	361	1.23E-05	1.14E-09	0.716
18	6630840	7373590	414	3.58E-05	1.10E-09	0.281
18	7374066	8440357	509	2.83E-05	1.06E-09	0.384
18	8440558	9414901	548	7.79E-05	1.41E-09	0.038
18	10465005	11028008	322	2.57E-05	6.36E-10	0.309
18	9415389	10462537	665	-2.50E-06	2.02E-09	0.956
18	11028129	11529011	226	-2.74E-06	5.85E-10	0.910
18	11530996	12737037	468	2.05E-05	8.66E-10	0.486
18	12737582	14358413	604	-3.36E-05	1.15E-09	0.323
18	18527782	21622994	743	3.35E-05	1.48E-09	0.383
18	21623416	22745505	571	2.82E-05	1.29E-09	0.432
18	22746547	24088696	488	-3.25E-05	9.46E-10	0.291
18	24088831	24971742	490	-3.44E-05	7.95E-10	0.223
18	24972728	26873513	697	3.99E-05	1.20E-09	0.251
18	26874132	28037427	397	-3.87E-06	6.43E-10	0.879
18	28037840	28560707	235	2.36E-05	4.85E-10	0.283
18	28561634	29563172	480	9.56E-06	8.98E-10	0.750
18	31777336	32456417	204	-1.81E-06	3.05E-10	0.918
18	29564408	31774120	674	2.76E-05	9.90E-10	0.380
18	32457437	33791804	485	5.59E-06	8.23E-10	0.846
18	35073616	35837704	322	7.84E-06	5.48E-10	0.738
18	33792671	35072855	572	-1.69E-05	9.39E-10	0.580
18	35838871	36364691	241	2.52E-05	4.87E-10	0.254
18	36365021	37682827	411	-4.25E-06	6.58E-10	0.868
18	37683967	38766853	525	5.96E-05	1.58E-09	0.134
18	38767938	39891215	366	-6.17E-06	6.03E-10	0.802
18	43295347	43881224	239	-4.33E-06	3.55E-10	0.818
18	42606091	43295247	413	5.83E-05	8.15E-10	0.041
18	39892281	42606024	955	9.12E-05	1.57E-09	0.021
18	43881706	45313390	679	1.07E-05	1.18E-09	0.755
18	45314528	46208355	424	6.15E-05	9.91E-10	0.051
18	46209339	47262925	482	1.76E-05	9.50E-10	0.568
18	47263681	47814821	330	1.66E-05	5.50E-10	0.480
18	47816777	48784027	395	-9.45E-06	6.62E-10	0.714

18	50152229	51059341	375	3.09E-06	5.98E-10	0.899
18	48784953	50151479	573	2.90E-05	1.19E-09	0.401
18	51059850	51952586	345	1.00E-05	4.98E-10	0.653
18	51953945	52707502	198	-1.97E-05	5.08E-10	0.381
18	52709949	53763665	408	1.24E-05	8.16E-10	0.664
18	53764157	55089715	500	7.84E-06	8.59E-10	0.789
18	55090919	55808073	393	2.35E-05	9.46E-10	0.444
18	55808692	56752598	490	1.93E-05	1.03E-09	0.548
18	56754050	57504565	479	1.98E-05	1.04E-09	0.540
18	57505073	58816387	484	-1.02E-06	8.45E-10	0.972
18	58817565	59585320	377	6.62E-06	8.80E-10	0.823
18	60823554	61439643	276	-1.38E-06	4.96E-10	0.950
18	59585959	60822264	549	-1.46E-05	1.02E-09	0.648
18	61441801	62075853	293	-8.00E-06	4.46E-10	0.705
18	62078305	63110788	403	1.83E-09	7.57E-10	1.000
18	63111628	64250279	397	-3.62E-06	6.46E-10	0.887
18	65830606	66404071	323	2.09E-08	5.91E-10	0.999
18	64251305	65830031	736	6.89E-06	1.53E-09	0.860
18	66404118	67569547	516	-1.31E-05	1.28E-09	0.714
18	67574191	69038112	511	-2.09E-05	1.21E-09	0.548
18	70094975	70717286	329	4.23E-06	7.43E-10	0.877
18	69039235	70094707	485	4.03E-06	1.12E-09	0.904
18	70719886	71243399	284	1.56E-05	6.84E-10	0.552
18	71244692	71892202	389	-5.46E-05	1.22E-09	0.119
18	71892443	72519795	346	1.27E-05	6.96E-10	0.630
18	72521276	73897594	733	-8.43E-06	1.52E-09	0.828
18	75029584	75473982	288	1.61E-05	7.30E-10	0.551
18	73898846	75028501	623	-3.54E-05	1.74E-09	0.397
18	75474664	75975253	233	2.07E-05	6.09E-10	0.401
18	75976567	77151520	503	-4.02E-05	1.33E-09	0.269
18	77151867	78017074	335	1.43E-05	6.99E-10	0.589
19	157892	757412	228	-1.57E-05	1.30E-09	0.663
19	759261	1089241	159	-3.26E-05	7.87E-10	0.245
19	1090803	1920712	265	7.49E-05	1.59E-09	0.061
19	1922476	2815372	342	-3.11E-06	1.40E-09	0.934
19	2816339	3301333	213	1.97E-05	9.40E-10	0.520
19	3304779	4347573	350	-5.04E-06	1.88E-09	0.907

19	4349416	5191547	337	3.18E-05	1.52E-09	0.415
19	5192372	5766090	218	-4.46E-05	1.09E-09	0.176
19	5767585	6612779	264	-3.48E-06	1.04E-09	0.914
19	6614637	7309688	317	1.14E-05	1.42E-09	0.761
19	7312727	8684707	486	-1.95E-05	2.10E-09	0.671
19	9013231	10029966	390	6.34E-06	1.13E-09	0.850
19	10030690	11279257	438	1.69E-06	2.50E-09	0.973
19	11280183	12497411	332	6.40E-07	1.06E-09	0.984
19	12500149	13903388	410	-1.73E-05	1.48E-09	0.654
19	15553878	16251518	445	4.38E-06	1.36E-09	0.906
19	13906229	15549939	595	-1.53E-05	2.21E-09	0.744
19	17944900	18506666	243	2.50E-05	9.48E-10	0.416
19	18506815	19873269	430	-1.07E-05	1.29E-09	0.767
19	16252072	17942927	622	-6.58E-06	2.38E-09	0.893
19	19873640	20520392	140	1.22E-05	4.44E-10	0.564
19	21379657	22036399	158	-6.65E-06	4.52E-10	0.754
19	22037866	22803640	245	-1.43E-05	7.05E-10	0.589
19	22805247	24059424	282	1.63E-06	7.36E-10	0.952
19	24059507	28555771	270	-5.12E-06	7.92E-10	0.856
19	28556284	28917565	189	-4.49E-06	5.52E-10	0.848
19	30113991	30858084	259	1.20E-05	1.03E-09	0.708
19	29318443	30113252	466	2.86E-07	1.50E-09	0.994
19	30858965	32136133	463	-8.05E-05	1.53E-09	0.039
19	33764103	34067891	139	4.35E-06	5.35E-10	0.851
19	32145233	33763475	600	7.07E-05	1.93E-09	0.108
19	35333176	36045772	366	3.68E-06	1.37E-09	0.921
19	34068435	35331436	447	-1.00E-05	1.59E-09	0.801
19	39371014	39757572	147	-8.68E-06	7.87E-10	0.757
19	36047098	37454119	411	3.40E-05	1.75E-09	0.416
19	41442597	41841098	165	8.30E-06	4.88E-10	0.707
19	37455512	39370456	576	5.14E-05	3.28E-09	0.370
19	39757926	41440863	613	-5.52E-05	2.00E-09	0.217
19	41843461	43954915	369	-6.65E-05	2.58E-09	0.190
19	43956095	44880657	396	-8.35E-06	1.20E-09	0.810
19	44882157	45255679	171	1.51E-05	7.77E-10	0.589
19	45259002	46433733	399	6.97E-06	1.55E-09	0.859
19	46433891	47333204	330	1.47E-05	1.42E-09	0.697

19	47333497	48509893	371	-2.44E-05	1.36E-09	0.507
19	49491035	49923166	159	-1.50E-05	5.54E-10	0.525
19	48697961	49485758	337	-1.34E-04	1.88E-09	0.002
19	49925208	50774141	253	-1.67E-07	9.24E-10	0.996
19	50774581	51331589	225	3.03E-05	9.89E-10	0.335
19	52217405	52604625	222	-1.26E-05	7.20E-10	0.638
19	51332061	52216563	534	2.24E-05	6.01E-09	0.772
19	52606936	53107086	222	-2.25E-05	1.03E-09	0.483
19	53107937	53537923	157	6.74E-06	7.79E-10	0.809
19	53538505	53899797	201	-1.71E-05	8.38E-10	0.555
19	54099626	54702450	223	-5.09E-05	1.39E-09	0.173
19	54704266	55174498	212	2.07E-05	1.05E-09	0.522
19	55175009	55791380	260	-1.02E-06	1.15E-09	0.976
19	55794266	56364684	248	-1.82E-06	1.15E-09	0.957
19	56365323	56898342	291	6.75E-05	1.49E-09	0.081
19	57952035	58531715	261	-1.40E-05	7.14E-10	0.601
19	56898853	57950377	491	1.95E-05	1.91E-09	0.656
19	58532756	59118784	238	-1.34E-05	7.52E-10	0.625
20	246392	887800	378	-2.92E-05	1.80E-09	0.491
20	888523	1445821	369	-6.84E-08	1.31E-09	0.998
20	1446580	1897442	256	-8.39E-06	7.26E-10	0.755
20	3083151	3655085	235	6.05E-05	5.15E-10	0.008
20	1899473	3081680	692	5.00E-05	1.90E-09	0.251
20	3655943	4051325	176	-2.02E-05	4.93E-10	0.364
20	4053020	4701211	402	-1.56E-05	1.47E-09	0.684
20	4701706	5304961	360	-6.85E-07	1.07E-09	0.983
20	5305400	6636139	709	-5.18E-05	2.09E-09	0.257
20	6637234	7065163	285	5.22E-05	9.44E-10	0.089
20	8601114	8881586	198	8.08E-07	4.18E-10	0.968
20	7066818	8600105	764	5.82E-06	1.81E-09	0.891
20	8884009	9745450	471	3.90E-06	1.19E-09	0.910
20	11014704	11534388	291	1.70E-05	6.58E-10	0.507
20	11534488	12153352	274	2.20E-05	6.59E-10	0.391
20	9746345	11013570	694	3.21E-05	2.07E-09	0.481
20	13201177	13666198	193	-6.49E-06	5.20E-10	0.776
20	12153396	13199755	620	-9.57E-06	1.62E-09	0.812
20	13670311	14929217	486	8.08E-07	1.15E-09	0.981

20	15148231	16150464	705	1.56E-05	2.02E-09	0.728
20	17449713	17905102	299	-1.32E-05	9.68E-10	0.670
20	17906490	18896415	440	-2.13E-05	9.94E-10	0.499
20	16151566	17449361	878	-6.73E-06	2.27E-09	0.888
20	18896895	19707457	462	-5.08E-05	1.15E-09	0.135
20	19708351	21676556	819	-7.98E-05	2.06E-09	0.079
20	21679930	22779512	406	2.76E-06	7.69E-10	0.921
20	22779865	23568490	399	-6.95E-06	8.81E-10	0.815
20	23568495	24053655	219	1.46E-05	4.86E-10	0.507
20	24054931	25019099	417	-1.64E-05	8.86E-10	0.581
20	25020047	26319418	309	5.96E-07	6.06E-10	0.981
20	26319535	30570889	234	9.23E-06	7.72E-10	0.740
20	30574455	31613985	334	-6.71E-06	6.78E-10	0.797
20	33885155	34891416	221	-3.49E-06	3.85E-10	0.859
20	34892880	35892530	205	1.95E-05	4.53E-10	0.359
20	31615242	33883015	705	-5.76E-05	1.53E-09	0.140
20	35899774	36904375	395	1.75E-06	8.32E-10	0.952
20	36905894	37875045	354	3.97E-06	7.80E-10	0.887
20	37875309	38431408	230	4.13E-06	4.45E-10	0.845
20	38435302	39453533	470	1.09E-05	1.11E-09	0.743
20	39455778	40584825	395	-9.12E-06	9.48E-10	0.767
20	40585126	41194048	393	-2.92E-06	1.07E-09	0.929
20	41195111	42188284	488	-9.14E-06	1.37E-09	0.805
20	42188914	43257406	508	6.24E-06	1.30E-09	0.863
20	44960997	45649369	359	1.47E-05	9.34E-10	0.630
20	43259306	44960236	711	6.02E-05	1.58E-09	0.130
20	45650139	46540102	374	-5.99E-06	1.14E-09	0.859
20	48081983	48655016	227	1.73E-05	7.86E-10	0.538
20	46540587	48080850	770	-8.60E-06	1.83E-09	0.840
20	48658113	49385779	312	-1.57E-05	8.68E-10	0.595
20	49387520	49945761	256	-8.49E-06	1.04E-09	0.793
20	49948016	50633472	360	5.82E-06	1.07E-09	0.858
20	50635347	51389135	319	-8.56E-06	7.29E-10	0.751
20	51389670	51884594	263	-2.01E-05	7.85E-10	0.472
20	51885269	52525657	292	-2.09E-06	1.39E-09	0.955
20	52526840	53406846	485	1.69E-05	1.36E-09	0.647
20	53408035	54321635	409	2.35E-05	9.87E-10	0.454

20	54321811	55431836	547	1.76E-05	1.46E-09	0.646
20	55434683	56214465	499	4.00E-05	1.69E-09	0.330
20	56216001	56679942	290	2.67E-05	9.44E-10	0.386
20	56679960	57319010	352	-5.37E-05	9.99E-10	0.089
20	57320545	58295563	478	-2.79E-05	1.61E-09	0.486
20	59361382	59892000	354	-6.44E-06	1.17E-09	0.850
20	58296350	59361064	553	-3.65E-05	1.45E-09	0.338
20	59892231	61091395	659	-1.87E-05	2.81E-09	0.724
20	61301855	62119318	400	-1.69E-05	1.37E-09	0.648
20	62119875	62962870	341	2.16E-07	1.53E-09	0.996
21	15451950	16139409	423	-2.91E-05	9.25E-10	0.339
21	17305471	18457656	408	-2.33E-05	1.33E-09	0.524
21	18457828	19151566	246	-3.08E-05	8.90E-10	0.302
21	16140244	17301757	441	3.94E-05	1.05E-09	0.223
21	19151940	20198061	542	-2.94E-05	1.13E-09	0.382
21	21022840	21905609	384	7.91E-06	9.44E-10	0.797
21	20199390	21022168	394	-6.31E-06	8.34E-10	0.827
21	24695869	25083563	190	-1.31E-06	4.01E-10	0.948
21	23270321	24695405	540	-3.47E-05	1.11E-09	0.299
21	25090174	25898082	385	-2.22E-05	7.29E-10	0.411
21	25899516	26465774	241	-3.10E-05	5.04E-10	0.167
21	21905658	23268216	670	-2.22E-05	1.36E-09	0.546
21	26467796	27776736	496	-8.15E-07	9.89E-10	0.979
21	27777181	28205789	289	2.43E-05	6.71E-10	0.348
21	28206883	28705495	309	3.16E-05	7.40E-10	0.245
21	28705732	29515396	352	-1.21E-05	8.18E-10	0.672
21	29515884	30844467	550	-1.60E-05	7.94E-10	0.571
21	30845873	32211778	531	1.22E-05	8.43E-10	0.673
21	32213678	33270766	453	1.50E-05	9.95E-10	0.633
21	33273162	33988973	358	-4.85E-07	7.30E-10	0.986
21	33991368	35219895	507	1.59E-07	1.44E-09	0.997
21	35220001	36188046	457	4.63E-05	1.95E-09	0.294
21	36190040	37303320	549	-3.98E-05	1.10E-09	0.230
21	37305159	37868817	300	-6.20E-05	5.54E-10	0.008
21	37870518	38429082	265	-3.62E-06	5.65E-10	0.879
21	38429389	39248065	402	2.04E-05	6.16E-10	0.411
21	39250321	40115483	422	-9.67E-06	9.55E-10	0.754

21	41260363	41705756	344	-3.56E-06	8.02E-10	0.900
21	40116044	41095241	608	-3.66E-05	1.20E-09	0.292
21	41707453	42543663	491	3.89E-05	1.16E-09	0.254
21	44690856	45598522	356	6.65E-06	5.84E-10	0.783
21	42545250	43342046	530	-6.86E-05	1.83E-09	0.109
21	45603905	46174491	279	-3.19E-05	6.86E-10	0.223
21	43343190	44632513	846	-2.84E-05	1.95E-09	0.519
21	46174981	46740019	297	-1.87E-06	5.75E-10	0.938
21	46741287	47491264	334	6.98E-06	6.51E-10	0.784
21	47491975	48119635	293	-3.02E-05	4.90E-10	0.173
22	17187276	17569017	168	-2.39E-05	4.65E-10	0.267
22	18333902	19138442	298	-3.08E-05	7.68E-10	0.266
22	17569439	18333467	393	3.76E-05	1.34E-09	0.304
22	19139368	20000428	424	3.00E-05	1.32E-09	0.410
22	20001006	20952650	273	-5.38E-05	7.91E-10	0.056
22	20952972	21863717	295	-2.29E-06	6.32E-10	0.927
22	21866569	23015302	370	1.14E-05	9.20E-10	0.708
22	23016570	23691564	208	-1.54E-06	4.80E-10	0.944
22	23697301	24370194	307	-2.43E-05	9.52E-10	0.430
22	24375019	25416292	359	-2.11E-05	7.92E-10	0.454
22	25419524	26113333	227	-9.92E-06	6.20E-10	0.690
22	26123092	26735929	447	-4.78E-05	1.30E-09	0.185
22	26736812	27294543	387	2.29E-05	1.16E-09	0.502
22	27295234	27653424	353	-6.46E-06	1.24E-09	0.854
22	29456360	30604102	391	-4.50E-05	7.77E-10	0.107
22	27654838	29454477	731	-4.81E-05	2.13E-09	0.297
22	30605601	31534127	380	-8.70E-07	9.29E-10	0.977
22	31535872	32324140	238	1.60E-06	4.05E-10	0.936
22	32324692	32856999	277	-1.66E-05	6.68E-10	0.521
22	33512106	33896495	178	1.16E-05	5.65E-10	0.625
22	32857285	33511391	411	-3.24E-06	1.16E-09	0.924
22	35865218	36131498	134	-1.43E-05	4.35E-10	0.492
22	34983275	35862655	468	-4.17E-06	8.90E-10	0.889
22	33898114	34982457	617	-3.60E-06	1.44E-09	0.924
22	36132263	36922822	314	2.33E-05	8.28E-10	0.418
22	36923144	37558356	398	4.44E-05	1.18E-09	0.196
22	38473398	39302783	269	1.97E-05	7.39E-10	0.468

22	37560592	38467784	418	-1.59E-05	1.01E-09	0.617
22	39304509	40092866	321	2.57E-05	8.10E-10	0.367
22	40095174	41405753	318	-2.08E-05	6.91E-10	0.430
22	43187900	43645335	232	-6.13E-05	3.29E-09	0.286
22	41406919	42870441	427	-2.42E-05	1.02E-09	0.448
22	43646241	44317084	380	2.30E-06	8.88E-10	0.938
22	44317416	44818986	414	5.27E-05	1.35E-09	0.151
22	45444397	45895495	268	2.61E-06	7.05E-10	0.922
22	44820796	45443845	418	-1.28E-05	1.48E-09	0.739
22	45895856	46512413	262	4.99E-06	8.48E-10	0.864
22	46513376	47106715	271	-2.44E-05	8.07E-10	0.391
22	47937891	48479731	373	-2.80E-05	1.06E-09	0.391
22	47106800	47937655	434	-9.92E-07	1.07E-09	0.976
22	49082979	49521080	313	-2.02E-05	1.20E-09	0.559
22	49522184	49751418	246	-1.41E-06	9.02E-10	0.963
22	48481280	49081753	464	9.75E-06	1.61E-09	0.808
22	49751512	50488153	335	8.78E-06	8.88E-10	0.768
22	50488995	51237713	281	-1.18E-05	8.23E-10	0.680
**Prostate cancer and COVID-19 hospitalization**						
1	1103150	2320702	359	-6.92E-05	3.07E-09	0.212
1	2321099	3065568	234	-7.18E-05	2.33E-09	0.137
1	3066761	3679461	290	-1.43E-04	5.55E-09	0.054
1	3679775	4749076	632	-1.19E-04	6.48E-09	0.138
1	4750013	5000086	168	-1.07E-05	1.75E-09	0.797
1	5000970	5471517	301	6.92E-05	3.10E-09	0.214
1	5472626	6133700	338	3.43E-05	3.18E-09	0.543
1	6134990	7245669	447	1.00E-05	3.52E-09	0.866
1	7245922	7604778	205	-2.48E-05	1.94E-09	0.574
1	7604831	9067142	513	9.96E-06	4.18E-09	0.878
1	9067673	9959051	319	8.01E-05	5.53E-09	0.281
1	9959748	11774956	584	7.70E-05	4.45E-09	0.248
1	11776196	12147753	192	-4.90E-05	1.43E-09	0.196
1	12150385	14625524	729	1.17E-04	7.57E-09	0.179
1	14626896	15423481	421	-7.29E-05	4.76E-09	0.291
1	15424441	17123862	613	-5.11E-05	4.21E-09	0.431
1	17176635	17872659	334	-2.13E-05	2.49E-09	0.670
1	17874843	18484945	341	1.20E-06	3.27E-09	0.983

1	18486272	20022063	799	-5.64E-05	7.59E-09	0.517
1	20023833	20763899	370	1.46E-05	4.32E-09	0.825
1	20765245	22634131	796	-6.52E-05	6.90E-09	0.432
1	22636103	23082667	208	-4.65E-05	2.07E-09	0.307
1	23085227	24527501	491	-6.78E-05	3.61E-09	0.259
1	24528210	25305673	317	-2.66E-05	3.14E-09	0.636
1	25305783	26976424	610	-1.33E-05	4.40E-09	0.841
1	26981479	28160199	205	6.20E-06	1.76E-09	0.883
1	28168872	29181525	242	-4.06E-05	1.50E-09	0.294
1	29184254	30792409	735	-4.37E-05	5.85E-09	0.568
1	30792984	32042421	610	9.69E-05	3.90E-09	0.121
1	32042540	34016219	486	4.72E-05	3.09E-09	0.396
1	34017349	34797348	442	-1.83E-05	3.86E-09	0.768
1	34798014	35364965	274	-1.93E-05	2.90E-09	0.720
1	35366056	38196841	762	6.42E-05	6.51E-09	0.426
1	38459210	39535259	436	6.92E-06	4.49E-09	0.918
1	39537291	40933221	445	2.18E-04	3.31E-09	0.000
1	40934284	42166782	487	-9.45E-05	4.33E-09	0.151
1	42168539	43365094	396	-5.62E-05	2.52E-09	0.263
1	43365565	44482312	463	-9.93E-05	2.94E-09	0.067
1	44483704	46895641	764	2.84E-05	4.07E-09	0.657
1	46897698	47999344	386	-1.41E-05	2.41E-09	0.774
1	48000156	50640662	790	2.95E-05	5.14E-09	0.681
1	50641944	53116453	445	1.85E-05	2.82E-09	0.727
1	53117066	54197141	419	-8.46E-05	3.07E-09	0.127
1	54197688	55012825	295	-2.78E-05	2.80E-09	0.600
1	55012881	55479198	250	-1.75E-05	2.43E-09	0.722
1	55479967	56413577	485	9.16E-05	3.88E-09	0.141
1	56414730	57585670	599	-1.05E-05	4.63E-09	0.878
1	57585980	58535140	555	-4.73E-05	3.85E-09	0.446
1	58536417	59306823	412	-9.34E-06	3.00E-09	0.865
1	59307290	59736866	187	8.78E-06	1.74E-09	0.833
1	59739179	61257170	537	-8.11E-05	4.13E-09	0.207
1	61258378	62107806	382	8.21E-05	4.71E-09	0.232
1	62108681	63376404	518	7.87E-05	3.98E-09	0.212
1	63379031	65008206	795	1.94E-05	6.58E-09	0.811
1	65010679	66773349	699	-2.78E-05	5.64E-09	0.711

1	66774350	67760291	537	1.54E-05	3.69E-09	0.799
1	67761365	69441414	724	-1.27E-04	6.08E-09	0.105
1	69441920	70486660	328	5.32E-05	2.61E-09	0.298
1	70490925	71818029	502	1.06E-05	3.79E-09	0.863
1	71822765	74326378	643	2.40E-05	3.95E-09	0.703
1	74326484	75992462	563	-7.85E-05	3.67E-09	0.195
1	75992778	76476360	183	-8.59E-06	1.07E-09	0.792
1	76477207	77139859	372	-2.98E-05	3.29E-09	0.603
1	77141039	79111862	686	6.51E-05	5.20E-09	0.367
1	79113455	80341320	483	-1.22E-06	3.38E-09	0.983
1	80341732	81056946	241	-1.03E-05	1.78E-09	0.807
1	81058295	82883310	839	-1.02E-04	7.21E-09	0.232
1	82884721	83990906	441	-1.82E-05	3.24E-09	0.749
1	83991748	84728665	328	-5.35E-07	2.19E-09	0.991
1	84729134	85940249	595	-3.38E-05	5.01E-09	0.633
1	85940875	86549498	270	-3.07E-07	1.86E-09	0.994
1	86549569	87652502	417	-7.69E-06	3.15E-09	0.891
1	87654606	89447978	634	-6.89E-05	5.94E-09	0.371
1	89451664	91866856	883	4.10E-06	6.73E-09	0.960
1	91867046	93351477	560	6.32E-06	3.83E-09	0.919
1	93352491	94451007	318	-2.04E-05	1.87E-09	0.637
1	94452062	95199048	406	1.57E-05	3.25E-09	0.783
1	95199771	96149941	435	3.48E-06	3.57E-09	0.954
1	96150318	97457893	467	-3.77E-05	3.09E-09	0.498
1	97458722	98562260	517	-4.77E-05	2.96E-09	0.380
1	98564736	100064599	575	8.42E-05	4.65E-09	0.217
1	100065413	101682573	614	1.05E-04	3.73E-09	0.086
1	101683165	102039858	221	-3.94E-05	1.85E-09	0.360
1	102040268	102893372	341	-5.45E-05	2.63E-09	0.288
1	102894567	104369301	288	1.63E-05	2.18E-09	0.728
1	104373157	105192916	270	-1.25E-05	2.18E-09	0.789
1	105193022	106641877	398	2.31E-05	2.91E-09	0.668
1	106642705	107432265	291	-4.86E-06	2.12E-09	0.916
1	107432576	109075191	691	5.82E-05	6.13E-09	0.458
1	109075561	110301260	464	4.58E-05	3.43E-09	0.435
1	110302505	111738116	680	-1.06E-04	5.83E-09	0.164
1	111739749	112560278	566	-8.28E-05	4.98E-09	0.241

1	112561693	114451307	681	-1.79E-05	4.16E-09	0.781
1	114451386	115878242	636	4.73E-06	4.91E-09	0.946
1	115879821	116884431	399	4.04E-05	3.14E-09	0.471
1	116884519	118837474	650	-4.51E-06	5.14E-09	0.950
1	118837888	119792751	365	-3.71E-05	2.50E-09	0.458
1	119793605	120347239	234	-4.97E-07	2.09E-09	0.991
1	144998563	147347713	454	4.39E-05	3.21E-09	0.439
1	147348339	151830844	599	-1.14E-05	8.25E-09	0.900
1	151832901	152535021	216	7.03E-05	1.19E-09	0.041
1	152535586	153163377	278	1.38E-05	1.45E-09	0.718
1	153165450	154544651	506	8.71E-06	2.96E-09	0.873
1	154547292	156334116	551	-3.50E-05	3.41E-08	0.850
1	156335111	157154186	313	6.61E-05	4.63E-09	0.332
1	157154324	158867538	820	8.46E-05	6.51E-09	0.295
1	158870085	160648549	764	4.47E-06	7.01E-09	0.957
1	160649926	161973976	510	8.19E-05	3.96E-09	0.193
1	161974584	162995355	563	-3.45E-05	4.50E-09	0.607
1	163000394	164410254	676	-8.06E-05	4.59E-09	0.234
1	164412847	165190876	436	-3.85E-05	4.49E-09	0.566
1	165191702	166363258	605	3.85E-05	5.17E-09	0.592
1	166364063	167158927	484	2.25E-05	3.45E-09	0.702
1	167159608	168168773	462	3.42E-05	4.06E-09	0.592
1	168169452	169077127	489	-2.13E-05	4.58E-09	0.753
1	169078572	169521853	193	-2.03E-05	1.12E-09	0.543
1	169522554	170372503	446	-4.14E-05	2.96E-09	0.446
1	170375663	170914261	181	-1.64E-05	1.40E-09	0.660
1	170915133	172647523	864	6.51E-05	5.55E-09	0.382
1	172648956	175384090	909	8.88E-07	5.41E-09	0.990
1	175385076	176416712	369	3.67E-05	2.58E-09	0.469
1	176418578	177431255	438	-1.91E-05	3.04E-09	0.729
1	177432237	178940717	621	2.05E-05	4.43E-09	0.758
1	178943012	179676274	338	-1.42E-05	2.04E-09	0.754
1	179678188	180567596	397	-6.02E-05	2.90E-09	0.263
1	180568720	181311800	309	5.29E-07	2.13E-09	0.991
1	181313404	182293001	421	-9.37E-06	3.28E-09	0.870
1	182294372	183796074	577	1.11E-06	3.64E-09	0.985
1	183796618	185511423	738	-7.27E-05	4.92E-09	0.300

1	185512838	187847742	807	-7.03E-06	5.43E-09	0.924
1	187848898	189756187	599	3.42E-05	3.38E-09	0.556
1	189758772	190107648	158	-1.53E-07	1.00E-09	0.996
1	190107883	191432168	473	1.26E-05	2.86E-09	0.814
1	191433917	192444256	331	-2.13E-05	2.12E-09	0.645
1	192452631	193504005	346	1.46E-05	2.11E-09	0.750
1	193505535	194000243	197	1.02E-06	1.26E-09	0.977
1	194000953	194733444	212	-2.55E-05	1.32E-09	0.482
1	194734415	195155161	176	-1.28E-05	1.08E-09	0.696
1	195155293	195995510	312	-1.56E-05	2.33E-09	0.746
1	195995948	197309603	344	3.85E-05	2.22E-09	0.414
1	197310096	198540028	417	-6.34E-05	2.64E-09	0.217
1	198540418	199578700	369	-3.70E-06	2.76E-09	0.944
1	199580285	201070422	658	-9.63E-05	4.98E-09	0.173
1	201070740	201365889	257	7.11E-06	2.19E-09	0.879
1	201366509	202820077	608	-4.42E-05	5.20E-09	0.540
1	202820815	203979519	488	5.40E-05	4.35E-09	0.413
1	203980019	205458538	791	-2.94E-04	1.33E-08	0.011
**1**	**205459511**	**206705609**	**336**	**5.00E-04**	**8.93E-09**	**0.000**
1	206706419	208158787	548	2.06E-05	4.06E-09	0.746
1	210741816	211763053	432	-8.13E-06	3.94E-09	0.897
1	208160220	210738604	1288	2.03E-05	1.07E-08	0.844
1	211763754	213578006	641	9.55E-05	5.24E-09	0.187
1	213578592	215128675	756	4.90E-05	6.40E-09	0.540
1	215129087	216672805	623	-1.35E-05	4.55E-09	0.842
1	216673326	217575665	684	-1.51E-07	5.94E-09	0.998
1	217577346	218860068	520	-1.89E-06	4.65E-09	0.978
1	218860456	220068681	530	-2.57E-05	3.63E-09	0.669
1	220069681	221856899	640	5.79E-05	4.71E-09	0.399
1	221857359	223630391	645	-1.55E-04	6.76E-09	0.060
1	223630619	224434769	280	7.51E-07	2.22E-09	0.987
1	224435934	226143348	570	-4.88E-05	4.10E-09	0.446
1	226146348	226808294	242	5.17E-06	1.96E-09	0.907
1	226808697	228100891	558	6.80E-05	3.80E-09	0.270
1	228101119	229075210	320	-2.31E-04	4.79E-09	0.001
1	229076157	230477970	634	2.49E-06	5.06E-09	0.972
1	230479324	231318678	503	2.88E-05	4.67E-09	0.673

1	231321215	232382234	476	-1.77E-05	3.60E-09	0.768
1	232384356	232949914	349	-2.00E-05	3.07E-09	0.718
1	232950888	233750725	429	1.77E-05	3.82E-09	0.775
1	233753991	234206593	224	9.95E-06	2.13E-09	0.829
1	234209087	235772624	702	2.44E-05	6.68E-09	0.765
1	235773790	236536723	313	2.71E-05	2.54E-09	0.591
1	236537770	237493854	565	-8.41E-06	4.47E-09	0.900
1	237495080	239297872	909	-2.55E-05	8.26E-09	0.779
1	239299272	240562764	672	-5.88E-06	6.16E-09	0.940
1	240563367	241178287	352	6.36E-06	4.26E-09	0.922
1	241178944	241766551	357	-4.54E-05	3.74E-09	0.458
1	241768617	242697031	497	4.26E-06	5.10E-09	0.952
1	242700803	244105053	454	6.01E-05	2.95E-09	0.269
1	244106113	244927751	382	-1.71E-05	3.94E-09	0.785
1	246606926	246989171	161	-4.09E-06	1.39E-09	0.912
1	244929262	246606531	851	1.46E-04	7.80E-09	0.098
1	246991094	247838628	369	-4.54E-05	3.38E-09	0.435
1	247838870	248585259	281	-1.16E-05	1.96E-09	0.793
2	11320	1158981	538	-5.54E-05	4.42E-09	0.405
2	1159982	1681720	220	1.11E-05	2.19E-09	0.812
2	1683187	3003071	698	5.32E-05	7.40E-09	0.536
2	3003472	4148069	589	6.18E-06	5.66E-09	0.935
2	4148784	5017172	423	-1.27E-05	3.95E-09	0.840
2	5118816	6361343	633	-4.34E-05	7.14E-09	0.607
2	6363082	7322437	569	-5.49E-05	5.57E-09	0.462
2	7323039	8980346	702	-8.99E-05	6.94E-09	0.281
2	8980472	10440987	657	-1.26E-04	7.48E-09	0.146
2	10442541	10879340	236	1.38E-04	4.44E-09	0.038
2	10881867	11652586	377	1.25E-04	6.69E-09	0.126
2	11652795	12720977	565	5.94E-06	6.64E-09	0.942
2	12723749	14648908	706	5.72E-05	5.97E-09	0.459
2	14649571	15720775	452	-1.94E-05	3.20E-09	0.732
2	15721423	16549199	386	-7.06E-05	4.57E-09	0.296
2	16549538	18344242	767	1.48E-04	6.32E-09	0.063
2	18344821	18646822	144	2.08E-06	1.23E-09	0.953
2	18647868	20059542	691	2.32E-05	6.05E-09	0.765
2	20062043	22427190	825	2.47E-05	1.13E-08	0.816

2	22428680	23855977	652	-3.32E-05	5.81E-09	0.663
2	23857184	25485735	455	1.29E-04	2.99E-09	0.018
2	25486770	26894217	438	1.04E-05	3.57E-09	0.862
2	26894589	28597305	482	1.67E-05	2.86E-09	0.755
2	28597624	29484922	455	1.48E-04	3.88E-09	0.018
2	29486078	29987735	338	-2.10E-05	2.83E-09	0.692
2	29988753	31009977	499	1.37E-05	4.49E-09	0.838
2	31010212	32489851	591	-1.75E-05	4.48E-09	0.794
2	32493210	33361425	336	1.49E-06	2.93E-09	0.978
2	33362643	34279720	532	-3.18E-05	4.88E-09	0.649
2	34280335	35607183	529	1.11E-06	4.23E-09	0.986
2	35608923	36121342	278	7.85E-05	2.12E-09	0.088
2	36122006	37004185	553	8.22E-06	5.40E-09	0.911
2	37008066	38882278	873	-1.18E-04	1.02E-08	0.244
2	38882577	40077971	314	-2.00E-05	1.99E-09	0.654
2	40080343	40625847	349	-6.91E-05	2.90E-09	0.199
2	40626336	41381901	294	-2.14E-05	2.16E-09	0.645
2	41382889	42087411	359	-3.50E-05	2.72E-09	0.501
2	42088016	42947335	404	2.77E-05	3.11E-09	0.619
2	42949616	43307776	218	6.86E-05	9.84E-09	0.489
2	43309247	44048346	326	-1.95E-04	8.90E-09	0.039
2	44049120	45194289	539	-1.66E-05	4.20E-09	0.798
2	45195022	45562814	268	-4.29E-05	3.44E-09	0.464
2	45563613	47052067	881	-2.48E-05	8.86E-09	0.792
2	47052788	47782756	370	-1.18E-04	4.25E-09	0.071
2	47782836	49112501	508	2.78E-05	4.82E-09	0.689
2	49113132	50757671	842	3.35E-05	6.82E-09	0.685
2	50758657	51667413	400	-4.91E-06	3.17E-09	0.930
2	51668142	53351493	835	6.41E-06	6.68E-09	0.937
2	53352175	54599930	554	-2.12E-05	4.62E-09	0.755
2	54600556	56158646	724	-2.98E-05	6.00E-09	0.700
2	56160647	56599354	240	-1.75E-05	2.66E-09	0.734
2	56600739	57938354	492	-3.90E-05	3.76E-09	0.524
2	57939866	60449069	1014	5.35E-05	8.72E-09	0.567
2	60449710	62420656	658	9.01E-05	4.76E-09	0.191
2	62425639	64623935	687	-1.80E-04	1.43E-08	0.132
2	64626895	65226463	256	-1.95E-05	3.52E-09	0.743

2	65228236	66374803	559	4.70E-05	4.66E-09	0.491
2	66375016	67978287	798	-8.25E-05	7.10E-09	0.327
2	67981487	68871573	395	-2.72E-05	3.13E-09	0.627
2	68871710	70753882	813	-7.15E-05	6.02E-09	0.357
2	70756057	71704491	467	-9.14E-06	4.35E-09	0.890
2	71704844	73172972	470	1.81E-05	3.89E-09	0.772
2	73174848	74121000	254	2.47E-06	1.51E-09	0.949
2	76616819	77384991	368	2.13E-05	3.13E-09	0.704
2	74121278	76615798	1069	-1.14E-05	8.98E-09	0.904
2	77386522	79007966	562	9.59E-07	4.97E-09	0.989
2	80804991	81588311	231	-2.46E-05	1.64E-09	0.544
2	79008155	80804284	1066	1.69E-05	9.79E-09	0.864
2	81588482	83604057	600	-1.72E-05	3.42E-09	0.769
2	83604796	85283713	481	2.41E-05	3.20E-09	0.671
2	85284353	86559358	572	1.40E-04	6.89E-09	0.093
2	86562902	89135764	461	-2.83E-05	3.51E-09	0.633
2	92324281	98790763	461	1.17E-05	2.93E-09	0.828
2	98791433	100195879	407	-5.62E-05	2.45E-09	0.257
2	100197638	101338509	453	3.46E-05	3.07E-09	0.532
2	101338644	102688272	529	8.42E-05	4.64E-09	0.216
2	102688495	103262415	357	2.21E-05	2.37E-09	0.649
2	103264434	104481488	294	-4.61E-06	2.25E-09	0.922
2	104485456	105871610	462	-2.16E-05	4.01E-09	0.733
2	105872982	107406122	724	-3.82E-05	7.03E-09	0.648
2	107406885	108427822	407	2.86E-05	3.14E-09	0.610
2	108427959	110109210	583	-5.92E-07	4.46E-09	0.993
2	110109222	112924907	586	1.14E-04	6.70E-09	0.164
2	112924973	114846282	718	-3.50E-05	5.48E-09	0.637
2	114848115	116770829	607	-1.65E-06	4.77E-09	0.981
2	116772945	117727045	304	-1.96E-06	1.92E-09	0.964
2	117728904	119581280	716	1.12E-05	6.02E-09	0.885
2	119581439	121070811	505	-8.39E-05	3.98E-09	0.184
2	121073253	123241656	763	1.50E-05	8.16E-09	0.868
2	123244233	124730999	532	-4.92E-06	3.82E-09	0.937
2	124733246	125735879	428	7.37E-05	3.65E-09	0.222
2	125741434	127372889	519	-1.07E-05	3.57E-09	0.858
2	127374341	129310292	757	-1.43E-05	6.43E-09	0.858

2	129312188	129864416	215	-1.67E-05	1.96E-09	0.706
2	129864730	131463038	407	2.38E-06	3.20E-09	0.966
2	131466136	133030349	226	-8.61E-06	2.04E-09	0.849
2	133034031	133950661	431	2.72E-05	4.04E-09	0.669
2	133951544	136822267	1074	-5.64E-05	7.82E-09	0.523
2	136825272	138619715	738	3.00E-05	5.87E-09	0.696
2	138620371	139412389	267	3.22E-06	1.92E-09	0.941
2	139413312	140902261	570	3.55E-05	4.65E-09	0.602
2	140902383	141803496	400	-3.54E-05	3.28E-09	0.536
2	141803602	143254981	699	8.94E-05	6.06E-09	0.251
2	143255262	145301314	618	-5.23E-05	5.44E-09	0.478
2	145303576	147252762	524	-2.57E-05	4.02E-09	0.686
2	147255216	149268931	497	-3.68E-06	3.47E-09	0.950
2	149270710	151184680	609	-8.81E-05	5.03E-09	0.214
2	151191481	152490219	540	-6.13E-05	4.30E-09	0.350
2	152491269	153369892	311	-1.28E-05	2.11E-09	0.780
2	153371042	154414456	449	1.00E-05	4.18E-09	0.876
2	154415112	155208173	297	2.79E-06	2.49E-09	0.955
2	155209736	156568446	482	1.29E-06	3.67E-09	0.983
2	156568806	158165818	441	5.93E-05	3.92E-09	0.344
2	158173710	159574883	463	-5.00E-06	4.14E-09	0.938
2	159575554	161077823	603	-3.65E-05	4.52E-09	0.587
2	161078891	163358537	678	2.16E-05	4.34E-09	0.742
2	163359084	165200324	484	-4.89E-05	4.14E-09	0.448
2	165202539	167120956	737	1.63E-04	6.54E-09	0.044
2	167123247	168427789	400	3.92E-06	2.91E-09	0.942
2	168431583	169176766	322	-7.12E-05	3.71E-09	0.242
2	170973461	172086127	479	-3.51E-05	4.44E-09	0.598
2	169178092	170973262	789	-6.10E-05	6.45E-09	0.447
2	172088943	173586201	589	1.05E-04	2.00E-08	0.458
2	173588663	175585616	784	4.87E-05	7.41E-09	0.572
2	175586510	177326682	629	3.78E-05	4.60E-09	0.577
2	177327202	178551958	558	2.27E-05	4.22E-09	0.727
2	178552788	180138313	565	-1.45E-04	5.85E-09	0.059
2	180139219	180661233	324	-7.29E-06	3.55E-09	0.903
2	180662102	181627195	413	2.91E-05	3.41E-09	0.618
2	181628139	182840114	361	-2.06E-05	2.76E-09	0.696

2	182845657	184442530	601	8.51E-05	4.50E-09	0.205
2	184443166	185277576	257	1.71E-05	1.79E-09	0.685
2	185279087	186731282	380	-2.16E-05	2.49E-09	0.666
2	186731309	188540877	478	-2.00E-05	3.02E-09	0.716
2	188542361	190794689	527	1.06E-05	3.81E-09	0.863
2	190797341	191870624	349	-2.48E-05	2.45E-09	0.616
2	191873553	192695107	283	-2.63E-05	2.83E-09	0.621
2	192696255	195109845	443	-3.52E-06	2.93E-09	0.948
2	195117932	197307918	619	2.55E-05	4.69E-09	0.710
2	197308707	198140953	269	-4.38E-05	1.76E-09	0.296
2	198141746	200498970	620	4.82E-05	3.82E-09	0.435
2	200504209	201571624	452	5.52E-05	3.34E-09	0.340
2	201572564	202829668	381	-1.12E-05	3.09E-09	0.840
2	202830132	204814896	524	-5.65E-06	3.91E-09	0.928
2	204815902	206043539	517	-1.18E-05	4.84E-09	0.865
2	206045927	207721890	728	-3.92E-05	6.24E-09	0.620
2	207722425	208639154	389	-3.18E-05	4.54E-09	0.637
2	208641174	209940909	408	-2.42E-05	3.27E-09	0.673
2	209941332	210832981	269	1.31E-06	1.84E-09	0.976
2	210838781	212377672	605	4.29E-05	4.86E-09	0.538
2	212377759	212845716	216	3.63E-05	2.29E-09	0.449
2	212845994	215005050	818	-7.57E-05	6.27E-09	0.339
2	215006557	216448007	604	-3.44E-05	4.97E-09	0.626
2	216449570	217184275	343	-4.91E-06	3.33E-09	0.932
2	217184715	218615634	620	-2.00E-05	5.49E-09	0.787
2	218616633	219671910	401	-2.10E-05	2.64E-09	0.683
2	219675067	220774229	409	4.65E-06	3.09E-09	0.933
2	220775971	221445218	359	-5.01E-05	2.91E-09	0.353
2	221445953	222985126	638	3.96E-05	5.81E-09	0.603
2	222985611	224002780	434	-5.12E-06	3.86E-09	0.934
2	224003069	225832409	736	1.45E-05	5.98E-09	0.851
2	225833758	227531180	618	-2.99E-05	4.52E-09	0.657
2	227532464	228456451	381	2.12E-05	2.95E-09	0.696
2	228457117	229368530	434	8.97E-06	3.96E-09	0.887
2	229368812	230282624	396	-2.92E-05	3.54E-09	0.624
2	230283310	231322125	471	5.94E-05	4.24E-09	0.362
2	231322831	232790491	543	1.12E-05	4.99E-09	0.874

2	232791948	233964632	468	-1.85E-05	3.83E-09	0.765
2	233968153	234699046	368	-1.97E-05	2.39E-09	0.687
2	234699366	235626316	630	2.80E-05	6.48E-09	0.728
2	235627087	236619385	515	-1.81E-05	6.09E-09	0.817
2	236619713	238209382	607	2.45E-05	5.44E-09	0.740
2	238209871	239122917	445	-1.37E-04	6.34E-09	0.086
2	239123082	239801883	289	2.71E-06	2.52E-09	0.957
2	239832097	240325991	242	2.23E-05	3.37E-09	0.701
2	240327154	240961852	336	-8.38E-05	3.80E-09	0.174
2	240962194	241907076	461	-5.68E-05	4.66E-09	0.405
2	241909927	243185680	317	6.59E-06	6.45E-09	0.935
3	60197	1128944	484	1.40E-05	5.95E-09	0.856
3	1129028	1527748	339	5.10E-05	4.48E-09	0.446
3	1528147	2423237	600	-5.04E-05	6.69E-09	0.538
3	2423999	2769230	241	-1.37E-05	3.29E-09	0.811
3	2769392	3364279	476	4.90E-05	7.08E-09	0.560
3	3364442	4430024	630	9.16E-06	6.72E-09	0.911
3	4430848	5564422	653	3.63E-05	8.57E-09	0.695
3	5565384	6421659	513	4.26E-05	6.02E-09	0.583
3	6422814	7509288	575	7.17E-05	6.11E-09	0.359
3	7509715	7991133	371	-1.09E-05	3.82E-09	0.860
3	7991981	9194649	780	-6.20E-05	8.85E-09	0.510
3	9195331	10526438	649	-2.87E-05	7.02E-09	0.732
3	10526803	11220278	388	8.36E-05	5.17E-09	0.245
3	11221721	12858028	674	1.64E-05	6.65E-09	0.841
3	12859004	13523338	333	-3.26E-05	3.97E-09	0.605
3	13524741	14309204	367	2.70E-06	4.30E-09	0.967
3	14309548	14816364	329	4.19E-05	4.16E-09	0.516
3	14816745	16661587	815	-7.56E-05	8.26E-09	0.405
3	16662488	18454062	541	-5.72E-05	5.18E-09	0.426
3	18457304	18886128	166	9.35E-06	1.57E-09	0.813
3	18886158	20752492	602	8.04E-05	6.29E-09	0.311
3	20753368	21522703	363	3.53E-05	4.39E-09	0.594
3	21523116	22102707	449	4.13E-05	5.19E-09	0.567
3	22103146	23122359	464	-3.26E-05	4.87E-09	0.641
3	23123318	23802647	259	4.58E-05	3.40E-09	0.432
3	23803541	25459497	887	-2.32E-05	9.69E-09	0.814

3	25460109	26392563	317	3.64E-06	2.88E-09	0.946
3	26393305	27606695	467	-9.00E-05	4.25E-09	0.168
3	27609447	29409547	794	-7.95E-05	9.01E-09	0.402
3	29409589	30716602	620	2.33E-05	7.16E-09	0.783
3	30717661	31720982	438	3.67E-05	3.89E-09	0.557
3	31721774	33011510	532	-4.98E-06	6.81E-09	0.952
3	33012981	33986974	341	4.10E-05	3.12E-09	0.463
3	33988115	35241960	454	2.58E-05	4.38E-09	0.697
3	35243764	36481385	482	-4.20E-05	4.49E-09	0.531
3	36484375	38078255	627	5.40E-05	5.96E-09	0.485
3	38080101	38723967	263	-1.50E-05	2.54E-09	0.766
3	38724849	40649760	701	-2.88E-05	6.21E-09	0.714
3	40660283	42184173	434	2.55E-05	3.82E-09	0.681
3	42184361	45162316	1057	3.26E-07	9.71E-09	0.997
3	46657428	48525955	410	4.60E-05	3.34E-09	0.426
3	45165430	46657162	653	6.95E-05	2.94E-08	0.685
3	48531227	52214640	855	5.13E-05	6.97E-09	0.539
3	52215002	54078568	705	3.24E-05	6.02E-09	0.676
3	54080241	55731145	920	1.62E-06	1.05E-08	0.987
3	55733390	58063819	873	-3.77E-05	7.69E-09	0.667
3	58067795	58700675	307	3.88E-05	2.79E-09	0.462
3	58700802	60347135	936	-8.95E-06	1.11E-08	0.932
3	60347701	61277928	614	2.25E-05	6.55E-09	0.781
3	61279726	62881664	855	-5.87E-06	1.04E-08	0.954
3	62882619	63504979	310	5.20E-06	3.18E-09	0.927
3	63505191	64473972	483	-3.09E-05	5.45E-09	0.675
3	64475164	65337581	550	3.41E-05	6.36E-09	0.669
3	65340365	66169323	368	-4.91E-05	4.62E-09	0.470
3	66170253	67294968	419	-1.67E-05	4.40E-09	0.801
3	67299401	69197507	757	-8.03E-05	7.38E-09	0.350
3	69205190	70288126	413	1.32E-05	4.24E-09	0.840
3	70289919	71418327	409	-2.59E-05	9.19E-09	0.787
3	71421152	73238935	813	-4.55E-05	1.01E-08	0.652
3	73239448	75376291	877	-3.65E-05	9.32E-09	0.705
3	75377075	76854466	341	1.93E-05	3.87E-09	0.756
3	76855676	77507524	288	-2.27E-05	3.03E-09	0.680
3	77508835	79022673	488	4.90E-05	4.83E-09	0.481

3	79024541	81187867	525	-1.59E-05	4.35E-09	0.809
3	81190039	82446513	356	-6.33E-06	2.79E-09	0.905
3	82446746	83946780	383	-2.03E-05	3.33E-09	0.725
3	83947451	85090935	310	2.80E-05	2.72E-09	0.592
3	85093629	86734415	435	1.16E-04	4.91E-09	0.098
3	86736368	87407921	210	-4.68E-05	7.34E-09	0.585
3	87408634	88725583	376	-1.37E-04	3.83E-09	0.027
3	88725754	94202010	445	6.98E-05	3.92E-09	0.264
3	94202771	96056259	472	-3.28E-05	3.70E-09	0.590
3	96679063	98299365	463	-1.51E-05	4.01E-09	0.811
3	98300731	100591006	762	8.94E-06	6.32E-09	0.910
3	100591848	101808006	473	1.62E-04	4.10E-09	0.011
3	101808379	103025517	322	8.94E-06	2.91E-09	0.868
3	103025715	103698105	210	3.00E-05	1.90E-09	0.492
3	103700909	104579408	324	2.40E-05	3.06E-09	0.664
3	104580063	104878270	154	4.91E-05	2.50E-09	0.325
3	104879008	106330349	517	2.49E-05	5.24E-09	0.731
3	106332831	107729547	480	1.41E-04	7.29E-09	0.098
3	107730020	108635884	340	3.85E-06	3.22E-09	0.946
3	108636466	109961316	402	4.79E-05	3.46E-09	0.416
3	109962369	111416310	404	-1.81E-05	3.52E-09	0.761
3	111416451	112182995	420	3.08E-05	3.92E-09	0.623
3	112183360	113323793	432	-8.54E-05	4.93E-09	0.224
3	113324119	115280312	582	4.88E-05	6.03E-09	0.530
3	115282058	116450487	509	-3.12E-05	5.15E-09	0.664
3	116452274	117998840	677	4.44E-05	7.57E-09	0.610
3	118000136	120520317	1012	-9.03E-05	9.42E-09	0.352
3	120521733	121335461	214	-3.14E-06	1.75E-09	0.940
3	121336367	122692039	596	1.14E-04	5.38E-09	0.119
3	122693533	123196819	219	-4.27E-05	2.13E-09	0.355
3	123200440	124677278	597	-5.13E-05	5.74E-09	0.498
3	124678230	125875831	441	-8.76E-05	4.46E-09	0.190
3	125876862	126213162	202	-2.54E-05	1.96E-09	0.566
3	126213467	127471230	536	-1.19E-05	5.27E-09	0.870
3	127471641	130243259	809	-2.33E-04	2.47E-08	0.137
3	130244120	131373405	333	-3.21E-05	2.72E-09	0.538
3	131373740	132115911	443	1.64E-05	8.33E-09	0.857

3	132117285	133128449	407	-2.98E-05	3.65E-09	0.622
3	133129272	134383242	550	1.77E-05	5.97E-09	0.819
3	137370076	138662229	384	6.29E-05	3.32E-09	0.275
3	134385789	137369181	1106	1.16E-04	9.92E-09	0.243
3	138665796	140275652	774	5.37E-05	7.86E-09	0.545
3	140276484	142863564	912	-2.29E-05	8.36E-09	0.803
3	142864473	143494546	387	6.29E-06	5.04E-09	0.929
3	143495679	145663879	729	6.95E-05	6.57E-09	0.391
3	145664135	146722271	402	5.86E-05	3.89E-09	0.347
3	146722383	148316359	475	-3.25E-05	4.88E-09	0.642
3	148316766	149988630	750	1.14E-04	8.04E-09	0.202
3	149994553	151178708	481	1.08E-05	4.99E-09	0.878
3	151178836	151904190	289	-4.14E-06	2.86E-09	0.938
3	151905386	153054901	368	-6.67E-05	3.86E-09	0.283
3	153056839	154181836	468	4.06E-05	4.91E-09	0.563
3	154183562	156005200	508	1.52E-05	5.08E-09	0.831
3	156005905	157311596	529	4.35E-05	5.00E-09	0.539
3	157312028	159272972	717	2.06E-05	6.31E-09	0.796
3	159274785	161523859	701	-4.37E-06	6.10E-09	0.955
3	161524489	162065286	249	7.58E-06	2.84E-09	0.887
3	162067622	163250087	376	1.19E-05	3.13E-09	0.832
3	163251056	164816939	494	-2.62E-05	3.86E-09	0.673
3	164820183	167116849	615	3.55E-05	5.60E-09	0.636
3	167117143	167912878	293	-3.42E-05	2.61E-09	0.503
3	167913564	170158128	714	2.93E-05	1.60E-08	0.817
3	170159134	171311936	486	-2.63E-05	5.28E-09	0.718
3	171312880	172965694	658	-3.63E-05	7.51E-09	0.675
3	172966405	174274308	524	-7.92E-05	5.16E-09	0.270
3	174275336	175049294	365	6.62E-06	4.00E-09	0.917
3	175050262	176268375	461	4.93E-05	4.68E-09	0.471
3	176269091	177530606	501	3.26E-05	5.52E-09	0.661
3	177531847	179359242	627	1.18E-04	6.21E-09	0.135
3	179366819	181426554	525	-6.31E-06	5.03E-09	0.929
3	181431571	182975975	457	5.95E-05	5.18E-09	0.409
3	182976382	183762614	363	-5.95E-05	4.03E-09	0.349
3	183762644	185262137	590	6.20E-05	6.89E-09	0.455
3	185263467	186780913	684	-1.23E-04	8.64E-09	0.185

3	186782999	187220633	227	5.66E-05	2.55E-09	0.262
3	187220777	188201380	508	1.94E-05	6.23E-09	0.806
3	188202550	189531496	585	-8.02E-05	1.01E-08	0.424
3	189531911	190303805	414	1.91E-05	4.67E-09	0.780
3	190304172	190992002	286	2.26E-05	2.88E-09	0.674
3	190993386	191840443	362	-7.34E-06	3.61E-09	0.903
3	191840983	192753870	381	-4.46E-05	3.93E-09	0.477
3	192754177	193885973	515	3.01E-05	5.83E-09	0.693
3	193886289	194464791	286	6.65E-05	3.64E-09	0.270
3	194465233	194756339	146	6.77E-05	2.20E-09	0.149
3	194756597	195854787	322	3.47E-05	3.41E-09	0.552
3	195855401	196414159	209	-5.37E-05	2.58E-09	0.290
3	196415355	197962382	528	2.85E-05	5.05E-09	0.688
4	10642	685993	149	1.66E-06	9.37E-10	0.957
4	686563	1422863	301	-6.02E-05	2.10E-09	0.189
4	1478711	3612028	639	-2.75E-05	4.99E-09	0.697
4	3613164	4263579	147	9.12E-05	2.21E-09	0.052
4	4264705	5044744	440	-3.61E-05	4.11E-09	0.573
4	5047695	5971152	586	-1.94E-05	6.08E-09	0.803
4	5971634	7135912	602	-8.55E-05	7.22E-09	0.314
4	7136158	8054472	690	-1.65E-04	8.19E-09	0.068
4	8055323	9408136	370	1.84E-05	4.77E-09	0.790
4	9408158	10696229	610	4.65E-06	3.65E-09	0.939
4	10696913	12198579	733	5.55E-05	5.62E-09	0.459
4	12201002	13002341	347	7.36E-06	2.26E-09	0.877
4	13002905	14394452	629	-8.47E-05	5.51E-09	0.254
4	14394902	15849986	606	3.65E-05	4.60E-09	0.590
4	15850685	16520118	382	2.38E-05	3.41E-09	0.684
4	16521003	18752293	860	1.60E-05	6.35E-09	0.841
4	18752722	20084049	441	7.11E-05	3.34E-09	0.218
4	20084563	21292213	550	8.44E-05	4.38E-09	0.202
4	21292651	22393873	439	-8.37E-06	3.22E-09	0.883
4	22395570	23563570	369	3.58E-05	2.96E-09	0.511
4	23563931	24992827	684	-6.16E-05	5.96E-09	0.425
4	24993167	26468997	679	7.08E-05	6.79E-09	0.390
4	26473220	27903383	509	-5.74E-06	4.28E-09	0.930
4	27904556	28871862	388	1.32E-05	2.73E-09	0.800

4	28872833	30453156	508	-1.59E-06	3.87E-09	0.980
4	30453619	31739254	482	9.53E-07	3.59E-09	0.987
4	31740009	32592949	294	-9.83E-06	2.12E-09	0.831
4	32593453	33640100	205	-4.73E-05	1.36E-09	0.199
4	33640864	35150284	380	-4.99E-06	1.93E-09	0.910
4	35151237	36301997	434	2.96E-05	3.12E-09	0.596
4	36304377	37534798	577	2.45E-06	4.85E-09	0.972
4	37536119	37886455	212	-1.05E-05	2.21E-09	0.823
4	37887735	38974458	602	3.80E-05	5.88E-09	0.620
4	38976533	40199852	438	-1.37E-05	4.03E-09	0.829
4	40200304	40869208	304	-1.79E-05	3.67E-09	0.768
4	40870402	42662283	725	-8.02E-05	5.79E-09	0.292
4	42662718	43962569	451	-2.52E-06	3.10E-09	0.964
4	43963518	45187835	414	1.61E-05	2.68E-09	0.755
4	45188855	47543891	622	-4.22E-06	4.00E-09	0.947
4	47544012	48226267	277	8.32E-06	1.71E-09	0.840
4	48227642	53412129	408	7.65E-05	1.87E-09	0.077
4	53413674	55202613	631	-3.16E-05	4.20E-09	0.626
4	55202697	57418400	883	1.09E-04	7.05E-09	0.192
4	57420107	58696072	630	1.67E-05	5.53E-09	0.822
4	58697912	59358251	234	4.43E-06	1.77E-09	0.916
4	59358729	60145179	251	-1.18E-05	1.87E-09	0.784
4	60145181	61371992	375	-4.50E-05	2.60E-09	0.377
4	61372415	61872517	167	-7.66E-06	1.21E-09	0.826
4	61872656	64117718	644	-1.62E-05	4.98E-09	0.819
4	64118963	65300308	243	1.17E-05	1.42E-09	0.756
4	65562213	66598615	369	2.77E-05	2.61E-09	0.588
4	66599449	68058793	478	-8.04E-05	3.41E-09	0.169
4	68059850	68455531	160	2.08E-05	1.09E-09	0.529
4	68456074	69954151	269	1.23E-06	1.45E-09	0.974
4	69954297	70517143	270	-5.61E-07	1.49E-09	0.988
4	70517792	71516586	418	1.78E-05	2.55E-09	0.724
4	72416258	75196731	712	1.13E-04	7.79E-09	0.202
4	75555658	76283179	293	4.49E-06	2.46E-09	0.928
4	76284248	77128724	364	1.67E-05	2.17E-09	0.720
4	77129568	78333381	607	-7.26E-05	4.64E-09	0.286
4	78333798	79469226	505	-1.28E-05	3.37E-09	0.825

4	79470550	80994124	436	2.23E-06	3.28E-09	0.969
4	80995881	81978151	261	-2.35E-06	1.75E-09	0.955
4	81979993	83012967	403	-2.06E-06	2.68E-09	0.968
4	83017789	83994716	385	-2.64E-05	2.58E-09	0.603
4	83998014	85080933	430	-1.08E-05	3.35E-09	0.852
4	85081980	87980070	901	1.24E-06	5.56E-09	0.987
4	87980748	89236855	568	9.09E-05	4.67E-09	0.183
4	89238369	90126190	323	-6.15E-05	3.46E-09	0.296
4	90126331	91453993	447	-6.66E-05	3.38E-09	0.252
4	91456722	94229509	751	2.60E-05	5.64E-09	0.729
4	94230511	95596188	496	-2.76E-04	1.91E-08	0.046
4	95596744	96181408	236	7.71E-06	1.81E-09	0.856
4	96182669	96464931	212	-2.15E-05	2.01E-09	0.632
4	96466095	97911191	514	4.54E-06	3.55E-09	0.939
4	97911459	99422081	371	4.96E-06	1.70E-09	0.904
4	99423280	100322786	399	1.35E-05	2.65E-09	0.792
4	100323029	101897750	598	9.42E-06	3.91E-09	0.880
4	101898276	103387944	552	1.91E-06	4.67E-09	0.978
4	103388441	104802530	435	-2.36E-05	2.28E-09	0.620
4	104803329	106479425	508	5.11E-04	4.63E-08	0.017
4	106482185	108468632	628	-1.24E-04	8.06E-09	0.167
4	108469756	109979131	574	-7.69E-05	5.78E-09	0.312
4	109980374	112204254	780	1.06E-04	6.26E-09	0.181
4	112204259	114902479	1024	6.56E-07	7.35E-09	0.994
4	114904730	117275534	678	1.49E-05	4.01E-09	0.814
4	117275542	118219755	308	-3.60E-05	2.23E-09	0.446
4	118220292	119945314	632	4.38E-05	4.38E-09	0.508
4	119946706	121391843	519	-2.05E-05	3.27E-09	0.720
4	121393020	121973864	244	6.59E-05	1.94E-09	0.135
4	121976226	123561869	551	4.28E-05	3.95E-09	0.496
4	123563078	125863762	773	4.64E-05	5.74E-09	0.540
4	125863974	127236805	494	-2.81E-05	4.73E-09	0.683
4	127237363	129514427	625	9.93E-06	3.77E-09	0.871
4	129514448	130364383	286	3.55E-05	2.16E-09	0.446
4	130364741	130949530	194	-2.67E-05	1.28E-09	0.456
4	130950008	131948861	379	7.10E-06	2.95E-09	0.896
4	131951466	133741365	442	-4.97E-05	2.86E-09	0.353

4	133741753	135744132	532	-1.13E-05	3.33E-09	0.845
4	135749591	137247266	391	-1.65E-05	3.14E-09	0.768
4	137247703	137987679	298	1.56E-05	2.70E-09	0.764
4	137988838	139114529	404	-8.92E-06	3.06E-09	0.872
4	139115093	139552965	239	-7.83E-06	2.55E-09	0.877
4	139553359	140546206	342	-7.60E-05	2.63E-09	0.138
4	140548713	141751418	489	-1.50E-06	7.12E-09	0.986
4	141752960	142807957	391	-6.74E-05	3.30E-09	0.241
4	142808503	145021868	627	-6.30E-05	3.38E-09	0.279
4	145024452	148047972	852	6.39E-05	5.88E-09	0.404
4	148053679	150632409	780	5.08E-05	5.30E-09	0.485
4	150634191	153226998	838	2.80E-05	4.98E-09	0.692
4	153233476	154947363	615	-2.78E-06	4.61E-09	0.967
4	154949091	156604003	633	-1.68E-05	5.32E-09	0.818
4	156604173	158112985	581	-6.27E-05	4.79E-09	0.365
4	158114105	160356254	643	5.46E-05	4.23E-09	0.401
4	160357877	160822830	147	-1.04E-05	9.13E-10	0.730
4	161056173	161796696	321	5.66E-06	2.49E-09	0.910
4	162151565	162629423	187	-8.70E-06	2.03E-09	0.847
4	162629512	163181531	216	7.42E-06	1.58E-09	0.852
4	163183341	163726178	186	-5.30E-05	1.96E-09	0.231
4	163728264	164910818	398	5.20E-05	3.57E-09	0.384
4	164913270	165817608	368	3.51E-05	2.95E-09	0.518
4	165820093	166609852	327	-4.35E-05	2.89E-09	0.418
4	166611193	167643023	297	6.44E-06	2.59E-09	0.899
4	169569516	170676328	420	4.17E-06	2.98E-09	0.939
4	167643601	169568082	733	2.79E-05	5.74E-09	0.712
4	170678993	172735897	572	-2.03E-05	3.94E-09	0.746
4	172737462	173760563	310	-3.03E-06	2.09E-09	0.947
4	173761916	174832248	301	5.30E-05	3.17E-09	0.347
4	174833988	176193705	541	-6.03E-06	5.40E-09	0.935
4	176199091	177290105	362	-7.44E-06	3.30E-09	0.897
4	177290945	177972278	282	-2.18E-05	2.21E-09	0.643
4	177974062	178758792	336	-3.00E-05	3.61E-09	0.617
4	178758930	180119509	494	4.94E-05	4.22E-09	0.447
4	180120693	181732608	834	7.33E-05	8.57E-09	0.428
4	181732746	182420390	387	3.44E-05	4.79E-09	0.619

4	182420863	184283167	1001	2.98E-05	1.14E-08	0.780
4	184283411	185477454	593	-6.00E-05	7.34E-09	0.483
4	185477964	185932497	229	-9.31E-06	1.86E-09	0.829
4	185934003	187113041	594	-5.82E-05	6.30E-09	0.464
4	187114073	188687052	716	4.80E-06	7.04E-09	0.954
4	188687155	189524815	363	5.55E-06	4.80E-09	0.936
4	189525752	190088167	270	-2.69E-05	2.74E-09	0.607
4	190088768	191043878	200	-2.03E-05	1.96E-09	0.647
5	10056	1267356	414	4.71E-05	6.83E-09	0.569
5	1270983	1762678	242	9.37E-05	9.29E-09	0.331
5	1763566	2369337	388	1.20E-04	7.27E-09	0.160
5	2369710	2979000	434	5.37E-05	3.80E-09	0.384
5	2979710	3600334	481	-4.83E-05	4.38E-09	0.465
5	3601924	4222111	306	3.39E-05	2.63E-09	0.509
5	4223577	5137474	433	5.44E-05	3.69E-09	0.370
5	5137788	5718773	375	1.19E-05	3.41E-09	0.838
5	5719162	6858229	683	-3.94E-05	5.77E-09	0.604
5	6859297	7852978	495	-5.68E-06	3.96E-09	0.928
5	7853304	9084076	627	6.26E-06	4.45E-09	0.925
5	9084701	10533767	857	-3.50E-05	7.04E-09	0.676
5	10666804	11170506	324	-1.43E-05	2.37E-09	0.769
5	11170703	13603734	759	6.83E-05	4.81E-09	0.324
5	13603883	14987807	739	-1.56E-04	5.08E-09	0.029
5	14988284	16034608	301	-5.61E-05	1.45E-09	0.141
5	16035369	17161451	494	-3.02E-05	4.31E-09	0.646
5	17162704	18048683	259	7.58E-05	2.56E-09	0.134
5	18050107	18791978	252	-4.43E-05	2.04E-09	0.326
5	18792568	19486153	258	1.58E-05	1.77E-09	0.708
5	19487359	20414139	285	3.82E-06	1.46E-09	0.920
5	20415799	21573200	239	6.35E-06	1.67E-09	0.877
5	21574347	23150924	573	-1.36E-05	3.02E-09	0.805
5	23153562	24794957	518	-3.18E-05	3.38E-09	0.585
5	24796795	26183141	453	6.38E-05	3.51E-09	0.282
5	26185302	26849079	203	3.01E-05	1.72E-09	0.469
5	26849808	28303480	392	1.35E-05	2.75E-09	0.796
5	28310093	30129786	583	1.04E-04	3.75E-09	0.088
5	30130713	31748529	717	-5.53E-06	5.83E-09	0.942

5	31748611	33944217	987	5.94E-05	8.72E-09	0.524
5	33945758	35798682	685	1.64E-05	5.13E-09	0.819
5	35799275	37888797	710	-1.95E-05	4.85E-09	0.780
5	37889231	39438373	674	3.60E-05	5.17E-09	0.617
5	39438575	40954879	626	8.27E-05	3.42E-09	0.157
5	40955561	42620153	556	-6.28E-06	2.80E-09	0.906
5	42623245	43859513	307	3.27E-05	1.35E-09	0.373
5	43862567	45286358	281	-4.55E-05	1.42E-09	0.227
5	45286977	50161698	290	-4.54E-06	1.00E-09	0.886
5	50162186	52133670	655	-8.19E-05	4.17E-09	0.205
5	52134410	52661197	301	-2.26E-05	1.97E-09	0.611
5	52662483	53513531	324	4.10E-05	2.59E-09	0.421
5	53515888	55412347	790	1.01E-06	4.75E-09	0.988
5	55413961	55932471	299	-4.34E-05	3.12E-09	0.438
5	55933701	57337744	583	-1.22E-04	5.55E-09	0.101
5	57338357	58834736	805	-3.69E-06	5.66E-09	0.961
5	58834852	60880207	583	1.30E-05	3.07E-09	0.814
5	60883078	62531300	658	9.08E-05	3.80E-09	0.141
5	62531952	63837838	373	3.74E-05	2.20E-09	0.425
5	63840208	65083148	459	4.74E-06	2.54E-09	0.925
5	65083209	65909616	334	-2.75E-05	2.66E-09	0.594
5	65910316	67096208	520	2.20E-05	3.61E-09	0.714
5	67096780	70682458	689	-3.43E-05	5.23E-09	0.635
5	70683839	72420748	629	-5.22E-05	3.31E-09	0.364
5	72420784	73507955	557	-3.29E-05	4.35E-09	0.617
5	73508509	75240469	728	-1.17E-04	4.70E-09	0.088
5	75242395	75740199	233	1.80E-06	1.75E-09	0.966
5	75743532	77203135	738	1.16E-04	5.80E-09	0.129
5	77203718	79068089	684	-9.78E-06	4.62E-09	0.886
5	79068300	80480761	705	1.94E-05	5.70E-09	0.797
5	80481996	81712715	433	-5.57E-05	2.96E-09	0.306
5	81714078	82658571	362	-4.06E-05	2.81E-09	0.445
5	82660901	84397685	586	-1.35E-05	5.17E-09	0.851
5	84399219	85315500	310	2.33E-05	1.69E-09	0.571
5	85316429	86490293	328	2.98E-05	2.32E-09	0.536
5	86492986	88065637	305	-9.21E-06	1.54E-09	0.814
5	88069593	90500398	713	1.16E-06	4.77E-09	0.987

5	90504842	93602613	719	-3.86E-05	4.05E-09	0.545
5	93607443	95822590	794	5.11E-05	5.43E-09	0.488
5	95822732	96867936	466	-7.18E-05	3.01E-09	0.191
5	96870861	97435808	143	-2.23E-06	9.51E-10	0.942
5	97436504	99258325	558	-4.32E-05	3.30E-09	0.452
5	99259751	101101103	482	-3.53E-05	2.51E-09	0.480
5	101101325	102681586	443	5.38E-06	2.02E-09	0.905
5	102686157	104542488	700	1.50E-05	5.30E-09	0.837
5	104543131	105885913	312	1.41E-05	2.00E-09	0.752
5	105887333	106800282	343	-2.95E-05	2.60E-09	0.563
5	106800765	108633176	758	2.02E-05	5.89E-09	0.793
5	108633347	109231597	246	-5.15E-06	1.29E-09	0.886
5	109232816	110819753	510	5.35E-07	3.24E-09	0.993
5	110821786	111962676	471	-4.89E-05	3.76E-09	0.425
5	111963025	112396750	156	5.75E-06	8.41E-10	0.843
5	112397011	113603768	561	2.87E-07	3.57E-09	0.996
5	113604579	114448326	333	-1.08E-06	2.21E-09	0.982
5	114448597	115828291	681	-6.26E-05	4.71E-09	0.362
5	115829425	117003674	543	-6.27E-05	4.16E-09	0.331
5	117004531	117712427	268	1.65E-05	1.93E-09	0.707
5	117714517	119668423	688	-2.64E-05	4.86E-09	0.705
5	119669457	120962383	495	-1.56E-05	3.68E-09	0.797
5	120963248	121481183	148	2.58E-05	9.81E-10	0.410
5	121485609	122603725	451	-8.71E-08	2.96E-09	0.999
5	122604501	123236124	257	4.14E-06	1.67E-09	0.919
5	123238611	123797013	229	2.66E-05	1.78E-09	0.529
5	123798436	124746813	465	2.17E-05	4.23E-09	0.738
5	124748624	126938920	874	-4.72E-05	6.24E-09	0.550
5	126940058	128727903	666	-5.23E-05	4.04E-09	0.411
5	128730275	130376511	412	-9.63E-07	1.81E-09	0.982
5	130376936	131873073	450	9.82E-06	2.32E-09	0.838
5	131874022	132603137	224	2.46E-05	1.35E-09	0.503
5	132609517	134321546	718	-1.59E-04	5.74E-09	0.035
5	134329463	135339464	501	-1.28E-05	3.50E-09	0.829
5	135340951	137948424	906	9.30E-06	4.87E-09	0.894
5	140644957	141640050	475	-2.41E-05	3.93E-09	0.701
5	137950183	140644484	766	-1.13E-05	3.40E-09	0.847

5	141640419	142230251	276	-2.11E-08	2.64E-09	1.000
5	142230949	144583138	963	6.67E-05	5.83E-09	0.383
5	144584265	145803004	431	-6.24E-06	2.19E-09	0.894
5	145803849	147651230	762	-3.14E-05	5.30E-09	0.666
5	150395351	150917796	368	-4.32E-05	2.65E-09	0.402
5	147665223	150393107	1253	-3.12E-07	8.32E-09	0.997
5	150918158	152360494	594	8.69E-05	3.73E-09	0.155
5	152361546	152864614	177	2.29E-06	1.05E-09	0.944
5	152867348	153494463	316	2.02E-05	1.59E-09	0.613
5	153495193	154448863	413	-1.72E-05	3.11E-09	0.757
5	154449856	155290211	358	3.70E-05	2.33E-09	0.444
5	155290613	156628287	483	-9.31E-06	3.31E-09	0.871
5	156629754	157197141	306	-6.07E-05	1.99E-09	0.173
5	157199547	158491655	551	6.25E-05	3.48E-09	0.289
5	158492279	159135376	279	6.06E-05	2.23E-09	0.200
5	159136348	160908680	774	2.26E-05	5.88E-09	0.768
5	160910101	161688620	224	-6.23E-06	1.45E-09	0.870
5	161688913	162741298	358	5.63E-05	2.66E-09	0.275
5	162742614	163769136	431	6.72E-05	3.21E-09	0.236
5	163769738	164944949	399	6.46E-05	2.73E-09	0.217
5	164947504	165638940	255	4.51E-06	2.79E-09	0.932
5	165642395	166432731	347	-6.89E-06	3.24E-09	0.904
5	166433367	167760730	549	-3.78E-06	4.62E-09	0.956
5	167761922	169124478	718	5.53E-05	6.25E-09	0.484
5	169125063	170889081	953	-6.87E-06	7.25E-09	0.936
5	170891834	171772889	429	-3.07E-05	3.70E-09	0.614
5	171775715	172376903	328	-1.28E-05	3.24E-09	0.822
5	172377031	173611384	634	-1.52E-04	7.20E-09	0.074
5	173613085	174468408	481	7.63E-05	5.84E-09	0.318
5	174468624	176128648	690	6.28E-05	6.36E-09	0.431
5	176129392	177991305	617	-7.24E-05	7.46E-09	0.402
5	177992368	178410668	199	6.94E-05	1.83E-09	0.105
5	178410839	179245749	390	-9.93E-05	3.47E-09	0.092
5	179245856	180007693	315	3.47E-05	2.79E-09	0.511
5	180008456	180790950	242	-8.98E-06	2.59E-09	0.860
6	63979	824529	331	-9.64E-06	4.05E-09	0.880
6	826312	1582647	523	-3.47E-05	6.41E-09	0.664

6	1582926	2890836	717	-1.04E-05	7.07E-09	0.901
6	2891016	4315210	770	-1.49E-04	7.96E-09	0.095
6	4317398	5056146	426	7.91E-05	3.92E-09	0.206
6	5057399	6246639	735	6.52E-05	7.29E-09	0.445
6	6246984	7328580	685	6.77E-05	8.43E-09	0.461
6	7329417	8791321	740	3.50E-05	6.06E-09	0.653
6	8791421	9760323	373	2.75E-05	3.57E-09	0.645
6	9764413	11234164	726	-8.02E-06	7.40E-09	0.926
6	11235682	12183952	544	-2.74E-06	4.83E-09	0.969
6	12186102	13388480	617	-5.10E-05	5.21E-09	0.480
6	13389521	14618107	554	1.11E-04	4.98E-09	0.115
6	14619148	15998368	596	-1.29E-05	5.44E-09	0.861
6	15998406	17067160	545	6.14E-05	6.44E-09	0.444
6	17069966	19203108	879	2.90E-05	7.31E-09	0.734
6	19203782	20228741	524	-6.82E-06	5.66E-09	0.928
6	20231516	22049758	824	3.90E-05	9.87E-09	0.695
6	22052425	23495209	747	-2.21E-04	1.03E-08	0.029
6	23495260	23935020	233	3.00E-05	2.04E-09	0.506
6	23935352	24799440	438	-7.62E-06	4.75E-09	0.912
6	24803038	25483400	411	-9.46E-06	3.82E-09	0.878
6	25484295	26774948	730	-1.05E-04	1.03E-08	0.301
6	26778827	28669698	609	-1.09E-04	6.48E-09	0.176
6	32900018	33863002	401	-1.12E-04	8.94E-09	0.236
6	33863662	35451187	514	1.63E-04	3.84E-09	0.009
6	35452346	36608267	395	3.25E-05	3.13E-09	0.561
6	36611164	38686222	997	9.07E-05	9.28E-09	0.347
6	38687185	39021712	205	-3.86E-05	2.93E-09	0.476
6	39022698	40344526	675	-4.42E-05	5.95E-09	0.567
6	40345542	42105449	886	-1.95E-04	8.75E-09	0.037
6	42105555	43957473	633	7.54E-05	6.45E-09	0.348
6	43959116	45787468	701	-9.69E-06	6.57E-09	0.905
6	45788111	46556325	327	-3.49E-05	2.55E-09	0.490
6	46560003	47885216	681	-5.85E-05	5.21E-09	0.418
6	47887063	49494241	468	2.06E-05	3.11E-09	0.712
6	49494933	50406476	309	-2.52E-05	2.53E-09	0.617
6	50406575	51703012	476	-1.41E-05	3.73E-09	0.818
6	51704668	53402679	834	-3.66E-05	7.42E-09	0.671

6	53405127	54291407	455	-1.38E-06	3.43E-09	0.981
6	54293261	56103747	744	3.73E-05	5.56E-09	0.617
6	56104522	57082026	352	-8.83E-06	2.47E-09	0.859
6	57085542	58084365	151	2.42E-06	1.17E-09	0.944
6	58139505	62691733	306	1.23E-05	2.03E-09	0.784
6	62692409	63712779	264	9.98E-07	1.61E-09	0.980
6	63713620	65760585	508	-3.36E-05	3.16E-09	0.551
6	65762553	67028107	493	-2.50E-05	3.70E-09	0.681
6	67029501	67556729	242	6.75E-06	2.10E-09	0.883
6	67557245	68546601	377	4.86E-05	3.05E-09	0.379
6	68546995	69295414	301	5.50E-06	3.03E-09	0.920
6	69295526	70359936	399	-8.19E-06	2.94E-09	0.880
6	70360869	72448598	882	5.41E-05	6.99E-09	0.518
6	72449421	73170712	296	2.30E-05	2.51E-09	0.646
6	73171594	75064563	683	6.92E-05	5.50E-09	0.351
6	75065279	75843360	172	-7.72E-06	1.49E-09	0.841
6	75843845	77257094	415	-1.23E-05	2.74E-09	0.815
6	77257275	78957547	680	-4.88E-06	4.93E-09	0.945
6	78958134	79861169	296	1.28E-05	2.09E-09	0.780
6	79862483	80556359	340	1.99E-05	3.10E-09	0.721
6	80557170	82387384	660	-2.81E-05	4.51E-09	0.675
6	82387920	83904869	519	7.24E-07	4.17E-09	0.991
6	83908232	86040334	810	2.65E-05	6.36E-09	0.740
6	86040497	87138115	340	-2.15E-06	2.40E-09	0.965
6	87140364	88532072	486	-1.05E-04	3.28E-09	0.068
6	88533324	89668547	467	5.50E-05	4.75E-09	0.425
6	89673861	91400644	815	9.14E-05	7.41E-09	0.289
6	91401234	93426186	759	-1.54E-05	6.81E-09	0.852
6	93429797	94919277	573	8.18E-05	4.88E-09	0.242
6	94920611	97093037	660	3.99E-05	5.32E-09	0.585
6	97093295	98893182	513	-1.79E-05	3.91E-09	0.774
6	98893830	100286683	584	-1.82E-05	4.93E-09	0.796
6	100287245	101862923	465	-9.21E-06	3.52E-09	0.877
6	101863563	102887537	348	-1.74E-05	3.16E-09	0.757
6	102888281	104211089	445	-4.13E-05	3.18E-09	0.464
6	104211475	105752435	548	-4.44E-05	4.30E-09	0.498
6	105754306	106921804	535	-7.60E-06	5.87E-09	0.921

6	106922281	108322638	655	-1.73E-05	6.44E-09	0.830
6	108325624	110654066	761	-9.19E-05	7.76E-09	0.297
6	110654605	111390344	274	2.71E-05	2.51E-09	0.589
6	111391537	114218733	1067	6.90E-05	8.55E-09	0.456
6	114220361	116130955	594	-3.85E-05	4.70E-09	0.574
6	116130983	117566682	496	1.12E-05	4.53E-09	0.868
6	117567468	119071713	561	2.09E-05	4.45E-09	0.755
6	119075856	120628075	540	-1.16E-05	4.50E-09	0.863
6	120628214	121673627	339	-1.93E-06	2.36E-09	0.968
6	121674337	123104703	522	-5.73E-08	3.93E-09	0.999
6	123105676	123889346	320	1.42E-05	3.04E-09	0.796
6	123890282	125333797	657	-1.95E-05	6.40E-09	0.807
6	125336799	128759251	900	1.09E-05	6.97E-09	0.897
6	128759755	130538497	787	1.18E-05	7.24E-09	0.890
6	130540383	131972731	564	9.30E-05	4.76E-09	0.178
6	131973932	133867802	873	5.60E-05	8.23E-09	0.537
6	136186368	137811295	508	1.42E-04	4.27E-09	0.030
6	133869809	136186007	802	1.41E-05	7.33E-09	0.869
6	137813095	139532424	748	8.27E-05	7.58E-09	0.342
6	139533572	142036032	708	-8.26E-05	5.77E-09	0.277
6	142036912	143719278	602	-3.28E-05	4.98E-09	0.643
6	143720467	146663272	1047	-3.66E-06	8.37E-09	0.968
6	146665424	148179283	592	-3.49E-05	4.87E-09	0.617
6	148180191	148917941	480	-9.34E-05	5.86E-09	0.222
6	148919158	150298842	639	4.10E-06	4.91E-09	0.953
6	150299299	151097980	538	-1.21E-04	6.19E-09	0.124
6	151100484	151929942	491	4.91E-05	5.59E-09	0.512
6	151930249	153550147	880	-1.60E-04	1.05E-08	0.119
6	153551177	155228511	745	-2.85E-05	7.48E-09	0.741
6	156761338	157398853	256	-2.98E-05	2.34E-09	0.538
6	155229566	156756457	768	4.75E-05	7.96E-09	0.594
6	157402041	158192951	273	1.96E-05	2.97E-09	0.719
6	158193244	159318774	547	-4.71E-05	4.16E-09	0.466
6	159319812	159792458	251	-2.22E-05	1.70E-09	0.591
6	159793334	161123451	690	-2.57E-04	2.75E-08	0.120
6	161123771	162298351	679	-5.48E-05	7.06E-09	0.515
6	162300151	163277414	510	7.25E-06	4.49E-09	0.914

6	163277858	164003180	378	6.91E-05	3.75E-09	0.259
6	164006012	165242243	674	1.15E-05	6.25E-09	0.885
6	165246062	166191910	572	1.77E-05	5.82E-09	0.817
6	166193932	167177733	678	7.55E-06	7.08E-09	0.929
6	167178790	168548525	628	3.18E-05	5.47E-09	0.667
6	168549335	169231503	390	-1.76E-06	3.96E-09	0.978
6	169231937	171049558	874	-4.71E-05	7.45E-09	0.586
7	20608	1490775	418	4.39E-06	3.97E-09	0.945
7	1493090	2479029	317	-1.22E-04	5.17E-09	0.090
7	2480729	2930941	207	3.11E-05	1.88E-09	0.473
7	2931676	3736942	499	8.53E-05	4.59E-09	0.208
7	3738087	4783224	512	-3.55E-05	4.72E-09	0.606
7	4783723	5412932	213	2.58E-05	2.62E-09	0.614
7	5414602	6912527	400	1.51E-04	3.93E-09	0.016
7	7168493	7679885	293	-8.49E-05	2.61E-09	0.097
7	7680697	8475147	543	4.61E-05	5.99E-09	0.551
7	8476787	9223721	526	-4.57E-05	5.07E-09	0.521
7	9223762	9893744	341	4.80E-05	3.49E-09	0.417
7	9894421	11298815	623	-1.55E-05	5.16E-09	0.830
7	11299552	12687871	879	-4.37E-06	8.11E-09	0.961
7	12689322	13674908	507	3.34E-06	4.55E-09	0.960
7	13675094	14941781	684	3.76E-05	6.54E-09	0.642
7	14942134	16480989	759	8.33E-05	6.35E-09	0.296
7	16481863	17277084	484	-5.10E-05	5.44E-09	0.489
7	17279294	18861958	710	-3.22E-05	6.31E-09	0.685
7	18863387	19975992	510	-1.12E-04	4.09E-09	0.080
7	19978609	21712233	879	2.41E-05	8.75E-09	0.797
7	21712615	22811384	708	8.91E-05	7.30E-09	0.297
7	22811921	24389035	702	7.66E-05	5.68E-09	0.310
7	24389147	24980408	323	1.24E-05	3.04E-09	0.822
7	24982494	26300992	674	-7.88E-05	7.87E-09	0.374
7	26302668	28038375	768	2.65E-04	3.67E-08	0.166
7	28039590	28710596	382	-6.73E-05	6.40E-09	0.400
7	28710857	30171413	827	-1.43E-05	7.56E-09	0.869
7	30172195	31608495	659	-5.50E-05	5.98E-09	0.477
7	31609317	33150311	750	-3.28E-05	5.98E-09	0.672
7	34940039	35614036	301	-3.09E-05	2.35E-09	0.523

7	33151181	34939884	766	9.45E-05	6.25E-09	0.232
7	35615611	37353302	858	3.24E-05	7.24E-09	0.703
7	37354091	38159855	466	-3.93E-05	4.04E-09	0.537
7	38160558	39534540	677	-3.24E-05	6.04E-09	0.677
7	39535570	40925323	373	-2.86E-06	2.63E-09	0.955
7	40926906	42494824	654	2.61E-05	5.81E-09	0.732
7	42495555	44971837	906	1.32E-05	6.27E-09	0.868
7	44972152	46665658	694	3.55E-05	5.48E-09	0.632
7	46667873	47279128	336	-2.76E-06	2.74E-09	0.958
7	47281095	49130572	862	1.24E-04	7.56E-09	0.155
7	49131824	49821750	300	-8.12E-06	2.27E-09	0.865
7	49822437	51674880	774	-1.90E-05	5.70E-09	0.801
7	51675218	52274523	293	-7.23E-06	2.83E-09	0.892
7	52275656	53175065	478	2.10E-05	3.93E-09	0.738
7	53348777	54707110	519	9.34E-06	3.75E-09	0.879
7	54707337	55641707	451	-5.16E-05	3.88E-09	0.408
7	55641798	57382254	387	1.21E-05	2.92E-09	0.823
7	57382271	62556935	174	-5.92E-06	1.01E-09	0.852
7	62557202	64193303	281	9.23E-06	1.87E-09	0.831
7	64699988	65570171	152	4.93E-06	8.12E-10	0.863
7	65570310	66453460	219	1.46E-05	8.54E-10	0.616
7	66454071	67369810	339	-4.85E-05	2.83E-09	0.362
7	67370861	68125403	317	2.08E-06	4.19E-09	0.974
7	68126378	68561421	188	-1.33E-05	1.57E-09	0.736
7	68562932	69806895	289	-1.28E-05	1.72E-09	0.757
7	69808153	71204524	594	-1.23E-05	6.27E-09	0.877
7	71205507	71873571	249	-1.58E-05	1.81E-09	0.710
7	71874997	73996533	396	2.84E-05	2.91E-09	0.599
**7**	**73997700**	**76550299**	**361**	**-2.68E-04**	**3.81E-09**	**0.000**
7	76551983	77101532	181	-5.00E-06	1.94E-09	0.910
7	77102731	77953248	411	-3.16E-06	3.14E-09	0.955
7	77954163	78839317	553	-8.90E-05	5.09E-09	0.213
7	78843289	80546745	614	1.27E-06	4.60E-09	0.985
7	80547047	82758529	882	-7.67E-05	8.22E-09	0.397
7	82758644	83959105	533	6.17E-06	4.28E-09	0.925
7	83959751	85570789	448	-3.15E-05	2.85E-09	0.556
7	85571372	86341446	218	-3.73E-05	1.42E-09	0.321

7	88732470	89270556	217	-3.57E-05	1.68E-09	0.384
7	86345013	88732142	836	2.96E-05	5.47E-09	0.689
7	89271899	91173749	774	4.40E-05	5.81E-09	0.564
7	91174778	93339775	617	9.10E-05	4.07E-09	0.154
7	93340137	95171731	630	2.33E-05	5.28E-09	0.748
7	95419256	96611864	459	-1.43E-05	3.40E-09	0.806
**7**	**96613246**	**98151455**	**496**	**-4.00E-04**	**8.19E-09**	**0.000**
7	98152191	100555361	665	6.45E-05	5.06E-09	0.364
7	100606141	101730309	387	-4.55E-05	4.72E-09	0.507
7	101731567	103193331	317	1.10E-05	2.37E-09	0.821
7	103193651	104157576	550	-8.94E-06	4.58E-09	0.895
7	104158491	105425027	481	-1.62E-05	3.26E-09	0.777
7	105427565	106052506	320	8.31E-06	3.40E-09	0.887
7	106052873	107305328	456	-1.97E-05	3.62E-09	0.744
7	107306421	108375939	543	-7.89E-05	3.91E-09	0.208
7	108376173	109584769	351	-8.72E-06	2.52E-09	0.862
7	109587777	111447153	593	-8.07E-05	4.21E-09	0.214
7	111450919	113336970	590	-1.40E-06	4.86E-09	0.984
7	113337327	116315205	824	-1.85E-05	5.90E-09	0.809
7	116317043	118347634	570	1.85E-05	3.63E-09	0.759
7	118348359	121094386	573	1.22E-05	3.43E-09	0.836
7	121094460	122368663	412	2.16E-05	3.36E-09	0.709
7	122369853	124154451	639	-3.95E-05	4.10E-09	0.537
7	124155319	125386718	379	4.26E-05	2.26E-09	0.371
7	125387168	126516683	442	-1.09E-05	2.98E-09	0.842
7	126517818	127905983	588	-7.85E-06	3.73E-09	0.898
7	127907054	128554075	209	-1.13E-05	1.77E-09	0.789
7	128555262	130074230	546	1.35E-05	4.27E-09	0.836
7	130075370	130797123	249	8.15E-07	2.65E-09	0.987
7	130797776	131856855	498	5.28E-05	4.85E-09	0.448
7	131857419	134306672	895	1.09E-04	6.24E-09	0.168
7	134307114	135464701	504	2.57E-05	4.01E-09	0.685
7	135466022	137137511	701	1.19E-05	5.74E-09	0.876
7	137138736	138742981	587	1.59E-05	6.38E-09	0.842
7	138744100	141106552	845	-7.48E-05	6.78E-09	0.364
7	141109797	142655113	557	6.28E-05	3.80E-09	0.308
7	142655717	144274735	430	-4.76E-06	3.16E-09	0.932

7	144276424	146018520	552	-1.55E-05	4.35E-09	0.814
7	146023023	147204086	503	5.03E-05	3.82E-09	0.415
7	147204549	148619685	685	1.16E-04	5.70E-09	0.124
7	148620581	150491084	655	2.17E-06	5.26E-09	0.976
7	150491851	151390799	462	-6.55E-05	4.74E-09	0.342
7	151391044	152409155	288	-5.88E-06	4.63E-09	0.931
7	152410248	153080056	288	1.73E-05	4.00E-09	0.785
7	153081698	153934166	320	2.13E-06	3.16E-09	0.970
7	153934210	154963787	621	2.16E-04	5.99E-09	0.005
7	154964316	155379409	239	-6.08E-05	3.75E-09	0.320
7	155380353	156814410	652	-4.95E-05	6.56E-09	0.541
7	156815867	158029340	505	4.97E-06	4.93E-09	0.944
7	158030463	158759939	286	-7.95E-06	2.05E-09	0.861
7	158760677	159127587	134	-2.31E-05	1.46E-09	0.544
8	246934	1162157	489	-8.01E-06	5.74E-09	0.916
8	1162454	1625424	273	9.45E-06	3.26E-09	0.869
8	1625732	2042364	303	4.34E-05	5.10E-09	0.543
8	2043187	2680255	318	3.40E-05	4.22E-09	0.601
8	2681051	3358423	619	-2.11E-05	7.49E-09	0.807
8	3358433	3584832	297	-2.60E-05	3.24E-09	0.649
8	3585058	4071438	628	-4.79E-05	6.70E-09	0.559
8	4071516	4274957	255	7.56E-06	2.80E-09	0.886
8	4275286	4479596	220	2.02E-06	3.25E-09	0.972
8	4479836	4754328	302	2.86E-05	3.13E-09	0.609
8	4755088	5146808	311	-1.91E-05	3.37E-09	0.742
8	5147734	5781592	597	3.36E-05	6.29E-09	0.672
8	5781947	6017670	226	-4.90E-05	2.11E-09	0.286
8	6018292	6350873	272	-1.98E-05	2.59E-09	0.697
8	6352358	7793703	489	-3.96E-05	5.46E-09	0.592
8	7793837	8735213	412	-7.19E-06	3.83E-09	0.907
8	8736386	10117293	800	-3.80E-05	7.79E-09	0.667
8	10117411	10535826	286	-1.99E-05	3.34E-09	0.730
8	10537940	11249261	410	-3.56E-05	3.51E-09	0.547
8	11250848	11836622	364	-6.07E-05	4.84E-09	0.383
8	11838355	12975295	284	-9.40E-06	3.07E-09	0.865
8	12975578	13940652	686	-3.41E-05	6.86E-09	0.680
8	13942500	14876660	523	-9.24E-05	4.81E-09	0.183

8	14877288	15273777	321	4.03E-06	2.78E-09	0.939
8	15274608	16087631	534	-4.22E-05	5.44E-09	0.567
8	16087729	17579518	803	-1.23E-08	8.50E-09	1.000
8	17581106	18670645	722	-6.35E-05	6.70E-09	0.438
8	18670908	19490180	560	-2.79E-05	5.91E-09	0.717
8	21022075	21402658	257	4.49E-06	2.49E-09	0.928
8	19491428	21021401	888	1.27E-04	9.59E-09	0.196
8	21404381	22539316	482	1.64E-05	4.14E-09	0.798
8	22539641	23548146	588	-7.95E-05	1.58E-08	0.528
8	23548749	24456913	317	2.21E-05	2.83E-09	0.678
8	24457122	25461315	424	-9.59E-06	3.37E-09	0.869
8	25461344	26681450	716	-2.33E-05	7.35E-09	0.786
8	26681799	27404354	421	-1.54E-05	3.59E-09	0.797
8	27405207	27759126	214	-4.93E-05	1.65E-09	0.225
8	27759839	29277590	622	-6.64E-05	5.56E-09	0.373
8	29277652	31947675	956	2.05E-06	8.67E-09	0.982
8	31947880	32448740	282	-2.13E-05	2.69E-09	0.681
8	32449533	33980486	355	6.67E-06	2.61E-09	0.896
8	33981742	35313077	399	4.88E-05	2.57E-09	0.336
8	35314837	37375458	435	-1.02E-06	3.40E-09	0.986
8	37376914	39779989	596	-6.07E-05	5.56E-09	0.416
8	39781444	40415799	340	8.77E-06	3.28E-09	0.878
8	40417433	41718356	592	-1.62E-05	5.82E-09	0.831
8	41719750	42501174	186	2.39E-05	1.47E-09	0.532
8	42501717	49028667	390	7.72E-06	2.27E-09	0.871
8	49038732	52156842	816	3.98E-05	5.37E-09	0.587
8	52157415	54115712	718	-3.73E-05	5.68E-09	0.620
8	54116775	55914038	659	5.25E-05	6.25E-09	0.507
8	55914314	57653028	655	-2.03E-05	5.77E-09	0.789
8	57655067	58674153	403	-5.90E-05	3.30E-09	0.305
8	58675131	59568410	383	1.87E-05	3.61E-09	0.756
8	59570176	61809929	767	-1.13E-04	6.53E-09	0.162
8	61810694	63710298	771	-8.74E-05	6.47E-09	0.277
8	63711348	65193342	460	-2.09E-05	3.81E-09	0.735
8	65195953	66024947	260	-6.53E-06	2.12E-09	0.887
8	66025862	67192642	387	-3.52E-05	3.07E-09	0.525
8	67193708	69108839	502	-4.78E-05	4.45E-09	0.473

8	69111244	70003448	401	4.82E-05	4.13E-09	0.453
8	70003607	72013005	727	2.69E-05	6.55E-09	0.739
8	72014069	72749564	427	-3.12E-05	4.68E-09	0.649
8	72751469	73423214	352	-5.67E-05	3.49E-09	0.337
8	73423786	74996061	687	-9.56E-07	6.15E-09	0.990
8	74998868	76294909	508	7.43E-05	4.47E-09	0.266
8	76295292	78015335	544	-2.16E-05	4.39E-09	0.744
8	78015611	78632215	197	1.11E-05	1.50E-09	0.775
8	78632893	79831888	374	-1.94E-05	2.63E-09	0.705
8	79832211	80654655	273	1.33E-06	2.48E-09	0.979
8	80656206	81330540	223	1.87E-05	1.92E-09	0.670
8	81331077	83726996	809	4.30E-05	7.17E-09	0.612
8	83727741	85093121	385	2.47E-05	3.35E-09	0.669
8	85094979	86979097	352	-8.24E-06	2.40E-09	0.866
8	86990451	87562786	212	8.07E-06	1.45E-09	0.832
8	87563878	89083319	540	-3.36E-05	4.49E-09	0.616
8	89083979	90605445	456	2.47E-05	3.39E-09	0.672
8	90607207	92342283	523	2.29E-05	3.59E-09	0.702
8	92344138	94269630	544	-2.46E-05	5.05E-09	0.729
8	94272436	95810190	578	-6.15E-05	4.91E-09	0.380
8	95810772	96533604	390	1.19E-05	3.79E-09	0.847
8	96534326	97318660	396	5.07E-05	3.95E-09	0.420
8	97320605	98867870	622	1.71E-05	5.34E-09	0.815
8	98869940	101450242	829	3.43E-05	6.22E-09	0.663
8	101451169	102937063	656	2.77E-05	6.60E-09	0.733
8	102944509	103730879	375	8.14E-06	4.15E-09	0.899
8	103970185	105389708	566	7.45E-06	4.43E-09	0.911
8	105391381	107409808	782	4.22E-05	6.80E-09	0.609
8	107410067	108108114	325	2.57E-05	2.68E-09	0.619
8	108115161	108646193	244	3.77E-06	2.41E-09	0.939
8	108646968	110761074	744	-5.11E-05	6.98E-09	0.541
8	110761776	112611637	402	1.93E-05	2.98E-09	0.723
8	112612842	114393054	481	-6.30E-06	3.16E-09	0.911
8	114394256	116095059	617	-9.31E-07	4.58E-09	0.989
8	116095815	117130004	272	9.10E-05	6.19E-09	0.247
8	117131947	118622352	563	2.38E-05	5.16E-09	0.741
8	118623844	120216957	719	4.96E-05	6.70E-09	0.545

8	120217356	121767384	601	1.56E-05	4.56E-09	0.817
8	121770585	123545992	691	3.41E-05	6.63E-09	0.675
8	123547148	124483938	403	6.59E-05	4.19E-09	0.309
8	124486753	125247268	404	1.31E-05	4.19E-09	0.840
8	125248576	126268046	461	-5.75E-05	4.64E-09	0.398
8	126269921	127434945	507	9.07E-06	7.53E-09	0.917
8	127436003	128162974	338	1.18E-04	6.06E-08	0.633
8	128166556	128542444	193	-1.54E-04	5.83E-08	0.524
8	128543956	129297518	432	-1.43E-04	9.87E-09	0.150
8	129297807	130379330	412	1.50E-05	3.53E-09	0.800
8	130380171	130812339	231	5.15E-06	2.23E-09	0.913
8	130814803	131553214	284	-3.37E-05	2.41E-09	0.492
8	131553327	132358990	430	5.09E-05	4.07E-09	0.425
8	132359241	133454722	553	-8.22E-07	4.76E-09	0.990
8	133455530	134566686	734	5.79E-05	6.82E-09	0.483
8	134567348	135269827	423	2.20E-05	4.76E-09	0.750
8	135270868	136209239	515	1.04E-05	4.98E-09	0.883
8	136209325	137306730	483	-1.49E-05	3.64E-09	0.805
8	137307361	138223632	346	-1.80E-05	2.81E-09	0.734
8	138224181	139194661	572	9.04E-05	4.98E-09	0.200
8	139195331	140093896	520	-9.83E-06	5.68E-09	0.896
8	140094627	141355502	731	3.95E-05	7.09E-09	0.639
8	143750619	146303789	716	-4.67E-05	5.80E-09	0.540
8	141358681	143750205	923	-3.02E-05	9.14E-09	0.752
9	342841	1006606	533	3.33E-05	4.78E-09	0.630
9	1007923	1792147	679	1.16E-05	5.65E-09	0.878
9	1792277	2665240	686	-3.24E-05	7.07E-09	0.700
9	2666964	3348816	358	-1.16E-04	2.88E-09	0.031
9	3349487	4378727	700	-9.33E-05	7.31E-09	0.275
9	4380096	4590305	142	4.53E-05	1.50E-09	0.243
9	4591655	5273194	342	1.30E-04	2.47E-09	0.009
9	5273896	6144065	384	-1.62E-05	2.78E-09	0.758
9	6144333	7232230	569	5.82E-05	4.62E-09	0.392
9	7232510	7757891	465	-2.11E-05	4.08E-09	0.741
9	7758579	8235898	325	5.68E-05	2.75E-09	0.279
9	8236122	9374664	803	8.13E-05	7.76E-09	0.356
9	9375194	9793342	253	-1.71E-05	2.17E-09	0.714

9	9793846	10358286	350	-5.57E-05	2.99E-09	0.308
9	10358672	10879071	263	5.38E-05	2.52E-09	0.283
9	10879188	11616822	307	-1.91E-05	3.33E-09	0.740
9	11884425	12274220	180	1.01E-06	1.36E-09	0.978
9	12274774	12778224	274	-6.82E-06	2.19E-09	0.884
9	12780295	13797055	477	1.44E-05	4.17E-09	0.823
9	13797496	14712257	605	4.16E-05	7.70E-09	0.635
9	14714681	16054406	644	-1.20E-04	4.85E-09	0.086
9	16056127	16833797	538	-8.87E-05	5.00E-09	0.210
9	16836011	17627988	363	2.43E-05	2.50E-09	0.627
9	17628726	18659920	604	2.14E-05	4.72E-09	0.755
9	18660695	19129349	353	-6.97E-05	5.08E-09	0.328
9	19456098	20324812	482	2.39E-05	4.08E-09	0.708
9	20324848	21549638	502	2.43E-05	3.74E-09	0.692
9	21550959	22968575	596	-6.84E-05	4.46E-09	0.306
9	22968789	24154728	439	4.20E-05	4.56E-09	0.534
9	24155142	25425160	409	3.50E-05	3.24E-09	0.539
9	25425511	26317104	404	6.59E-05	4.20E-09	0.309
9	26317973	27335346	535	-4.34E-05	4.63E-09	0.523
9	27336207	28252383	497	-5.36E-05	4.76E-09	0.437
9	28253887	29622939	660	2.76E-06	4.64E-09	0.968
9	29623053	30819576	414	2.63E-06	2.36E-09	0.957
9	30821676	31836697	373	-2.56E-05	2.48E-09	0.607
9	31837040	32249649	172	-4.00E-06	1.07E-09	0.903
9	32250247	33186788	464	-5.07E-05	3.28E-09	0.376
9	33187897	34769004	521	-2.57E-04	6.92E-09	0.002
9	34771534	36712124	645	-5.71E-05	5.08E-09	0.423
9	36714252	37678932	456	3.12E-05	3.73E-09	0.609
9	37679713	42220384	544	4.45E-05	4.77E-09	0.520
9	69576527	71968825	445	-2.06E-05	3.38E-09	0.723
9	71969482	73398448	622	-1.12E-05	3.88E-09	0.858
9	73399750	73916072	309	-3.44E-05	2.02E-09	0.444
9	73916583	74600739	231	-2.69E-05	1.96E-09	0.544
9	74600822	75517582	356	-3.85E-05	2.19E-09	0.411
9	75519251	76785168	379	-1.00E-05	2.63E-09	0.845
9	76786297	78214833	562	-2.05E-05	4.41E-09	0.757
9	78215299	78900183	469	2.97E-06	5.68E-09	0.969

9	81066078	81860861	384	-5.38E-06	3.18E-09	0.924
9	78901112	81064897	968	-3.53E-06	7.69E-09	0.968
9	81863722	82348771	246	-3.76E-05	2.35E-09	0.438
9	82349606	83041982	353	2.16E-05	3.11E-09	0.698
9	83043376	83455568	219	5.78E-05	1.65E-09	0.155
9	83455867	84388566	421	-5.69E-05	3.53E-09	0.339
9	84389168	86180165	683	-1.48E-05	5.41E-09	0.841
9	87571959	87811640	150	4.83E-06	1.08E-09	0.883
9	86181461	87571563	629	-9.14E-05	4.24E-09	0.161
9	87813612	88867151	355	1.97E-05	2.44E-09	0.689
9	88869630	89948499	516	-4.64E-05	4.09E-09	0.468
9	89949778	90198251	198	-4.76E-05	1.56E-09	0.228
9	90198375	90893610	338	-1.62E-07	2.79E-09	0.998
9	90893774	91608628	247	-3.52E-05	1.93E-09	0.423
9	91610543	92342927	327	1.87E-05	2.78E-09	0.723
9	92443570	93441674	461	4.77E-05	3.67E-09	0.431
9	93442205	94163515	361	5.81E-05	3.15E-09	0.301
9	94164373	95854872	626	2.35E-05	4.53E-09	0.727
9	95855100	97719592	731	4.71E-05	4.72E-09	0.493
9	97720818	98889276	456	-1.96E-05	3.16E-09	0.728
9	98891743	100917602	625	2.06E-05	3.77E-09	0.737
9	100919318	101473404	376	-4.27E-05	3.35E-09	0.460
9	101474837	103543692	679	-9.50E-05	3.85E-09	0.125
9	103544757	103969076	179	1.30E-07	8.80E-10	0.996
9	103969339	104552711	293	1.94E-05	2.22E-09	0.681
9	104553938	105041375	303	-2.37E-05	2.35E-09	0.626
9	105041756	105743445	320	-6.05E-06	2.17E-09	0.897
9	105744075	106487777	333	2.12E-05	2.54E-09	0.674
9	106488291	107545903	496	3.41E-05	2.57E-09	0.501
9	107546901	108751162	555	1.47E-05	4.09E-09	0.818
9	108754828	109972524	499	-8.52E-05	5.90E-09	0.268
9	109974206	111137001	674	5.18E-05	7.93E-09	0.561
9	111138232	111997222	549	1.30E-05	3.80E-09	0.833
9	112001473	112593531	326	-1.30E-05	2.43E-09	0.792
9	112594858	113255402	401	2.19E-05	3.72E-09	0.720
9	113255544	114077164	462	2.77E-05	3.64E-09	0.646
9	114078103	114828332	330	-4.79E-05	2.43E-09	0.332

9	114830330	115391053	230	-8.79E-07	1.19E-09	0.980
9	115393806	116148630	399	-2.80E-05	3.17E-09	0.619
9	116149967	117306807	689	-6.83E-05	5.82E-09	0.371
9	117307880	118569429	682	-1.92E-05	4.95E-09	0.784
9	118570426	120030166	743	-5.88E-06	5.95E-09	0.939
9	121009165	121318886	151	9.16E-06	1.11E-09	0.783
9	120030269	121008844	499	2.24E-05	3.64E-09	0.710
9	121319662	122258204	434	-3.79E-05	3.09E-09	0.495
9	122258577	124870612	937	5.49E-05	5.97E-09	0.477
9	124871322	125542861	336	-3.97E-05	2.96E-09	0.466
9	125545194	126926376	449	-5.53E-05	3.06E-09	0.317
9	128927088	129477470	292	4.43E-05	3.46E-09	0.451
9	126927204	128926989	624	-3.16E-05	3.32E-09	0.583
9	129477886	131429495	582	3.29E-05	3.93E-09	0.600
9	131430602	132891863	505	-8.62E-05	4.08E-09	0.177
9	132895131	133640777	305	-5.49E-06	3.60E-09	0.927
9	133642918	134639655	432	-3.13E-05	3.22E-09	0.582
9	134640928	135401279	345	6.45E-05	4.13E-09	0.316
9	135401721	136417159	501	6.12E-05	5.15E-09	0.394
9	136429816	136979539	317	4.75E-05	3.43E-09	0.417
9	136979744	137928846	495	-2.16E-05	5.62E-09	0.773
9	137929853	138817142	514	-4.79E-05	6.03E-09	0.537
9	138817178	141146684	723	-1.43E-05	5.74E-09	0.850
10	60969	747115	154	-1.79E-05	1.31E-09	0.620
10	747772	1330771	280	2.51E-05	2.25E-09	0.596
10	1331955	1609370	172	-1.26E-05	1.69E-09	0.760
10	1610839	2104472	389	-4.57E-05	3.58E-09	0.445
10	2106418	3143283	689	1.22E-05	6.26E-09	0.877
10	3144251	3785605	441	1.72E-05	3.84E-09	0.781
10	3786476	5358542	956	5.12E-05	8.07E-09	0.569
10	5359010	6316910	614	5.90E-05	5.28E-09	0.417
10	6318458	7639684	883	9.82E-05	8.95E-09	0.299
10	9433090	10540465	440	-5.92E-06	3.44E-09	0.920
10	7639932	9432277	1108	-1.11E-04	9.21E-09	0.249
10	12092208	12798954	371	-5.71E-05	4.00E-09	0.367
10	10542217	12092029	828	-1.06E-04	7.49E-09	0.223
10	12799286	13718384	597	-3.23E-05	5.57E-09	0.665

10	13719970	14411956	499	1.20E-05	5.18E-09	0.867
10	14412084	15303527	489	6.48E-06	5.11E-09	0.928
10	15303983	16443442	476	1.56E-05	3.38E-09	0.788
10	16444693	17591626	664	-4.92E-05	5.75E-09	0.516
10	17591919	19202731	694	1.04E-05	5.17E-09	0.885
10	19203300	20131614	450	-2.53E-06	3.18E-09	0.964
10	20132516	21243558	680	-6.21E-05	5.47E-09	0.401
10	21244087	23696890	631	-3.84E-05	6.04E-09	0.621
10	23698033	25414215	686	-1.89E-05	4.86E-09	0.786
10	25416598	26631987	555	9.27E-06	3.82E-09	0.881
10	26633775	27667904	411	7.00E-05	2.93E-09	0.196
10	27668052	28734655	508	-5.44E-05	3.68E-09	0.370
10	28735045	29174963	234	-8.55E-06	1.67E-09	0.834
10	29175101	29961838	535	1.59E-05	7.15E-09	0.851
10	29962511	30653567	388	6.91E-05	3.52E-09	0.244
10	30654528	31479103	433	6.26E-05	4.03E-09	0.324
10	31479422	33656047	779	2.07E-05	4.75E-09	0.764
10	36020445	36884906	379	1.16E-05	3.17E-09	0.837
10	33656119	36017592	829	9.14E-07	5.39E-09	0.990
10	36885921	37845308	313	2.12E-06	3.09E-09	0.970
10	38367233	43220539	218	2.84E-07	1.12E-09	0.993
10	43222372	43605902	162	-1.31E-05	1.53E-09	0.738
10	43606687	45247184	692	3.01E-07	4.62E-09	0.996
10	45247418	46769302	372	1.45E-04	3.58E-09	0.016
10	47768664	49812917	361	-2.30E-05	3.70E-09	0.706
10	49816189	50839403	568	-1.89E-05	4.20E-09	0.771
10	50840736	52086621	209	-6.40E-05	1.07E-08	0.535
10	52086939	52707026	238	-2.99E-05	1.50E-09	0.440
10	52708822	53362540	343	-3.56E-05	2.80E-09	0.501
10	53362932	54413554	512	6.95E-06	4.09E-09	0.913
10	54413909	55334166	523	-8.69E-05	4.20E-09	0.180
10	55334740	56204677	453	-2.16E-06	2.74E-09	0.967
10	56207098	56883966	279	1.52E-05	3.53E-09	0.798
10	56885639	58806290	704	-5.67E-05	4.05E-09	0.373
10	59158617	59822195	312	4.26E-05	1.90E-09	0.328
10	59822974	60375613	206	3.38E-06	1.09E-09	0.919
10	60375857	62558737	972	-9.31E-05	7.03E-09	0.267

10	62558842	64263322	626	-4.12E-05	4.52E-09	0.540
10	64263402	65469133	435	-8.05E-06	2.73E-09	0.878
10	65735028	66980708	394	1.56E-05	2.59E-09	0.759
10	66980980	67545544	227	2.25E-06	1.71E-09	0.957
10	67546331	68429563	514	-2.16E-05	3.99E-09	0.732
10	68430236	69893580	549	-4.51E-05	6.99E-09	0.589
10	69896336	71052508	440	-2.41E-06	3.32E-09	0.967
10	71052888	72479853	930	-3.14E-05	9.81E-09	0.751
10	72480072	73612902	784	-1.27E-05	7.99E-09	0.887
10	73614288	75696752	549	1.02E-05	2.09E-09	0.823
10	78642246	79740262	595	-1.57E-05	4.64E-09	0.818
10	75700056	78638957	888	4.67E-05	5.01E-09	0.509
10	79742617	80874092	594	-5.83E-05	5.70E-09	0.440
10	80874523	81881954	307	1.51E-04	7.18E-09	0.074
10	81882370	82378624	279	3.77E-05	3.65E-09	0.533
10	82379403	82881996	243	3.29E-05	2.19E-09	0.482
10	82883949	83597042	269	-6.01E-05	2.15E-09	0.195
10	83597818	84383108	342	1.38E-06	2.29E-09	0.977
10	84383277	85424102	487	2.73E-05	3.11E-09	0.624
10	85424493	86459792	528	-9.35E-05	4.40E-09	0.159
10	86460169	87285511	356	1.82E-05	2.21E-09	0.699
10	87285907	88450872	497	-1.76E-05	4.23E-09	0.786
10	88451869	89845801	371	1.37E-05	2.85E-09	0.798
10	89846944	90734906	410	7.55E-06	3.18E-09	0.894
10	90735832	91959588	592	6.41E-05	4.73E-09	0.351
10	91960142	92682235	320	3.05E-05	1.98E-09	0.494
10	92682369	94196248	469	2.01E-05	3.07E-09	0.717
10	94196690	95064828	355	1.60E-05	2.01E-09	0.722
10	95065655	95946938	409	1.13E-06	3.35E-09	0.984
10	95949432	97984754	853	-7.71E-05	4.84E-09	0.268
10	97985690	99554180	665	-8.75E-05	4.70E-09	0.202
10	99554895	100240681	333	-5.40E-05	2.51E-09	0.282
10	100241204	102089663	714	5.65E-07	4.25E-09	0.993
10	102090924	104207799	690	-2.12E-05	4.15E-09	0.742
10	104208761	106139081	683	-1.22E-04	6.68E-09	0.135
10	106139294	107874146	733	-1.88E-05	4.67E-09	0.784
10	107875182	109577686	742	2.54E-05	5.72E-09	0.737

10	109578567	110311094	318	-1.77E-05	2.08E-09	0.698
10	110311425	112559414	764	1.11E-05	4.88E-09	0.873
10	112560012	113839866	517	-8.47E-06	3.21E-09	0.881
10	113845990	115698315	912	-7.08E-05	7.94E-09	0.427
10	117705146	118416795	358	-1.02E-06	2.55E-09	0.984
10	115700005	117702645	679	-3.74E-05	4.40E-09	0.572
10	118417453	119697663	621	-2.00E-05	5.65E-09	0.790
10	119698700	120994671	529	-3.23E-05	4.46E-09	0.629
10	120997666	121874991	393	4.10E-05	3.15E-09	0.465
10	121875531	122805770	507	1.11E-04	6.26E-09	0.161
10	122807254	123853223	521	9.40E-05	4.55E-09	0.164
10	123855124	124894743	544	-8.06E-06	4.33E-09	0.902
10	124894909	125868860	561	6.20E-05	5.51E-09	0.404
10	125919795	126739513	451	7.75E-05	7.80E-09	0.380
10	126739798	127820978	517	5.63E-05	3.91E-09	0.368
10	127821105	128409952	405	-6.11E-06	3.86E-09	0.922
10	128410393	129482709	632	-8.22E-05	6.84E-09	0.320
10	129483173	130282731	491	2.46E-05	4.78E-09	0.722
10	130282817	131192968	537	-5.38E-05	5.85E-09	0.482
10	131195759	132251820	595	1.89E-05	5.20E-09	0.793
10	132252499	132964299	471	4.62E-05	4.55E-09	0.494
10	132966372	133355925	285	1.45E-05	3.42E-09	0.804
10	133356417	134858046	638	-4.66E-05	5.57E-09	0.533
10	134858720	135524335	243	-7.30E-06	1.75E-09	0.861
11	333347	1057948	320	-2.28E-05	2.37E-09	0.640
11	1058583	1861912	309	5.34E-05	5.62E-09	0.476
11	1862168	2422034	244	-1.19E-04	9.43E-09	0.222
11	2423545	3660412	524	-6.83E-05	5.72E-09	0.366
11	3661873	4745931	519	-4.28E-05	4.31E-09	0.514
11	4746391	5529152	655	-2.01E-05	4.65E-09	0.768
11	5532222	6010718	357	7.81E-05	2.99E-09	0.153
11	6011761	6917273	555	-4.54E-05	4.48E-09	0.498
11	6917772	7715871	483	-7.21E-05	4.78E-09	0.297
11	7717884	9068716	668	1.04E-04	5.24E-09	0.149
11	9069046	10370675	440	-8.06E-05	2.99E-09	0.140
11	10371614	11361034	681	-6.62E-05	6.27E-09	0.403
11	11361458	12018942	492	4.34E-05	4.85E-09	0.533

11	12019010	13133902	717	-9.56E-05	7.12E-09	0.257
11	13134600	14934686	720	3.30E-05	4.62E-09	0.627
11	14936943	16789155	642	-1.86E-05	3.95E-09	0.767
11	16790799	18788126	918	8.17E-05	6.82E-09	0.323
11	18789820	20113591	792	-3.20E-05	7.58E-09	0.713
11	21773680	23111273	502	-7.44E-06	3.58E-09	0.901
11	20115277	21773251	1016	2.23E-05	9.76E-09	0.822
11	24750113	25106557	163	2.52E-06	1.19E-09	0.942
11	23112584	24749839	709	-6.70E-05	6.03E-09	0.389
11	25109191	26046095	403	-1.08E-06	2.78E-09	0.984
11	26046627	27018778	418	4.28E-07	2.88E-09	0.994
11	27019873	28741185	545	2.15E-05	3.40E-09	0.713
11	28742220	30056918	404	-4.99E-06	3.02E-09	0.928
11	30057440	31853968	577	7.48E-06	3.05E-09	0.892
11	31856654	33383448	567	2.99E-05	6.11E-09	0.702
11	33384046	34321466	430	2.22E-05	3.83E-09	0.719
11	34321876	35034307	445	-1.77E-04	9.81E-09	0.074
11	35036029	36117340	598	7.66E-05	5.39E-09	0.297
11	36118027	37838625	746	-1.46E-05	6.23E-09	0.854
11	37839347	40357803	640	2.05E-05	4.29E-09	0.754
11	40359663	40716222	154	-1.67E-05	1.08E-09	0.613
11	40716823	42243196	591	-5.04E-06	4.28E-09	0.939
11	42244050	42854352	257	2.54E-06	1.72E-09	0.951
11	42854878	43952796	470	-4.62E-05	3.50E-09	0.435
11	46252956	47893120	417	-4.43E-05	2.70E-09	0.393
11	43953774	46245900	1084	1.12E-05	1.07E-08	0.914
11	48351331	49659131	215	-1.03E-05	1.06E-09	0.751
11	49660004	55116367	304	-2.18E-05	1.44E-09	0.566
11	55116451	56160712	426	-4.99E-06	2.06E-09	0.913
11	56625775	58455947	713	-3.85E-05	3.91E-09	0.538
11	58457495	59615953	298	2.18E-05	1.53E-09	0.577
11	59620206	61870732	816	-3.22E-05	6.25E-09	0.684
11	61873755	63153602	405	-6.18E-05	3.63E-09	0.305
11	66835372	68515239	414	-3.38E-06	4.74E-09	0.961
11	63154309	66835194	1070	8.65E-05	6.79E-09	0.294
11	68521072	69508567	391	-4.66E-05	1.28E-08	0.681
11	69509343	70613763	469	7.58E-05	5.39E-09	0.302

11	70616465	71842303	364	2.39E-05	2.45E-09	0.630
11	71842976	74158869	681	-4.83E-05	4.89E-09	0.490
11	74159542	76431100	890	3.84E-05	6.41E-09	0.632
11	76432156	77903706	543	1.35E-05	3.09E-09	0.808
11	77904339	79723318	879	7.51E-07	6.58E-09	0.993
11	79723675	80720242	465	5.78E-05	4.50E-09	0.389
11	80721384	81653514	551	1.78E-05	4.45E-09	0.790
11	81654617	82844918	481	-3.30E-05	3.58E-09	0.581
11	82847086	83575703	267	-2.69E-06	1.56E-09	0.946
11	83576460	84208616	304	-1.57E-05	2.37E-09	0.746
11	84212522	84618654	169	4.22E-06	1.52E-09	0.914
11	84618788	86623913	833	1.07E-05	6.02E-09	0.890
11	86625137	87429545	370	-3.14E-05	2.48E-09	0.529
11	87431647	87827395	167	2.77E-06	1.54E-09	0.944
11	87827514	89208590	526	5.12E-05	4.13E-09	0.425
11	89441680	91115475	481	-1.92E-05	2.85E-09	0.719
11	91118661	91676282	198	1.37E-05	1.53E-09	0.726
11	91677374	92626428	367	2.98E-06	2.16E-09	0.949
11	92627643	93270431	278	-2.59E-05	2.07E-09	0.570
11	93272186	94389331	421	-8.89E-06	2.85E-09	0.868
11	94391845	95329108	416	4.65E-05	3.96E-09	0.459
11	95332310	96848965	669	-6.87E-06	5.39E-09	0.925
11	96849530	98703542	778	2.99E-05	6.54E-09	0.712
11	98703733	99613901	508	4.10E-05	4.43E-09	0.538
11	101158517	101892922	344	6.02E-06	2.02E-09	0.894
11	99614560	101156501	814	-2.30E-05	6.73E-09	0.779
11	101894471	102668702	379	-3.02E-05	6.83E-09	0.715
11	102668882	103491310	383	3.33E-06	2.80E-09	0.950
11	103491586	104871322	551	5.81E-05	3.62E-09	0.334
11	104872103	106251514	419	2.53E-05	2.45E-09	0.610
11	106252171	107489662	537	-6.75E-06	4.60E-09	0.921
11	107491606	108495975	327	-6.40E-05	2.09E-09	0.161
11	108496357	110701645	740	4.52E-05	4.81E-09	0.515
11	110702636	111981853	469	4.23E-05	2.99E-09	0.439
11	111985737	113103996	469	5.78E-06	3.27E-09	0.919
11	113105405	113958177	414	-1.15E-04	5.60E-09	0.126
11	113959061	114742322	448	-2.49E-05	3.83E-09	0.687

11	114743956	115627217	415	-1.44E-06	3.51E-09	0.981
11	115652723	117097952	689	2.17E-05	6.40E-09	0.786
11	117098436	118898319	897	1.84E-05	7.28E-09	0.829
11	118899218	120171371	574	6.34E-06	5.04E-09	0.929
11	120173354	121660636	713	-7.07E-05	5.43E-09	0.337
11	121661507	123289851	721	-3.78E-05	7.10E-09	0.654
11	123291301	124403522	618	3.49E-05	4.94E-09	0.620
11	124404297	125984562	770	7.54E-05	6.89E-09	0.364
11	125984970	126772347	528	-2.04E-05	4.84E-09	0.770
11	126772905	127715860	459	1.52E-05	3.85E-09	0.807
11	127716140	129186210	717	-2.80E-05	6.19E-09	0.722
11	129187995	129838375	322	-2.84E-08	3.27E-09	1.000
11	129839528	130892029	437	-9.86E-05	3.97E-09	0.117
11	130894131	131870763	676	2.08E-05	6.64E-09	0.798
11	131871467	132386355	340	-1.19E-05	2.49E-09	0.811
11	132386834	133679495	783	9.07E-06	8.44E-09	0.921
11	133679758	134946382	719	-1.48E-04	7.39E-09	0.085
12	68657	665200	216	1.02E-05	2.03E-09	0.820
12	665655	1602602	326	3.21E-05	2.63E-09	0.531
12	1602652	2176046	281	1.13E-05	3.04E-09	0.837
12	2176265	2886299	325	4.56E-07	3.19E-09	0.994
12	2886946	3558488	354	4.17E-05	3.64E-09	0.489
12	3559429	4693778	656	-9.64E-05	7.29E-09	0.259
12	4695425	5877653	808	5.92E-05	7.35E-09	0.490
12	5878686	6879279	441	9.18E-05	4.36E-09	0.164
12	8658644	10477423	257	-6.10E-07	2.20E-09	0.990
12	10477712	11684725	448	1.88E-05	2.87E-09	0.725
12	11687997	12530718	424	-1.22E-04	4.45E-09	0.067
12	12533494	13362446	381	-3.61E-05	4.49E-09	0.589
12	13362818	14658341	700	2.08E-08	1.13E-08	1.000
12	14659441	16423287	588	-1.01E-05	4.11E-09	0.875
12	16423998	18466533	744	1.80E-05	5.80E-09	0.813
12	18467618	19924288	582	-2.03E-05	5.45E-09	0.783
12	19925541	21568051	878	-4.21E-05	6.10E-09	0.590
12	21569730	22325878	305	-2.10E-05	2.08E-09	0.645
12	22327634	22943718	204	1.56E-05	1.64E-09	0.700
12	22944771	23819079	359	-1.63E-04	3.46E-09	0.005

12	23819364	24545631	412	1.52E-04	4.70E-09	0.026
12	24545834	25991825	716	6.67E-05	5.68E-09	0.376
12	25992543	27529660	775	1.18E-05	7.00E-09	0.888
12	27529712	29111136	700	2.30E-05	4.84E-09	0.740
12	29111480	30354932	709	-4.82E-05	4.86E-09	0.489
12	30355547	31067009	415	-2.56E-06	2.65E-09	0.960
12	31067490	32081072	408	-2.96E-05	3.78E-09	0.630
12	32082695	32645755	311	1.12E-05	2.79E-09	0.832
12	32645848	33435901	369	4.34E-06	2.53E-09	0.931
12	33436298	38231063	325	-1.26E-05	1.73E-09	0.762
12	38233264	40017827	410	-1.93E-05	2.11E-09	0.674
12	40022029	41015211	475	6.21E-06	3.21E-09	0.913
12	41015333	42346846	665	3.35E-05	4.43E-09	0.615
12	42347363	43074061	261	2.76E-05	1.72E-09	0.506
12	43074434	43982146	422	8.62E-06	2.70E-09	0.868
12	43982342	44922863	221	-2.00E-05	1.45E-09	0.600
12	44923334	46022099	342	-3.41E-06	2.22E-09	0.942
12	46023426	47504173	481	1.51E-05	3.09E-09	0.786
12	47505424	48267360	361	2.81E-05	3.56E-09	0.637
12	48267635	48965461	366	1.98E-05	7.85E-09	0.824
12	48967935	50160195	315	1.07E-04	1.74E-09	0.010
12	50160662	51582048	414	1.10E-04	4.64E-09	0.105
12	51582188	52913730	700	5.30E-05	4.77E-09	0.443
12	52914211	54379670	717	6.60E-05	1.62E-08	0.604
12	56795415	57534912	235	6.19E-05	1.57E-09	0.118
12	54380016	56792337	763	-1.18E-04	5.56E-09	0.114
12	57535266	58748272	385	-1.18E-05	2.20E-09	0.802
12	58748846	60859665	699	-2.44E-06	4.23E-09	0.970
12	60859809	61738896	266	-1.27E-06	1.40E-09	0.973
12	61740561	63158569	602	2.47E-05	4.78E-09	0.721
12	63158712	63908647	359	-3.51E-05	2.91E-09	0.514
12	63910665	65383507	408	4.56E-06	3.25E-09	0.936
12	65926411	67039427	476	-2.60E-05	3.14E-09	0.642
12	67039600	67817612	376	6.31E-05	3.91E-09	0.313
12	67818111	68740414	516	-4.12E-05	4.42E-09	0.535
12	68742112	69826093	486	-9.36E-05	4.74E-09	0.174
12	69826237	70815755	526	1.43E-05	3.68E-09	0.814

12	70817279	71688642	413	-4.97E-05	3.61E-09	0.408
12	71690933	72513196	316	2.08E-05	2.15E-09	0.654
12	72525849	74846997	634	-3.97E-06	4.13E-09	0.951
12	75193041	76058346	327	1.46E-05	2.22E-09	0.757
12	76060283	77098899	452	4.71E-05	3.49E-09	0.425
12	77099081	78398357	624	3.60E-05	4.80E-09	0.603
12	78400884	78925445	243	4.74E-05	2.32E-09	0.325
12	78926240	80944201	511	9.51E-06	3.21E-09	0.867
12	80945774	83137872	721	9.92E-06	5.50E-09	0.894
12	83143660	83854442	270	-1.10E-06	2.40E-09	0.982
12	83856540	85014953	417	7.73E-06	2.23E-09	0.870
12	85016898	87296535	569	-1.45E-05	3.13E-09	0.796
12	87297692	87905878	166	-5.97E-06	1.08E-09	0.856
12	87909726	88848006	218	6.65E-06	1.38E-09	0.858
12	88851543	91827908	896	-2.97E-06	6.53E-09	0.971
12	91827930	93792073	767	-3.78E-05	6.64E-09	0.643
12	93792655	95717265	1015	-7.94E-05	9.03E-09	0.404
12	95717497	97439396	838	-4.55E-05	6.71E-09	0.579
12	99224060	99816442	205	-2.00E-05	1.80E-09	0.638
12	97439589	99220284	912	-6.02E-05	7.80E-09	0.496
12	99816842	101857521	803	7.34E-05	6.51E-09	0.363
12	101857796	102961329	417	-1.48E-04	2.94E-09	0.006
12	102961450	104011319	433	-1.67E-05	3.39E-09	0.774
12	105817813	106155478	204	1.34E-05	1.98E-09	0.763
12	106156001	107521770	612	5.71E-05	3.92E-09	0.362
12	107527013	108504440	424	1.35E-04	3.16E-09	0.016
12	104012667	105816376	1163	8.67E-05	8.23E-09	0.339
12	108506696	110111462	700	-8.01E-05	4.76E-09	0.246
12	110112932	113027651	567	-7.04E-05	4.76E-09	0.308
12	113030227	113877766	334	7.35E-05	2.33E-09	0.128
12	113885696	114882476	596	1.86E-04	1.24E-08	0.095
12	114883085	115326972	185	-1.73E-05	2.87E-09	0.747
12	117087697	117644288	184	-1.63E-05	1.43E-09	0.667
12	115329869	117087376	969	2.41E-05	9.15E-09	0.801
12	117644436	118888131	675	4.63E-05	6.48E-09	0.565
12	118888731	119301273	203	-7.79E-08	1.96E-09	0.999
12	119303044	120007172	394	-1.31E-04	5.06E-09	0.065

12	120008093	121720040	644	-6.04E-05	4.22E-09	0.352
12	121721831	124710880	880	6.19E-06	4.94E-09	0.930
12	124710996	125416169	363	-3.10E-05	5.22E-09	0.668
12	125417493	126461057	519	1.76E-05	5.06E-09	0.804
12	126463562	127382559	515	-8.88E-05	3.95E-09	0.158
12	127383648	128141516	402	1.93E-05	4.49E-09	0.773
12	128141610	128810520	447	1.09E-05	4.58E-09	0.872
12	128814615	129688509	573	5.84E-05	7.37E-09	0.496
12	129689639	130157939	346	-1.66E-05	3.94E-09	0.792
12	130158997	130859973	457	3.44E-05	4.98E-09	0.626
12	130861322	131407836	344	7.34E-05	3.92E-09	0.241
12	131408716	132012266	375	-7.65E-06	3.30E-09	0.894
12	132014206	132706778	276	-1.09E-05	2.32E-09	0.821
12	132807034	133841558	235	-1.46E-04	1.41E-08	0.219
13	19020095	20682304	395	-1.98E-05	2.01E-09	0.659
13	20684769	21688499	463	1.70E-05	3.23E-09	0.764
13	21688974	22209012	220	-5.04E-05	1.60E-09	0.208
13	23590702	23827019	193	-1.87E-05	2.46E-09	0.707
13	22211354	23590611	814	1.37E-05	8.97E-09	0.885
13	23827815	24477568	384	9.04E-05	2.91E-09	0.094
13	24477971	25210959	503	-4.62E-05	4.38E-09	0.485
13	25212359	25639070	222	-6.70E-05	1.68E-09	0.103
13	25640695	27282973	736	-2.08E-05	5.04E-09	0.769
13	27283115	28283059	561	-1.64E-06	4.86E-09	0.981
13	28284459	28876214	285	-2.73E-05	1.98E-09	0.539
13	28879332	29872965	451	-5.67E-05	3.45E-09	0.334
13	29873521	31379027	787	4.61E-05	6.83E-09	0.577
13	31384910	32983250	798	-3.99E-05	5.72E-09	0.598
13	32984197	34681078	710	-5.64E-05	3.92E-09	0.368
13	34683262	36042839	439	-2.93E-06	3.51E-09	0.961
13	36043978	37027089	549	2.59E-05	3.76E-09	0.672
13	38291011	38877678	287	-5.73E-06	1.73E-09	0.890
13	37029009	38290705	619	2.59E-05	3.73E-09	0.672
13	38887155	40179288	575	-3.06E-05	3.40E-09	0.600
13	40181792	41773356	639	-8.40E-05	4.37E-09	0.204
13	41774489	43099692	548	3.20E-05	3.34E-09	0.580
13	46493234	46866562	161	-1.17E-05	9.20E-10	0.699

13	43099889	44637493	786	1.23E-05	5.05E-09	0.863
13	44638337	46491101	755	-3.47E-05	5.22E-09	0.631
13	46869621	48439005	725	-7.73E-06	4.74E-09	0.911
13	48440893	49987145	462	1.64E-06	2.10E-09	0.971
13	49987663	51254196	497	6.69E-05	5.96E-09	0.386
13	51254712	53336663	702	-5.27E-05	4.02E-09	0.405
13	53337952	54681048	611	3.23E-05	4.21E-09	0.619
13	54681363	56433642	544	5.16E-05	2.82E-09	0.331
13	56434161	58247964	413	1.87E-05	1.79E-09	0.658
13	58248124	60085069	714	2.35E-05	4.67E-09	0.731
13	60087834	60854106	241	-4.62E-05	1.13E-09	0.170
13	60855021	61589772	348	8.54E-07	2.03E-09	0.985
13	61591949	62912481	486	-6.09E-06	2.82E-09	0.909
13	62913542	64201284	415	-1.18E-05	2.60E-09	0.816
13	64202212	65339575	366	-9.34E-06	1.82E-09	0.827
13	65340421	66197786	243	-7.06E-06	1.02E-09	0.825
13	66200191	67713633	592	-1.77E-05	3.40E-09	0.762
13	67714255	69561090	645	-3.53E-05	3.94E-09	0.574
13	69561894	70642326	429	-7.12E-06	3.03E-09	0.897
13	70643712	71095217	239	8.36E-06	1.70E-09	0.839
13	71095483	71759743	313	-3.61E-05	3.58E-09	0.546
13	71763633	72958993	473	1.15E-05	3.86E-09	0.853
13	72959394	73795534	346	5.48E-05	7.07E-09	0.514
13	73796728	75084163	678	-1.03E-06	5.51E-09	0.989
13	75084473	76504672	666	1.78E-04	1.01E-08	0.078
13	76505282	78030962	671	-1.41E-05	4.42E-09	0.832
13	78032193	78807439	318	-1.40E-05	1.81E-09	0.742
13	78807836	80252742	553	-2.46E-06	2.99E-09	0.964
13	82580994	84132765	494	3.91E-05	2.19E-09	0.404
13	80257200	82580321	856	5.29E-06	6.00E-09	0.946
13	84133082	85035423	269	1.02E-05	1.62E-09	0.801
13	85037313	85609664	262	1.69E-05	1.52E-09	0.664
13	85609760	87039195	528	3.91E-05	3.32E-09	0.498
13	87039360	88190520	314	3.96E-05	1.45E-09	0.298
13	88191656	89057861	239	-2.21E-05	1.37E-09	0.550
13	89059635	90177618	305	5.75E-06	1.34E-09	0.875
13	90179364	90867683	318	-2.01E-05	2.16E-09	0.665

13	90869873	91967960	405	-2.60E-05	2.60E-09	0.610
13	91970313	92912119	365	-1.26E-05	2.24E-09	0.790
13	92912423	93918144	412	7.50E-06	2.60E-09	0.883
13	93918533	94562549	322	4.82E-05	1.79E-09	0.254
13	94563601	95540024	474	5.87E-05	3.89E-09	0.346
13	97521207	98589842	367	1.36E-05	2.32E-09	0.778
13	95540709	97520182	918	2.91E-05	5.64E-09	0.699
13	98594975	100043997	776	-5.17E-05	5.09E-09	0.468
13	100044673	101392748	562	-5.17E-06	3.95E-09	0.934
13	101394726	102299385	584	5.16E-05	3.88E-09	0.407
13	102299498	103339911	558	5.94E-05	3.73E-09	0.330
13	103340536	104065375	505	1.49E-05	3.73E-09	0.808
13	104065534	104842764	367	7.49E-06	2.99E-09	0.891
13	104843186	105990071	584	7.55E-06	6.43E-09	0.925
13	105990812	106439396	245	-2.79E-05	1.99E-09	0.533
13	106442608	107785431	712	-2.43E-05	6.25E-09	0.758
13	107788025	108518444	439	4.57E-05	3.97E-09	0.469
13	108519987	109359792	437	3.56E-05	3.91E-09	0.569
13	109359834	110338482	539	1.62E-05	4.86E-09	0.816
13	110338913	111432821	729	4.04E-05	7.01E-09	0.630
13	113269127	115109853	670	2.89E-06	4.34E-09	0.965
13	111434814	113266084	884	6.88E-05	7.12E-09	0.415
14	20544512	20877114	188	1.19E-05	1.79E-09	0.778
14	20878890	21267077	308	-3.10E-05	4.24E-09	0.634
14	21271573	22378835	522	-4.97E-05	5.44E-09	0.501
14	22379132	22738105	229	5.17E-05	2.30E-09	0.281
14	22738161	23607730	506	1.53E-04	5.44E-09	0.038
14	23607866	24904154	515	4.70E-05	6.43E-09	0.558
14	24904240	26134815	654	-2.44E-06	4.72E-09	0.972
14	26135167	26679549	244	3.46E-06	2.23E-09	0.942
14	26680794	28466225	589	2.72E-05	4.49E-09	0.685
14	28466533	29196856	255	-8.75E-06	1.92E-09	0.841
14	29198059	32014712	797	4.55E-05	6.82E-09	0.581
14	32016504	33033695	420	2.28E-05	3.80E-09	0.712
14	34769883	36344816	497	2.88E-06	3.62E-09	0.962
14	36345767	36682357	143	5.77E-06	9.54E-10	0.852
14	33036081	34769821	1119	-2.13E-04	1.26E-08	0.058

14	36683516	38481516	689	2.55E-04	1.05E-08	0.013
14	38486187	39370633	361	-3.89E-05	2.82E-09	0.464
14	39370726	40336296	361	-2.18E-05	2.49E-09	0.662
14	40336990	41615299	437	1.68E-06	2.70E-09	0.974
14	41615331	43065019	495	2.41E-05	3.10E-09	0.665
14	43068948	44541699	471	-2.12E-05	4.17E-09	0.743
14	44542368	45247021	304	-2.49E-06	2.34E-09	0.959
14	45250418	46311209	221	-2.75E-06	1.06E-09	0.933
14	46312678	47456738	355	7.98E-06	2.04E-09	0.860
14	47457846	48351664	389	9.21E-06	2.84E-09	0.863
14	48352461	49001226	256	2.27E-05	2.23E-09	0.631
14	49004194	49955015	359	2.51E-05	2.47E-09	0.613
14	52078316	52761693	415	1.19E-05	3.60E-09	0.843
14	49955381	52077965	934	4.06E-05	7.56E-09	0.640
14	52762622	54162101	504	6.98E-05	6.20E-09	0.375
14	54162655	55043520	312	-1.07E-05	2.70E-09	0.837
14	55044905	56215082	474	-4.20E-05	3.24E-09	0.461
14	56215477	56949485	467	-2.58E-05	3.37E-09	0.657
14	56949644	58448681	677	-3.97E-05	5.77E-09	0.601
14	58449526	59135770	213	2.22E-05	1.47E-09	0.563
14	59135989	61649612	943	-5.64E-05	5.77E-09	0.458
14	63166807	63890839	304	-1.74E-05	2.11E-09	0.705
14	61651278	63164649	666	9.62E-05	5.83E-09	0.208
14	63897095	65218457	418	-5.54E-05	2.55E-09	0.272
14	65220775	66353557	440	3.96E-05	2.96E-09	0.467
14	66354477	67990648	525	2.97E-05	3.00E-09	0.587
14	67991028	69355606	630	3.83E-05	7.70E-09	0.662
14	71132759	72213258	357	-7.33E-06	2.03E-09	0.871
14	69356353	71131097	787	4.83E-05	5.97E-09	0.532
14	72214790	72888979	421	-2.75E-05	3.08E-09	0.620
14	72889376	74250730	550	-6.49E-05	4.57E-09	0.337
14	74252993	75747118	538	2.85E-05	2.98E-09	0.601
14	75747258	76583135	371	-9.34E-06	3.10E-09	0.867
14	76583775	77332160	386	-3.78E-05	3.80E-09	0.539
14	77332408	78421884	573	3.31E-05	4.43E-09	0.619
14	78422260	79026816	371	2.31E-06	2.97E-09	0.966
14	79027504	80998547	757	4.92E-05	5.83E-09	0.519

14	80999361	81787255	327	1.07E-05	2.28E-09	0.823
14	81787481	82459297	259	1.31E-05	2.20E-09	0.780
14	82461794	84087895	607	-3.08E-05	5.20E-09	0.669
14	84087979	85035235	352	9.81E-06	2.52E-09	0.845
14	85037974	86051751	438	-5.32E-05	3.94E-09	0.397
14	86053777	87334624	501	1.37E-05	3.74E-09	0.823
14	87335256	88641174	451	-2.24E-05	3.13E-09	0.689
14	88641335	90069965	638	-6.08E-05	4.96E-09	0.388
14	90075441	92098486	749	4.51E-05	6.56E-09	0.577
14	92099719	93378838	646	4.72E-05	5.70E-09	0.532
14	93379151	94420996	456	1.62E-05	3.99E-09	0.797
14	94421488	95347359	703	6.02E-05	6.11E-09	0.441
14	95347761	96250900	641	-2.31E-05	6.19E-09	0.769
14	96251418	97570260	662	8.11E-07	6.13E-09	0.992
14	97571437	98987472	651	-2.90E-06	5.48E-09	0.969
14	98989483	99980087	544	-2.70E-05	5.80E-09	0.723
14	99981105	101244250	481	-2.79E-05	3.83E-09	0.652
14	101244293	102339561	445	9.75E-05	5.51E-09	0.189
14	104761778	105440784	241	3.52E-05	2.29E-09	0.461
**14**	**102341650**	**104759919**	**714**	**3.68E-04**	**6.93E-09**	**0.000**
14	105443695	106552839	140	7.91E-06	1.02E-09	0.805
15	22362658	23288776	159	-4.80E-05	1.76E-09	0.252
15	23292175	24320772	300	-6.12E-05	3.28E-09	0.286
15	24321414	24976269	147	-6.89E-06	1.31E-09	0.849
15	25107905	25750941	208	-7.29E-06	1.85E-09	0.865
15	25752114	26185554	309	-1.04E-04	3.56E-09	0.080
15	26186113	26675188	259	8.09E-05	2.95E-09	0.136
15	26676258	27265824	242	-1.56E-05	2.66E-09	0.762
15	27266835	27819408	254	-2.57E-05	2.40E-09	0.600
15	27820352	28325439	264	2.58E-05	2.31E-09	0.591
15	28326536	29617158	227	2.22E-05	1.73E-09	0.593
15	29617517	31314317	411	4.18E-05	3.27E-09	0.465
15	31315570	32177326	354	1.93E-04	1.12E-08	0.068
15	32180607	33251727	348	6.43E-05	3.50E-09	0.277
15	33253484	33792116	378	-1.69E-04	5.58E-09	0.024
15	33793179	34273308	356	-3.74E-05	3.75E-09	0.541
15	34273346	34970673	233	-6.16E-06	1.92E-09	0.888

15	34971983	35941864	367	5.43E-05	2.68E-09	0.294
15	35943377	36424581	299	-1.58E-04	2.63E-09	0.002
15	36425448	36968033	285	-5.32E-06	3.02E-09	0.923
15	36968291	37930544	442	-1.19E-05	3.81E-09	0.847
15	37931874	38804104	337	-8.31E-05	2.79E-09	0.116
15	38805185	39518563	386	5.54E-06	3.38E-09	0.924
15	39519092	40382441	407	1.25E-06	3.35E-09	0.983
15	40383779	41211827	276	-1.33E-04	3.33E-09	0.021
15	41212265	42222063	300	2.39E-05	1.73E-09	0.566
15	42222390	43473907	378	-1.91E-05	2.15E-09	0.680
15	43474655	44988610	261	2.74E-05	1.32E-09	0.451
15	44992475	46292382	424	-6.10E-05	2.69E-09	0.240
15	46294385	47000090	290	2.77E-05	1.91E-09	0.526
15	47000857	47977097	382	-2.47E-05	2.49E-09	0.621
15	47979127	49075918	359	6.99E-05	2.23E-09	0.139
15	49078966	50004817	260	-1.26E-05	1.43E-09	0.738
15	51682775	52589995	273	2.05E-05	1.31E-09	0.572
15	50006184	51681073	717	5.08E-05	4.39E-09	0.443
15	52590615	53014125	170	8.49E-06	9.92E-10	0.788
15	53015101	53766542	361	-2.72E-05	3.78E-09	0.658
15	53767876	54336662	313	-3.67E-05	2.44E-09	0.457
15	54337243	55134743	392	1.81E-05	3.07E-09	0.744
15	55135408	55469163	140	3.61E-06	9.68E-10	0.908
15	55470187	55877891	151	2.14E-06	1.21E-09	0.951
15	55879453	56784345	319	-2.50E-05	2.20E-09	0.593
15	56784506	57637678	235	9.77E-06	1.26E-09	0.783
15	57638075	58438535	510	-7.38E-06	4.09E-09	0.908
15	59507391	60018662	244	3.83E-05	1.88E-09	0.377
15	58439935	59505191	502	1.38E-05	4.28E-09	0.833
15	60019136	60670149	285	-1.69E-05	2.36E-09	0.728
15	60670287	61803306	669	1.71E-05	6.89E-09	0.837
15	61803892	62735671	460	6.75E-05	3.42E-09	0.249
15	62737452	64335240	693	-6.50E-05	5.23E-09	0.369
15	65104668	66358474	354	2.31E-05	1.92E-09	0.599
15	66359486	66948204	296	-9.07E-05	2.47E-09	0.068
15	66948951	68640917	759	1.44E-05	4.97E-09	0.839
15	68642047	69936117	406	-3.15E-05	3.47E-09	0.592

15	69938058	70772135	451	1.53E-04	5.10E-09	0.032
15	70772414	71919256	463	-6.23E-05	3.50E-09	0.292
15	74453873	75516403	321	-1.70E-04	3.54E-09	0.004
15	71920065	74447996	707	-1.68E-05	4.21E-09	0.795
15	75520945	76397358	160	1.29E-05	9.90E-10	0.681
15	76397390	77910873	428	1.93E-06	2.21E-09	0.967
15	77912408	78637877	276	2.08E-05	2.61E-09	0.684
15	78640760	79259009	192	-1.99E-05	1.42E-09	0.597
15	79261077	79991408	436	-4.70E-05	3.36E-09	0.418
15	79992629	80635045	302	-8.75E-06	2.85E-09	0.870
15	80637749	81604871	477	-2.16E-05	4.41E-09	0.745
15	81607249	84062334	626	2.39E-06	4.55E-09	0.972
15	84063245	85517022	506	-1.15E-05	4.04E-09	0.856
15	85518062	86341812	361	-4.02E-05	2.07E-09	0.377
15	86342119	86650301	159	2.65E-05	1.32E-09	0.466
15	86650331	87113409	248	-8.62E-06	2.01E-09	0.848
15	87114392	87693557	314	-4.98E-05	3.05E-09	0.367
15	87693885	88032107	160	2.09E-05	1.34E-09	0.568
15	88032736	88742960	322	-5.15E-06	2.62E-09	0.920
15	88743108	89561223	336	4.53E-05	3.30E-09	0.430
15	89562631	90452882	361	1.92E-06	2.54E-09	0.970
15	90453183	91221307	299	1.27E-05	2.19E-09	0.787
15	91226586	91745246	252	-4.50E-05	2.60E-09	0.377
15	91749626	92164207	203	7.70E-05	2.18E-09	0.099
15	92165068	92667377	253	1.23E-06	2.55E-09	0.981
15	92669184	93590376	566	6.80E-05	5.19E-09	0.345
15	93591893	93908402	232	1.05E-04	3.64E-09	0.081
15	93909052	94547933	368	1.97E-06	3.62E-09	0.974
15	94548924	95576904	643	2.09E-05	6.97E-09	0.803
15	95577096	96497729	429	-1.07E-04	4.58E-09	0.115
15	96500358	97091511	333	-6.20E-05	3.49E-09	0.294
15	97092654	98496698	629	5.83E-05	7.31E-09	0.495
15	98497881	99128454	384	-1.05E-04	4.30E-09	0.110
15	100078196	100456641	152	-1.14E-05	1.31E-09	0.754
15	99128932	100077720	485	1.84E-05	4.73E-09	0.789
15	101408616	101918220	304	9.16E-05	2.80E-09	0.084
15	100457692	101403655	488	-9.53E-05	4.93E-09	0.175

15	101918830	102516259	287	3.67E-05	3.00E-09	0.503
16	60291	596605	208	-6.40E-06	2.00E-09	0.886
16	598838	945806	136	-2.95E-06	1.20E-09	0.932
16	946205	1547999	261	4.03E-06	2.46E-09	0.935
16	1551082	2293296	333	6.21E-05	2.45E-09	0.209
16	2294890	3960458	489	-5.67E-05	5.00E-09	0.423
16	3961770	4689471	275	9.91E-06	5.33E-09	0.892
16	4690187	5114499	203	-1.27E-05	1.60E-09	0.750
16	5115228	5854496	426	-8.48E-05	4.04E-09	0.183
16	5855169	6264138	318	7.63E-05	3.69E-09	0.209
16	7046430	7524343	442	1.35E-05	4.09E-09	0.833
16	6264139	7045506	647	-2.70E-05	6.09E-09	0.729
16	7525825	8236887	555	2.93E-05	5.55E-09	0.694
16	8237157	8572350	256	4.42E-05	2.89E-09	0.411
16	8572557	8960864	188	-1.20E-05	2.16E-09	0.796
16	8962907	9658861	335	-1.22E-04	3.42E-09	0.037
16	9659584	9997178	212	1.53E-05	1.63E-09	0.705
16	10002419	10430046	325	5.68E-06	2.72E-09	0.913
16	10431221	11493266	531	-1.92E-06	4.74E-09	0.978
16	11612436	12151685	190	1.66E-05	1.77E-09	0.693
16	12775897	13119544	141	-2.13E-05	1.36E-09	0.564
16	12151797	12775407	451	-2.20E-05	3.27E-09	0.700
16	13120367	13978116	506	-2.30E-06	4.26E-09	0.972
16	13979558	15885158	356	2.86E-05	2.71E-09	0.583
16	15885866	16220858	240	-2.60E-06	1.72E-09	0.950
16	16222763	17334499	271	6.45E-08	2.59E-09	0.999
16	17335780	18062164	300	3.66E-05	2.73E-09	0.484
16	18065944	20054371	507	-4.77E-05	3.86E-09	0.443
16	20055884	21235343	435	-5.32E-06	3.31E-09	0.926
16	21237217	23369433	478	-1.51E-05	4.07E-09	0.813
16	23371110	24308825	377	-2.85E-05	2.71E-09	0.585
16	24309108	25589623	527	-5.84E-05	4.18E-09	0.366
16	26683251	26943239	138	-2.65E-05	1.71E-09	0.521
16	25590147	26681997	620	-1.01E-04	6.31E-09	0.205
16	26944850	27445360	274	1.13E-04	3.40E-09	0.053
16	27446054	29023966	445	1.81E-05	2.53E-09	0.719
16	29694822	31380596	353	3.36E-06	2.29E-09	0.944

16	31384554	47981754	267	3.15E-05	1.77E-09	0.453
16	47982405	49006844	259	4.37E-05	2.28E-09	0.360
16	49007408	50694011	658	7.37E-05	6.06E-09	0.344
16	50698071	51700822	458	-4.40E-05	4.28E-09	0.501
16	51703888	53845487	830	8.07E-06	6.01E-09	0.917
16	53848448	54671719	496	-2.36E-04	1.00E-08	0.018
16	54673195	55505040	496	-1.31E-05	4.67E-09	0.848
16	56908045	57663112	346	-8.51E-05	3.83E-09	0.169
16	55506137	56906006	602	-1.30E-04	4.40E-09	0.050
16	57663619	58508278	391	-9.44E-05	7.77E-09	0.284
16	59889269	60744157	301	-2.03E-05	2.60E-09	0.690
16	58510034	59888170	534	2.36E-05	3.92E-09	0.706
16	60745208	61991704	352	-2.40E-05	2.47E-09	0.629
16	61993462	63395786	493	-8.89E-05	4.11E-09	0.165
16	64716851	65538017	376	-1.84E-05	2.43E-09	0.709
16	63396006	64714759	477	-1.87E-05	3.34E-09	0.746
16	68429894	70885818	618	-1.78E-05	3.51E-09	0.764
16	70887506	72915425	541	-1.06E-04	3.11E-09	0.058
16	65538484	68429077	907	-3.48E-05	5.92E-09	0.651
16	72915753	73542956	354	1.17E-05	4.21E-09	0.856
16	73543220	74724933	470	1.99E-05	3.78E-09	0.746
16	74730819	75517115	291	-3.06E-05	2.38E-09	0.531
16	75518129	76056374	169	-2.43E-05	1.68E-09	0.554
16	76058697	76987262	437	-1.92E-05	3.13E-09	0.731
16	76988130	77455781	322	2.65E-05	2.86E-09	0.619
16	77456244	77688760	199	-1.61E-05	1.63E-09	0.691
16	77689074	78446696	584	-4.51E-05	5.76E-09	0.553
16	78452348	78709939	217	-3.09E-06	2.53E-09	0.951
16	78710328	79368760	702	-4.39E-05	7.42E-09	0.610
16	79369779	80184442	562	-8.47E-05	5.16E-09	0.238
16	80184495	81209234	657	-1.09E-07	5.03E-09	0.999
16	81209631	82481987	784	8.44E-05	8.45E-09	0.359
16	82482458	83078581	572	1.46E-05	5.95E-09	0.850
16	83078959	83971446	825	-1.41E-05	8.15E-09	0.876
16	84646612	85146709	319	6.66E-05	3.63E-09	0.269
16	85146935	85663943	211	-1.33E-05	3.52E-09	0.822
16	83972415	84645588	626	-9.66E-05	7.97E-09	0.279

16	85664453	86221644	412	6.39E-05	4.98E-09	0.365
16	86222417	86745679	508	-2.33E-05	6.47E-09	0.773
16	86745955	87173290	254	4.42E-07	3.57E-09	0.994
16	87175053	88389287	532	-9.41E-06	5.22E-09	0.896
16	88439429	88964934	242	-4.90E-06	2.99E-09	0.929
16	88966667	89604610	217	2.82E-07	2.95E-09	0.996
16	89605495	90292767	240	-7.03E-06	2.11E-09	0.879
17	828	296442	133	-5.41E-06	3.14E-09	0.923
17	396660	820493	178	1.49E-04	4.15E-09	0.021
17	820511	1480393	260	-3.59E-05	4.86E-09	0.606
17	1481897	2613925	471	-3.07E-05	6.54E-09	0.704
17	2616352	3425788	387	4.47E-05	6.40E-09	0.576
17	3426133	4321755	384	-7.03E-06	5.29E-09	0.923
17	4322053	4650487	176	-7.88E-06	3.40E-09	0.892
17	4652156	5573766	370	-7.70E-06	4.74E-09	0.911
17	6572170	6975193	181	7.95E-05	2.99E-09	0.146
17	5574433	6570522	609	2.30E-05	9.63E-09	0.815
17	6976284	7723513	317	-1.59E-04	4.69E-09	0.020
17	7725660	8704551	399	7.81E-05	8.63E-09	0.401
17	8705612	9229793	253	-4.74E-05	7.53E-09	0.585
17	9233113	9714549	223	-1.42E-05	3.39E-09	0.808
17	9714995	10573151	522	-5.25E-05	7.89E-09	0.554
17	10573457	11667346	523	1.39E-05	7.80E-09	0.875
17	11668104	13000883	588	-2.99E-05	9.14E-09	0.754
17	13001700	13625425	351	-8.37E-05	5.89E-09	0.276
17	13626102	14209988	323	2.65E-05	4.96E-09	0.706
17	14210015	14499605	185	4.02E-05	3.04E-09	0.466
17	14500054	14859339	269	3.28E-05	3.89E-09	0.599
17	14860784	16319512	496	1.71E-04	7.09E-09	0.042
17	16321959	17292491	307	8.65E-05	4.30E-09	0.187
17	17299591	18446092	382	-3.20E-05	4.48E-09	0.632
17	18456679	19666001	259	-4.80E-06	3.30E-09	0.933
17	20679485	21284673	159	-1.99E-05	2.60E-09	0.697
17	21285166	25677370	210	5.62E-05	2.55E-09	0.266
17	25679294	26272308	203	-5.04E-06	2.47E-09	0.919
17	26273607	27884779	447	-4.89E-05	5.15E-09	0.495
17	27885675	29259899	375	-3.14E-06	4.50E-09	0.963

17	29262773	30878805	457	1.83E-05	7.89E-09	0.837
17	30879433	31989867	616	4.50E-06	8.56E-09	0.961
17	31990385	32677709	410	2.81E-05	5.47E-09	0.704
17	33616080	34393028	274	-3.22E-05	4.09E-09	0.615
17	32677947	33614452	507	-1.66E-05	7.12E-09	0.844
17	34393296	35725997	359	-3.92E-05	4.82E-09	0.573
17	35731855	37341235	493	3.49E-04	6.08E-08	0.157
17	37344586	38649203	331	-7.21E-05	5.34E-09	0.324
17	38652220	39657179	455	-8.47E-06	5.11E-09	0.906
17	39657337	40231221	205	-2.74E-05	2.40E-09	0.577
17	40232810	41769732	392	7.99E-06	4.46E-09	0.905
17	41771040	43317574	552	-5.87E-05	6.87E-09	0.479
17	44863413	45874715	302	3.17E-05	4.65E-09	0.642
17	45876022	46828302	408	-1.11E-04	5.88E-09	0.148
17	46828412	48027295	402	-3.37E-05	1.36E-08	0.773
17	48027688	48832008	360	-4.20E-05	6.06E-09	0.589
17	48832437	49892805	428	-3.28E-05	5.22E-09	0.650
17	49894599	51282380	527	1.09E-05	5.81E-09	0.886
17	52863321	53338298	227	1.88E-05	2.98E-09	0.730
17	51284441	52861905	537	-4.31E-05	6.84E-09	0.602
17	53346718	54164464	384	-4.53E-05	4.73E-09	0.510
17	54164935	55278525	429	-1.46E-05	5.99E-09	0.850
17	57487538	59307733	302	-1.33E-05	5.06E-09	0.852
17	60885287	62035655	261	-2.53E-05	2.92E-09	0.640
17	59309085	60883279	464	-4.01E-05	5.95E-09	0.603
17	55279508	57486793	880	1.37E-04	1.25E-08	0.219
17	62037685	64434272	753	-4.77E-05	9.77E-09	0.629
17	64434524	65013985	328	-9.84E-06	4.75E-09	0.887
17	65016683	66315468	382	-1.92E-04	6.43E-09	0.017
17	66318466	66903293	310	4.65E-05	4.32E-09	0.479
17	66906217	68199133	460	6.18E-05	5.87E-09	0.420
17	68199783	68875761	304	-1.52E-05	4.01E-09	0.810
17	68877746	69242135	206	2.61E-04	2.03E-08	0.067
17	69242932	70147289	397	-5.88E-05	8.52E-09	0.524
17	70148198	70947580	477	3.15E-05	7.99E-09	0.724
17	70948535	71825523	480	1.43E-04	7.55E-09	0.099
17	71829519	72740027	438	-1.86E-05	6.75E-09	0.821

17	72742484	74016834	403	-5.39E-05	5.10E-09	0.450
17	74018640	74909405	368	5.17E-05	5.26E-09	0.475
17	74910943	75471247	314	7.55E-05	5.45E-09	0.306
17	75476450	75842792	220	1.90E-05	4.14E-09	0.768
17	75844316	76445231	219	3.84E-05	3.87E-09	0.536
17	76446689	77093768	322	1.34E-05	5.19E-09	0.852
17	77094717	77468875	306	-2.16E-05	5.96E-09	0.780
17	77471383	78780080	510	1.14E-05	7.59E-09	0.895
17	78780686	79683249	363	-7.98E-05	5.39E-09	0.277
17	79683698	80969550	416	3.00E-05	5.11E-09	0.675
18	349293	906343	394	-2.61E-05	3.88E-09	0.675
18	1940863	2206099	166	-3.03E-05	2.01E-09	0.499
18	906673	1940581	435	-2.37E-05	3.72E-09	0.697
18	2206891	2889826	315	1.83E-05	2.86E-09	0.731
18	2891847	3357120	219	-4.90E-05	2.42E-09	0.319
18	3359237	3861997	218	-1.69E-05	3.24E-09	0.767
18	3862461	4597021	432	3.28E-05	4.58E-09	0.628
18	4601573	5197395	229	5.25E-05	2.29E-09	0.272
18	5198562	5852964	326	-4.74E-05	2.97E-09	0.385
18	5855598	6630394	360	-4.94E-06	3.47E-09	0.933
18	6630840	7373590	413	4.11E-05	4.07E-09	0.519
18	7374066	8440357	509	-1.55E-05	3.99E-09	0.806
18	8440558	9414901	548	1.23E-04	5.28E-09	0.091
18	10465005	11028008	322	6.11E-05	2.39E-09	0.211
18	9415389	10462537	665	3.63E-05	7.49E-09	0.675
18	11028129	11529011	226	1.08E-05	2.19E-09	0.818
18	11530996	12737037	467	-8.66E-06	3.30E-09	0.880
18	12737582	14358413	604	-8.21E-06	4.36E-09	0.901
18	18527782	21622994	744	2.11E-05	6.38E-09	0.791
18	21623416	22745505	572	3.74E-05	4.86E-09	0.592
18	22746547	24088696	488	4.69E-06	3.56E-09	0.937
18	24088831	24971742	490	-4.65E-05	3.04E-09	0.399
18	26874132	28037427	397	-2.66E-06	2.42E-09	0.957
18	28037840	28560707	235	2.31E-06	1.82E-09	0.957
18	24972728	26873513	697	-1.10E-04	4.55E-09	0.103
18	28561634	29563172	480	1.87E-05	3.38E-09	0.748
18	31777336	32456417	204	-3.62E-05	1.16E-09	0.287

18	29564408	31774120	674	-3.33E-05	3.78E-09	0.588
18	32457437	33791804	485	-4.85E-05	3.10E-09	0.384
18	35073616	35837704	322	-8.78E-06	2.06E-09	0.847
18	33792671	35072855	572	-6.04E-06	3.58E-09	0.920
18	35838871	36364691	241	1.90E-05	2.36E-09	0.696
18	36365021	37682827	411	-1.28E-05	2.49E-09	0.797
18	37683967	38766853	525	7.14E-05	4.39E-09	0.281
18	38767938	39891215	366	-2.42E-05	2.27E-09	0.612
18	42606091	43295247	413	3.80E-05	3.05E-09	0.491
18	43295347	43881224	239	1.05E-05	1.34E-09	0.775
18	39892281	42606024	955	1.00E-04	5.95E-09	0.193
18	45314528	46208355	424	8.13E-05	3.98E-09	0.197
18	43881706	45313390	680	-7.66E-06	4.49E-09	0.909
18	46209339	47262925	483	3.96E-05	3.62E-09	0.510
18	47263681	47814821	330	2.74E-05	2.08E-09	0.549
18	47816777	48784027	395	-4.27E-06	2.51E-09	0.932
18	48784953	50151479	574	4.14E-05	4.46E-09	0.535
18	50152229	51059341	375	-4.30E-06	2.26E-09	0.928
18	51059850	51952586	345	1.32E-06	5.23E-09	0.985
18	51953945	52707502	198	-5.32E-05	1.80E-09	0.211
18	52709949	53763665	408	-8.20E-05	3.59E-09	0.171
18	53764157	55089715	500	-3.17E-05	3.30E-09	0.581
18	55090919	55808073	393	3.93E-05	3.53E-09	0.509
18	55808692	56752598	490	3.67E-06	3.97E-09	0.953
18	56754050	57504565	479	6.87E-05	3.89E-09	0.271
18	57505073	58816387	484	5.38E-07	3.21E-09	0.992
18	58817565	59585320	377	5.33E-05	3.30E-09	0.353
18	59585959	60822264	549	-1.63E-05	3.84E-09	0.793
18	60823554	61439643	276	9.68E-06	1.87E-09	0.823
18	61441801	62075853	293	-1.67E-05	1.70E-09	0.685
18	62078305	63110788	403	-2.06E-05	2.85E-09	0.700
18	63111628	64250279	397	-2.21E-05	2.44E-09	0.655
18	64251305	65830031	735	4.37E-05	5.78E-09	0.565
18	65830606	66404071	323	4.94E-05	2.22E-09	0.294
18	66404118	67569547	516	-5.68E-06	4.76E-09	0.934
18	67574191	69038112	511	-3.33E-05	4.52E-09	0.620
18	69039235	70094707	484	-8.12E-05	4.21E-09	0.211

18	70094975	70717286	329	-1.87E-05	2.77E-09	0.722
18	70719886	71243399	284	2.83E-05	2.56E-09	0.575
18	71244692	71892202	389	-5.10E-05	3.95E-09	0.417
18	71892443	72519795	346	2.64E-05	2.61E-09	0.605
18	72521276	73897594	733	-5.95E-05	5.69E-09	0.430
18	73898846	75028501	623	1.18E-05	6.52E-09	0.884
18	75029584	75473982	288	7.06E-05	2.61E-09	0.167
18	75474664	75975253	233	-3.10E-05	2.27E-09	0.515
18	75976567	77151520	503	-1.17E-04	5.77E-09	0.125
18	77151867	78017074	335	-6.37E-05	2.63E-09	0.214
19	157892	757412	227	-2.46E-05	4.24E-09	0.705
19	759261	1089241	159	-1.10E-05	2.92E-09	0.839
19	1090803	1920712	264	1.08E-04	5.46E-09	0.145
19	1922476	2815372	342	-3.46E-05	5.23E-09	0.632
19	2816339	3301333	213	2.64E-05	3.50E-09	0.656
19	3304779	4347573	350	3.33E-05	7.72E-09	0.704
19	4349416	5191547	336	1.11E-05	4.52E-09	0.869
19	5192372	5766090	218	-4.45E-05	4.03E-09	0.484
19	5767585	6612779	264	-3.33E-05	3.90E-09	0.594
19	6614637	7309688	316	5.72E-06	5.27E-09	0.937
19	7312727	8684707	486	5.16E-06	7.83E-09	0.953
19	9013231	10029966	390	-3.16E-05	4.29E-09	0.630
19	10030690	11279257	438	5.11E-05	1.53E-08	0.679
19	11280183	12497411	332	-6.85E-05	4.01E-09	0.280
19	12500149	13903388	408	-3.94E-05	5.48E-09	0.594
19	15553878	16251518	444	2.24E-05	5.12E-09	0.754
19	13906229	15549939	595	-6.85E-05	8.29E-09	0.452
19	17944900	18506666	242	-3.31E-05	3.65E-09	0.584
19	16252072	17942927	622	-7.57E-05	8.91E-09	0.423
19	19873640	20520392	140	-1.94E-05	1.67E-09	0.636
19	18506815	19873269	429	3.12E-05	4.88E-09	0.655
19	21379657	22036399	158	-5.61E-06	1.71E-09	0.892
19	22037866	22803640	245	-2.68E-05	2.68E-09	0.605
19	22805247	24059424	282	7.63E-06	2.82E-09	0.886
19	24059507	28555771	270	2.30E-06	3.00E-09	0.966
19	28556284	28917565	189	-1.66E-05	2.08E-09	0.716
19	29318443	30113252	466	-2.07E-05	5.65E-09	0.783

19	30113991	30858084	259	1.79E-05	3.84E-09	0.773
19	30858965	32136133	462	-1.34E-04	5.73E-09	0.078
19	33764103	34067891	139	3.14E-05	2.00E-09	0.483
19	32145233	33763475	600	2.17E-05	7.28E-09	0.799
19	34068435	35331436	448	1.30E-05	5.96E-09	0.866
19	35333176	36045772	365	3.02E-06	5.11E-09	0.966
19	36047098	37454119	411	1.77E-05	4.63E-09	0.795
19	37455512	39370456	574	-1.85E-05	1.14E-08	0.863
19	39371014	39757572	147	2.32E-05	1.88E-09	0.592
19	41442597	41841098	164	7.06E-06	1.87E-09	0.870
19	41843461	43954915	365	-1.20E-04	8.61E-09	0.196
19	43956095	44880657	396	1.61E-05	4.54E-09	0.811
19	39757926	41440863	613	-1.17E-04	7.52E-09	0.177
19	44882157	45255679	171	2.34E-05	2.89E-09	0.663
19	45259002	46433733	399	-9.81E-05	7.83E-09	0.268
19	46433891	47333204	330	-2.94E-05	5.29E-09	0.686
19	47333497	48509893	371	-7.11E-05	4.85E-09	0.307
19	48697961	49485758	337	-1.48E-04	5.00E-09	0.037
19	49491035	49923166	159	8.38E-08	1.83E-09	0.998
19	49925208	50774141	253	-5.47E-05	3.47E-09	0.352
19	50774581	51331589	225	2.33E-05	3.67E-09	0.701
19	52217405	52604625	221	-1.50E-05	2.70E-09	0.773
19	52606936	53107086	222	-7.83E-05	3.71E-09	0.199
19	51332061	52216563	534	-2.49E-05	3.51E-08	0.894
19	53107937	53537923	157	2.69E-05	2.89E-09	0.616
19	53538505	53899797	201	-2.22E-05	3.13E-09	0.692
19	54099626	54702450	223	1.11E-05	5.49E-09	0.881
19	54704266	55174498	210	4.06E-05	3.87E-09	0.514
19	55175009	55791380	259	-5.78E-05	4.29E-09	0.378
19	55794266	56364684	248	-1.88E-05	5.51E-09	0.800
19	56365323	56898342	291	-4.00E-05	5.11E-09	0.576
19	56898853	57950377	491	3.74E-05	7.14E-09	0.658
19	57952035	58531715	259	-2.06E-05	2.67E-09	0.691
19	58532756	59118784	238	-1.67E-05	2.83E-09	0.754
20	246392	887800	377	-3.54E-05	6.38E-09	0.658
20	888523	1445821	369	-1.45E-05	4.64E-09	0.831
20	1446580	1897442	256	-1.47E-05	2.56E-09	0.772

20	3083151	3655085	235	4.04E-05	2.18E-09	0.387
20	3655943	4051325	176	-3.24E-05	2.19E-09	0.489
20	1899473	3081680	692	2.57E-05	6.65E-09	0.753
20	4053020	4701211	402	-1.97E-05	5.22E-09	0.785
20	4701706	5304961	360	1.31E-05	3.76E-09	0.831
20	5305400	6636139	709	-1.68E-04	7.35E-09	0.050
20	6637234	7065163	285	1.56E-05	3.35E-09	0.788
20	7066818	8600105	764	-7.26E-05	6.32E-09	0.361
20	8601114	8881586	198	2.21E-05	1.45E-09	0.561
20	8884009	9745450	471	-4.41E-05	4.19E-09	0.495
20	11014704	11534388	291	4.62E-05	2.28E-09	0.333
20	9746345	11013570	694	-1.30E-05	7.30E-09	0.879
20	11534488	12153352	274	3.88E-05	2.30E-09	0.418
20	12153396	13199755	619	-1.91E-04	5.67E-09	0.011
20	13201177	13666198	193	2.49E-05	1.81E-09	0.558
20	13670311	14929217	486	1.90E-05	4.02E-09	0.764
20	15148231	16150464	705	1.05E-04	7.13E-09	0.215
20	17449713	17905102	299	-2.12E-05	3.43E-09	0.718
20	17906490	18896415	440	-2.85E-05	3.43E-09	0.626
20	18896895	19707457	462	-4.15E-05	4.03E-09	0.513
20	16151566	17449361	878	2.98E-05	7.97E-09	0.738
20	19708351	21676556	819	-3.80E-05	7.18E-09	0.654
20	21679930	22779512	406	3.11E-06	2.61E-09	0.951
20	22779865	23568490	399	1.46E-05	3.06E-09	0.792
20	23568495	24053655	219	2.61E-05	1.69E-09	0.526
20	24054931	25019099	416	5.95E-06	3.05E-09	0.914
20	26319535	30570889	234	3.63E-05	1.49E-09	0.347
20	25020047	26319418	309	-4.36E-05	2.10E-09	0.342
20	30574455	31613985	334	-5.62E-05	2.33E-09	0.244
20	33885155	34891416	221	2.22E-05	1.29E-09	0.538
20	34892880	35892530	205	-3.29E-06	1.57E-09	0.934
20	31615242	33883015	705	-5.82E-05	4.18E-09	0.368
20	35899774	36904375	395	-7.37E-05	2.87E-09	0.169
20	36905894	37875045	354	2.58E-06	2.69E-09	0.960
20	37875309	38431408	230	5.81E-09	1.52E-09	1.000
20	38435302	39453533	470	-7.72E-06	3.87E-09	0.901
20	39455778	40584825	395	-1.64E-05	3.30E-09	0.775

20	40585126	41194048	393	-1.01E-06	3.73E-09	0.987
20	41195111	42188284	488	-1.87E-05	4.80E-09	0.788
20	42188914	43257406	508	2.28E-05	4.52E-09	0.734
20	44960997	45649369	359	4.63E-05	3.28E-09	0.419
20	45650139	46540102	374	8.29E-05	4.03E-09	0.191
20	43259306	44960236	711	-9.12E-05	5.45E-09	0.216
20	48081983	48655016	227	-1.11E-05	4.17E-09	0.863
20	48658113	49385779	312	-1.06E-05	3.29E-09	0.853
20	46540587	48080850	770	-5.81E-05	6.34E-09	0.466
20	49387520	49945761	256	-3.61E-05	3.69E-09	0.553
20	49948016	50633472	360	-1.52E-05	3.74E-09	0.804
20	50635347	51389135	319	-3.07E-05	2.52E-09	0.541
20	51389670	51884594	263	-3.05E-05	2.77E-09	0.562
20	51885269	52525657	292	2.94E-05	5.53E-09	0.693
20	52526840	53406846	485	5.17E-05	4.80E-09	0.456
20	53408035	54321635	409	4.75E-05	3.45E-09	0.419
20	54321811	55431836	547	4.59E-05	5.08E-09	0.519
20	56216001	56679942	290	-6.77E-06	3.35E-09	0.907
20	55434683	56214465	499	9.10E-05	5.99E-09	0.240
20	56679960	57319010	352	-5.57E-05	3.49E-09	0.345
20	57320545	58295563	478	4.26E-05	5.70E-09	0.573
20	59361382	59892000	354	3.70E-05	4.13E-09	0.565
20	58296350	59361064	553	-3.55E-05	5.08E-09	0.619
20	59892231	61091395	657	-9.31E-05	1.32E-08	0.417
20	61301855	62119318	401	-2.41E-05	4.82E-09	0.729
20	62119875	62962870	340	-2.14E-04	1.07E-08	0.039
21	15451950	16139409	423	-9.16E-05	3.93E-09	0.144
21	18457828	19151566	246	-1.22E-04	3.04E-09	0.027
21	16140244	17301757	441	8.91E-05	3.90E-09	0.153
21	17305471	18457656	408	-3.74E-05	4.87E-09	0.592
21	19151940	20198061	542	-3.10E-05	4.86E-09	0.656
21	21022840	21905609	384	1.32E-05	3.95E-09	0.834
21	20199390	21022168	395	-3.03E-05	3.49E-09	0.608
21	24695869	25083563	190	-2.95E-06	1.74E-09	0.944
21	23270321	24695405	541	-3.41E-05	4.74E-09	0.621
21	21905658	23268216	670	1.96E-05	5.86E-09	0.798
21	25899516	26465774	241	-9.08E-06	2.15E-09	0.845

21	25090174	25898082	385	-4.89E-05	3.18E-09	0.386
21	26467796	27776736	496	2.57E-05	4.26E-09	0.694
21	27777181	28205789	289	2.17E-05	2.82E-09	0.682
21	28206883	28705495	309	3.20E-05	3.15E-09	0.568
21	28705732	29515396	352	1.07E-07	3.45E-09	0.999
21	30845873	32211778	531	4.29E-05	3.91E-09	0.492
21	29515884	30844467	550	-4.03E-05	3.66E-09	0.506
21	33273162	33988973	358	-3.10E-05	3.16E-09	0.581
21	32213678	33270766	453	-1.36E-05	4.23E-09	0.835
21	33991368	35219895	507	-1.68E-04	1.08E-08	0.106
21	35220001	36188046	457	-8.84E-05	1.78E-08	0.508
21	37870518	38429082	265	-1.36E-06	2.23E-09	0.977
21	36190040	37303320	549	1.41E-05	4.78E-09	0.838
21	37305159	37868817	300	4.88E-05	2.42E-09	0.321
21	38429389	39248065	402	-2.09E-05	2.89E-09	0.697
21	39250321	40115483	422	4.53E-06	4.13E-09	0.944
21	40116044	41095241	608	-1.07E-04	4.97E-09	0.129
21	41260363	41705756	344	-2.11E-05	3.41E-09	0.718
21	41707453	42543663	491	6.96E-05	4.61E-09	0.305
21	42545250	43342046	530	-2.01E-04	1.05E-08	0.050
21	44690856	45598522	356	-8.85E-08	2.79E-09	0.999
21	45603905	46174491	279	3.95E-06	3.37E-09	0.946
21	46174981	46740019	297	4.56E-05	2.55E-09	0.367
21	46741287	47491264	334	-2.41E-05	2.91E-09	0.655
21	47491975	48119635	293	-3.01E-05	2.21E-09	0.523
21	43343190	44632513	845	-2.03E-05	8.37E-09	0.824
22	17187276	17569017	168	-2.33E-05	1.70E-09	0.571
22	18333902	19138442	298	-1.34E-05	2.82E-09	0.800
22	17569439	18333467	393	5.86E-05	4.90E-09	0.402
22	19139368	20000428	424	-2.55E-05	4.63E-09	0.707
22	20001006	20952650	271	-1.03E-04	3.68E-09	0.090
22	20952972	21863717	295	-9.15E-06	2.33E-09	0.850
22	21866569	23015302	368	-5.91E-06	3.33E-09	0.918
22	23016570	23691564	208	2.00E-05	1.76E-09	0.634
22	23697301	24370194	306	1.27E-06	3.47E-09	0.983
22	24375019	25416292	358	4.76E-06	2.94E-09	0.930
22	25419524	26113333	226	3.38E-06	2.27E-09	0.943

22	26123092	26735929	447	-7.75E-05	4.76E-09	0.261
22	26736812	27294543	387	-1.89E-05	4.25E-09	0.772
22	27295234	27653424	353	-1.36E-05	4.53E-09	0.840
22	29456360	30604102	391	1.51E-05	2.85E-09	0.778
22	30605601	31534127	380	-2.46E-05	3.40E-09	0.673
22	31535872	32324140	238	-5.00E-06	1.49E-09	0.897
22	32324692	32856999	277	-1.78E-05	2.47E-09	0.720
22	27654838	29454477	731	-2.04E-04	7.81E-09	0.021
22	32857285	33511391	411	-3.73E-05	4.25E-09	0.567
22	33512106	33896495	178	6.29E-06	2.07E-09	0.890
22	35865218	36131498	134	-4.26E-05	1.59E-09	0.286
22	34983275	35862655	468	7.86E-06	3.26E-09	0.891
22	36132263	36922822	314	3.08E-05	3.85E-09	0.620
22	33898114	34982457	618	-5.74E-06	5.21E-09	0.937
22	36923144	37558356	398	2.38E-06	4.32E-09	0.971
22	38473398	39302783	269	1.65E-05	2.08E-09	0.717
22	37560592	38467784	418	-3.47E-05	3.87E-09	0.577
22	39304509	40092866	320	3.74E-05	2.88E-09	0.487
22	40095174	41405753	318	-3.88E-05	3.41E-09	0.507
22	43187900	43645335	232	-7.89E-05	1.76E-08	0.552
22	41406919	42870441	427	1.95E-05	4.12E-09	0.761
22	43646241	44317084	380	-1.41E-05	3.16E-09	0.803
22	44317416	44818986	414	1.28E-04	4.93E-09	0.069
22	45444397	45895495	269	3.58E-06	2.79E-09	0.946
22	44820796	45443845	418	-4.41E-05	5.41E-09	0.548
22	45895856	46512413	261	1.16E-05	3.10E-09	0.835
22	46513376	47106715	271	-6.70E-05	2.96E-09	0.218
22	47106800	47937655	434	-3.26E-06	3.92E-09	0.958
22	47937891	48479731	373	-4.88E-05	3.89E-09	0.434
22	49082979	49521080	313	-4.43E-05	4.37E-09	0.503
22	49522184	49751418	246	-1.82E-05	3.30E-09	0.752
22	48481280	49081753	463	1.09E-05	5.92E-09	0.887
22	49751512	50488153	335	3.34E-05	3.26E-09	0.558
22	50488995	51237713	281	3.49E-05	3.01E-09	0.525
**Prostate cancer and COVID-19 critical illness**						
1	1103150	2320702	355	-3.51E-05	7.04E-09	0.676
1	2321099	3065568	234	-4.75E-05	8.73E-09	0.611

1	3066761	3679461	289	-2.03E-04	1.32E-08	0.078
1	3679775	4749076	631	-1.59E-04	1.51E-08	0.196
1	4750013	5000086	168	4.29E-06	4.12E-09	0.947
1	5000970	5471517	301	-1.41E-05	7.28E-09	0.869
1	5472626	6133700	338	2.18E-05	7.47E-09	0.801
1	6134990	7245669	446	4.12E-05	8.17E-09	0.648
1	7245922	7604778	205	-3.49E-06	4.56E-09	0.959
1	7604831	9067142	512	1.09E-04	9.89E-09	0.272
1	9067673	9959051	318	1.77E-04	1.39E-08	0.134
1	9959748	11774956	583	9.76E-05	1.04E-08	0.339
1	11776196	12147753	192	-3.30E-05	3.35E-09	0.569
1	12150385	14625524	723	9.10E-05	1.75E-08	0.492
1	14626896	15423481	421	-7.63E-05	1.12E-08	0.470
1	15424441	17123862	613	-1.06E-04	1.32E-08	0.354
1	17176635	17872659	333	-1.59E-05	7.39E-09	0.853
1	17874843	18484945	340	-2.06E-05	7.48E-09	0.812
1	18486272	20022063	798	-1.27E-04	1.77E-08	0.340
1	20023833	20763899	370	3.68E-06	1.02E-08	0.971
1	20765245	22634131	796	-1.09E-04	1.62E-08	0.393
1	22636103	23082667	208	-1.15E-04	5.64E-09	0.125
1	23085227	24527501	489	-3.25E-05	8.46E-09	0.724
1	24528210	25305673	316	3.14E-05	7.23E-09	0.712
1	25305783	26976424	610	-7.80E-05	1.49E-08	0.523
1	26981479	28160199	204	1.88E-05	3.17E-09	0.738
1	28168872	29181525	242	-9.06E-05	3.51E-09	0.127
1	29184254	30792409	733	-8.75E-05	1.37E-08	0.454
1	30792984	32042421	610	2.71E-05	9.13E-09	0.776
1	32042540	34016219	485	-4.45E-06	9.10E-09	0.963
1	34017349	34797348	442	-4.89E-05	9.06E-09	0.607
1	34798014	35364965	274	-2.44E-05	6.81E-09	0.767
1	35366056	38196841	760	9.76E-05	1.38E-08	0.407
1	38459210	39535259	434	-6.89E-05	1.05E-08	0.501
1	39537291	40933221	445	2.87E-04	7.77E-09	0.001
1	40934284	42166782	487	-1.64E-05	8.57E-09	0.859
1	42168539	43365094	396	-7.93E-05	5.91E-09	0.302
1	43365565	44482312	463	-1.65E-04	6.90E-09	0.046
1	44483704	46895641	764	8.87E-05	9.53E-09	0.363

1	46897698	47999344	385	2.31E-05	5.70E-09	0.760
1	48000156	50640662	789	3.39E-05	1.20E-08	0.757
1	50641944	53116453	445	9.50E-05	6.58E-09	0.242
1	53117066	54197141	419	1.13E-05	7.19E-09	0.894
1	54197688	55012825	295	1.53E-05	6.59E-09	0.850
1	55012881	55479198	250	3.07E-05	5.70E-09	0.685
1	55479967	56413577	485	1.93E-04	1.43E-08	0.107
1	56414730	57585670	599	4.89E-05	1.09E-08	0.639
1	57585980	58535140	555	-6.22E-05	9.02E-09	0.513
1	58536417	59306823	412	6.41E-05	7.05E-09	0.445
1	59307290	59736866	187	9.84E-06	4.09E-09	0.878
1	59739179	61257170	537	-9.05E-05	9.69E-09	0.358
1	61258378	62107806	382	2.94E-04	1.22E-08	0.008
1	62108681	63376404	518	1.66E-04	9.33E-09	0.086
1	63379031	65008206	795	3.69E-05	1.54E-08	0.766
1	65010679	66773349	699	-7.61E-05	1.23E-08	0.493
1	66774350	67760291	536	4.87E-05	8.72E-09	0.602
1	67761365	69441414	724	-1.05E-04	1.43E-08	0.381
1	69441920	70486660	328	8.57E-05	5.96E-09	0.267
1	70490925	71818029	501	4.80E-05	8.91E-09	0.611
1	71822765	74326378	642	3.42E-05	9.23E-09	0.722
1	74326484	75992462	562	-3.52E-05	8.74E-09	0.706
1	75992778	76476360	183	-2.85E-05	2.50E-09	0.569
1	76477207	77139859	372	-6.84E-05	7.74E-09	0.436
1	77141039	79111862	685	2.08E-05	1.21E-08	0.850
1	79113455	80341320	482	3.12E-06	7.91E-09	0.972
1	80341732	81056946	241	-3.77E-06	4.16E-09	0.953
1	81058295	82883310	839	-2.41E-04	1.69E-08	0.064
1	82884721	83990906	440	1.92E-05	7.58E-09	0.826
1	83991748	84728665	328	-1.70E-05	5.14E-09	0.813
1	84729134	85940249	595	-1.27E-05	1.18E-08	0.907
1	85940875	86549498	268	1.27E-05	4.39E-09	0.848
1	86549569	87652502	416	-3.11E-05	7.34E-09	0.717
1	87654606	89447978	631	5.45E-06	1.59E-08	0.965
1	89451664	91866856	883	-1.06E-04	1.58E-08	0.397
1	91867046	93351477	560	1.50E-05	8.99E-09	0.875
1	93352491	94451007	318	-3.55E-05	4.38E-09	0.592

1	94452062	95199048	406	-5.63E-06	7.63E-09	0.949
1	95199771	96149941	435	5.99E-06	8.37E-09	0.948
1	96150318	97457893	467	-3.58E-05	7.26E-09	0.674
1	97458722	98562260	516	-1.00E-04	6.88E-09	0.226
1	98564736	100064599	575	1.56E-04	1.09E-08	0.136
1	100065413	101682573	614	1.78E-04	8.73E-09	0.056
1	101683165	102039858	221	-5.81E-05	4.35E-09	0.378
1	102040268	102893372	341	-2.09E-06	6.18E-09	0.979
1	102894567	104369301	287	5.86E-05	5.13E-09	0.413
1	104373157	105192916	269	-1.14E-05	5.00E-09	0.872
1	105193022	106641877	398	-2.36E-05	6.83E-09	0.775
1	106642705	107432265	291	-8.59E-05	4.97E-09	0.223
1	107432576	109075191	691	7.93E-05	1.44E-08	0.508
1	109075561	110301260	462	3.42E-05	7.95E-09	0.702
1	110302505	111738116	679	-3.84E-05	1.55E-08	0.758
1	111739749	112560278	566	-6.12E-05	1.17E-08	0.572
1	112561693	114451307	681	-7.28E-05	9.74E-09	0.461
1	114451386	115878242	636	-8.60E-05	1.15E-08	0.423
1	115879821	116884431	398	4.38E-05	7.35E-09	0.610
1	116884519	118837474	648	8.73E-05	1.20E-08	0.425
1	118837888	119792751	365	1.13E-05	5.86E-09	0.882
1	119793605	120347239	234	3.81E-05	5.82E-09	0.618
1	144998563	147347713	448	2.44E-05	7.46E-09	0.778
1	147348339	151830844	597	-3.95E-05	2.13E-08	0.787
1	151832901	152535021	216	6.94E-05	2.79E-09	0.189
1	152535586	153163377	278	1.91E-05	3.40E-09	0.744
1	153165450	154544651	506	1.18E-04	8.67E-09	0.205
1	154547292	156334116	549	6.58E-05	7.76E-08	0.813
1	156335111	157154186	313	2.02E-04	1.27E-08	0.073
1	157154324	158867538	819	3.07E-05	1.54E-08	0.805
1	158870085	160648549	764	-1.30E-04	1.64E-08	0.311
1	160649926	161973976	510	8.34E-05	9.30E-09	0.387
1	161974584	162995355	563	-2.87E-05	1.06E-08	0.780
1	163000394	164410254	676	-1.42E-04	1.08E-08	0.172
1	164412847	165190876	436	-5.64E-05	1.05E-08	0.582
1	165191702	166363258	604	-1.29E-05	1.21E-08	0.906
1	166364063	167158927	484	-1.35E-05	8.09E-09	0.880

1	167159608	168168773	462	9.84E-05	9.01E-09	0.300
1	168169452	169077127	489	-8.40E-05	1.08E-08	0.418
1	169078572	169521853	193	-4.94E-05	2.62E-09	0.335
1	169522554	170372503	446	-6.98E-05	6.94E-09	0.403
1	170375663	170914261	181	-1.86E-05	3.28E-09	0.745
1	170915133	172647523	864	8.87E-05	1.30E-08	0.437
1	172648956	175384090	908	-3.87E-07	1.26E-08	0.997
1	175385076	176416712	369	-5.47E-05	7.45E-09	0.526
1	176418578	177431255	438	-3.27E-05	7.14E-09	0.699
1	177432237	178940717	621	5.78E-05	1.04E-08	0.570
1	178943012	179676274	338	-8.57E-06	4.78E-09	0.901
1	179678188	180567596	397	-1.11E-04	8.70E-09	0.234
1	180568720	181311800	309	4.74E-06	4.99E-09	0.946
1	181313404	182293001	421	-2.23E-05	8.83E-09	0.812
1	182294372	183796074	577	1.18E-04	8.51E-09	0.202
1	183796618	185511423	737	-2.23E-04	1.15E-08	0.038
1	185512838	187847742	807	4.65E-05	1.27E-08	0.680
1	187848898	189756187	598	4.87E-05	7.93E-09	0.585
1	189758772	190107648	158	1.05E-05	2.35E-09	0.828
1	190107883	191432168	472	2.98E-05	6.71E-09	0.716
1	191433917	192444256	330	-6.83E-05	4.93E-09	0.330
1	192452631	193504005	346	3.02E-05	4.95E-09	0.667
1	193505535	194000243	197	-4.19E-06	2.95E-09	0.939
1	194000953	194733444	212	-1.54E-05	3.10E-09	0.782
1	194734415	195155161	176	-8.33E-06	2.53E-09	0.869
1	195155293	195995510	312	-5.20E-05	5.46E-09	0.482
1	195995948	197309603	342	8.94E-05	5.19E-09	0.215
1	197310096	198540028	417	-5.04E-05	6.19E-09	0.522
1	198540418	199578700	369	-3.19E-05	6.48E-09	0.692
1	199580285	201070422	658	-1.04E-04	1.17E-08	0.338
1	201070740	201365889	257	6.04E-05	5.15E-09	0.400
1	201366509	202820077	608	-5.46E-05	1.22E-08	0.621
1	202820815	203979519	487	1.18E-04	1.16E-08	0.271
1	203980019	205458538	791	-4.98E-04	3.47E-08	0.008
**1**	**205459511**	**206705609**	**334**	**5.36E-04**	**1.41E-08**	**0.000**
1	206706419	208158787	548	4.59E-05	9.52E-09	0.638
1	210741816	211763053	432	-3.07E-05	8.78E-09	0.743

1	208160220	210738604	1287	-1.16E-04	2.51E-08	0.464
1	211763754	213578006	640	1.90E-04	1.24E-08	0.088
1	213578592	215128675	755	1.22E-05	1.50E-08	0.921
1	215129087	216672805	623	3.81E-05	1.07E-08	0.712
1	216673326	217575665	684	-8.16E-06	1.40E-08	0.945
1	217577346	218860068	519	4.60E-05	1.09E-08	0.659
1	218860456	220068681	530	-9.21E-06	8.52E-09	0.920
1	220069681	221856899	640	-9.23E-07	1.11E-08	0.993
1	221857359	223630391	645	-2.31E-04	1.15E-08	0.031
1	223630619	224434769	280	-3.09E-05	5.18E-09	0.668
1	224435934	226143348	569	1.14E-06	9.60E-09	0.991
1	226146348	226808294	242	-1.73E-05	4.60E-09	0.799
1	226808697	228100891	557	1.26E-04	8.95E-09	0.181
1	228101119	229075210	320	-2.81E-04	1.05E-08	0.006
1	229076157	230477970	634	8.44E-05	1.19E-08	0.439
1	230479324	231318678	503	5.08E-05	1.10E-08	0.628
1	231321215	232382234	476	-4.50E-06	8.46E-09	0.961
1	232384356	232949914	349	3.67E-05	7.20E-09	0.665
1	232950888	233750725	429	2.94E-05	8.97E-09	0.757
1	233753991	234206593	224	-3.95E-06	5.00E-09	0.955
1	234209087	235772624	700	2.01E-04	1.56E-08	0.108
1	235773790	236536723	313	3.79E-05	6.29E-09	0.632
1	236537770	237493854	565	2.57E-05	1.05E-08	0.802
1	237495080	239297872	909	1.10E-04	1.94E-08	0.429
1	239299272	240562764	671	4.53E-05	1.45E-08	0.707
1	240563367	241178287	350	8.64E-06	9.97E-09	0.931
1	241178944	241766551	356	-4.08E-05	8.84E-09	0.664
1	241768617	242697031	496	-1.64E-05	1.19E-08	0.881
1	242700803	244105053	454	8.00E-05	6.91E-09	0.336
1	244106113	244927751	381	3.02E-05	9.20E-09	0.753
1	244929262	246606531	849	4.60E-05	1.84E-08	0.734
1	246606926	246989171	160	1.06E-05	3.31E-09	0.853
1	246991094	247838628	369	-7.00E-05	7.93E-09	0.432
1	247838870	248585259	279	-2.08E-05	4.58E-09	0.759
2	11320	1158981	538	-1.71E-04	1.05E-08	0.095
2	1159982	1681720	218	4.07E-05	5.12E-09	0.569
2	1683187	3003071	697	1.05E-04	1.76E-08	0.427

2	3003472	4148069	588	-2.82E-05	1.36E-08	0.808
2	4148784	5017172	422	-6.95E-05	9.24E-09	0.470
2	5118816	6361343	633	-3.92E-05	1.69E-08	0.763
2	6363082	7322437	567	-1.01E-04	1.31E-08	0.377
2	7323039	8980346	697	-6.48E-05	1.64E-08	0.612
2	8980472	10440987	657	-1.99E-04	1.86E-08	0.144
2	10442541	10879340	235	2.06E-04	7.74E-09	0.019
2	10881867	11652586	376	1.32E-04	1.15E-08	0.216
2	11652795	12720977	565	-5.93E-05	1.57E-08	0.636
2	12723749	14648908	706	6.21E-05	1.41E-08	0.601
2	14649571	15720775	452	-2.84E-05	7.63E-09	0.745
2	15721423	16549199	386	-1.07E-04	9.64E-09	0.277
2	16549538	18344242	767	1.40E-04	1.54E-08	0.258
2	18344821	18646822	144	-5.13E-06	2.93E-09	0.924
2	18647868	20059542	691	2.21E-05	1.44E-08	0.854
2	20062043	22427190	824	-6.85E-05	3.69E-08	0.721
2	22428680	23855977	652	-8.47E-05	1.38E-08	0.470
2	23857184	25485735	454	1.17E-04	7.12E-09	0.165
2	25486770	26894217	438	3.28E-05	8.46E-09	0.721
2	26894589	28597305	482	-9.66E-06	6.83E-09	0.907
2	28597624	29484922	455	5.81E-05	9.20E-09	0.545
2	29486078	29987735	338	-2.47E-05	6.71E-09	0.763
2	29988753	31009977	499	7.02E-05	1.06E-08	0.496
2	31010212	32489851	591	-2.13E-05	1.07E-08	0.837
2	32493210	33361425	336	1.75E-05	6.95E-09	0.834
2	33362643	34279720	532	-1.26E-04	1.16E-08	0.242
2	34280335	35607183	528	2.55E-05	1.01E-08	0.800
2	35608923	36121342	278	9.99E-05	5.04E-09	0.159
2	36122006	37004185	552	-1.08E-04	1.33E-08	0.349
2	37008066	38882278	873	-1.11E-04	1.94E-08	0.423
2	38882577	40077971	310	-8.07E-07	4.72E-09	0.991
2	40080343	40625847	349	-1.25E-04	6.89E-09	0.131
2	40626336	41381901	284	-7.77E-05	5.00E-09	0.272
2	41382889	42087411	359	-3.46E-05	6.46E-09	0.666
2	42088016	42947335	404	-4.55E-05	7.00E-09	0.586
2	42949616	43307776	218	-5.64E-05	8.88E-09	0.550
2	43309247	44048346	326	6.54E-06	2.10E-08	0.964

2	44049120	45194289	539	-7.56E-05	9.98E-09	0.449
2	45195022	45562814	268	-3.62E-05	8.13E-09	0.688
2	45563613	47052067	879	6.07E-05	2.07E-08	0.673
2	47052788	47782756	369	-2.61E-05	9.99E-09	0.794
2	47782836	49112501	506	-3.43E-05	1.13E-08	0.747
2	49113132	50757671	842	-1.78E-05	1.62E-08	0.889
2	50758657	51667413	400	1.18E-05	7.51E-09	0.892
2	51668142	53351493	834	2.02E-05	1.58E-08	0.872
2	53352175	54599930	553	-4.77E-05	1.09E-08	0.649
2	54600556	56158646	724	-9.55E-05	1.42E-08	0.423
2	56160647	56599354	240	-4.04E-05	6.28E-09	0.611
2	56600739	57938354	491	-8.30E-05	1.03E-08	0.413
2	57939866	60449069	1013	-7.84E-05	2.09E-08	0.587
2	60449710	62420656	657	1.26E-04	1.12E-08	0.233
2	62425639	64623935	687	1.38E-05	2.78E-08	0.934
2	64626895	65226463	256	-2.19E-05	6.06E-09	0.778
2	65228236	66374803	558	6.69E-05	1.10E-08	0.524
2	66375016	67978287	797	-5.78E-05	1.70E-08	0.657
2	67981487	68871573	395	-4.84E-05	7.43E-09	0.574
2	68871710	70753882	813	-9.68E-05	1.43E-08	0.418
2	70756057	71704491	467	-2.06E-05	1.03E-08	0.839
2	71704844	73172972	470	3.09E-05	9.25E-09	0.748
2	73174848	74121000	253	3.41E-05	3.58E-09	0.569
2	74121278	76615798	1069	-4.47E-05	2.13E-08	0.759
2	76616819	77384991	368	4.72E-05	7.43E-09	0.584
2	77386522	79007966	561	3.39E-05	1.18E-08	0.754
2	80804991	81588311	230	-1.23E-05	3.84E-09	0.842
2	79008155	80804284	1066	2.64E-05	2.32E-08	0.862
2	81588482	83604057	600	-2.26E-05	8.16E-09	0.803
2	83604796	85283713	481	5.70E-05	7.62E-09	0.514
2	85284353	86559358	571	-8.61E-06	1.45E-08	0.943
2	86562902	89135764	457	-7.39E-05	8.24E-09	0.416
2	92324281	98790763	459	3.58E-05	6.89E-09	0.666
2	98791433	100195879	407	-9.61E-05	5.84E-09	0.209
2	100197638	101338509	453	3.39E-05	7.30E-09	0.692
2	101338644	102688272	529	1.10E-04	1.10E-08	0.294
2	102688495	103262415	357	-5.33E-05	5.62E-09	0.477

2	103264434	104481488	294	6.99E-06	5.34E-09	0.924
2	104485456	105871610	462	3.18E-06	9.50E-09	0.974
2	105872982	107406122	722	6.76E-05	1.66E-08	0.600
2	107406885	108427822	407	1.29E-05	7.46E-09	0.881
2	108427959	110109210	583	4.33E-05	1.06E-08	0.674
2	110109222	112924907	583	6.35E-05	3.27E-08	0.725
2	112924973	114846282	716	-3.91E-05	1.30E-08	0.731
2	114848115	116770829	607	3.43E-05	1.16E-08	0.750
2	116772945	117727045	302	1.34E-05	4.51E-09	0.842
2	117728904	119581280	716	-4.94E-05	1.43E-08	0.679
2	119581439	121070811	505	-4.40E-05	9.46E-09	0.651
2	121073253	123241656	762	-4.64E-05	2.28E-08	0.758
2	123244233	124730999	532	-5.09E-06	9.06E-09	0.957
2	124733246	125735879	428	6.06E-05	8.64E-09	0.515
2	125741434	127372889	519	1.54E-05	8.47E-09	0.867
2	127374341	129310292	757	3.31E-05	1.52E-08	0.788
2	129312188	129864416	215	-7.16E-07	4.63E-09	0.992
2	129864730	131463038	407	-4.94E-05	7.59E-09	0.570
2	131466136	133030349	223	2.32E-05	4.76E-09	0.736
2	133034031	133950661	429	4.61E-05	9.47E-09	0.635
2	133951544	136822267	1073	-5.75E-05	1.87E-08	0.674
2	136825272	138619715	738	3.63E-05	1.48E-08	0.766
2	138620371	139412389	267	6.93E-05	4.55E-09	0.305
2	139413312	140902261	570	-1.17E-05	1.10E-08	0.911
2	140902383	141803496	400	-6.96E-05	7.79E-09	0.430
2	141803602	143254981	699	1.13E-04	1.44E-08	0.346
2	143255262	145301314	617	-5.75E-05	1.55E-08	0.644
2	145303576	147252762	524	-1.74E-05	9.55E-09	0.859
2	147255216	149268931	496	-3.89E-05	9.07E-09	0.683
2	149270710	151184680	609	-1.96E-04	1.19E-08	0.073
2	151191481	152490219	540	-1.01E-04	1.02E-08	0.315
2	152491269	153369892	310	1.13E-06	5.06E-09	0.987
2	153371042	154414456	449	-3.92E-06	9.89E-09	0.969
2	154415112	155208173	297	-1.24E-05	5.90E-09	0.872
2	155209736	156568446	482	3.37E-05	8.73E-09	0.718
2	156568806	158165818	441	1.52E-05	9.28E-09	0.875
2	158173710	159574883	463	-1.66E-05	9.81E-09	0.867

2	159575554	161077823	603	-4.13E-05	1.07E-08	0.690
2	161078891	163358537	677	-1.32E-05	1.04E-08	0.897
2	163359084	165200324	482	7.23E-07	9.73E-09	0.994
2	165202539	167120956	737	3.19E-04	1.43E-08	0.008
2	167123247	168427789	400	-1.91E-05	6.91E-09	0.818
2	168431583	169176766	322	-2.33E-05	7.22E-09	0.784
2	169178092	170973262	788	-6.08E-05	1.54E-08	0.624
2	170973461	172086127	479	-2.50E-05	1.05E-08	0.807
2	172088943	173586201	588	1.05E-04	3.66E-08	0.585
2	173588663	175585616	784	-3.73E-05	1.76E-08	0.779
2	175586510	177326682	628	1.41E-04	1.09E-08	0.177
2	177327202	178551958	557	6.72E-05	1.01E-08	0.503
2	178552788	180138313	563	-1.07E-04	1.01E-08	0.287
2	180139219	180661233	321	1.96E-05	9.36E-09	0.840
2	180662102	181627195	413	-3.23E-06	1.22E-08	0.977
2	181628139	182840114	361	-1.07E-04	6.56E-09	0.188
2	182845657	184442530	601	6.61E-05	1.07E-08	0.522
2	184443166	185277576	257	5.52E-05	4.56E-09	0.413
2	185279087	186731282	380	-1.60E-05	5.92E-09	0.835
2	186731309	188540877	478	4.46E-06	7.19E-09	0.958
2	188542361	190794689	527	2.32E-05	9.05E-09	0.807
2	190797341	191870624	348	-6.70E-05	8.36E-09	0.464
2	191873553	192695107	283	-3.22E-05	6.70E-09	0.694
2	192696255	195109845	440	-2.30E-05	7.02E-09	0.784
2	195117932	197307918	618	6.98E-05	1.10E-08	0.506
2	197308707	198140953	269	-3.82E-05	4.19E-09	0.555
2	198141746	200498970	618	9.24E-05	8.95E-09	0.329
2	200504209	201571624	452	9.74E-05	7.93E-09	0.274
2	201572564	202829668	381	-4.65E-05	7.68E-09	0.596
2	202830132	204814896	524	-5.49E-05	9.30E-09	0.569
2	204815902	206043539	517	-1.75E-05	1.15E-08	0.870
2	206045927	207721890	726	-2.21E-05	1.53E-08	0.858
2	207722425	208639154	388	-1.04E-04	1.23E-08	0.347
2	208641174	209940909	408	-5.85E-05	7.77E-09	0.507
2	209941332	210832981	269	-3.63E-06	4.37E-09	0.956
2	210838781	212377672	605	-2.26E-06	1.15E-08	0.983
2	212377759	212845716	216	-2.50E-05	5.42E-09	0.735

2	212845994	215005050	818	-1.00E-04	1.49E-08	0.411
2	215006557	216448007	604	-1.95E-05	1.18E-08	0.858
2	216449570	217184275	342	-4.12E-05	7.87E-09	0.643
2	217184715	218615634	619	-3.12E-05	1.30E-08	0.784
2	218616633	219671910	400	-2.77E-05	6.24E-09	0.726
2	219675067	220774229	409	4.27E-05	7.34E-09	0.618
2	220775971	221445218	359	-4.63E-05	6.89E-09	0.577
2	221445953	222985126	638	1.50E-05	1.37E-08	0.898
2	222985611	224002780	432	-8.37E-05	9.14E-09	0.382
2	224003069	225832409	735	8.02E-06	1.42E-08	0.946
2	225833758	227531180	618	-2.63E-05	1.07E-08	0.800
2	227532464	228456451	380	-2.14E-05	7.00E-09	0.798
2	228457117	229368530	432	-3.87E-05	9.35E-09	0.689
2	229368812	230282624	395	-8.61E-05	8.35E-09	0.346
2	230283310	231322125	471	1.31E-04	1.01E-08	0.191
2	231322831	232790491	543	-2.66E-05	1.18E-08	0.807
2	232791948	233964632	468	5.15E-05	9.11E-09	0.590
2	233968153	234699046	367	-1.49E-05	5.65E-09	0.843
2	234699366	235626316	630	5.37E-05	1.53E-08	0.665
2	235627087	236619385	515	-3.44E-06	1.44E-08	0.977
2	236619713	238209382	607	8.96E-05	1.29E-08	0.431
2	238209871	239122917	445	4.65E-05	1.03E-08	0.647
2	239123082	239801883	288	4.15E-05	5.98E-09	0.592
2	239832097	240325991	241	-6.83E-05	7.96E-09	0.444
2	240327154	240961852	336	-1.03E-04	9.00E-09	0.276
2	240962194	241907076	460	-1.72E-05	1.12E-08	0.871
2	241909927	243185680	318	8.60E-05	2.05E-08	0.547
3	60197	1128944	484	3.19E-05	1.41E-08	0.789
3	1129028	1527748	338	4.08E-05	1.06E-08	0.691
3	1528147	2423237	600	2.08E-06	1.59E-08	0.987
3	2423999	2769230	240	-8.39E-05	7.63E-09	0.337
3	2769392	3364279	476	7.03E-05	1.68E-08	0.587
3	3364442	4430024	629	-1.86E-05	1.61E-08	0.884
3	4430848	5564422	653	2.50E-06	2.04E-08	0.986
3	5565384	6421659	512	-4.11E-05	1.43E-08	0.731
3	6422814	7509288	573	1.14E-04	1.46E-08	0.345
3	7509715	7991133	371	-3.30E-06	9.10E-09	0.972

3	7991981	9194649	775	-2.06E-04	2.11E-08	0.155
3	9195331	10526438	649	-1.01E-05	1.67E-08	0.938
3	10526803	11220278	388	6.02E-05	1.23E-08	0.587
3	11221721	12858028	673	-1.16E-04	1.57E-08	0.356
3	12859004	13523338	331	-1.88E-04	9.31E-09	0.052
3	13524741	14309204	364	9.53E-06	1.02E-08	0.925
3	14309548	14816364	328	-3.44E-05	1.03E-08	0.735
3	14816745	16661587	812	-4.79E-05	1.96E-08	0.733
3	16662488	18454062	541	-8.85E-05	1.24E-08	0.426
3	18457304	18886128	166	1.53E-04	5.81E-09	0.044
3	18886158	20752492	602	1.12E-04	1.50E-08	0.359
3	20753368	21522703	362	1.19E-04	9.61E-09	0.224
3	21523116	22102707	446	-1.27E-05	1.21E-08	0.908
3	22103146	23122359	464	-5.29E-05	1.33E-08	0.647
3	23123318	23802647	259	2.27E-05	7.19E-09	0.789
3	23803541	25459497	887	4.85E-06	2.31E-08	0.975
3	25460109	26392563	317	4.71E-05	6.90E-09	0.571
3	26393305	27606695	465	-8.51E-05	1.02E-08	0.400
3	27609447	29409547	794	-6.24E-05	2.14E-08	0.670
3	29409589	30716602	619	8.80E-05	1.69E-08	0.498
3	30717661	31720982	438	1.19E-05	9.30E-09	0.902
3	31721774	33011510	531	-6.90E-05	1.63E-08	0.589
3	33012981	33986974	341	6.15E-05	7.44E-09	0.476
3	33988115	35241960	452	2.33E-05	1.03E-08	0.819
3	35243764	36481385	481	-6.48E-05	1.08E-08	0.533
3	36484375	38078255	626	1.49E-04	1.43E-08	0.213
3	38080101	38723967	263	-1.13E-05	6.08E-09	0.885
3	38724849	40649760	701	-2.67E-06	1.49E-08	0.983
3	40660283	42184173	434	-1.66E-05	9.15E-09	0.862
3	42184361	45162316	1057	-6.16E-05	2.32E-08	0.686
3	45165430	46657162	653	1.20E-04	7.05E-08	0.651
3	46657428	48525955	404	4.97E-05	8.03E-09	0.579
3	48531227	52214640	853	6.59E-05	1.68E-08	0.612
3	52215002	54078568	705	5.74E-05	1.45E-08	0.633
3	54080241	55731145	920	-5.21E-05	2.51E-08	0.742
3	55733390	58063819	872	-1.87E-04	1.84E-08	0.168
3	58067795	58700675	307	1.36E-05	6.66E-09	0.867

3	58700802	60347135	935	-1.74E-05	2.61E-08	0.914
3	60347701	61277928	613	1.65E-05	1.56E-08	0.895
3	61279726	62881664	854	-5.18E-05	2.48E-08	0.742
3	62882619	63504979	310	-9.94E-05	7.59E-09	0.254
3	63505191	64473972	483	-5.00E-05	1.30E-08	0.660
3	64475164	65337581	545	1.04E-04	1.50E-08	0.397
3	65340365	66169323	368	-4.89E-05	1.10E-08	0.640
3	66170253	67294968	419	-6.42E-05	1.05E-08	0.531
3	67299401	69197507	757	-8.68E-05	1.76E-08	0.513
3	69205190	70288126	413	4.20E-05	1.01E-08	0.676
3	70289919	71418327	409	-1.60E-04	1.83E-08	0.236
3	71421152	73238935	812	-8.59E-05	2.40E-08	0.579
3	73239448	75376291	876	-5.08E-05	2.23E-08	0.734
3	75377075	76854466	340	8.13E-05	8.02E-09	0.364
3	76855676	77507524	287	-1.68E-05	7.20E-09	0.843
3	77508835	79022673	488	5.90E-05	1.15E-08	0.583
3	79024541	81187867	525	-3.39E-05	1.04E-08	0.740
3	81190039	82446513	356	-6.24E-06	6.70E-09	0.939
3	82446746	83946780	383	-1.21E-05	7.97E-09	0.893
3	83947451	85090935	310	-2.30E-07	6.50E-09	0.998
3	85093629	86734415	434	1.33E-04	8.26E-09	0.143
3	86736368	87407921	210	2.65E-05	1.93E-08	0.848
3	87408634	88725583	376	-5.63E-05	7.65E-09	0.520
3	88725754	94202010	444	1.02E-04	9.27E-09	0.289
3	94202771	96056259	472	-1.01E-04	8.87E-09	0.284
3	96679063	98299365	463	-3.80E-05	9.60E-09	0.698
3	98300731	100591006	762	6.81E-05	1.52E-08	0.580
3	100591848	101808006	473	1.15E-04	9.82E-09	0.246
3	101808379	103025517	322	1.09E-05	6.95E-09	0.896
3	103025715	103698105	210	6.27E-05	4.53E-09	0.352
3	103700909	104579408	324	7.47E-05	7.29E-09	0.382
3	104580063	104878270	154	5.04E-05	3.44E-09	0.390
3	104879008	106330349	517	1.67E-05	1.25E-08	0.881
3	106332831	107729547	479	4.59E-05	1.61E-08	0.718
3	107730020	108635884	340	-6.45E-05	7.69E-09	0.462
3	108636466	109961316	402	8.65E-05	8.28E-09	0.342
3	109962369	111416310	404	2.57E-05	8.41E-09	0.779

3	111416451	112182995	420	5.11E-08	9.37E-09	1.000
3	112183360	113323793	431	-1.16E-05	1.55E-08	0.926
3	113324119	115280312	582	1.23E-04	1.44E-08	0.305
3	115282058	116450487	508	1.95E-05	1.23E-08	0.860
3	116452274	117998840	676	1.82E-04	1.81E-08	0.176
3	118000136	120520317	1012	-4.62E-05	2.25E-08	0.758
3	120521733	121335461	214	-7.31E-06	4.20E-09	0.910
3	121336367	122692039	596	1.37E-04	1.28E-08	0.225
3	122693533	123196819	219	-1.21E-05	5.10E-09	0.865
3	123200440	124677278	597	-3.71E-05	1.37E-08	0.751
3	124678230	125875831	441	-1.67E-04	1.06E-08	0.106
3	125876862	126213162	202	3.59E-05	5.33E-09	0.623
3	126213467	127471230	536	1.13E-05	1.26E-08	0.920
3	127471641	130243259	807	-2.74E-04	5.20E-08	0.230
3	130244120	131373405	333	-6.04E-05	6.52E-09	0.454
3	131373740	132115911	443	2.72E-05	9.96E-09	0.785
3	132117285	133128449	407	-7.53E-05	8.73E-09	0.420
3	133129272	134383242	550	1.61E-05	1.42E-08	0.893
3	137370076	138662229	384	1.16E-04	7.96E-09	0.194
3	134385789	137369181	1105	9.25E-05	2.38E-08	0.549
3	138665796	140275652	774	6.21E-05	1.87E-08	0.650
3	140276484	142863564	911	-5.76E-05	2.01E-08	0.684
3	142864473	143494546	387	-2.42E-05	1.20E-08	0.825
3	143495679	145663879	729	9.21E-05	1.57E-08	0.462
3	145664135	146722271	401	1.57E-04	9.20E-09	0.102
3	146722383	148316359	475	-5.97E-05	1.16E-08	0.580
3	148316766	149988630	749	1.74E-04	1.91E-08	0.208
3	149994553	151178708	481	-6.72E-05	1.19E-08	0.538
3	151178836	151904190	289	-5.30E-05	6.82E-09	0.521
3	151905386	153054901	368	3.00E-05	9.20E-09	0.755
3	153056839	154181836	468	6.04E-05	1.02E-08	0.550
3	154183562	156005200	508	-6.37E-06	1.21E-08	0.954
3	156005905	157311596	528	6.56E-05	1.20E-08	0.549
3	157312028	159272972	716	1.87E-05	1.50E-08	0.879
3	159274785	161523859	701	7.26E-05	1.46E-08	0.548
3	161524489	162065286	248	5.46E-06	6.81E-09	0.947
3	162067622	163250087	374	-2.10E-06	7.42E-09	0.981

3	163251056	164816939	493	-4.84E-05	9.25E-09	0.615
3	164820183	167116849	615	5.06E-05	1.34E-08	0.662
3	167117143	167912878	293	7.71E-06	6.24E-09	0.922
3	167913564	170158128	713	-6.81E-05	3.60E-08	0.720
3	170159134	171311936	486	-5.06E-05	1.38E-08	0.667
3	171312880	172965694	657	1.80E-05	1.79E-08	0.893
3	172966405	174274308	513	-1.93E-04	1.22E-08	0.081
3	174275336	175049294	365	-2.76E-05	9.52E-09	0.778
3	175050262	176268375	459	-1.80E-05	1.10E-08	0.864
3	176269091	177530606	501	-5.79E-05	1.31E-08	0.614
3	177531847	179359242	627	1.31E-04	1.48E-08	0.282
3	179366819	181426554	525	-7.34E-05	1.20E-08	0.503
3	181431571	182975975	457	5.54E-05	1.23E-08	0.618
3	182976382	183762614	363	-1.02E-04	7.63E-09	0.241
3	183762644	185262137	590	-2.52E-05	1.64E-08	0.844
3	185263467	186780913	684	-1.13E-04	2.05E-08	0.430
3	186782999	187220633	227	1.73E-04	6.07E-09	0.027
3	187220777	188201380	508	6.37E-05	1.48E-08	0.601
3	188202550	189531496	581	-1.76E-05	1.74E-08	0.893
3	189531911	190303805	414	8.21E-06	1.11E-08	0.938
3	190304172	190992002	285	5.26E-05	6.84E-09	0.524
3	190993386	191840443	361	-1.48E-05	8.49E-09	0.872
3	191840983	192753870	379	-1.26E-04	9.29E-09	0.193
3	192754177	193885973	511	7.67E-05	1.39E-08	0.515
3	193886289	194464791	285	1.08E-04	8.57E-09	0.243
3	194465233	194756339	146	4.48E-05	5.22E-09	0.536
3	194756597	195854787	319	3.17E-05	8.06E-09	0.724
3	195855401	196414159	209	-8.42E-05	6.14E-09	0.282
3	196415355	197962382	525	3.65E-05	1.20E-08	0.739
4	10642	685993	148	-1.52E-05	2.09E-09	0.739
4	686563	1422863	300	-1.17E-04	5.23E-09	0.104
4	3613164	4263579	146	9.09E-05	5.07E-09	0.202
4	1478711	3612028	636	-6.54E-05	1.03E-08	0.518
4	4264705	5044744	439	2.13E-05	9.19E-09	0.824
4	5047695	5971152	585	-2.64E-05	1.39E-08	0.822
4	5971634	7135912	601	-9.07E-05	1.62E-08	0.476
4	7136158	8054472	690	-3.06E-04	2.35E-08	0.045

4	8055323	9408136	367	3.96E-05	1.25E-08	0.723
4	9408158	10696229	605	8.85E-06	7.88E-09	0.921
4	10696913	12198579	733	8.07E-05	1.25E-08	0.470
4	12201002	13002341	347	-1.28E-05	4.98E-09	0.856
4	13002905	14394452	629	3.67E-06	1.24E-08	0.974
4	14394902	15849986	605	7.34E-07	1.02E-08	0.994
4	15850685	16520118	382	5.27E-05	7.35E-09	0.538
4	16521003	18752293	860	4.62E-05	1.41E-08	0.697
4	18752722	20084049	441	6.61E-05	7.46E-09	0.444
4	20084563	21292213	549	1.90E-04	9.71E-09	0.054
4	21292651	22393873	439	9.89E-06	7.13E-09	0.907
4	22395570	23563570	369	4.07E-05	6.66E-09	0.618
4	23563931	24992827	683	-2.64E-05	1.34E-08	0.820
4	24993167	26468997	677	1.70E-04	1.62E-08	0.182
4	26473220	27903383	509	-2.18E-05	9.64E-09	0.824
4	27904556	28871862	388	8.61E-05	6.07E-09	0.269
4	28872833	30453156	508	-9.55E-06	8.63E-09	0.918
4	30453619	31739254	480	2.65E-05	8.05E-09	0.768
4	31740009	32592949	294	3.35E-06	4.71E-09	0.961
4	32593453	33640100	204	-1.01E-04	2.89E-09	0.060
4	33640864	35150284	378	-2.22E-06	4.01E-09	0.972
4	35151237	36301997	434	1.55E-05	6.92E-09	0.853
4	36304377	37534798	577	-6.18E-06	1.09E-08	0.953
4	37536119	37886455	212	-8.25E-05	5.01E-09	0.244
4	37887735	38974458	602	5.86E-05	1.33E-08	0.611
4	38976533	40199852	438	-1.47E-04	8.10E-09	0.103
4	40200304	40869208	304	2.10E-05	8.37E-09	0.818
4	40870402	42662283	725	-9.71E-05	1.29E-08	0.392
4	42662718	43962569	451	-1.55E-05	6.80E-09	0.851
4	43963518	45187835	413	1.58E-05	5.84E-09	0.836
4	45188855	47543891	621	-9.87E-07	8.67E-09	0.992
4	47544012	48226267	277	2.02E-05	3.74E-09	0.741
4	48227642	53412129	408	7.42E-05	3.86E-09	0.232
4	53413674	55202613	629	-3.51E-05	9.22E-09	0.715
4	55202697	57418400	883	8.25E-05	1.56E-08	0.510
4	57420107	58696072	630	1.48E-05	1.24E-08	0.894
4	58697912	59358251	234	1.16E-05	3.94E-09	0.854

4	59358729	60145179	250	-1.97E-05	4.05E-09	0.757
4	60145181	61371992	375	-5.35E-05	5.78E-09	0.482
4	61372415	61872517	167	-1.31E-05	2.71E-09	0.801
4	61872656	64117718	644	-2.19E-05	1.11E-08	0.835
4	64118963	65300308	239	-5.00E-06	3.02E-09	0.927
4	65562213	66598615	369	2.87E-05	5.83E-09	0.707
4	66599449	68058793	478	-9.35E-05	7.58E-09	0.282
4	68059850	68455531	160	-6.79E-06	2.40E-09	0.890
4	68456074	69954151	269	-2.32E-05	4.14E-09	0.718
4	69954297	70517143	265	1.97E-05	3.14E-09	0.725
4	70517792	71516586	418	6.95E-05	5.60E-09	0.353
4	72416258	75196731	712	1.27E-04	1.22E-08	0.251
4	75555658	76283179	293	1.72E-05	5.56E-09	0.817
4	76284248	77128724	363	-3.16E-06	4.52E-09	0.963
4	77129568	78333381	606	-7.11E-05	1.01E-08	0.480
4	78333798	79469226	505	-3.11E-05	7.42E-09	0.718
4	79470550	80994124	436	-4.89E-06	7.30E-09	0.954
4	80995881	81978151	261	-1.26E-05	3.88E-09	0.840
4	81979993	83012967	403	-1.79E-05	5.88E-09	0.815
4	83017789	83994716	385	-3.81E-05	5.65E-09	0.612
4	83998014	85080933	430	-1.57E-05	7.49E-09	0.856
4	85081980	87980070	900	-1.03E-05	1.22E-08	0.926
4	87980748	89236855	568	2.67E-05	1.05E-08	0.794
4	89238369	90126190	323	-8.89E-05	5.72E-09	0.240
4	90126331	91453993	446	-1.08E-04	7.52E-09	0.214
4	91456722	94229509	751	1.73E-05	1.25E-08	0.877
4	94230511	95596188	496	-1.92E-04	4.01E-08	0.337
4	95596744	96181408	236	2.79E-05	4.06E-09	0.661
4	96182669	96464931	212	2.66E-05	4.56E-09	0.694
4	96466095	97911191	514	7.00E-05	7.83E-09	0.429
4	97911459	99422081	371	-6.53E-06	3.46E-09	0.912
4	99423280	100322786	399	1.65E-06	5.74E-09	0.983
4	100323029	101897750	598	2.49E-05	8.59E-09	0.788
4	101898276	103387944	552	-7.46E-05	8.14E-09	0.408
4	103388441	104802530	434	1.25E-05	4.88E-09	0.858
4	104803329	106479425	508	7.21E-04	7.92E-08	0.010
4	106482185	108468632	626	-1.30E-04	1.83E-08	0.337

4	108469756	109979131	574	2.02E-05	8.51E-09	0.827
4	109980374	112204254	780	1.70E-04	1.39E-08	0.151
4	112204259	114902479	1023	3.68E-05	1.62E-08	0.773
4	114904730	117275534	678	-8.51E-05	8.80E-09	0.364
4	117275542	118219755	308	-8.65E-05	5.02E-09	0.222
4	118220292	119945314	632	-2.64E-05	9.72E-09	0.789
4	119946706	121391843	518	3.06E-05	6.96E-09	0.713
4	121393020	121973864	244	1.99E-04	4.36E-09	0.003
4	121976226	123561869	550	-3.18E-05	8.68E-09	0.733
4	123563078	125863762	773	5.01E-05	1.27E-08	0.657
4	125863974	127236805	494	-1.45E-04	1.29E-08	0.201
4	127237363	129514427	625	-1.32E-05	7.99E-09	0.882
4	129514448	130364383	286	8.46E-05	4.79E-09	0.221
4	130364741	130949530	194	-4.31E-05	4.34E-09	0.513
4	130950008	131948861	379	4.53E-05	6.61E-09	0.578
4	131951466	133741365	442	-5.32E-05	6.35E-09	0.505
4	133741753	135744132	532	1.46E-05	7.29E-09	0.865
4	135749591	137247266	391	1.47E-05	7.08E-09	0.861
4	137247703	137987679	298	2.19E-05	6.10E-09	0.779
4	137988838	139114529	402	-1.45E-06	6.98E-09	0.986
4	139115093	139552965	239	7.32E-05	4.73E-09	0.287
4	139553359	140546206	342	-7.49E-05	5.62E-09	0.318
4	140548713	141751418	489	5.32E-05	1.43E-08	0.656
4	141752960	142807957	391	-7.42E-05	7.43E-09	0.389
4	142808503	145021868	626	-1.70E-05	7.30E-09	0.842
4	145024452	148047972	851	1.70E-04	1.31E-08	0.137
4	148053679	150632409	779	1.32E-04	1.18E-08	0.224
4	150634191	153226998	835	-3.78E-06	1.07E-08	0.971
4	153233476	154947363	614	4.17E-05	1.01E-08	0.678
4	154949091	156604003	633	-2.88E-05	1.20E-08	0.792
4	156604173	158112985	581	1.65E-05	1.07E-08	0.873
4	158114105	160356254	643	-2.99E-05	9.36E-09	0.758
4	160357877	160822830	147	-2.76E-05	2.02E-09	0.539
4	161056173	161796696	321	1.50E-05	5.59E-09	0.841
4	162151565	162629423	187	-5.07E-05	4.64E-09	0.456
4	162629512	163181531	215	2.32E-05	3.57E-09	0.698
4	163183341	163726178	186	-1.52E-05	3.66E-09	0.801

4	163728264	164910818	398	4.18E-05	6.81E-09	0.613
4	164913270	165817608	367	7.56E-06	6.68E-09	0.926
4	165820093	166609852	327	-3.69E-05	6.53E-09	0.648
4	166611193	167643023	297	3.36E-06	5.84E-09	0.965
4	167643601	169568082	733	7.26E-05	1.29E-08	0.522
4	169569516	170676328	420	-3.56E-05	6.61E-09	0.662
4	170678993	172735897	572	-1.85E-05	8.71E-09	0.843
4	172737462	173760563	310	2.27E-06	4.63E-09	0.973
4	173761916	174832248	301	-9.18E-06	7.22E-09	0.914
4	174833988	176193705	541	9.87E-06	1.23E-08	0.929
4	176199091	177290105	362	7.01E-06	7.45E-09	0.935
4	177290945	177972278	282	7.50E-06	4.95E-09	0.915
4	177974062	178758792	336	-5.12E-05	8.20E-09	0.572
4	178758930	180119509	494	2.46E-05	9.50E-09	0.801
4	180120693	181732608	833	2.63E-06	1.94E-08	0.985
4	181732746	182420390	387	2.30E-05	1.09E-08	0.826
4	184283411	185477454	593	-7.91E-05	1.67E-08	0.541
4	182420863	184283167	1001	-7.94E-05	2.60E-08	0.622
4	185477964	185932497	229	-4.12E-05	4.08E-09	0.519
4	185934003	187113041	594	-1.31E-04	1.42E-08	0.273
4	187114073	188687052	716	-7.44E-05	1.59E-08	0.556
4	188687155	189524815	363	-9.21E-05	1.10E-08	0.380
4	189525752	190088167	269	-5.75E-05	6.10E-09	0.462
4	190088768	191043878	198	-2.98E-05	4.50E-09	0.657
5	10056	1267356	414	7.79E-05	1.01E-08	0.438
5	1270983	1762678	241	1.84E-04	1.63E-08	0.149
5	1763566	2369337	388	5.53E-06	2.25E-08	0.971
5	2369710	2979000	434	8.66E-05	9.68E-09	0.379
5	2979710	3600334	481	9.27E-05	1.01E-08	0.357
5	3601924	4222111	305	5.76E-05	6.07E-09	0.460
5	4223577	5137474	431	5.06E-05	8.46E-09	0.582
5	5137788	5718773	374	-3.49E-05	7.84E-09	0.694
5	5719162	6858229	682	-5.39E-05	1.32E-08	0.639
5	6859297	7852978	494	8.04E-05	9.18E-09	0.402
5	7853304	9084076	627	2.32E-05	1.03E-08	0.819
5	9084701	10533767	856	5.01E-05	1.64E-08	0.696
5	10666804	11170506	324	-1.90E-05	5.47E-09	0.797

5	11170703	13603734	759	1.11E-04	1.10E-08	0.290
5	13603883	14987807	739	-7.50E-05	1.17E-08	0.488
5	14988284	16034608	298	-2.95E-06	3.26E-09	0.959
5	16035369	17161451	493	-4.38E-05	9.95E-09	0.661
5	17162704	18048683	258	2.13E-04	5.71E-09	0.005
5	18050107	18791978	251	-2.33E-05	4.72E-09	0.735
5	18792568	19486153	258	2.82E-05	4.08E-09	0.659
5	19487359	20414139	285	1.17E-05	3.35E-09	0.840
5	20415799	21573200	238	9.20E-06	3.82E-09	0.882
5	21574347	23150924	572	2.13E-05	6.88E-09	0.797
5	23153562	24794957	518	1.47E-06	7.76E-09	0.987
5	24796795	26183141	453	9.24E-05	8.06E-09	0.303
5	26185302	26849079	203	7.26E-05	5.13E-09	0.311
5	26849808	28303480	390	4.84E-05	6.27E-09	0.541
5	28310093	30129786	582	1.62E-04	9.60E-09	0.098
5	30130713	31748529	717	4.12E-05	1.35E-08	0.722
5	31748611	33944217	987	1.35E-05	2.02E-08	0.924
5	33945758	35798682	685	4.87E-05	1.18E-08	0.654
5	35799275	37888797	709	1.59E-05	1.12E-08	0.880
5	37889231	39438373	674	6.43E-05	1.19E-08	0.556
5	39438575	40954879	626	6.51E-05	7.82E-09	0.462
5	40955561	42620153	556	4.26E-05	6.37E-09	0.594
5	42623245	43859513	306	2.95E-05	3.02E-09	0.592
5	43862567	45286358	281	3.97E-05	2.77E-09	0.450
5	45286977	50161698	288	-4.90E-07	2.28E-09	0.992
5	50162186	52133670	655	-9.71E-05	9.60E-09	0.322
5	52134410	52661197	301	7.40E-05	4.54E-09	0.272
5	52662483	53513531	324	5.23E-05	5.70E-09	0.489
5	53515888	55412347	789	-6.94E-05	1.07E-08	0.502
5	55413961	55932471	298	-7.36E-05	8.18E-09	0.416
5	55933701	57337744	583	-2.78E-05	8.47E-09	0.762
5	57338357	58834736	805	1.19E-04	1.31E-08	0.297
5	58834852	60880207	583	8.08E-06	6.99E-09	0.923
5	60883078	62531300	657	4.65E-05	8.66E-09	0.618
5	62531952	63837838	373	8.94E-06	4.98E-09	0.899
5	63840208	65083148	458	8.02E-05	5.76E-09	0.290
5	65083209	65909616	333	-1.11E-04	6.12E-09	0.156

5	65910316	67096208	517	1.07E-04	8.52E-09	0.247
5	67096780	70682458	687	-5.89E-05	1.18E-08	0.587
5	70683839	72420748	628	-1.42E-04	7.40E-09	0.098
5	72420784	73507955	557	-7.55E-05	1.00E-08	0.451
5	73508509	75240469	728	-8.99E-05	1.08E-08	0.387
5	75242395	75740199	233	2.16E-05	4.04E-09	0.734
5	75743532	77203135	737	2.69E-04	1.36E-08	0.021
5	77203718	79068089	683	-2.47E-05	1.07E-08	0.811
5	79068300	80480761	702	5.04E-05	1.30E-08	0.659
5	80481996	81712715	433	-4.12E-05	6.79E-09	0.617
5	81714078	82658571	362	-1.17E-04	6.48E-09	0.144
5	82660901	84397685	585	6.52E-05	1.19E-08	0.551
5	84399219	85315500	310	-7.35E-06	3.87E-09	0.906
5	85316429	86490293	328	3.68E-06	5.36E-09	0.960
5	86492986	88065637	303	-1.04E-05	3.47E-09	0.859
5	88069593	90500398	713	-5.55E-05	1.10E-08	0.596
5	90504842	93602613	719	-9.13E-05	9.18E-09	0.341
5	93607443	95822590	794	-1.09E-04	1.25E-08	0.328
5	95822732	96867936	466	-2.44E-05	6.91E-09	0.769
5	96870861	97435808	143	4.06E-05	2.19E-09	0.385
5	97436504	99258325	556	-3.74E-05	7.51E-09	0.666
5	99259751	101101103	482	2.64E-06	5.72E-09	0.972
5	101101325	102681586	443	3.23E-05	4.56E-09	0.632
5	102686157	104542488	700	4.39E-05	1.22E-08	0.692
5	104543131	105885913	312	1.09E-06	4.60E-09	0.987
5	105887333	106800282	343	-1.31E-04	6.42E-09	0.101
5	108633347	109231597	246	-1.25E-05	2.94E-09	0.817
5	106800765	108633176	758	3.93E-05	1.36E-08	0.736
5	109232816	110819753	510	1.29E-04	7.43E-09	0.133
5	110821786	111962676	470	-1.10E-04	8.52E-09	0.234
5	111963025	112396750	155	7.78E-06	1.89E-09	0.858
5	112397011	113603768	561	2.17E-05	8.21E-09	0.811
5	113604579	114448326	332	1.12E-05	5.06E-09	0.875
5	114448597	115828291	681	-4.17E-05	1.08E-08	0.689
5	115829425	117003674	543	1.56E-04	9.59E-09	0.112
5	117004531	117712427	262	4.27E-05	4.48E-09	0.524
5	117714517	119668423	688	-6.27E-05	1.12E-08	0.553

5	119669457	120962383	495	2.88E-05	8.50E-09	0.755
5	120963248	121481183	148	3.59E-05	2.25E-09	0.449
5	121485609	122603725	451	-3.23E-05	6.80E-09	0.696
5	122604501	123236124	257	-3.03E-05	3.85E-09	0.625
5	123238611	123797013	229	3.48E-05	4.11E-09	0.587
5	123798436	124746813	465	4.52E-05	9.80E-09	0.648
5	124748624	126938920	873	-1.21E-04	1.43E-08	0.311
5	126940058	128727903	666	-4.36E-05	9.30E-09	0.651
5	128730275	130376511	412	6.94E-06	4.09E-09	0.914
5	130376936	131873073	450	2.30E-05	6.13E-09	0.769
5	131874022	132603137	223	-2.15E-05	3.35E-09	0.710
5	132609517	134321546	718	-1.56E-04	1.32E-08	0.174
5	134329463	135339464	501	-4.45E-05	8.06E-09	0.620
5	135340951	137948424	905	2.60E-05	1.11E-08	0.806
5	137950183	140644484	763	-3.61E-05	7.63E-09	0.679
5	140644957	141640050	474	-7.21E-05	9.10E-09	0.449
5	141640419	142230251	276	-8.57E-05	5.99E-09	0.268
5	142230949	144583138	961	1.29E-04	1.33E-08	0.263
5	144584265	145803004	431	-7.43E-06	4.97E-09	0.916
5	145803849	147651230	761	-4.80E-06	1.22E-08	0.965
5	150395351	150917796	368	4.58E-05	6.11E-09	0.558
5	150918158	152360494	593	1.90E-04	8.76E-09	0.042
5	147665223	150393107	1251	2.99E-05	1.90E-08	0.828
5	152361546	152864614	177	-4.53E-06	2.42E-09	0.927
5	152867348	153494463	316	2.06E-05	3.63E-09	0.733
5	153495193	154448863	413	-4.17E-05	7.15E-09	0.622
5	154449856	155290211	358	4.33E-05	5.36E-09	0.554
5	155290613	156628287	483	-3.04E-05	7.63E-09	0.728
5	156629754	157197141	306	-4.13E-05	4.52E-09	0.539
5	157199547	158491655	551	4.13E-07	7.99E-09	0.996
5	158492279	159135376	279	6.89E-05	5.16E-09	0.337
5	159136348	160908680	774	4.34E-05	1.36E-08	0.709
5	160910101	161688620	224	-2.14E-05	3.33E-09	0.711
5	161688913	162741298	358	7.35E-05	5.65E-09	0.328
5	162742614	163769136	431	1.20E-04	7.43E-09	0.163
5	163769738	164944949	399	5.39E-05	6.30E-09	0.497
5	164947504	165638940	255	1.06E-05	6.47E-09	0.895

5	165642395	166432731	347	-9.29E-05	7.51E-09	0.284
5	166433367	167760730	549	-2.89E-05	1.07E-08	0.780
5	167761922	169124478	717	1.84E-04	1.43E-08	0.123
5	169125063	170889081	951	-5.53E-05	1.65E-08	0.667
5	170891834	171772889	429	5.01E-06	8.53E-09	0.957
5	171775715	172376903	328	6.19E-06	7.48E-09	0.943
5	172377031	173611384	633	2.71E-05	1.27E-08	0.810
5	173613085	174468408	480	1.68E-04	1.71E-08	0.200
5	174468624	176128648	690	4.14E-05	1.47E-08	0.733
5	176129392	177991305	614	-1.20E-04	1.44E-08	0.316
5	177992368	178410668	199	4.52E-05	4.26E-09	0.489
5	178410839	179245749	390	-1.40E-06	8.01E-09	0.988
5	179245856	180007693	315	3.20E-05	6.83E-09	0.699
5	180008456	180790950	242	-2.53E-05	6.01E-09	0.744
6	63979	824529	327	4.67E-05	9.15E-09	0.626
6	826312	1582647	522	-7.00E-05	1.48E-08	0.565
6	1582926	2890836	717	1.07E-04	1.62E-08	0.402
6	2891016	4315210	770	-1.72E-04	1.84E-08	0.205
6	4317398	5056146	425	6.60E-05	9.03E-09	0.488
6	5057399	6246639	732	1.33E-04	1.69E-08	0.307
6	6246984	7328580	685	1.67E-04	1.95E-08	0.233
6	7329417	8791321	740	-4.12E-06	1.39E-08	0.972
6	8791421	9760323	373	-8.40E-06	9.51E-09	0.931
6	9764413	11234164	726	-1.57E-06	1.93E-08	0.991
6	11235682	12183952	542	8.98E-05	1.10E-08	0.392
6	12186102	13388480	617	-4.69E-05	1.20E-08	0.668
6	13389521	14618107	554	9.84E-05	1.14E-08	0.358
6	14619148	15998368	595	-5.75E-06	1.24E-08	0.959
6	15998406	17067160	545	1.84E-04	1.49E-08	0.132
6	17069966	19203108	878	6.59E-05	1.68E-08	0.611
6	19203782	20228741	524	1.53E-05	1.31E-08	0.893
6	20231516	22049758	823	-3.58E-06	2.13E-08	0.980
6	22052425	23495209	747	-1.01E-04	1.91E-08	0.466
6	23495260	23935020	233	6.56E-05	4.69E-09	0.339
6	23935352	24799440	436	-3.03E-05	1.11E-08	0.774
6	24803038	25483400	411	-1.72E-05	8.78E-09	0.855
6	25484295	26774948	727	-1.53E-04	1.47E-08	0.207

6	26778827	28669698	609	-7.61E-05	1.05E-08	0.457
6	32900018	33863002	400	-1.15E-04	1.36E-08	0.323
6	33863662	35451187	511	-4.87E-05	8.62E-09	0.600
6	35452346	36608267	395	6.58E-06	7.14E-09	0.938
6	38687185	39021712	205	-2.41E-05	4.14E-09	0.708
6	36611164	38686222	996	1.59E-04	2.12E-08	0.275
6	39022698	40344526	674	1.16E-04	1.37E-08	0.321
6	40345542	42105449	884	-1.77E-04	1.92E-08	0.201
6	42105555	43957473	632	2.43E-05	1.50E-08	0.843
6	43959116	45787468	700	3.33E-05	1.50E-08	0.785
6	45788111	46556325	327	-5.87E-05	5.84E-09	0.442
6	46560003	47885216	680	-6.58E-05	1.17E-08	0.543
6	47887063	49494241	468	-5.70E-05	7.06E-09	0.497
6	49494933	50406476	309	-8.50E-05	5.80E-09	0.264
6	50406575	51703012	476	-1.04E-05	8.54E-09	0.910
6	51704668	53402679	834	-1.03E-04	1.70E-08	0.431
6	53405127	54291407	455	-4.73E-05	7.85E-09	0.593
6	54293261	56103747	744	3.76E-05	1.27E-08	0.739
6	56104522	57082026	352	3.66E-05	5.59E-09	0.624
6	57085542	58084365	149	5.21E-06	2.67E-09	0.920
6	58139505	62691733	301	1.25E-05	4.55E-09	0.853
6	62692409	63712779	264	5.01E-06	3.61E-09	0.934
6	63713620	65760585	508	-3.13E-05	7.06E-09	0.710
6	65762553	67028107	491	-1.50E-05	8.41E-09	0.870
6	67029501	67556729	233	2.31E-05	4.59E-09	0.733
6	67557245	68546601	377	7.78E-05	7.00E-09	0.352
6	68546995	69295414	301	-1.55E-05	6.99E-09	0.853
6	69295526	70359936	399	-6.68E-06	6.72E-09	0.935
6	70360869	72448598	881	-1.50E-05	1.60E-08	0.906
6	72449421	73170712	296	2.89E-05	5.75E-09	0.703
6	73171594	75064563	682	2.56E-05	1.27E-08	0.821
6	75065279	75843360	172	-1.25E-05	3.42E-09	0.831
6	75843845	77257094	415	1.65E-05	6.18E-09	0.833
6	77257275	78957547	677	-2.36E-05	1.12E-08	0.823
6	78958134	79861169	296	-1.51E-05	4.72E-09	0.826
6	79862483	80556359	340	-1.36E-05	7.15E-09	0.872
6	80557170	82387384	657	8.53E-05	1.01E-08	0.397

6	82387920	83904869	519	7.32E-05	9.54E-09	0.454
6	83908232	86040334	810	2.82E-05	1.45E-08	0.815
6	86040497	87138115	340	-1.12E-05	5.40E-09	0.879
6	87140364	88532072	484	-1.79E-04	8.92E-09	0.058
6	88533324	89668547	467	1.16E-04	1.09E-08	0.268
6	89673861	91400644	815	9.10E-05	1.70E-08	0.485
6	91401234	93426186	759	4.63E-05	1.57E-08	0.711
6	93429797	94919277	573	8.71E-05	1.12E-08	0.411
6	94920611	97093037	660	3.95E-05	1.22E-08	0.720
6	97093295	98893182	513	-6.46E-05	8.94E-09	0.495
6	98893830	100286683	584	-2.87E-05	1.13E-08	0.787
6	100287245	101862923	464	-4.21E-05	7.98E-09	0.637
6	101863563	102887537	347	8.63E-06	7.24E-09	0.919
6	102888281	104211089	445	-8.92E-05	7.24E-09	0.295
6	104211475	105752435	548	-5.37E-05	9.86E-09	0.589
6	105754306	106921804	533	-1.09E-04	1.36E-08	0.351
6	106922281	108322638	653	-1.76E-05	1.50E-08	0.886
6	108325624	110654066	761	-1.05E-04	1.81E-08	0.434
6	110654605	111390344	273	6.26E-06	5.75E-09	0.934
6	111391537	114218733	1067	8.73E-05	1.95E-08	0.532
6	114220361	116130955	594	-9.34E-05	1.08E-08	0.368
6	116130983	117566682	495	-5.21E-05	1.00E-08	0.603
6	117567468	119071713	561	-1.26E-05	1.02E-08	0.900
6	119075856	120628075	540	-5.13E-05	1.03E-08	0.614
6	120628214	121673627	338	-1.13E-05	5.44E-09	0.878
6	121674337	123104703	522	-3.79E-06	8.96E-09	0.968
6	123105676	123889346	320	-5.91E-06	7.02E-09	0.944
6	123890282	125333797	656	4.57E-06	1.48E-08	0.970
6	125336799	128759251	899	-8.73E-05	1.59E-08	0.489
6	128759755	130538497	786	5.30E-05	1.68E-08	0.682
6	130540383	131972731	564	2.26E-04	1.09E-08	0.030
6	131973932	133867802	872	2.25E-05	1.89E-08	0.870
6	133869809	136186007	801	-4.71E-05	2.07E-08	0.744
6	136186368	137811295	507	3.30E-05	9.78E-09	0.738
6	137813095	139532424	747	6.99E-05	1.75E-08	0.597
6	139533572	142036032	706	-1.13E-04	1.31E-08	0.324
6	142036912	143719278	602	-7.35E-05	1.14E-08	0.491

6	146665424	148179283	590	4.95E-06	1.13E-08	0.963
6	143720467	146663272	1046	1.64E-05	1.89E-08	0.905
6	148180191	148917941	479	-6.91E-05	1.36E-08	0.553
6	148919158	150298842	637	5.82E-05	1.10E-08	0.579
6	150299299	151097980	538	-1.58E-04	1.43E-08	0.186
6	151100484	151929942	489	7.59E-05	1.29E-08	0.504
6	151930249	153550147	880	-2.93E-04	3.43E-08	0.114
6	153551177	155228511	744	-5.57E-05	1.72E-08	0.672
6	155229566	156756457	767	1.11E-04	1.82E-08	0.409
6	156761338	157398853	255	-9.81E-05	5.21E-09	0.174
6	157402041	158192951	273	1.61E-05	6.85E-09	0.846
6	158193244	159318774	547	-9.50E-05	9.51E-09	0.330
6	159319812	159792458	251	-5.68E-06	3.85E-09	0.927
6	159793334	161123451	690	-2.85E-04	6.82E-08	0.275
6	161123771	162298351	679	-1.03E-04	2.04E-08	0.472
6	162300151	163277414	507	4.30E-05	1.02E-08	0.670
6	163277858	164003180	378	1.24E-04	8.63E-09	0.180
6	164006012	165242243	674	-3.41E-06	1.44E-08	0.977
6	165246062	166191910	571	9.40E-05	1.35E-08	0.418
6	166193932	167177733	678	2.21E-05	1.64E-08	0.863
6	167178790	168548525	625	9.18E-05	1.26E-08	0.413
6	168549335	169231503	389	-1.72E-05	9.16E-09	0.857
6	169231937	171049558	872	-4.79E-05	1.70E-08	0.713
7	20608	1490775	409	-2.06E-05	8.89E-09	0.827
7	1493090	2479029	317	-1.67E-04	1.27E-08	0.140
7	2480729	2930941	207	4.08E-05	4.36E-09	0.537
7	2931676	3736942	499	5.10E-06	1.06E-08	0.961
7	3738087	4783224	511	2.04E-05	1.10E-08	0.846
7	4783723	5412932	213	4.07E-06	6.11E-09	0.958
7	5414602	6912527	397	1.77E-04	8.82E-09	0.059
7	7168493	7679885	293	-1.02E-04	6.08E-09	0.191
7	7680697	8475147	543	4.78E-05	1.39E-08	0.686
7	8476787	9223721	524	-5.19E-05	1.20E-08	0.635
7	9223762	9893744	340	2.63E-05	8.18E-09	0.771
7	9894421	11298815	623	-1.19E-05	1.20E-08	0.914
7	11299552	12687871	878	-1.78E-05	1.88E-08	0.897
7	12689322	13674908	507	-3.13E-05	1.06E-08	0.761

7	13675094	14941781	683	5.26E-05	1.52E-08	0.670
7	14942134	16480989	759	-1.85E-05	1.48E-08	0.879
7	16481863	17277084	484	-6.23E-05	1.27E-08	0.580
7	17279294	18861958	710	-1.03E-04	1.47E-08	0.393
7	18863387	19975992	510	-7.56E-05	9.47E-09	0.437
7	19978609	21712233	877	-2.18E-04	3.03E-08	0.210
7	21712615	22811384	707	2.77E-05	1.67E-08	0.830
7	22811921	24389035	702	1.64E-04	1.84E-08	0.227
7	24389147	24980408	322	-7.34E-07	7.06E-09	0.993
7	24982494	26300992	674	-2.45E-04	2.32E-08	0.107
7	26302668	28038375	768	5.28E-04	1.21E-07	0.129
7	28039590	28710596	382	-7.21E-05	1.46E-08	0.551
7	28710857	30171413	826	3.74E-05	1.75E-08	0.777
7	30172195	31608495	659	2.87E-05	1.39E-08	0.808
7	31609317	33150311	749	-2.86E-05	1.40E-08	0.808
7	33151181	34939884	766	9.63E-05	1.45E-08	0.424
7	34940039	35614036	299	-3.80E-05	5.45E-09	0.607
7	35615611	37353302	858	-1.12E-04	2.01E-08	0.430
7	37354091	38159855	465	-3.45E-05	9.41E-09	0.722
7	38160558	39534540	677	-1.14E-04	1.40E-08	0.335
7	39535570	40925323	371	2.35E-07	6.58E-09	0.998
7	40926906	42494824	653	6.17E-05	1.36E-08	0.596
7	42495555	44971837	905	1.42E-05	1.45E-08	0.906
7	44972152	46665658	694	7.42E-05	1.27E-08	0.511
7	46667873	47279128	336	-1.04E-05	6.36E-09	0.896
7	47281095	49130572	862	2.32E-05	1.77E-08	0.861
7	49131824	49821750	300	3.90E-05	5.28E-09	0.591
7	49822437	51674880	774	-4.17E-06	1.40E-08	0.972
7	51675218	52274523	293	-3.35E-05	6.60E-09	0.680
7	52275656	53175065	478	-7.19E-06	9.15E-09	0.940
7	53348777	54707110	519	8.62E-06	8.71E-09	0.926
7	54707337	55641707	450	-6.44E-05	9.06E-09	0.499
7	55641798	57382254	384	5.19E-05	6.69E-09	0.525
7	57382271	62556935	172	1.08E-05	2.34E-09	0.823
7	62557202	64193303	280	-1.09E-05	4.35E-09	0.869
7	64699988	65570171	150	7.78E-06	1.87E-09	0.857
7	65570310	66453460	218	2.65E-05	1.91E-09	0.545

7	66454071	67369810	338	-7.62E-06	6.65E-09	0.926
7	67370861	68125403	315	4.81E-05	7.44E-09	0.577
7	68126378	68561421	188	-4.42E-05	3.65E-09	0.464
7	68562932	69806895	289	1.56E-05	4.20E-09	0.810
7	69808153	71204524	593	-6.71E-05	1.47E-08	0.580
7	71205507	71873571	248	-4.29E-05	4.18E-09	0.507
7	71874997	73996533	396	1.79E-06	6.74E-09	0.983
7	73997700	76550299	360	-3.26E-04	9.31E-09	0.001
7	76551983	77101532	179	-3.97E-05	3.62E-09	0.509
7	77102731	77953248	410	2.25E-05	7.24E-09	0.792
7	77954163	78839317	553	-1.43E-04	1.19E-08	0.190
7	78843289	80546745	613	-2.53E-05	1.06E-08	0.807
7	80547047	82758529	882	4.18E-05	1.91E-08	0.763
7	82758644	83959105	533	8.55E-05	9.94E-09	0.391
7	83959751	85570789	448	-1.10E-05	6.58E-09	0.892
7	85571372	86341446	218	-3.02E-05	3.28E-09	0.598
7	88732470	89270556	217	-7.44E-05	3.90E-09	0.234
7	86345013	88732142	836	3.64E-05	1.26E-08	0.746
7	89271899	91173749	772	2.32E-05	1.34E-08	0.841
7	91174778	93339775	616	-1.82E-05	9.24E-09	0.850
7	93340137	95171731	630	1.31E-04	1.23E-08	0.238
7	95419256	96611864	459	-1.60E-05	7.86E-09	0.857
7	96613246	98151455	495	4.59E-05	9.78E-09	0.643
7	98152191	100555361	664	1.13E-04	1.32E-08	0.324
7	100606141	101730309	387	-3.90E-05	9.75E-09	0.693
7	101731567	103193331	317	-1.12E-05	5.49E-09	0.880
7	103193651	104157576	550	2.72E-05	1.12E-08	0.797
7	104158491	105425027	480	-3.78E-06	7.43E-09	0.965
7	105427565	106052506	320	6.24E-05	7.93E-09	0.483
7	106052873	107305328	456	6.21E-05	8.40E-09	0.498
7	107306421	108375939	543	-7.07E-05	9.08E-09	0.458
7	108376173	109584769	351	-1.38E-05	5.84E-09	0.857
7	109587777	111447153	592	-4.19E-05	9.87E-09	0.673
7	111450919	113336970	590	7.13E-06	1.13E-08	0.946
7	113337327	116315205	824	-4.93E-05	1.37E-08	0.673
7	116317043	118347634	568	8.57E-05	9.56E-09	0.381
7	118348359	121094386	572	1.69E-05	7.90E-09	0.849

7	121094460	122368663	412	4.56E-05	7.80E-09	0.606
7	122369853	124154451	639	-7.72E-05	9.49E-09	0.428
7	124155319	125386718	379	4.92E-05	5.19E-09	0.495
7	125387168	126516683	442	-6.39E-05	6.92E-09	0.442
7	126517818	127905983	588	3.50E-05	8.61E-09	0.706
7	127907054	128554075	209	-2.28E-05	4.11E-09	0.723
7	128555262	130074230	546	-5.64E-05	9.91E-09	0.571
7	130075370	130797123	249	-1.58E-05	6.19E-09	0.841
7	130797776	131856855	497	-1.95E-04	1.13E-08	0.067
7	131857419	134306672	895	1.17E-04	1.44E-08	0.328
7	134307114	135464701	503	1.22E-04	9.28E-09	0.205
7	135466022	137137511	701	-3.07E-06	1.34E-08	0.979
7	137138736	138742981	585	-3.44E-06	1.48E-08	0.977
7	138744100	141106552	843	1.89E-05	1.46E-08	0.876
7	141109797	142655113	558	1.09E-05	8.79E-09	0.907
7	142655717	144274735	430	-2.20E-05	7.32E-09	0.797
7	144276424	146018520	551	-1.95E-05	1.02E-08	0.846
7	146023023	147204086	503	9.33E-05	8.88E-09	0.322
7	147204549	148619685	683	8.25E-05	1.31E-08	0.471
7	148620581	150491084	651	6.41E-05	1.21E-08	0.560
7	150491851	151390799	462	5.32E-05	1.11E-08	0.613
7	151391044	152409155	286	2.17E-05	1.08E-08	0.834
7	152410248	153080056	287	-8.01E-05	7.70E-09	0.361
7	153081698	153934166	317	8.93E-06	7.40E-09	0.917
7	154964316	155379409	238	8.40E-05	8.78E-09	0.370
7	153934210	154963787	619	1.76E-04	1.39E-08	0.135
7	155380353	156814410	650	-3.32E-05	1.51E-08	0.787
7	156815867	158029340	505	1.70E-04	1.15E-08	0.113
7	158030463	158759939	285	-1.65E-05	4.71E-09	0.809
7	158760677	159127587	134	6.20E-06	4.19E-09	0.924
8	246934	1162157	487	3.49E-05	1.31E-08	0.760
8	1162454	1625424	272	-1.16E-05	7.53E-09	0.894
8	1625732	2042364	302	7.23E-05	9.88E-09	0.467
8	2043187	2680255	316	5.52E-05	9.59E-09	0.573
8	2681051	3358423	619	-1.11E-05	1.72E-08	0.933
8	3358433	3584832	296	-1.32E-05	7.40E-09	0.878
8	3585058	4071438	625	6.12E-05	1.50E-08	0.616

8	4071516	4274957	255	1.98E-05	6.38E-09	0.804
8	4275286	4479596	219	1.66E-05	7.51E-09	0.848
8	4479836	4754328	302	4.74E-05	7.12E-09	0.574
8	4755088	5146808	311	-6.52E-05	7.70E-09	0.457
8	5147734	5781592	595	-9.52E-05	1.43E-08	0.426
8	5781947	6017670	226	-1.03E-04	4.81E-09	0.137
8	6018292	6350873	272	-2.35E-05	5.73E-09	0.756
8	6352358	7793703	489	-1.36E-05	1.25E-08	0.903
8	7793837	8735213	411	-1.46E-06	8.82E-09	0.988
8	10117411	10535826	286	-1.04E-06	7.64E-09	0.991
8	8736386	10117293	799	3.21E-05	1.83E-08	0.813
8	10537940	11249261	408	-4.74E-05	7.99E-09	0.596
8	11250848	11836622	364	-5.28E-05	1.02E-08	0.601
8	11838355	12975295	284	1.19E-05	7.03E-09	0.888
8	12975578	13940652	686	-6.11E-05	1.56E-08	0.625
8	13942500	14876660	522	-1.02E-04	1.09E-08	0.332
8	14877288	15273777	320	-1.92E-05	6.24E-09	0.808
8	15274608	16087631	534	-3.86E-05	1.24E-08	0.728
8	16087729	17579518	802	-1.57E-05	1.92E-08	0.910
8	17581106	18670645	721	-1.66E-04	1.51E-08	0.178
8	18670908	19490180	560	-1.19E-04	1.34E-08	0.304
8	21022075	21402658	257	-3.49E-05	5.64E-09	0.643
8	19491428	21021401	888	1.65E-04	2.18E-08	0.265
8	21404381	22539316	482	6.92E-05	9.22E-09	0.471
8	22539641	23548146	588	-5.17E-05	5.44E-08	0.825
8	23548749	24456913	316	2.07E-05	8.04E-09	0.817
8	24457122	25461315	424	2.69E-05	7.44E-09	0.755
8	25461344	26681450	716	2.34E-05	2.01E-08	0.869
8	26681799	27404354	421	2.99E-05	8.46E-09	0.745
8	27405207	27759126	213	-1.56E-05	3.65E-09	0.797
8	27759839	29277590	622	-1.34E-04	1.25E-08	0.230
8	31947880	32448740	282	-6.26E-05	6.11E-09	0.424
8	29277652	31947675	952	-3.90E-05	1.95E-08	0.780
8	32449533	33980486	355	-3.35E-05	5.77E-09	0.659
8	33981742	35313077	399	2.45E-05	5.62E-09	0.744
8	35314837	37375458	435	-3.23E-05	7.59E-09	0.711
8	37376914	39779989	596	-1.55E-04	1.25E-08	0.165

8	39781444	40415799	338	1.29E-06	7.41E-09	0.988
8	40417433	41718356	592	-8.43E-05	1.32E-08	0.463
8	41719750	42501174	186	6.28E-05	3.25E-09	0.271
8	42501717	49028667	387	-6.24E-06	4.74E-09	0.928
8	49038732	52156842	813	1.29E-05	1.16E-08	0.905
8	52157415	54115712	718	-2.33E-05	1.27E-08	0.836
8	54116775	55914038	657	1.78E-05	1.34E-08	0.878
8	55914314	57653028	655	5.60E-05	1.30E-08	0.623
8	57655067	58674153	403	-8.10E-05	7.40E-09	0.347
8	58675131	59568410	383	1.12E-05	8.14E-09	0.901
8	59570176	61809929	767	-1.94E-04	1.45E-08	0.107
8	61810694	63710298	770	-1.20E-04	1.45E-08	0.319
8	63711348	65193342	460	-2.93E-05	8.56E-09	0.751
8	65195953	66024947	259	-2.83E-05	4.73E-09	0.681
8	66025862	67192642	386	-5.49E-05	6.93E-09	0.509
8	67193708	69108839	502	-1.32E-05	1.00E-08	0.895
8	69111244	70003448	401	-1.20E-05	9.41E-09	0.902
8	70003607	72013005	726	9.30E-05	1.45E-08	0.440
8	72014069	72749564	426	1.14E-04	1.06E-08	0.267
8	72751469	73423214	352	-1.33E-04	7.95E-09	0.136
8	73423786	74996061	687	2.31E-05	1.39E-08	0.845
8	74998868	76294909	508	6.11E-05	1.01E-08	0.543
8	76295292	78015335	544	-7.52E-06	9.76E-09	0.939
8	78015611	78632215	197	2.78E-06	3.34E-09	0.962
8	78632893	79831888	374	4.70E-06	5.85E-09	0.951
8	79832211	80654655	272	2.68E-05	5.49E-09	0.717
8	80656206	81330540	223	-7.12E-06	4.72E-09	0.918
8	81331077	83726996	809	-7.56E-05	1.62E-08	0.552
8	83727741	85093121	385	2.47E-05	7.58E-09	0.777
8	85094979	86979097	352	6.73E-05	5.23E-09	0.352
8	86990451	87562786	212	2.01E-05	3.23E-09	0.724
8	87563878	89083319	540	-3.87E-05	1.01E-08	0.700
8	89083979	90605445	456	2.84E-05	7.49E-09	0.743
8	90607207	92342283	521	-3.24E-05	7.95E-09	0.717
8	92344138	94269630	544	8.77E-08	1.13E-08	0.999
8	94272436	95810190	578	-5.84E-05	1.23E-08	0.598
8	95810772	96533604	390	-2.62E-06	8.53E-09	0.977

8	96534326	97318660	395	5.81E-06	8.92E-09	0.951
8	97320605	98867870	621	-7.55E-06	1.20E-08	0.945
8	98869940	101450242	829	2.03E-05	1.30E-08	0.859
8	101451169	102937063	655	1.46E-04	1.47E-08	0.228
8	102944509	103730879	375	-4.51E-06	9.46E-09	0.963
8	103970185	105389708	565	2.29E-06	9.93E-09	0.982
8	105391381	107409808	782	6.88E-05	1.53E-08	0.578
8	107410067	108108114	324	2.49E-05	5.96E-09	0.747
8	108115161	108646193	244	-2.90E-05	5.51E-09	0.696
8	108646968	110761074	742	-4.63E-05	1.25E-08	0.679
8	110761776	112611637	402	2.15E-05	6.62E-09	0.792
8	112612842	114393054	481	3.16E-06	6.93E-09	0.970
8	114394256	116095059	613	2.97E-05	1.01E-08	0.767
8	116095815	117130004	272	9.52E-05	6.59E-09	0.241
8	117131947	118622352	563	1.12E-04	1.17E-08	0.301
8	118623844	120216957	717	1.31E-04	1.50E-08	0.284
8	120217356	121767384	601	6.38E-05	1.02E-08	0.528
8	121770585	123545992	691	1.67E-04	1.51E-08	0.174
8	123547148	124483938	403	9.73E-05	9.56E-09	0.320
8	124486753	125247268	404	-3.53E-05	9.52E-09	0.717
8	125248576	126268046	461	-5.85E-05	1.06E-08	0.569
8	126269921	127434945	507	-1.64E-05	1.08E-08	0.875
8	127436003	128162974	337	4.21E-05	1.67E-07	0.918
8	128166556	128542444	193	-3.13E-05	1.59E-07	0.937
8	128543956	129297518	432	-2.31E-04	4.47E-08	0.275
8	129297807	130379330	412	-4.01E-05	7.95E-09	0.653
8	130380171	130812339	230	-6.14E-05	4.98E-09	0.384
8	130814803	131553214	284	-3.10E-05	5.36E-09	0.672
8	131553327	132358990	430	7.22E-05	9.23E-09	0.452
8	132359241	133454722	553	3.59E-05	1.07E-08	0.729
8	133455530	134566686	734	4.25E-05	1.53E-08	0.731
8	134567348	135269827	423	1.73E-05	1.09E-08	0.868
8	135270868	136209239	515	-1.06E-04	1.13E-08	0.319
8	136209325	137306730	483	-6.10E-05	8.15E-09	0.499
8	137307361	138223632	346	2.34E-07	6.32E-09	0.998
8	138224181	139194661	572	5.14E-05	1.12E-08	0.628
8	139195331	140093896	520	2.17E-05	1.30E-08	0.849

8	140094627	141355502	730	8.79E-05	1.60E-08	0.487
8	143750619	146303789	713	2.45E-05	1.89E-08	0.859
8	141358681	143750205	919	-8.02E-05	2.04E-08	0.574
9	342841	1006606	532	-3.69E-06	1.07E-08	0.971
9	1007923	1792147	679	2.73E-05	1.26E-08	0.807
9	1792277	2665240	685	-8.04E-05	1.57E-08	0.521
9	2666964	3348816	358	-3.79E-04	1.19E-08	0.001
9	4380096	4590305	142	-5.69E-06	3.41E-09	0.922
9	3349487	4378727	698	9.10E-06	1.66E-08	0.944
9	4591655	5273194	341	1.27E-04	5.53E-09	0.087
9	5273896	6144065	383	1.49E-06	6.11E-09	0.985
9	6144333	7232230	569	-5.10E-05	1.03E-08	0.616
9	7232510	7757891	465	-4.69E-05	9.14E-09	0.624
9	7758579	8235898	325	4.39E-05	6.16E-09	0.576
9	9375194	9793342	253	-3.67E-05	4.88E-09	0.599
9	8236122	9374664	803	1.49E-04	1.80E-08	0.265
9	9793846	10358286	350	-5.26E-05	6.70E-09	0.520
9	10358672	10879071	263	3.35E-05	5.67E-09	0.656
9	10879188	11616822	305	-2.57E-05	5.66E-09	0.733
9	11884425	12274220	180	-2.60E-05	3.03E-09	0.637
9	12274774	12778224	274	-1.76E-05	4.90E-09	0.801
9	12780295	13797055	477	-1.44E-05	9.32E-09	0.882
9	13797496	14712257	603	-1.98E-06	1.46E-08	0.987
9	14714681	16054406	644	-2.12E-04	1.06E-08	0.039
9	16056127	16833797	538	-5.03E-05	1.13E-08	0.636
9	16836011	17627988	363	1.47E-05	5.42E-09	0.842
9	17628726	18659920	604	-2.53E-05	1.05E-08	0.805
9	18660695	19129349	353	-1.36E-04	7.22E-09	0.111
9	19456098	20324812	481	2.01E-05	9.02E-09	0.833
9	20324848	21549638	502	9.13E-06	7.69E-09	0.917
9	21550959	22968575	596	-7.06E-05	9.87E-09	0.477
9	22968789	24154728	439	3.20E-05	1.03E-08	0.753
9	24155142	25425160	409	8.26E-05	8.62E-09	0.374
9	25425511	26317104	404	6.19E-05	9.52E-09	0.526
9	26317973	27335346	535	-6.11E-05	1.04E-08	0.549
9	27336207	28252383	496	3.56E-06	1.08E-08	0.973
9	28253887	29622939	659	2.07E-05	1.02E-08	0.838

9	29623053	30819576	414	5.01E-05	5.11E-09	0.483
9	30821676	31836697	373	-8.64E-05	5.50E-09	0.244
9	31837040	32249649	172	1.72E-06	2.33E-09	0.972
9	32250247	33186788	463	-9.45E-05	7.16E-09	0.264
9	33187897	34769004	521	3.85E-05	8.00E-09	0.667
9	36714252	37678932	456	2.30E-05	8.26E-09	0.800
9	34771534	36712124	645	1.05E-04	9.96E-09	0.291
9	37679713	42220384	538	1.50E-04	1.03E-08	0.140
9	69576527	71968825	444	-3.63E-05	7.49E-09	0.675
9	71969482	73398448	622	-1.34E-05	8.49E-09	0.884
9	73399750	73916072	309	6.19E-06	4.41E-09	0.926
9	73916583	74600739	231	6.08E-06	4.41E-09	0.927
9	74600822	75517582	356	-4.31E-05	4.74E-09	0.531
9	75519251	76785168	378	3.63E-05	5.76E-09	0.633
9	76786297	78214833	562	4.93E-05	9.77E-09	0.618
9	78215299	78900183	469	-2.29E-05	1.30E-08	0.841
9	81066078	81860861	384	-5.72E-05	7.12E-09	0.498
9	78901112	81064897	966	-4.69E-06	1.71E-08	0.971
9	81863722	82348771	246	-4.04E-05	4.01E-09	0.523
9	82349606	83041982	352	9.27E-06	7.00E-09	0.912
9	83043376	83455568	219	2.94E-05	3.68E-09	0.628
9	83455867	84388566	420	-3.16E-05	7.87E-09	0.722
9	84389168	86180165	683	9.03E-05	1.20E-08	0.409
9	87571959	87811640	150	1.10E-05	2.40E-09	0.822
9	86181461	87571563	628	-1.47E-04	9.30E-09	0.126
9	87813612	88867151	355	6.50E-05	5.36E-09	0.375
9	88869630	89948499	516	-1.32E-06	9.14E-09	0.989
9	89949778	90198251	198	2.21E-05	2.92E-09	0.682
9	90198375	90893610	336	-2.22E-05	6.29E-09	0.780
9	90893774	91608628	247	3.95E-06	4.27E-09	0.952
9	91610543	92342927	327	-1.92E-05	6.23E-09	0.808
9	92443570	93441674	461	1.60E-04	8.16E-09	0.076
9	93442205	94163515	361	1.14E-04	7.05E-09	0.176
9	94164373	95854872	626	1.72E-05	7.26E-09	0.840
9	95855100	97719592	729	5.31E-06	1.03E-08	0.958
9	97720818	98889276	456	2.09E-05	6.63E-09	0.798
9	98891743	100917602	625	5.29E-05	8.14E-09	0.558

9	100919318	101473404	376	-3.20E-05	7.52E-09	0.713
9	103544757	103969076	179	3.88E-06	1.86E-09	0.928
9	101474837	103543692	676	-5.52E-05	8.20E-09	0.542
9	103969339	104552711	293	4.20E-05	4.96E-09	0.551
9	104553938	105041375	303	-1.87E-05	5.22E-09	0.795
9	105041756	105743445	320	1.53E-05	4.83E-09	0.826
9	105744075	106487777	333	2.53E-05	5.67E-09	0.737
9	106488291	107545903	496	2.03E-05	5.51E-09	0.784
9	107546901	108751162	555	1.84E-05	8.98E-09	0.846
9	108754828	109972524	499	-4.36E-05	1.46E-08	0.718
9	109974206	111137001	673	1.19E-05	1.73E-08	0.928
9	111138232	111997222	549	6.66E-05	8.40E-09	0.467
9	112001473	112593531	326	1.34E-04	5.42E-09	0.070
9	112594858	113255402	400	-4.18E-05	8.60E-09	0.652
9	113255544	114077164	462	2.27E-05	8.09E-09	0.801
9	114078103	114828332	329	-1.92E-05	5.36E-09	0.793
9	114830330	115391053	230	1.51E-06	2.54E-09	0.976
9	115393806	116148630	399	-2.53E-05	7.06E-09	0.764
9	116149967	117306807	686	-1.44E-04	1.29E-08	0.205
9	117307880	118569429	682	3.80E-05	1.10E-08	0.717
9	118570426	120030166	743	4.82E-05	1.33E-08	0.676
9	121009165	121318886	151	1.83E-05	2.48E-09	0.713
9	120030269	121008844	499	9.80E-05	8.75E-09	0.295
9	121319662	122258204	434	-4.33E-05	6.85E-09	0.601
9	122258577	124870612	937	-6.92E-05	1.30E-08	0.543
9	124871322	125542861	333	-9.61E-05	6.30E-09	0.226
9	125545194	126926376	448	-4.05E-05	6.55E-09	0.616
9	126927204	128926989	624	-8.23E-05	6.91E-09	0.322
9	128927088	129477470	292	-5.11E-05	6.64E-09	0.530
9	129477886	131429495	582	-1.61E-05	7.42E-09	0.851
9	131430602	132891863	504	-3.69E-05	8.95E-09	0.696
9	132895131	133640777	304	-2.54E-05	7.97E-09	0.776
9	133642918	134639655	431	-5.85E-06	7.08E-09	0.945
9	134640928	135401279	345	5.04E-05	7.34E-09	0.556
9	135401721	136417159	500	1.32E-04	9.22E-09	0.168
9	136429816	136979539	317	-7.25E-05	7.80E-09	0.411
9	136979744	137928846	495	8.39E-05	1.28E-08	0.458

9	137929853	138817142	510	-9.90E-05	1.36E-08	0.396
9	138817178	141146684	716	-3.11E-05	1.25E-08	0.780
10	60969	747115	150	-5.03E-05	2.90E-09	0.350
10	747772	1330771	280	5.32E-05	5.24E-09	0.462
10	1331955	1609370	171	-2.09E-05	3.96E-09	0.741
10	1610839	2104472	389	-2.50E-05	8.32E-09	0.784
10	2106418	3143283	688	-5.19E-05	1.45E-08	0.666
10	3144251	3785605	441	5.73E-05	8.93E-09	0.545
10	5359010	6316910	614	6.70E-05	1.22E-08	0.544
10	3786476	5358542	954	2.05E-04	1.88E-08	0.136
10	6318458	7639684	881	1.07E-04	2.22E-08	0.471
10	9433090	10540465	440	2.43E-05	8.05E-09	0.787
10	7639932	9432277	1108	-5.10E-05	2.14E-08	0.727
10	10542217	12092029	827	-4.55E-05	1.74E-08	0.730
10	12092208	12798954	370	-3.97E-05	9.30E-09	0.681
10	12799286	13718384	597	-1.64E-04	1.29E-08	0.150
10	13719970	14411956	499	-4.10E-05	1.21E-08	0.709
10	14412084	15303527	488	2.56E-05	1.18E-08	0.814
10	15303983	16443442	476	1.31E-05	7.82E-09	0.883
10	16444693	17591626	663	-8.00E-05	1.33E-08	0.489
10	17591919	19202731	694	1.33E-05	1.20E-08	0.903
10	19203300	20131614	450	-1.18E-05	7.37E-09	0.890
10	20132516	21243558	679	-3.72E-05	1.27E-08	0.742
10	21244087	23696890	630	-4.67E-05	1.19E-08	0.669
10	23698033	25414215	686	-5.86E-05	1.12E-08	0.581
10	25416598	26631987	555	1.68E-05	8.83E-09	0.858
10	26633775	27667904	403	7.99E-05	6.63E-09	0.327
10	27668052	28734655	508	-1.14E-04	9.75E-09	0.246
10	28735045	29174963	234	-3.01E-05	3.86E-09	0.628
10	29175101	29961838	534	8.31E-05	1.16E-08	0.441
10	29962511	30653567	387	3.03E-06	8.16E-09	0.973
10	30654528	31479103	433	2.95E-07	7.86E-09	0.997
10	31479422	33656047	778	-9.52E-05	1.11E-08	0.366
10	33656119	36017592	829	-6.52E-05	1.24E-08	0.559
10	36020445	36884906	379	1.14E-05	7.36E-09	0.894
10	36885921	37845308	312	8.38E-06	5.11E-09	0.907
10	38367233	43220539	214	3.58E-07	2.55E-09	0.994

10	43222372	43605902	162	-1.28E-05	3.57E-09	0.830
10	43606687	45247184	692	4.27E-05	1.07E-08	0.680
10	45247418	46769302	372	1.33E-04	1.49E-08	0.275
10	47768664	49812917	342	-4.34E-05	6.83E-09	0.599
10	49816189	50839403	568	4.58E-05	9.74E-09	0.643
10	50840736	52086621	206	1.50E-04	2.28E-08	0.320
10	52086939	52707026	235	-1.61E-05	3.42E-09	0.784
10	52708822	53362540	343	9.29E-06	6.51E-09	0.908
10	53362932	54413554	512	5.81E-06	9.49E-09	0.952
10	54413909	55334166	522	-1.29E-04	9.82E-09	0.193
10	55334740	56204677	453	2.62E-05	6.33E-09	0.742
10	56207098	56883966	278	6.93E-05	6.78E-09	0.400
10	56885639	58806290	703	-3.91E-05	9.32E-09	0.686
10	59158617	59822195	312	5.94E-05	4.39E-09	0.370
10	59822974	60375613	205	2.98E-05	2.49E-09	0.550
10	62558842	64263322	625	-8.42E-05	1.05E-08	0.411
10	60375857	62558737	969	-1.53E-04	1.62E-08	0.230
10	64263402	65469133	435	1.42E-05	6.32E-09	0.858
10	65735028	66980708	393	-3.01E-05	6.04E-09	0.699
10	66980980	67545544	227	1.80E-05	3.96E-09	0.775
10	67546331	68429563	514	-4.89E-05	9.26E-09	0.611
10	68430236	69893580	549	-1.94E-05	9.84E-09	0.845
10	69896336	71052508	439	2.47E-05	7.61E-09	0.777
10	71052888	72479853	927	1.92E-05	2.29E-08	0.899
10	72480072	73612902	783	4.97E-05	1.87E-08	0.716
10	73614288	75696752	545	1.29E-05	4.67E-09	0.850
10	75700056	78638957	888	9.97E-06	1.15E-08	0.926
10	78642246	79740262	593	-2.79E-05	1.06E-08	0.787
10	79742617	80874092	594	8.68E-05	1.32E-08	0.451
10	80874523	81881954	306	2.96E-04	1.91E-08	0.033
10	81882370	82378624	276	2.10E-05	5.20E-09	0.771
10	82379403	82881996	242	6.19E-05	5.02E-09	0.382
10	82883949	83597042	269	-2.01E-05	4.38E-09	0.762
10	83597818	84383108	342	-1.69E-05	5.29E-09	0.816
10	84383277	85424102	486	9.56E-05	7.18E-09	0.259
10	85424493	86459792	528	3.84E-05	1.02E-08	0.704
10	86460169	87285511	356	3.72E-06	5.12E-09	0.959

10	87285907	88450872	496	-2.09E-05	9.84E-09	0.833
10	88451869	89845801	371	1.09E-05	6.60E-09	0.893
10	89846944	90734906	410	-9.56E-05	7.38E-09	0.266
10	90735832	91959588	592	-2.10E-05	9.83E-09	0.832
10	91960142	92682235	319	-3.08E-06	4.58E-09	0.964
10	92682369	94196248	469	3.77E-05	7.09E-09	0.655
10	94196690	95064828	355	2.86E-05	4.64E-09	0.674
10	95065655	95946938	409	-3.26E-05	7.78E-09	0.711
10	95949432	97984754	852	-9.43E-05	1.11E-08	0.370
10	97985690	99554180	665	-5.95E-05	1.09E-08	0.568
10	99554895	100240681	333	-2.10E-05	5.82E-09	0.783
10	100241204	102089663	714	3.59E-06	9.79E-09	0.971
10	102090924	104207799	687	-3.82E-05	9.49E-09	0.695
10	104208761	106139081	683	-9.82E-05	9.98E-09	0.325
10	106139294	107874146	733	-9.91E-05	1.08E-08	0.340
10	107875182	109577686	742	1.01E-04	1.33E-08	0.382
10	109578567	110311094	318	-2.18E-05	4.81E-09	0.753
10	110311425	112559414	764	-4.14E-05	1.13E-08	0.697
10	112560012	113839866	517	-7.69E-05	7.42E-09	0.372
10	113845990	115698315	909	-1.79E-04	1.93E-08	0.197
10	117705146	118416795	358	-6.52E-05	5.90E-09	0.396
10	115700005	117702645	679	-9.40E-05	1.02E-08	0.351
10	118417453	119697663	620	-6.76E-05	1.31E-08	0.555
10	119698700	120994671	529	-9.73E-05	1.03E-08	0.339
10	120997666	121874991	393	5.65E-05	7.32E-09	0.509
10	121875531	122805770	507	-1.02E-05	1.01E-08	0.920
10	122807254	123853223	520	1.78E-04	1.27E-08	0.114
10	123855124	124894743	543	-2.96E-05	1.01E-08	0.768
10	124894909	125868860	561	5.70E-05	1.28E-08	0.614
10	125919795	126739513	451	9.09E-05	1.17E-08	0.400
10	126739798	127820978	515	6.98E-05	9.06E-09	0.463
10	127821105	128409952	404	-1.11E-05	9.07E-09	0.907
10	128410393	129482709	632	-1.54E-04	1.40E-08	0.191
10	129483173	130282731	491	5.54E-05	1.11E-08	0.599
10	130282817	131192968	536	-2.88E-05	1.35E-08	0.804
10	131195759	132251820	595	-3.40E-05	1.21E-08	0.757
10	132252499	132964299	471	3.77E-05	1.06E-08	0.714

10	132966372	133355925	285	-3.41E-06	7.98E-09	0.970
10	133356417	134858046	637	-1.02E-04	1.30E-08	0.369
10	134858720	135524335	224	8.07E-06	3.74E-09	0.895
11	333347	1057948	319	-2.53E-05	5.45E-09	0.731
11	1058583	1861912	307	6.25E-05	1.18E-08	0.565
11	1862168	2422034	203	-1.93E-04	1.44E-08	0.107
11	2423545	3660412	521	-1.08E-04	1.32E-08	0.348
11	3661873	4745931	518	-6.35E-05	1.01E-08	0.528
11	4746391	5529152	652	-1.45E-05	1.07E-08	0.888
11	5532222	6010718	357	7.81E-05	6.60E-09	0.336
11	6011761	6917273	554	3.82E-06	1.02E-08	0.970
11	6917772	7715871	483	-5.45E-05	1.19E-08	0.617
11	7717884	9068716	661	7.17E-05	1.21E-08	0.515
11	9069046	10370675	439	-9.23E-05	6.95E-09	0.268
11	10371614	11361034	681	-4.56E-05	1.46E-08	0.706
11	11361458	12018942	492	7.00E-06	1.13E-08	0.947
11	12019010	13133902	717	-6.12E-05	1.66E-08	0.634
11	13134600	14934686	720	3.73E-05	1.06E-08	0.718
11	14936943	16789155	641	-5.29E-06	9.12E-09	0.956
11	16790799	18788126	916	2.14E-04	1.58E-08	0.088
11	18789820	20113591	791	1.36E-04	1.76E-08	0.304
11	20115277	21773251	1016	1.86E-05	2.27E-08	0.902
11	21773680	23111273	502	-9.63E-05	8.30E-09	0.290
11	24750113	25106557	162	-3.51E-06	2.71E-09	0.946
11	23112584	24749839	708	1.87E-05	1.41E-08	0.875
11	25109191	26046095	403	-3.40E-05	6.43E-09	0.671
11	26046627	27018778	418	-5.71E-05	6.67E-09	0.484
11	27019873	28741185	545	6.08E-05	7.96E-09	0.496
11	28742220	30056918	404	2.59E-05	6.98E-09	0.756
11	30057440	31853968	575	-1.06E-05	6.97E-09	0.899
11	31856654	33383448	566	-6.94E-05	9.25E-09	0.471
11	33384046	34321466	430	-3.57E-05	8.90E-09	0.705
11	34321876	35034307	445	-2.28E-04	2.20E-08	0.125
11	35036029	36117340	598	6.69E-05	1.25E-08	0.550
11	36118027	37838625	746	4.67E-05	1.44E-08	0.698
11	37839347	40357803	640	-9.99E-06	9.90E-09	0.920
11	40359663	40716222	154	-2.95E-05	2.51E-09	0.555

11	40716823	42243196	591	-1.22E-05	9.90E-09	0.902
11	42244050	42854352	256	2.26E-05	3.91E-09	0.717
11	42854878	43952796	470	-1.15E-05	8.13E-09	0.898
11	43953774	46245900	1080	3.20E-05	2.48E-08	0.839
11	46252956	47893120	417	-3.33E-05	8.10E-09	0.711
11	48351331	49659131	215	-2.07E-05	2.42E-09	0.674
11	49660004	55116367	287	-4.20E-05	3.00E-09	0.444
11	55116451	56160712	419	-3.10E-05	4.66E-09	0.650
11	56625775	58455947	713	-4.53E-05	8.96E-09	0.633
11	58457495	59615953	298	1.87E-05	3.50E-09	0.752
11	59620206	61870732	816	2.03E-05	1.45E-08	0.866
11	61873755	63153602	403	-1.04E-04	6.09E-09	0.181
11	63154309	66835194	1070	6.18E-05	1.51E-08	0.615
11	66835372	68515239	413	-2.61E-05	1.21E-08	0.813
11	68521072	69508567	391	-1.71E-04	6.81E-08	0.511
11	69509343	70613763	469	1.69E-04	1.34E-08	0.144
11	70616465	71842303	362	2.10E-05	5.58E-09	0.779
11	71842976	74158869	681	-5.50E-05	1.13E-08	0.605
11	74159542	76431100	890	1.23E-04	1.86E-08	0.368
11	76432156	77903706	543	1.46E-06	7.07E-09	0.986
11	79723675	80720242	465	1.42E-04	1.05E-08	0.164
11	77904339	79723318	879	-3.56E-05	1.51E-08	0.772
11	80721384	81653514	543	-1.18E-05	1.01E-08	0.907
11	81654617	82844918	481	-1.43E-05	8.30E-09	0.875
11	82847086	83575703	267	-1.24E-05	3.60E-09	0.836
11	83576460	84208616	304	3.97E-05	5.49E-09	0.592
11	84212522	84618654	169	1.73E-05	3.54E-09	0.771
11	84618788	86623913	828	4.48E-07	1.38E-08	0.997
11	86625137	87429545	370	-2.20E-05	5.74E-09	0.771
11	87431647	87827395	167	1.72E-05	3.57E-09	0.773
11	87827514	89208590	526	7.76E-05	9.58E-09	0.428
11	89441680	91115475	480	9.63E-07	6.47E-09	0.990
11	91118661	91676282	198	1.71E-05	3.56E-09	0.775
11	91677374	92626428	367	9.02E-06	4.97E-09	0.898
11	92627643	93270431	278	-9.71E-06	4.82E-09	0.889
11	93272186	94389331	421	3.46E-05	6.58E-09	0.670
11	94391845	95329108	415	7.58E-05	9.17E-09	0.429

11	95332310	96848965	669	7.00E-05	1.25E-08	0.531
11	96849530	98703542	778	3.07E-05	1.52E-08	0.803
11	98703733	99613901	507	4.11E-05	1.03E-08	0.686
11	101158517	101892922	344	1.59E-05	4.66E-09	0.816
11	99614560	101156501	812	-1.59E-04	1.58E-08	0.207
11	101894471	102668702	378	-7.16E-05	9.44E-09	0.461
11	102668882	103491310	383	-1.01E-05	6.47E-09	0.900
11	103491586	104871322	551	4.83E-05	8.36E-09	0.597
11	104872103	106251514	419	6.42E-05	5.65E-09	0.393
11	106252171	107489662	535	-6.38E-05	1.08E-08	0.539
11	107491606	108495975	326	-6.83E-05	4.75E-09	0.322
11	108496357	110701645	740	7.68E-05	1.11E-08	0.466
11	110702636	111981853	468	1.10E-04	6.86E-09	0.182
11	111985737	113103996	469	1.15E-05	7.58E-09	0.895
11	113105405	113958177	414	-1.30E-04	1.79E-08	0.332
11	113959061	114742322	447	-2.42E-05	8.89E-09	0.798
11	114743956	115627217	415	6.46E-05	8.16E-09	0.474
11	115652723	117097952	688	-1.73E-05	1.48E-08	0.887
11	118899218	120171371	573	1.80E-05	1.17E-08	0.868
11	117098436	118898319	897	1.75E-04	1.69E-08	0.178
11	120173354	121660636	712	-1.24E-04	1.25E-08	0.269
11	121661507	123289851	720	-6.49E-05	1.65E-08	0.613
11	123291301	124403522	616	4.29E-05	1.16E-08	0.690
11	124404297	125984562	770	1.46E-04	1.60E-08	0.249
11	125984970	126772347	528	-1.94E-05	1.12E-08	0.854
11	126772905	127715860	459	5.34E-05	8.95E-09	0.572
11	127716140	129186210	717	4.53E-05	1.44E-08	0.706
11	129187995	129838375	322	-1.06E-04	7.62E-09	0.225
11	129839528	130892029	436	-1.62E-04	9.21E-09	0.091
11	130894131	131870763	676	1.79E-05	1.54E-08	0.886
11	131871467	132386355	340	-5.78E-05	5.77E-09	0.446
11	132386834	133679495	782	-1.01E-04	1.94E-08	0.470
11	133679758	134946382	714	-1.84E-04	1.70E-08	0.158
12	68657	665200	216	-2.80E-05	4.71E-09	0.683
12	665655	1602602	326	6.09E-05	6.10E-09	0.436
12	1602652	2176046	281	2.81E-05	7.10E-09	0.739
12	2176265	2886299	324	5.92E-06	8.36E-09	0.948

12	2886946	3558488	354	9.32E-05	8.48E-09	0.312
12	3559429	4693778	654	-1.28E-04	1.70E-08	0.326
12	4695425	5877653	808	6.05E-05	1.71E-08	0.644
12	5878686	6879279	440	8.81E-05	1.17E-08	0.416
12	8658644	10477423	257	-7.30E-05	5.12E-09	0.308
12	10477712	11684725	445	-2.30E-05	6.61E-09	0.777
12	11687997	12530718	423	-2.58E-04	1.01E-08	0.010
12	12533494	13362446	379	-2.33E-05	8.38E-09	0.799
12	13362818	14658341	700	-3.44E-06	1.78E-08	0.979
12	14659441	16423287	588	-8.42E-05	9.52E-09	0.388
12	16423998	18466533	744	-6.40E-05	1.35E-08	0.582
12	18467618	19924288	581	-3.77E-05	1.23E-08	0.734
12	19925541	21568051	876	5.06E-06	1.42E-08	0.966
12	21569730	22325878	305	-3.20E-05	4.82E-09	0.645
12	22327634	22943718	204	4.21E-05	3.82E-09	0.496
12	22944771	23819079	359	-1.00E-04	1.02E-08	0.322
12	23819364	24545631	412	1.31E-04	1.03E-08	0.197
12	24545834	25991825	716	1.86E-05	1.32E-08	0.872
12	25992543	27529660	775	-4.31E-05	1.56E-08	0.730
12	27529712	29111136	699	9.17E-05	1.10E-08	0.381
12	29111480	30354932	708	-1.05E-04	1.13E-08	0.323
12	30355547	31067009	415	5.08E-05	6.14E-09	0.517
12	31067490	32081072	396	-4.82E-05	8.48E-09	0.600
12	32082695	32645755	311	-2.84E-05	6.49E-09	0.725
12	32645848	33435901	367	-1.23E-05	5.84E-09	0.873
12	33436298	38231063	325	-1.02E-05	4.00E-09	0.872
12	38233264	40017827	410	-1.11E-05	4.85E-09	0.873
12	40022029	41015211	475	-4.80E-06	7.45E-09	0.956
12	41015333	42346846	665	5.02E-05	1.03E-08	0.620
12	42347363	43074061	260	5.96E-05	3.85E-09	0.336
12	43074434	43982146	422	7.09E-06	6.25E-09	0.928
12	43982342	44922863	221	-6.09E-05	3.36E-09	0.293
12	44923334	46022099	342	-1.28E-05	5.17E-09	0.859
12	46023426	47504173	481	7.91E-05	7.15E-09	0.350
12	47505424	48267360	361	1.03E-05	8.31E-09	0.910
12	48267635	48965461	366	-4.60E-05	1.03E-08	0.650
12	48967935	50160195	313	1.82E-04	4.92E-09	0.009

12	50160662	51582048	414	-3.12E-05	7.26E-09	0.714
12	51582188	52913730	699	2.17E-05	1.11E-08	0.837
12	52914211	54379670	715	9.29E-06	2.99E-08	0.957
12	54380016	56792337	760	-1.61E-04	1.27E-08	0.155
12	56795415	57534912	235	1.08E-04	3.64E-09	0.074
12	57535266	58748272	384	1.49E-05	5.01E-09	0.833
12	58748846	60859665	698	6.56E-06	9.66E-09	0.947
12	60859809	61738896	266	2.37E-06	3.22E-09	0.967
12	61740561	63158569	602	1.09E-05	1.11E-08	0.918
12	63158712	63908647	359	-9.07E-05	6.76E-09	0.270
12	63910665	65383507	407	-1.69E-04	1.06E-08	0.100
12	65926411	67039427	475	-5.70E-05	7.32E-09	0.505
12	67039600	67817612	376	3.97E-05	7.48E-09	0.646
12	67818111	68740414	516	-4.53E-05	1.03E-08	0.655
12	68742112	69826093	484	-8.11E-05	1.12E-08	0.443
12	69826237	70815755	524	9.34E-05	8.57E-09	0.313
12	70817279	71688642	411	-5.33E-05	8.49E-09	0.563
12	71690933	72513196	315	5.00E-05	4.92E-09	0.476
12	72525849	74846997	634	-1.73E-05	9.57E-09	0.859
12	75193041	76058346	327	1.92E-05	5.14E-09	0.789
12	76060283	77098899	452	-9.42E-06	8.09E-09	0.917
12	77099081	78398357	623	3.27E-05	1.12E-08	0.757
12	78400884	78925445	243	2.20E-05	5.41E-09	0.765
12	78926240	80944201	508	3.31E-05	7.47E-09	0.702
12	80945774	83137872	721	-7.74E-05	1.28E-08	0.494
12	83143660	83854442	269	-1.35E-05	5.54E-09	0.856
12	83856540	85014953	417	5.95E-07	5.14E-09	0.993
12	85016898	87296535	569	-3.34E-05	7.22E-09	0.694
12	87297692	87905878	166	1.05E-05	2.50E-09	0.834
12	87909726	88848006	218	4.74E-05	3.21E-09	0.402
12	88851543	91827908	896	6.88E-05	1.51E-08	0.576
12	91827930	93792073	767	-9.58E-05	1.54E-08	0.441
12	93792655	95717265	1014	-7.75E-05	2.09E-08	0.592
12	99224060	99816442	203	-2.72E-05	3.63E-09	0.651
12	95717497	97439396	837	-1.41E-04	1.61E-08	0.266
12	97439589	99220284	910	-2.33E-06	1.82E-08	0.986
12	101857796	102961329	417	-1.83E-04	6.78E-09	0.027

12	99816842	101857521	802	1.30E-04	1.51E-08	0.291
12	102961450	104011319	432	-2.39E-06	7.84E-09	0.978
12	105817813	106155478	204	2.73E-05	4.63E-09	0.689
12	104012667	105816376	1163	6.56E-05	1.91E-08	0.635
12	106156001	107521770	612	4.04E-05	9.06E-09	0.671
12	107527013	108504440	424	8.91E-05	7.34E-09	0.298
12	108506696	110111462	699	2.85E-06	1.11E-08	0.978
12	110112932	113027651	567	-9.32E-05	6.67E-09	0.254
12	113030227	113877766	334	1.62E-04	5.40E-09	0.027
12	113885696	114882476	594	1.25E-04	1.57E-08	0.319
12	114883085	115326972	185	-5.35E-05	6.00E-09	0.490
12	117087697	117644288	184	4.59E-05	3.32E-09	0.426
12	115329869	117087376	969	7.95E-05	2.13E-08	0.586
12	117644436	118888131	674	3.91E-05	1.37E-08	0.738
12	118888731	119301273	203	-4.97E-06	4.57E-09	0.941
12	119303044	120007172	394	-1.64E-04	1.33E-08	0.155
12	120008093	121720040	643	3.56E-05	9.77E-09	0.719
12	121721831	124710880	878	-3.05E-05	1.12E-08	0.774
12	124710996	125416169	363	-2.32E-05	1.67E-08	0.858
12	125417493	126461057	519	-5.35E-05	1.18E-08	0.622
12	126463562	127382559	515	-1.09E-04	9.20E-09	0.256
12	127383648	128141516	401	-7.44E-05	1.04E-08	0.466
12	128141610	128810520	445	5.05E-05	1.06E-08	0.623
12	128814615	129688509	569	5.65E-05	1.71E-08	0.666
12	129689639	130157939	346	-9.02E-05	9.21E-09	0.347
12	130158997	130859973	457	7.60E-05	1.16E-08	0.481
12	130861322	131407836	344	5.10E-05	9.16E-09	0.594
12	131408716	132012266	369	1.21E-05	7.66E-09	0.890
12	132014206	132706778	276	-4.91E-05	5.39E-09	0.504
12	132807034	133841558	234	-2.37E-04	3.42E-08	0.200
13	19020095	20682304	388	-1.33E-05	4.65E-09	0.846
13	20684769	21688499	462	1.07E-04	7.60E-09	0.220
13	21688974	22209012	217	-5.61E-05	3.87E-09	0.367
13	23590702	23827019	193	-4.48E-05	5.82E-09	0.557
13	22211354	23590611	811	9.73E-05	2.08E-08	0.501
13	23827815	24477568	384	4.06E-05	6.88E-09	0.625
13	24477971	25210959	501	-1.64E-05	1.04E-08	0.872

13	25212359	25639070	222	-9.76E-05	3.98E-09	0.122
13	25640695	27282973	736	1.30E-05	1.19E-08	0.905
13	27283115	28283059	561	5.98E-05	1.15E-08	0.577
13	28284459	28876214	284	-7.26E-05	4.61E-09	0.285
13	28879332	29872965	448	-1.82E-04	1.09E-08	0.081
13	29873521	31379027	783	1.53E-04	1.60E-08	0.226
13	31384910	32983250	791	3.22E-05	1.35E-08	0.782
13	32984197	34681078	708	-7.07E-05	9.45E-09	0.467
13	34683262	36042839	439	-1.16E-05	8.30E-09	0.898
13	36043978	37027089	548	-1.14E-05	8.95E-09	0.904
13	37029009	38290705	619	-6.65E-05	8.84E-09	0.479
13	38291011	38877678	287	2.68E-06	4.09E-09	0.967
13	38887155	40179288	575	-3.66E-06	8.04E-09	0.967
13	40181792	41773356	639	7.21E-05	1.03E-08	0.478
13	41774489	43099692	546	-4.22E-05	7.99E-09	0.637
13	43099889	44637493	786	6.11E-05	1.19E-08	0.576
13	46493234	46866562	161	5.69E-06	2.17E-09	0.903
13	44638337	46491101	753	-4.92E-05	1.22E-08	0.656
13	48440893	49987145	462	-5.34E-07	4.99E-09	0.994
13	46869621	48439005	725	4.48E-05	1.12E-08	0.672
13	49987663	51254196	497	6.29E-05	1.58E-08	0.617
13	51254712	53336663	702	-4.16E-05	9.54E-09	0.670
13	53337952	54681048	610	6.09E-05	1.30E-08	0.593
13	54681363	56433642	544	1.90E-05	6.67E-09	0.816
13	56434161	58247964	411	-2.04E-05	4.24E-09	0.755
13	60087834	60854106	241	-1.05E-04	2.69E-09	0.043
13	58248124	60085069	714	5.10E-05	1.10E-08	0.628
13	60855021	61589772	347	2.89E-05	4.83E-09	0.678
13	61591949	62912481	486	-1.23E-05	6.69E-09	0.881
13	62913542	64201284	414	-2.55E-05	6.06E-09	0.744
13	64202212	65339575	366	7.28E-06	4.32E-09	0.912
13	65340421	66197786	242	4.02E-05	2.43E-09	0.415
13	66200191	67713633	592	-1.81E-05	8.05E-09	0.840
13	67714255	69561090	643	1.03E-05	9.34E-09	0.915
13	70643712	71095217	239	-3.25E-06	4.02E-09	0.959
13	69561894	70642326	429	-2.53E-06	7.16E-09	0.976
13	71095483	71759743	313	-4.78E-05	6.49E-09	0.553

13	71763633	72958993	471	3.27E-06	7.57E-09	0.970
13	72959394	73795534	346	2.57E-04	1.68E-08	0.047
13	73796728	75084163	678	-5.89E-05	1.35E-08	0.612
13	75084473	76504672	666	2.98E-04	1.17E-08	0.006
13	78032193	78807439	318	-3.47E-05	4.29E-09	0.596
13	76505282	78030962	671	5.07E-05	1.05E-08	0.620
13	78807836	80252742	553	6.13E-05	7.10E-09	0.467
13	80257200	82580321	856	1.55E-05	1.27E-08	0.890
13	82580994	84132765	493	1.67E-05	5.16E-09	0.816
13	84133082	85035423	269	7.59E-06	3.84E-09	0.903
13	85037313	85609664	262	4.09E-06	3.59E-09	0.946
13	85609760	87039195	528	2.99E-05	9.23E-09	0.756
13	87039360	88190520	314	6.21E-05	3.45E-09	0.290
13	88191656	89057861	239	-2.55E-05	3.25E-09	0.655
13	89059635	90177618	305	3.16E-05	3.17E-09	0.575
13	90179364	90867683	318	-2.88E-05	5.10E-09	0.687
13	90869873	91967960	404	-5.08E-05	6.15E-09	0.517
13	91970313	92912119	364	-2.58E-05	5.28E-09	0.722
13	92912423	93918144	412	-5.04E-05	6.15E-09	0.521
13	93918533	94562549	322	1.06E-04	4.23E-09	0.105
13	94563601	95540024	474	4.81E-05	9.19E-09	0.616
13	97521207	98589842	365	-1.42E-05	5.53E-09	0.849
13	95540709	97520182	918	2.41E-05	1.33E-08	0.835
13	98594975	100043997	776	-1.99E-06	1.20E-08	0.986
13	100044673	101392748	562	4.67E-05	9.35E-09	0.629
13	101394726	102299385	583	3.02E-05	9.12E-09	0.752
13	102299498	103339911	558	1.40E-04	8.82E-09	0.137
13	103340536	104065375	505	2.76E-05	8.81E-09	0.769
13	104065534	104842764	367	-7.64E-06	7.07E-09	0.928
13	104843186	105990071	584	4.48E-05	1.30E-08	0.694
13	105990812	106439396	245	-5.67E-05	4.71E-09	0.409
13	106442608	107785431	712	-1.03E-04	1.48E-08	0.394
13	107788025	108518444	439	3.12E-05	9.39E-09	0.747
13	108519987	109359792	437	9.84E-05	9.24E-09	0.306
13	109359834	110338482	539	9.08E-05	1.15E-08	0.397
13	110338913	111432821	728	1.38E-04	1.68E-08	0.287
13	111434814	113266084	864	3.54E-05	1.62E-08	0.781

13	113269127	115109853	666	1.20E-05	1.02E-08	0.906
14	20544512	20877114	188	1.01E-05	4.23E-09	0.876
14	20878890	21267077	308	4.75E-05	9.99E-09	0.635
14	21271573	22378835	520	-1.51E-05	1.26E-08	0.893
14	22379132	22738105	228	1.12E-04	5.36E-09	0.126
14	22738161	23607730	506	2.22E-04	1.24E-08	0.047
14	23607866	24904154	514	1.29E-04	1.62E-08	0.309
14	24904240	26134815	652	2.77E-05	1.11E-08	0.792
14	26135167	26679549	244	-1.62E-06	5.23E-09	0.982
14	26680794	28466225	588	-1.28E-05	1.06E-08	0.901
14	28466533	29196856	255	1.03E-05	4.53E-09	0.878
14	29198059	32014712	795	-3.66E-05	1.61E-08	0.773
14	32016504	33033695	420	5.51E-05	8.95E-09	0.561
14	36345767	36682357	143	1.74E-05	2.25E-09	0.714
14	34769883	36344816	496	8.36E-06	8.45E-09	0.928
14	33036081	34769821	1119	-1.83E-04	2.97E-08	0.287
14	36683516	38481516	689	2.53E-04	1.93E-08	0.069
14	38486187	39370633	360	-2.81E-05	6.21E-09	0.722
14	39370726	40336296	361	-5.13E-05	5.88E-09	0.504
14	40336990	41615299	437	-1.61E-05	6.38E-09	0.840
14	41615331	43065019	493	1.84E-05	7.29E-09	0.830
14	43068948	44541699	471	-6.68E-05	9.83E-09	0.501
14	44542368	45247021	304	2.07E-06	5.52E-09	0.978
14	45250418	46311209	220	1.49E-05	2.51E-09	0.765
14	46312678	47456738	355	-6.19E-06	4.82E-09	0.929
14	47457846	48351664	389	2.69E-05	6.69E-09	0.742
14	48352461	49001226	256	3.29E-06	5.26E-09	0.964
14	49004194	49955015	359	5.34E-05	5.82E-09	0.484
14	49955381	52077965	933	3.31E-05	1.79E-08	0.804
14	52078316	52761693	415	1.23E-06	8.49E-09	0.989
14	52762622	54162101	504	7.09E-05	1.27E-08	0.529
14	54162655	55043520	312	-4.64E-06	6.36E-09	0.954
14	55044905	56215082	474	-1.06E-04	7.65E-09	0.227
14	56215477	56949485	467	-7.76E-05	7.95E-09	0.384
14	58449526	59135770	213	9.78E-05	3.47E-09	0.097
14	56949644	58448681	677	-9.30E-05	1.36E-08	0.425
14	59135989	61649612	943	-4.61E-06	1.36E-08	0.968

14	63166807	63890839	304	-5.63E-05	4.98E-09	0.425
14	61651278	63164649	666	4.71E-05	1.37E-08	0.688
14	63897095	65218457	418	-2.32E-05	7.96E-09	0.795
14	65220775	66353557	440	4.14E-05	6.99E-09	0.621
14	66354477	67990648	525	3.60E-05	7.30E-09	0.674
14	67991028	69355606	630	-5.04E-05	1.92E-08	0.716
14	71132759	72213258	357	3.01E-05	4.79E-09	0.664
14	69356353	71131097	786	2.51E-05	1.78E-08	0.851
14	72214790	72888979	421	-4.30E-05	7.26E-09	0.614
14	72889376	74250730	549	-9.67E-05	1.32E-08	0.399
14	74252993	75747118	538	1.41E-04	7.73E-09	0.108
14	75747258	76583135	370	-9.45E-06	7.27E-09	0.912
14	76583775	77332160	385	-3.06E-05	9.01E-09	0.748
14	77332408	78421884	573	8.57E-06	1.04E-08	0.933
14	78422260	79026816	371	3.63E-05	7.01E-09	0.665
14	80999361	81787255	327	3.33E-05	5.37E-09	0.649
14	81787481	82459297	259	-1.80E-05	5.19E-09	0.803
14	79027504	80998547	757	7.34E-05	1.37E-08	0.531
14	82461794	84087895	606	-9.52E-05	1.22E-08	0.389
14	84087979	85035235	352	2.80E-05	5.94E-09	0.716
14	85037974	86051751	438	-4.33E-05	9.28E-09	0.653
14	86053777	87334624	501	-2.13E-05	8.81E-09	0.821
14	87335256	88641174	451	-5.82E-05	7.37E-09	0.498
14	88641335	90069965	638	-3.02E-05	1.17E-08	0.780
14	90075441	92098486	749	-4.25E-06	1.60E-08	0.973
14	92099719	93378838	646	3.48E-05	1.34E-08	0.764
14	93379151	94420996	456	2.33E-05	1.32E-08	0.839
14	94421488	95347359	703	8.06E-05	1.44E-08	0.502
14	96251418	97570260	662	3.05E-07	1.44E-08	0.998
14	95347761	96250900	641	-9.27E-05	1.46E-08	0.443
14	97571437	98987472	651	-4.64E-05	1.29E-08	0.683
14	98989483	99980087	542	-2.37E-05	1.36E-08	0.839
14	99981105	101244250	481	-1.07E-05	9.02E-09	0.910
14	101244293	102339561	445	1.49E-04	1.30E-08	0.192
14	104761778	105440784	241	-6.97E-05	5.39E-09	0.342
14	105443695	106552839	139	-4.18E-05	2.66E-09	0.418
14	102341650	104759919	713	4.83E-04	1.39E-08	0.000

15	22362658	23288776	142	-3.52E-05	4.09E-09	0.582
15	23292175	24320772	294	1.19E-05	7.47E-09	0.890
15	24321414	24976269	145	-1.21E-05	3.13E-09	0.828
15	25107905	25750941	208	-1.97E-05	4.35E-09	0.765
15	25752114	26185554	309	-9.21E-05	8.37E-09	0.314
15	26186113	26675188	259	1.98E-04	6.95E-09	0.018
15	26676258	27265824	242	-2.89E-05	6.26E-09	0.715
15	27266835	27819408	253	-8.78E-05	5.74E-09	0.247
15	27820352	28325439	264	5.25E-05	5.44E-09	0.476
15	28326536	29617158	224	1.16E-04	3.95E-09	0.064
15	29617517	31314317	409	3.63E-05	7.56E-09	0.676
15	31315570	32177326	353	2.81E-04	1.57E-08	0.025
15	32180607	33251727	347	1.10E-04	8.34E-09	0.227
15	33253484	33792116	378	-3.72E-05	1.25E-08	0.739
15	33793179	34273308	356	-5.01E-05	8.82E-09	0.594
15	34273346	34970673	223	1.67E-05	4.26E-09	0.798
15	34971983	35941864	367	2.96E-05	6.32E-09	0.710
15	35943377	36424581	299	-1.70E-04	5.92E-09	0.028
15	36425448	36968033	285	-7.13E-05	7.10E-09	0.397
15	36968291	37930544	441	4.09E-05	8.96E-09	0.666
15	37931874	38804104	335	-2.07E-04	6.52E-09	0.010
15	38805185	39518563	383	3.03E-05	7.86E-09	0.732
15	39519092	40382441	407	-8.54E-06	7.90E-09	0.923
15	40383779	41211827	274	-2.16E-04	6.89E-09	0.009
15	41212265	42222063	298	3.43E-05	3.94E-09	0.585
15	42222390	43473907	377	-6.87E-05	5.05E-09	0.333
15	43474655	44988610	261	2.94E-05	2.79E-09	0.578
15	44992475	46292382	423	4.82E-05	6.21E-09	0.541
15	46294385	47000090	290	-5.39E-06	4.51E-09	0.936
15	47000857	47977097	382	-2.47E-05	5.88E-09	0.747
15	47979127	49075918	359	-9.41E-06	5.25E-09	0.897
15	49078966	50004817	260	-1.52E-05	3.36E-09	0.794
15	50006184	51681073	716	-3.70E-05	1.03E-08	0.716
15	51682775	52589995	272	4.88E-05	3.05E-09	0.378
15	52590615	53014125	168	2.37E-06	2.33E-09	0.961
15	53015101	53766542	361	-8.54E-05	8.82E-09	0.364
15	53767876	54336662	313	-5.94E-05	5.74E-09	0.433

15	54337243	55134743	391	1.13E-05	7.27E-09	0.895
15	55135408	55469163	140	4.92E-05	2.28E-09	0.303
15	55470187	55877891	151	-7.82E-08	2.84E-09	0.999
15	55879453	56784345	319	-3.63E-05	5.19E-09	0.615
15	56784506	57637678	234	6.63E-06	2.92E-09	0.902
15	57638075	58438535	510	-4.22E-05	9.63E-09	0.667
15	58439935	59505191	501	5.63E-05	1.01E-08	0.574
15	59507391	60018662	244	8.84E-06	4.43E-09	0.894
15	60019136	60670149	284	1.08E-04	5.60E-09	0.151
15	61803892	62735671	460	8.11E-05	8.05E-09	0.366
15	60670287	61803306	669	2.59E-05	1.62E-08	0.839
15	62737452	64335240	693	-3.18E-05	1.23E-08	0.775
15	65104668	66358474	354	2.61E-05	4.54E-09	0.698
15	66359486	66948204	296	-1.08E-04	9.67E-09	0.271
15	66948951	68640917	759	4.16E-05	1.17E-08	0.701
15	68642047	69936117	406	-1.29E-04	8.18E-09	0.154
15	69938058	70772135	451	1.47E-04	1.10E-08	0.161
15	70772414	71919256	463	-3.19E-05	8.25E-09	0.725
15	71920065	74447996	707	7.14E-05	9.92E-09	0.473
15	74453873	75516403	321	-1.01E-04	5.07E-09	0.158
15	75520945	76397358	160	5.54E-07	2.33E-09	0.991
15	76397390	77910873	426	-1.62E-05	5.30E-09	0.824
15	77912408	78637877	276	-1.01E-04	6.15E-09	0.199
15	78640760	79259009	192	-4.62E-05	3.34E-09	0.424
15	79261077	79991408	436	6.46E-05	7.92E-09	0.468
15	79992629	80635045	302	5.59E-05	6.71E-09	0.495
15	80637749	81604871	477	5.43E-05	1.04E-08	0.594
15	84063245	85517022	506	-4.71E-05	9.51E-09	0.629
15	81607249	84062334	626	6.60E-05	1.07E-08	0.523
15	85518062	86341812	361	-1.25E-04	4.87E-09	0.074
15	86342119	86650301	159	3.05E-05	3.12E-09	0.585
15	86650331	87113409	248	-1.53E-05	4.74E-09	0.824
15	87114392	87693557	314	-2.52E-05	7.17E-09	0.766
15	87693885	88032107	160	7.83E-06	3.15E-09	0.889
15	88032736	88742960	322	-1.57E-05	6.16E-09	0.841
15	88743108	89561223	336	7.56E-05	7.83E-09	0.393
15	89562631	90452882	361	-8.05E-05	5.97E-09	0.298

15	90453183	91221307	299	-3.72E-06	5.17E-09	0.959
15	91226586	91745246	252	-6.65E-05	6.13E-09	0.396
15	91749626	92164207	203	3.37E-05	5.14E-09	0.638
15	92165068	92667377	253	3.70E-05	6.00E-09	0.633
15	92669184	93590376	566	8.13E-05	1.22E-08	0.462
15	93591893	93908402	232	2.40E-05	8.56E-09	0.795
15	93909052	94547933	367	3.32E-06	8.47E-09	0.971
15	94548924	95576904	643	-3.16E-05	1.64E-08	0.805
15	95577096	96497729	429	-9.16E-05	1.08E-08	0.378
15	96500358	97091511	333	-1.01E-04	8.22E-09	0.265
15	97092654	98496698	628	3.09E-06	1.71E-08	0.981
15	98497881	99128454	384	-7.79E-05	1.01E-08	0.439
15	100078196	100456641	150	-1.97E-05	2.98E-09	0.718
15	99128932	100077720	485	-5.90E-05	1.11E-08	0.576
15	100457692	101403655	488	-1.63E-04	1.16E-08	0.130
15	101408616	101918220	304	1.05E-04	6.61E-09	0.195
15	101918830	102516259	285	5.06E-05	6.94E-09	0.543
16	60291	596605	208	-8.74E-05	4.57E-09	0.196
16	946205	1547999	260	-3.20E-05	5.44E-09	0.665
16	1551082	2293296	333	8.59E-05	4.79E-09	0.215
16	2294890	3960458	485	-3.00E-05	1.22E-08	0.786
16	3961770	4689471	274	5.42E-05	8.36E-09	0.553
16	4690187	5114499	203	-1.06E-04	3.58E-09	0.078
16	5115228	5854496	425	-7.21E-05	9.14E-09	0.451
16	5855169	6264138	317	6.81E-05	8.44E-09	0.459
16	6264139	7045506	647	-1.55E-05	1.39E-08	0.895
16	7046430	7524343	442	-4.20E-05	9.34E-09	0.664
16	8237157	8572350	255	5.71E-05	6.65E-09	0.484
16	8572557	8960864	188	1.28E-05	4.95E-09	0.856
16	7525825	8236887	554	3.10E-05	1.20E-08	0.777
16	8962907	9658861	334	-1.62E-04	7.47E-09	0.062
16	9659584	9997178	212	4.73E-05	3.65E-09	0.434
16	10002419	10430046	324	1.90E-05	6.14E-09	0.809
16	10431221	11493266	531	4.91E-05	1.07E-08	0.635
16	11612436	12151685	190	9.19E-06	4.02E-09	0.885
16	12775897	13119544	141	-2.45E-05	3.12E-09	0.661
16	12151797	12775407	451	-1.01E-05	7.32E-09	0.906

16	13120367	13978116	506	-2.24E-05	9.66E-09	0.820
16	13979558	15885158	355	-4.89E-05	6.04E-09	0.529
16	15885866	16220858	240	2.82E-05	3.87E-09	0.650
16	16222763	17334499	270	2.21E-05	5.90E-09	0.773
16	17335780	18062164	300	3.31E-05	6.21E-09	0.674
16	18065944	20054371	500	2.14E-06	8.79E-09	0.982
16	20055884	21235343	433	2.50E-05	7.49E-09	0.773
16	21237217	23369433	478	2.07E-05	9.13E-09	0.829
16	23371110	24308825	377	-5.98E-05	5.88E-09	0.435
16	24309108	25589623	527	-1.07E-04	9.40E-09	0.271
16	26683251	26943239	137	-4.47E-05	3.99E-09	0.479
16	25590147	26681997	620	-1.44E-04	1.44E-08	0.231
16	26944850	27445360	274	1.64E-04	7.83E-09	0.064
16	27446054	29023966	442	2.42E-05	5.37E-09	0.741
16	29694822	31380596	353	-3.93E-05	4.25E-09	0.547
16	31384554	47981754	255	8.54E-06	3.76E-09	0.889
16	47982405	49006844	259	5.63E-05	3.23E-09	0.322
16	49007408	50694011	658	-5.94E-05	1.38E-08	0.613
16	50698071	51700822	458	-4.96E-05	9.74E-09	0.615
16	51703888	53845487	830	3.08E-05	1.35E-08	0.790
16	53848448	54671719	496	-1.57E-04	1.38E-08	0.182
16	54673195	55505040	496	2.73E-05	1.07E-08	0.791
16	55506137	56906006	601	9.00E-06	9.75E-09	0.927
16	56908045	57663112	346	-3.62E-05	8.76E-09	0.699
16	57663619	58508278	391	-1.97E-04	1.49E-08	0.106
16	58510034	59888170	534	3.23E-05	8.07E-09	0.719
16	59889269	60744157	301	6.99E-06	5.93E-09	0.928
16	60745208	61991704	352	-4.56E-05	5.57E-09	0.542
16	61993462	63395786	493	-8.75E-05	9.31E-09	0.364
16	63396006	64714759	477	-3.71E-06	7.51E-09	0.966
16	64716851	65538017	376	-5.87E-05	5.43E-09	0.426
16	68429894	70885818	618	-1.55E-05	7.74E-09	0.860
16	65538484	68429077	906	-1.86E-05	1.30E-08	0.871
16	72915753	73542956	354	-4.78E-05	9.69E-09	0.627
16	70887506	72915425	539	-5.29E-05	6.76E-09	0.520
16	73543220	74724933	470	-1.43E-05	8.42E-09	0.876
16	74730819	75517115	291	-6.68E-05	5.38E-09	0.363

16	75518129	76056374	169	-2.84E-05	2.81E-09	0.592
16	76058697	76987262	437	2.91E-05	7.04E-09	0.728
16	76988130	77455781	322	-1.89E-05	6.50E-09	0.815
16	77456244	77688760	199	-1.87E-05	3.71E-09	0.759
16	77689074	78446696	583	-8.10E-05	1.31E-08	0.479
16	78452348	78709939	217	-6.80E-05	3.97E-09	0.280
16	78710328	79368760	699	-2.29E-05	1.69E-08	0.861
16	79369779	80184442	562	-1.70E-04	1.69E-08	0.191
16	80184495	81209234	656	7.19E-05	1.13E-08	0.498
16	82482458	83078581	572	7.90E-06	1.36E-08	0.946
16	81209631	82481987	784	7.03E-05	1.80E-08	0.601
16	83972415	84645588	626	-1.65E-04	1.83E-08	0.224
16	83078959	83971446	825	-1.25E-04	1.86E-08	0.358
16	84646612	85146709	319	2.03E-04	8.33E-09	0.026
16	85146935	85663943	210	3.90E-05	8.19E-09	0.666
16	85664453	86221644	410	1.01E-04	1.12E-08	0.340
16	86222417	86745679	507	6.43E-05	1.50E-08	0.599
16	86745955	87173290	253	2.51E-06	8.17E-09	0.978
16	87175053	88389287	532	-1.43E-05	1.27E-08	0.899
16	88439429	88964934	242	-6.53E-05	6.87E-09	0.431
16	88966667	89604610	217	-2.82E-05	4.14E-09	0.660
16	89605495	90292767	238	4.44E-06	4.93E-09	0.950
17	828	296442	132	-8.61E-05	1.17E-08	0.427
17	396660	820493	178	2.09E-04	1.66E-08	0.104
17	820511	1480393	258	-3.62E-05	1.32E-08	0.753
17	1481897	2613925	468	6.11E-05	1.47E-08	0.614
17	2616352	3425788	386	3.83E-05	1.42E-08	0.748
17	3426133	4321755	383	-1.84E-05	1.20E-08	0.866
17	4322053	4650487	175	3.63E-05	7.94E-09	0.684
17	4652156	5573766	370	3.40E-05	1.08E-08	0.743
17	6572170	6975193	180	5.57E-05	7.13E-09	0.509
17	5574433	6570522	609	-3.18E-06	2.22E-08	0.983
17	6976284	7723513	317	-2.12E-04	1.07E-08	0.040
17	7725660	8704551	398	3.91E-05	1.76E-08	0.768
17	8705612	9229793	253	-1.07E-04	1.99E-08	0.449
17	9233113	9714549	223	-6.21E-05	7.82E-09	0.483
17	9714995	10573151	522	1.96E-05	1.81E-08	0.884

17	10573457	11667346	523	-1.26E-04	1.80E-08	0.347
17	11668104	13000883	588	-5.72E-06	2.64E-08	0.972
17	13001700	13625425	351	-5.60E-05	1.36E-08	0.632
17	13626102	14209988	321	1.36E-05	1.16E-08	0.900
17	14210015	14499605	184	2.52E-05	6.99E-09	0.763
17	14500054	14859339	269	1.71E-05	8.95E-09	0.857
17	14860784	16319512	495	2.27E-04	1.60E-08	0.073
17	16321959	17292491	305	4.15E-05	9.82E-09	0.675
17	17299591	18446092	382	9.08E-05	1.00E-08	0.364
17	18456679	19666001	255	-5.26E-06	7.45E-09	0.951
17	20679485	21284673	159	1.82E-05	4.66E-09	0.790
17	21285166	25677370	210	2.31E-05	5.75E-09	0.761
17	25679294	26272308	203	-6.00E-05	5.64E-09	0.424
17	26273607	27884779	445	-9.95E-05	1.15E-08	0.354
17	27885675	29259899	374	3.38E-06	9.80E-09	0.973
17	29262773	30878805	457	-2.25E-06	1.80E-08	0.987
17	30879433	31989867	615	6.42E-05	1.97E-08	0.647
17	31990385	32677709	409	1.27E-04	1.25E-08	0.254
17	33616080	34393028	274	-5.74E-05	1.19E-08	0.599
17	32677947	33614452	507	-7.46E-05	1.64E-08	0.560
17	34393296	35725997	358	-4.20E-05	1.09E-08	0.687
17	35731855	37341235	493	4.85E-05	1.25E-07	0.891
17	37344586	38649203	329	-2.58E-04	1.75E-08	0.051
17	38652220	39657179	453	-2.88E-05	1.16E-08	0.790
17	39657337	40231221	206	-1.73E-06	5.49E-09	0.981
17	40232810	41769732	392	5.03E-05	1.00E-08	0.616
17	41771040	43317574	551	-9.44E-05	1.54E-08	0.447
17	44863413	45874715	302	2.57E-05	8.28E-09	0.778
17	45876022	46828302	408	-5.58E-05	1.21E-08	0.613
17	46828412	48027295	402	-1.11E-04	2.42E-08	0.478
17	48027688	48832008	360	-5.47E-05	1.40E-08	0.644
17	48832437	49892805	427	-2.55E-05	1.20E-08	0.816
17	49894599	51282380	527	3.75E-05	1.32E-08	0.744
17	52863321	53338298	227	4.49E-05	6.79E-09	0.586
17	51284441	52861905	537	-1.34E-04	1.56E-08	0.282
17	53346718	54164464	383	-2.79E-05	1.09E-08	0.789
17	54164935	55278525	429	-2.70E-05	1.37E-08	0.818

17	57487538	59307733	300	5.57E-07	7.26E-09	0.995
17	60885287	62035655	261	-5.71E-05	6.51E-09	0.479
17	59309085	60883279	464	-4.94E-05	1.35E-08	0.671
17	55279508	57486793	879	-1.73E-05	2.83E-08	0.918
17	64434524	65013985	328	-5.11E-05	1.09E-08	0.625
17	62037685	64434272	752	-9.10E-07	2.22E-08	0.995
17	65016683	66315468	381	-1.88E-04	1.28E-08	0.096
17	66318466	66903293	310	5.77E-05	9.95E-09	0.563
17	66906217	68199133	459	4.27E-05	1.34E-08	0.712
17	68199783	68875761	303	-3.60E-05	9.24E-09	0.708
17	68877746	69242135	206	1.87E-04	2.88E-08	0.271
17	69242932	70147289	397	-1.43E-04	3.35E-08	0.434
17	70148198	70947580	476	8.19E-05	1.83E-08	0.546
17	70948535	71825523	479	1.94E-04	1.73E-08	0.139
17	71829519	72740027	435	9.60E-06	1.55E-08	0.938
17	72742484	74016834	403	-4.22E-05	1.16E-08	0.695
17	74018640	74909405	367	4.49E-05	1.09E-08	0.667
17	74910943	75471247	313	-4.46E-05	1.26E-08	0.690
17	75476450	75842792	220	-3.12E-06	9.60E-09	0.975
17	75844316	76445231	219	8.00E-05	8.94E-09	0.397
17	76446689	77093768	321	1.19E-05	1.19E-08	0.913
17	77094717	77468875	305	-2.48E-05	1.37E-08	0.832
17	77471383	78780080	508	1.97E-05	1.75E-08	0.882
17	78780686	79683249	362	-7.76E-05	1.27E-08	0.492
17	79683698	80969550	415	2.56E-05	1.16E-08	0.812
18	349293	906343	394	-5.51E-06	9.19E-09	0.954
18	906673	1940581	433	-5.56E-06	8.88E-09	0.953
18	1940863	2206099	166	-3.00E-05	4.76E-09	0.664
18	2206891	2889826	314	-3.86E-05	6.74E-09	0.638
18	2891847	3357120	218	-1.48E-04	5.77E-09	0.052
18	3359237	3861997	217	2.22E-05	7.60E-09	0.799
18	3862461	4597021	431	1.17E-04	1.11E-08	0.266
18	4601573	5197395	229	-2.76E-06	5.42E-09	0.970
18	5198562	5852964	326	-2.65E-05	7.43E-09	0.759
18	5855598	6630394	359	-2.24E-05	8.28E-09	0.806
18	6630840	7373590	413	-1.25E-05	9.65E-09	0.899
18	7374066	8440357	509	-4.73E-06	9.47E-09	0.961

18	8440558	9414901	547	2.79E-04	1.28E-08	0.014
18	9415389	10462537	664	7.24E-05	1.75E-08	0.584
18	10465005	11028008	321	4.69E-05	5.64E-09	0.532
18	11028129	11529011	226	3.94E-05	5.18E-09	0.584
18	11530996	12737037	465	6.49E-05	7.86E-09	0.464
18	12737582	14358413	604	1.10E-04	1.04E-08	0.279
18	18527782	21622994	744	2.77E-05	1.42E-08	0.816
18	21623416	22745505	572	9.84E-05	1.15E-08	0.359
18	22746547	24088696	488	-5.30E-05	8.46E-09	0.565
18	24088831	24971742	490	-1.81E-05	7.26E-09	0.831
18	26874132	28037427	397	9.64E-06	5.76E-09	0.899
18	24972728	26873513	697	-2.73E-05	1.08E-08	0.793
18	28037840	28560707	235	-2.14E-05	4.31E-09	0.745
18	28561634	29563172	480	3.98E-05	8.03E-09	0.657
18	31777336	32456417	204	-1.03E-05	2.75E-09	0.845
18	29564408	31774120	674	7.20E-05	9.03E-09	0.448
18	32457437	33791804	485	-4.96E-05	7.38E-09	0.564
18	33792671	35072855	572	4.31E-05	8.55E-09	0.641
18	35073616	35837704	322	-1.73E-06	4.91E-09	0.980
18	35838871	36364691	241	4.41E-05	4.33E-09	0.502
18	36365021	37682827	411	-2.10E-05	5.92E-09	0.785
18	37683967	38766853	525	6.77E-05	7.96E-09	0.448
18	38767938	39891215	366	1.02E-05	5.41E-09	0.890
18	42606091	43295247	412	9.98E-05	7.19E-09	0.239
18	43295347	43881224	239	4.93E-05	3.20E-09	0.384
18	39892281	42606024	953	8.55E-05	1.41E-08	0.472
18	45314528	46208355	424	-2.71E-05	8.82E-09	0.773
18	43881706	45313390	680	5.40E-05	1.07E-08	0.602
18	46209339	47262925	483	5.49E-05	8.63E-09	0.555
18	47263681	47814821	330	-3.44E-05	4.95E-09	0.625
18	47816777	48784027	395	1.80E-05	5.99E-09	0.816
18	48784953	50151479	574	4.15E-05	1.06E-08	0.687
18	50152229	51059341	375	-9.27E-05	5.38E-09	0.206
18	51059850	51952586	343	-5.30E-05	5.64E-09	0.481
18	51953945	52707502	198	-5.83E-05	3.22E-09	0.304
18	52709949	53763665	408	-4.12E-05	7.24E-09	0.628
18	53764157	55089715	500	-9.48E-06	7.72E-09	0.914

18	55090919	55808073	392	1.81E-04	9.96E-09	0.069
18	55808692	56752598	490	3.36E-05	1.64E-08	0.793
18	56754050	57504565	478	1.43E-04	9.22E-09	0.136
18	57505073	58816387	484	-2.78E-05	7.66E-09	0.751
18	58817565	59585320	377	5.64E-05	8.18E-09	0.533
18	60823554	61439643	275	-9.75E-06	4.42E-09	0.883
18	59585959	60822264	549	4.57E-06	9.13E-09	0.962
18	61441801	62075853	293	-3.08E-05	4.06E-09	0.629
18	62078305	63110788	403	-3.19E-05	6.76E-09	0.698
18	63111628	64250279	396	2.21E-05	5.79E-09	0.772
18	65830606	66404071	323	-1.92E-05	5.28E-09	0.792
18	64251305	65830031	733	4.85E-05	1.37E-08	0.679
18	66404118	67569547	516	-8.20E-05	1.13E-08	0.440
18	67574191	69038112	511	-5.43E-05	1.07E-08	0.600
18	69039235	70094707	484	-3.58E-05	9.98E-09	0.720
18	70094975	70717286	329	-4.46E-05	6.58E-09	0.583
18	70719886	71243399	284	5.94E-05	6.08E-09	0.446
18	71244692	71892202	389	-7.62E-05	1.42E-08	0.523
18	71892443	72519795	346	7.66E-05	6.20E-09	0.330
18	75029584	75473982	288	1.22E-04	6.72E-09	0.137
18	72521276	73897594	733	1.30E-04	1.73E-08	0.322
18	73898846	75028501	623	2.68E-04	1.73E-08	0.041
18	75474664	75975253	233	-1.11E-05	5.37E-09	0.880
18	75976567	77151520	501	3.09E-06	1.31E-08	0.978
18	77151867	78017074	333	-1.13E-05	6.35E-09	0.887
19	157892	757412	226	2.30E-05	9.94E-09	0.817
19	759261	1089241	159	-4.15E-05	6.91E-09	0.618
19	1090803	1920712	260	1.29E-04	1.09E-08	0.217
19	1922476	2815372	338	-1.00E-05	1.24E-08	0.928
19	2816339	3301333	213	6.16E-05	8.29E-09	0.499
19	3304779	4347573	345	1.17E-04	1.46E-08	0.332
19	4349416	5191547	333	-8.25E-05	2.26E-08	0.583
19	5192372	5766090	218	3.13E-06	9.54E-09	0.974
19	5767585	6612779	262	-5.01E-06	9.21E-09	0.958
19	6614637	7309688	315	9.39E-05	1.24E-08	0.398
19	7312727	8684707	486	7.52E-05	1.85E-08	0.581
19	9013231	10029966	389	-3.77E-05	1.02E-08	0.709

19	11280183	12497411	332	-1.67E-04	9.54E-09	0.087
19	12500149	13903388	403	-2.13E-05	1.29E-08	0.851
19	10030690	11279257	438	8.24E-05	4.16E-08	0.686
19	13906229	15549939	594	-3.54E-05	1.97E-08	0.801
19	15553878	16251518	443	-1.49E-06	1.22E-08	0.989
19	17944900	18506666	241	3.13E-05	8.30E-09	0.731
19	16252072	17942927	622	-2.44E-04	2.11E-08	0.093
19	19873640	20520392	140	-4.41E-05	3.97E-09	0.484
19	18506815	19873269	429	-6.17E-05	1.16E-08	0.567
19	21379657	22036399	158	6.57E-07	4.05E-09	0.992
19	22037866	22803640	244	-3.34E-05	6.38E-09	0.676
19	22805247	24059424	282	1.11E-05	6.72E-09	0.892
19	24059507	28555771	269	3.87E-06	7.12E-09	0.963
19	28556284	28917565	189	-3.15E-05	4.95E-09	0.654
19	30113991	30858084	257	3.00E-05	9.17E-09	0.754
19	29318443	30113252	464	-3.90E-05	1.34E-08	0.736
19	30858965	32136133	462	-2.69E-04	1.36E-08	0.021
19	33764103	34067891	139	7.08E-05	4.74E-09	0.304
19	32145233	33763475	600	2.68E-05	1.73E-08	0.839
19	34068435	35331436	448	2.35E-06	1.41E-08	0.984
19	35333176	36045772	363	1.70E-07	1.31E-08	0.999
19	39371014	39757572	147	6.68E-05	5.24E-09	0.356
19	36047098	37454119	410	-1.71E-05	1.12E-08	0.872
19	41442597	41841098	164	2.43E-05	4.44E-09	0.715
19	37455512	39370456	573	-2.68E-05	2.02E-08	0.850
19	39757926	41440863	611	-1.66E-04	1.79E-08	0.216
19	41843461	43954915	363	-2.02E-04	1.83E-08	0.136
19	44882157	45255679	171	-2.73E-05	6.83E-09	0.741
19	43956095	44880657	396	6.99E-05	1.08E-08	0.501
19	45259002	46433733	397	-1.05E-04	1.33E-08	0.362
19	46433891	47333204	327	8.56E-06	1.24E-08	0.939
19	47333497	48509893	369	-1.33E-04	1.14E-08	0.214
19	48697961	49485758	336	-2.85E-04	1.08E-08	0.006
19	49491035	49923166	159	2.07E-06	4.36E-09	0.975
19	49925208	50774141	253	9.28E-05	8.22E-09	0.306
19	50774581	51331589	225	2.60E-05	8.70E-09	0.780
19	52217405	52604625	221	-2.03E-05	6.41E-09	0.800

19	51332061	52216563	531	1.32E-06	6.47E-08	0.996
19	52606936	53107086	221	-7.33E-05	8.52E-09	0.427
19	53107937	53537923	153	5.49E-06	6.59E-09	0.946
19	53538505	53899797	196	-1.62E-05	7.11E-09	0.847
19	54099626	54702450	222	-1.14E-04	1.15E-08	0.288
19	54704266	55174498	204	5.26E-05	8.83E-09	0.575
19	55175009	55791380	259	-1.08E-04	1.01E-08	0.284
19	55794266	56364684	248	8.26E-05	1.39E-08	0.483
19	56365323	56898342	290	-1.51E-05	1.20E-08	0.890
19	57952035	58531715	259	-3.93E-05	6.35E-09	0.621
19	56898853	57950377	491	4.48E-05	1.69E-08	0.731
19	58532756	59118784	238	-7.75E-05	6.72E-09	0.344
20	246392	887800	376	-1.27E-04	1.43E-08	0.288
20	888523	1445821	368	-2.29E-05	1.04E-08	0.822
20	1446580	1897442	255	-1.13E-05	5.69E-09	0.881
20	3083151	3655085	235	6.69E-06	5.56E-09	0.929
20	3655943	4051325	176	-3.53E-05	5.95E-09	0.647
20	1899473	3081680	688	2.68E-05	1.47E-08	0.825
20	4053020	4701211	402	-1.11E-05	1.19E-08	0.919
20	4701706	5304961	360	7.32E-05	8.42E-09	0.425
20	6637234	7065163	285	-9.85E-05	8.46E-09	0.284
20	5305400	6636139	709	-2.56E-04	1.65E-08	0.046
20	8601114	8881586	198	4.09E-05	3.20E-09	0.470
20	7066818	8600105	763	-6.45E-05	1.39E-08	0.584
20	8884009	9745450	471	-1.37E-05	9.33E-09	0.887
20	9746345	11013570	694	2.28E-05	1.64E-08	0.859
20	11014704	11534388	290	4.62E-05	4.96E-09	0.512
20	11534488	12153352	274	1.21E-04	5.08E-09	0.090
20	13201177	13666198	193	3.26E-06	4.00E-09	0.959
20	12153396	13199755	619	-2.56E-04	1.27E-08	0.023
20	13670311	14929217	484	-5.00E-05	8.93E-09	0.597
20	17449713	17905102	299	-1.02E-04	7.76E-09	0.246
20	15148231	16150464	704	1.17E-04	1.60E-08	0.353
20	17906490	18896415	440	-2.91E-05	7.47E-09	0.736
20	16151566	17449361	877	2.58E-05	1.78E-08	0.847
20	18896895	19707457	462	-9.31E-05	8.94E-09	0.325
20	21679930	22779512	406	2.04E-06	5.55E-09	0.978

20	19708351	21676556	817	-1.06E-05	1.56E-08	0.932
20	23568495	24053655	218	3.96E-05	3.76E-09	0.519
20	22779865	23568490	399	-2.89E-06	6.71E-09	0.972
20	24054931	25019099	416	-1.05E-05	6.64E-09	0.897
20	25020047	26319418	306	-1.84E-05	4.03E-09	0.772
20	26319535	30570889	230	-4.79E-05	3.06E-09	0.386
20	30574455	31613985	334	-7.48E-05	5.02E-09	0.291
20	33885155	34891416	221	2.24E-05	2.71E-09	0.667
20	34892880	35892530	205	-4.34E-05	3.42E-09	0.458
20	31615242	33883015	704	-1.01E-04	9.50E-09	0.298
20	35899774	36904375	395	-1.12E-04	6.26E-09	0.158
20	36905894	37875045	354	-4.07E-06	5.85E-09	0.958
20	37875309	38431408	230	1.38E-05	3.26E-09	0.809
20	38435302	39453533	470	-4.19E-05	8.57E-09	0.651
20	39455778	40584825	395	-1.03E-04	7.26E-09	0.229
20	40585126	41194048	393	-3.02E-05	8.29E-09	0.740
20	41195111	42188284	488	9.63E-06	1.08E-08	0.926
20	42188914	43257406	508	5.50E-05	9.88E-09	0.580
20	44960997	45649369	359	3.74E-05	7.30E-09	0.661
20	43259306	44960236	710	-1.47E-04	1.18E-08	0.177
20	45650139	46540102	374	6.91E-05	9.06E-09	0.468
20	48081983	48655016	227	5.09E-06	6.73E-09	0.950
20	48658113	49385779	312	2.18E-05	6.85E-09	0.793
20	46540587	48080850	769	-2.54E-05	1.39E-08	0.829
20	49387520	49945761	256	-1.72E-04	1.31E-08	0.134
20	49948016	50633472	360	1.99E-05	8.37E-09	0.828
20	50635347	51389135	319	-2.80E-05	5.53E-09	0.706
20	51389670	51884594	263	-1.93E-06	6.25E-09	0.981
20	51885269	52525657	291	2.13E-06	1.60E-08	0.987
20	52526840	53406846	483	6.19E-05	1.08E-08	0.551
20	53408035	54321635	406	3.93E-05	7.62E-09	0.653
20	54321811	55431836	547	6.98E-05	1.12E-08	0.510
20	56216001	56679942	290	3.11E-05	7.61E-09	0.722
20	55434683	56214465	498	9.28E-05	1.35E-08	0.425
20	56679960	57319010	352	-6.30E-05	7.72E-09	0.473
20	57320545	58295563	478	1.71E-05	1.28E-08	0.880
20	58296350	59361064	553	3.47E-05	1.13E-08	0.744

20	59361382	59892000	354	1.16E-05	9.36E-09	0.905
20	59892231	61091395	656	-2.30E-04	3.66E-08	0.230
20	61301855	62119318	399	-9.88E-05	1.16E-08	0.358
20	62119875	62962870	340	-4.93E-06	1.61E-08	0.969
21	15451950	16139409	423	-1.52E-04	9.07E-09	0.109
21	18457828	19151566	246	-1.40E-04	7.14E-09	0.097
21	16140244	17301757	441	7.65E-05	9.01E-09	0.420
21	17305471	18457656	407	7.06E-06	1.01E-08	0.944
21	19151940	20198061	541	-7.08E-05	1.12E-08	0.504
21	20199390	21022168	395	-3.82E-05	8.06E-09	0.671
21	21022840	21905609	384	7.23E-05	9.14E-09	0.449
21	24695869	25083563	190	-3.67E-05	4.97E-09	0.603
21	23270321	24695405	541	-8.79E-05	1.09E-08	0.400
21	25090174	25898082	385	-6.72E-05	7.31E-09	0.432
21	25899516	26465774	241	-6.45E-05	4.96E-09	0.360
21	21905658	23268216	669	-3.27E-05	1.36E-08	0.779
21	27777181	28205789	287	-1.45E-05	6.45E-09	0.857
21	26467796	27776736	496	8.17E-05	9.82E-09	0.410
21	28206883	28705495	308	3.62E-05	7.30E-09	0.672
21	28705732	29515396	352	1.26E-05	7.97E-09	0.888
21	30845873	32211778	531	-2.59E-05	8.94E-09	0.784
21	29515884	30844467	549	-8.35E-05	8.38E-09	0.362
21	32213678	33270766	453	-5.59E-05	9.76E-09	0.571
21	33273162	33988973	358	-8.64E-05	7.29E-09	0.311
21	33991368	35219895	507	-2.16E-04	2.47E-08	0.169
21	35220001	36188046	410	-2.01E-04	3.93E-08	0.311
21	37870518	38429082	265	-8.28E-05	5.16E-09	0.249
21	37305159	37868817	300	7.62E-06	5.57E-09	0.919
21	36190040	37303320	549	-5.37E-05	1.10E-08	0.609
21	38429389	39248065	402	-6.75E-05	6.60E-09	0.406
21	39250321	40115483	422	2.55E-05	9.53E-09	0.794
21	41260363	41705756	344	-4.40E-05	7.87E-09	0.620
21	40116044	41095241	608	-1.57E-04	1.14E-08	0.142
21	41707453	42543663	490	3.60E-05	1.05E-08	0.725
21	44690856	45598522	250	5.86E-05	6.71E-09	0.475
21	45603905	46174491	233	-3.00E-05	5.94E-09	0.697
21	42545250	43342046	529	-3.10E-04	2.86E-08	0.066

21	46174981	46740019	297	2.00E-05	5.87E-09	0.794
21	46741287	47491264	334	-5.52E-05	6.68E-09	0.500
21	43343190	44632513	758	1.18E-04	1.75E-08	0.373
21	47491975	48119635	293	-4.36E-05	5.07E-09	0.540
22	17187276	17569017	166	-5.42E-05	4.23E-09	0.404
22	17569439	18333467	393	1.19E-04	1.20E-08	0.279
22	18333902	19138442	274	4.83E-05	6.27E-09	0.542
22	19139368	20000428	424	-9.39E-05	1.28E-08	0.407
22	20001006	20952650	270	-2.02E-04	1.31E-08	0.077
22	20952972	21863717	294	3.68E-06	6.24E-09	0.963
22	23016570	23691564	209	2.81E-05	4.39E-09	0.671
22	21866569	23015302	361	2.43E-05	8.08E-09	0.787
22	23697301	24370194	304	4.55E-05	8.51E-09	0.622
22	25419524	26113333	224	1.57E-05	5.57E-09	0.833
22	24375019	25416292	357	1.45E-05	7.49E-09	0.867
22	26123092	26735929	447	-1.37E-04	1.17E-08	0.205
22	26736812	27294543	386	1.07E-05	1.05E-08	0.917
22	27295234	27653424	353	4.31E-05	1.20E-08	0.694
22	29456360	30604102	391	-3.74E-06	7.21E-09	0.965
22	31535872	32324140	238	-2.80E-05	3.87E-09	0.652
22	30605601	31534127	380	-1.03E-05	8.50E-09	0.911
22	27654838	29454477	730	-2.06E-04	1.94E-08	0.140
22	32324692	32856999	277	-5.54E-05	6.10E-09	0.478
22	32857285	33511391	411	2.21E-05	1.05E-08	0.829
22	33512106	33896495	177	-1.20E-05	4.97E-09	0.865
22	35865218	36131498	134	-3.23E-05	3.88E-09	0.604
22	33898114	34982457	618	-7.32E-05	1.31E-08	0.522
22	36132263	36922822	314	5.07E-05	7.52E-09	0.559
22	34983275	35862655	468	6.57E-05	8.36E-09	0.473
22	36923144	37558356	395	3.96E-05	1.06E-08	0.700
22	37560592	38467784	415	-3.78E-05	9.32E-09	0.696
22	38473398	39302783	269	1.11E-05	5.27E-09	0.878
22	39304509	40092866	319	1.09E-04	7.14E-09	0.195
22	40095174	41405753	318	4.31E-06	1.50E-08	0.972
22	41406919	42870441	427	-3.74E-06	7.90E-09	0.966
22	43187900	43645335	232	9.23E-05	3.46E-08	0.620
22	43646241	44317084	380	-7.95E-07	7.99E-09	0.993

22	44317416	44818986	414	1.09E-05	1.21E-08	0.921
22	44820796	45443845	417	-9.34E-05	1.32E-08	0.417
22	45444397	45895495	269	-1.21E-05	9.56E-09	0.902
22	45895856	46512413	260	-3.15E-05	7.54E-09	0.717
22	46513376	47106715	271	-4.96E-05	7.38E-09	0.564
22	47106800	47937655	432	-1.09E-05	9.70E-09	0.912
22	47937891	48479731	373	-7.04E-05	9.57E-09	0.472
22	49082979	49521080	312	-3.97E-05	1.05E-08	0.698
22	48481280	49081753	463	1.41E-05	1.45E-08	0.907
22	49522184	49751418	245	-4.97E-05	7.98E-09	0.578
22	49751512	50488153	334	3.20E-05	8.16E-09	0.723
22	50488995	51237713	280	6.62E-05	7.46E-09	0.443

SNP: single nucleotide polymorphisms. A Bonferroni correction was applied to account for multiple testing (*P* < ~0.05/2353).

Supplementary Table 2. Results from local genetic correlation analysis conducted for prostate cancer and COVID-19 phenotypes.

Chromosome	Start position	End position	Number of SNPs	Local genetic covariance	Variance	P-value
**Prostate cancer and SARS-CoV-2 infection**						
1	1103150	2320702	361	-9.51E-06	8.21E-10	0.740
1	2321099	3065568	234	4.20E-06	5.42E-10	0.857
1	3066761	3679461	290	-1.62E-04	1.95E-09	0.000
1	3679775	4749076	633	-3.65E-05	1.75E-09	0.383
1	4750013	5000086	168	-9.40E-06	4.71E-10	0.665
1	5000970	5471517	301	2.62E-05	8.33E-10	0.364
1	5472626	6133700	338	4.16E-05	8.49E-10	0.154
1	6134990	7245669	447	-2.21E-07	9.35E-10	0.994
1	7245922	7604778	205	-1.15E-05	5.20E-10	0.615
1	7604831	9067142	513	2.29E-05	1.11E-09	0.491
1	9067673	9959051	319	2.66E-05	1.03E-09	0.406
1	9959748	11774956	584	2.01E-05	1.45E-09	0.598
1	11776196	12147753	192	6.05E-06	3.79E-10	0.756
1	12150385	14625524	731	1.49E-05	2.05E-09	0.743
1	14626896	15423481	421	-4.78E-05	1.28E-09	0.182
1	15424441	17123862	613	-5.83E-05	1.52E-09	0.134
1	17176635	17872659	337	-4.41E-06	6.81E-10	0.866
1	17874843	18484945	341	-8.65E-06	9.56E-10	0.780

1	18486272	20022063	799	-1.24E-05	2.03E-09	0.783
1	20023833	20763899	370	-1.90E-05	1.16E-09	0.577
1	20765245	22634131	796	2.56E-05	1.82E-09	0.549
1	22636103	23082667	208	-2.05E-06	5.54E-10	0.931
1	23085227	24527501	491	-2.07E-05	1.13E-09	0.537
1	24528210	25305673	317	-1.42E-06	7.40E-10	0.958
1	25305783	26976424	612	3.31E-05	1.18E-09	0.335
1	26981479	28160199	205	3.98E-05	6.06E-10	0.106
1	28168872	29181525	242	2.23E-05	3.93E-10	0.261
1	29184254	30792409	735	-1.89E-05	1.55E-09	0.631
1	30792984	32042421	610	3.30E-05	1.02E-09	0.301
1	32042540	34016219	486	8.23E-06	8.12E-10	0.773
1	34017349	34797348	442	-2.23E-05	1.03E-09	0.486
1	34798014	35364965	274	-2.68E-05	7.79E-10	0.337
1	35366056	38196841	762	3.45E-05	1.64E-09	0.393
1	38459210	39535259	436	-4.31E-07	1.20E-09	0.990
1	39537291	40933221	445	1.03E-04	8.73E-10	0.001
1	40934284	42166782	487	-6.85E-05	9.67E-10	0.028
1	42168539	43365094	396	-2.17E-05	6.67E-10	0.400
1	43365565	44482312	462	7.24E-06	7.64E-10	0.793
1	44483704	46895641	764	5.63E-05	1.06E-09	0.083
1	46897698	47999344	386	1.04E-05	6.59E-10	0.686
1	48000156	50640662	790	1.21E-05	1.33E-09	0.741
1	50641944	53116453	445	3.88E-05	7.37E-10	0.153
1	53117066	54197141	419	1.49E-05	8.10E-10	0.600
1	54197688	55012825	295	-2.95E-05	7.50E-10	0.281
1	55012881	55479198	250	1.18E-05	6.50E-10	0.643
1	55479967	56413577	485	2.18E-05	1.03E-09	0.497
1	56414730	57585670	599	1.50E-05	1.23E-09	0.669
1	57585980	58535140	555	2.98E-05	1.02E-09	0.350
1	58536417	59306823	412	-5.48E-06	7.96E-10	0.846
1	59307290	59736866	187	-8.15E-06	4.67E-10	0.706
1	59739179	61257170	537	-4.87E-05	1.10E-09	0.141
1	61258378	62107806	382	5.00E-06	1.26E-09	0.888
1	62108681	63376404	518	-8.69E-06	1.05E-09	0.788
1	63379031	65008206	795	2.66E-05	1.75E-09	0.525
1	65010679	66773349	698	-1.16E-05	1.39E-09	0.756

1	66774350	67760291	537	2.23E-05	9.77E-10	0.475
1	67761365	69441414	724	-4.56E-06	1.62E-09	0.910
1	69441920	70486660	328	3.28E-05	8.30E-10	0.255
1	70490925	71818029	502	6.49E-06	1.00E-09	0.838
1	71822765	74326378	643	9.37E-06	1.04E-09	0.771
1	74326484	75992462	563	-2.14E-05	9.74E-10	0.493
1	75992778	76476360	183	-6.83E-06	2.81E-10	0.684
1	76477207	77139859	372	-2.70E-05	8.81E-10	0.364
1	77141039	79111862	686	2.57E-06	1.37E-09	0.945
1	79113455	80341320	483	-1.37E-05	8.95E-10	0.646
1	80341732	81056946	241	-1.34E-05	4.71E-10	0.536
1	81058295	82883310	839	-5.19E-06	1.92E-09	0.906
1	82884721	83990906	441	1.88E-07	8.59E-10	0.995
1	83991748	84728665	328	8.18E-06	5.79E-10	0.734
1	84729134	85940249	595	-4.22E-06	1.35E-09	0.908
1	85940875	86549498	270	1.53E-06	4.93E-10	0.945
1	86549569	87652502	417	-1.21E-05	8.34E-10	0.676
1	87654606	89447978	634	-2.79E-06	1.49E-09	0.942
1	89451664	91866856	883	1.46E-05	1.79E-09	0.729
1	91867046	93351477	560	9.74E-06	1.01E-09	0.759
1	93352491	94451007	318	-9.02E-08	4.91E-10	0.997
1	94452062	95199048	406	-7.27E-06	8.63E-10	0.804
1	95199771	96149941	435	4.03E-06	9.53E-10	0.896
1	96150318	97457893	467	5.00E-06	8.18E-10	0.861
1	97458722	98562260	517	-3.17E-05	7.76E-10	0.255
1	98564736	100064599	575	1.14E-05	1.24E-09	0.746
1	100065413	101682573	614	-5.62E-06	9.76E-10	0.857
1	101683165	102039858	221	6.85E-06	4.94E-10	0.758
1	102040268	102893372	341	-1.06E-05	7.02E-10	0.689
1	102894567	104369301	288	2.18E-06	5.80E-10	0.928
1	104373157	105192916	270	1.12E-06	5.81E-10	0.963
1	105193022	106641877	398	3.75E-05	7.74E-10	0.178
1	106642705	107432265	291	3.87E-06	5.63E-10	0.871
1	107432576	109075191	693	2.22E-05	1.63E-09	0.582
1	109075561	110301260	464	-2.14E-06	9.07E-10	0.943
1	110302505	111738116	679	-2.21E-05	1.56E-09	0.577
1	111739749	112560278	566	-3.77E-05	1.33E-09	0.302

1	112561693	114451307	682	3.85E-07	1.09E-09	0.991
1	114451386	115878242	635	-3.91E-05	1.30E-09	0.278
1	115879821	116884431	399	8.26E-06	8.34E-10	0.775
1	116884519	118837474	650	-3.21E-05	1.37E-09	0.386
1	118837888	119792751	365	-2.15E-06	6.61E-10	0.933
1	119793605	120347239	234	1.13E-05	5.60E-10	0.634
1	144998563	147347713	453	-4.35E-06	8.42E-10	0.881
1	147348339	151830844	601	-4.24E-06	2.11E-09	0.926
1	151832901	152535021	216	7.60E-06	3.10E-10	0.666
1	152535586	153163377	278	-6.69E-06	3.77E-10	0.731
1	153165450	154544651	506	2.55E-05	7.73E-10	0.359
1	154547292	156334116	551	-1.83E-05	3.97E-09	0.772
1	156335111	157154186	313	-4.52E-06	6.45E-10	0.859
1	157154324	158867538	820	2.16E-05	1.73E-09	0.603
1	158870085	160648549	764	1.02E-05	1.87E-09	0.813
1	160649926	161973976	510	5.19E-05	1.05E-09	0.109
1	161974584	162995355	563	-3.35E-05	1.20E-09	0.333
1	163000394	164410254	676	3.13E-06	1.22E-09	0.929
1	164412847	165190876	436	2.77E-05	1.20E-09	0.424
1	165191702	166363258	605	2.68E-05	1.38E-09	0.470
1	166364063	167158927	484	2.63E-06	9.12E-10	0.931
1	167159608	168168773	462	1.39E-05	1.02E-09	0.664
1	168169452	169077127	489	6.47E-06	1.23E-09	0.854
1	169078572	169521853	193	-4.68E-07	2.91E-10	0.978
1	169522554	170372503	446	-7.73E-06	7.82E-10	0.782
1	170375663	170914261	181	7.56E-06	3.73E-10	0.696
1	170915133	172647523	864	3.29E-05	1.46E-09	0.390
1	172648956	175384090	909	4.47E-07	1.36E-09	0.990
1	175385076	176416712	369	9.46E-06	6.81E-10	0.717
1	176418578	177431255	438	-1.09E-05	8.09E-10	0.701
1	177432237	178940717	621	-1.53E-05	1.17E-09	0.654
1	178943012	179676274	338	6.48E-07	5.36E-10	0.978
1	179678188	180567596	397	-2.80E-05	7.66E-10	0.311
1	180568720	181311800	309	-8.85E-07	5.60E-10	0.970
1	181313404	182293001	421	2.21E-05	8.73E-10	0.454
1	182294372	183796074	577	-3.50E-05	9.52E-10	0.256
1	183796618	185511423	737	5.32E-05	1.52E-09	0.173

1	185512838	187847742	807	-2.49E-05	1.43E-09	0.510
1	187848898	189756187	599	8.06E-06	8.78E-10	0.786
1	189758772	190107648	158	5.30E-06	2.64E-10	0.744
1	190107883	191432168	473	2.99E-05	7.56E-10	0.276
1	191433917	192444256	331	-8.97E-06	5.60E-10	0.705
1	192452631	193504005	346	1.73E-05	5.47E-10	0.460
1	193505535	194000243	197	-6.27E-07	3.33E-10	0.973
1	194000953	194733444	212	-1.20E-05	3.49E-10	0.521
1	194734415	195155161	176	4.51E-06	2.85E-10	0.789
1	195155293	195995510	312	3.37E-07	6.20E-10	0.989
1	195995948	197309603	347	2.13E-06	5.90E-10	0.930
1	197310096	198540028	417	-1.74E-05	6.91E-10	0.507
1	198540418	199578700	369	-9.05E-06	7.33E-10	0.738
1	199580285	201070422	658	-8.19E-05	1.32E-09	0.024
1	201070740	201365889	257	2.40E-05	6.02E-10	0.327
1	201366509	202820077	608	-1.68E-05	1.38E-09	0.651
1	202820815	203979519	488	-4.94E-05	1.32E-09	0.173
1	203980019	205458538	791	-1.26E-04	2.26E-09	0.008
1	205459511	206705609	336	7.76E-05	1.58E-09	0.051
1	206706419	208158787	548	2.76E-05	1.08E-09	0.400
1	210741816	211763053	432	5.35E-06	1.00E-09	0.866
1	208160220	210738604	1288	1.17E-05	2.84E-09	0.826
1	211763754	213578006	641	-5.38E-05	1.39E-09	0.149
1	213578592	215128675	756	1.80E-05	1.71E-09	0.663
1	215129087	216672805	623	1.84E-05	1.20E-09	0.597
1	216673326	217575665	684	-4.30E-08	1.59E-09	0.999
1	217577346	218860068	520	-1.84E-06	1.24E-09	0.958
1	218860456	220068681	530	-1.49E-05	9.61E-10	0.630
1	220069681	221856899	640	4.13E-05	1.25E-09	0.243
1	221857359	223630391	645	-1.93E-05	1.54E-09	0.623
1	223630619	224434769	280	-5.47E-06	5.89E-10	0.822
1	224435934	226143348	570	-7.30E-06	1.09E-09	0.825
1	226146348	226808294	242	-1.91E-05	5.23E-10	0.403
1	226808697	228100891	558	1.03E-05	1.00E-09	0.744
1	228101119	229075210	320	-4.60E-05	7.46E-10	0.092
1	229076157	230477970	634	3.83E-06	1.35E-09	0.917
1	230479324	231318678	503	2.03E-05	1.25E-09	0.565

1	231321215	232382234	476	2.53E-05	9.72E-10	0.418
1	232384356	232949914	349	-2.28E-05	8.14E-10	0.424
1	232950888	233750725	429	-3.76E-05	1.02E-09	0.239
1	233753991	234206593	224	2.48E-05	6.44E-10	0.330
1	234209087	235772624	702	2.34E-05	1.78E-09	0.579
1	235773790	236536723	314	2.53E-06	6.17E-10	0.919
1	236537770	237493854	566	2.89E-06	1.17E-09	0.933
1	237495080	239297872	909	-2.33E-05	2.21E-09	0.621
1	239299272	240562764	672	-2.68E-05	1.65E-09	0.509
1	240563367	241178287	352	-3.10E-05	1.14E-09	0.360
1	241178944	241766551	357	-8.02E-06	1.01E-09	0.800
1	241768617	242697031	497	-1.24E-05	1.37E-09	0.738
1	242700803	244105053	454	1.97E-05	7.69E-10	0.477
1	244106113	244927751	383	-2.84E-05	1.08E-09	0.387
1	244929262	246606531	851	5.38E-05	2.08E-09	0.238
1	246606926	246989171	161	-1.30E-05	5.36E-10	0.575
1	246991094	247838628	369	-1.79E-05	9.01E-10	0.552
1	247838870	248585259	281	1.14E-05	5.18E-10	0.616
2	11320	1158981	538	2.30E-05	1.20E-09	0.508
2	1159982	1681720	220	-1.56E-05	5.97E-10	0.523
2	1683187	3003071	698	7.08E-05	2.02E-09	0.115
2	3003472	4148069	589	-1.62E-05	1.54E-09	0.681
2	4148784	5017172	423	-8.02E-06	1.08E-09	0.807
2	5118816	6361343	633	-5.04E-05	1.95E-09	0.254
2	6363082	7322437	569	-3.84E-05	1.52E-09	0.324
2	7323039	8980346	703	-1.96E-06	1.92E-09	0.964
2	8980472	10440987	658	-7.80E-05	2.57E-09	0.124
2	10442541	10879340	236	6.03E-05	6.40E-10	0.017
2	10881867	11652586	377	6.36E-05	1.46E-09	0.096
2	11652795	12720977	565	-1.85E-05	1.81E-09	0.663
2	12723749	14648908	706	3.65E-05	1.62E-09	0.364
2	14649571	15720775	452	-2.42E-05	8.70E-10	0.412
2	15721423	16549199	386	4.63E-06	1.11E-09	0.890
2	16549538	18344242	768	7.97E-05	1.72E-09	0.055
2	18344821	18646822	144	-1.17E-05	3.38E-10	0.526
2	18647868	20059542	690	1.70E-05	1.64E-09	0.674
2	20062043	22427190	825	5.09E-05	2.63E-09	0.321

2	22428680	23855977	652	2.27E-05	1.58E-09	0.569
2	23857184	25485735	455	-2.48E-05	8.13E-10	0.384
2	25486770	26894217	438	-8.25E-06	9.71E-10	0.791
2	26894589	28597305	482	-2.33E-06	7.76E-10	0.933
2	28597624	29484922	455	5.42E-05	1.06E-09	0.095
2	29486078	29987735	338	-2.44E-06	7.69E-10	0.930
2	29988753	31009977	499	-1.35E-05	1.22E-09	0.700
2	31010212	32489851	591	-1.34E-05	1.22E-09	0.700
2	32493210	33361425	336	4.23E-06	7.98E-10	0.881
2	33362643	34279720	532	8.10E-06	1.33E-09	0.824
2	34280335	35607183	529	-3.11E-05	1.15E-09	0.360
2	35608923	36121342	278	-4.41E-07	5.78E-10	0.985
2	36122006	37004185	554	-2.20E-06	1.48E-09	0.954
2	37008066	38882278	873	-5.81E-05	2.22E-09	0.218
2	38882577	40077971	314	-9.31E-07	5.41E-10	0.968
2	40080343	40625847	349	-2.24E-05	7.91E-10	0.425
2	40626336	41381901	294	-9.59E-06	5.87E-10	0.692
2	41382889	42087411	359	1.53E-06	7.40E-10	0.955
2	42088016	42947335	404	-2.73E-05	8.01E-10	0.335
2	42949616	43307776	218	-1.83E-05	2.01E-09	0.683
2	43309247	44048346	326	-8.88E-05	2.19E-09	0.058
2	44049120	45194289	539	-1.50E-05	1.14E-09	0.656
2	45195022	45562814	268	-6.08E-06	9.38E-10	0.843
2	45563613	47052067	882	-6.36E-06	2.41E-09	0.897
2	47052788	47782756	370	-2.82E-05	1.16E-09	0.407
2	47782836	49112501	508	-2.13E-05	1.34E-09	0.559
2	49113132	50757671	842	-1.62E-06	1.86E-09	0.970
2	50758657	51667413	400	-8.80E-06	8.63E-10	0.765
2	51668142	53351493	835	-4.01E-06	1.82E-09	0.925
2	53352175	54599930	554	-2.03E-05	1.26E-09	0.567
2	54600556	56158646	724	-8.66E-06	1.64E-09	0.830
2	56160647	56599354	240	-4.15E-06	7.25E-10	0.877
2	56600739	57938354	492	-3.69E-05	1.02E-09	0.249
2	57939866	60449069	1014	-2.27E-05	2.38E-09	0.642
2	60449710	62420656	658	4.55E-05	1.29E-09	0.206
2	62425639	64623935	687	2.52E-06	4.73E-09	0.971
2	64626895	65226463	256	1.24E-05	6.97E-10	0.640

2	65228236	66374803	559	-4.02E-06	1.27E-09	0.910
2	66375016	67978287	798	-1.44E-05	1.94E-09	0.743
2	67981487	68871573	395	-3.38E-06	8.54E-10	0.908
2	68871710	70753882	812	3.89E-05	1.64E-09	0.337
2	70756057	71704491	467	-2.08E-05	1.19E-09	0.545
2	71704844	73172972	469	3.07E-06	1.07E-09	0.925
2	73174848	74121000	254	2.50E-06	4.09E-10	0.901
2	74121278	76615798	1069	-2.84E-05	2.45E-09	0.566
2	76616819	77384991	368	-1.26E-05	8.54E-10	0.667
2	77386522	79007966	562	-1.13E-05	1.35E-09	0.758
2	80804991	81588311	231	6.88E-06	4.46E-10	0.745
2	79008155	80804284	1066	2.14E-05	2.67E-09	0.678
2	81588482	83604057	599	-4.90E-07	9.25E-10	0.987
2	83604796	85283713	481	7.72E-06	8.71E-10	0.794
2	85284353	86559358	572	-1.79E-05	1.22E-09	0.608
2	86562902	89135764	464	-1.79E-05	9.63E-10	0.565
2	92324281	98790763	461	1.10E-05	7.94E-10	0.696
2	98791433	100195879	407	-3.13E-05	6.65E-10	0.225
2	100197638	101338509	453	-8.70E-07	8.35E-10	0.976
2	101338644	102688272	529	7.03E-05	1.38E-09	0.058
2	102688495	103262415	357	2.51E-05	6.43E-10	0.322
2	103264434	104481488	294	-1.20E-05	6.12E-10	0.626
2	104485456	105871610	462	-1.46E-05	1.09E-09	0.658
2	105872982	107406122	724	-1.94E-05	1.92E-09	0.657
2	107406885	108427822	407	-2.04E-05	8.56E-10	0.486
2	108427959	110109210	583	4.18E-06	1.21E-09	0.905
2	110109222	112924907	587	5.74E-05	3.14E-09	0.305
2	112924973	114846282	718	-1.65E-06	1.49E-09	0.966
2	114848115	116770829	607	7.89E-06	1.30E-09	0.827
2	116772945	117727045	304	2.41E-06	5.22E-10	0.916
2	117728904	119581280	716	-1.69E-05	1.64E-09	0.676
2	119581439	121070811	505	-8.50E-06	1.08E-09	0.796
2	121073253	123241656	763	7.62E-05	1.91E-09	0.082
2	123244233	124730999	532	-9.11E-06	1.04E-09	0.777
2	124733246	125735879	428	-3.21E-06	9.95E-10	0.919
2	125741434	127372889	518	-2.24E-06	9.65E-10	0.943
2	127374341	129310292	756	-3.03E-05	1.78E-09	0.473

2	129312188	129864416	215	-3.02E-07	5.33E-10	0.990
2	129864730	131463038	407	8.06E-06	8.72E-10	0.785
2	131466136	133030349	226	-1.62E-05	5.60E-10	0.492
2	133034031	133950661	431	-2.48E-06	1.10E-09	0.941
2	133951544	136822267	1074	-5.83E-06	2.12E-09	0.899
2	136825272	138619715	738	-2.38E-05	1.60E-09	0.553
2	138620371	139412389	267	8.31E-06	5.22E-10	0.716
2	139413312	140902261	570	1.27E-05	1.27E-09	0.722
2	140902383	141803496	400	-1.24E-05	8.95E-10	0.679
2	141803602	143254981	699	2.95E-05	1.65E-09	0.468
2	143255262	145301314	618	-2.73E-05	1.66E-09	0.502
2	145303576	147252762	524	-4.20E-05	1.10E-09	0.205
2	147255216	149268931	497	1.98E-05	9.44E-10	0.519
2	149270710	151184680	609	-9.29E-06	1.37E-09	0.802
2	151191481	152490219	540	-7.09E-05	1.17E-09	0.038
2	152491269	153369892	311	-5.85E-06	5.74E-10	0.807
2	153371042	154414456	449	1.17E-05	1.14E-09	0.730
2	154415112	155208173	297	1.85E-08	6.78E-10	0.999
2	155209736	156568446	482	-1.71E-05	1.00E-09	0.590
2	156568806	158165818	441	-1.20E-06	1.07E-09	0.971
2	158173710	159574883	463	-1.89E-05	1.13E-09	0.574
2	159575554	161077823	603	-5.53E-06	1.23E-09	0.875
2	161078891	163358537	678	2.70E-05	1.18E-09	0.431
2	163359084	165200324	484	-6.71E-05	1.13E-09	0.046
2	165202539	167120956	737	6.30E-05	1.75E-09	0.132
2	167123247	168427789	400	1.46E-06	7.92E-10	0.959
2	168431583	169176766	322	-2.44E-05	8.68E-10	0.408
2	169178092	170973262	789	-2.85E-05	1.79E-09	0.500
2	170973461	172086127	479	-7.68E-06	1.21E-09	0.825
2	172088943	173586201	589	7.24E-05	3.26E-09	0.205
2	173588663	175585616	785	3.62E-05	2.38E-09	0.458
2	175586510	177326682	629	3.19E-05	1.47E-09	0.405
2	177327202	178551958	558	-8.07E-06	1.15E-09	0.812
2	178552788	180138313	565	-4.34E-05	1.18E-09	0.206
2	180139219	180661233	324	-7.65E-06	9.70E-10	0.806
2	180662102	181627195	413	-5.46E-05	9.62E-10	0.079
2	181628139	182840114	361	-2.75E-05	7.53E-10	0.316

2	182845657	184442530	601	1.36E-05	1.22E-09	0.698
2	184443166	185277576	257	6.83E-06	4.88E-10	0.757
2	185279087	186731282	380	-1.77E-05	6.77E-10	0.495
2	186731309	188540877	478	1.38E-05	8.21E-10	0.631
2	188542361	190794689	527	3.11E-05	1.04E-09	0.334
2	190797341	191870624	350	-5.01E-05	6.42E-10	0.048
2	191873553	192695107	283	-2.26E-05	7.95E-10	0.422
2	192696255	195109845	444	-2.77E-05	8.00E-10	0.328
2	195117932	197307918	619	-9.94E-06	1.28E-09	0.781
2	197308707	198140953	270	-1.30E-05	4.74E-10	0.550
2	198141746	200498970	621	9.35E-06	1.04E-09	0.772
2	200504209	201571624	452	-5.34E-06	9.09E-10	0.859
2	201572564	202829668	382	-6.51E-05	1.41E-09	0.083
2	202830132	204814896	525	2.33E-05	1.07E-09	0.475
2	204815902	206043539	517	8.98E-06	1.32E-09	0.805
2	206045927	207721890	728	4.23E-05	1.70E-09	0.305
2	207722425	208639154	389	6.15E-05	1.22E-09	0.079
2	208641174	209940909	408	3.76E-06	8.92E-10	0.900
2	209941332	210832981	269	-1.65E-06	5.01E-10	0.941
2	210838781	212377672	605	-6.38E-07	1.32E-09	0.986
2	212377759	212845716	216	-2.11E-05	6.25E-10	0.398
2	212845994	215005050	818	-2.42E-05	1.71E-09	0.557
2	215006557	216448007	604	-2.08E-05	1.35E-09	0.572
2	216449570	217184275	343	-2.12E-06	9.08E-10	0.944
2	217184715	218615634	620	-2.99E-05	1.50E-09	0.439
2	218616633	219671910	401	-1.62E-05	7.13E-10	0.543
2	219675067	220774229	409	2.26E-05	8.41E-10	0.436
2	220775971	221445218	359	-3.84E-05	8.93E-10	0.199
2	221445953	222985126	638	4.08E-05	1.58E-09	0.305
2	222985611	224002780	434	3.66E-05	1.05E-09	0.258
2	224003069	225832409	736	-8.25E-07	1.63E-09	0.984
2	225833758	227531180	618	-1.90E-05	1.23E-09	0.587
2	227532464	228456451	380	-5.65E-06	8.00E-10	0.842
2	228457117	229368530	433	-2.55E-06	1.09E-09	0.938
2	229368812	230282624	396	3.16E-05	9.65E-10	0.309
2	230283310	231322125	471	7.96E-05	1.25E-09	0.024
2	231322831	232790491	543	3.12E-05	1.46E-09	0.414

2	232791948	233964632	468	-3.98E-05	1.04E-09	0.217
2	233968153	234699046	368	1.71E-05	6.48E-10	0.503
2	234699366	235626316	630	-2.96E-05	1.77E-09	0.481
2	235627087	236619385	515	-8.00E-06	1.66E-09	0.845
2	236619713	238209382	608	1.77E-05	1.51E-09	0.649
2	238209871	239122917	445	-5.63E-05	1.31E-09	0.120
2	239123082	239801883	289	-1.72E-05	6.86E-10	0.512
2	239832097	240325991	242	1.21E-05	7.81E-10	0.664
2	240327154	240961852	336	-2.93E-05	1.19E-09	0.395
2	240962194	241907076	460	1.77E-05	1.55E-09	0.653
2	241909927	243185680	317	5.32E-05	1.77E-09	0.206
3	60197	1128944	484	3.35E-05	1.55E-09	0.394
3	1129028	1527748	339	2.49E-05	1.17E-09	0.467
3	1528147	2423237	601	-1.66E-05	1.73E-09	0.689
3	2423999	2769230	241	-1.52E-05	8.62E-10	0.604
3	2769392	3364279	476	1.09E-05	1.97E-09	0.806
3	3364442	4430024	630	1.57E-05	1.72E-09	0.706
3	4430848	5564422	653	5.35E-05	2.23E-09	0.257
3	5565384	6421659	513	2.74E-06	1.56E-09	0.945
3	6422814	7509288	575	8.91E-06	1.57E-09	0.822
3	7509715	7991133	371	9.26E-07	9.81E-10	0.976
3	7991981	9194649	780	-1.07E-04	2.26E-09	0.025
3	9195331	10526438	649	-1.53E-05	1.78E-09	0.716
3	10526803	11220278	388	1.61E-06	1.35E-09	0.965
3	11221721	12858028	673	5.10E-05	1.65E-09	0.210
3	12859004	13523338	332	4.05E-05	1.01E-09	0.202
3	13524741	14309204	367	-4.92E-07	1.11E-09	0.988
3	14309548	14816364	329	1.39E-05	1.08E-09	0.673
3	14816745	16661587	815	-7.64E-05	2.08E-09	0.094
3	16662488	18454062	540	-6.21E-06	1.29E-09	0.863
3	18457304	18886128	167	8.74E-07	3.95E-10	0.965
3	18886158	20752492	602	2.54E-05	1.61E-09	0.527
3	20753368	21522703	363	3.18E-05	1.10E-09	0.338
3	21523116	22102707	449	-1.41E-06	1.35E-09	0.969
3	22103146	23122359	464	-1.58E-05	1.25E-09	0.656
3	23123318	23802647	259	5.73E-06	7.11E-10	0.830
3	25460109	26392563	317	4.95E-06	7.15E-10	0.853

3	23803541	25459497	887	-2.95E-05	2.49E-09	0.554
3	26393305	27606695	467	1.29E-06	1.07E-09	0.969
3	27609447	29409547	794	6.22E-06	2.31E-09	0.897
3	29409589	30716602	620	-3.88E-06	1.85E-09	0.928
3	30717661	31720982	438	-5.47E-06	9.84E-10	0.862
3	31721774	33011510	533	6.54E-06	1.78E-09	0.877
3	33012981	33986974	341	1.55E-05	7.69E-10	0.575
3	33988115	35241960	454	5.23E-06	1.12E-09	0.875
3	35243764	36481385	482	-4.41E-05	1.13E-09	0.190
3	36484375	38078255	627	-6.57E-06	1.48E-09	0.864
3	38080101	38723967	263	2.75E-06	6.40E-10	0.913
3	38724849	40649760	701	-1.55E-05	1.54E-09	0.694
3	40660283	42184173	434	1.73E-05	9.52E-10	0.574
3	42184361	45162316	1057	5.85E-05	2.42E-09	0.234
3	45165430	46657162	653	2.46E-05	4.23E-09	0.705
3	46657428	48525955	411	1.82E-05	7.78E-10	0.515
3	48531227	52214640	856	1.71E-05	1.60E-09	0.670
3	52215002	54078568	705	9.46E-08	1.47E-09	0.998
3	54080241	55731145	920	3.32E-05	2.72E-09	0.525
3	55733390	58063819	873	-3.89E-05	1.92E-09	0.376
3	58067795	58700675	307	3.47E-05	7.01E-10	0.190
3	58700802	60347135	936	-2.96E-05	2.86E-09	0.580
3	60347701	61277928	614	4.94E-06	1.67E-09	0.904
3	61279726	62881664	855	1.54E-06	2.70E-09	0.976
3	62882619	63504979	310	2.59E-05	8.09E-10	0.363
3	63505191	64473972	483	-2.25E-05	1.41E-09	0.549
3	64475164	65337581	550	-1.66E-05	1.64E-09	0.683
3	65340365	66169323	368	-2.95E-05	1.20E-09	0.395
3	66170253	67294968	419	8.66E-06	1.13E-09	0.797
3	67299401	69197507	756	-4.74E-05	1.85E-09	0.271
3	69205190	70288126	413	9.59E-07	1.09E-09	0.977
3	70289919	71418327	409	-2.73E-06	1.73E-09	0.948
3	71421152	73238935	813	-5.32E-05	2.64E-09	0.301
3	73239448	75376291	877	-7.46E-06	2.39E-09	0.879
3	75377075	76854466	345	2.44E-06	8.25E-10	0.932
3	76855676	77507524	288	-1.86E-05	7.80E-10	0.506
3	77508835	79022673	488	1.13E-05	1.24E-09	0.749

3	79024541	81187867	525	-1.21E-06	1.07E-09	0.970
3	81190039	82446513	356	-4.22E-06	6.85E-10	0.872
3	82446746	83946780	382	1.91E-05	8.33E-10	0.509
3	83947451	85090935	310	-5.73E-07	6.89E-10	0.983
3	85093629	86734415	435	6.15E-05	8.54E-10	0.035
3	86736368	87407921	210	-5.96E-05	2.95E-09	0.273
3	87408634	88725583	376	-2.36E-05	8.00E-10	0.404
3	88725754	94202010	445	8.03E-07	9.74E-10	0.979
3	94202771	96056259	472	-7.27E-06	9.03E-10	0.809
3	96679063	98299365	463	8.55E-07	9.95E-10	0.978
3	98300731	100591006	762	1.08E-05	1.55E-09	0.784
3	100591848	101808006	473	1.68E-04	2.45E-09	0.001
3	101808379	103025517	322	-8.29E-07	7.34E-10	0.976
3	103025715	103698105	210	1.79E-05	4.84E-10	0.417
3	103700909	104579408	324	1.87E-05	7.76E-10	0.502
3	104580063	104878270	154	-1.14E-06	3.63E-10	0.952
3	104879008	106330349	517	3.27E-05	1.34E-09	0.373
3	106332831	107729547	480	1.10E-04	2.14E-09	0.018
3	107730020	108635884	340	-3.92E-06	8.20E-10	0.891
3	108636466	109961316	401	2.29E-05	8.65E-10	0.437
3	109962369	111416310	404	1.43E-05	8.81E-10	0.631
3	111416451	112182995	420	6.48E-06	9.91E-10	0.837
3	112183360	113323793	432	1.71E-06	1.35E-09	0.963
3	113324119	115280312	582	2.39E-05	1.52E-09	0.540
3	115282058	116450487	509	-1.79E-05	1.31E-09	0.621
3	116452274	117998840	678	3.73E-05	1.96E-09	0.399
3	120521733	121335461	214	-1.95E-06	4.24E-10	0.925
3	118000136	120520317	1012	-1.01E-04	2.39E-09	0.039
3	121336367	122692039	596	4.19E-05	1.35E-09	0.254
3	122693533	123196819	219	-6.75E-06	5.38E-10	0.771
3	123200440	124677278	597	-1.61E-06	1.46E-09	0.966
3	124678230	125875831	442	-4.28E-06	1.16E-09	0.900
3	125876862	126213162	202	3.52E-05	4.95E-10	0.114
3	126213467	127471230	536	-1.12E-05	1.33E-09	0.757
3	127471641	130243259	811	-2.14E-04	5.61E-09	0.004
3	130244120	131373405	333	-4.19E-05	6.70E-10	0.106
3	131373740	132115911	443	7.67E-06	1.48E-09	0.842

3	132117285	133128449	407	2.99E-06	9.22E-10	0.922
3	133129272	134383242	550	-2.26E-05	1.52E-09	0.562
3	134385789	137369181	1106	5.50E-05	2.46E-09	0.267
3	137370076	138662229	384	3.78E-05	1.42E-09	0.315
3	138665796	140275652	774	4.96E-05	2.00E-09	0.267
3	140276484	142863564	912	-4.34E-06	2.09E-09	0.924
3	142864473	143494546	387	-1.08E-05	1.32E-09	0.767
3	143495679	145663879	729	1.98E-05	1.65E-09	0.627
3	145664135	146722271	402	1.80E-05	9.85E-10	0.566
3	146722383	148316359	475	-2.40E-05	1.25E-09	0.497
3	148316766	149988630	750	3.73E-05	2.05E-09	0.410
3	149994553	151178708	481	-1.07E-05	1.27E-09	0.765
3	151178836	151904190	289	1.14E-05	8.84E-10	0.701
3	151905386	153054901	368	7.08E-06	1.03E-09	0.825
3	153056839	154181836	468	1.85E-05	1.34E-09	0.613
3	154183562	156005200	508	5.78E-06	1.30E-09	0.872
3	156005905	157311596	529	2.40E-05	1.27E-09	0.499
3	157312028	159272972	716	-1.46E-06	1.57E-09	0.971
3	159274785	161523859	700	1.66E-05	1.51E-09	0.669
3	161524489	162065286	249	7.80E-07	7.39E-10	0.977
3	162067622	163250087	376	9.82E-06	7.75E-10	0.724
3	163251056	164816939	494	-3.50E-07	9.56E-10	0.991
3	164820183	167116849	615	1.77E-05	1.41E-09	0.638
3	167117143	167912878	293	-3.49E-05	6.47E-10	0.170
3	167913564	170158128	714	4.30E-06	4.59E-09	0.949
3	170159134	171311936	486	1.18E-05	1.54E-09	0.764
3	171312880	172965694	658	2.03E-05	1.94E-09	0.644
3	172966405	174274308	526	-5.69E-05	1.34E-09	0.121
3	174275336	175049294	365	1.05E-05	1.03E-09	0.745
3	175050262	176268375	462	-1.03E-05	1.21E-09	0.768
3	176269091	177530606	501	4.69E-06	1.42E-09	0.901
3	177531847	179359242	627	5.68E-05	1.58E-09	0.153
3	179366819	181426554	525	-2.09E-06	1.27E-09	0.953
3	181431571	182975975	457	5.72E-05	1.34E-09	0.118
3	182976382	183762614	363	-3.65E-05	7.91E-10	0.194
3	183762644	185262137	590	1.89E-05	1.77E-09	0.653
3	185263467	186780913	684	5.67E-06	2.24E-09	0.905

3	186782999	187220633	227	-7.39E-06	6.61E-10	0.774
3	187220777	188201380	508	-3.15E-05	1.62E-09	0.434
3	188202550	189531496	585	-8.12E-05	1.95E-09	0.066
3	189531911	190303805	414	-4.91E-05	1.22E-09	0.159
3	190304172	190992002	286	-7.36E-06	7.36E-10	0.786
3	190993386	191840443	362	1.60E-05	9.22E-10	0.598
3	191840983	192753870	381	-2.69E-05	1.01E-09	0.397
3	192754177	193885973	516	2.54E-05	1.48E-09	0.508
3	193886289	194464791	287	6.00E-06	9.18E-10	0.843
3	194465233	194756339	146	6.22E-06	5.79E-10	0.796
3	194756597	195854787	325	-7.23E-06	1.41E-09	0.848
3	195855401	196414159	209	-1.60E-05	6.62E-10	0.534
3	196415355	197962382	529	4.02E-07	1.27E-09	0.991
4	10642	685993	149	1.30E-07	2.40E-10	0.993
4	686563	1422863	301	-4.33E-05	7.22E-10	0.107
4	1478711	3612028	639	4.08E-05	1.27E-09	0.252
4	3613164	4263579	147	1.96E-05	5.90E-10	0.419
4	4264705	5044744	440	4.76E-06	1.06E-09	0.884
4	5047695	5971152	587	1.36E-05	1.59E-09	0.733
4	5971634	7135912	602	-8.45E-05	1.91E-09	0.053
4	7136158	8054472	690	-5.53E-05	2.16E-09	0.234
4	8055323	9408136	371	-4.99E-05	1.03E-09	0.119
4	9408158	10696229	611	2.44E-06	9.27E-10	0.936
4	10696913	12198579	733	9.85E-06	1.45E-09	0.796
4	12201002	13002341	347	-9.07E-06	5.77E-10	0.706
4	13002905	14394452	629	-3.40E-05	1.44E-09	0.369
4	14394902	15849986	606	-7.77E-06	1.19E-09	0.822
4	15850685	16520118	382	2.31E-05	8.52E-10	0.428
4	16521003	18752293	861	-6.44E-07	1.64E-09	0.987
4	18752722	20084049	441	3.91E-05	8.65E-10	0.184
4	20084563	21292213	550	3.61E-05	1.13E-09	0.284
4	21292651	22393873	439	1.24E-05	8.26E-10	0.665
4	22395570	23563570	369	2.38E-05	7.72E-10	0.392
4	23563931	24992827	684	-1.41E-05	1.56E-09	0.721
4	24993167	26468997	679	3.76E-05	2.42E-09	0.445
4	26473220	27903383	509	6.74E-06	1.12E-09	0.840
4	27904556	28871862	388	-2.09E-06	7.04E-10	0.937

4	28872833	30453156	508	1.22E-05	1.00E-09	0.701
4	30453619	31739254	482	2.93E-06	9.26E-10	0.923
4	31740009	32592949	294	-1.91E-05	5.45E-10	0.414
4	32593453	33640100	205	1.21E-05	3.47E-10	0.515
4	33640864	35150284	380	-8.70E-06	4.66E-10	0.687
4	35151237	36301997	434	-1.18E-05	8.02E-10	0.676
4	36304377	37534798	577	1.86E-05	1.27E-09	0.602
4	37536119	37886455	212	-2.80E-05	5.82E-10	0.245
4	37887735	38974458	602	-3.67E-05	1.55E-09	0.351
4	38976533	40199852	440	-3.14E-05	9.55E-10	0.309
4	40200304	40869208	304	-2.56E-05	9.72E-10	0.412
4	40870402	42662283	725	-6.03E-05	1.49E-09	0.119
4	42662718	43962569	451	1.05E-05	7.87E-10	0.707
4	43963518	45187835	414	-2.33E-05	6.75E-10	0.370
4	45188855	47543891	621	8.24E-07	1.00E-09	0.979
4	47544012	48226267	277	1.21E-05	4.33E-10	0.561
4	48227642	53412129	409	2.28E-05	4.47E-10	0.281
4	53413674	55202613	631	3.57E-06	1.07E-09	0.913
4	55202697	57418400	884	4.45E-05	1.79E-09	0.293
4	57420107	58696072	630	-2.38E-05	1.44E-09	0.530
4	58697912	59358251	234	-2.92E-06	4.57E-10	0.892
4	59358729	60145179	251	-2.95E-06	4.82E-10	0.893
4	60145181	61371992	375	7.89E-06	6.70E-10	0.761
4	61372415	61872517	167	-2.49E-06	3.14E-10	0.888
4	61872656	64117718	644	-4.59E-06	1.29E-09	0.898
4	64118963	65300308	243	-1.16E-05	3.59E-10	0.539
4	65562213	66598615	369	1.24E-05	6.75E-10	0.633
4	66599449	68058793	478	-1.61E-05	8.78E-10	0.588
4	68059850	68455531	160	1.40E-05	2.78E-10	0.402
4	68456074	69954151	270	1.16E-05	6.33E-10	0.645
4	69954297	70517143	270	4.02E-06	3.73E-10	0.835
4	70517792	71516586	418	1.44E-05	6.49E-10	0.572
4	72416258	75196731	712	-1.50E-06	1.86E-09	0.972
4	75555658	76283179	293	1.69E-05	6.45E-10	0.505
4	76284248	77128724	364	3.48E-06	7.46E-10	0.899
4	77129568	78333381	607	8.94E-06	1.13E-09	0.790
4	78333798	79469226	505	2.48E-05	8.59E-10	0.398

4	79470550	80994124	436	6.73E-06	8.47E-10	0.817
4	80995881	81978151	261	1.45E-06	4.49E-10	0.945
4	81979993	83012967	403	2.34E-05	6.80E-10	0.369
4	83017789	83994716	385	2.13E-05	6.54E-10	0.404
4	83998014	85080933	430	4.09E-05	1.13E-09	0.224
4	85081980	87980070	902	8.19E-06	1.41E-09	0.827
4	87980748	89236855	568	5.03E-06	1.21E-09	0.885
4	89238369	90126190	323	-5.06E-05	7.13E-10	0.058
4	90126331	91453993	447	-2.99E-05	8.73E-10	0.311
4	91456722	94229509	751	1.30E-05	1.45E-09	0.732
4	94230511	95596188	496	-2.16E-05	1.08E-09	0.511
4	95596744	96181408	236	3.96E-06	4.86E-10	0.858
4	96182669	96464931	212	-6.00E-06	5.29E-10	0.794
4	96466095	97911191	514	2.25E-05	9.07E-10	0.455
4	97911459	99422081	372	4.36E-06	4.02E-10	0.828
4	99423280	100322786	399	7.04E-06	6.63E-10	0.785
4	100323029	101897750	598	-6.63E-06	9.94E-10	0.833
4	101898276	103387944	551	-3.13E-05	1.40E-09	0.403
4	103388441	104802530	435	-1.64E-05	5.63E-10	0.491
4	104803329	106479425	508	1.15E-04	4.30E-09	0.079
4	106482185	108468632	628	-2.70E-05	1.09E-09	0.414
4	108469756	109979131	575	-1.98E-06	9.84E-10	0.950
4	109980374	112204254	780	4.94E-05	1.61E-09	0.219
4	112204259	114902479	1024	-8.66E-06	1.88E-09	0.842
4	117275542	118219755	308	1.34E-05	5.82E-10	0.577
4	114904730	117275534	678	1.75E-05	1.02E-09	0.583
4	118220292	119945314	632	-1.44E-05	1.13E-09	0.667
4	119946706	121391843	519	3.68E-06	8.13E-10	0.897
4	121393020	121973864	244	1.36E-05	5.06E-10	0.546
4	121976226	123561869	551	2.14E-05	1.01E-09	0.501
4	123563078	125863762	773	-3.57E-05	1.48E-09	0.354
4	125863974	127236805	494	-4.55E-06	1.35E-09	0.902
4	127237363	129514427	625	-2.64E-06	9.21E-10	0.931
4	129514448	130364383	286	-4.99E-06	5.54E-10	0.832
4	130364741	130949530	194	1.09E-06	3.29E-10	0.952
4	130950008	131948861	379	-1.05E-05	7.66E-10	0.705
4	131951466	133741365	442	-3.60E-06	7.36E-10	0.894

4	133741753	135744132	532	-3.34E-05	9.13E-10	0.269
4	135749591	137247266	391	8.02E-06	8.18E-10	0.779
4	137247703	137987679	298	1.45E-05	7.07E-10	0.585
4	137988838	139114529	404	2.43E-05	7.94E-10	0.388
4	139115093	139552965	239	6.26E-06	5.49E-10	0.789
4	139553359	140546206	342	-1.68E-05	6.51E-10	0.509
4	140548713	141751418	489	-1.65E-05	1.35E-09	0.653
4	141752960	142807957	391	-4.58E-05	8.62E-10	0.119
4	142808503	145021868	627	1.42E-06	8.40E-10	0.961
4	145024452	148047972	852	2.63E-05	1.50E-09	0.498
4	148053679	150632409	780	1.33E-05	1.35E-09	0.719
4	150634191	153226998	838	-3.61E-06	1.25E-09	0.919
4	153233476	154947363	615	-2.97E-05	1.17E-09	0.386
4	154949091	156604003	632	-1.99E-05	1.38E-09	0.592
4	156604173	158112985	581	-5.90E-06	1.24E-09	0.867
4	158114105	160356254	644	-4.95E-06	1.08E-09	0.880
4	160357877	160822830	147	5.45E-06	2.34E-10	0.721
4	161056173	161796696	321	8.05E-06	6.48E-10	0.752
4	162151565	162629423	187	-5.18E-06	5.39E-10	0.823
4	162629512	163181531	216	7.94E-06	4.10E-10	0.695
4	163183341	163726178	186	-1.16E-05	4.25E-10	0.574
4	163728264	164910818	398	-5.90E-06	7.89E-10	0.834
4	164913270	165817608	368	-5.95E-06	7.67E-10	0.830
4	165820093	166609852	327	1.70E-05	7.58E-10	0.536
4	166611193	167643023	297	-9.86E-07	6.78E-10	0.970
4	167643601	169568082	733	-2.71E-05	1.49E-09	0.484
4	169569516	170676328	421	3.15E-05	7.70E-10	0.257
4	170678993	172735897	573	-3.64E-05	1.01E-09	0.252
4	172737462	173760563	310	-2.66E-05	5.36E-10	0.250
4	173761916	174832248	301	6.59E-05	8.57E-10	0.024
4	174833988	176193705	541	-1.83E-05	1.42E-09	0.628
4	176199091	177290105	362	-3.47E-05	8.65E-10	0.238
4	177290945	177972278	282	4.37E-06	5.74E-10	0.855
4	177974062	178758792	336	-1.41E-05	9.52E-10	0.647
4	178758930	180119509	494	3.90E-05	1.10E-09	0.240
4	180120693	181732608	834	3.03E-05	2.26E-09	0.523
4	181732746	182420390	387	2.23E-05	1.27E-09	0.532

4	184283411	185477454	594	-7.69E-05	1.94E-09	0.081
4	182420863	184283167	1001	4.68E-06	3.02E-09	0.932
4	185477964	185932497	229	-7.92E-06	4.72E-10	0.716
4	185934003	187113041	594	-3.18E-05	1.65E-09	0.434
4	187114073	188687052	716	-1.20E-05	1.85E-09	0.780
4	188687155	189524815	363	3.34E-05	1.28E-09	0.351
4	189525752	190088167	270	1.71E-05	7.11E-10	0.521
4	190088768	191043878	201	1.06E-05	5.29E-10	0.646
5	10056	1267356	414	3.39E-05	9.09E-10	0.261
5	1270983	1762678	242	3.50E-06	1.38E-09	0.925
5	1763566	2369337	388	3.47E-05	2.39E-09	0.478
5	2369710	2979000	434	4.26E-05	1.30E-09	0.237
5	2979710	3600334	481	-9.17E-06	1.20E-09	0.792
5	3601924	4222111	306	2.53E-05	7.97E-10	0.371
5	4223577	5137474	433	-1.71E-05	1.01E-09	0.591
5	5137788	5718773	375	6.87E-06	9.38E-10	0.822
5	5719162	6858229	683	3.68E-06	1.59E-09	0.926
5	6859297	7852978	495	-3.61E-06	1.09E-09	0.913
5	7853304	9084076	627	3.43E-05	1.22E-09	0.327
5	9084701	10533767	857	1.18E-05	1.94E-09	0.788
5	10666804	11170506	324	-2.92E-05	6.52E-10	0.253
5	11170703	13603734	759	2.42E-05	1.32E-09	0.506
5	13603883	14987807	739	-3.74E-05	1.40E-09	0.318
5	14988284	16034608	301	-1.69E-05	4.01E-10	0.399
5	16035369	17161451	494	-4.00E-05	1.19E-09	0.245
5	17162704	18048683	259	9.14E-06	9.30E-10	0.764
5	18050107	18791978	252	-8.73E-06	6.80E-10	0.738
5	18792568	19486153	258	5.24E-06	4.89E-10	0.812
5	19487359	20414139	285	1.53E-06	4.03E-10	0.939
5	20415799	21573200	239	6.90E-06	4.60E-10	0.748
5	21574347	23150924	573	-4.39E-07	8.34E-10	0.988
5	23153562	24794957	518	1.92E-06	9.31E-10	0.950
5	24796795	26183141	454	2.20E-05	9.60E-10	0.478
5	26185302	26849079	203	1.45E-05	4.65E-10	0.501
5	26849808	28303480	392	2.74E-05	7.58E-10	0.321
5	28310093	30129786	583	2.60E-05	1.03E-09	0.419
5	30130713	31748529	717	-2.08E-06	1.60E-09	0.959

5	31748611	33944217	987	9.19E-06	2.40E-09	0.851
5	33945758	35798682	686	2.74E-05	1.41E-09	0.466
5	35799275	37888797	710	-2.80E-05	1.34E-09	0.443
5	37889231	39438373	674	-1.32E-05	1.42E-09	0.727
5	39438575	40954879	626	-8.76E-06	9.44E-10	0.776
5	40955561	42620153	556	-9.93E-06	7.75E-10	0.721
5	42623245	43859513	307	2.71E-05	4.80E-10	0.215
5	43862567	45286358	281	-5.10E-05	4.95E-10	0.022
5	45286977	50161698	290	-4.52E-06	2.78E-10	0.786
5	50162186	52133670	654	-7.63E-05	1.15E-09	0.024
5	52134410	52661197	301	-2.03E-05	5.42E-10	0.384
5	52662483	53513531	324	1.07E-05	6.78E-10	0.681
5	53515888	55412347	790	-6.74E-05	1.31E-09	0.063
5	55413961	55932471	299	-2.68E-05	8.59E-10	0.361
5	55933701	57337744	583	-4.69E-05	1.02E-09	0.142
5	57338357	58834736	805	4.87E-06	1.56E-09	0.902
5	58834852	60880207	583	9.64E-06	8.47E-10	0.740
5	60883078	62531300	658	4.95E-05	1.05E-09	0.127
5	62531952	63837838	373	1.58E-05	6.06E-10	0.520
5	63840208	65083148	459	1.56E-05	7.00E-10	0.554
5	65083209	65909616	334	-1.50E-05	7.31E-10	0.578
5	65910316	67096208	520	-1.25E-05	9.94E-10	0.691
5	67096780	70682458	690	3.44E-05	1.64E-09	0.397
5	70683839	72420748	629	-8.35E-07	9.15E-10	0.978
5	72420784	73507955	557	-3.00E-05	1.20E-09	0.386
5	73508509	75240469	728	1.44E-05	1.30E-09	0.689
5	75242395	75740199	233	2.37E-06	5.05E-10	0.916
5	75743532	77203135	737	6.35E-05	1.96E-09	0.151
5	77203718	79068089	685	4.41E-06	1.28E-09	0.902
5	79068300	80480761	705	6.45E-08	1.57E-09	0.999
5	80481996	81712715	434	-4.39E-05	8.18E-10	0.125
5	81714078	82658571	362	1.14E-05	7.75E-10	0.681
5	82660901	84397685	586	-2.90E-05	1.42E-09	0.441
5	84399219	85315500	310	6.04E-06	4.66E-10	0.780
5	85316429	86490293	328	1.77E-05	6.40E-10	0.484
5	86492986	88065637	305	1.43E-05	4.26E-10	0.487
5	88069593	90500398	712	9.40E-06	1.30E-09	0.794

5	90504842	93602613	721	1.87E-05	1.14E-09	0.579
5	93607443	95822590	793	4.78E-05	1.51E-09	0.218
5	95822732	96867936	466	-5.46E-05	8.31E-10	0.058
5	96870861	97435808	143	-1.30E-05	2.62E-10	0.421
5	97436504	99258325	558	-2.35E-05	9.10E-10	0.437
5	99259751	101101103	483	-3.29E-06	6.98E-10	0.901
5	101101325	102681586	443	2.07E-06	5.60E-10	0.930
5	102686157	104542488	700	-8.10E-06	1.46E-09	0.832
5	104543131	105885913	312	9.23E-06	5.52E-10	0.694
5	105887333	106800282	343	-3.78E-05	7.16E-10	0.158
5	106800765	108633176	758	3.89E-05	1.62E-09	0.333
5	108633347	109231597	246	-3.13E-06	3.55E-10	0.868
5	109232816	110819753	510	-1.39E-05	8.93E-10	0.642
5	110821786	111962676	471	1.21E-05	1.03E-09	0.707
5	111963025	112396750	156	2.05E-06	2.32E-10	0.893
5	112397011	113603768	561	1.06E-05	9.85E-10	0.736
5	113604579	114448326	333	-6.50E-06	6.08E-10	0.792
5	114448597	115828291	681	-5.09E-05	1.37E-09	0.170
5	115829425	117003674	543	-6.61E-05	1.14E-09	0.051
5	117004531	117712427	268	-8.31E-06	5.31E-10	0.718
5	117714517	119668423	688	-2.48E-05	1.34E-09	0.497
5	119669457	120962383	495	1.30E-06	1.01E-09	0.967
5	120963248	121481183	148	6.70E-06	2.71E-10	0.684
5	121485609	122603725	451	-1.94E-05	8.16E-10	0.498
5	122604501	123236124	257	1.80E-05	4.61E-10	0.403
5	123238611	123797013	229	-7.72E-06	4.90E-10	0.727
5	123798436	124746813	465	-2.35E-05	1.16E-09	0.491
5	124748624	126938920	874	7.58E-06	1.72E-09	0.855
5	126940058	128727903	666	-1.39E-05	1.11E-09	0.676
5	128730275	130376511	412	-5.84E-06	5.02E-10	0.794
5	130376936	131873073	450	4.98E-06	5.08E-10	0.825
5	131874022	132603137	224	1.17E-05	3.72E-10	0.544
5	132609517	134321546	718	-6.86E-05	1.58E-09	0.084
5	134329463	135339464	501	-1.58E-05	9.64E-10	0.611
5	135340951	137948424	905	4.83E-05	1.34E-09	0.187
5	137950183	140644484	766	-7.21E-06	9.43E-10	0.814
5	140644957	141640050	475	-1.31E-05	1.08E-09	0.691

5	141640419	142230251	276	-2.02E-05	7.10E-10	0.449
5	144584265	145803004	431	2.63E-06	6.04E-10	0.915
5	142230949	144583138	963	1.66E-05	1.61E-09	0.679
5	145803849	147651230	762	-7.78E-05	1.46E-09	0.042
5	150395351	150917796	368	-3.81E-05	7.31E-10	0.159
5	147665223	150393107	1253	9.56E-06	2.29E-09	0.842
5	150918158	152360494	594	8.48E-05	1.38E-09	0.022
5	152361546	152864614	177	5.04E-06	2.90E-10	0.767
5	152867348	153494463	316	-5.40E-06	4.40E-10	0.797
5	153495193	154448863	413	-1.90E-06	8.56E-10	0.948
5	154449856	155290211	358	-3.24E-06	6.42E-10	0.898
5	155290613	156628287	483	-1.99E-06	9.11E-10	0.947
5	156629754	157197141	306	-5.09E-05	5.41E-10	0.029
5	157199547	158491655	551	-6.14E-06	9.58E-10	0.843
5	158492279	159135376	279	5.50E-05	6.13E-10	0.026
5	159136348	160908680	774	-1.41E-06	1.62E-09	0.972
5	160910101	161688620	224	-4.27E-07	3.99E-10	0.983
5	161688913	162741298	358	8.44E-06	6.75E-10	0.745
5	162742614	163769136	431	5.89E-06	8.85E-10	0.843
5	163769738	164944949	399	-1.02E-05	7.52E-10	0.710
5	164947504	165638940	255	-2.33E-06	7.66E-10	0.933
5	165642395	166432731	347	6.92E-06	8.92E-10	0.817
5	166433367	167760730	549	-6.57E-06	1.27E-09	0.854
5	167761922	169124478	717	-2.89E-05	1.71E-09	0.486
5	169125063	170889081	954	6.57E-05	1.99E-09	0.141
5	170891834	171772889	429	-1.25E-05	1.02E-09	0.696
5	171775715	172376903	328	2.29E-05	8.92E-10	0.444
5	172377031	173611384	635	-5.50E-05	1.51E-09	0.157
5	173613085	174468408	481	7.37E-05	1.88E-09	0.089
5	174468624	176128648	690	1.00E-05	1.75E-09	0.811
5	176129392	177991305	618	1.52E-05	1.45E-09	0.691
5	177992368	178410668	199	7.14E-05	5.26E-10	0.002
5	178410839	179245749	391	-7.28E-05	9.70E-10	0.019
5	179245856	180007693	316	4.71E-05	9.08E-10	0.118
5	180008456	180790950	242	1.37E-06	7.10E-10	0.959
6	63979	824529	335	-2.68E-05	1.09E-09	0.417
6	826312	1582647	523	-1.11E-06	1.73E-09	0.979

6	1582926	2890836	718	3.02E-05	1.89E-09	0.488
6	2891016	4315210	772	-7.10E-05	2.15E-09	0.125
6	4317398	5056146	426	3.69E-05	1.05E-09	0.254
6	5057399	6246639	735	-1.40E-06	1.95E-09	0.975
6	6246984	7328580	685	2.51E-05	2.27E-09	0.598
6	7329417	8791321	741	-2.11E-05	2.40E-09	0.666
6	8791421	9760323	374	2.29E-06	9.59E-10	0.941
6	9764413	11234164	726	-3.55E-05	2.16E-09	0.445
6	11235682	12183952	544	1.62E-05	1.29E-09	0.652
6	12186102	13388480	617	2.08E-05	1.39E-09	0.578
6	13389521	14618107	554	4.66E-05	1.35E-09	0.205
6	14619148	15998368	596	-4.89E-05	2.67E-09	0.344
6	15998406	17067160	545	-4.56E-05	1.73E-09	0.273
6	17069966	19203108	880	-1.16E-05	1.95E-09	0.792
6	19203782	20228741	524	-1.28E-05	1.52E-09	0.742
6	20231516	22049758	824	1.40E-05	2.63E-09	0.784
6	22052425	23495209	748	-4.30E-06	3.00E-09	0.937
6	23495260	23935020	233	6.28E-06	5.45E-10	0.788
6	23935352	24799440	438	4.01E-06	1.28E-09	0.911
6	24803038	25483400	411	1.79E-05	1.02E-09	0.576
6	25484295	26774948	730	8.13E-06	2.49E-09	0.870
6	26778827	28669698	609	-1.93E-05	1.39E-09	0.604
6	32900018	33863002	401	-5.70E-05	1.81E-09	0.181
6	33863662	35451187	514	7.71E-05	1.01E-09	0.015
6	35452346	36608267	395	1.01E-05	8.28E-10	0.725
6	38687185	39021712	205	-1.54E-07	4.54E-10	0.994
6	36611164	38686222	997	2.67E-05	2.48E-09	0.591
6	39022698	40344526	675	1.92E-05	1.59E-09	0.630
6	40345542	42105449	887	-4.60E-05	2.62E-09	0.369
6	42105555	43957473	633	-2.90E-05	1.68E-09	0.479
6	43959116	45787468	702	1.86E-05	1.75E-09	0.658
6	45788111	46556325	327	-3.52E-06	6.77E-10	0.892
6	46560003	47885216	681	-1.21E-05	1.37E-09	0.744
6	47887063	49494241	468	-1.01E-06	8.19E-10	0.972
6	49494933	50406476	309	-1.43E-05	6.74E-10	0.581
6	50406575	51703012	475	2.87E-06	9.83E-10	0.927
6	51704668	53402679	834	1.10E-05	1.98E-09	0.804

6	53405127	54291407	454	-2.04E-06	9.10E-10	0.946
6	54293261	56103747	744	-1.01E-05	1.48E-09	0.793
6	56104522	57082026	352	-7.02E-06	6.48E-10	0.783
6	57085542	58084365	151	-2.38E-06	3.11E-10	0.892
6	58139505	62691733	306	2.91E-06	5.33E-10	0.900
6	62692409	63712779	264	3.16E-06	4.18E-10	0.877
6	63713620	65760585	508	-1.08E-05	8.16E-10	0.706
6	65762553	67028107	493	-1.58E-05	9.84E-10	0.614
6	67029501	67556729	242	-4.61E-07	5.61E-10	0.984
6	67557245	68546601	376	3.56E-05	8.21E-10	0.214
6	68546995	69295414	301	-1.51E-06	8.12E-10	0.958
6	69295526	70359936	399	-1.79E-05	7.80E-10	0.522
6	70360869	72448598	882	2.85E-05	1.85E-09	0.508
6	72449421	73170712	296	5.75E-06	6.67E-10	0.824
6	73171594	75064563	683	5.84E-05	1.46E-09	0.127
6	75065279	75843360	172	-3.44E-06	3.95E-10	0.863
6	75843845	77257094	415	-2.06E-05	7.15E-10	0.442
6	77257275	78957547	680	1.27E-05	1.30E-09	0.724
6	78958134	79861169	296	-6.15E-06	5.46E-10	0.793
6	79862483	80556359	340	1.43E-05	8.31E-10	0.619
6	80557170	82387384	660	5.59E-05	1.19E-09	0.105
6	82387920	83904869	519	-9.15E-06	1.11E-09	0.783
6	83908232	86040334	810	-8.15E-07	1.68E-09	0.984
6	86040497	87138115	340	-1.24E-05	6.25E-10	0.619
6	87140364	88532072	486	-7.55E-05	8.58E-10	0.010
6	88533324	89668547	467	3.90E-05	1.27E-09	0.274
6	89673861	91400644	815	5.64E-05	1.98E-09	0.205
6	91401234	93426186	759	-9.20E-06	1.82E-09	0.829
6	93429797	94919277	573	4.60E-05	1.30E-09	0.203
6	94920611	97093037	660	-2.67E-05	1.41E-09	0.478
6	97093295	98893182	514	-8.79E-07	1.04E-09	0.978
6	98893830	100286683	585	2.11E-05	1.32E-09	0.562
6	100287245	101862923	465	6.93E-06	9.28E-10	0.820
6	101863563	102887537	348	6.88E-06	8.45E-10	0.813
6	102888281	104211089	445	-2.28E-05	8.40E-10	0.431
6	104211475	105752435	549	-1.97E-05	1.15E-09	0.560
6	105754306	106921804	537	-1.59E-05	1.58E-09	0.690

6	106922281	108322638	656	-1.03E-05	1.74E-09	0.805
6	108325624	110654066	761	-3.62E-05	1.40E-09	0.332
6	110654605	111390344	274	5.45E-06	6.64E-10	0.833
6	114220361	116130955	594	-7.86E-06	1.25E-09	0.824
6	111391537	114218733	1067	2.06E-05	2.27E-09	0.666
6	116130983	117566682	496	-7.52E-05	1.25E-09	0.034
6	117567468	119071713	561	2.41E-06	1.18E-09	0.944
6	119075856	120628075	540	1.48E-05	1.20E-09	0.668
6	120628214	121673627	339	1.49E-05	6.25E-10	0.552
6	121674337	123104703	522	-1.64E-05	1.04E-09	0.610
6	123105676	123889346	320	3.38E-05	8.16E-10	0.236
6	123890282	125333797	658	-2.05E-05	1.72E-09	0.621
6	125336799	128759251	900	-1.54E-05	1.84E-09	0.719
6	128759755	130538497	787	-4.18E-06	1.94E-09	0.924
6	130540383	131972731	564	3.94E-05	1.26E-09	0.268
6	131973932	133867802	873	2.94E-05	2.20E-09	0.531
6	133869809	136186007	802	-1.14E-05	1.95E-09	0.796
6	136186368	137811295	508	7.97E-05	1.13E-09	0.018
6	137813095	139532424	748	2.50E-05	2.03E-09	0.579
6	139533572	142036032	709	3.74E-06	1.54E-09	0.924
6	142036912	143719278	602	-1.85E-06	1.32E-09	0.959
6	143720467	146663272	1047	1.70E-05	2.20E-09	0.718
6	146665424	148179283	592	-3.38E-05	1.29E-09	0.347
6	148180191	148917941	479	-3.19E-05	1.59E-09	0.423
6	148919158	150298842	639	5.88E-06	1.28E-09	0.870
6	150299299	151097980	538	7.85E-06	1.67E-09	0.847
6	151100484	151929942	491	1.93E-05	1.50E-09	0.619
6	151930249	153550147	880	-4.25E-05	2.80E-09	0.422
6	153551177	155228511	745	-1.08E-05	2.00E-09	0.810
6	155229566	156756457	768	6.89E-06	2.13E-09	0.881
6	156761338	157398853	256	4.47E-05	6.35E-10	0.076
6	157402041	158192951	273	-1.57E-05	7.96E-10	0.579
6	158193244	159318774	547	-2.64E-05	1.10E-09	0.426
6	159319812	159792458	251	-1.88E-05	4.46E-10	0.374
6	159793334	161123451	690	2.12E-05	7.00E-09	0.800
6	161123771	162298351	679	-1.70E-05	4.19E-09	0.793
6	162300151	163277414	510	-5.00E-07	1.20E-09	0.988

6	163277858	164003180	378	5.03E-05	1.00E-09	0.112
6	164006012	165242243	674	-1.90E-05	1.67E-09	0.642
6	165246062	166191910	572	1.17E-05	1.56E-09	0.767
6	166193932	167177733	678	3.08E-05	1.90E-09	0.480
6	167178790	168548525	628	6.44E-06	1.45E-09	0.866
6	168549335	169231503	390	-2.25E-05	1.06E-09	0.490
6	169231937	171049558	874	8.00E-06	1.98E-09	0.857
7	20608	1490775	419	3.74E-05	1.24E-09	0.289
7	1493090	2479029	317	1.20E-06	7.48E-10	0.965
7	2480729	2930941	207	1.61E-05	5.01E-10	0.472
7	2931676	3736942	499	-3.98E-06	1.22E-09	0.910
7	3738087	4783224	512	1.38E-07	1.26E-09	0.997
7	4783723	5412932	213	7.57E-06	7.06E-10	0.776
7	5414602	6912527	401	2.44E-05	1.04E-09	0.450
7	7168493	7679885	293	-3.11E-05	7.00E-10	0.239
7	7680697	8475147	543	5.92E-05	1.61E-09	0.140
7	8476787	9223721	526	-1.79E-05	1.36E-09	0.627
7	9223762	9893744	341	-2.06E-05	9.41E-10	0.502
7	9894421	11298815	623	-6.94E-06	1.38E-09	0.852
7	11299552	12687871	880	-4.28E-05	2.19E-09	0.360
7	12689322	13674908	508	-3.39E-07	1.22E-09	0.992
7	13675094	14941781	684	3.39E-05	1.75E-09	0.417
7	14942134	16480989	759	1.33E-05	1.70E-09	0.747
7	16481863	17277084	484	-5.64E-06	1.47E-09	0.883
7	17279294	18861958	710	5.79E-06	1.69E-09	0.888
7	18863387	19975992	510	2.30E-05	1.09E-09	0.486
7	19978609	21712233	880	-5.74E-05	3.36E-09	0.322
7	21712615	22811384	708	4.77E-05	1.96E-09	0.281
7	22811921	24389035	702	-2.75E-05	1.36E-09	0.456
7	24389147	24980408	323	-2.09E-06	8.15E-10	0.942
7	24982494	26300992	674	-3.65E-05	2.77E-09	0.487
7	26302668	28038375	768	7.19E-05	8.90E-09	0.446
7	28039590	28710596	382	-2.65E-05	1.46E-09	0.487
7	28710857	30171413	827	3.65E-05	2.02E-09	0.417
7	30172195	31608495	659	4.35E-06	1.60E-09	0.913
7	31609317	33150311	750	-9.72E-06	1.60E-09	0.808
7	33151181	34939884	766	1.95E-05	1.67E-09	0.633

7	34940039	35614036	301	-7.69E-06	6.28E-10	0.759
7	35615611	37353302	858	5.72E-05	2.03E-09	0.204
7	37354091	38159855	466	-7.22E-06	1.08E-09	0.826
7	38160558	39534540	677	8.46E-06	1.62E-09	0.833
7	39535570	40925323	373	-2.91E-05	7.23E-10	0.279
7	40926906	42494824	654	9.05E-06	1.56E-09	0.819
7	46667873	47279128	336	-2.30E-06	7.29E-10	0.932
7	42495555	44971837	906	1.87E-05	1.67E-09	0.647
7	44972152	46665658	694	-3.61E-05	1.47E-09	0.346
7	47281095	49130572	861	6.05E-05	2.15E-09	0.192
7	49131824	49821750	300	3.31E-05	6.07E-10	0.179
7	49822437	51674880	774	-7.22E-06	1.51E-09	0.853
7	51675218	52274523	293	-8.46E-06	7.75E-10	0.761
7	52275656	53175065	478	2.91E-06	1.05E-09	0.928
7	53348777	54707110	519	3.72E-06	1.00E-09	0.906
7	54707337	55641707	451	1.30E-06	1.04E-09	0.968
7	55641798	57382254	387	-2.89E-06	7.77E-10	0.917
7	57382271	62556935	175	-1.19E-05	2.70E-10	0.467
7	62557202	64193303	281	1.92E-06	4.97E-10	0.931
7	64699988	65570171	153	-2.50E-06	2.14E-10	0.865
7	65570310	66453460	219	4.88E-06	2.15E-10	0.739
7	66454071	67369810	339	-1.00E-05	7.56E-10	0.716
7	67370861	68125403	317	1.33E-05	8.56E-10	0.649
7	68126378	68561421	188	1.24E-05	4.20E-10	0.544
7	68562932	69806895	289	-6.77E-05	4.55E-10	0.002
7	69808153	71204524	594	3.41E-06	1.69E-09	0.934
7	71205507	71873571	249	9.61E-07	4.84E-10	0.965
7	71874997	73996533	397	1.54E-06	7.71E-10	0.956
7	73997700	76550299	364	-6.67E-05	8.41E-10	0.021
7	76551983	77101532	181	3.50E-06	4.13E-10	0.863
7	77102731	77953248	411	3.97E-06	8.33E-10	0.891
7	77954163	78839317	553	-1.79E-05	1.37E-09	0.628
7	78843289	80546745	614	1.44E-05	1.22E-09	0.681
7	80547047	82758529	882	-1.09E-05	2.21E-09	0.816
7	82758644	83959105	533	2.21E-05	1.14E-09	0.513
7	83959751	85570789	448	-2.97E-05	7.52E-10	0.278
7	85571372	86341446	218	-7.72E-06	3.77E-10	0.691

7	88732470	89270556	217	-1.32E-05	4.48E-10	0.533
7	86345013	88732142	836	1.67E-05	1.45E-09	0.662
7	89271899	91173749	774	-3.95E-05	1.55E-09	0.316
7	91174778	93339775	617	2.45E-07	1.06E-09	0.994
7	93340137	95171731	630	1.39E-05	1.41E-09	0.711
7	95419256	96611864	460	-3.92E-05	9.03E-10	0.192
**7**	**96613246**	**98151455**	**496**	**-4.79E-04**	**8.58E-09**	**0.000**
7	98152191	100555361	666	2.40E-05	1.25E-09	0.498
7	100606141	101730309	387	3.30E-05	1.30E-09	0.360
7	101731567	103193331	319	5.56E-06	6.32E-10	0.825
7	103193651	104157576	550	-3.89E-06	1.22E-09	0.911
7	104158491	105425027	481	-3.67E-05	8.44E-10	0.206
7	105427565	106052506	320	1.57E-07	9.15E-10	0.996
7	106052873	107305328	457	2.51E-05	9.66E-10	0.420
7	107306421	108375939	543	-2.26E-05	1.04E-09	0.484
7	108376173	109584769	351	4.01E-06	6.70E-10	0.877
7	109587777	111447153	593	-2.31E-05	1.12E-09	0.490
7	111450919	113336970	590	-6.47E-06	1.29E-09	0.857
7	113337327	116315205	824	-8.76E-05	1.57E-09	0.027
7	116317043	118347634	570	3.03E-05	9.56E-10	0.328
7	118348359	121094386	573	5.50E-06	9.05E-10	0.855
7	121094460	122368663	413	-1.54E-05	9.06E-10	0.609
7	122369853	124154451	639	4.94E-06	1.09E-09	0.881
7	124155319	125386718	379	2.31E-05	5.90E-10	0.342
7	125387168	126516683	442	-1.02E-05	7.94E-10	0.717
7	126517818	127905983	588	-3.90E-05	9.83E-10	0.214
7	127907054	128554075	209	-3.40E-06	4.74E-10	0.876
7	128555262	130074230	546	-2.39E-05	1.14E-09	0.478
7	130075370	130797123	249	-1.25E-05	7.15E-10	0.641
7	130797776	131856855	498	-1.75E-05	1.30E-09	0.628
7	131857419	134306672	895	8.06E-05	1.65E-09	0.048
7	134307114	135464701	504	1.85E-05	1.07E-09	0.572
7	135466022	137137511	701	1.15E-05	1.54E-09	0.770
7	137138736	138742981	587	-4.44E-05	1.72E-09	0.285
7	138744100	141106552	845	-4.81E-05	2.30E-09	0.316
7	141109797	142655113	482	2.25E-05	8.54E-10	0.440
7	142655717	144274735	430	-1.20E-05	8.40E-10	0.678

7	144276424	146018520	552	-7.03E-06	1.16E-09	0.837
7	146023023	147204086	503	1.38E-05	1.02E-09	0.666
7	147204549	148619685	685	1.04E-05	1.52E-09	0.791
7	148620581	150491084	657	1.77E-05	1.39E-09	0.635
7	150491851	151390799	461	-2.23E-05	1.27E-09	0.532
7	151391044	152409155	288	3.62E-05	1.26E-09	0.308
7	152410248	153080056	288	-8.68E-05	9.13E-10	0.004
7	153081698	153934166	322	6.78E-06	8.63E-10	0.818
7	153934210	154963787	621	-7.72E-06	1.61E-09	0.847
7	154964316	155379409	239	1.53E-06	1.01E-09	0.962
7	155380353	156814410	651	1.68E-06	1.74E-09	0.968
7	156815867	158029340	505	4.79E-05	1.32E-09	0.188
7	158030463	158759939	286	4.77E-06	5.53E-10	0.839
7	158760677	159127587	134	-4.79E-06	3.04E-10	0.783
8	246934	1162157	489	1.51E-05	1.53E-09	0.698
8	1162454	1625424	273	-6.25E-06	8.71E-10	0.832
8	1625732	2042364	303	1.10E-05	1.14E-09	0.745
8	2043187	2680255	317	1.39E-05	1.12E-09	0.678
8	2681051	3358423	619	-6.93E-06	2.00E-09	0.877
8	3358433	3584832	297	-2.30E-06	8.64E-10	0.938
8	3585058	4071438	630	-2.62E-05	1.77E-09	0.533
8	4071516	4274957	255	7.15E-06	7.44E-10	0.793
8	4275286	4479596	220	1.44E-05	8.73E-10	0.626
8	4479836	4754328	302	-2.01E-06	8.30E-10	0.944
8	4755088	5146808	311	-1.52E-05	8.97E-10	0.611
8	5147734	5781592	596	2.00E-05	1.67E-09	0.624
8	5781947	6017670	226	-7.85E-06	5.61E-10	0.740
8	6018292	6350873	272	-3.66E-07	6.69E-10	0.989
8	6352358	7793703	490	-2.86E-05	1.46E-09	0.455
8	7793837	8735213	412	1.34E-05	1.01E-09	0.673
8	8736386	10117293	800	-1.16E-05	2.14E-09	0.803
8	10117411	10535826	286	-1.06E-05	8.91E-10	0.724
8	10537940	11249261	410	-1.92E-05	9.16E-10	0.525
8	11250848	11836622	364	-1.85E-05	1.19E-09	0.591
8	11838355	12975295	284	-3.65E-05	8.19E-10	0.202
8	12975578	13940652	686	3.53E-05	1.82E-09	0.408
8	13942500	14876660	523	-2.71E-05	1.27E-09	0.446

8	14877288	15273777	321	-9.11E-06	7.30E-10	0.736
8	15274608	16087631	534	-7.77E-06	1.44E-09	0.838
8	16087729	17579518	803	-2.46E-05	2.25E-09	0.604
8	17581106	18670645	722	-4.84E-05	1.77E-09	0.250
8	18670908	19490180	559	-2.96E-05	1.56E-09	0.453
8	19491428	21021401	887	5.73E-05	2.56E-09	0.257
8	21022075	21402658	257	-2.57E-06	6.58E-10	0.920
8	21404381	22539316	482	-2.89E-07	1.08E-09	0.993
8	22539641	23548146	588	-8.04E-05	5.60E-09	0.282
8	23548749	24456913	317	-1.26E-06	7.46E-10	0.963
8	24457122	25461315	425	3.66E-05	8.68E-10	0.214
8	25461344	26681450	716	-6.28E-05	2.75E-09	0.231
8	26681799	27404354	420	-1.33E-05	9.37E-10	0.663
8	27405207	27759126	214	-2.54E-06	4.25E-10	0.902
8	27759839	29277590	622	-4.76E-07	1.46E-09	0.990
8	31947880	32448740	282	-1.08E-05	7.12E-10	0.685
8	29277652	31947675	956	6.56E-05	2.41E-09	0.181
8	32449533	33980486	355	1.45E-05	6.74E-10	0.578
8	33981742	35313077	399	1.78E-05	6.56E-10	0.488
8	35314837	37375458	435	-4.72E-06	8.86E-10	0.874
8	37376914	39779989	596	-2.25E-05	1.45E-09	0.554
8	39781444	40415799	340	2.36E-05	8.69E-10	0.423
8	40417433	41718356	592	1.24E-05	1.54E-09	0.751
8	41719750	42501174	186	8.45E-06	3.78E-10	0.664
8	42501717	49028667	390	3.27E-07	5.52E-10	0.989
8	49038732	52156842	816	2.05E-05	1.36E-09	0.578
8	52157415	54115712	718	-1.59E-05	1.49E-09	0.680
8	54116775	55914038	660	-1.28E-06	1.79E-09	0.976
8	55914314	57653028	655	1.52E-05	1.52E-09	0.696
8	57655067	58674153	403	1.63E-05	8.64E-10	0.580
8	58675131	59568410	383	-3.63E-06	9.50E-10	0.906
8	59570176	61809929	767	-6.53E-05	1.70E-09	0.113
8	61810694	63710298	771	3.50E-06	1.70E-09	0.932
8	63711348	65193342	460	-2.44E-06	9.99E-10	0.938
8	65195953	66024947	260	-1.05E-05	5.50E-10	0.653
8	66025862	67192642	387	-2.86E-05	8.03E-10	0.313
8	67193708	69108839	501	-2.86E-05	1.17E-09	0.403

8	69111244	70003448	401	1.96E-05	1.10E-09	0.554
8	70003607	72013005	727	1.34E-05	1.70E-09	0.746
8	72014069	72749564	427	1.17E-05	1.25E-09	0.740
8	72751469	73423214	352	-1.27E-05	9.26E-10	0.677
8	73423786	74996061	687	1.34E-07	1.62E-09	0.997
8	74998868	76294909	508	3.07E-06	1.18E-09	0.929
8	76295292	78015335	544	-1.89E-05	1.14E-09	0.575
8	78015611	78632215	197	1.20E-05	3.90E-10	0.544
8	78632893	79831888	374	9.20E-06	6.83E-10	0.725
8	79832211	80654655	273	-1.06E-05	7.35E-10	0.697
8	80656206	81330540	223	-8.64E-06	5.75E-10	0.719
8	81331077	83726996	809	-1.38E-05	1.89E-09	0.751
8	83727741	85093121	385	-1.37E-05	8.84E-10	0.644
8	85094979	86979097	352	1.18E-05	6.11E-10	0.634
8	86990451	87562786	212	1.80E-05	3.76E-10	0.354
8	87563878	89083319	540	1.50E-05	1.18E-09	0.662
8	89083979	90605445	456	-7.47E-06	8.74E-10	0.801
8	90607207	92342283	523	2.89E-06	9.26E-10	0.924
8	92344138	94269630	544	4.68E-06	1.32E-09	0.898
8	94272436	95810190	578	-1.16E-05	1.29E-09	0.747
8	95810772	96533604	390	2.50E-05	9.95E-10	0.428
8	96534326	97318660	396	-1.39E-05	1.05E-09	0.666
8	97320605	98867870	622	6.48E-06	1.40E-09	0.862
8	98869940	101450242	830	-5.46E-06	1.53E-09	0.889
8	101451169	102937063	656	-3.28E-05	1.73E-09	0.431
8	102944509	103730879	375	-1.37E-06	1.10E-09	0.967
8	103970185	105389708	566	-1.02E-05	1.15E-09	0.763
8	105391381	107409808	782	1.36E-05	1.84E-09	0.752
8	107410067	108108114	325	1.49E-05	7.05E-10	0.575
8	108115161	108646193	244	4.82E-06	6.42E-10	0.849
8	108646968	110761074	744	-2.04E-05	1.39E-09	0.584
8	110761776	112611637	402	1.83E-05	7.73E-10	0.510
8	112612842	114393054	481	-9.85E-06	8.09E-10	0.729
8	114394256	116095059	617	9.54E-07	1.19E-09	0.978
8	116095815	117130004	271	1.99E-05	5.50E-10	0.395
8	117131947	118622352	563	2.68E-05	1.36E-09	0.469
8	118623844	120216957	719	2.12E-05	1.77E-09	0.614

8	120217356	121767384	601	-1.48E-05	1.19E-09	0.668
8	121770585	123545992	691	1.85E-05	1.76E-09	0.659
8	123547148	124483938	403	8.00E-06	1.12E-09	0.811
8	124486753	125247268	404	6.43E-06	1.11E-09	0.847
8	125248576	126268046	461	2.60E-06	1.23E-09	0.941
8	126269921	127434945	507	-4.43E-06	1.35E-09	0.904
8	127436003	128162974	338	-5.29E-05	1.50E-08	0.666
8	128166556	128542444	193	-3.15E-04	1.83E-08	0.020
8	128543956	129297518	432	-3.83E-05	1.90E-09	0.379
8	129297807	130379330	411	1.84E-05	9.21E-10	0.543
8	130380171	130812339	231	-1.60E-05	5.89E-10	0.510
8	130814803	131553214	285	-2.40E-05	6.30E-10	0.340
8	131553327	132358990	430	1.54E-05	1.08E-09	0.639
8	132359241	133454722	553	-1.13E-05	1.25E-09	0.750
8	133455530	134566686	734	-2.55E-05	1.78E-09	0.546
8	134567348	135269827	424	6.31E-07	1.25E-09	0.986
8	135270868	136209239	515	1.73E-05	1.32E-09	0.633
8	136209325	137306730	483	3.25E-06	1.02E-09	0.919
8	137307361	138223632	347	-6.58E-06	7.39E-10	0.809
8	138224181	139194661	572	3.34E-05	1.31E-09	0.356
8	139195331	140093896	520	4.28E-05	1.51E-09	0.271
8	140094627	141355502	731	-1.10E-05	1.87E-09	0.800
8	141358681	143750205	924	-1.71E-05	2.38E-09	0.725
8	143750619	146303789	716	-1.15E-06	1.51E-09	0.976
9	342841	1006606	533	2.63E-05	1.39E-09	0.480
9	1007923	1792147	678	2.06E-05	1.65E-09	0.612
9	1792277	2665240	685	-4.02E-05	1.99E-09	0.368
9	2666964	3348816	358	-2.96E-05	8.45E-10	0.309
9	3349487	4378727	699	1.86E-05	2.67E-09	0.719
9	4380096	4590305	142	2.37E-05	4.24E-10	0.250
9	4591655	5273194	342	4.68E-05	7.45E-10	0.086
9	5273896	6144065	384	-1.79E-05	8.25E-10	0.533
9	6144333	7232230	569	4.00E-06	1.35E-09	0.913
9	7232510	7757891	465	4.40E-06	1.19E-09	0.898
9	7758579	8235898	325	6.59E-06	8.03E-10	0.816
9	8236122	9374664	803	4.57E-05	2.24E-09	0.335
9	9375194	9793342	253	-1.33E-05	6.32E-10	0.596

9	9793846	10358286	350	6.21E-06	8.70E-10	0.833
9	10358672	10879071	263	2.51E-05	7.27E-10	0.352
9	10879188	11616822	307	-1.59E-06	6.47E-10	0.950
9	11884425	12274220	180	-7.52E-06	3.96E-10	0.705
9	12274774	12778224	274	-2.91E-05	6.40E-10	0.251
9	12780295	13797055	477	-2.00E-05	1.22E-09	0.566
9	13797496	14712257	604	-8.97E-06	2.09E-09	0.844
9	14714681	16054406	644	-3.52E-05	1.45E-09	0.354
9	16056127	16833797	538	2.33E-05	1.45E-09	0.540
9	16836011	17627988	363	1.07E-05	7.53E-10	0.696
9	17628726	18659920	605	-6.86E-06	1.40E-09	0.854
9	18660695	19129349	353	3.09E-06	9.41E-10	0.920
9	19456098	20324812	482	-2.04E-05	1.20E-09	0.556
9	20324848	21549638	502	1.46E-05	1.05E-09	0.653
9	21550959	22968575	596	1.37E-05	1.32E-09	0.706
9	22968789	24154728	439	2.19E-05	1.31E-09	0.545
9	24155142	25425160	409	3.83E-05	1.36E-09	0.298
9	25425511	26317104	404	1.63E-05	1.21E-09	0.639
9	26317973	27335346	535	-1.93E-05	1.35E-09	0.598
9	27336207	28252383	497	-7.75E-06	1.38E-09	0.834
9	28253887	29622939	660	-7.70E-06	1.38E-09	0.836
9	29623053	30819576	414	-8.82E-06	7.16E-10	0.742
9	30821676	31836697	373	-1.99E-05	7.34E-10	0.463
9	31837040	32249649	172	1.41E-07	3.24E-10	0.994
9	32250247	33186788	464	-2.99E-05	9.72E-10	0.338
9	33187897	34769004	522	-2.61E-05	1.07E-09	0.424
9	34771534	36712124	646	-7.88E-06	1.34E-09	0.829
9	36714252	37678932	456	6.02E-05	1.10E-09	0.070
9	37679713	42220384	545	3.16E-05	1.41E-09	0.400
9	69576527	71968825	445	9.97E-06	9.95E-10	0.752
9	71969482	73398448	622	-3.33E-05	1.16E-09	0.329
9	73399750	73916072	309	-1.35E-05	6.03E-10	0.583
9	73916583	74600739	231	1.47E-05	5.71E-10	0.539
9	74600822	75517582	356	-2.75E-05	6.62E-10	0.285
9	75519251	76785168	379	-2.26E-05	7.78E-10	0.417
9	76786297	78214833	562	-2.65E-05	1.30E-09	0.462
9	78215299	78900183	469	-1.43E-05	1.62E-09	0.722

9	81066078	81860861	384	-1.25E-05	9.29E-10	0.681
9	81863722	82348771	246	1.47E-05	5.35E-10	0.525
9	78901112	81064897	968	-4.23E-05	2.27E-09	0.375
9	82349606	83041982	353	1.43E-05	9.01E-10	0.633
9	83043376	83455568	219	9.83E-06	4.84E-10	0.655
9	83455867	84388566	421	1.83E-05	1.03E-09	0.569
9	84389168	86180165	683	-1.38E-06	1.60E-09	0.973
9	86181461	87571563	629	-2.46E-05	1.40E-09	0.511
9	87571959	87811640	150	-7.14E-06	3.19E-10	0.690
9	87813612	88867151	355	1.46E-05	7.25E-10	0.588
9	88869630	89948499	515	-2.65E-05	1.18E-09	0.441
9	89949778	90198251	198	-3.70E-05	3.94E-10	0.062
9	90198375	90893610	338	4.99E-06	8.11E-10	0.861
9	90893774	91608628	247	-1.24E-05	5.69E-10	0.604
9	91610543	92342927	327	9.01E-06	8.11E-10	0.752
9	92443570	93441674	461	-2.55E-05	1.08E-09	0.437
9	93442205	94163515	361	3.24E-05	9.17E-10	0.284
9	94164373	95854872	626	2.68E-05	1.10E-09	0.418
9	95855100	97719592	731	9.70E-06	1.41E-09	0.796
9	97720818	98889276	456	5.23E-06	9.05E-10	0.862
9	98891743	100917602	625	-5.36E-06	1.14E-09	0.874
9	100919318	101473404	376	-1.90E-05	9.75E-10	0.542
9	103544757	103969076	179	3.25E-06	2.73E-10	0.844
9	101474837	103543692	679	-9.12E-05	1.18E-09	0.008
9	103969339	104552711	293	1.60E-05	6.50E-10	0.531
9	104553938	105041375	303	1.50E-05	6.92E-10	0.568
9	105041756	105743445	320	-1.75E-05	6.39E-10	0.489
9	105744075	106487777	333	-2.41E-05	7.41E-10	0.376
9	106488291	107545903	496	-1.45E-06	7.86E-10	0.959
9	107546901	108751162	555	-4.34E-06	1.22E-09	0.901
9	108754828	109972524	499	-3.11E-05	1.18E-09	0.364
9	109974206	111137001	674	3.50E-05	1.67E-09	0.392
9	111138232	111997222	549	1.26E-05	1.12E-09	0.708
9	112001473	112593531	326	-2.91E-05	7.11E-10	0.275
9	112594858	113255402	401	2.19E-05	1.08E-09	0.504
9	113255544	114077164	462	4.95E-05	1.07E-09	0.130
9	114078103	114828332	330	-7.23E-06	7.16E-10	0.787

9	114830330	115391053	230	1.96E-05	3.62E-10	0.303
9	115393806	116148630	399	-1.99E-05	9.31E-10	0.514
9	116149967	117306807	689	-9.02E-05	1.72E-09	0.030
9	117307880	118569429	682	-1.99E-05	1.46E-09	0.602
9	118570426	120030166	743	-3.96E-05	1.74E-09	0.342
9	120030269	121008844	499	7.53E-06	1.07E-09	0.818
9	121009165	121318886	151	-5.03E-06	3.23E-10	0.780
9	121319662	122258204	433	-1.86E-05	8.87E-10	0.532
9	122258577	124870612	937	5.61E-05	1.79E-09	0.185
9	124871322	125542861	336	-1.36E-05	8.19E-10	0.634
9	125545194	126926376	449	-3.37E-05	9.35E-10	0.270
9	126927204	128926989	624	3.48E-05	1.10E-09	0.294
9	128927088	129477470	292	-1.75E-05	8.50E-10	0.549
9	129477886	131429495	582	-2.74E-05	1.04E-09	0.395
9	131430602	132891863	507	4.19E-06	1.21E-09	0.904
9	132895131	133640777	305	4.28E-06	1.03E-09	0.894
9	133642918	134639655	432	-1.07E-05	9.57E-10	0.731
9	134640928	135401279	345	2.34E-06	9.39E-10	0.939
9	135401721	136417159	502	4.93E-05	4.35E-09	0.455
9	136429816	136979539	317	2.54E-05	1.01E-09	0.423
9	136979744	137928846	496	3.44E-05	1.62E-09	0.393
9	137929853	138817142	515	-4.60E-05	1.74E-09	0.269
9	138817178	141146684	725	2.13E-05	1.71E-09	0.606
10	60969	747115	154	-2.56E-08	3.65E-10	0.999
10	747772	1330771	280	-9.20E-06	7.02E-10	0.728
10	1331955	1609370	172	-1.55E-06	4.71E-10	0.943
10	1610839	2104472	389	-1.30E-06	9.97E-10	0.967
10	2106418	3143283	689	-2.37E-05	1.74E-09	0.570
10	3144251	3785605	442	2.09E-05	1.09E-09	0.526
10	3786476	5358542	956	1.99E-05	2.25E-09	0.674
10	5359010	6316910	615	4.74E-05	1.70E-09	0.250
10	6318458	7639684	883	5.58E-05	2.48E-09	0.263
10	9433090	10540465	440	4.93E-05	1.30E-09	0.171
10	7639932	9432277	1108	-2.55E-05	2.57E-09	0.615
10	10542217	12092029	828	-4.15E-05	2.09E-09	0.364
10	12092208	12798954	371	-8.16E-05	1.33E-09	0.025
10	12799286	13718384	597	-7.40E-06	1.55E-09	0.851

10	13719970	14411956	499	-1.75E-05	1.44E-09	0.644
10	14412084	15303527	489	-7.73E-06	1.42E-09	0.837
10	15303983	16443442	476	3.49E-05	9.46E-10	0.257
10	16444693	17591626	664	-1.78E-05	1.60E-09	0.657
10	17591919	19202731	694	-1.44E-05	1.45E-09	0.705
10	19203300	20131614	450	1.61E-05	8.93E-10	0.591
10	20132516	21243558	680	-1.71E-06	1.53E-09	0.965
10	21244087	23696890	631	-2.44E-05	1.42E-09	0.518
10	23698033	25414215	686	-9.11E-06	1.37E-09	0.805
10	25416598	26631987	555	-8.91E-06	1.07E-09	0.785
10	26633775	27667904	411	-3.29E-06	8.24E-10	0.909
10	27668052	28734655	508	-1.25E-05	1.01E-09	0.696
10	28735045	29174963	234	-1.58E-05	4.68E-10	0.466
10	29175101	29961838	535	-1.78E-05	1.39E-09	0.634
10	29962511	30653567	388	6.86E-05	9.81E-10	0.029
10	30654528	31479103	433	1.46E-05	9.50E-10	0.636
10	31479422	33656047	779	1.44E-05	1.34E-09	0.695
10	36020445	36884906	379	8.16E-06	8.85E-10	0.784
10	33656119	36017592	829	-5.57E-06	1.52E-09	0.886
10	36885921	37845308	313	8.69E-07	6.20E-10	0.972
10	38367233	43220539	218	4.03E-07	3.16E-10	0.982
10	43222372	43605902	162	-5.65E-07	4.26E-10	0.978
10	43606687	45247184	692	-1.97E-05	1.30E-09	0.584
10	45247418	46769302	372	5.58E-05	1.91E-09	0.202
10	47768664	49812917	361	-6.10E-06	8.36E-10	0.833
10	49816189	50839403	568	-2.19E-05	1.18E-09	0.524
10	50840736	52086621	209	-1.08E-04	5.86E-09	0.157
10	52086939	52707026	238	-9.58E-06	4.21E-10	0.641
10	52708822	53362540	343	-2.78E-05	7.81E-10	0.320
10	53362932	54413554	512	1.17E-05	1.14E-09	0.729
10	54413909	55334166	523	3.00E-05	1.17E-09	0.381
10	55334740	56204677	453	-9.85E-06	7.70E-10	0.723
10	56207098	56883966	279	-2.27E-05	1.25E-09	0.521
10	56885639	58806290	704	-2.08E-05	1.14E-09	0.538
10	59158617	59822195	312	-1.41E-05	5.33E-10	0.542
10	59822974	60375613	206	-1.21E-05	3.10E-10	0.493
10	60375857	62558737	972	-5.93E-05	1.97E-09	0.182

10	62558842	64263322	626	-1.94E-05	1.27E-09	0.586
10	64263402	65469133	435	1.73E-05	7.70E-10	0.533
10	65735028	66980708	395	-2.42E-05	7.27E-10	0.369
10	66980980	67545544	227	-8.45E-06	4.77E-10	0.699
10	67546331	68429563	514	-3.83E-06	1.11E-09	0.909
10	68430236	69893580	549	8.61E-06	9.70E-10	0.782
10	69896336	71052508	440	1.50E-05	9.29E-10	0.624
10	71052888	72479853	929	5.61E-05	2.73E-09	0.283
10	72480072	73612902	784	-5.19E-05	2.22E-09	0.271
10	73614288	75696752	550	3.81E-06	6.27E-10	0.879
10	75700056	78638957	888	5.42E-06	1.42E-09	0.886
10	78642246	79740262	594	-8.55E-06	1.29E-09	0.812
10	79742617	80874092	594	-2.54E-05	1.69E-09	0.537
10	80874523	81881954	307	4.81E-05	1.35E-09	0.190
10	81882370	82378624	279	1.90E-05	9.08E-10	0.527
10	82379403	82881996	243	-2.02E-05	6.11E-10	0.414
10	82883949	83597042	269	1.10E-05	5.28E-10	0.633
10	83597818	84383108	342	6.68E-06	6.44E-10	0.792
10	84383277	85424102	487	2.32E-05	8.73E-10	0.433
10	85424493	86459792	527	-2.75E-05	1.32E-09	0.448
10	86460169	87285511	356	6.37E-07	6.27E-10	0.980
10	87285907	88450872	497	5.95E-06	1.17E-09	0.862
10	88451869	89845801	371	-1.33E-05	7.97E-10	0.637
10	89846944	90734906	410	-4.03E-05	8.91E-10	0.178
10	90735832	91959588	592	1.68E-05	1.19E-09	0.627
10	91960142	92682235	320	1.58E-05	5.58E-10	0.505
10	92682369	94196248	469	1.23E-05	8.68E-10	0.676
10	94196690	95064828	355	7.17E-06	5.70E-10	0.764
10	95065655	95946938	409	1.38E-06	9.35E-10	0.964
10	95949432	97984754	853	-2.50E-05	1.37E-09	0.499
10	99554895	100240681	333	-2.15E-05	7.05E-10	0.418
10	97985690	99554180	665	-5.54E-05	1.32E-09	0.127
10	100241204	102089663	714	9.52E-06	1.20E-09	0.784
10	102090924	104207799	690	-1.56E-05	1.18E-09	0.650
10	104208761	106139081	683	9.24E-06	1.30E-09	0.798
10	106139294	107874146	733	1.25E-05	1.31E-09	0.731
10	107875182	109577686	742	2.09E-05	1.60E-09	0.601

10	109578567	110311094	318	-1.01E-05	5.85E-10	0.677
10	110311425	112559414	764	-3.89E-05	1.38E-09	0.295
10	112560012	113839866	516	-4.91E-06	8.99E-10	0.870
10	113845990	115698315	912	-4.41E-05	2.22E-09	0.349
10	117705146	118416795	358	2.50E-05	7.14E-10	0.349
10	115700005	117702645	680	3.66E-05	1.23E-09	0.298
10	118417453	119697663	621	2.80E-07	1.58E-09	0.994
10	119698700	120994671	529	-2.20E-05	1.24E-09	0.533
10	120997666	121874991	393	-7.42E-06	8.81E-10	0.803
10	121875531	122805770	507	-2.61E-05	1.86E-09	0.545
10	122807254	123853223	521	1.29E-04	2.69E-09	0.013
10	123855124	124894743	545	-4.72E-07	1.22E-09	0.989
10	124894909	125868860	560	5.71E-06	1.53E-09	0.884
10	125919795	126739513	451	4.67E-05	1.43E-09	0.216
10	126739798	127820978	517	1.23E-05	1.09E-09	0.711
10	127821105	128409952	405	-2.36E-05	1.08E-09	0.472
10	128410393	129482709	634	-2.26E-06	1.67E-09	0.956
10	129483173	130282731	491	3.44E-05	1.33E-09	0.346
10	130282817	131192968	537	-2.45E-05	1.63E-09	0.544
10	131195759	132251820	596	4.96E-05	1.45E-09	0.194
10	132252499	132964299	471	-5.56E-06	1.27E-09	0.876
10	132966372	133355925	285	9.41E-06	9.48E-10	0.760
10	133356417	134858046	638	3.19E-05	1.55E-09	0.419
10	134858720	135524335	243	-6.31E-06	4.92E-10	0.776
11	333347	1057948	321	1.32E-05	6.66E-10	0.608
11	1058583	1861912	309	-1.17E-06	8.38E-10	0.968
11	1862168	2422034	247	1.29E-04	6.33E-09	0.106
11	2423545	3660412	527	-3.68E-05	1.95E-09	0.404
11	3661873	4745931	519	4.58E-06	1.64E-09	0.910
11	4746391	5529152	655	-7.89E-05	1.29E-09	0.028
11	5532222	6010718	357	-8.51E-06	7.56E-10	0.757
11	6011761	6917273	555	-2.37E-05	1.25E-09	0.502
11	6917772	7715871	483	-2.81E-05	1.82E-09	0.509
11	7717884	9068716	668	5.40E-05	1.46E-09	0.158
11	9069046	10370675	440	-2.01E-05	8.32E-10	0.485
11	10371614	11361034	681	-1.42E-05	1.74E-09	0.733
11	11361458	12018942	492	-6.31E-08	1.35E-09	0.999

11	12019010	13133902	717	-3.82E-05	2.15E-09	0.410
11	13134600	14934686	720	3.28E-05	1.29E-09	0.362
11	14936943	16789155	642	-5.62E-05	1.10E-09	0.091
11	16790799	18788126	918	-3.03E-05	1.90E-09	0.486
11	18789820	20113591	792	-3.87E-05	2.10E-09	0.399
11	21773680	23111273	502	3.90E-06	9.95E-10	0.902
11	20115277	21773251	1017	-2.87E-05	2.69E-09	0.580
11	24750113	25106557	163	2.32E-06	3.31E-10	0.899
11	23112584	24749839	709	-4.07E-05	1.67E-09	0.319
11	25109191	26046095	403	-1.92E-05	7.73E-10	0.491
11	26046627	27018778	419	-1.97E-05	8.06E-10	0.487
11	27019873	28741185	545	1.39E-05	9.53E-10	0.652
11	28742220	30056918	404	3.16E-07	8.40E-10	0.991
11	30057440	31853968	577	1.36E-05	8.59E-10	0.644
11	31856654	33383448	567	-7.08E-05	1.19E-09	0.041
11	33384046	34321466	429	-1.44E-06	1.07E-09	0.965
11	34321876	35034307	445	-8.84E-05	1.41E-09	0.019
11	35036029	36117340	598	3.37E-05	1.50E-09	0.383
11	36118027	37838625	746	1.84E-06	1.73E-09	0.965
11	37839347	40357803	640	1.53E-05	1.20E-09	0.658
11	40359663	40716222	154	2.18E-05	3.16E-10	0.220
11	40716823	42243196	591	-6.57E-07	1.19E-09	0.985
11	42244050	42854352	257	-3.21E-06	4.80E-10	0.884
11	42854878	43952796	470	1.32E-05	9.74E-10	0.672
11	46252956	47893120	417	3.58E-07	6.38E-10	0.989
11	43953774	46245900	1084	3.47E-05	2.96E-09	0.523
11	48351331	49659131	215	-2.37E-07	3.00E-10	0.989
11	49660004	55116367	305	-4.33E-06	4.12E-10	0.831
11	55116451	56160712	429	2.51E-06	5.90E-10	0.918
11	56625775	58455947	714	-2.29E-05	1.10E-09	0.490
11	58457495	59615953	298	1.76E-05	4.31E-10	0.396
11	59620206	61870732	816	-2.79E-05	1.74E-09	0.503
11	61873755	63153602	405	-4.56E-07	8.14E-10	0.987
11	63154309	66835194	1068	1.89E-05	2.67E-09	0.715
11	66835372	68515239	414	-1.80E-05	1.11E-09	0.589
11	68521072	69508567	391	1.59E-04	1.52E-08	0.198
11	69509343	70613763	469	-2.44E-05	1.34E-09	0.505

11	70616465	71842303	364	1.20E-05	6.84E-10	0.648
11	71842976	74158869	681	-1.81E-05	1.36E-09	0.625
11	74159542	76431100	889	-2.86E-05	1.98E-09	0.520
11	76432156	77903706	543	4.43E-05	8.68E-10	0.133
11	79723675	80720242	465	-2.69E-05	1.26E-09	0.449
11	77904339	79723318	879	2.78E-05	1.84E-09	0.517
11	80721384	81653514	551	-1.04E-05	1.24E-09	0.768
11	81654617	82844918	481	-1.35E-05	9.97E-10	0.669
11	82847086	83575703	267	3.09E-06	4.37E-10	0.882
11	83576460	84208616	304	2.30E-05	6.58E-10	0.369
11	84212522	84618654	169	-2.71E-06	4.22E-10	0.895
11	84618788	86623913	833	-4.94E-06	1.68E-09	0.904
11	86625137	87429545	370	1.20E-05	6.92E-10	0.648
11	87431647	87827395	167	1.34E-05	4.27E-10	0.517
11	87827514	89208590	526	1.73E-05	1.15E-09	0.609
11	89441680	91115475	483	4.84E-06	8.01E-10	0.864
11	91118661	91676282	198	-1.07E-06	4.25E-10	0.958
11	91677374	92626428	367	-8.67E-06	6.03E-10	0.724
11	92627643	93270431	278	-8.88E-06	5.77E-10	0.711
11	93272186	94389331	421	1.48E-05	7.93E-10	0.600
11	94391845	95329108	415	-7.73E-06	1.10E-09	0.816
11	95332310	96848965	669	-5.66E-05	1.50E-09	0.144
11	96849530	98703542	778	1.24E-05	1.82E-09	0.770
11	98703733	99613901	508	-7.37E-06	1.23E-09	0.834
11	99614560	101156501	814	2.29E-06	1.87E-09	0.958
11	101158517	101892922	344	-1.46E-05	5.65E-10	0.540
11	101894471	102668702	379	-2.21E-05	1.04E-09	0.493
11	102668882	103491310	383	7.05E-07	7.80E-10	0.980
11	103491586	104871322	551	-3.07E-06	1.01E-09	0.923
11	104872103	106251514	419	8.72E-06	6.87E-10	0.739
11	106252171	107489662	537	-4.43E-06	1.28E-09	0.901
11	107491606	108495975	327	-3.33E-05	8.90E-10	0.265
11	108496357	110701645	740	-1.58E-06	1.34E-09	0.966
11	110702636	111981853	469	2.21E-05	8.38E-10	0.446
11	111985737	113103996	469	-2.12E-05	9.11E-10	0.482
11	113105405	113958177	414	-8.63E-05	1.76E-09	0.040
11	113959061	114742322	448	-2.53E-05	1.06E-09	0.439

11	114743956	115627217	414	2.85E-05	9.67E-10	0.360
11	115652723	117097952	689	-1.14E-05	1.77E-09	0.788
11	117098436	118898319	898	2.96E-05	2.04E-09	0.512
11	118899218	120171371	574	8.00E-07	1.40E-09	0.983
11	120173354	121660636	713	-1.16E-05	1.51E-09	0.765
11	121661507	123289851	721	-2.90E-05	1.97E-09	0.513
11	123291301	124403522	618	1.55E-05	1.37E-09	0.675
11	124404297	125984562	770	4.58E-05	1.91E-09	0.295
11	125984970	126772347	528	8.66E-06	1.34E-09	0.813
11	126772905	127715860	459	-6.35E-06	1.07E-09	0.846
11	127716140	129186210	718	-8.12E-07	1.74E-09	0.984
11	129187995	129838375	321	-2.43E-05	9.03E-10	0.418
11	129839528	130892029	437	-3.74E-05	1.10E-09	0.260
11	130894131	131870763	676	2.85E-05	1.84E-09	0.507
11	131871467	132386355	340	-7.73E-06	6.95E-10	0.769
11	132386834	133679495	783	5.19E-05	2.34E-09	0.283
11	133679758	134946382	719	1.76E-05	3.34E-09	0.761
12	68657	665200	216	4.59E-06	5.66E-10	0.847
12	665655	1602602	326	2.78E-05	7.34E-10	0.304
12	1602652	2176046	281	2.13E-05	8.45E-10	0.464
12	2176265	2886299	325	-1.94E-05	8.85E-10	0.515
12	2886946	3558488	354	1.76E-05	1.01E-09	0.578
12	3559429	4693778	656	-3.83E-05	2.02E-09	0.393
12	4695425	5877653	808	2.47E-05	2.04E-09	0.584
12	5878686	6879279	441	2.96E-05	1.08E-09	0.368
12	8658644	10477423	257	4.12E-05	6.12E-10	0.096
12	10477712	11684725	448	-7.12E-06	8.04E-10	0.802
12	11687997	12530718	424	-7.62E-05	1.84E-09	0.076
12	12533494	13362446	381	-2.07E-05	1.15E-09	0.541
12	13362818	14658341	699	1.42E-05	2.14E-09	0.759
12	14659441	16423287	588	2.34E-05	1.15E-09	0.490
12	16423998	18466533	744	-9.83E-06	1.61E-09	0.807
12	18467618	19924288	582	-7.64E-05	1.47E-09	0.046
12	19925541	21568051	878	-1.12E-05	1.71E-09	0.787
12	21569730	22325878	305	-2.77E-05	5.81E-10	0.250
12	22327634	22943718	204	1.48E-05	4.56E-10	0.489
12	22944771	23819079	359	-4.99E-05	1.08E-09	0.128

12	23819364	24545631	412	8.28E-05	1.22E-09	0.018
12	24545834	25991825	716	2.89E-05	1.58E-09	0.468
12	25992543	27529660	775	-2.01E-06	1.86E-09	0.963
12	29111480	30354932	709	-2.65E-05	1.36E-09	0.472
12	27529712	29111136	700	1.18E-05	1.36E-09	0.750
12	30355547	31067009	415	-1.59E-05	7.43E-10	0.559
12	31067490	32081072	408	1.18E-05	1.05E-09	0.717
12	32082695	32645755	311	-7.85E-06	7.74E-10	0.778
12	32645848	33435901	369	-1.14E-05	7.09E-10	0.669
12	33436298	38231063	326	1.30E-06	4.89E-10	0.953
12	38233264	40017827	410	-3.22E-06	5.96E-10	0.895
12	40022029	41015211	475	2.78E-06	8.98E-10	0.926
12	41015333	42346846	665	1.21E-05	1.24E-09	0.732
12	42347363	43074061	261	5.99E-06	4.84E-10	0.785
12	43074434	43982146	422	5.82E-06	7.57E-10	0.832
12	43982342	44922863	220	6.82E-06	4.12E-10	0.737
12	44923334	46022099	343	-5.37E-06	6.23E-10	0.830
12	46023426	47504173	481	-4.71E-05	8.69E-10	0.110
12	47505424	48267360	361	1.93E-05	1.24E-09	0.584
12	48267635	48965461	365	-3.97E-05	1.56E-09	0.315
12	48967935	50160195	315	2.70E-05	8.05E-10	0.341
12	50160662	51582048	414	-5.18E-05	7.52E-10	0.059
12	51582188	52913730	700	3.20E-05	1.34E-09	0.382
12	52914211	54379670	717	4.14E-05	3.06E-09	0.454
12	54380016	56792337	763	-5.27E-05	1.55E-09	0.181
12	56795415	57534912	235	4.04E-05	4.40E-10	0.054
12	57535266	58748272	385	1.42E-06	6.18E-10	0.955
12	58748846	60859665	699	-4.12E-06	1.19E-09	0.905
12	60859809	61738896	266	7.53E-06	3.95E-10	0.705
12	61740561	63158569	602	1.90E-05	1.34E-09	0.603
12	63158712	63908647	359	-3.23E-05	8.09E-10	0.256
12	63910665	65383507	408	-4.40E-05	9.06E-10	0.143
12	65926411	67039427	476	-3.12E-05	8.93E-10	0.297
12	67039600	67817612	375	3.58E-05	9.00E-10	0.232
12	67818111	68740414	516	-2.15E-05	1.23E-09	0.540
12	68742112	69826093	486	-5.27E-05	1.32E-09	0.147
12	69826237	70815755	526	-1.96E-06	1.03E-09	0.951

12	70817279	71688642	413	-4.11E-05	1.01E-09	0.195
12	71690933	72513196	316	-8.88E-07	5.88E-10	0.971
12	72525849	74846997	634	1.11E-05	1.16E-09	0.743
12	75193041	76058346	327	-1.25E-05	6.20E-10	0.617
12	76060283	77098899	451	3.14E-05	9.71E-10	0.314
12	77099081	78398357	624	9.99E-06	1.34E-09	0.785
12	78400884	78925445	243	4.09E-06	6.44E-10	0.872
12	78926240	80944201	511	-1.45E-05	8.99E-10	0.630
12	80945774	83137872	721	-3.32E-06	1.53E-09	0.932
12	83143660	83854442	270	8.33E-06	6.66E-10	0.747
12	83856540	85014953	417	5.12E-06	6.62E-10	0.842
12	85016898	87296535	569	-4.38E-05	8.83E-10	0.141
12	87297692	87905878	166	1.86E-06	3.02E-10	0.915
12	87909726	88848006	218	-4.49E-06	3.88E-10	0.820
12	88851543	91827908	896	6.66E-05	1.82E-09	0.119
12	91827930	93792073	768	-5.39E-05	1.86E-09	0.212
12	93792655	95717265	1015	7.08E-06	2.51E-09	0.888
12	95717497	97439396	838	4.34E-05	1.86E-09	0.314
12	99224060	99816442	206	-3.41E-06	4.37E-10	0.870
12	97439589	99220284	912	-4.68E-05	2.17E-09	0.315
12	99816842	101857521	803	1.92E-05	1.81E-09	0.651
12	101857796	102961329	417	3.12E-05	8.76E-10	0.292
12	102961450	104011319	433	3.64E-05	9.44E-10	0.236
12	105817813	106155478	204	6.38E-06	5.50E-10	0.786
12	104012667	105816376	1163	3.15E-05	2.30E-09	0.512
12	106156001	107521770	612	9.61E-06	1.10E-09	0.772
12	107527013	108504440	424	4.01E-05	8.81E-10	0.176
12	108506696	110111462	700	-6.90E-05	1.80E-09	0.104
12	110112932	113027651	567	8.98E-06	1.06E-09	0.783
12	113030227	113877766	334	4.87E-05	7.36E-10	0.073
12	113885696	114882476	596	6.52E-05	1.45E-09	0.087
12	114883085	115326972	186	1.39E-05	7.00E-10	0.599
12	117087697	117644288	184	5.58E-06	3.99E-10	0.780
12	115329869	117087376	969	1.02E-05	2.54E-09	0.840
12	117644436	118888131	675	7.32E-06	1.64E-09	0.856
12	118888731	119301273	203	1.05E-05	5.43E-10	0.654
12	119303044	120007172	394	-5.78E-05	1.01E-09	0.069

12	120008093	121720040	644	-1.42E-05	1.18E-09	0.679
12	124710996	125416169	363	2.09E-05	1.38E-09	0.573
12	121721831	124710880	880	-2.97E-05	1.39E-09	0.427
12	125417493	126461057	519	-4.92E-06	1.41E-09	0.896
12	126463562	127382559	515	-5.02E-06	1.10E-09	0.880
12	127383648	128141516	402	-1.04E-06	1.24E-09	0.977
12	128141610	128810520	447	-1.24E-06	1.27E-09	0.972
12	128814615	129688509	572	5.71E-05	2.05E-09	0.207
12	129689639	130157939	346	2.14E-06	1.09E-09	0.948
12	130158997	130859973	457	1.80E-05	1.38E-09	0.628
12	130861322	131407836	344	6.15E-06	1.09E-09	0.852
12	131408716	132012266	376	-2.64E-05	9.32E-10	0.387
12	132014206	132706778	276	1.13E-06	6.47E-10	0.965
12	132807034	133841558	235	-8.21E-05	3.65E-09	0.174
13	19020095	20682304	395	2.11E-06	5.81E-10	0.930
13	20684769	21688499	462	1.25E-05	8.99E-10	0.678
13	21688974	22209012	221	7.78E-06	4.48E-10	0.713
13	22211354	23590611	815	-1.67E-05	2.49E-09	0.739
13	23590702	23827019	193	-1.89E-05	6.87E-10	0.471
13	23827815	24477568	384	1.32E-05	8.20E-10	0.645
13	24477971	25210959	503	-4.29E-06	1.23E-09	0.903
13	25212359	25639070	222	-2.43E-05	4.76E-10	0.265
13	25640695	27282973	736	-4.85E-05	1.43E-09	0.199
13	27283115	28283059	562	1.45E-07	1.35E-09	0.997
13	28284459	28876214	285	-1.58E-05	5.59E-10	0.503
13	28879332	29872965	453	-1.67E-05	9.83E-10	0.595
13	29873521	31379027	787	2.62E-05	1.93E-09	0.551
13	31384910	32983250	798	9.97E-06	1.62E-09	0.804
13	32984197	34681078	709	-5.60E-05	1.13E-09	0.096
13	34683262	36042839	439	-1.04E-05	9.88E-10	0.740
13	36043978	37027089	549	5.42E-06	1.06E-09	0.868
13	37029009	38290705	619	3.73E-05	1.07E-09	0.253
13	38291011	38877678	287	1.77E-06	4.89E-10	0.936
13	38887155	40179288	575	-3.03E-05	9.66E-10	0.330
13	40181792	41773356	639	-2.52E-05	1.24E-09	0.474
13	41774489	43099692	548	-1.73E-05	9.47E-10	0.573
13	43099889	44637493	786	2.45E-05	1.43E-09	0.517

13	46493234	46866562	161	-9.15E-06	2.62E-10	0.572
13	44638337	46491101	755	-3.42E-05	1.48E-09	0.374
13	48440893	49987145	462	6.89E-07	6.14E-10	0.978
13	46869621	48439005	725	-8.65E-06	1.34E-09	0.813
13	49987663	51254196	496	-2.41E-05	1.53E-09	0.539
13	51254712	53336663	702	-7.82E-06	1.15E-09	0.818
13	53337952	54681048	611	1.29E-05	1.19E-09	0.709
13	54681363	56433642	544	-1.59E-05	8.09E-10	0.577
13	56434161	58247964	413	-3.61E-06	5.17E-10	0.874
13	60087834	60854106	241	-3.84E-05	3.31E-10	0.035
13	58248124	60085069	714	-9.15E-06	1.32E-09	0.801
13	60855021	61589772	348	2.30E-06	5.79E-10	0.924
13	61591949	62912481	486	-7.75E-06	8.05E-10	0.785
13	62913542	64201284	415	-2.26E-06	7.38E-10	0.934
13	64202212	65339575	366	-1.26E-06	5.24E-10	0.956
13	65340421	66197786	243	3.73E-06	2.94E-10	0.828
13	66200191	67713633	592	2.66E-05	9.69E-10	0.393
13	67714255	69561090	645	1.69E-05	1.12E-09	0.613
13	69561894	70642326	429	3.63E-07	8.56E-10	0.990
13	70643712	71095217	239	5.32E-06	4.81E-10	0.808
13	71095483	71759743	313	-5.99E-06	7.60E-10	0.828
13	71763633	72958993	473	1.08E-05	9.05E-10	0.719
13	72959394	73795534	346	2.52E-07	1.45E-09	0.995
13	73796728	75084163	678	-3.78E-05	1.62E-09	0.348
13	75084473	76504672	666	1.29E-04	2.10E-09	0.005
13	78032193	78807439	318	1.88E-06	5.17E-10	0.934
13	76505282	78030962	671	2.24E-05	1.25E-09	0.527
13	78807836	80252742	553	-1.48E-05	8.60E-10	0.614
13	80257200	82580321	856	2.28E-05	1.52E-09	0.558
13	82580994	84132765	494	1.49E-06	6.33E-10	0.953
13	84133082	85035423	269	-1.45E-05	4.60E-10	0.499
13	85037313	85609664	262	2.35E-07	4.31E-10	0.991
13	85609760	87039195	528	1.54E-05	9.47E-10	0.617
13	87039360	88190520	314	1.04E-05	4.19E-10	0.613
13	88191656	89057861	239	8.44E-06	3.90E-10	0.669
13	89059635	90177618	305	1.36E-05	3.87E-10	0.490
13	90179364	90867683	318	-8.21E-06	6.09E-10	0.740

13	90869873	91967960	405	-4.50E-05	7.38E-10	0.098
13	91970313	92912119	365	5.22E-06	6.36E-10	0.836
13	92912423	93918144	412	-2.31E-05	7.35E-10	0.394
13	93918533	94562549	322	-5.89E-06	5.08E-10	0.794
13	94563601	95540024	474	2.14E-05	1.09E-09	0.518
13	97521207	98589842	367	-3.32E-05	6.58E-10	0.196
13	95540709	97520182	918	-1.60E-05	1.61E-09	0.689
13	98594975	100043997	777	-3.86E-05	1.45E-09	0.311
13	100044673	101392748	563	-1.07E-05	1.12E-09	0.750
13	101394726	102299385	584	1.88E-06	1.10E-09	0.955
13	102299498	103339911	558	2.78E-05	1.06E-09	0.393
13	103340536	104065375	505	1.74E-05	1.05E-09	0.591
13	104065534	104842764	367	-2.60E-06	8.42E-10	0.928
13	104843186	105990071	584	5.05E-06	1.49E-09	0.896
13	105990812	106439396	245	4.94E-06	5.62E-10	0.835
13	106442608	107785431	712	-1.74E-05	1.75E-09	0.678
13	107788025	108518444	439	4.99E-05	1.12E-09	0.136
13	108519987	109359792	437	-7.22E-06	1.10E-09	0.827
13	109359834	110338482	539	4.78E-05	1.49E-09	0.216
13	110338913	111432821	729	-2.86E-05	1.96E-09	0.519
13	111434814	113266084	883	-1.20E-05	2.02E-09	0.790
13	113269127	115109853	671	-9.50E-06	1.24E-09	0.788
14	20544512	20877114	188	-2.06E-05	4.79E-10	0.347
14	20878890	21267077	308	2.27E-05	1.34E-09	0.536
14	21271573	22378835	523	-5.26E-05	1.46E-09	0.169
14	22379132	22738105	229	3.60E-05	6.19E-10	0.148
14	22738161	23607730	506	-2.56E-05	1.42E-09	0.496
14	23607866	24904154	516	-5.82E-06	1.59E-09	0.884
14	26135167	26679549	244	6.75E-06	5.94E-10	0.782
14	24904240	26134815	654	3.87E-05	1.25E-09	0.275
14	26680794	28466225	589	-1.94E-05	1.19E-09	0.575
14	28466533	29196856	255	1.27E-05	5.10E-10	0.575
14	29198059	32014712	797	3.28E-06	1.81E-09	0.939
14	32016504	33033695	420	-5.04E-06	1.01E-09	0.874
14	36345767	36682357	143	1.03E-06	2.49E-10	0.948
14	34769883	36344816	498	1.78E-05	9.58E-10	0.566
14	33036081	34769821	1119	-1.13E-04	3.39E-09	0.051

14	36683516	38481516	689	4.40E-05	1.95E-09	0.318
14	38486187	39370633	361	-2.31E-05	7.01E-10	0.384
14	39370726	40336296	361	-4.89E-06	6.62E-10	0.849
14	40336990	41615299	437	-1.95E-06	7.11E-10	0.942
14	41615331	43065019	495	1.78E-05	8.17E-10	0.533
14	43068948	44541699	471	5.82E-06	1.12E-09	0.862
14	44542368	45247021	304	1.53E-05	6.25E-10	0.540
14	45250418	46311209	221	-9.63E-06	2.74E-10	0.561
14	46312678	47456738	355	1.26E-06	5.34E-10	0.957
14	47457846	48351664	389	7.01E-07	7.55E-10	0.980
14	48352461	49001226	256	-9.55E-06	5.95E-10	0.695
14	49004194	49955015	359	6.48E-06	6.54E-10	0.800
14	52078316	52761693	415	-1.47E-06	9.65E-10	0.962
14	49955381	52077965	934	3.12E-06	2.01E-09	0.944
14	52762622	54162101	504	2.19E-05	2.05E-09	0.629
14	54162655	55043520	312	1.73E-05	7.23E-10	0.520
14	55044905	56215082	474	-3.41E-05	1.11E-09	0.307
14	56215477	56949485	467	1.14E-05	8.90E-10	0.703
14	58449526	59135770	213	-9.86E-06	3.89E-10	0.617
14	56949644	58448681	677	2.01E-05	1.54E-09	0.608
14	63166807	63890839	304	8.79E-06	5.59E-10	0.710
14	61651278	63164649	666	3.41E-05	1.56E-09	0.388
14	63897095	65218457	418	-2.00E-05	6.70E-10	0.440
14	59135989	61649612	944	-2.94E-05	1.57E-09	0.458
14	65220775	66353557	440	7.77E-08	7.80E-10	0.998
14	66354477	67990648	525	1.17E-05	6.46E-10	0.645
14	67991028	69355606	630	-4.41E-05	2.01E-09	0.326
14	71132759	72213258	357	-1.95E-05	5.30E-10	0.398
14	69356353	71131097	787	7.26E-05	2.15E-09	0.117
14	72214790	72888979	421	2.90E-06	8.14E-10	0.919
14	72889376	74250730	550	1.30E-05	1.22E-09	0.710
14	74252993	75747118	538	-1.65E-05	7.76E-10	0.554
14	75747258	76583135	371	6.18E-06	8.20E-10	0.829
14	76583775	77332160	386	6.05E-06	1.02E-09	0.850
14	77332408	78421884	573	9.13E-06	1.17E-09	0.790
14	78422260	79026816	371	-9.54E-06	7.94E-10	0.735
14	80999361	81787255	327	9.83E-06	6.03E-10	0.689

14	79027504	80998547	757	4.74E-05	1.55E-09	0.229
14	81787481	82459297	259	1.35E-05	5.89E-10	0.579
14	84087979	85035235	352	-2.63E-05	6.70E-10	0.309
14	82461794	84087895	607	-2.43E-05	1.39E-09	0.514
14	85037974	86051751	438	-1.07E-05	1.06E-09	0.743
14	86053777	87334624	501	2.07E-05	9.97E-10	0.512
14	87335256	88641174	451	1.39E-05	8.28E-10	0.628
14	88641335	90069965	638	2.28E-05	1.31E-09	0.530
14	90075441	92098486	749	-1.80E-05	1.82E-09	0.673
14	92099719	93378838	646	4.89E-05	1.52E-09	0.209
14	93379151	94420996	456	-4.90E-05	1.07E-09	0.133
14	94421488	95347359	703	3.19E-05	1.62E-09	0.428
14	95347761	96250900	641	-7.30E-05	1.66E-09	0.073
14	96251418	97570260	662	2.11E-05	1.64E-09	0.603
14	97571437	98987472	651	-1.92E-05	1.46E-09	0.615
14	98989483	99980087	544	-6.06E-05	1.56E-09	0.125
14	99981105	101244250	481	-1.08E-05	1.02E-09	0.736
14	101244293	102339561	444	2.78E-05	1.48E-09	0.470
14	104761778	105440784	241	2.36E-05	6.14E-10	0.340
14	102341650	104759919	714	1.43E-04	2.06E-09	0.002
14	105443695	106552839	140	8.42E-07	2.76E-10	0.960
15	22362658	23288776	160	-1.18E-05	4.62E-10	0.582
15	23292175	24320772	301	-1.48E-05	8.59E-10	0.613
15	24321414	24976269	148	-3.77E-06	3.49E-10	0.840
15	25107905	25750941	208	6.25E-06	4.79E-10	0.775
15	25752114	26185554	310	-4.62E-05	9.49E-10	0.134
15	26186113	26675188	259	3.66E-05	7.86E-10	0.192
15	26676258	27265824	242	-1.21E-06	7.05E-10	0.964
15	27266835	27819408	254	-3.47E-06	6.28E-10	0.890
15	27820352	28325439	266	-5.82E-06	6.17E-10	0.815
15	28326536	29617158	230	-4.37E-06	4.69E-10	0.840
15	29617517	31314317	411	4.23E-05	1.14E-09	0.210
15	31315570	32177326	354	5.08E-05	1.38E-09	0.172
15	32180607	33251727	348	1.00E-05	9.33E-10	0.742
15	33253484	33792116	378	-2.39E-05	1.09E-09	0.469
15	33793179	34273308	356	1.37E-06	9.89E-10	0.965
15	34273346	34970673	239	-1.55E-05	5.19E-10	0.495

15	34971983	35941864	367	-4.03E-06	6.94E-10	0.878
15	35943377	36424581	299	-2.32E-06	6.57E-10	0.928
15	36425448	36968033	285	-9.91E-06	7.99E-10	0.726
15	36968291	37930544	442	1.28E-05	9.94E-10	0.684
15	37931874	38804104	337	-1.39E-05	7.29E-10	0.607
15	38805185	39518563	386	1.40E-05	8.83E-10	0.638
15	39519092	40382441	407	-1.63E-05	8.77E-10	0.583
15	40383779	41211827	277	-3.87E-06	7.85E-10	0.890
15	41212265	42222063	300	1.21E-05	4.19E-10	0.556
15	42222390	43473907	378	-9.29E-06	5.35E-10	0.688
15	43474655	44988610	261	7.61E-06	2.78E-10	0.648
15	44992475	46292382	424	-3.11E-05	6.81E-10	0.234
15	46294385	47000090	290	1.80E-05	4.85E-10	0.414
15	47000857	47977097	382	2.39E-06	6.42E-10	0.925
15	47979127	49075918	359	1.52E-05	5.66E-10	0.522
15	49078966	50004817	260	1.79E-05	3.55E-10	0.342
15	51682775	52589995	273	1.52E-05	3.17E-10	0.394
15	50006184	51681073	716	3.77E-05	1.12E-09	0.258
15	52590615	53014125	170	1.07E-05	2.49E-10	0.497
15	53015101	53766542	361	-8.01E-06	7.55E-10	0.771
15	53767876	54336662	313	-5.62E-06	6.36E-10	0.824
15	54337243	55134743	392	1.39E-05	7.99E-10	0.624
15	55135408	55469163	140	1.13E-05	2.52E-10	0.476
15	55470187	55877891	151	2.21E-05	3.16E-10	0.213
15	55879453	56784345	319	2.92E-05	5.63E-10	0.218
15	56784506	57637678	235	3.56E-06	3.16E-10	0.841
15	57638075	58438535	510	-1.24E-05	1.06E-09	0.704
15	58439935	59505191	502	-3.03E-06	1.11E-09	0.928
15	59507391	60018662	244	6.99E-05	4.88E-10	0.002
15	60019136	60670149	285	1.85E-05	6.17E-10	0.455
15	60670287	61803306	669	-4.44E-06	1.82E-09	0.917
15	61803892	62735671	460	-7.09E-06	8.73E-10	0.810
15	62737452	64335240	693	-3.67E-05	1.35E-09	0.318
15	65104668	66358474	354	3.09E-05	4.82E-10	0.159
15	66359486	66948204	296	3.19E-06	7.24E-10	0.906
15	68642047	69936117	406	1.93E-05	1.13E-09	0.565
15	66948951	68640917	759	-3.71E-05	1.27E-09	0.297

15	69938058	70772135	450	9.97E-05	1.22E-09	0.004
15	70772414	71919256	463	-2.24E-05	9.03E-10	0.457
15	74453873	75516403	321	-1.17E-04	7.85E-10	0.000
15	71920065	74447996	707	-4.76E-05	1.14E-09	0.160
15	75520945	76397358	160	-4.64E-06	2.51E-10	0.769
15	76397390	77910873	428	-1.25E-05	5.50E-10	0.594
15	77912408	78637877	276	-8.77E-07	6.88E-10	0.973
15	78640760	79259009	192	-9.74E-06	3.67E-10	0.611
15	79261077	79991408	437	1.43E-05	8.77E-10	0.629
15	79992629	80635045	302	-1.78E-05	7.50E-10	0.517
15	80637749	81604871	477	-2.28E-05	1.16E-09	0.504
15	81607249	84062334	626	1.17E-05	1.17E-09	0.733
15	84063245	85517022	507	-7.20E-07	1.05E-09	0.982
15	85518062	86341812	361	-1.96E-05	5.25E-10	0.392
15	86342119	86650301	159	1.65E-05	3.45E-10	0.375
15	86650331	87113409	248	-8.55E-07	5.24E-10	0.970
15	87114392	87693557	314	-7.64E-06	8.02E-10	0.787
15	87693885	88032107	160	-9.17E-06	3.51E-10	0.625
15	88032736	88742960	322	-2.95E-06	6.86E-10	0.910
15	88743108	89561223	336	-1.21E-05	8.71E-10	0.682
15	89562631	90452882	361	8.10E-06	6.55E-10	0.752
15	90453183	91221307	299	1.32E-05	5.68E-10	0.579
15	91226586	91745246	251	-1.83E-05	6.87E-10	0.484
15	91749626	92164207	203	3.80E-05	6.59E-10	0.139
15	92165068	92667377	253	-5.07E-06	6.74E-10	0.845
15	92669184	93590376	566	2.94E-05	1.36E-09	0.425
15	93591893	93908402	232	-3.88E-07	9.74E-10	0.990
15	93909052	94547933	368	1.30E-06	9.53E-10	0.966
15	94548924	95576904	643	3.82E-06	1.85E-09	0.929
15	95577096	96497729	429	-1.88E-05	1.21E-09	0.589
15	96500358	97091511	333	2.07E-06	9.24E-10	0.946
15	97092654	98496698	629	8.38E-05	1.94E-09	0.057
15	98497881	99128454	384	-8.46E-06	1.14E-09	0.802
15	100078196	100456641	152	-1.30E-05	3.42E-10	0.483
15	99128932	100077720	485	-3.10E-06	1.25E-09	0.930
15	100457692	101403655	489	3.10E-06	1.29E-09	0.931
15	101408616	101918220	304	2.20E-05	7.39E-10	0.419

15	101918830	102516259	287	3.49E-05	7.90E-10	0.215
16	60291	596605	208	-7.65E-06	5.89E-10	0.753
16	598838	945806	136	-6.35E-06	3.63E-10	0.739
16	946205	1547999	261	-6.65E-06	7.47E-10	0.808
16	1551082	2293296	333	2.82E-05	6.92E-10	0.284
16	2294890	3960458	489	-1.53E-05	1.26E-09	0.666
16	3961770	4689471	275	-4.54E-06	8.77E-10	0.878
16	4690187	5114499	203	3.78E-05	4.95E-10	0.090
16	5115228	5854496	426	-2.21E-05	1.21E-09	0.525
16	5855169	6264138	318	1.60E-05	1.08E-09	0.626
16	6264139	7045506	647	2.82E-05	1.80E-09	0.506
16	7046430	7524343	442	1.10E-05	1.21E-09	0.752
16	7525825	8236887	555	-2.37E-06	1.55E-09	0.952
16	8237157	8572350	256	2.80E-05	8.41E-10	0.334
16	8572557	8960864	188	9.16E-06	6.31E-10	0.715
16	8962907	9658861	335	-7.01E-05	1.35E-09	0.056
16	9659584	9997178	212	1.60E-05	5.02E-10	0.475
16	10002419	10430046	325	8.79E-06	8.14E-10	0.758
16	10431221	11493266	531	-2.35E-05	1.43E-09	0.535
16	11612436	12151685	190	1.63E-05	5.27E-10	0.479
16	12151797	12775407	451	1.38E-06	1.01E-09	0.965
16	12775897	13119544	140	-3.89E-06	3.97E-10	0.845
16	13120367	13978116	506	8.68E-07	1.28E-09	0.981
16	13979558	15885158	356	-8.37E-06	8.35E-10	0.772
16	15885866	16220858	240	6.04E-06	5.27E-10	0.792
16	16222763	17334499	272	2.55E-06	7.69E-10	0.927
16	17335780	18062164	300	3.88E-05	8.11E-10	0.174
16	18065944	20054371	508	-1.99E-05	1.20E-09	0.564
16	20055884	21235343	435	-8.95E-06	1.02E-09	0.779
16	21237217	23369433	478	3.10E-05	1.24E-09	0.379
16	23371110	24308825	377	-6.77E-06	8.81E-10	0.820
16	24309108	25589623	527	1.42E-05	1.28E-09	0.691
16	26683251	26943239	138	-3.14E-05	6.38E-10	0.213
16	25590147	26681997	620	-3.20E-05	1.87E-09	0.459
16	26944850	27445360	274	3.06E-05	9.81E-10	0.329
16	27446054	29023966	445	2.22E-05	8.53E-10	0.447
16	29694822	31380596	353	-4.46E-05	8.42E-10	0.124

16	31384554	47981754	267	-3.65E-05	5.48E-10	0.119
16	47982405	49006844	259	1.16E-06	7.14E-10	0.965
16	49007408	50694011	658	3.92E-05	1.87E-09	0.365
16	50698071	51700822	458	1.29E-05	1.27E-09	0.718
16	53848448	54671719	496	-3.71E-05	2.28E-09	0.437
16	51703888	53845487	830	7.48E-05	1.85E-09	0.082
16	54673195	55505040	496	-1.44E-05	1.38E-09	0.699
16	55506137	56906006	604	-7.94E-05	1.36E-09	0.031
16	56908045	57663112	346	-2.95E-05	1.12E-09	0.379
16	57663619	58508278	391	-3.16E-05	2.00E-09	0.479
16	59889269	60744157	301	-3.74E-06	7.72E-10	0.893
16	58510034	59888170	534	2.87E-05	1.12E-09	0.391
16	60745208	61991704	352	-1.65E-05	7.54E-10	0.548
16	61993462	63395786	493	-6.36E-05	1.38E-09	0.087
16	63396006	64714759	477	-5.48E-05	1.02E-09	0.087
16	64716851	65538017	376	-1.42E-05	7.57E-10	0.605
16	65538484	68429077	907	-1.09E-06	1.89E-09	0.980
16	72915753	73542956	354	2.34E-05	1.22E-09	0.504
16	68429894	70885818	619	-3.52E-05	1.12E-09	0.293
16	70887506	72915425	541	-5.72E-05	9.99E-10	0.070
16	73543220	74724933	470	2.05E-05	1.17E-09	0.550
16	74730819	75517115	291	6.16E-07	7.22E-10	0.982
16	75518129	76056374	169	-9.45E-06	3.82E-10	0.629
16	76058697	76987262	437	-7.85E-06	9.53E-10	0.799
16	76988130	77455781	322	1.65E-05	8.49E-10	0.572
16	77456244	77688760	199	-4.16E-06	4.90E-10	0.851
16	77689074	78446696	584	-6.39E-07	1.71E-09	0.988
16	78452348	78709939	217	-4.06E-05	6.78E-10	0.119
16	78710328	79368760	703	6.70E-06	2.18E-09	0.886
16	79369779	80184442	562	-6.01E-05	1.41E-09	0.110
16	80184495	81209234	657	-4.92E-05	1.54E-09	0.211
16	82482458	83078581	571	2.50E-05	1.75E-09	0.551
16	81209631	82481987	785	1.03E-04	2.57E-09	0.041
16	84646612	85146709	319	-1.64E-05	1.07E-09	0.616
16	83078959	83971446	826	1.73E-05	2.39E-09	0.724
16	83972415	84645588	626	1.08E-05	2.31E-09	0.822
16	85146935	85663943	211	-2.98E-05	1.01E-09	0.347

16	85664453	86221644	412	3.79E-05	1.44E-09	0.319
16	86745955	87173290	254	1.00E-06	1.03E-09	0.975
16	86222417	86745679	508	-5.26E-06	1.87E-09	0.903
16	87175053	88389287	532	-2.99E-06	1.88E-09	0.945
16	88439429	88964934	242	-1.62E-05	8.68E-10	0.583
16	88966667	89604610	217	1.56E-05	6.32E-10	0.536
16	89605495	90292767	240	-9.64E-07	6.38E-10	0.970
17	828	296442	133	2.00E-05	1.10E-09	0.546
17	396660	820493	179	8.80E-05	1.78E-09	0.037
17	820511	1480393	260	-1.98E-05	1.26E-09	0.577
17	1481897	2613925	472	-6.75E-06	1.64E-09	0.868
17	2616352	3425788	387	-8.32E-06	1.58E-09	0.834
17	3426133	4321755	384	-6.94E-06	1.31E-09	0.848
17	4322053	4650487	176	2.85E-05	8.93E-10	0.340
17	4652156	5573766	371	8.21E-06	1.19E-09	0.812
17	5574433	6570522	608	8.51E-06	2.49E-09	0.865
17	6572170	6975193	181	1.93E-05	7.78E-10	0.489
17	6976284	7723513	318	5.25E-05	1.47E-09	0.171
17	8705612	9229793	253	-3.63E-05	1.44E-09	0.338
17	7725660	8704551	399	1.70E-05	2.71E-09	0.743
17	9233113	9714549	223	-4.01E-06	8.79E-10	0.893
17	9714995	10573151	522	7.67E-06	2.00E-09	0.864
17	10573457	11667346	523	3.08E-05	2.01E-09	0.492
17	11668104	13000883	588	-1.75E-05	3.32E-09	0.761
17	13001700	13625425	351	2.15E-05	1.54E-09	0.584
17	13626102	14209988	323	-1.10E-05	1.28E-09	0.759
17	14210015	14499605	185	-1.11E-05	7.93E-10	0.694
17	14500054	14859339	269	1.39E-05	1.00E-09	0.660
17	14860784	16319512	496	6.35E-05	1.73E-09	0.127
17	16321959	17292491	308	1.11E-05	1.09E-09	0.737
17	17299591	18446092	382	-2.34E-05	1.06E-09	0.472
17	18456679	19666001	259	8.48E-06	8.25E-10	0.768
17	20679485	21284673	159	-1.52E-06	7.59E-10	0.956
17	21285166	25677370	211	-6.08E-06	6.18E-10	0.807
17	25679294	26272308	203	-1.52E-06	6.21E-10	0.952
17	26273607	27884779	447	-2.27E-05	1.27E-09	0.523
17	27885675	29259899	376	-7.27E-06	1.16E-09	0.831

17	29262773	30878805	457	2.38E-05	1.49E-09	0.538
17	30879433	31989867	616	-4.68E-05	2.19E-09	0.317
17	31990385	32677709	410	1.04E-05	1.40E-09	0.781
17	32677947	33614452	507	-1.16E-05	1.83E-09	0.785
17	33616080	34393028	274	-2.68E-05	8.73E-10	0.364
17	34393296	35725997	364	-1.50E-06	1.21E-09	0.966
17	37344586	38649203	332	-1.80E-05	1.28E-09	0.614
17	35731855	37341235	493	9.68E-05	1.42E-08	0.416
17	38652220	39657179	455	8.42E-06	1.26E-09	0.813
17	39657337	40231221	206	-2.13E-06	5.92E-10	0.930
17	40232810	41769732	392	-5.07E-06	1.07E-09	0.877
17	41771040	43317574	552	-1.58E-05	1.71E-09	0.703
17	44863413	45874715	302	2.33E-06	8.99E-10	0.938
17	45876022	46828302	406	-1.12E-05	1.48E-09	0.770
17	46828412	48027295	402	2.49E-05	2.09E-09	0.586
17	48027688	48832008	360	1.42E-05	1.58E-09	0.721
17	48832437	49892805	428	-3.78E-05	1.31E-09	0.296
17	49894599	51282380	527	-2.02E-06	1.43E-09	0.957
17	52863321	53338298	227	1.14E-05	7.39E-10	0.675
17	51284441	52861905	537	-4.74E-05	1.72E-09	0.254
17	53346718	54164464	385	-9.08E-06	1.20E-09	0.793
17	54164935	55278525	429	-2.13E-05	1.53E-09	0.586
17	57487538	59307733	303	-1.65E-05	8.19E-10	0.565
17	55279508	57486793	880	4.72E-05	3.09E-09	0.396
17	59309085	60883279	464	-1.34E-05	1.47E-09	0.726
17	60885287	62035655	261	-1.01E-05	6.86E-10	0.701
17	62037685	64434272	754	9.32E-06	2.45E-09	0.851
17	64434524	65013985	328	1.34E-05	1.22E-09	0.702
17	65016683	66315468	382	-7.22E-05	1.41E-09	0.054
17	66318466	66903293	310	2.97E-05	1.11E-09	0.374
17	66906217	68199133	460	7.12E-06	1.48E-09	0.853
17	68199783	68875761	304	-5.28E-06	1.02E-09	0.869
17	68877746	69242135	206	-1.02E-04	2.37E-09	0.036
17	69242932	70147289	397	-5.64E-05	1.92E-09	0.198
17	70148198	70947580	477	2.70E-06	2.07E-09	0.953
17	70948535	71825523	480	1.78E-05	1.94E-09	0.687
17	71829519	72740027	438	-7.08E-07	1.74E-09	0.986

17	72742484	74016834	403	-2.92E-05	1.28E-09	0.413
17	74018640	74909405	368	2.93E-05	1.21E-09	0.400
17	74910943	75471247	314	7.72E-05	1.43E-09	0.041
17	75476450	75842792	220	4.87E-07	1.09E-09	0.988
17	75844316	76445231	219	1.98E-05	1.01E-09	0.534
17	76446689	77093768	322	-1.81E-05	1.33E-09	0.620
17	77094717	77468875	306	6.16E-05	1.57E-09	0.119
17	78780686	79683249	363	-3.57E-05	1.38E-09	0.336
17	77471383	78780080	510	3.93E-05	1.94E-09	0.373
17	79683698	80969550	416	-2.56E-05	1.26E-09	0.470
18	349293	906343	394	-3.53E-06	1.04E-09	0.913
18	906673	1940581	435	-9.02E-06	9.95E-10	0.775
18	1940863	2206099	166	-4.45E-05	1.07E-09	0.173
18	2206891	2889826	315	2.18E-05	7.65E-10	0.430
18	2891847	3357120	219	-2.39E-05	6.53E-10	0.350
18	3359237	3861997	218	-3.01E-07	8.77E-10	0.992
18	3862461	4597021	433	-9.84E-06	1.24E-09	0.780
18	4601573	5197395	229	2.93E-05	6.13E-10	0.237
18	5198562	5852964	327	-3.04E-05	7.92E-10	0.281
18	5855598	6630394	361	1.23E-05	1.14E-09	0.716
18	6630840	7373590	414	3.58E-05	1.10E-09	0.281
18	7374066	8440357	509	2.83E-05	1.06E-09	0.384
18	8440558	9414901	548	7.79E-05	1.41E-09	0.038
18	10465005	11028008	322	2.57E-05	6.36E-10	0.309
18	9415389	10462537	665	-2.50E-06	2.02E-09	0.956
18	11028129	11529011	226	-2.74E-06	5.85E-10	0.910
18	11530996	12737037	468	2.05E-05	8.66E-10	0.486
18	12737582	14358413	604	-3.36E-05	1.15E-09	0.323
18	18527782	21622994	743	3.35E-05	1.48E-09	0.383
18	21623416	22745505	571	2.82E-05	1.29E-09	0.432
18	22746547	24088696	488	-3.25E-05	9.46E-10	0.291
18	24088831	24971742	490	-3.44E-05	7.95E-10	0.223
18	24972728	26873513	697	3.99E-05	1.20E-09	0.251
18	26874132	28037427	397	-3.87E-06	6.43E-10	0.879
18	28037840	28560707	235	2.36E-05	4.85E-10	0.283
18	28561634	29563172	480	9.56E-06	8.98E-10	0.750
18	31777336	32456417	204	-1.81E-06	3.05E-10	0.918

18	29564408	31774120	674	2.76E-05	9.90E-10	0.380
18	32457437	33791804	485	5.59E-06	8.23E-10	0.846
18	35073616	35837704	322	7.84E-06	5.48E-10	0.738
18	33792671	35072855	572	-1.69E-05	9.39E-10	0.580
18	35838871	36364691	241	2.52E-05	4.87E-10	0.254
18	36365021	37682827	411	-4.25E-06	6.58E-10	0.868
18	37683967	38766853	525	5.96E-05	1.58E-09	0.134
18	38767938	39891215	366	-6.17E-06	6.03E-10	0.802
18	43295347	43881224	239	-4.33E-06	3.55E-10	0.818
18	42606091	43295247	413	5.83E-05	8.15E-10	0.041
18	39892281	42606024	955	9.12E-05	1.57E-09	0.021
18	43881706	45313390	679	1.07E-05	1.18E-09	0.755
18	45314528	46208355	424	6.15E-05	9.91E-10	0.051
18	46209339	47262925	482	1.76E-05	9.50E-10	0.568
18	47263681	47814821	330	1.66E-05	5.50E-10	0.480
18	47816777	48784027	395	-9.45E-06	6.62E-10	0.714
18	50152229	51059341	375	3.09E-06	5.98E-10	0.899
18	48784953	50151479	573	2.90E-05	1.19E-09	0.401
18	51059850	51952586	345	1.00E-05	4.98E-10	0.653
18	51953945	52707502	198	-1.97E-05	5.08E-10	0.381
18	52709949	53763665	408	1.24E-05	8.16E-10	0.664
18	53764157	55089715	500	7.84E-06	8.59E-10	0.789
18	55090919	55808073	393	2.35E-05	9.46E-10	0.444
18	55808692	56752598	490	1.93E-05	1.03E-09	0.548
18	56754050	57504565	479	1.98E-05	1.04E-09	0.540
18	57505073	58816387	484	-1.02E-06	8.45E-10	0.972
18	58817565	59585320	377	6.62E-06	8.80E-10	0.823
18	60823554	61439643	276	-1.38E-06	4.96E-10	0.950
18	59585959	60822264	549	-1.46E-05	1.02E-09	0.648
18	61441801	62075853	293	-8.00E-06	4.46E-10	0.705
18	62078305	63110788	403	1.83E-09	7.57E-10	1.000
18	63111628	64250279	397	-3.62E-06	6.46E-10	0.887
18	65830606	66404071	323	2.09E-08	5.91E-10	0.999
18	64251305	65830031	736	6.89E-06	1.53E-09	0.860
18	66404118	67569547	516	-1.31E-05	1.28E-09	0.714
18	67574191	69038112	511	-2.09E-05	1.21E-09	0.548
18	70094975	70717286	329	4.23E-06	7.43E-10	0.877

18	69039235	70094707	485	4.03E-06	1.12E-09	0.904
18	70719886	71243399	284	1.56E-05	6.84E-10	0.552
18	71244692	71892202	389	-5.46E-05	1.22E-09	0.119
18	71892443	72519795	346	1.27E-05	6.96E-10	0.630
18	72521276	73897594	733	-8.43E-06	1.52E-09	0.828
18	75029584	75473982	288	1.61E-05	7.30E-10	0.551
18	73898846	75028501	623	-3.54E-05	1.74E-09	0.397
18	75474664	75975253	233	2.07E-05	6.09E-10	0.401
18	75976567	77151520	503	-4.02E-05	1.33E-09	0.269
18	77151867	78017074	335	1.43E-05	6.99E-10	0.589
19	157892	757412	228	-1.57E-05	1.30E-09	0.663
19	759261	1089241	159	-3.26E-05	7.87E-10	0.245
19	1090803	1920712	265	7.49E-05	1.59E-09	0.061
19	1922476	2815372	342	-3.11E-06	1.40E-09	0.934
19	2816339	3301333	213	1.97E-05	9.40E-10	0.520
19	3304779	4347573	350	-5.04E-06	1.88E-09	0.907
19	4349416	5191547	337	3.18E-05	1.52E-09	0.415
19	5192372	5766090	218	-4.46E-05	1.09E-09	0.176
19	5767585	6612779	264	-3.48E-06	1.04E-09	0.914
19	6614637	7309688	317	1.14E-05	1.42E-09	0.761
19	7312727	8684707	486	-1.95E-05	2.10E-09	0.671
19	9013231	10029966	390	6.34E-06	1.13E-09	0.850
19	10030690	11279257	438	1.69E-06	2.50E-09	0.973
19	11280183	12497411	332	6.40E-07	1.06E-09	0.984
19	12500149	13903388	410	-1.73E-05	1.48E-09	0.654
19	15553878	16251518	445	4.38E-06	1.36E-09	0.906
19	13906229	15549939	595	-1.53E-05	2.21E-09	0.744
19	17944900	18506666	243	2.50E-05	9.48E-10	0.416
19	18506815	19873269	430	-1.07E-05	1.29E-09	0.767
19	16252072	17942927	622	-6.58E-06	2.38E-09	0.893
19	19873640	20520392	140	1.22E-05	4.44E-10	0.564
19	21379657	22036399	158	-6.65E-06	4.52E-10	0.754
19	22037866	22803640	245	-1.43E-05	7.05E-10	0.589
19	22805247	24059424	282	1.63E-06	7.36E-10	0.952
19	24059507	28555771	270	-5.12E-06	7.92E-10	0.856
19	28556284	28917565	189	-4.49E-06	5.52E-10	0.848
19	30113991	30858084	259	1.20E-05	1.03E-09	0.708

19	29318443	30113252	466	2.86E-07	1.50E-09	0.994
19	30858965	32136133	463	-8.05E-05	1.53E-09	0.039
19	33764103	34067891	139	4.35E-06	5.35E-10	0.851
19	32145233	33763475	600	7.07E-05	1.93E-09	0.108
19	35333176	36045772	366	3.68E-06	1.37E-09	0.921
19	34068435	35331436	447	-1.00E-05	1.59E-09	0.801
19	39371014	39757572	147	-8.68E-06	7.87E-10	0.757
19	36047098	37454119	411	3.40E-05	1.75E-09	0.416
19	41442597	41841098	165	8.30E-06	4.88E-10	0.707
19	37455512	39370456	576	5.14E-05	3.28E-09	0.370
19	39757926	41440863	613	-5.52E-05	2.00E-09	0.217
19	41843461	43954915	369	-6.65E-05	2.58E-09	0.190
19	43956095	44880657	396	-8.35E-06	1.20E-09	0.810
19	44882157	45255679	171	1.51E-05	7.77E-10	0.589
19	45259002	46433733	399	6.97E-06	1.55E-09	0.859
19	46433891	47333204	330	1.47E-05	1.42E-09	0.697
19	47333497	48509893	371	-2.44E-05	1.36E-09	0.507
19	49491035	49923166	159	-1.50E-05	5.54E-10	0.525
19	48697961	49485758	337	-1.34E-04	1.88E-09	0.002
19	49925208	50774141	253	-1.67E-07	9.24E-10	0.996
19	50774581	51331589	225	3.03E-05	9.89E-10	0.335
19	52217405	52604625	222	-1.26E-05	7.20E-10	0.638
19	51332061	52216563	534	2.24E-05	6.01E-09	0.772
19	52606936	53107086	222	-2.25E-05	1.03E-09	0.483
19	53107937	53537923	157	6.74E-06	7.79E-10	0.809
19	53538505	53899797	201	-1.71E-05	8.38E-10	0.555
19	54099626	54702450	223	-5.09E-05	1.39E-09	0.173
19	54704266	55174498	212	2.07E-05	1.05E-09	0.522
19	55175009	55791380	260	-1.02E-06	1.15E-09	0.976
19	55794266	56364684	248	-1.82E-06	1.15E-09	0.957
19	56365323	56898342	291	6.75E-05	1.49E-09	0.081
19	57952035	58531715	261	-1.40E-05	7.14E-10	0.601
19	56898853	57950377	491	1.95E-05	1.91E-09	0.656
19	58532756	59118784	238	-1.34E-05	7.52E-10	0.625
20	246392	887800	378	-2.92E-05	1.80E-09	0.491
20	888523	1445821	369	-6.84E-08	1.31E-09	0.998
20	1446580	1897442	256	-8.39E-06	7.26E-10	0.755

20	3083151	3655085	235	6.05E-05	5.15E-10	0.008
20	1899473	3081680	692	5.00E-05	1.90E-09	0.251
20	3655943	4051325	176	-2.02E-05	4.93E-10	0.364
20	4053020	4701211	402	-1.56E-05	1.47E-09	0.684
20	4701706	5304961	360	-6.85E-07	1.07E-09	0.983
20	5305400	6636139	709	-5.18E-05	2.09E-09	0.257
20	6637234	7065163	285	5.22E-05	9.44E-10	0.089
20	8601114	8881586	198	8.08E-07	4.18E-10	0.968
20	7066818	8600105	764	5.82E-06	1.81E-09	0.891
20	8884009	9745450	471	3.90E-06	1.19E-09	0.910
20	11014704	11534388	291	1.70E-05	6.58E-10	0.507
20	11534488	12153352	274	2.20E-05	6.59E-10	0.391
20	9746345	11013570	694	3.21E-05	2.07E-09	0.481
20	13201177	13666198	193	-6.49E-06	5.20E-10	0.776
20	12153396	13199755	620	-9.57E-06	1.62E-09	0.812
20	13670311	14929217	486	8.08E-07	1.15E-09	0.981
20	15148231	16150464	705	1.56E-05	2.02E-09	0.728
20	17449713	17905102	299	-1.32E-05	9.68E-10	0.670
20	17906490	18896415	440	-2.13E-05	9.94E-10	0.499
20	16151566	17449361	878	-6.73E-06	2.27E-09	0.888
20	18896895	19707457	462	-5.08E-05	1.15E-09	0.135
20	19708351	21676556	819	-7.98E-05	2.06E-09	0.079
20	21679930	22779512	406	2.76E-06	7.69E-10	0.921
20	22779865	23568490	399	-6.95E-06	8.81E-10	0.815
20	23568495	24053655	219	1.46E-05	4.86E-10	0.507
20	24054931	25019099	417	-1.64E-05	8.86E-10	0.581
20	25020047	26319418	309	5.96E-07	6.06E-10	0.981
20	26319535	30570889	234	9.23E-06	7.72E-10	0.740
20	30574455	31613985	334	-6.71E-06	6.78E-10	0.797
20	33885155	34891416	221	-3.49E-06	3.85E-10	0.859
20	34892880	35892530	205	1.95E-05	4.53E-10	0.359
20	31615242	33883015	705	-5.76E-05	1.53E-09	0.140
20	35899774	36904375	395	1.75E-06	8.32E-10	0.952
20	36905894	37875045	354	3.97E-06	7.80E-10	0.887
20	37875309	38431408	230	4.13E-06	4.45E-10	0.845
20	38435302	39453533	470	1.09E-05	1.11E-09	0.743
20	39455778	40584825	395	-9.12E-06	9.48E-10	0.767

20	40585126	41194048	393	-2.92E-06	1.07E-09	0.929
20	41195111	42188284	488	-9.14E-06	1.37E-09	0.805
20	42188914	43257406	508	6.24E-06	1.30E-09	0.863
20	44960997	45649369	359	1.47E-05	9.34E-10	0.630
20	43259306	44960236	711	6.02E-05	1.58E-09	0.130
20	45650139	46540102	374	-5.99E-06	1.14E-09	0.859
20	48081983	48655016	227	1.73E-05	7.86E-10	0.538
20	46540587	48080850	770	-8.60E-06	1.83E-09	0.840
20	48658113	49385779	312	-1.57E-05	8.68E-10	0.595
20	49387520	49945761	256	-8.49E-06	1.04E-09	0.793
20	49948016	50633472	360	5.82E-06	1.07E-09	0.858
20	50635347	51389135	319	-8.56E-06	7.29E-10	0.751
20	51389670	51884594	263	-2.01E-05	7.85E-10	0.472
20	51885269	52525657	292	-2.09E-06	1.39E-09	0.955
20	52526840	53406846	485	1.69E-05	1.36E-09	0.647
20	53408035	54321635	409	2.35E-05	9.87E-10	0.454
20	54321811	55431836	547	1.76E-05	1.46E-09	0.646
20	55434683	56214465	499	4.00E-05	1.69E-09	0.330
20	56216001	56679942	290	2.67E-05	9.44E-10	0.386
20	56679960	57319010	352	-5.37E-05	9.99E-10	0.089
20	57320545	58295563	478	-2.79E-05	1.61E-09	0.486
20	59361382	59892000	354	-6.44E-06	1.17E-09	0.850
20	58296350	59361064	553	-3.65E-05	1.45E-09	0.338
20	59892231	61091395	659	-1.87E-05	2.81E-09	0.724
20	61301855	62119318	400	-1.69E-05	1.37E-09	0.648
20	62119875	62962870	341	2.16E-07	1.53E-09	0.996
21	15451950	16139409	423	-2.91E-05	9.25E-10	0.339
21	17305471	18457656	408	-2.33E-05	1.33E-09	0.524
21	18457828	19151566	246	-3.08E-05	8.90E-10	0.302
21	16140244	17301757	441	3.94E-05	1.05E-09	0.223
21	19151940	20198061	542	-2.94E-05	1.13E-09	0.382
21	21022840	21905609	384	7.91E-06	9.44E-10	0.797
21	20199390	21022168	394	-6.31E-06	8.34E-10	0.827
21	24695869	25083563	190	-1.31E-06	4.01E-10	0.948
21	23270321	24695405	540	-3.47E-05	1.11E-09	0.299
21	25090174	25898082	385	-2.22E-05	7.29E-10	0.411
21	25899516	26465774	241	-3.10E-05	5.04E-10	0.167

21	21905658	23268216	670	-2.22E-05	1.36E-09	0.546
21	26467796	27776736	496	-8.15E-07	9.89E-10	0.979
21	27777181	28205789	289	2.43E-05	6.71E-10	0.348
21	28206883	28705495	309	3.16E-05	7.40E-10	0.245
21	28705732	29515396	352	-1.21E-05	8.18E-10	0.672
21	29515884	30844467	550	-1.60E-05	7.94E-10	0.571
21	30845873	32211778	531	1.22E-05	8.43E-10	0.673
21	32213678	33270766	453	1.50E-05	9.95E-10	0.633
21	33273162	33988973	358	-4.85E-07	7.30E-10	0.986
21	33991368	35219895	507	1.59E-07	1.44E-09	0.997
21	35220001	36188046	457	4.63E-05	1.95E-09	0.294
21	36190040	37303320	549	-3.98E-05	1.10E-09	0.230
21	37305159	37868817	300	-6.20E-05	5.54E-10	0.008
21	37870518	38429082	265	-3.62E-06	5.65E-10	0.879
21	38429389	39248065	402	2.04E-05	6.16E-10	0.411
21	39250321	40115483	422	-9.67E-06	9.55E-10	0.754
21	41260363	41705756	344	-3.56E-06	8.02E-10	0.900
21	40116044	41095241	608	-3.66E-05	1.20E-09	0.292
21	41707453	42543663	491	3.89E-05	1.16E-09	0.254
21	44690856	45598522	356	6.65E-06	5.84E-10	0.783
21	42545250	43342046	530	-6.86E-05	1.83E-09	0.109
21	45603905	46174491	279	-3.19E-05	6.86E-10	0.223
21	43343190	44632513	846	-2.84E-05	1.95E-09	0.519
21	46174981	46740019	297	-1.87E-06	5.75E-10	0.938
21	46741287	47491264	334	6.98E-06	6.51E-10	0.784
21	47491975	48119635	293	-3.02E-05	4.90E-10	0.173
22	17187276	17569017	168	-2.39E-05	4.65E-10	0.267
22	18333902	19138442	298	-3.08E-05	7.68E-10	0.266
22	17569439	18333467	393	3.76E-05	1.34E-09	0.304
22	19139368	20000428	424	3.00E-05	1.32E-09	0.410
22	20001006	20952650	273	-5.38E-05	7.91E-10	0.056
22	20952972	21863717	295	-2.29E-06	6.32E-10	0.927
22	21866569	23015302	370	1.14E-05	9.20E-10	0.708
22	23016570	23691564	208	-1.54E-06	4.80E-10	0.944
22	23697301	24370194	307	-2.43E-05	9.52E-10	0.430
22	24375019	25416292	359	-2.11E-05	7.92E-10	0.454
22	25419524	26113333	227	-9.92E-06	6.20E-10	0.690

22	26123092	26735929	447	-4.78E-05	1.30E-09	0.185
22	26736812	27294543	387	2.29E-05	1.16E-09	0.502
22	27295234	27653424	353	-6.46E-06	1.24E-09	0.854
22	29456360	30604102	391	-4.50E-05	7.77E-10	0.107
22	27654838	29454477	731	-4.81E-05	2.13E-09	0.297
22	30605601	31534127	380	-8.70E-07	9.29E-10	0.977
22	31535872	32324140	238	1.60E-06	4.05E-10	0.936
22	32324692	32856999	277	-1.66E-05	6.68E-10	0.521
22	33512106	33896495	178	1.16E-05	5.65E-10	0.625
22	32857285	33511391	411	-3.24E-06	1.16E-09	0.924
22	35865218	36131498	134	-1.43E-05	4.35E-10	0.492
22	34983275	35862655	468	-4.17E-06	8.90E-10	0.889
22	33898114	34982457	617	-3.60E-06	1.44E-09	0.924
22	36132263	36922822	314	2.33E-05	8.28E-10	0.418
22	36923144	37558356	398	4.44E-05	1.18E-09	0.196
22	38473398	39302783	269	1.97E-05	7.39E-10	0.468
22	37560592	38467784	418	-1.59E-05	1.01E-09	0.617
22	39304509	40092866	321	2.57E-05	8.10E-10	0.367
22	40095174	41405753	318	-2.08E-05	6.91E-10	0.430
22	43187900	43645335	232	-6.13E-05	3.29E-09	0.286
22	41406919	42870441	427	-2.42E-05	1.02E-09	0.448
22	43646241	44317084	380	2.30E-06	8.88E-10	0.938
22	44317416	44818986	414	5.27E-05	1.35E-09	0.151
22	45444397	45895495	268	2.61E-06	7.05E-10	0.922
22	44820796	45443845	418	-1.28E-05	1.48E-09	0.739
22	45895856	46512413	262	4.99E-06	8.48E-10	0.864
22	46513376	47106715	271	-2.44E-05	8.07E-10	0.391
22	47937891	48479731	373	-2.80E-05	1.06E-09	0.391
22	47106800	47937655	434	-9.92E-07	1.07E-09	0.976
22	49082979	49521080	313	-2.02E-05	1.20E-09	0.559
22	49522184	49751418	246	-1.41E-06	9.02E-10	0.963
22	48481280	49081753	464	9.75E-06	1.61E-09	0.808
22	49751512	50488153	335	8.78E-06	8.88E-10	0.768
22	50488995	51237713	281	-1.18E-05	8.23E-10	0.680
**Prostate cancer and COVID-19 hospitalization**						
1	1103150	2320702	359	-6.92E-05	3.07E-09	0.212
1	2321099	3065568	234	-7.18E-05	2.33E-09	0.137

1	3066761	3679461	290	-1.43E-04	5.55E-09	0.054
1	3679775	4749076	632	-1.19E-04	6.48E-09	0.138
1	4750013	5000086	168	-1.07E-05	1.75E-09	0.797
1	5000970	5471517	301	6.92E-05	3.10E-09	0.214
1	5472626	6133700	338	3.43E-05	3.18E-09	0.543
1	6134990	7245669	447	1.00E-05	3.52E-09	0.866
1	7245922	7604778	205	-2.48E-05	1.94E-09	0.574
1	7604831	9067142	513	9.96E-06	4.18E-09	0.878
1	9067673	9959051	319	8.01E-05	5.53E-09	0.281
1	9959748	11774956	584	7.70E-05	4.45E-09	0.248
1	11776196	12147753	192	-4.90E-05	1.43E-09	0.196
1	12150385	14625524	729	1.17E-04	7.57E-09	0.179
1	14626896	15423481	421	-7.29E-05	4.76E-09	0.291
1	15424441	17123862	613	-5.11E-05	4.21E-09	0.431
1	17176635	17872659	334	-2.13E-05	2.49E-09	0.670
1	17874843	18484945	341	1.20E-06	3.27E-09	0.983
1	18486272	20022063	799	-5.64E-05	7.59E-09	0.517
1	20023833	20763899	370	1.46E-05	4.32E-09	0.825
1	20765245	22634131	796	-6.52E-05	6.90E-09	0.432
1	22636103	23082667	208	-4.65E-05	2.07E-09	0.307
1	23085227	24527501	491	-6.78E-05	3.61E-09	0.259
1	24528210	25305673	317	-2.66E-05	3.14E-09	0.636
1	25305783	26976424	610	-1.33E-05	4.40E-09	0.841
1	26981479	28160199	205	6.20E-06	1.76E-09	0.883
1	28168872	29181525	242	-4.06E-05	1.50E-09	0.294
1	29184254	30792409	735	-4.37E-05	5.85E-09	0.568
1	30792984	32042421	610	9.69E-05	3.90E-09	0.121
1	32042540	34016219	486	4.72E-05	3.09E-09	0.396
1	34017349	34797348	442	-1.83E-05	3.86E-09	0.768
1	34798014	35364965	274	-1.93E-05	2.90E-09	0.720
1	35366056	38196841	762	6.42E-05	6.51E-09	0.426
1	38459210	39535259	436	6.92E-06	4.49E-09	0.918
1	39537291	40933221	445	2.18E-04	3.31E-09	0.000
1	40934284	42166782	487	-9.45E-05	4.33E-09	0.151
1	42168539	43365094	396	-5.62E-05	2.52E-09	0.263
1	43365565	44482312	463	-9.93E-05	2.94E-09	0.067
1	44483704	46895641	764	2.84E-05	4.07E-09	0.657

1	46897698	47999344	386	-1.41E-05	2.41E-09	0.774
1	48000156	50640662	790	2.95E-05	5.14E-09	0.681
1	50641944	53116453	445	1.85E-05	2.82E-09	0.727
1	53117066	54197141	419	-8.46E-05	3.07E-09	0.127
1	54197688	55012825	295	-2.78E-05	2.80E-09	0.600
1	55012881	55479198	250	-1.75E-05	2.43E-09	0.722
1	55479967	56413577	485	9.16E-05	3.88E-09	0.141
1	56414730	57585670	599	-1.05E-05	4.63E-09	0.878
1	57585980	58535140	555	-4.73E-05	3.85E-09	0.446
1	58536417	59306823	412	-9.34E-06	3.00E-09	0.865
1	59307290	59736866	187	8.78E-06	1.74E-09	0.833
1	59739179	61257170	537	-8.11E-05	4.13E-09	0.207
1	61258378	62107806	382	8.21E-05	4.71E-09	0.232
1	62108681	63376404	518	7.87E-05	3.98E-09	0.212
1	63379031	65008206	795	1.94E-05	6.58E-09	0.811
1	65010679	66773349	699	-2.78E-05	5.64E-09	0.711
1	66774350	67760291	537	1.54E-05	3.69E-09	0.799
1	67761365	69441414	724	-1.27E-04	6.08E-09	0.105
1	69441920	70486660	328	5.32E-05	2.61E-09	0.298
1	70490925	71818029	502	1.06E-05	3.79E-09	0.863
1	71822765	74326378	643	2.40E-05	3.95E-09	0.703
1	74326484	75992462	563	-7.85E-05	3.67E-09	0.195
1	75992778	76476360	183	-8.59E-06	1.07E-09	0.792
1	76477207	77139859	372	-2.98E-05	3.29E-09	0.603
1	77141039	79111862	686	6.51E-05	5.20E-09	0.367
1	79113455	80341320	483	-1.22E-06	3.38E-09	0.983
1	80341732	81056946	241	-1.03E-05	1.78E-09	0.807
1	81058295	82883310	839	-1.02E-04	7.21E-09	0.232
1	82884721	83990906	441	-1.82E-05	3.24E-09	0.749
1	83991748	84728665	328	-5.35E-07	2.19E-09	0.991
1	84729134	85940249	595	-3.38E-05	5.01E-09	0.633
1	85940875	86549498	270	-3.07E-07	1.86E-09	0.994
1	86549569	87652502	417	-7.69E-06	3.15E-09	0.891
1	87654606	89447978	634	-6.89E-05	5.94E-09	0.371
1	89451664	91866856	883	4.10E-06	6.73E-09	0.960
1	91867046	93351477	560	6.32E-06	3.83E-09	0.919
1	93352491	94451007	318	-2.04E-05	1.87E-09	0.637

1	94452062	95199048	406	1.57E-05	3.25E-09	0.783
1	95199771	96149941	435	3.48E-06	3.57E-09	0.954
1	96150318	97457893	467	-3.77E-05	3.09E-09	0.498
1	97458722	98562260	517	-4.77E-05	2.96E-09	0.380
1	98564736	100064599	575	8.42E-05	4.65E-09	0.217
1	100065413	101682573	614	1.05E-04	3.73E-09	0.086
1	101683165	102039858	221	-3.94E-05	1.85E-09	0.360
1	102040268	102893372	341	-5.45E-05	2.63E-09	0.288
1	102894567	104369301	288	1.63E-05	2.18E-09	0.728
1	104373157	105192916	270	-1.25E-05	2.18E-09	0.789
1	105193022	106641877	398	2.31E-05	2.91E-09	0.668
1	106642705	107432265	291	-4.86E-06	2.12E-09	0.916
1	107432576	109075191	691	5.82E-05	6.13E-09	0.458
1	109075561	110301260	464	4.58E-05	3.43E-09	0.435
1	110302505	111738116	680	-1.06E-04	5.83E-09	0.164
1	111739749	112560278	566	-8.28E-05	4.98E-09	0.241
1	112561693	114451307	681	-1.79E-05	4.16E-09	0.781
1	114451386	115878242	636	4.73E-06	4.91E-09	0.946
1	115879821	116884431	399	4.04E-05	3.14E-09	0.471
1	116884519	118837474	650	-4.51E-06	5.14E-09	0.950
1	118837888	119792751	365	-3.71E-05	2.50E-09	0.458
1	119793605	120347239	234	-4.97E-07	2.09E-09	0.991
1	144998563	147347713	454	4.39E-05	3.21E-09	0.439
1	147348339	151830844	599	-1.14E-05	8.25E-09	0.900
1	151832901	152535021	216	7.03E-05	1.19E-09	0.041
1	152535586	153163377	278	1.38E-05	1.45E-09	0.718
1	153165450	154544651	506	8.71E-06	2.96E-09	0.873
1	154547292	156334116	551	-3.50E-05	3.41E-08	0.850
1	156335111	157154186	313	6.61E-05	4.63E-09	0.332
1	157154324	158867538	820	8.46E-05	6.51E-09	0.295
1	158870085	160648549	764	4.47E-06	7.01E-09	0.957
1	160649926	161973976	510	8.19E-05	3.96E-09	0.193
1	161974584	162995355	563	-3.45E-05	4.50E-09	0.607
1	163000394	164410254	676	-8.06E-05	4.59E-09	0.234
1	164412847	165190876	436	-3.85E-05	4.49E-09	0.566
1	165191702	166363258	605	3.85E-05	5.17E-09	0.592
1	166364063	167158927	484	2.25E-05	3.45E-09	0.702

1	167159608	168168773	462	3.42E-05	4.06E-09	0.592
1	168169452	169077127	489	-2.13E-05	4.58E-09	0.753
1	169078572	169521853	193	-2.03E-05	1.12E-09	0.543
1	169522554	170372503	446	-4.14E-05	2.96E-09	0.446
1	170375663	170914261	181	-1.64E-05	1.40E-09	0.660
1	170915133	172647523	864	6.51E-05	5.55E-09	0.382
1	172648956	175384090	909	8.88E-07	5.41E-09	0.990
1	175385076	176416712	369	3.67E-05	2.58E-09	0.469
1	176418578	177431255	438	-1.91E-05	3.04E-09	0.729
1	177432237	178940717	621	2.05E-05	4.43E-09	0.758
1	178943012	179676274	338	-1.42E-05	2.04E-09	0.754
1	179678188	180567596	397	-6.02E-05	2.90E-09	0.263
1	180568720	181311800	309	5.29E-07	2.13E-09	0.991
1	181313404	182293001	421	-9.37E-06	3.28E-09	0.870
1	182294372	183796074	577	1.11E-06	3.64E-09	0.985
1	183796618	185511423	738	-7.27E-05	4.92E-09	0.300
1	185512838	187847742	807	-7.03E-06	5.43E-09	0.924
1	187848898	189756187	599	3.42E-05	3.38E-09	0.556
1	189758772	190107648	158	-1.53E-07	1.00E-09	0.996
1	190107883	191432168	473	1.26E-05	2.86E-09	0.814
1	191433917	192444256	331	-2.13E-05	2.12E-09	0.645
1	192452631	193504005	346	1.46E-05	2.11E-09	0.750
1	193505535	194000243	197	1.02E-06	1.26E-09	0.977
1	194000953	194733444	212	-2.55E-05	1.32E-09	0.482
1	194734415	195155161	176	-1.28E-05	1.08E-09	0.696
1	195155293	195995510	312	-1.56E-05	2.33E-09	0.746
1	195995948	197309603	344	3.85E-05	2.22E-09	0.414
1	197310096	198540028	417	-6.34E-05	2.64E-09	0.217
1	198540418	199578700	369	-3.70E-06	2.76E-09	0.944
1	199580285	201070422	658	-9.63E-05	4.98E-09	0.173
1	201070740	201365889	257	7.11E-06	2.19E-09	0.879
1	201366509	202820077	608	-4.42E-05	5.20E-09	0.540
1	202820815	203979519	488	5.40E-05	4.35E-09	0.413
1	203980019	205458538	791	-2.94E-04	1.33E-08	0.011
**1**	**205459511**	**206705609**	**336**	**5.00E-04**	**8.93E-09**	**0.000**
1	206706419	208158787	548	2.06E-05	4.06E-09	0.746
1	210741816	211763053	432	-8.13E-06	3.94E-09	0.897

1	208160220	210738604	1288	2.03E-05	1.07E-08	0.844
1	211763754	213578006	641	9.55E-05	5.24E-09	0.187
1	213578592	215128675	756	4.90E-05	6.40E-09	0.540
1	215129087	216672805	623	-1.35E-05	4.55E-09	0.842
1	216673326	217575665	684	-1.51E-07	5.94E-09	0.998
1	217577346	218860068	520	-1.89E-06	4.65E-09	0.978
1	218860456	220068681	530	-2.57E-05	3.63E-09	0.669
1	220069681	221856899	640	5.79E-05	4.71E-09	0.399
1	221857359	223630391	645	-1.55E-04	6.76E-09	0.060
1	223630619	224434769	280	7.51E-07	2.22E-09	0.987
1	224435934	226143348	570	-4.88E-05	4.10E-09	0.446
1	226146348	226808294	242	5.17E-06	1.96E-09	0.907
1	226808697	228100891	558	6.80E-05	3.80E-09	0.270
1	228101119	229075210	320	-2.31E-04	4.79E-09	0.001
1	229076157	230477970	634	2.49E-06	5.06E-09	0.972
1	230479324	231318678	503	2.88E-05	4.67E-09	0.673
1	231321215	232382234	476	-1.77E-05	3.60E-09	0.768
1	232384356	232949914	349	-2.00E-05	3.07E-09	0.718
1	232950888	233750725	429	1.77E-05	3.82E-09	0.775
1	233753991	234206593	224	9.95E-06	2.13E-09	0.829
1	234209087	235772624	702	2.44E-05	6.68E-09	0.765
1	235773790	236536723	313	2.71E-05	2.54E-09	0.591
1	236537770	237493854	565	-8.41E-06	4.47E-09	0.900
1	237495080	239297872	909	-2.55E-05	8.26E-09	0.779
1	239299272	240562764	672	-5.88E-06	6.16E-09	0.940
1	240563367	241178287	352	6.36E-06	4.26E-09	0.922
1	241178944	241766551	357	-4.54E-05	3.74E-09	0.458
1	241768617	242697031	497	4.26E-06	5.10E-09	0.952
1	242700803	244105053	454	6.01E-05	2.95E-09	0.269
1	244106113	244927751	382	-1.71E-05	3.94E-09	0.785
1	246606926	246989171	161	-4.09E-06	1.39E-09	0.912
1	244929262	246606531	851	1.46E-04	7.80E-09	0.098
1	246991094	247838628	369	-4.54E-05	3.38E-09	0.435
1	247838870	248585259	281	-1.16E-05	1.96E-09	0.793
2	11320	1158981	538	-5.54E-05	4.42E-09	0.405
2	1159982	1681720	220	1.11E-05	2.19E-09	0.812
2	1683187	3003071	698	5.32E-05	7.40E-09	0.536

2	3003472	4148069	589	6.18E-06	5.66E-09	0.935
2	4148784	5017172	423	-1.27E-05	3.95E-09	0.840
2	5118816	6361343	633	-4.34E-05	7.14E-09	0.607
2	6363082	7322437	569	-5.49E-05	5.57E-09	0.462
2	7323039	8980346	702	-8.99E-05	6.94E-09	0.281
2	8980472	10440987	657	-1.26E-04	7.48E-09	0.146
2	10442541	10879340	236	1.38E-04	4.44E-09	0.038
2	10881867	11652586	377	1.25E-04	6.69E-09	0.126
2	11652795	12720977	565	5.94E-06	6.64E-09	0.942
2	12723749	14648908	706	5.72E-05	5.97E-09	0.459
2	14649571	15720775	452	-1.94E-05	3.20E-09	0.732
2	15721423	16549199	386	-7.06E-05	4.57E-09	0.296
2	16549538	18344242	767	1.48E-04	6.32E-09	0.063
2	18344821	18646822	144	2.08E-06	1.23E-09	0.953
2	18647868	20059542	691	2.32E-05	6.05E-09	0.765
2	20062043	22427190	825	2.47E-05	1.13E-08	0.816
2	22428680	23855977	652	-3.32E-05	5.81E-09	0.663
2	23857184	25485735	455	1.29E-04	2.99E-09	0.018
2	25486770	26894217	438	1.04E-05	3.57E-09	0.862
2	26894589	28597305	482	1.67E-05	2.86E-09	0.755
2	28597624	29484922	455	1.48E-04	3.88E-09	0.018
2	29486078	29987735	338	-2.10E-05	2.83E-09	0.692
2	29988753	31009977	499	1.37E-05	4.49E-09	0.838
2	31010212	32489851	591	-1.75E-05	4.48E-09	0.794
2	32493210	33361425	336	1.49E-06	2.93E-09	0.978
2	33362643	34279720	532	-3.18E-05	4.88E-09	0.649
2	34280335	35607183	529	1.11E-06	4.23E-09	0.986
2	35608923	36121342	278	7.85E-05	2.12E-09	0.088
2	36122006	37004185	553	8.22E-06	5.40E-09	0.911
2	37008066	38882278	873	-1.18E-04	1.02E-08	0.244
2	38882577	40077971	314	-2.00E-05	1.99E-09	0.654
2	40080343	40625847	349	-6.91E-05	2.90E-09	0.199
2	40626336	41381901	294	-2.14E-05	2.16E-09	0.645
2	41382889	42087411	359	-3.50E-05	2.72E-09	0.501
2	42088016	42947335	404	2.77E-05	3.11E-09	0.619
2	42949616	43307776	218	6.86E-05	9.84E-09	0.489
2	43309247	44048346	326	-1.95E-04	8.90E-09	0.039

2	44049120	45194289	539	-1.66E-05	4.20E-09	0.798
2	45195022	45562814	268	-4.29E-05	3.44E-09	0.464
2	45563613	47052067	881	-2.48E-05	8.86E-09	0.792
2	47052788	47782756	370	-1.18E-04	4.25E-09	0.071
2	47782836	49112501	508	2.78E-05	4.82E-09	0.689
2	49113132	50757671	842	3.35E-05	6.82E-09	0.685
2	50758657	51667413	400	-4.91E-06	3.17E-09	0.930
2	51668142	53351493	835	6.41E-06	6.68E-09	0.937
2	53352175	54599930	554	-2.12E-05	4.62E-09	0.755
2	54600556	56158646	724	-2.98E-05	6.00E-09	0.700
2	56160647	56599354	240	-1.75E-05	2.66E-09	0.734
2	56600739	57938354	492	-3.90E-05	3.76E-09	0.524
2	57939866	60449069	1014	5.35E-05	8.72E-09	0.567
2	60449710	62420656	658	9.01E-05	4.76E-09	0.191
2	62425639	64623935	687	-1.80E-04	1.43E-08	0.132
2	64626895	65226463	256	-1.95E-05	3.52E-09	0.743
2	65228236	66374803	559	4.70E-05	4.66E-09	0.491
2	66375016	67978287	798	-8.25E-05	7.10E-09	0.327
2	67981487	68871573	395	-2.72E-05	3.13E-09	0.627
2	68871710	70753882	813	-7.15E-05	6.02E-09	0.357
2	70756057	71704491	467	-9.14E-06	4.35E-09	0.890
2	71704844	73172972	470	1.81E-05	3.89E-09	0.772
2	73174848	74121000	254	2.47E-06	1.51E-09	0.949
2	76616819	77384991	368	2.13E-05	3.13E-09	0.704
2	74121278	76615798	1069	-1.14E-05	8.98E-09	0.904
2	77386522	79007966	562	9.59E-07	4.97E-09	0.989
2	80804991	81588311	231	-2.46E-05	1.64E-09	0.544
2	79008155	80804284	1066	1.69E-05	9.79E-09	0.864
2	81588482	83604057	600	-1.72E-05	3.42E-09	0.769
2	83604796	85283713	481	2.41E-05	3.20E-09	0.671
2	85284353	86559358	572	1.40E-04	6.89E-09	0.093
2	86562902	89135764	461	-2.83E-05	3.51E-09	0.633
2	92324281	98790763	461	1.17E-05	2.93E-09	0.828
2	98791433	100195879	407	-5.62E-05	2.45E-09	0.257
2	100197638	101338509	453	3.46E-05	3.07E-09	0.532
2	101338644	102688272	529	8.42E-05	4.64E-09	0.216
2	102688495	103262415	357	2.21E-05	2.37E-09	0.649

2	103264434	104481488	294	-4.61E-06	2.25E-09	0.922
2	104485456	105871610	462	-2.16E-05	4.01E-09	0.733
2	105872982	107406122	724	-3.82E-05	7.03E-09	0.648
2	107406885	108427822	407	2.86E-05	3.14E-09	0.610
2	108427959	110109210	583	-5.92E-07	4.46E-09	0.993
2	110109222	112924907	586	1.14E-04	6.70E-09	0.164
2	112924973	114846282	718	-3.50E-05	5.48E-09	0.637
2	114848115	116770829	607	-1.65E-06	4.77E-09	0.981
2	116772945	117727045	304	-1.96E-06	1.92E-09	0.964
2	117728904	119581280	716	1.12E-05	6.02E-09	0.885
2	119581439	121070811	505	-8.39E-05	3.98E-09	0.184
2	121073253	123241656	763	1.50E-05	8.16E-09	0.868
2	123244233	124730999	532	-4.92E-06	3.82E-09	0.937
2	124733246	125735879	428	7.37E-05	3.65E-09	0.222
2	125741434	127372889	519	-1.07E-05	3.57E-09	0.858
2	127374341	129310292	757	-1.43E-05	6.43E-09	0.858
2	129312188	129864416	215	-1.67E-05	1.96E-09	0.706
2	129864730	131463038	407	2.38E-06	3.20E-09	0.966
2	131466136	133030349	226	-8.61E-06	2.04E-09	0.849
2	133034031	133950661	431	2.72E-05	4.04E-09	0.669
2	133951544	136822267	1074	-5.64E-05	7.82E-09	0.523
2	136825272	138619715	738	3.00E-05	5.87E-09	0.696
2	138620371	139412389	267	3.22E-06	1.92E-09	0.941
2	139413312	140902261	570	3.55E-05	4.65E-09	0.602
2	140902383	141803496	400	-3.54E-05	3.28E-09	0.536
2	141803602	143254981	699	8.94E-05	6.06E-09	0.251
2	143255262	145301314	618	-5.23E-05	5.44E-09	0.478
2	145303576	147252762	524	-2.57E-05	4.02E-09	0.686
2	147255216	149268931	497	-3.68E-06	3.47E-09	0.950
2	149270710	151184680	609	-8.81E-05	5.03E-09	0.214
2	151191481	152490219	540	-6.13E-05	4.30E-09	0.350
2	152491269	153369892	311	-1.28E-05	2.11E-09	0.780
2	153371042	154414456	449	1.00E-05	4.18E-09	0.876
2	154415112	155208173	297	2.79E-06	2.49E-09	0.955
2	155209736	156568446	482	1.29E-06	3.67E-09	0.983
2	156568806	158165818	441	5.93E-05	3.92E-09	0.344
2	158173710	159574883	463	-5.00E-06	4.14E-09	0.938

2	159575554	161077823	603	-3.65E-05	4.52E-09	0.587
2	161078891	163358537	678	2.16E-05	4.34E-09	0.742
2	163359084	165200324	484	-4.89E-05	4.14E-09	0.448
2	165202539	167120956	737	1.63E-04	6.54E-09	0.044
2	167123247	168427789	400	3.92E-06	2.91E-09	0.942
2	168431583	169176766	322	-7.12E-05	3.71E-09	0.242
2	170973461	172086127	479	-3.51E-05	4.44E-09	0.598
2	169178092	170973262	789	-6.10E-05	6.45E-09	0.447
2	172088943	173586201	589	1.05E-04	2.00E-08	0.458
2	173588663	175585616	784	4.87E-05	7.41E-09	0.572
2	175586510	177326682	629	3.78E-05	4.60E-09	0.577
2	177327202	178551958	558	2.27E-05	4.22E-09	0.727
2	178552788	180138313	565	-1.45E-04	5.85E-09	0.059
2	180139219	180661233	324	-7.29E-06	3.55E-09	0.903
2	180662102	181627195	413	2.91E-05	3.41E-09	0.618
2	181628139	182840114	361	-2.06E-05	2.76E-09	0.696
2	182845657	184442530	601	8.51E-05	4.50E-09	0.205
2	184443166	185277576	257	1.71E-05	1.79E-09	0.685
2	185279087	186731282	380	-2.16E-05	2.49E-09	0.666
2	186731309	188540877	478	-2.00E-05	3.02E-09	0.716
2	188542361	190794689	527	1.06E-05	3.81E-09	0.863
2	190797341	191870624	349	-2.48E-05	2.45E-09	0.616
2	191873553	192695107	283	-2.63E-05	2.83E-09	0.621
2	192696255	195109845	443	-3.52E-06	2.93E-09	0.948
2	195117932	197307918	619	2.55E-05	4.69E-09	0.710
2	197308707	198140953	269	-4.38E-05	1.76E-09	0.296
2	198141746	200498970	620	4.82E-05	3.82E-09	0.435
2	200504209	201571624	452	5.52E-05	3.34E-09	0.340
2	201572564	202829668	381	-1.12E-05	3.09E-09	0.840
2	202830132	204814896	524	-5.65E-06	3.91E-09	0.928
2	204815902	206043539	517	-1.18E-05	4.84E-09	0.865
2	206045927	207721890	728	-3.92E-05	6.24E-09	0.620
2	207722425	208639154	389	-3.18E-05	4.54E-09	0.637
2	208641174	209940909	408	-2.42E-05	3.27E-09	0.673
2	209941332	210832981	269	1.31E-06	1.84E-09	0.976
2	210838781	212377672	605	4.29E-05	4.86E-09	0.538
2	212377759	212845716	216	3.63E-05	2.29E-09	0.449

2	212845994	215005050	818	-7.57E-05	6.27E-09	0.339
2	215006557	216448007	604	-3.44E-05	4.97E-09	0.626
2	216449570	217184275	343	-4.91E-06	3.33E-09	0.932
2	217184715	218615634	620	-2.00E-05	5.49E-09	0.787
2	218616633	219671910	401	-2.10E-05	2.64E-09	0.683
2	219675067	220774229	409	4.65E-06	3.09E-09	0.933
2	220775971	221445218	359	-5.01E-05	2.91E-09	0.353
2	221445953	222985126	638	3.96E-05	5.81E-09	0.603
2	222985611	224002780	434	-5.12E-06	3.86E-09	0.934
2	224003069	225832409	736	1.45E-05	5.98E-09	0.851
2	225833758	227531180	618	-2.99E-05	4.52E-09	0.657
2	227532464	228456451	381	2.12E-05	2.95E-09	0.696
2	228457117	229368530	434	8.97E-06	3.96E-09	0.887
2	229368812	230282624	396	-2.92E-05	3.54E-09	0.624
2	230283310	231322125	471	5.94E-05	4.24E-09	0.362
2	231322831	232790491	543	1.12E-05	4.99E-09	0.874
2	232791948	233964632	468	-1.85E-05	3.83E-09	0.765
2	233968153	234699046	368	-1.97E-05	2.39E-09	0.687
2	234699366	235626316	630	2.80E-05	6.48E-09	0.728
2	235627087	236619385	515	-1.81E-05	6.09E-09	0.817
2	236619713	238209382	607	2.45E-05	5.44E-09	0.740
2	238209871	239122917	445	-1.37E-04	6.34E-09	0.086
2	239123082	239801883	289	2.71E-06	2.52E-09	0.957
2	239832097	240325991	242	2.23E-05	3.37E-09	0.701
2	240327154	240961852	336	-8.38E-05	3.80E-09	0.174
2	240962194	241907076	461	-5.68E-05	4.66E-09	0.405
2	241909927	243185680	317	6.59E-06	6.45E-09	0.935
3	60197	1128944	484	1.40E-05	5.95E-09	0.856
3	1129028	1527748	339	5.10E-05	4.48E-09	0.446
3	1528147	2423237	600	-5.04E-05	6.69E-09	0.538
3	2423999	2769230	241	-1.37E-05	3.29E-09	0.811
3	2769392	3364279	476	4.90E-05	7.08E-09	0.560
3	3364442	4430024	630	9.16E-06	6.72E-09	0.911
3	4430848	5564422	653	3.63E-05	8.57E-09	0.695
3	5565384	6421659	513	4.26E-05	6.02E-09	0.583
3	6422814	7509288	575	7.17E-05	6.11E-09	0.359
3	7509715	7991133	371	-1.09E-05	3.82E-09	0.860

3	7991981	9194649	780	-6.20E-05	8.85E-09	0.510
3	9195331	10526438	649	-2.87E-05	7.02E-09	0.732
3	10526803	11220278	388	8.36E-05	5.17E-09	0.245
3	11221721	12858028	674	1.64E-05	6.65E-09	0.841
3	12859004	13523338	333	-3.26E-05	3.97E-09	0.605
3	13524741	14309204	367	2.70E-06	4.30E-09	0.967
3	14309548	14816364	329	4.19E-05	4.16E-09	0.516
3	14816745	16661587	815	-7.56E-05	8.26E-09	0.405
3	16662488	18454062	541	-5.72E-05	5.18E-09	0.426
3	18457304	18886128	166	9.35E-06	1.57E-09	0.813
3	18886158	20752492	602	8.04E-05	6.29E-09	0.311
3	20753368	21522703	363	3.53E-05	4.39E-09	0.594
3	21523116	22102707	449	4.13E-05	5.19E-09	0.567
3	22103146	23122359	464	-3.26E-05	4.87E-09	0.641
3	23123318	23802647	259	4.58E-05	3.40E-09	0.432
3	23803541	25459497	887	-2.32E-05	9.69E-09	0.814
3	25460109	26392563	317	3.64E-06	2.88E-09	0.946
3	26393305	27606695	467	-9.00E-05	4.25E-09	0.168
3	27609447	29409547	794	-7.95E-05	9.01E-09	0.402
3	29409589	30716602	620	2.33E-05	7.16E-09	0.783
3	30717661	31720982	438	3.67E-05	3.89E-09	0.557
3	31721774	33011510	532	-4.98E-06	6.81E-09	0.952
3	33012981	33986974	341	4.10E-05	3.12E-09	0.463
3	33988115	35241960	454	2.58E-05	4.38E-09	0.697
3	35243764	36481385	482	-4.20E-05	4.49E-09	0.531
3	36484375	38078255	627	5.40E-05	5.96E-09	0.485
3	38080101	38723967	263	-1.50E-05	2.54E-09	0.766
3	38724849	40649760	701	-2.88E-05	6.21E-09	0.714
3	40660283	42184173	434	2.55E-05	3.82E-09	0.681
3	42184361	45162316	1057	3.26E-07	9.71E-09	0.997
3	46657428	48525955	410	4.60E-05	3.34E-09	0.426
3	45165430	46657162	653	6.95E-05	2.94E-08	0.685
3	48531227	52214640	855	5.13E-05	6.97E-09	0.539
3	52215002	54078568	705	3.24E-05	6.02E-09	0.676
3	54080241	55731145	920	1.62E-06	1.05E-08	0.987
3	55733390	58063819	873	-3.77E-05	7.69E-09	0.667
3	58067795	58700675	307	3.88E-05	2.79E-09	0.462

3	58700802	60347135	936	-8.95E-06	1.11E-08	0.932
3	60347701	61277928	614	2.25E-05	6.55E-09	0.781
3	61279726	62881664	855	-5.87E-06	1.04E-08	0.954
3	62882619	63504979	310	5.20E-06	3.18E-09	0.927
3	63505191	64473972	483	-3.09E-05	5.45E-09	0.675
3	64475164	65337581	550	3.41E-05	6.36E-09	0.669
3	65340365	66169323	368	-4.91E-05	4.62E-09	0.470
3	66170253	67294968	419	-1.67E-05	4.40E-09	0.801
3	67299401	69197507	757	-8.03E-05	7.38E-09	0.350
3	69205190	70288126	413	1.32E-05	4.24E-09	0.840
3	70289919	71418327	409	-2.59E-05	9.19E-09	0.787
3	71421152	73238935	813	-4.55E-05	1.01E-08	0.652
3	73239448	75376291	877	-3.65E-05	9.32E-09	0.705
3	75377075	76854466	341	1.93E-05	3.87E-09	0.756
3	76855676	77507524	288	-2.27E-05	3.03E-09	0.680
3	77508835	79022673	488	4.90E-05	4.83E-09	0.481
3	79024541	81187867	525	-1.59E-05	4.35E-09	0.809
3	81190039	82446513	356	-6.33E-06	2.79E-09	0.905
3	82446746	83946780	383	-2.03E-05	3.33E-09	0.725
3	83947451	85090935	310	2.80E-05	2.72E-09	0.592
3	85093629	86734415	435	1.16E-04	4.91E-09	0.098
3	86736368	87407921	210	-4.68E-05	7.34E-09	0.585
3	87408634	88725583	376	-1.37E-04	3.83E-09	0.027
3	88725754	94202010	445	6.98E-05	3.92E-09	0.264
3	94202771	96056259	472	-3.28E-05	3.70E-09	0.590
3	96679063	98299365	463	-1.51E-05	4.01E-09	0.811
3	98300731	100591006	762	8.94E-06	6.32E-09	0.910
3	100591848	101808006	473	1.62E-04	4.10E-09	0.011
3	101808379	103025517	322	8.94E-06	2.91E-09	0.868
3	103025715	103698105	210	3.00E-05	1.90E-09	0.492
3	103700909	104579408	324	2.40E-05	3.06E-09	0.664
3	104580063	104878270	154	4.91E-05	2.50E-09	0.325
3	104879008	106330349	517	2.49E-05	5.24E-09	0.731
3	106332831	107729547	480	1.41E-04	7.29E-09	0.098
3	107730020	108635884	340	3.85E-06	3.22E-09	0.946
3	108636466	109961316	402	4.79E-05	3.46E-09	0.416
3	109962369	111416310	404	-1.81E-05	3.52E-09	0.761

3	111416451	112182995	420	3.08E-05	3.92E-09	0.623
3	112183360	113323793	432	-8.54E-05	4.93E-09	0.224
3	113324119	115280312	582	4.88E-05	6.03E-09	0.530
3	115282058	116450487	509	-3.12E-05	5.15E-09	0.664
3	116452274	117998840	677	4.44E-05	7.57E-09	0.610
3	118000136	120520317	1012	-9.03E-05	9.42E-09	0.352
3	120521733	121335461	214	-3.14E-06	1.75E-09	0.940
3	121336367	122692039	596	1.14E-04	5.38E-09	0.119
3	122693533	123196819	219	-4.27E-05	2.13E-09	0.355
3	123200440	124677278	597	-5.13E-05	5.74E-09	0.498
3	124678230	125875831	441	-8.76E-05	4.46E-09	0.190
3	125876862	126213162	202	-2.54E-05	1.96E-09	0.566
3	126213467	127471230	536	-1.19E-05	5.27E-09	0.870
3	127471641	130243259	809	-2.33E-04	2.47E-08	0.137
3	130244120	131373405	333	-3.21E-05	2.72E-09	0.538
3	131373740	132115911	443	1.64E-05	8.33E-09	0.857
3	132117285	133128449	407	-2.98E-05	3.65E-09	0.622
3	133129272	134383242	550	1.77E-05	5.97E-09	0.819
3	137370076	138662229	384	6.29E-05	3.32E-09	0.275
3	134385789	137369181	1106	1.16E-04	9.92E-09	0.243
3	138665796	140275652	774	5.37E-05	7.86E-09	0.545
3	140276484	142863564	912	-2.29E-05	8.36E-09	0.803
3	142864473	143494546	387	6.29E-06	5.04E-09	0.929
3	143495679	145663879	729	6.95E-05	6.57E-09	0.391
3	145664135	146722271	402	5.86E-05	3.89E-09	0.347
3	146722383	148316359	475	-3.25E-05	4.88E-09	0.642
3	148316766	149988630	750	1.14E-04	8.04E-09	0.202
3	149994553	151178708	481	1.08E-05	4.99E-09	0.878
3	151178836	151904190	289	-4.14E-06	2.86E-09	0.938
3	151905386	153054901	368	-6.67E-05	3.86E-09	0.283
3	153056839	154181836	468	4.06E-05	4.91E-09	0.563
3	154183562	156005200	508	1.52E-05	5.08E-09	0.831
3	156005905	157311596	529	4.35E-05	5.00E-09	0.539
3	157312028	159272972	717	2.06E-05	6.31E-09	0.796
3	159274785	161523859	701	-4.37E-06	6.10E-09	0.955
3	161524489	162065286	249	7.58E-06	2.84E-09	0.887
3	162067622	163250087	376	1.19E-05	3.13E-09	0.832

3	163251056	164816939	494	-2.62E-05	3.86E-09	0.673
3	164820183	167116849	615	3.55E-05	5.60E-09	0.636
3	167117143	167912878	293	-3.42E-05	2.61E-09	0.503
3	167913564	170158128	714	2.93E-05	1.60E-08	0.817
3	170159134	171311936	486	-2.63E-05	5.28E-09	0.718
3	171312880	172965694	658	-3.63E-05	7.51E-09	0.675
3	172966405	174274308	524	-7.92E-05	5.16E-09	0.270
3	174275336	175049294	365	6.62E-06	4.00E-09	0.917
3	175050262	176268375	461	4.93E-05	4.68E-09	0.471
3	176269091	177530606	501	3.26E-05	5.52E-09	0.661
3	177531847	179359242	627	1.18E-04	6.21E-09	0.135
3	179366819	181426554	525	-6.31E-06	5.03E-09	0.929
3	181431571	182975975	457	5.95E-05	5.18E-09	0.409
3	182976382	183762614	363	-5.95E-05	4.03E-09	0.349
3	183762644	185262137	590	6.20E-05	6.89E-09	0.455
3	185263467	186780913	684	-1.23E-04	8.64E-09	0.185
3	186782999	187220633	227	5.66E-05	2.55E-09	0.262
3	187220777	188201380	508	1.94E-05	6.23E-09	0.806
3	188202550	189531496	585	-8.02E-05	1.01E-08	0.424
3	189531911	190303805	414	1.91E-05	4.67E-09	0.780
3	190304172	190992002	286	2.26E-05	2.88E-09	0.674
3	190993386	191840443	362	-7.34E-06	3.61E-09	0.903
3	191840983	192753870	381	-4.46E-05	3.93E-09	0.477
3	192754177	193885973	515	3.01E-05	5.83E-09	0.693
3	193886289	194464791	286	6.65E-05	3.64E-09	0.270
3	194465233	194756339	146	6.77E-05	2.20E-09	0.149
3	194756597	195854787	322	3.47E-05	3.41E-09	0.552
3	195855401	196414159	209	-5.37E-05	2.58E-09	0.290
3	196415355	197962382	528	2.85E-05	5.05E-09	0.688
4	10642	685993	149	1.66E-06	9.37E-10	0.957
4	686563	1422863	301	-6.02E-05	2.10E-09	0.189
4	1478711	3612028	639	-2.75E-05	4.99E-09	0.697
4	3613164	4263579	147	9.12E-05	2.21E-09	0.052
4	4264705	5044744	440	-3.61E-05	4.11E-09	0.573
4	5047695	5971152	586	-1.94E-05	6.08E-09	0.803
4	5971634	7135912	602	-8.55E-05	7.22E-09	0.314
4	7136158	8054472	690	-1.65E-04	8.19E-09	0.068

4	8055323	9408136	370	1.84E-05	4.77E-09	0.790
4	9408158	10696229	610	4.65E-06	3.65E-09	0.939
4	10696913	12198579	733	5.55E-05	5.62E-09	0.459
4	12201002	13002341	347	7.36E-06	2.26E-09	0.877
4	13002905	14394452	629	-8.47E-05	5.51E-09	0.254
4	14394902	15849986	606	3.65E-05	4.60E-09	0.590
4	15850685	16520118	382	2.38E-05	3.41E-09	0.684
4	16521003	18752293	860	1.60E-05	6.35E-09	0.841
4	18752722	20084049	441	7.11E-05	3.34E-09	0.218
4	20084563	21292213	550	8.44E-05	4.38E-09	0.202
4	21292651	22393873	439	-8.37E-06	3.22E-09	0.883
4	22395570	23563570	369	3.58E-05	2.96E-09	0.511
4	23563931	24992827	684	-6.16E-05	5.96E-09	0.425
4	24993167	26468997	679	7.08E-05	6.79E-09	0.390
4	26473220	27903383	509	-5.74E-06	4.28E-09	0.930
4	27904556	28871862	388	1.32E-05	2.73E-09	0.800
4	28872833	30453156	508	-1.59E-06	3.87E-09	0.980
4	30453619	31739254	482	9.53E-07	3.59E-09	0.987
4	31740009	32592949	294	-9.83E-06	2.12E-09	0.831
4	32593453	33640100	205	-4.73E-05	1.36E-09	0.199
4	33640864	35150284	380	-4.99E-06	1.93E-09	0.910
4	35151237	36301997	434	2.96E-05	3.12E-09	0.596
4	36304377	37534798	577	2.45E-06	4.85E-09	0.972
4	37536119	37886455	212	-1.05E-05	2.21E-09	0.823
4	37887735	38974458	602	3.80E-05	5.88E-09	0.620
4	38976533	40199852	438	-1.37E-05	4.03E-09	0.829
4	40200304	40869208	304	-1.79E-05	3.67E-09	0.768
4	40870402	42662283	725	-8.02E-05	5.79E-09	0.292
4	42662718	43962569	451	-2.52E-06	3.10E-09	0.964
4	43963518	45187835	414	1.61E-05	2.68E-09	0.755
4	45188855	47543891	622	-4.22E-06	4.00E-09	0.947
4	47544012	48226267	277	8.32E-06	1.71E-09	0.840
4	48227642	53412129	408	7.65E-05	1.87E-09	0.077
4	53413674	55202613	631	-3.16E-05	4.20E-09	0.626
4	55202697	57418400	883	1.09E-04	7.05E-09	0.192
4	57420107	58696072	630	1.67E-05	5.53E-09	0.822
4	58697912	59358251	234	4.43E-06	1.77E-09	0.916

4	59358729	60145179	251	-1.18E-05	1.87E-09	0.784
4	60145181	61371992	375	-4.50E-05	2.60E-09	0.377
4	61372415	61872517	167	-7.66E-06	1.21E-09	0.826
4	61872656	64117718	644	-1.62E-05	4.98E-09	0.819
4	64118963	65300308	243	1.17E-05	1.42E-09	0.756
4	65562213	66598615	369	2.77E-05	2.61E-09	0.588
4	66599449	68058793	478	-8.04E-05	3.41E-09	0.169
4	68059850	68455531	160	2.08E-05	1.09E-09	0.529
4	68456074	69954151	269	1.23E-06	1.45E-09	0.974
4	69954297	70517143	270	-5.61E-07	1.49E-09	0.988
4	70517792	71516586	418	1.78E-05	2.55E-09	0.724
4	72416258	75196731	712	1.13E-04	7.79E-09	0.202
4	75555658	76283179	293	4.49E-06	2.46E-09	0.928
4	76284248	77128724	364	1.67E-05	2.17E-09	0.720
4	77129568	78333381	607	-7.26E-05	4.64E-09	0.286
4	78333798	79469226	505	-1.28E-05	3.37E-09	0.825
4	79470550	80994124	436	2.23E-06	3.28E-09	0.969
4	80995881	81978151	261	-2.35E-06	1.75E-09	0.955
4	81979993	83012967	403	-2.06E-06	2.68E-09	0.968
4	83017789	83994716	385	-2.64E-05	2.58E-09	0.603
4	83998014	85080933	430	-1.08E-05	3.35E-09	0.852
4	85081980	87980070	901	1.24E-06	5.56E-09	0.987
4	87980748	89236855	568	9.09E-05	4.67E-09	0.183
4	89238369	90126190	323	-6.15E-05	3.46E-09	0.296
4	90126331	91453993	447	-6.66E-05	3.38E-09	0.252
4	91456722	94229509	751	2.60E-05	5.64E-09	0.729
4	94230511	95596188	496	-2.76E-04	1.91E-08	0.046
4	95596744	96181408	236	7.71E-06	1.81E-09	0.856
4	96182669	96464931	212	-2.15E-05	2.01E-09	0.632
4	96466095	97911191	514	4.54E-06	3.55E-09	0.939
4	97911459	99422081	371	4.96E-06	1.70E-09	0.904
4	99423280	100322786	399	1.35E-05	2.65E-09	0.792
4	100323029	101897750	598	9.42E-06	3.91E-09	0.880
4	101898276	103387944	552	1.91E-06	4.67E-09	0.978
4	103388441	104802530	435	-2.36E-05	2.28E-09	0.620
4	104803329	106479425	508	5.11E-04	4.63E-08	0.017
4	106482185	108468632	628	-1.24E-04	8.06E-09	0.167

4	108469756	109979131	574	-7.69E-05	5.78E-09	0.312
4	109980374	112204254	780	1.06E-04	6.26E-09	0.181
4	112204259	114902479	1024	6.56E-07	7.35E-09	0.994
4	114904730	117275534	678	1.49E-05	4.01E-09	0.814
4	117275542	118219755	308	-3.60E-05	2.23E-09	0.446
4	118220292	119945314	632	4.38E-05	4.38E-09	0.508
4	119946706	121391843	519	-2.05E-05	3.27E-09	0.720
4	121393020	121973864	244	6.59E-05	1.94E-09	0.135
4	121976226	123561869	551	4.28E-05	3.95E-09	0.496
4	123563078	125863762	773	4.64E-05	5.74E-09	0.540
4	125863974	127236805	494	-2.81E-05	4.73E-09	0.683
4	127237363	129514427	625	9.93E-06	3.77E-09	0.871
4	129514448	130364383	286	3.55E-05	2.16E-09	0.446
4	130364741	130949530	194	-2.67E-05	1.28E-09	0.456
4	130950008	131948861	379	7.10E-06	2.95E-09	0.896
4	131951466	133741365	442	-4.97E-05	2.86E-09	0.353
4	133741753	135744132	532	-1.13E-05	3.33E-09	0.845
4	135749591	137247266	391	-1.65E-05	3.14E-09	0.768
4	137247703	137987679	298	1.56E-05	2.70E-09	0.764
4	137988838	139114529	404	-8.92E-06	3.06E-09	0.872
4	139115093	139552965	239	-7.83E-06	2.55E-09	0.877
4	139553359	140546206	342	-7.60E-05	2.63E-09	0.138
4	140548713	141751418	489	-1.50E-06	7.12E-09	0.986
4	141752960	142807957	391	-6.74E-05	3.30E-09	0.241
4	142808503	145021868	627	-6.30E-05	3.38E-09	0.279
4	145024452	148047972	852	6.39E-05	5.88E-09	0.404
4	148053679	150632409	780	5.08E-05	5.30E-09	0.485
4	150634191	153226998	838	2.80E-05	4.98E-09	0.692
4	153233476	154947363	615	-2.78E-06	4.61E-09	0.967
4	154949091	156604003	633	-1.68E-05	5.32E-09	0.818
4	156604173	158112985	581	-6.27E-05	4.79E-09	0.365
4	158114105	160356254	643	5.46E-05	4.23E-09	0.401
4	160357877	160822830	147	-1.04E-05	9.13E-10	0.730
4	161056173	161796696	321	5.66E-06	2.49E-09	0.910
4	162151565	162629423	187	-8.70E-06	2.03E-09	0.847
4	162629512	163181531	216	7.42E-06	1.58E-09	0.852
4	163183341	163726178	186	-5.30E-05	1.96E-09	0.231

4	163728264	164910818	398	5.20E-05	3.57E-09	0.384
4	164913270	165817608	368	3.51E-05	2.95E-09	0.518
4	165820093	166609852	327	-4.35E-05	2.89E-09	0.418
4	166611193	167643023	297	6.44E-06	2.59E-09	0.899
4	169569516	170676328	420	4.17E-06	2.98E-09	0.939
4	167643601	169568082	733	2.79E-05	5.74E-09	0.712
4	170678993	172735897	572	-2.03E-05	3.94E-09	0.746
4	172737462	173760563	310	-3.03E-06	2.09E-09	0.947
4	173761916	174832248	301	5.30E-05	3.17E-09	0.347
4	174833988	176193705	541	-6.03E-06	5.40E-09	0.935
4	176199091	177290105	362	-7.44E-06	3.30E-09	0.897
4	177290945	177972278	282	-2.18E-05	2.21E-09	0.643
4	177974062	178758792	336	-3.00E-05	3.61E-09	0.617
4	178758930	180119509	494	4.94E-05	4.22E-09	0.447
4	180120693	181732608	834	7.33E-05	8.57E-09	0.428
4	181732746	182420390	387	3.44E-05	4.79E-09	0.619
4	182420863	184283167	1001	2.98E-05	1.14E-08	0.780
4	184283411	185477454	593	-6.00E-05	7.34E-09	0.483
4	185477964	185932497	229	-9.31E-06	1.86E-09	0.829
4	185934003	187113041	594	-5.82E-05	6.30E-09	0.464
4	187114073	188687052	716	4.80E-06	7.04E-09	0.954
4	188687155	189524815	363	5.55E-06	4.80E-09	0.936
4	189525752	190088167	270	-2.69E-05	2.74E-09	0.607
4	190088768	191043878	200	-2.03E-05	1.96E-09	0.647
5	10056	1267356	414	4.71E-05	6.83E-09	0.569
5	1270983	1762678	242	9.37E-05	9.29E-09	0.331
5	1763566	2369337	388	1.20E-04	7.27E-09	0.160
5	2369710	2979000	434	5.37E-05	3.80E-09	0.384
5	2979710	3600334	481	-4.83E-05	4.38E-09	0.465
5	3601924	4222111	306	3.39E-05	2.63E-09	0.509
5	4223577	5137474	433	5.44E-05	3.69E-09	0.370
5	5137788	5718773	375	1.19E-05	3.41E-09	0.838
5	5719162	6858229	683	-3.94E-05	5.77E-09	0.604
5	6859297	7852978	495	-5.68E-06	3.96E-09	0.928
5	7853304	9084076	627	6.26E-06	4.45E-09	0.925
5	9084701	10533767	857	-3.50E-05	7.04E-09	0.676
5	10666804	11170506	324	-1.43E-05	2.37E-09	0.769

5	11170703	13603734	759	6.83E-05	4.81E-09	0.324
5	13603883	14987807	739	-1.56E-04	5.08E-09	0.029
5	14988284	16034608	301	-5.61E-05	1.45E-09	0.141
5	16035369	17161451	494	-3.02E-05	4.31E-09	0.646
5	17162704	18048683	259	7.58E-05	2.56E-09	0.134
5	18050107	18791978	252	-4.43E-05	2.04E-09	0.326
5	18792568	19486153	258	1.58E-05	1.77E-09	0.708
5	19487359	20414139	285	3.82E-06	1.46E-09	0.920
5	20415799	21573200	239	6.35E-06	1.67E-09	0.877
5	21574347	23150924	573	-1.36E-05	3.02E-09	0.805
5	23153562	24794957	518	-3.18E-05	3.38E-09	0.585
5	24796795	26183141	453	6.38E-05	3.51E-09	0.282
5	26185302	26849079	203	3.01E-05	1.72E-09	0.469
5	26849808	28303480	392	1.35E-05	2.75E-09	0.796
5	28310093	30129786	583	1.04E-04	3.75E-09	0.088
5	30130713	31748529	717	-5.53E-06	5.83E-09	0.942
5	31748611	33944217	987	5.94E-05	8.72E-09	0.524
5	33945758	35798682	685	1.64E-05	5.13E-09	0.819
5	35799275	37888797	710	-1.95E-05	4.85E-09	0.780
5	37889231	39438373	674	3.60E-05	5.17E-09	0.617
5	39438575	40954879	626	8.27E-05	3.42E-09	0.157
5	40955561	42620153	556	-6.28E-06	2.80E-09	0.906
5	42623245	43859513	307	3.27E-05	1.35E-09	0.373
5	43862567	45286358	281	-4.55E-05	1.42E-09	0.227
5	45286977	50161698	290	-4.54E-06	1.00E-09	0.886
5	50162186	52133670	655	-8.19E-05	4.17E-09	0.205
5	52134410	52661197	301	-2.26E-05	1.97E-09	0.611
5	52662483	53513531	324	4.10E-05	2.59E-09	0.421
5	53515888	55412347	790	1.01E-06	4.75E-09	0.988
5	55413961	55932471	299	-4.34E-05	3.12E-09	0.438
5	55933701	57337744	583	-1.22E-04	5.55E-09	0.101
5	57338357	58834736	805	-3.69E-06	5.66E-09	0.961
5	58834852	60880207	583	1.30E-05	3.07E-09	0.814
5	60883078	62531300	658	9.08E-05	3.80E-09	0.141
5	62531952	63837838	373	3.74E-05	2.20E-09	0.425
5	63840208	65083148	459	4.74E-06	2.54E-09	0.925
5	65083209	65909616	334	-2.75E-05	2.66E-09	0.594

5	65910316	67096208	520	2.20E-05	3.61E-09	0.714
5	67096780	70682458	689	-3.43E-05	5.23E-09	0.635
5	70683839	72420748	629	-5.22E-05	3.31E-09	0.364
5	72420784	73507955	557	-3.29E-05	4.35E-09	0.617
5	73508509	75240469	728	-1.17E-04	4.70E-09	0.088
5	75242395	75740199	233	1.80E-06	1.75E-09	0.966
5	75743532	77203135	738	1.16E-04	5.80E-09	0.129
5	77203718	79068089	684	-9.78E-06	4.62E-09	0.886
5	79068300	80480761	705	1.94E-05	5.70E-09	0.797
5	80481996	81712715	433	-5.57E-05	2.96E-09	0.306
5	81714078	82658571	362	-4.06E-05	2.81E-09	0.445
5	82660901	84397685	586	-1.35E-05	5.17E-09	0.851
5	84399219	85315500	310	2.33E-05	1.69E-09	0.571
5	85316429	86490293	328	2.98E-05	2.32E-09	0.536
5	86492986	88065637	305	-9.21E-06	1.54E-09	0.814
5	88069593	90500398	713	1.16E-06	4.77E-09	0.987
5	90504842	93602613	719	-3.86E-05	4.05E-09	0.545
5	93607443	95822590	794	5.11E-05	5.43E-09	0.488
5	95822732	96867936	466	-7.18E-05	3.01E-09	0.191
5	96870861	97435808	143	-2.23E-06	9.51E-10	0.942
5	97436504	99258325	558	-4.32E-05	3.30E-09	0.452
5	99259751	101101103	482	-3.53E-05	2.51E-09	0.480
5	101101325	102681586	443	5.38E-06	2.02E-09	0.905
5	102686157	104542488	700	1.50E-05	5.30E-09	0.837
5	104543131	105885913	312	1.41E-05	2.00E-09	0.752
5	105887333	106800282	343	-2.95E-05	2.60E-09	0.563
5	106800765	108633176	758	2.02E-05	5.89E-09	0.793
5	108633347	109231597	246	-5.15E-06	1.29E-09	0.886
5	109232816	110819753	510	5.35E-07	3.24E-09	0.993
5	110821786	111962676	471	-4.89E-05	3.76E-09	0.425
5	111963025	112396750	156	5.75E-06	8.41E-10	0.843
5	112397011	113603768	561	2.87E-07	3.57E-09	0.996
5	113604579	114448326	333	-1.08E-06	2.21E-09	0.982
5	114448597	115828291	681	-6.26E-05	4.71E-09	0.362
5	115829425	117003674	543	-6.27E-05	4.16E-09	0.331
5	117004531	117712427	268	1.65E-05	1.93E-09	0.707
5	117714517	119668423	688	-2.64E-05	4.86E-09	0.705

5	119669457	120962383	495	-1.56E-05	3.68E-09	0.797
5	120963248	121481183	148	2.58E-05	9.81E-10	0.410
5	121485609	122603725	451	-8.71E-08	2.96E-09	0.999
5	122604501	123236124	257	4.14E-06	1.67E-09	0.919
5	123238611	123797013	229	2.66E-05	1.78E-09	0.529
5	123798436	124746813	465	2.17E-05	4.23E-09	0.738
5	124748624	126938920	874	-4.72E-05	6.24E-09	0.550
5	126940058	128727903	666	-5.23E-05	4.04E-09	0.411
5	128730275	130376511	412	-9.63E-07	1.81E-09	0.982
5	130376936	131873073	450	9.82E-06	2.32E-09	0.838
5	131874022	132603137	224	2.46E-05	1.35E-09	0.503
5	132609517	134321546	718	-1.59E-04	5.74E-09	0.035
5	134329463	135339464	501	-1.28E-05	3.50E-09	0.829
5	135340951	137948424	906	9.30E-06	4.87E-09	0.894
5	140644957	141640050	475	-2.41E-05	3.93E-09	0.701
5	137950183	140644484	766	-1.13E-05	3.40E-09	0.847
5	141640419	142230251	276	-2.11E-08	2.64E-09	1.000
5	142230949	144583138	963	6.67E-05	5.83E-09	0.383
5	144584265	145803004	431	-6.24E-06	2.19E-09	0.894
5	145803849	147651230	762	-3.14E-05	5.30E-09	0.666
5	150395351	150917796	368	-4.32E-05	2.65E-09	0.402
5	147665223	150393107	1253	-3.12E-07	8.32E-09	0.997
5	150918158	152360494	594	8.69E-05	3.73E-09	0.155
5	152361546	152864614	177	2.29E-06	1.05E-09	0.944
5	152867348	153494463	316	2.02E-05	1.59E-09	0.613
5	153495193	154448863	413	-1.72E-05	3.11E-09	0.757
5	154449856	155290211	358	3.70E-05	2.33E-09	0.444
5	155290613	156628287	483	-9.31E-06	3.31E-09	0.871
5	156629754	157197141	306	-6.07E-05	1.99E-09	0.173
5	157199547	158491655	551	6.25E-05	3.48E-09	0.289
5	158492279	159135376	279	6.06E-05	2.23E-09	0.200
5	159136348	160908680	774	2.26E-05	5.88E-09	0.768
5	160910101	161688620	224	-6.23E-06	1.45E-09	0.870
5	161688913	162741298	358	5.63E-05	2.66E-09	0.275
5	162742614	163769136	431	6.72E-05	3.21E-09	0.236
5	163769738	164944949	399	6.46E-05	2.73E-09	0.217
5	164947504	165638940	255	4.51E-06	2.79E-09	0.932

5	165642395	166432731	347	-6.89E-06	3.24E-09	0.904
5	166433367	167760730	549	-3.78E-06	4.62E-09	0.956
5	167761922	169124478	718	5.53E-05	6.25E-09	0.484
5	169125063	170889081	953	-6.87E-06	7.25E-09	0.936
5	170891834	171772889	429	-3.07E-05	3.70E-09	0.614
5	171775715	172376903	328	-1.28E-05	3.24E-09	0.822
5	172377031	173611384	634	-1.52E-04	7.20E-09	0.074
5	173613085	174468408	481	7.63E-05	5.84E-09	0.318
5	174468624	176128648	690	6.28E-05	6.36E-09	0.431
5	176129392	177991305	617	-7.24E-05	7.46E-09	0.402
5	177992368	178410668	199	6.94E-05	1.83E-09	0.105
5	178410839	179245749	390	-9.93E-05	3.47E-09	0.092
5	179245856	180007693	315	3.47E-05	2.79E-09	0.511
5	180008456	180790950	242	-8.98E-06	2.59E-09	0.860
6	63979	824529	331	-9.64E-06	4.05E-09	0.880
6	826312	1582647	523	-3.47E-05	6.41E-09	0.664
6	1582926	2890836	717	-1.04E-05	7.07E-09	0.901
6	2891016	4315210	770	-1.49E-04	7.96E-09	0.095
6	4317398	5056146	426	7.91E-05	3.92E-09	0.206
6	5057399	6246639	735	6.52E-05	7.29E-09	0.445
6	6246984	7328580	685	6.77E-05	8.43E-09	0.461
6	7329417	8791321	740	3.50E-05	6.06E-09	0.653
6	8791421	9760323	373	2.75E-05	3.57E-09	0.645
6	9764413	11234164	726	-8.02E-06	7.40E-09	0.926
6	11235682	12183952	544	-2.74E-06	4.83E-09	0.969
6	12186102	13388480	617	-5.10E-05	5.21E-09	0.480
6	13389521	14618107	554	1.11E-04	4.98E-09	0.115
6	14619148	15998368	596	-1.29E-05	5.44E-09	0.861
6	15998406	17067160	545	6.14E-05	6.44E-09	0.444
6	17069966	19203108	879	2.90E-05	7.31E-09	0.734
6	19203782	20228741	524	-6.82E-06	5.66E-09	0.928
6	20231516	22049758	824	3.90E-05	9.87E-09	0.695
6	22052425	23495209	747	-2.21E-04	1.03E-08	0.029
6	23495260	23935020	233	3.00E-05	2.04E-09	0.506
6	23935352	24799440	438	-7.62E-06	4.75E-09	0.912
6	24803038	25483400	411	-9.46E-06	3.82E-09	0.878
6	25484295	26774948	730	-1.05E-04	1.03E-08	0.301

6	26778827	28669698	609	-1.09E-04	6.48E-09	0.176
6	32900018	33863002	401	-1.12E-04	8.94E-09	0.236
6	33863662	35451187	514	1.63E-04	3.84E-09	0.009
6	35452346	36608267	395	3.25E-05	3.13E-09	0.561
6	36611164	38686222	997	9.07E-05	9.28E-09	0.347
6	38687185	39021712	205	-3.86E-05	2.93E-09	0.476
6	39022698	40344526	675	-4.42E-05	5.95E-09	0.567
6	40345542	42105449	886	-1.95E-04	8.75E-09	0.037
6	42105555	43957473	633	7.54E-05	6.45E-09	0.348
6	43959116	45787468	701	-9.69E-06	6.57E-09	0.905
6	45788111	46556325	327	-3.49E-05	2.55E-09	0.490
6	46560003	47885216	681	-5.85E-05	5.21E-09	0.418
6	47887063	49494241	468	2.06E-05	3.11E-09	0.712
6	49494933	50406476	309	-2.52E-05	2.53E-09	0.617
6	50406575	51703012	476	-1.41E-05	3.73E-09	0.818
6	51704668	53402679	834	-3.66E-05	7.42E-09	0.671
6	53405127	54291407	455	-1.38E-06	3.43E-09	0.981
6	54293261	56103747	744	3.73E-05	5.56E-09	0.617
6	56104522	57082026	352	-8.83E-06	2.47E-09	0.859
6	57085542	58084365	151	2.42E-06	1.17E-09	0.944
6	58139505	62691733	306	1.23E-05	2.03E-09	0.784
6	62692409	63712779	264	9.98E-07	1.61E-09	0.980
6	63713620	65760585	508	-3.36E-05	3.16E-09	0.551
6	65762553	67028107	493	-2.50E-05	3.70E-09	0.681
6	67029501	67556729	242	6.75E-06	2.10E-09	0.883
6	67557245	68546601	377	4.86E-05	3.05E-09	0.379
6	68546995	69295414	301	5.50E-06	3.03E-09	0.920
6	69295526	70359936	399	-8.19E-06	2.94E-09	0.880
6	70360869	72448598	882	5.41E-05	6.99E-09	0.518
6	72449421	73170712	296	2.30E-05	2.51E-09	0.646
6	73171594	75064563	683	6.92E-05	5.50E-09	0.351
6	75065279	75843360	172	-7.72E-06	1.49E-09	0.841
6	75843845	77257094	415	-1.23E-05	2.74E-09	0.815
6	77257275	78957547	680	-4.88E-06	4.93E-09	0.945
6	78958134	79861169	296	1.28E-05	2.09E-09	0.780
6	79862483	80556359	340	1.99E-05	3.10E-09	0.721
6	80557170	82387384	660	-2.81E-05	4.51E-09	0.675

6	82387920	83904869	519	7.24E-07	4.17E-09	0.991
6	83908232	86040334	810	2.65E-05	6.36E-09	0.740
6	86040497	87138115	340	-2.15E-06	2.40E-09	0.965
6	87140364	88532072	486	-1.05E-04	3.28E-09	0.068
6	88533324	89668547	467	5.50E-05	4.75E-09	0.425
6	89673861	91400644	815	9.14E-05	7.41E-09	0.289
6	91401234	93426186	759	-1.54E-05	6.81E-09	0.852
6	93429797	94919277	573	8.18E-05	4.88E-09	0.242
6	94920611	97093037	660	3.99E-05	5.32E-09	0.585
6	97093295	98893182	513	-1.79E-05	3.91E-09	0.774
6	98893830	100286683	584	-1.82E-05	4.93E-09	0.796
6	100287245	101862923	465	-9.21E-06	3.52E-09	0.877
6	101863563	102887537	348	-1.74E-05	3.16E-09	0.757
6	102888281	104211089	445	-4.13E-05	3.18E-09	0.464
6	104211475	105752435	548	-4.44E-05	4.30E-09	0.498
6	105754306	106921804	535	-7.60E-06	5.87E-09	0.921
6	106922281	108322638	655	-1.73E-05	6.44E-09	0.830
6	108325624	110654066	761	-9.19E-05	7.76E-09	0.297
6	110654605	111390344	274	2.71E-05	2.51E-09	0.589
6	111391537	114218733	1067	6.90E-05	8.55E-09	0.456
6	114220361	116130955	594	-3.85E-05	4.70E-09	0.574
6	116130983	117566682	496	1.12E-05	4.53E-09	0.868
6	117567468	119071713	561	2.09E-05	4.45E-09	0.755
6	119075856	120628075	540	-1.16E-05	4.50E-09	0.863
6	120628214	121673627	339	-1.93E-06	2.36E-09	0.968
6	121674337	123104703	522	-5.73E-08	3.93E-09	0.999
6	123105676	123889346	320	1.42E-05	3.04E-09	0.796
6	123890282	125333797	657	-1.95E-05	6.40E-09	0.807
6	125336799	128759251	900	1.09E-05	6.97E-09	0.897
6	128759755	130538497	787	1.18E-05	7.24E-09	0.890
6	130540383	131972731	564	9.30E-05	4.76E-09	0.178
6	131973932	133867802	873	5.60E-05	8.23E-09	0.537
6	136186368	137811295	508	1.42E-04	4.27E-09	0.030
6	133869809	136186007	802	1.41E-05	7.33E-09	0.869
6	137813095	139532424	748	8.27E-05	7.58E-09	0.342
6	139533572	142036032	708	-8.26E-05	5.77E-09	0.277
6	142036912	143719278	602	-3.28E-05	4.98E-09	0.643

6	143720467	146663272	1047	-3.66E-06	8.37E-09	0.968
6	146665424	148179283	592	-3.49E-05	4.87E-09	0.617
6	148180191	148917941	480	-9.34E-05	5.86E-09	0.222
6	148919158	150298842	639	4.10E-06	4.91E-09	0.953
6	150299299	151097980	538	-1.21E-04	6.19E-09	0.124
6	151100484	151929942	491	4.91E-05	5.59E-09	0.512
6	151930249	153550147	880	-1.60E-04	1.05E-08	0.119
6	153551177	155228511	745	-2.85E-05	7.48E-09	0.741
6	156761338	157398853	256	-2.98E-05	2.34E-09	0.538
6	155229566	156756457	768	4.75E-05	7.96E-09	0.594
6	157402041	158192951	273	1.96E-05	2.97E-09	0.719
6	158193244	159318774	547	-4.71E-05	4.16E-09	0.466
6	159319812	159792458	251	-2.22E-05	1.70E-09	0.591
6	159793334	161123451	690	-2.57E-04	2.75E-08	0.120
6	161123771	162298351	679	-5.48E-05	7.06E-09	0.515
6	162300151	163277414	510	7.25E-06	4.49E-09	0.914
6	163277858	164003180	378	6.91E-05	3.75E-09	0.259
6	164006012	165242243	674	1.15E-05	6.25E-09	0.885
6	165246062	166191910	572	1.77E-05	5.82E-09	0.817
6	166193932	167177733	678	7.55E-06	7.08E-09	0.929
6	167178790	168548525	628	3.18E-05	5.47E-09	0.667
6	168549335	169231503	390	-1.76E-06	3.96E-09	0.978
6	169231937	171049558	874	-4.71E-05	7.45E-09	0.586
7	20608	1490775	418	4.39E-06	3.97E-09	0.945
7	1493090	2479029	317	-1.22E-04	5.17E-09	0.090
7	2480729	2930941	207	3.11E-05	1.88E-09	0.473
7	2931676	3736942	499	8.53E-05	4.59E-09	0.208
7	3738087	4783224	512	-3.55E-05	4.72E-09	0.606
7	4783723	5412932	213	2.58E-05	2.62E-09	0.614
7	5414602	6912527	400	1.51E-04	3.93E-09	0.016
7	7168493	7679885	293	-8.49E-05	2.61E-09	0.097
7	7680697	8475147	543	4.61E-05	5.99E-09	0.551
7	8476787	9223721	526	-4.57E-05	5.07E-09	0.521
7	9223762	9893744	341	4.80E-05	3.49E-09	0.417
7	9894421	11298815	623	-1.55E-05	5.16E-09	0.830
7	11299552	12687871	879	-4.37E-06	8.11E-09	0.961
7	12689322	13674908	507	3.34E-06	4.55E-09	0.960

7	13675094	14941781	684	3.76E-05	6.54E-09	0.642
7	14942134	16480989	759	8.33E-05	6.35E-09	0.296
7	16481863	17277084	484	-5.10E-05	5.44E-09	0.489
7	17279294	18861958	710	-3.22E-05	6.31E-09	0.685
7	18863387	19975992	510	-1.12E-04	4.09E-09	0.080
7	19978609	21712233	879	2.41E-05	8.75E-09	0.797
7	21712615	22811384	708	8.91E-05	7.30E-09	0.297
7	22811921	24389035	702	7.66E-05	5.68E-09	0.310
7	24389147	24980408	323	1.24E-05	3.04E-09	0.822
7	24982494	26300992	674	-7.88E-05	7.87E-09	0.374
7	26302668	28038375	768	2.65E-04	3.67E-08	0.166
7	28039590	28710596	382	-6.73E-05	6.40E-09	0.400
7	28710857	30171413	827	-1.43E-05	7.56E-09	0.869
7	30172195	31608495	659	-5.50E-05	5.98E-09	0.477
7	31609317	33150311	750	-3.28E-05	5.98E-09	0.672
7	34940039	35614036	301	-3.09E-05	2.35E-09	0.523
7	33151181	34939884	766	9.45E-05	6.25E-09	0.232
7	35615611	37353302	858	3.24E-05	7.24E-09	0.703
7	37354091	38159855	466	-3.93E-05	4.04E-09	0.537
7	38160558	39534540	677	-3.24E-05	6.04E-09	0.677
7	39535570	40925323	373	-2.86E-06	2.63E-09	0.955
7	40926906	42494824	654	2.61E-05	5.81E-09	0.732
7	42495555	44971837	906	1.32E-05	6.27E-09	0.868
7	44972152	46665658	694	3.55E-05	5.48E-09	0.632
7	46667873	47279128	336	-2.76E-06	2.74E-09	0.958
7	47281095	49130572	862	1.24E-04	7.56E-09	0.155
7	49131824	49821750	300	-8.12E-06	2.27E-09	0.865
7	49822437	51674880	774	-1.90E-05	5.70E-09	0.801
7	51675218	52274523	293	-7.23E-06	2.83E-09	0.892
7	52275656	53175065	478	2.10E-05	3.93E-09	0.738
7	53348777	54707110	519	9.34E-06	3.75E-09	0.879
7	54707337	55641707	451	-5.16E-05	3.88E-09	0.408
7	55641798	57382254	387	1.21E-05	2.92E-09	0.823
7	57382271	62556935	174	-5.92E-06	1.01E-09	0.852
7	62557202	64193303	281	9.23E-06	1.87E-09	0.831
7	64699988	65570171	152	4.93E-06	8.12E-10	0.863
7	65570310	66453460	219	1.46E-05	8.54E-10	0.616

7	66454071	67369810	339	-4.85E-05	2.83E-09	0.362
7	67370861	68125403	317	2.08E-06	4.19E-09	0.974
7	68126378	68561421	188	-1.33E-05	1.57E-09	0.736
7	68562932	69806895	289	-1.28E-05	1.72E-09	0.757
7	69808153	71204524	594	-1.23E-05	6.27E-09	0.877
7	71205507	71873571	249	-1.58E-05	1.81E-09	0.710
7	71874997	73996533	396	2.84E-05	2.91E-09	0.599
**7**	**73997700**	**76550299**	**361**	**-2.68E-04**	**3.81E-09**	**0.000**
7	76551983	77101532	181	-5.00E-06	1.94E-09	0.910
7	77102731	77953248	411	-3.16E-06	3.14E-09	0.955
7	77954163	78839317	553	-8.90E-05	5.09E-09	0.213
7	78843289	80546745	614	1.27E-06	4.60E-09	0.985
7	80547047	82758529	882	-7.67E-05	8.22E-09	0.397
7	82758644	83959105	533	6.17E-06	4.28E-09	0.925
7	83959751	85570789	448	-3.15E-05	2.85E-09	0.556
7	85571372	86341446	218	-3.73E-05	1.42E-09	0.321
7	88732470	89270556	217	-3.57E-05	1.68E-09	0.384
7	86345013	88732142	836	2.96E-05	5.47E-09	0.689
7	89271899	91173749	774	4.40E-05	5.81E-09	0.564
7	91174778	93339775	617	9.10E-05	4.07E-09	0.154
7	93340137	95171731	630	2.33E-05	5.28E-09	0.748
7	95419256	96611864	459	-1.43E-05	3.40E-09	0.806
**7**	**96613246**	**98151455**	**496**	**-4.00E-04**	**8.19E-09**	**0.000**
7	98152191	100555361	665	6.45E-05	5.06E-09	0.364
7	100606141	101730309	387	-4.55E-05	4.72E-09	0.507
7	101731567	103193331	317	1.10E-05	2.37E-09	0.821
7	103193651	104157576	550	-8.94E-06	4.58E-09	0.895
7	104158491	105425027	481	-1.62E-05	3.26E-09	0.777
7	105427565	106052506	320	8.31E-06	3.40E-09	0.887
7	106052873	107305328	456	-1.97E-05	3.62E-09	0.744
7	107306421	108375939	543	-7.89E-05	3.91E-09	0.208
7	108376173	109584769	351	-8.72E-06	2.52E-09	0.862
7	109587777	111447153	593	-8.07E-05	4.21E-09	0.214
7	111450919	113336970	590	-1.40E-06	4.86E-09	0.984
7	113337327	116315205	824	-1.85E-05	5.90E-09	0.809
7	116317043	118347634	570	1.85E-05	3.63E-09	0.759
7	118348359	121094386	573	1.22E-05	3.43E-09	0.836

7	121094460	122368663	412	2.16E-05	3.36E-09	0.709
7	122369853	124154451	639	-3.95E-05	4.10E-09	0.537
7	124155319	125386718	379	4.26E-05	2.26E-09	0.371
7	125387168	126516683	442	-1.09E-05	2.98E-09	0.842
7	126517818	127905983	588	-7.85E-06	3.73E-09	0.898
7	127907054	128554075	209	-1.13E-05	1.77E-09	0.789
7	128555262	130074230	546	1.35E-05	4.27E-09	0.836
7	130075370	130797123	249	8.15E-07	2.65E-09	0.987
7	130797776	131856855	498	5.28E-05	4.85E-09	0.448
7	131857419	134306672	895	1.09E-04	6.24E-09	0.168
7	134307114	135464701	504	2.57E-05	4.01E-09	0.685
7	135466022	137137511	701	1.19E-05	5.74E-09	0.876
7	137138736	138742981	587	1.59E-05	6.38E-09	0.842
7	138744100	141106552	845	-7.48E-05	6.78E-09	0.364
7	141109797	142655113	557	6.28E-05	3.80E-09	0.308
7	142655717	144274735	430	-4.76E-06	3.16E-09	0.932
7	144276424	146018520	552	-1.55E-05	4.35E-09	0.814
7	146023023	147204086	503	5.03E-05	3.82E-09	0.415
7	147204549	148619685	685	1.16E-04	5.70E-09	0.124
7	148620581	150491084	655	2.17E-06	5.26E-09	0.976
7	150491851	151390799	462	-6.55E-05	4.74E-09	0.342
7	151391044	152409155	288	-5.88E-06	4.63E-09	0.931
7	152410248	153080056	288	1.73E-05	4.00E-09	0.785
7	153081698	153934166	320	2.13E-06	3.16E-09	0.970
7	153934210	154963787	621	2.16E-04	5.99E-09	0.005
7	154964316	155379409	239	-6.08E-05	3.75E-09	0.320
7	155380353	156814410	652	-4.95E-05	6.56E-09	0.541
7	156815867	158029340	505	4.97E-06	4.93E-09	0.944
7	158030463	158759939	286	-7.95E-06	2.05E-09	0.861
7	158760677	159127587	134	-2.31E-05	1.46E-09	0.544
8	246934	1162157	489	-8.01E-06	5.74E-09	0.916
8	1162454	1625424	273	9.45E-06	3.26E-09	0.869
8	1625732	2042364	303	4.34E-05	5.10E-09	0.543
8	2043187	2680255	318	3.40E-05	4.22E-09	0.601
8	2681051	3358423	619	-2.11E-05	7.49E-09	0.807
8	3358433	3584832	297	-2.60E-05	3.24E-09	0.649
8	3585058	4071438	628	-4.79E-05	6.70E-09	0.559

8	4071516	4274957	255	7.56E-06	2.80E-09	0.886
8	4275286	4479596	220	2.02E-06	3.25E-09	0.972
8	4479836	4754328	302	2.86E-05	3.13E-09	0.609
8	4755088	5146808	311	-1.91E-05	3.37E-09	0.742
8	5147734	5781592	597	3.36E-05	6.29E-09	0.672
8	5781947	6017670	226	-4.90E-05	2.11E-09	0.286
8	6018292	6350873	272	-1.98E-05	2.59E-09	0.697
8	6352358	7793703	489	-3.96E-05	5.46E-09	0.592
8	7793837	8735213	412	-7.19E-06	3.83E-09	0.907
8	8736386	10117293	800	-3.80E-05	7.79E-09	0.667
8	10117411	10535826	286	-1.99E-05	3.34E-09	0.730
8	10537940	11249261	410	-3.56E-05	3.51E-09	0.547
8	11250848	11836622	364	-6.07E-05	4.84E-09	0.383
8	11838355	12975295	284	-9.40E-06	3.07E-09	0.865
8	12975578	13940652	686	-3.41E-05	6.86E-09	0.680
8	13942500	14876660	523	-9.24E-05	4.81E-09	0.183
8	14877288	15273777	321	4.03E-06	2.78E-09	0.939
8	15274608	16087631	534	-4.22E-05	5.44E-09	0.567
8	16087729	17579518	803	-1.23E-08	8.50E-09	1.000
8	17581106	18670645	722	-6.35E-05	6.70E-09	0.438
8	18670908	19490180	560	-2.79E-05	5.91E-09	0.717
8	21022075	21402658	257	4.49E-06	2.49E-09	0.928
8	19491428	21021401	888	1.27E-04	9.59E-09	0.196
8	21404381	22539316	482	1.64E-05	4.14E-09	0.798
8	22539641	23548146	588	-7.95E-05	1.58E-08	0.528
8	23548749	24456913	317	2.21E-05	2.83E-09	0.678
8	24457122	25461315	424	-9.59E-06	3.37E-09	0.869
8	25461344	26681450	716	-2.33E-05	7.35E-09	0.786
8	26681799	27404354	421	-1.54E-05	3.59E-09	0.797
8	27405207	27759126	214	-4.93E-05	1.65E-09	0.225
8	27759839	29277590	622	-6.64E-05	5.56E-09	0.373
8	29277652	31947675	956	2.05E-06	8.67E-09	0.982
8	31947880	32448740	282	-2.13E-05	2.69E-09	0.681
8	32449533	33980486	355	6.67E-06	2.61E-09	0.896
8	33981742	35313077	399	4.88E-05	2.57E-09	0.336
8	35314837	37375458	435	-1.02E-06	3.40E-09	0.986
8	37376914	39779989	596	-6.07E-05	5.56E-09	0.416

8	39781444	40415799	340	8.77E-06	3.28E-09	0.878
8	40417433	41718356	592	-1.62E-05	5.82E-09	0.831
8	41719750	42501174	186	2.39E-05	1.47E-09	0.532
8	42501717	49028667	390	7.72E-06	2.27E-09	0.871
8	49038732	52156842	816	3.98E-05	5.37E-09	0.587
8	52157415	54115712	718	-3.73E-05	5.68E-09	0.620
8	54116775	55914038	659	5.25E-05	6.25E-09	0.507
8	55914314	57653028	655	-2.03E-05	5.77E-09	0.789
8	57655067	58674153	403	-5.90E-05	3.30E-09	0.305
8	58675131	59568410	383	1.87E-05	3.61E-09	0.756
8	59570176	61809929	767	-1.13E-04	6.53E-09	0.162
8	61810694	63710298	771	-8.74E-05	6.47E-09	0.277
8	63711348	65193342	460	-2.09E-05	3.81E-09	0.735
8	65195953	66024947	260	-6.53E-06	2.12E-09	0.887
8	66025862	67192642	387	-3.52E-05	3.07E-09	0.525
8	67193708	69108839	502	-4.78E-05	4.45E-09	0.473
8	69111244	70003448	401	4.82E-05	4.13E-09	0.453
8	70003607	72013005	727	2.69E-05	6.55E-09	0.739
8	72014069	72749564	427	-3.12E-05	4.68E-09	0.649
8	72751469	73423214	352	-5.67E-05	3.49E-09	0.337
8	73423786	74996061	687	-9.56E-07	6.15E-09	0.990
8	74998868	76294909	508	7.43E-05	4.47E-09	0.266
8	76295292	78015335	544	-2.16E-05	4.39E-09	0.744
8	78015611	78632215	197	1.11E-05	1.50E-09	0.775
8	78632893	79831888	374	-1.94E-05	2.63E-09	0.705
8	79832211	80654655	273	1.33E-06	2.48E-09	0.979
8	80656206	81330540	223	1.87E-05	1.92E-09	0.670
8	81331077	83726996	809	4.30E-05	7.17E-09	0.612
8	83727741	85093121	385	2.47E-05	3.35E-09	0.669
8	85094979	86979097	352	-8.24E-06	2.40E-09	0.866
8	86990451	87562786	212	8.07E-06	1.45E-09	0.832
8	87563878	89083319	540	-3.36E-05	4.49E-09	0.616
8	89083979	90605445	456	2.47E-05	3.39E-09	0.672
8	90607207	92342283	523	2.29E-05	3.59E-09	0.702
8	92344138	94269630	544	-2.46E-05	5.05E-09	0.729
8	94272436	95810190	578	-6.15E-05	4.91E-09	0.380
8	95810772	96533604	390	1.19E-05	3.79E-09	0.847

8	96534326	97318660	396	5.07E-05	3.95E-09	0.420
8	97320605	98867870	622	1.71E-05	5.34E-09	0.815
8	98869940	101450242	829	3.43E-05	6.22E-09	0.663
8	101451169	102937063	656	2.77E-05	6.60E-09	0.733
8	102944509	103730879	375	8.14E-06	4.15E-09	0.899
8	103970185	105389708	566	7.45E-06	4.43E-09	0.911
8	105391381	107409808	782	4.22E-05	6.80E-09	0.609
8	107410067	108108114	325	2.57E-05	2.68E-09	0.619
8	108115161	108646193	244	3.77E-06	2.41E-09	0.939
8	108646968	110761074	744	-5.11E-05	6.98E-09	0.541
8	110761776	112611637	402	1.93E-05	2.98E-09	0.723
8	112612842	114393054	481	-6.30E-06	3.16E-09	0.911
8	114394256	116095059	617	-9.31E-07	4.58E-09	0.989
8	116095815	117130004	272	9.10E-05	6.19E-09	0.247
8	117131947	118622352	563	2.38E-05	5.16E-09	0.741
8	118623844	120216957	719	4.96E-05	6.70E-09	0.545
8	120217356	121767384	601	1.56E-05	4.56E-09	0.817
8	121770585	123545992	691	3.41E-05	6.63E-09	0.675
8	123547148	124483938	403	6.59E-05	4.19E-09	0.309
8	124486753	125247268	404	1.31E-05	4.19E-09	0.840
8	125248576	126268046	461	-5.75E-05	4.64E-09	0.398
8	126269921	127434945	507	9.07E-06	7.53E-09	0.917
8	127436003	128162974	338	1.18E-04	6.06E-08	0.633
8	128166556	128542444	193	-1.54E-04	5.83E-08	0.524
8	128543956	129297518	432	-1.43E-04	9.87E-09	0.150
8	129297807	130379330	412	1.50E-05	3.53E-09	0.800
8	130380171	130812339	231	5.15E-06	2.23E-09	0.913
8	130814803	131553214	284	-3.37E-05	2.41E-09	0.492
8	131553327	132358990	430	5.09E-05	4.07E-09	0.425
8	132359241	133454722	553	-8.22E-07	4.76E-09	0.990
8	133455530	134566686	734	5.79E-05	6.82E-09	0.483
8	134567348	135269827	423	2.20E-05	4.76E-09	0.750
8	135270868	136209239	515	1.04E-05	4.98E-09	0.883
8	136209325	137306730	483	-1.49E-05	3.64E-09	0.805
8	137307361	138223632	346	-1.80E-05	2.81E-09	0.734
8	138224181	139194661	572	9.04E-05	4.98E-09	0.200
8	139195331	140093896	520	-9.83E-06	5.68E-09	0.896

8	140094627	141355502	731	3.95E-05	7.09E-09	0.639
8	143750619	146303789	716	-4.67E-05	5.80E-09	0.540
8	141358681	143750205	923	-3.02E-05	9.14E-09	0.752
9	342841	1006606	533	3.33E-05	4.78E-09	0.630
9	1007923	1792147	679	1.16E-05	5.65E-09	0.878
9	1792277	2665240	686	-3.24E-05	7.07E-09	0.700
9	2666964	3348816	358	-1.16E-04	2.88E-09	0.031
9	3349487	4378727	700	-9.33E-05	7.31E-09	0.275
9	4380096	4590305	142	4.53E-05	1.50E-09	0.243
9	4591655	5273194	342	1.30E-04	2.47E-09	0.009
9	5273896	6144065	384	-1.62E-05	2.78E-09	0.758
9	6144333	7232230	569	5.82E-05	4.62E-09	0.392
9	7232510	7757891	465	-2.11E-05	4.08E-09	0.741
9	7758579	8235898	325	5.68E-05	2.75E-09	0.279
9	8236122	9374664	803	8.13E-05	7.76E-09	0.356
9	9375194	9793342	253	-1.71E-05	2.17E-09	0.714
9	9793846	10358286	350	-5.57E-05	2.99E-09	0.308
9	10358672	10879071	263	5.38E-05	2.52E-09	0.283
9	10879188	11616822	307	-1.91E-05	3.33E-09	0.740
9	11884425	12274220	180	1.01E-06	1.36E-09	0.978
9	12274774	12778224	274	-6.82E-06	2.19E-09	0.884
9	12780295	13797055	477	1.44E-05	4.17E-09	0.823
9	13797496	14712257	605	4.16E-05	7.70E-09	0.635
9	14714681	16054406	644	-1.20E-04	4.85E-09	0.086
9	16056127	16833797	538	-8.87E-05	5.00E-09	0.210
9	16836011	17627988	363	2.43E-05	2.50E-09	0.627
9	17628726	18659920	604	2.14E-05	4.72E-09	0.755
9	18660695	19129349	353	-6.97E-05	5.08E-09	0.328
9	19456098	20324812	482	2.39E-05	4.08E-09	0.708
9	20324848	21549638	502	2.43E-05	3.74E-09	0.692
9	21550959	22968575	596	-6.84E-05	4.46E-09	0.306
9	22968789	24154728	439	4.20E-05	4.56E-09	0.534
9	24155142	25425160	409	3.50E-05	3.24E-09	0.539
9	25425511	26317104	404	6.59E-05	4.20E-09	0.309
9	26317973	27335346	535	-4.34E-05	4.63E-09	0.523
9	27336207	28252383	497	-5.36E-05	4.76E-09	0.437
9	28253887	29622939	660	2.76E-06	4.64E-09	0.968

9	29623053	30819576	414	2.63E-06	2.36E-09	0.957
9	30821676	31836697	373	-2.56E-05	2.48E-09	0.607
9	31837040	32249649	172	-4.00E-06	1.07E-09	0.903
9	32250247	33186788	464	-5.07E-05	3.28E-09	0.376
9	33187897	34769004	521	-2.57E-04	6.92E-09	0.002
9	34771534	36712124	645	-5.71E-05	5.08E-09	0.423
9	36714252	37678932	456	3.12E-05	3.73E-09	0.609
9	37679713	42220384	544	4.45E-05	4.77E-09	0.520
9	69576527	71968825	445	-2.06E-05	3.38E-09	0.723
9	71969482	73398448	622	-1.12E-05	3.88E-09	0.858
9	73399750	73916072	309	-3.44E-05	2.02E-09	0.444
9	73916583	74600739	231	-2.69E-05	1.96E-09	0.544
9	74600822	75517582	356	-3.85E-05	2.19E-09	0.411
9	75519251	76785168	379	-1.00E-05	2.63E-09	0.845
9	76786297	78214833	562	-2.05E-05	4.41E-09	0.757
9	78215299	78900183	469	2.97E-06	5.68E-09	0.969
9	81066078	81860861	384	-5.38E-06	3.18E-09	0.924
9	78901112	81064897	968	-3.53E-06	7.69E-09	0.968
9	81863722	82348771	246	-3.76E-05	2.35E-09	0.438
9	82349606	83041982	353	2.16E-05	3.11E-09	0.698
9	83043376	83455568	219	5.78E-05	1.65E-09	0.155
9	83455867	84388566	421	-5.69E-05	3.53E-09	0.339
9	84389168	86180165	683	-1.48E-05	5.41E-09	0.841
9	87571959	87811640	150	4.83E-06	1.08E-09	0.883
9	86181461	87571563	629	-9.14E-05	4.24E-09	0.161
9	87813612	88867151	355	1.97E-05	2.44E-09	0.689
9	88869630	89948499	516	-4.64E-05	4.09E-09	0.468
9	89949778	90198251	198	-4.76E-05	1.56E-09	0.228
9	90198375	90893610	338	-1.62E-07	2.79E-09	0.998
9	90893774	91608628	247	-3.52E-05	1.93E-09	0.423
9	91610543	92342927	327	1.87E-05	2.78E-09	0.723
9	92443570	93441674	461	4.77E-05	3.67E-09	0.431
9	93442205	94163515	361	5.81E-05	3.15E-09	0.301
9	94164373	95854872	626	2.35E-05	4.53E-09	0.727
9	95855100	97719592	731	4.71E-05	4.72E-09	0.493
9	97720818	98889276	456	-1.96E-05	3.16E-09	0.728
9	98891743	100917602	625	2.06E-05	3.77E-09	0.737

9	100919318	101473404	376	-4.27E-05	3.35E-09	0.460
9	101474837	103543692	679	-9.50E-05	3.85E-09	0.125
9	103544757	103969076	179	1.30E-07	8.80E-10	0.996
9	103969339	104552711	293	1.94E-05	2.22E-09	0.681
9	104553938	105041375	303	-2.37E-05	2.35E-09	0.626
9	105041756	105743445	320	-6.05E-06	2.17E-09	0.897
9	105744075	106487777	333	2.12E-05	2.54E-09	0.674
9	106488291	107545903	496	3.41E-05	2.57E-09	0.501
9	107546901	108751162	555	1.47E-05	4.09E-09	0.818
9	108754828	109972524	499	-8.52E-05	5.90E-09	0.268
9	109974206	111137001	674	5.18E-05	7.93E-09	0.561
9	111138232	111997222	549	1.30E-05	3.80E-09	0.833
9	112001473	112593531	326	-1.30E-05	2.43E-09	0.792
9	112594858	113255402	401	2.19E-05	3.72E-09	0.720
9	113255544	114077164	462	2.77E-05	3.64E-09	0.646
9	114078103	114828332	330	-4.79E-05	2.43E-09	0.332
9	114830330	115391053	230	-8.79E-07	1.19E-09	0.980
9	115393806	116148630	399	-2.80E-05	3.17E-09	0.619
9	116149967	117306807	689	-6.83E-05	5.82E-09	0.371
9	117307880	118569429	682	-1.92E-05	4.95E-09	0.784
9	118570426	120030166	743	-5.88E-06	5.95E-09	0.939
9	121009165	121318886	151	9.16E-06	1.11E-09	0.783
9	120030269	121008844	499	2.24E-05	3.64E-09	0.710
9	121319662	122258204	434	-3.79E-05	3.09E-09	0.495
9	122258577	124870612	937	5.49E-05	5.97E-09	0.477
9	124871322	125542861	336	-3.97E-05	2.96E-09	0.466
9	125545194	126926376	449	-5.53E-05	3.06E-09	0.317
9	128927088	129477470	292	4.43E-05	3.46E-09	0.451
9	126927204	128926989	624	-3.16E-05	3.32E-09	0.583
9	129477886	131429495	582	3.29E-05	3.93E-09	0.600
9	131430602	132891863	505	-8.62E-05	4.08E-09	0.177
9	132895131	133640777	305	-5.49E-06	3.60E-09	0.927
9	133642918	134639655	432	-3.13E-05	3.22E-09	0.582
9	134640928	135401279	345	6.45E-05	4.13E-09	0.316
9	135401721	136417159	501	6.12E-05	5.15E-09	0.394
9	136429816	136979539	317	4.75E-05	3.43E-09	0.417
9	136979744	137928846	495	-2.16E-05	5.62E-09	0.773

9	137929853	138817142	514	-4.79E-05	6.03E-09	0.537
9	138817178	141146684	723	-1.43E-05	5.74E-09	0.850
10	60969	747115	154	-1.79E-05	1.31E-09	0.620
10	747772	1330771	280	2.51E-05	2.25E-09	0.596
10	1331955	1609370	172	-1.26E-05	1.69E-09	0.760
10	1610839	2104472	389	-4.57E-05	3.58E-09	0.445
10	2106418	3143283	689	1.22E-05	6.26E-09	0.877
10	3144251	3785605	441	1.72E-05	3.84E-09	0.781
10	3786476	5358542	956	5.12E-05	8.07E-09	0.569
10	5359010	6316910	614	5.90E-05	5.28E-09	0.417
10	6318458	7639684	883	9.82E-05	8.95E-09	0.299
10	9433090	10540465	440	-5.92E-06	3.44E-09	0.920
10	7639932	9432277	1108	-1.11E-04	9.21E-09	0.249
10	12092208	12798954	371	-5.71E-05	4.00E-09	0.367
10	10542217	12092029	828	-1.06E-04	7.49E-09	0.223
10	12799286	13718384	597	-3.23E-05	5.57E-09	0.665
10	13719970	14411956	499	1.20E-05	5.18E-09	0.867
10	14412084	15303527	489	6.48E-06	5.11E-09	0.928
10	15303983	16443442	476	1.56E-05	3.38E-09	0.788
10	16444693	17591626	664	-4.92E-05	5.75E-09	0.516
10	17591919	19202731	694	1.04E-05	5.17E-09	0.885
10	19203300	20131614	450	-2.53E-06	3.18E-09	0.964
10	20132516	21243558	680	-6.21E-05	5.47E-09	0.401
10	21244087	23696890	631	-3.84E-05	6.04E-09	0.621
10	23698033	25414215	686	-1.89E-05	4.86E-09	0.786
10	25416598	26631987	555	9.27E-06	3.82E-09	0.881
10	26633775	27667904	411	7.00E-05	2.93E-09	0.196
10	27668052	28734655	508	-5.44E-05	3.68E-09	0.370
10	28735045	29174963	234	-8.55E-06	1.67E-09	0.834
10	29175101	29961838	535	1.59E-05	7.15E-09	0.851
10	29962511	30653567	388	6.91E-05	3.52E-09	0.244
10	30654528	31479103	433	6.26E-05	4.03E-09	0.324
10	31479422	33656047	779	2.07E-05	4.75E-09	0.764
10	36020445	36884906	379	1.16E-05	3.17E-09	0.837
10	33656119	36017592	829	9.14E-07	5.39E-09	0.990
10	36885921	37845308	313	2.12E-06	3.09E-09	0.970
10	38367233	43220539	218	2.84E-07	1.12E-09	0.993

10	43222372	43605902	162	-1.31E-05	1.53E-09	0.738
10	43606687	45247184	692	3.01E-07	4.62E-09	0.996
10	45247418	46769302	372	1.45E-04	3.58E-09	0.016
10	47768664	49812917	361	-2.30E-05	3.70E-09	0.706
10	49816189	50839403	568	-1.89E-05	4.20E-09	0.771
10	50840736	52086621	209	-6.40E-05	1.07E-08	0.535
10	52086939	52707026	238	-2.99E-05	1.50E-09	0.440
10	52708822	53362540	343	-3.56E-05	2.80E-09	0.501
10	53362932	54413554	512	6.95E-06	4.09E-09	0.913
10	54413909	55334166	523	-8.69E-05	4.20E-09	0.180
10	55334740	56204677	453	-2.16E-06	2.74E-09	0.967
10	56207098	56883966	279	1.52E-05	3.53E-09	0.798
10	56885639	58806290	704	-5.67E-05	4.05E-09	0.373
10	59158617	59822195	312	4.26E-05	1.90E-09	0.328
10	59822974	60375613	206	3.38E-06	1.09E-09	0.919
10	60375857	62558737	972	-9.31E-05	7.03E-09	0.267
10	62558842	64263322	626	-4.12E-05	4.52E-09	0.540
10	64263402	65469133	435	-8.05E-06	2.73E-09	0.878
10	65735028	66980708	394	1.56E-05	2.59E-09	0.759
10	66980980	67545544	227	2.25E-06	1.71E-09	0.957
10	67546331	68429563	514	-2.16E-05	3.99E-09	0.732
10	68430236	69893580	549	-4.51E-05	6.99E-09	0.589
10	69896336	71052508	440	-2.41E-06	3.32E-09	0.967
10	71052888	72479853	930	-3.14E-05	9.81E-09	0.751
10	72480072	73612902	784	-1.27E-05	7.99E-09	0.887
10	73614288	75696752	549	1.02E-05	2.09E-09	0.823
10	78642246	79740262	595	-1.57E-05	4.64E-09	0.818
10	75700056	78638957	888	4.67E-05	5.01E-09	0.509
10	79742617	80874092	594	-5.83E-05	5.70E-09	0.440
10	80874523	81881954	307	1.51E-04	7.18E-09	0.074
10	81882370	82378624	279	3.77E-05	3.65E-09	0.533
10	82379403	82881996	243	3.29E-05	2.19E-09	0.482
10	82883949	83597042	269	-6.01E-05	2.15E-09	0.195
10	83597818	84383108	342	1.38E-06	2.29E-09	0.977
10	84383277	85424102	487	2.73E-05	3.11E-09	0.624
10	85424493	86459792	528	-9.35E-05	4.40E-09	0.159
10	86460169	87285511	356	1.82E-05	2.21E-09	0.699

10	87285907	88450872	497	-1.76E-05	4.23E-09	0.786
10	88451869	89845801	371	1.37E-05	2.85E-09	0.798
10	89846944	90734906	410	7.55E-06	3.18E-09	0.894
10	90735832	91959588	592	6.41E-05	4.73E-09	0.351
10	91960142	92682235	320	3.05E-05	1.98E-09	0.494
10	92682369	94196248	469	2.01E-05	3.07E-09	0.717
10	94196690	95064828	355	1.60E-05	2.01E-09	0.722
10	95065655	95946938	409	1.13E-06	3.35E-09	0.984
10	95949432	97984754	853	-7.71E-05	4.84E-09	0.268
10	97985690	99554180	665	-8.75E-05	4.70E-09	0.202
10	99554895	100240681	333	-5.40E-05	2.51E-09	0.282
10	100241204	102089663	714	5.65E-07	4.25E-09	0.993
10	102090924	104207799	690	-2.12E-05	4.15E-09	0.742
10	104208761	106139081	683	-1.22E-04	6.68E-09	0.135
10	106139294	107874146	733	-1.88E-05	4.67E-09	0.784
10	107875182	109577686	742	2.54E-05	5.72E-09	0.737
10	109578567	110311094	318	-1.77E-05	2.08E-09	0.698
10	110311425	112559414	764	1.11E-05	4.88E-09	0.873
10	112560012	113839866	517	-8.47E-06	3.21E-09	0.881
10	113845990	115698315	912	-7.08E-05	7.94E-09	0.427
10	117705146	118416795	358	-1.02E-06	2.55E-09	0.984
10	115700005	117702645	679	-3.74E-05	4.40E-09	0.572
10	118417453	119697663	621	-2.00E-05	5.65E-09	0.790
10	119698700	120994671	529	-3.23E-05	4.46E-09	0.629
10	120997666	121874991	393	4.10E-05	3.15E-09	0.465
10	121875531	122805770	507	1.11E-04	6.26E-09	0.161
10	122807254	123853223	521	9.40E-05	4.55E-09	0.164
10	123855124	124894743	544	-8.06E-06	4.33E-09	0.902
10	124894909	125868860	561	6.20E-05	5.51E-09	0.404
10	125919795	126739513	451	7.75E-05	7.80E-09	0.380
10	126739798	127820978	517	5.63E-05	3.91E-09	0.368
10	127821105	128409952	405	-6.11E-06	3.86E-09	0.922
10	128410393	129482709	632	-8.22E-05	6.84E-09	0.320
10	129483173	130282731	491	2.46E-05	4.78E-09	0.722
10	130282817	131192968	537	-5.38E-05	5.85E-09	0.482
10	131195759	132251820	595	1.89E-05	5.20E-09	0.793
10	132252499	132964299	471	4.62E-05	4.55E-09	0.494

10	132966372	133355925	285	1.45E-05	3.42E-09	0.804
10	133356417	134858046	638	-4.66E-05	5.57E-09	0.533
10	134858720	135524335	243	-7.30E-06	1.75E-09	0.861
11	333347	1057948	320	-2.28E-05	2.37E-09	0.640
11	1058583	1861912	309	5.34E-05	5.62E-09	0.476
11	1862168	2422034	244	-1.19E-04	9.43E-09	0.222
11	2423545	3660412	524	-6.83E-05	5.72E-09	0.366
11	3661873	4745931	519	-4.28E-05	4.31E-09	0.514
11	4746391	5529152	655	-2.01E-05	4.65E-09	0.768
11	5532222	6010718	357	7.81E-05	2.99E-09	0.153
11	6011761	6917273	555	-4.54E-05	4.48E-09	0.498
11	6917772	7715871	483	-7.21E-05	4.78E-09	0.297
11	7717884	9068716	668	1.04E-04	5.24E-09	0.149
11	9069046	10370675	440	-8.06E-05	2.99E-09	0.140
11	10371614	11361034	681	-6.62E-05	6.27E-09	0.403
11	11361458	12018942	492	4.34E-05	4.85E-09	0.533
11	12019010	13133902	717	-9.56E-05	7.12E-09	0.257
11	13134600	14934686	720	3.30E-05	4.62E-09	0.627
11	14936943	16789155	642	-1.86E-05	3.95E-09	0.767
11	16790799	18788126	918	8.17E-05	6.82E-09	0.323
11	18789820	20113591	792	-3.20E-05	7.58E-09	0.713
11	21773680	23111273	502	-7.44E-06	3.58E-09	0.901
11	20115277	21773251	1016	2.23E-05	9.76E-09	0.822
11	24750113	25106557	163	2.52E-06	1.19E-09	0.942
11	23112584	24749839	709	-6.70E-05	6.03E-09	0.389
11	25109191	26046095	403	-1.08E-06	2.78E-09	0.984
11	26046627	27018778	418	4.28E-07	2.88E-09	0.994
11	27019873	28741185	545	2.15E-05	3.40E-09	0.713
11	28742220	30056918	404	-4.99E-06	3.02E-09	0.928
11	30057440	31853968	577	7.48E-06	3.05E-09	0.892
11	31856654	33383448	567	2.99E-05	6.11E-09	0.702
11	33384046	34321466	430	2.22E-05	3.83E-09	0.719
11	34321876	35034307	445	-1.77E-04	9.81E-09	0.074
11	35036029	36117340	598	7.66E-05	5.39E-09	0.297
11	36118027	37838625	746	-1.46E-05	6.23E-09	0.854
11	37839347	40357803	640	2.05E-05	4.29E-09	0.754
11	40359663	40716222	154	-1.67E-05	1.08E-09	0.613

11	40716823	42243196	591	-5.04E-06	4.28E-09	0.939
11	42244050	42854352	257	2.54E-06	1.72E-09	0.951
11	42854878	43952796	470	-4.62E-05	3.50E-09	0.435
11	46252956	47893120	417	-4.43E-05	2.70E-09	0.393
11	43953774	46245900	1084	1.12E-05	1.07E-08	0.914
11	48351331	49659131	215	-1.03E-05	1.06E-09	0.751
11	49660004	55116367	304	-2.18E-05	1.44E-09	0.566
11	55116451	56160712	426	-4.99E-06	2.06E-09	0.913
11	56625775	58455947	713	-3.85E-05	3.91E-09	0.538
11	58457495	59615953	298	2.18E-05	1.53E-09	0.577
11	59620206	61870732	816	-3.22E-05	6.25E-09	0.684
11	61873755	63153602	405	-6.18E-05	3.63E-09	0.305
11	66835372	68515239	414	-3.38E-06	4.74E-09	0.961
11	63154309	66835194	1070	8.65E-05	6.79E-09	0.294
11	68521072	69508567	391	-4.66E-05	1.28E-08	0.681
11	69509343	70613763	469	7.58E-05	5.39E-09	0.302
11	70616465	71842303	364	2.39E-05	2.45E-09	0.630
11	71842976	74158869	681	-4.83E-05	4.89E-09	0.490
11	74159542	76431100	890	3.84E-05	6.41E-09	0.632
11	76432156	77903706	543	1.35E-05	3.09E-09	0.808
11	77904339	79723318	879	7.51E-07	6.58E-09	0.993
11	79723675	80720242	465	5.78E-05	4.50E-09	0.389
11	80721384	81653514	551	1.78E-05	4.45E-09	0.790
11	81654617	82844918	481	-3.30E-05	3.58E-09	0.581
11	82847086	83575703	267	-2.69E-06	1.56E-09	0.946
11	83576460	84208616	304	-1.57E-05	2.37E-09	0.746
11	84212522	84618654	169	4.22E-06	1.52E-09	0.914
11	84618788	86623913	833	1.07E-05	6.02E-09	0.890
11	86625137	87429545	370	-3.14E-05	2.48E-09	0.529
11	87431647	87827395	167	2.77E-06	1.54E-09	0.944
11	87827514	89208590	526	5.12E-05	4.13E-09	0.425
11	89441680	91115475	481	-1.92E-05	2.85E-09	0.719
11	91118661	91676282	198	1.37E-05	1.53E-09	0.726
11	91677374	92626428	367	2.98E-06	2.16E-09	0.949
11	92627643	93270431	278	-2.59E-05	2.07E-09	0.570
11	93272186	94389331	421	-8.89E-06	2.85E-09	0.868
11	94391845	95329108	416	4.65E-05	3.96E-09	0.459

11	95332310	96848965	669	-6.87E-06	5.39E-09	0.925
11	96849530	98703542	778	2.99E-05	6.54E-09	0.712
11	98703733	99613901	508	4.10E-05	4.43E-09	0.538
11	101158517	101892922	344	6.02E-06	2.02E-09	0.894
11	99614560	101156501	814	-2.30E-05	6.73E-09	0.779
11	101894471	102668702	379	-3.02E-05	6.83E-09	0.715
11	102668882	103491310	383	3.33E-06	2.80E-09	0.950
11	103491586	104871322	551	5.81E-05	3.62E-09	0.334
11	104872103	106251514	419	2.53E-05	2.45E-09	0.610
11	106252171	107489662	537	-6.75E-06	4.60E-09	0.921
11	107491606	108495975	327	-6.40E-05	2.09E-09	0.161
11	108496357	110701645	740	4.52E-05	4.81E-09	0.515
11	110702636	111981853	469	4.23E-05	2.99E-09	0.439
11	111985737	113103996	469	5.78E-06	3.27E-09	0.919
11	113105405	113958177	414	-1.15E-04	5.60E-09	0.126
11	113959061	114742322	448	-2.49E-05	3.83E-09	0.687
11	114743956	115627217	415	-1.44E-06	3.51E-09	0.981
11	115652723	117097952	689	2.17E-05	6.40E-09	0.786
11	117098436	118898319	897	1.84E-05	7.28E-09	0.829
11	118899218	120171371	574	6.34E-06	5.04E-09	0.929
11	120173354	121660636	713	-7.07E-05	5.43E-09	0.337
11	121661507	123289851	721	-3.78E-05	7.10E-09	0.654
11	123291301	124403522	618	3.49E-05	4.94E-09	0.620
11	124404297	125984562	770	7.54E-05	6.89E-09	0.364
11	125984970	126772347	528	-2.04E-05	4.84E-09	0.770
11	126772905	127715860	459	1.52E-05	3.85E-09	0.807
11	127716140	129186210	717	-2.80E-05	6.19E-09	0.722
11	129187995	129838375	322	-2.84E-08	3.27E-09	1.000
11	129839528	130892029	437	-9.86E-05	3.97E-09	0.117
11	130894131	131870763	676	2.08E-05	6.64E-09	0.798
11	131871467	132386355	340	-1.19E-05	2.49E-09	0.811
11	132386834	133679495	783	9.07E-06	8.44E-09	0.921
11	133679758	134946382	719	-1.48E-04	7.39E-09	0.085
12	68657	665200	216	1.02E-05	2.03E-09	0.820
12	665655	1602602	326	3.21E-05	2.63E-09	0.531
12	1602652	2176046	281	1.13E-05	3.04E-09	0.837
12	2176265	2886299	325	4.56E-07	3.19E-09	0.994

12	2886946	3558488	354	4.17E-05	3.64E-09	0.489
12	3559429	4693778	656	-9.64E-05	7.29E-09	0.259
12	4695425	5877653	808	5.92E-05	7.35E-09	0.490
12	5878686	6879279	441	9.18E-05	4.36E-09	0.164
12	8658644	10477423	257	-6.10E-07	2.20E-09	0.990
12	10477712	11684725	448	1.88E-05	2.87E-09	0.725
12	11687997	12530718	424	-1.22E-04	4.45E-09	0.067
12	12533494	13362446	381	-3.61E-05	4.49E-09	0.589
12	13362818	14658341	700	2.08E-08	1.13E-08	1.000
12	14659441	16423287	588	-1.01E-05	4.11E-09	0.875
12	16423998	18466533	744	1.80E-05	5.80E-09	0.813
12	18467618	19924288	582	-2.03E-05	5.45E-09	0.783
12	19925541	21568051	878	-4.21E-05	6.10E-09	0.590
12	21569730	22325878	305	-2.10E-05	2.08E-09	0.645
12	22327634	22943718	204	1.56E-05	1.64E-09	0.700
12	22944771	23819079	359	-1.63E-04	3.46E-09	0.005
12	23819364	24545631	412	1.52E-04	4.70E-09	0.026
12	24545834	25991825	716	6.67E-05	5.68E-09	0.376
12	25992543	27529660	775	1.18E-05	7.00E-09	0.888
12	27529712	29111136	700	2.30E-05	4.84E-09	0.740
12	29111480	30354932	709	-4.82E-05	4.86E-09	0.489
12	30355547	31067009	415	-2.56E-06	2.65E-09	0.960
12	31067490	32081072	408	-2.96E-05	3.78E-09	0.630
12	32082695	32645755	311	1.12E-05	2.79E-09	0.832
12	32645848	33435901	369	4.34E-06	2.53E-09	0.931
12	33436298	38231063	325	-1.26E-05	1.73E-09	0.762
12	38233264	40017827	410	-1.93E-05	2.11E-09	0.674
12	40022029	41015211	475	6.21E-06	3.21E-09	0.913
12	41015333	42346846	665	3.35E-05	4.43E-09	0.615
12	42347363	43074061	261	2.76E-05	1.72E-09	0.506
12	43074434	43982146	422	8.62E-06	2.70E-09	0.868
12	43982342	44922863	221	-2.00E-05	1.45E-09	0.600
12	44923334	46022099	342	-3.41E-06	2.22E-09	0.942
12	46023426	47504173	481	1.51E-05	3.09E-09	0.786
12	47505424	48267360	361	2.81E-05	3.56E-09	0.637
12	48267635	48965461	366	1.98E-05	7.85E-09	0.824
12	48967935	50160195	315	1.07E-04	1.74E-09	0.010

12	50160662	51582048	414	1.10E-04	4.64E-09	0.105
12	51582188	52913730	700	5.30E-05	4.77E-09	0.443
12	52914211	54379670	717	6.60E-05	1.62E-08	0.604
12	56795415	57534912	235	6.19E-05	1.57E-09	0.118
12	54380016	56792337	763	-1.18E-04	5.56E-09	0.114
12	57535266	58748272	385	-1.18E-05	2.20E-09	0.802
12	58748846	60859665	699	-2.44E-06	4.23E-09	0.970
12	60859809	61738896	266	-1.27E-06	1.40E-09	0.973
12	61740561	63158569	602	2.47E-05	4.78E-09	0.721
12	63158712	63908647	359	-3.51E-05	2.91E-09	0.514
12	63910665	65383507	408	4.56E-06	3.25E-09	0.936
12	65926411	67039427	476	-2.60E-05	3.14E-09	0.642
12	67039600	67817612	376	6.31E-05	3.91E-09	0.313
12	67818111	68740414	516	-4.12E-05	4.42E-09	0.535
12	68742112	69826093	486	-9.36E-05	4.74E-09	0.174
12	69826237	70815755	526	1.43E-05	3.68E-09	0.814
12	70817279	71688642	413	-4.97E-05	3.61E-09	0.408
12	71690933	72513196	316	2.08E-05	2.15E-09	0.654
12	72525849	74846997	634	-3.97E-06	4.13E-09	0.951
12	75193041	76058346	327	1.46E-05	2.22E-09	0.757
12	76060283	77098899	452	4.71E-05	3.49E-09	0.425
12	77099081	78398357	624	3.60E-05	4.80E-09	0.603
12	78400884	78925445	243	4.74E-05	2.32E-09	0.325
12	78926240	80944201	511	9.51E-06	3.21E-09	0.867
12	80945774	83137872	721	9.92E-06	5.50E-09	0.894
12	83143660	83854442	270	-1.10E-06	2.40E-09	0.982
12	83856540	85014953	417	7.73E-06	2.23E-09	0.870
12	85016898	87296535	569	-1.45E-05	3.13E-09	0.796
12	87297692	87905878	166	-5.97E-06	1.08E-09	0.856
12	87909726	88848006	218	6.65E-06	1.38E-09	0.858
12	88851543	91827908	896	-2.97E-06	6.53E-09	0.971
12	91827930	93792073	767	-3.78E-05	6.64E-09	0.643
12	93792655	95717265	1015	-7.94E-05	9.03E-09	0.404
12	95717497	97439396	838	-4.55E-05	6.71E-09	0.579
12	99224060	99816442	205	-2.00E-05	1.80E-09	0.638
12	97439589	99220284	912	-6.02E-05	7.80E-09	0.496
12	99816842	101857521	803	7.34E-05	6.51E-09	0.363

12	101857796	102961329	417	-1.48E-04	2.94E-09	0.006
12	102961450	104011319	433	-1.67E-05	3.39E-09	0.774
12	105817813	106155478	204	1.34E-05	1.98E-09	0.763
12	106156001	107521770	612	5.71E-05	3.92E-09	0.362
12	107527013	108504440	424	1.35E-04	3.16E-09	0.016
12	104012667	105816376	1163	8.67E-05	8.23E-09	0.339
12	108506696	110111462	700	-8.01E-05	4.76E-09	0.246
12	110112932	113027651	567	-7.04E-05	4.76E-09	0.308
12	113030227	113877766	334	7.35E-05	2.33E-09	0.128
12	113885696	114882476	596	1.86E-04	1.24E-08	0.095
12	114883085	115326972	185	-1.73E-05	2.87E-09	0.747
12	117087697	117644288	184	-1.63E-05	1.43E-09	0.667
12	115329869	117087376	969	2.41E-05	9.15E-09	0.801
12	117644436	118888131	675	4.63E-05	6.48E-09	0.565
12	118888731	119301273	203	-7.79E-08	1.96E-09	0.999
12	119303044	120007172	394	-1.31E-04	5.06E-09	0.065
12	120008093	121720040	644	-6.04E-05	4.22E-09	0.352
12	121721831	124710880	880	6.19E-06	4.94E-09	0.930
12	124710996	125416169	363	-3.10E-05	5.22E-09	0.668
12	125417493	126461057	519	1.76E-05	5.06E-09	0.804
12	126463562	127382559	515	-8.88E-05	3.95E-09	0.158
12	127383648	128141516	402	1.93E-05	4.49E-09	0.773
12	128141610	128810520	447	1.09E-05	4.58E-09	0.872
12	128814615	129688509	573	5.84E-05	7.37E-09	0.496
12	129689639	130157939	346	-1.66E-05	3.94E-09	0.792
12	130158997	130859973	457	3.44E-05	4.98E-09	0.626
12	130861322	131407836	344	7.34E-05	3.92E-09	0.241
12	131408716	132012266	375	-7.65E-06	3.30E-09	0.894
12	132014206	132706778	276	-1.09E-05	2.32E-09	0.821
12	132807034	133841558	235	-1.46E-04	1.41E-08	0.219
13	19020095	20682304	395	-1.98E-05	2.01E-09	0.659
13	20684769	21688499	463	1.70E-05	3.23E-09	0.764
13	21688974	22209012	220	-5.04E-05	1.60E-09	0.208
13	23590702	23827019	193	-1.87E-05	2.46E-09	0.707
13	22211354	23590611	814	1.37E-05	8.97E-09	0.885
13	23827815	24477568	384	9.04E-05	2.91E-09	0.094
13	24477971	25210959	503	-4.62E-05	4.38E-09	0.485

13	25212359	25639070	222	-6.70E-05	1.68E-09	0.103
13	25640695	27282973	736	-2.08E-05	5.04E-09	0.769
13	27283115	28283059	561	-1.64E-06	4.86E-09	0.981
13	28284459	28876214	285	-2.73E-05	1.98E-09	0.539
13	28879332	29872965	451	-5.67E-05	3.45E-09	0.334
13	29873521	31379027	787	4.61E-05	6.83E-09	0.577
13	31384910	32983250	798	-3.99E-05	5.72E-09	0.598
13	32984197	34681078	710	-5.64E-05	3.92E-09	0.368
13	34683262	36042839	439	-2.93E-06	3.51E-09	0.961
13	36043978	37027089	549	2.59E-05	3.76E-09	0.672
13	38291011	38877678	287	-5.73E-06	1.73E-09	0.890
13	37029009	38290705	619	2.59E-05	3.73E-09	0.672
13	38887155	40179288	575	-3.06E-05	3.40E-09	0.600
13	40181792	41773356	639	-8.40E-05	4.37E-09	0.204
13	41774489	43099692	548	3.20E-05	3.34E-09	0.580
13	46493234	46866562	161	-1.17E-05	9.20E-10	0.699
13	43099889	44637493	786	1.23E-05	5.05E-09	0.863
13	44638337	46491101	755	-3.47E-05	5.22E-09	0.631
13	46869621	48439005	725	-7.73E-06	4.74E-09	0.911
13	48440893	49987145	462	1.64E-06	2.10E-09	0.971
13	49987663	51254196	497	6.69E-05	5.96E-09	0.386
13	51254712	53336663	702	-5.27E-05	4.02E-09	0.405
13	53337952	54681048	611	3.23E-05	4.21E-09	0.619
13	54681363	56433642	544	5.16E-05	2.82E-09	0.331
13	56434161	58247964	413	1.87E-05	1.79E-09	0.658
13	58248124	60085069	714	2.35E-05	4.67E-09	0.731
13	60087834	60854106	241	-4.62E-05	1.13E-09	0.170
13	60855021	61589772	348	8.54E-07	2.03E-09	0.985
13	61591949	62912481	486	-6.09E-06	2.82E-09	0.909
13	62913542	64201284	415	-1.18E-05	2.60E-09	0.816
13	64202212	65339575	366	-9.34E-06	1.82E-09	0.827
13	65340421	66197786	243	-7.06E-06	1.02E-09	0.825
13	66200191	67713633	592	-1.77E-05	3.40E-09	0.762
13	67714255	69561090	645	-3.53E-05	3.94E-09	0.574
13	69561894	70642326	429	-7.12E-06	3.03E-09	0.897
13	70643712	71095217	239	8.36E-06	1.70E-09	0.839
13	71095483	71759743	313	-3.61E-05	3.58E-09	0.546

13	71763633	72958993	473	1.15E-05	3.86E-09	0.853
13	72959394	73795534	346	5.48E-05	7.07E-09	0.514
13	73796728	75084163	678	-1.03E-06	5.51E-09	0.989
13	75084473	76504672	666	1.78E-04	1.01E-08	0.078
13	76505282	78030962	671	-1.41E-05	4.42E-09	0.832
13	78032193	78807439	318	-1.40E-05	1.81E-09	0.742
13	78807836	80252742	553	-2.46E-06	2.99E-09	0.964
13	82580994	84132765	494	3.91E-05	2.19E-09	0.404
13	80257200	82580321	856	5.29E-06	6.00E-09	0.946
13	84133082	85035423	269	1.02E-05	1.62E-09	0.801
13	85037313	85609664	262	1.69E-05	1.52E-09	0.664
13	85609760	87039195	528	3.91E-05	3.32E-09	0.498
13	87039360	88190520	314	3.96E-05	1.45E-09	0.298
13	88191656	89057861	239	-2.21E-05	1.37E-09	0.550
13	89059635	90177618	305	5.75E-06	1.34E-09	0.875
13	90179364	90867683	318	-2.01E-05	2.16E-09	0.665
13	90869873	91967960	405	-2.60E-05	2.60E-09	0.610
13	91970313	92912119	365	-1.26E-05	2.24E-09	0.790
13	92912423	93918144	412	7.50E-06	2.60E-09	0.883
13	93918533	94562549	322	4.82E-05	1.79E-09	0.254
13	94563601	95540024	474	5.87E-05	3.89E-09	0.346
13	97521207	98589842	367	1.36E-05	2.32E-09	0.778
13	95540709	97520182	918	2.91E-05	5.64E-09	0.699
13	98594975	100043997	776	-5.17E-05	5.09E-09	0.468
13	100044673	101392748	562	-5.17E-06	3.95E-09	0.934
13	101394726	102299385	584	5.16E-05	3.88E-09	0.407
13	102299498	103339911	558	5.94E-05	3.73E-09	0.330
13	103340536	104065375	505	1.49E-05	3.73E-09	0.808
13	104065534	104842764	367	7.49E-06	2.99E-09	0.891
13	104843186	105990071	584	7.55E-06	6.43E-09	0.925
13	105990812	106439396	245	-2.79E-05	1.99E-09	0.533
13	106442608	107785431	712	-2.43E-05	6.25E-09	0.758
13	107788025	108518444	439	4.57E-05	3.97E-09	0.469
13	108519987	109359792	437	3.56E-05	3.91E-09	0.569
13	109359834	110338482	539	1.62E-05	4.86E-09	0.816
13	110338913	111432821	729	4.04E-05	7.01E-09	0.630
13	113269127	115109853	670	2.89E-06	4.34E-09	0.965

13	111434814	113266084	884	6.88E-05	7.12E-09	0.415
14	20544512	20877114	188	1.19E-05	1.79E-09	0.778
14	20878890	21267077	308	-3.10E-05	4.24E-09	0.634
14	21271573	22378835	522	-4.97E-05	5.44E-09	0.501
14	22379132	22738105	229	5.17E-05	2.30E-09	0.281
14	22738161	23607730	506	1.53E-04	5.44E-09	0.038
14	23607866	24904154	515	4.70E-05	6.43E-09	0.558
14	24904240	26134815	654	-2.44E-06	4.72E-09	0.972
14	26135167	26679549	244	3.46E-06	2.23E-09	0.942
14	26680794	28466225	589	2.72E-05	4.49E-09	0.685
14	28466533	29196856	255	-8.75E-06	1.92E-09	0.841
14	29198059	32014712	797	4.55E-05	6.82E-09	0.581
14	32016504	33033695	420	2.28E-05	3.80E-09	0.712
14	34769883	36344816	497	2.88E-06	3.62E-09	0.962
14	36345767	36682357	143	5.77E-06	9.54E-10	0.852
14	33036081	34769821	1119	-2.13E-04	1.26E-08	0.058
14	36683516	38481516	689	2.55E-04	1.05E-08	0.013
14	38486187	39370633	361	-3.89E-05	2.82E-09	0.464
14	39370726	40336296	361	-2.18E-05	2.49E-09	0.662
14	40336990	41615299	437	1.68E-06	2.70E-09	0.974
14	41615331	43065019	495	2.41E-05	3.10E-09	0.665
14	43068948	44541699	471	-2.12E-05	4.17E-09	0.743
14	44542368	45247021	304	-2.49E-06	2.34E-09	0.959
14	45250418	46311209	221	-2.75E-06	1.06E-09	0.933
14	46312678	47456738	355	7.98E-06	2.04E-09	0.860
14	47457846	48351664	389	9.21E-06	2.84E-09	0.863
14	48352461	49001226	256	2.27E-05	2.23E-09	0.631
14	49004194	49955015	359	2.51E-05	2.47E-09	0.613
14	52078316	52761693	415	1.19E-05	3.60E-09	0.843
14	49955381	52077965	934	4.06E-05	7.56E-09	0.640
14	52762622	54162101	504	6.98E-05	6.20E-09	0.375
14	54162655	55043520	312	-1.07E-05	2.70E-09	0.837
14	55044905	56215082	474	-4.20E-05	3.24E-09	0.461
14	56215477	56949485	467	-2.58E-05	3.37E-09	0.657
14	56949644	58448681	677	-3.97E-05	5.77E-09	0.601
14	58449526	59135770	213	2.22E-05	1.47E-09	0.563
14	59135989	61649612	943	-5.64E-05	5.77E-09	0.458

14	63166807	63890839	304	-1.74E-05	2.11E-09	0.705
14	61651278	63164649	666	9.62E-05	5.83E-09	0.208
14	63897095	65218457	418	-5.54E-05	2.55E-09	0.272
14	65220775	66353557	440	3.96E-05	2.96E-09	0.467
14	66354477	67990648	525	2.97E-05	3.00E-09	0.587
14	67991028	69355606	630	3.83E-05	7.70E-09	0.662
14	71132759	72213258	357	-7.33E-06	2.03E-09	0.871
14	69356353	71131097	787	4.83E-05	5.97E-09	0.532
14	72214790	72888979	421	-2.75E-05	3.08E-09	0.620
14	72889376	74250730	550	-6.49E-05	4.57E-09	0.337
14	74252993	75747118	538	2.85E-05	2.98E-09	0.601
14	75747258	76583135	371	-9.34E-06	3.10E-09	0.867
14	76583775	77332160	386	-3.78E-05	3.80E-09	0.539
14	77332408	78421884	573	3.31E-05	4.43E-09	0.619
14	78422260	79026816	371	2.31E-06	2.97E-09	0.966
14	79027504	80998547	757	4.92E-05	5.83E-09	0.519
14	80999361	81787255	327	1.07E-05	2.28E-09	0.823
14	81787481	82459297	259	1.31E-05	2.20E-09	0.780
14	82461794	84087895	607	-3.08E-05	5.20E-09	0.669
14	84087979	85035235	352	9.81E-06	2.52E-09	0.845
14	85037974	86051751	438	-5.32E-05	3.94E-09	0.397
14	86053777	87334624	501	1.37E-05	3.74E-09	0.823
14	87335256	88641174	451	-2.24E-05	3.13E-09	0.689
14	88641335	90069965	638	-6.08E-05	4.96E-09	0.388
14	90075441	92098486	749	4.51E-05	6.56E-09	0.577
14	92099719	93378838	646	4.72E-05	5.70E-09	0.532
14	93379151	94420996	456	1.62E-05	3.99E-09	0.797
14	94421488	95347359	703	6.02E-05	6.11E-09	0.441
14	95347761	96250900	641	-2.31E-05	6.19E-09	0.769
14	96251418	97570260	662	8.11E-07	6.13E-09	0.992
14	97571437	98987472	651	-2.90E-06	5.48E-09	0.969
14	98989483	99980087	544	-2.70E-05	5.80E-09	0.723
14	99981105	101244250	481	-2.79E-05	3.83E-09	0.652
14	101244293	102339561	445	9.75E-05	5.51E-09	0.189
14	104761778	105440784	241	3.52E-05	2.29E-09	0.461
**14**	**102341650**	**104759919**	**714**	**3.68E-04**	**6.93E-09**	**0.000**
14	105443695	106552839	140	7.91E-06	1.02E-09	0.805

15	22362658	23288776	159	-4.80E-05	1.76E-09	0.252
15	23292175	24320772	300	-6.12E-05	3.28E-09	0.286
15	24321414	24976269	147	-6.89E-06	1.31E-09	0.849
15	25107905	25750941	208	-7.29E-06	1.85E-09	0.865
15	25752114	26185554	309	-1.04E-04	3.56E-09	0.080
15	26186113	26675188	259	8.09E-05	2.95E-09	0.136
15	26676258	27265824	242	-1.56E-05	2.66E-09	0.762
15	27266835	27819408	254	-2.57E-05	2.40E-09	0.600
15	27820352	28325439	264	2.58E-05	2.31E-09	0.591
15	28326536	29617158	227	2.22E-05	1.73E-09	0.593
15	29617517	31314317	411	4.18E-05	3.27E-09	0.465
15	31315570	32177326	354	1.93E-04	1.12E-08	0.068
15	32180607	33251727	348	6.43E-05	3.50E-09	0.277
15	33253484	33792116	378	-1.69E-04	5.58E-09	0.024
15	33793179	34273308	356	-3.74E-05	3.75E-09	0.541
15	34273346	34970673	233	-6.16E-06	1.92E-09	0.888
15	34971983	35941864	367	5.43E-05	2.68E-09	0.294
15	35943377	36424581	299	-1.58E-04	2.63E-09	0.002
15	36425448	36968033	285	-5.32E-06	3.02E-09	0.923
15	36968291	37930544	442	-1.19E-05	3.81E-09	0.847
15	37931874	38804104	337	-8.31E-05	2.79E-09	0.116
15	38805185	39518563	386	5.54E-06	3.38E-09	0.924
15	39519092	40382441	407	1.25E-06	3.35E-09	0.983
15	40383779	41211827	276	-1.33E-04	3.33E-09	0.021
15	41212265	42222063	300	2.39E-05	1.73E-09	0.566
15	42222390	43473907	378	-1.91E-05	2.15E-09	0.680
15	43474655	44988610	261	2.74E-05	1.32E-09	0.451
15	44992475	46292382	424	-6.10E-05	2.69E-09	0.240
15	46294385	47000090	290	2.77E-05	1.91E-09	0.526
15	47000857	47977097	382	-2.47E-05	2.49E-09	0.621
15	47979127	49075918	359	6.99E-05	2.23E-09	0.139
15	49078966	50004817	260	-1.26E-05	1.43E-09	0.738
15	51682775	52589995	273	2.05E-05	1.31E-09	0.572
15	50006184	51681073	717	5.08E-05	4.39E-09	0.443
15	52590615	53014125	170	8.49E-06	9.92E-10	0.788
15	53015101	53766542	361	-2.72E-05	3.78E-09	0.658
15	53767876	54336662	313	-3.67E-05	2.44E-09	0.457

15	54337243	55134743	392	1.81E-05	3.07E-09	0.744
15	55135408	55469163	140	3.61E-06	9.68E-10	0.908
15	55470187	55877891	151	2.14E-06	1.21E-09	0.951
15	55879453	56784345	319	-2.50E-05	2.20E-09	0.593
15	56784506	57637678	235	9.77E-06	1.26E-09	0.783
15	57638075	58438535	510	-7.38E-06	4.09E-09	0.908
15	59507391	60018662	244	3.83E-05	1.88E-09	0.377
15	58439935	59505191	502	1.38E-05	4.28E-09	0.833
15	60019136	60670149	285	-1.69E-05	2.36E-09	0.728
15	60670287	61803306	669	1.71E-05	6.89E-09	0.837
15	61803892	62735671	460	6.75E-05	3.42E-09	0.249
15	62737452	64335240	693	-6.50E-05	5.23E-09	0.369
15	65104668	66358474	354	2.31E-05	1.92E-09	0.599
15	66359486	66948204	296	-9.07E-05	2.47E-09	0.068
15	66948951	68640917	759	1.44E-05	4.97E-09	0.839
15	68642047	69936117	406	-3.15E-05	3.47E-09	0.592
15	69938058	70772135	451	1.53E-04	5.10E-09	0.032
15	70772414	71919256	463	-6.23E-05	3.50E-09	0.292
15	74453873	75516403	321	-1.70E-04	3.54E-09	0.004
15	71920065	74447996	707	-1.68E-05	4.21E-09	0.795
15	75520945	76397358	160	1.29E-05	9.90E-10	0.681
15	76397390	77910873	428	1.93E-06	2.21E-09	0.967
15	77912408	78637877	276	2.08E-05	2.61E-09	0.684
15	78640760	79259009	192	-1.99E-05	1.42E-09	0.597
15	79261077	79991408	436	-4.70E-05	3.36E-09	0.418
15	79992629	80635045	302	-8.75E-06	2.85E-09	0.870
15	80637749	81604871	477	-2.16E-05	4.41E-09	0.745
15	81607249	84062334	626	2.39E-06	4.55E-09	0.972
15	84063245	85517022	506	-1.15E-05	4.04E-09	0.856
15	85518062	86341812	361	-4.02E-05	2.07E-09	0.377
15	86342119	86650301	159	2.65E-05	1.32E-09	0.466
15	86650331	87113409	248	-8.62E-06	2.01E-09	0.848
15	87114392	87693557	314	-4.98E-05	3.05E-09	0.367
15	87693885	88032107	160	2.09E-05	1.34E-09	0.568
15	88032736	88742960	322	-5.15E-06	2.62E-09	0.920
15	88743108	89561223	336	4.53E-05	3.30E-09	0.430
15	89562631	90452882	361	1.92E-06	2.54E-09	0.970

15	90453183	91221307	299	1.27E-05	2.19E-09	0.787
15	91226586	91745246	252	-4.50E-05	2.60E-09	0.377
15	91749626	92164207	203	7.70E-05	2.18E-09	0.099
15	92165068	92667377	253	1.23E-06	2.55E-09	0.981
15	92669184	93590376	566	6.80E-05	5.19E-09	0.345
15	93591893	93908402	232	1.05E-04	3.64E-09	0.081
15	93909052	94547933	368	1.97E-06	3.62E-09	0.974
15	94548924	95576904	643	2.09E-05	6.97E-09	0.803
15	95577096	96497729	429	-1.07E-04	4.58E-09	0.115
15	96500358	97091511	333	-6.20E-05	3.49E-09	0.294
15	97092654	98496698	629	5.83E-05	7.31E-09	0.495
15	98497881	99128454	384	-1.05E-04	4.30E-09	0.110
15	100078196	100456641	152	-1.14E-05	1.31E-09	0.754
15	99128932	100077720	485	1.84E-05	4.73E-09	0.789
15	101408616	101918220	304	9.16E-05	2.80E-09	0.084
15	100457692	101403655	488	-9.53E-05	4.93E-09	0.175
15	101918830	102516259	287	3.67E-05	3.00E-09	0.503
16	60291	596605	208	-6.40E-06	2.00E-09	0.886
16	598838	945806	136	-2.95E-06	1.20E-09	0.932
16	946205	1547999	261	4.03E-06	2.46E-09	0.935
16	1551082	2293296	333	6.21E-05	2.45E-09	0.209
16	2294890	3960458	489	-5.67E-05	5.00E-09	0.423
16	3961770	4689471	275	9.91E-06	5.33E-09	0.892
16	4690187	5114499	203	-1.27E-05	1.60E-09	0.750
16	5115228	5854496	426	-8.48E-05	4.04E-09	0.183
16	5855169	6264138	318	7.63E-05	3.69E-09	0.209
16	7046430	7524343	442	1.35E-05	4.09E-09	0.833
16	6264139	7045506	647	-2.70E-05	6.09E-09	0.729
16	7525825	8236887	555	2.93E-05	5.55E-09	0.694
16	8237157	8572350	256	4.42E-05	2.89E-09	0.411
16	8572557	8960864	188	-1.20E-05	2.16E-09	0.796
16	8962907	9658861	335	-1.22E-04	3.42E-09	0.037
16	9659584	9997178	212	1.53E-05	1.63E-09	0.705
16	10002419	10430046	325	5.68E-06	2.72E-09	0.913
16	10431221	11493266	531	-1.92E-06	4.74E-09	0.978
16	11612436	12151685	190	1.66E-05	1.77E-09	0.693
16	12775897	13119544	141	-2.13E-05	1.36E-09	0.564

16	12151797	12775407	451	-2.20E-05	3.27E-09	0.700
16	13120367	13978116	506	-2.30E-06	4.26E-09	0.972
16	13979558	15885158	356	2.86E-05	2.71E-09	0.583
16	15885866	16220858	240	-2.60E-06	1.72E-09	0.950
16	16222763	17334499	271	6.45E-08	2.59E-09	0.999
16	17335780	18062164	300	3.66E-05	2.73E-09	0.484
16	18065944	20054371	507	-4.77E-05	3.86E-09	0.443
16	20055884	21235343	435	-5.32E-06	3.31E-09	0.926
16	21237217	23369433	478	-1.51E-05	4.07E-09	0.813
16	23371110	24308825	377	-2.85E-05	2.71E-09	0.585
16	24309108	25589623	527	-5.84E-05	4.18E-09	0.366
16	26683251	26943239	138	-2.65E-05	1.71E-09	0.521
16	25590147	26681997	620	-1.01E-04	6.31E-09	0.205
16	26944850	27445360	274	1.13E-04	3.40E-09	0.053
16	27446054	29023966	445	1.81E-05	2.53E-09	0.719
16	29694822	31380596	353	3.36E-06	2.29E-09	0.944
16	31384554	47981754	267	3.15E-05	1.77E-09	0.453
16	47982405	49006844	259	4.37E-05	2.28E-09	0.360
16	49007408	50694011	658	7.37E-05	6.06E-09	0.344
16	50698071	51700822	458	-4.40E-05	4.28E-09	0.501
16	51703888	53845487	830	8.07E-06	6.01E-09	0.917
16	53848448	54671719	496	-2.36E-04	1.00E-08	0.018
16	54673195	55505040	496	-1.31E-05	4.67E-09	0.848
16	56908045	57663112	346	-8.51E-05	3.83E-09	0.169
16	55506137	56906006	602	-1.30E-04	4.40E-09	0.050
16	57663619	58508278	391	-9.44E-05	7.77E-09	0.284
16	59889269	60744157	301	-2.03E-05	2.60E-09	0.690
16	58510034	59888170	534	2.36E-05	3.92E-09	0.706
16	60745208	61991704	352	-2.40E-05	2.47E-09	0.629
16	61993462	63395786	493	-8.89E-05	4.11E-09	0.165
16	64716851	65538017	376	-1.84E-05	2.43E-09	0.709
16	63396006	64714759	477	-1.87E-05	3.34E-09	0.746
16	68429894	70885818	618	-1.78E-05	3.51E-09	0.764
16	70887506	72915425	541	-1.06E-04	3.11E-09	0.058
16	65538484	68429077	907	-3.48E-05	5.92E-09	0.651
16	72915753	73542956	354	1.17E-05	4.21E-09	0.856
16	73543220	74724933	470	1.99E-05	3.78E-09	0.746

16	74730819	75517115	291	-3.06E-05	2.38E-09	0.531
16	75518129	76056374	169	-2.43E-05	1.68E-09	0.554
16	76058697	76987262	437	-1.92E-05	3.13E-09	0.731
16	76988130	77455781	322	2.65E-05	2.86E-09	0.619
16	77456244	77688760	199	-1.61E-05	1.63E-09	0.691
16	77689074	78446696	584	-4.51E-05	5.76E-09	0.553
16	78452348	78709939	217	-3.09E-06	2.53E-09	0.951
16	78710328	79368760	702	-4.39E-05	7.42E-09	0.610
16	79369779	80184442	562	-8.47E-05	5.16E-09	0.238
16	80184495	81209234	657	-1.09E-07	5.03E-09	0.999
16	81209631	82481987	784	8.44E-05	8.45E-09	0.359
16	82482458	83078581	572	1.46E-05	5.95E-09	0.850
16	83078959	83971446	825	-1.41E-05	8.15E-09	0.876
16	84646612	85146709	319	6.66E-05	3.63E-09	0.269
16	85146935	85663943	211	-1.33E-05	3.52E-09	0.822
16	83972415	84645588	626	-9.66E-05	7.97E-09	0.279
16	85664453	86221644	412	6.39E-05	4.98E-09	0.365
16	86222417	86745679	508	-2.33E-05	6.47E-09	0.773
16	86745955	87173290	254	4.42E-07	3.57E-09	0.994
16	87175053	88389287	532	-9.41E-06	5.22E-09	0.896
16	88439429	88964934	242	-4.90E-06	2.99E-09	0.929
16	88966667	89604610	217	2.82E-07	2.95E-09	0.996
16	89605495	90292767	240	-7.03E-06	2.11E-09	0.879
17	828	296442	133	-5.41E-06	3.14E-09	0.923
17	396660	820493	178	1.49E-04	4.15E-09	0.021
17	820511	1480393	260	-3.59E-05	4.86E-09	0.606
17	1481897	2613925	471	-3.07E-05	6.54E-09	0.704
17	2616352	3425788	387	4.47E-05	6.40E-09	0.576
17	3426133	4321755	384	-7.03E-06	5.29E-09	0.923
17	4322053	4650487	176	-7.88E-06	3.40E-09	0.892
17	4652156	5573766	370	-7.70E-06	4.74E-09	0.911
17	6572170	6975193	181	7.95E-05	2.99E-09	0.146
17	5574433	6570522	609	2.30E-05	9.63E-09	0.815
17	6976284	7723513	317	-1.59E-04	4.69E-09	0.020
17	7725660	8704551	399	7.81E-05	8.63E-09	0.401
17	8705612	9229793	253	-4.74E-05	7.53E-09	0.585
17	9233113	9714549	223	-1.42E-05	3.39E-09	0.808

17	9714995	10573151	522	-5.25E-05	7.89E-09	0.554
17	10573457	11667346	523	1.39E-05	7.80E-09	0.875
17	11668104	13000883	588	-2.99E-05	9.14E-09	0.754
17	13001700	13625425	351	-8.37E-05	5.89E-09	0.276
17	13626102	14209988	323	2.65E-05	4.96E-09	0.706
17	14210015	14499605	185	4.02E-05	3.04E-09	0.466
17	14500054	14859339	269	3.28E-05	3.89E-09	0.599
17	14860784	16319512	496	1.71E-04	7.09E-09	0.042
17	16321959	17292491	307	8.65E-05	4.30E-09	0.187
17	17299591	18446092	382	-3.20E-05	4.48E-09	0.632
17	18456679	19666001	259	-4.80E-06	3.30E-09	0.933
17	20679485	21284673	159	-1.99E-05	2.60E-09	0.697
17	21285166	25677370	210	5.62E-05	2.55E-09	0.266
17	25679294	26272308	203	-5.04E-06	2.47E-09	0.919
17	26273607	27884779	447	-4.89E-05	5.15E-09	0.495
17	27885675	29259899	375	-3.14E-06	4.50E-09	0.963
17	29262773	30878805	457	1.83E-05	7.89E-09	0.837
17	30879433	31989867	616	4.50E-06	8.56E-09	0.961
17	31990385	32677709	410	2.81E-05	5.47E-09	0.704
17	33616080	34393028	274	-3.22E-05	4.09E-09	0.615
17	32677947	33614452	507	-1.66E-05	7.12E-09	0.844
17	34393296	35725997	359	-3.92E-05	4.82E-09	0.573
17	35731855	37341235	493	3.49E-04	6.08E-08	0.157
17	37344586	38649203	331	-7.21E-05	5.34E-09	0.324
17	38652220	39657179	455	-8.47E-06	5.11E-09	0.906
17	39657337	40231221	205	-2.74E-05	2.40E-09	0.577
17	40232810	41769732	392	7.99E-06	4.46E-09	0.905
17	41771040	43317574	552	-5.87E-05	6.87E-09	0.479
17	44863413	45874715	302	3.17E-05	4.65E-09	0.642
17	45876022	46828302	408	-1.11E-04	5.88E-09	0.148
17	46828412	48027295	402	-3.37E-05	1.36E-08	0.773
17	48027688	48832008	360	-4.20E-05	6.06E-09	0.589
17	48832437	49892805	428	-3.28E-05	5.22E-09	0.650
17	49894599	51282380	527	1.09E-05	5.81E-09	0.886
17	52863321	53338298	227	1.88E-05	2.98E-09	0.730
17	51284441	52861905	537	-4.31E-05	6.84E-09	0.602
17	53346718	54164464	384	-4.53E-05	4.73E-09	0.510

17	54164935	55278525	429	-1.46E-05	5.99E-09	0.850
17	57487538	59307733	302	-1.33E-05	5.06E-09	0.852
17	60885287	62035655	261	-2.53E-05	2.92E-09	0.640
17	59309085	60883279	464	-4.01E-05	5.95E-09	0.603
17	55279508	57486793	880	1.37E-04	1.25E-08	0.219
17	62037685	64434272	753	-4.77E-05	9.77E-09	0.629
17	64434524	65013985	328	-9.84E-06	4.75E-09	0.887
17	65016683	66315468	382	-1.92E-04	6.43E-09	0.017
17	66318466	66903293	310	4.65E-05	4.32E-09	0.479
17	66906217	68199133	460	6.18E-05	5.87E-09	0.420
17	68199783	68875761	304	-1.52E-05	4.01E-09	0.810
17	68877746	69242135	206	2.61E-04	2.03E-08	0.067
17	69242932	70147289	397	-5.88E-05	8.52E-09	0.524
17	70148198	70947580	477	3.15E-05	7.99E-09	0.724
17	70948535	71825523	480	1.43E-04	7.55E-09	0.099
17	71829519	72740027	438	-1.86E-05	6.75E-09	0.821
17	72742484	74016834	403	-5.39E-05	5.10E-09	0.450
17	74018640	74909405	368	5.17E-05	5.26E-09	0.475
17	74910943	75471247	314	7.55E-05	5.45E-09	0.306
17	75476450	75842792	220	1.90E-05	4.14E-09	0.768
17	75844316	76445231	219	3.84E-05	3.87E-09	0.536
17	76446689	77093768	322	1.34E-05	5.19E-09	0.852
17	77094717	77468875	306	-2.16E-05	5.96E-09	0.780
17	77471383	78780080	510	1.14E-05	7.59E-09	0.895
17	78780686	79683249	363	-7.98E-05	5.39E-09	0.277
17	79683698	80969550	416	3.00E-05	5.11E-09	0.675
18	349293	906343	394	-2.61E-05	3.88E-09	0.675
18	1940863	2206099	166	-3.03E-05	2.01E-09	0.499
18	906673	1940581	435	-2.37E-05	3.72E-09	0.697
18	2206891	2889826	315	1.83E-05	2.86E-09	0.731
18	2891847	3357120	219	-4.90E-05	2.42E-09	0.319
18	3359237	3861997	218	-1.69E-05	3.24E-09	0.767
18	3862461	4597021	432	3.28E-05	4.58E-09	0.628
18	4601573	5197395	229	5.25E-05	2.29E-09	0.272
18	5198562	5852964	326	-4.74E-05	2.97E-09	0.385
18	5855598	6630394	360	-4.94E-06	3.47E-09	0.933
18	6630840	7373590	413	4.11E-05	4.07E-09	0.519

18	7374066	8440357	509	-1.55E-05	3.99E-09	0.806
18	8440558	9414901	548	1.23E-04	5.28E-09	0.091
18	10465005	11028008	322	6.11E-05	2.39E-09	0.211
18	9415389	10462537	665	3.63E-05	7.49E-09	0.675
18	11028129	11529011	226	1.08E-05	2.19E-09	0.818
18	11530996	12737037	467	-8.66E-06	3.30E-09	0.880
18	12737582	14358413	604	-8.21E-06	4.36E-09	0.901
18	18527782	21622994	744	2.11E-05	6.38E-09	0.791
18	21623416	22745505	572	3.74E-05	4.86E-09	0.592
18	22746547	24088696	488	4.69E-06	3.56E-09	0.937
18	24088831	24971742	490	-4.65E-05	3.04E-09	0.399
18	26874132	28037427	397	-2.66E-06	2.42E-09	0.957
18	28037840	28560707	235	2.31E-06	1.82E-09	0.957
18	24972728	26873513	697	-1.10E-04	4.55E-09	0.103
18	28561634	29563172	480	1.87E-05	3.38E-09	0.748
18	31777336	32456417	204	-3.62E-05	1.16E-09	0.287
18	29564408	31774120	674	-3.33E-05	3.78E-09	0.588
18	32457437	33791804	485	-4.85E-05	3.10E-09	0.384
18	35073616	35837704	322	-8.78E-06	2.06E-09	0.847
18	33792671	35072855	572	-6.04E-06	3.58E-09	0.920
18	35838871	36364691	241	1.90E-05	2.36E-09	0.696
18	36365021	37682827	411	-1.28E-05	2.49E-09	0.797
18	37683967	38766853	525	7.14E-05	4.39E-09	0.281
18	38767938	39891215	366	-2.42E-05	2.27E-09	0.612
18	42606091	43295247	413	3.80E-05	3.05E-09	0.491
18	43295347	43881224	239	1.05E-05	1.34E-09	0.775
18	39892281	42606024	955	1.00E-04	5.95E-09	0.193
18	45314528	46208355	424	8.13E-05	3.98E-09	0.197
18	43881706	45313390	680	-7.66E-06	4.49E-09	0.909
18	46209339	47262925	483	3.96E-05	3.62E-09	0.510
18	47263681	47814821	330	2.74E-05	2.08E-09	0.549
18	47816777	48784027	395	-4.27E-06	2.51E-09	0.932
18	48784953	50151479	574	4.14E-05	4.46E-09	0.535
18	50152229	51059341	375	-4.30E-06	2.26E-09	0.928
18	51059850	51952586	345	1.32E-06	5.23E-09	0.985
18	51953945	52707502	198	-5.32E-05	1.80E-09	0.211
18	52709949	53763665	408	-8.20E-05	3.59E-09	0.171

18	53764157	55089715	500	-3.17E-05	3.30E-09	0.581
18	55090919	55808073	393	3.93E-05	3.53E-09	0.509
18	55808692	56752598	490	3.67E-06	3.97E-09	0.953
18	56754050	57504565	479	6.87E-05	3.89E-09	0.271
18	57505073	58816387	484	5.38E-07	3.21E-09	0.992
18	58817565	59585320	377	5.33E-05	3.30E-09	0.353
18	59585959	60822264	549	-1.63E-05	3.84E-09	0.793
18	60823554	61439643	276	9.68E-06	1.87E-09	0.823
18	61441801	62075853	293	-1.67E-05	1.70E-09	0.685
18	62078305	63110788	403	-2.06E-05	2.85E-09	0.700
18	63111628	64250279	397	-2.21E-05	2.44E-09	0.655
18	64251305	65830031	735	4.37E-05	5.78E-09	0.565
18	65830606	66404071	323	4.94E-05	2.22E-09	0.294
18	66404118	67569547	516	-5.68E-06	4.76E-09	0.934
18	67574191	69038112	511	-3.33E-05	4.52E-09	0.620
18	69039235	70094707	484	-8.12E-05	4.21E-09	0.211
18	70094975	70717286	329	-1.87E-05	2.77E-09	0.722
18	70719886	71243399	284	2.83E-05	2.56E-09	0.575
18	71244692	71892202	389	-5.10E-05	3.95E-09	0.417
18	71892443	72519795	346	2.64E-05	2.61E-09	0.605
18	72521276	73897594	733	-5.95E-05	5.69E-09	0.430
18	73898846	75028501	623	1.18E-05	6.52E-09	0.884
18	75029584	75473982	288	7.06E-05	2.61E-09	0.167
18	75474664	75975253	233	-3.10E-05	2.27E-09	0.515
18	75976567	77151520	503	-1.17E-04	5.77E-09	0.125
18	77151867	78017074	335	-6.37E-05	2.63E-09	0.214
19	157892	757412	227	-2.46E-05	4.24E-09	0.705
19	759261	1089241	159	-1.10E-05	2.92E-09	0.839
19	1090803	1920712	264	1.08E-04	5.46E-09	0.145
19	1922476	2815372	342	-3.46E-05	5.23E-09	0.632
19	2816339	3301333	213	2.64E-05	3.50E-09	0.656
19	3304779	4347573	350	3.33E-05	7.72E-09	0.704
19	4349416	5191547	336	1.11E-05	4.52E-09	0.869
19	5192372	5766090	218	-4.45E-05	4.03E-09	0.484
19	5767585	6612779	264	-3.33E-05	3.90E-09	0.594
19	6614637	7309688	316	5.72E-06	5.27E-09	0.937
19	7312727	8684707	486	5.16E-06	7.83E-09	0.953

19	9013231	10029966	390	-3.16E-05	4.29E-09	0.630
19	10030690	11279257	438	5.11E-05	1.53E-08	0.679
19	11280183	12497411	332	-6.85E-05	4.01E-09	0.280
19	12500149	13903388	408	-3.94E-05	5.48E-09	0.594
19	15553878	16251518	444	2.24E-05	5.12E-09	0.754
19	13906229	15549939	595	-6.85E-05	8.29E-09	0.452
19	17944900	18506666	242	-3.31E-05	3.65E-09	0.584
19	16252072	17942927	622	-7.57E-05	8.91E-09	0.423
19	19873640	20520392	140	-1.94E-05	1.67E-09	0.636
19	18506815	19873269	429	3.12E-05	4.88E-09	0.655
19	21379657	22036399	158	-5.61E-06	1.71E-09	0.892
19	22037866	22803640	245	-2.68E-05	2.68E-09	0.605
19	22805247	24059424	282	7.63E-06	2.82E-09	0.886
19	24059507	28555771	270	2.30E-06	3.00E-09	0.966
19	28556284	28917565	189	-1.66E-05	2.08E-09	0.716
19	29318443	30113252	466	-2.07E-05	5.65E-09	0.783
19	30113991	30858084	259	1.79E-05	3.84E-09	0.773
19	30858965	32136133	462	-1.34E-04	5.73E-09	0.078
19	33764103	34067891	139	3.14E-05	2.00E-09	0.483
19	32145233	33763475	600	2.17E-05	7.28E-09	0.799
19	34068435	35331436	448	1.30E-05	5.96E-09	0.866
19	35333176	36045772	365	3.02E-06	5.11E-09	0.966
19	36047098	37454119	411	1.77E-05	4.63E-09	0.795
19	37455512	39370456	574	-1.85E-05	1.14E-08	0.863
19	39371014	39757572	147	2.32E-05	1.88E-09	0.592
19	41442597	41841098	164	7.06E-06	1.87E-09	0.870
19	41843461	43954915	365	-1.20E-04	8.61E-09	0.196
19	43956095	44880657	396	1.61E-05	4.54E-09	0.811
19	39757926	41440863	613	-1.17E-04	7.52E-09	0.177
19	44882157	45255679	171	2.34E-05	2.89E-09	0.663
19	45259002	46433733	399	-9.81E-05	7.83E-09	0.268
19	46433891	47333204	330	-2.94E-05	5.29E-09	0.686
19	47333497	48509893	371	-7.11E-05	4.85E-09	0.307
19	48697961	49485758	337	-1.48E-04	5.00E-09	0.037
19	49491035	49923166	159	8.38E-08	1.83E-09	0.998
19	49925208	50774141	253	-5.47E-05	3.47E-09	0.352
19	50774581	51331589	225	2.33E-05	3.67E-09	0.701

19	52217405	52604625	221	-1.50E-05	2.70E-09	0.773
19	52606936	53107086	222	-7.83E-05	3.71E-09	0.199
19	51332061	52216563	534	-2.49E-05	3.51E-08	0.894
19	53107937	53537923	157	2.69E-05	2.89E-09	0.616
19	53538505	53899797	201	-2.22E-05	3.13E-09	0.692
19	54099626	54702450	223	1.11E-05	5.49E-09	0.881
19	54704266	55174498	210	4.06E-05	3.87E-09	0.514
19	55175009	55791380	259	-5.78E-05	4.29E-09	0.378
19	55794266	56364684	248	-1.88E-05	5.51E-09	0.800
19	56365323	56898342	291	-4.00E-05	5.11E-09	0.576
19	56898853	57950377	491	3.74E-05	7.14E-09	0.658
19	57952035	58531715	259	-2.06E-05	2.67E-09	0.691
19	58532756	59118784	238	-1.67E-05	2.83E-09	0.754
20	246392	887800	377	-3.54E-05	6.38E-09	0.658
20	888523	1445821	369	-1.45E-05	4.64E-09	0.831
20	1446580	1897442	256	-1.47E-05	2.56E-09	0.772
20	3083151	3655085	235	4.04E-05	2.18E-09	0.387
20	3655943	4051325	176	-3.24E-05	2.19E-09	0.489
20	1899473	3081680	692	2.57E-05	6.65E-09	0.753
20	4053020	4701211	402	-1.97E-05	5.22E-09	0.785
20	4701706	5304961	360	1.31E-05	3.76E-09	0.831
20	5305400	6636139	709	-1.68E-04	7.35E-09	0.050
20	6637234	7065163	285	1.56E-05	3.35E-09	0.788
20	7066818	8600105	764	-7.26E-05	6.32E-09	0.361
20	8601114	8881586	198	2.21E-05	1.45E-09	0.561
20	8884009	9745450	471	-4.41E-05	4.19E-09	0.495
20	11014704	11534388	291	4.62E-05	2.28E-09	0.333
20	9746345	11013570	694	-1.30E-05	7.30E-09	0.879
20	11534488	12153352	274	3.88E-05	2.30E-09	0.418
20	12153396	13199755	619	-1.91E-04	5.67E-09	0.011
20	13201177	13666198	193	2.49E-05	1.81E-09	0.558
20	13670311	14929217	486	1.90E-05	4.02E-09	0.764
20	15148231	16150464	705	1.05E-04	7.13E-09	0.215
20	17449713	17905102	299	-2.12E-05	3.43E-09	0.718
20	17906490	18896415	440	-2.85E-05	3.43E-09	0.626
20	18896895	19707457	462	-4.15E-05	4.03E-09	0.513
20	16151566	17449361	878	2.98E-05	7.97E-09	0.738

20	19708351	21676556	819	-3.80E-05	7.18E-09	0.654
20	21679930	22779512	406	3.11E-06	2.61E-09	0.951
20	22779865	23568490	399	1.46E-05	3.06E-09	0.792
20	23568495	24053655	219	2.61E-05	1.69E-09	0.526
20	24054931	25019099	416	5.95E-06	3.05E-09	0.914
20	26319535	30570889	234	3.63E-05	1.49E-09	0.347
20	25020047	26319418	309	-4.36E-05	2.10E-09	0.342
20	30574455	31613985	334	-5.62E-05	2.33E-09	0.244
20	33885155	34891416	221	2.22E-05	1.29E-09	0.538
20	34892880	35892530	205	-3.29E-06	1.57E-09	0.934
20	31615242	33883015	705	-5.82E-05	4.18E-09	0.368
20	35899774	36904375	395	-7.37E-05	2.87E-09	0.169
20	36905894	37875045	354	2.58E-06	2.69E-09	0.960
20	37875309	38431408	230	5.81E-09	1.52E-09	1.000
20	38435302	39453533	470	-7.72E-06	3.87E-09	0.901
20	39455778	40584825	395	-1.64E-05	3.30E-09	0.775
20	40585126	41194048	393	-1.01E-06	3.73E-09	0.987
20	41195111	42188284	488	-1.87E-05	4.80E-09	0.788
20	42188914	43257406	508	2.28E-05	4.52E-09	0.734
20	44960997	45649369	359	4.63E-05	3.28E-09	0.419
20	45650139	46540102	374	8.29E-05	4.03E-09	0.191
20	43259306	44960236	711	-9.12E-05	5.45E-09	0.216
20	48081983	48655016	227	-1.11E-05	4.17E-09	0.863
20	48658113	49385779	312	-1.06E-05	3.29E-09	0.853
20	46540587	48080850	770	-5.81E-05	6.34E-09	0.466
20	49387520	49945761	256	-3.61E-05	3.69E-09	0.553
20	49948016	50633472	360	-1.52E-05	3.74E-09	0.804
20	50635347	51389135	319	-3.07E-05	2.52E-09	0.541
20	51389670	51884594	263	-3.05E-05	2.77E-09	0.562
20	51885269	52525657	292	2.94E-05	5.53E-09	0.693
20	52526840	53406846	485	5.17E-05	4.80E-09	0.456
20	53408035	54321635	409	4.75E-05	3.45E-09	0.419
20	54321811	55431836	547	4.59E-05	5.08E-09	0.519
20	56216001	56679942	290	-6.77E-06	3.35E-09	0.907
20	55434683	56214465	499	9.10E-05	5.99E-09	0.240
20	56679960	57319010	352	-5.57E-05	3.49E-09	0.345
20	57320545	58295563	478	4.26E-05	5.70E-09	0.573

20	59361382	59892000	354	3.70E-05	4.13E-09	0.565
20	58296350	59361064	553	-3.55E-05	5.08E-09	0.619
20	59892231	61091395	657	-9.31E-05	1.32E-08	0.417
20	61301855	62119318	401	-2.41E-05	4.82E-09	0.729
20	62119875	62962870	340	-2.14E-04	1.07E-08	0.039
21	15451950	16139409	423	-9.16E-05	3.93E-09	0.144
21	18457828	19151566	246	-1.22E-04	3.04E-09	0.027
21	16140244	17301757	441	8.91E-05	3.90E-09	0.153
21	17305471	18457656	408	-3.74E-05	4.87E-09	0.592
21	19151940	20198061	542	-3.10E-05	4.86E-09	0.656
21	21022840	21905609	384	1.32E-05	3.95E-09	0.834
21	20199390	21022168	395	-3.03E-05	3.49E-09	0.608
21	24695869	25083563	190	-2.95E-06	1.74E-09	0.944
21	23270321	24695405	541	-3.41E-05	4.74E-09	0.621
21	21905658	23268216	670	1.96E-05	5.86E-09	0.798
21	25899516	26465774	241	-9.08E-06	2.15E-09	0.845
21	25090174	25898082	385	-4.89E-05	3.18E-09	0.386
21	26467796	27776736	496	2.57E-05	4.26E-09	0.694
21	27777181	28205789	289	2.17E-05	2.82E-09	0.682
21	28206883	28705495	309	3.20E-05	3.15E-09	0.568
21	28705732	29515396	352	1.07E-07	3.45E-09	0.999
21	30845873	32211778	531	4.29E-05	3.91E-09	0.492
21	29515884	30844467	550	-4.03E-05	3.66E-09	0.506
21	33273162	33988973	358	-3.10E-05	3.16E-09	0.581
21	32213678	33270766	453	-1.36E-05	4.23E-09	0.835
21	33991368	35219895	507	-1.68E-04	1.08E-08	0.106
21	35220001	36188046	457	-8.84E-05	1.78E-08	0.508
21	37870518	38429082	265	-1.36E-06	2.23E-09	0.977
21	36190040	37303320	549	1.41E-05	4.78E-09	0.838
21	37305159	37868817	300	4.88E-05	2.42E-09	0.321
21	38429389	39248065	402	-2.09E-05	2.89E-09	0.697
21	39250321	40115483	422	4.53E-06	4.13E-09	0.944
21	40116044	41095241	608	-1.07E-04	4.97E-09	0.129
21	41260363	41705756	344	-2.11E-05	3.41E-09	0.718
21	41707453	42543663	491	6.96E-05	4.61E-09	0.305
21	42545250	43342046	530	-2.01E-04	1.05E-08	0.050
21	44690856	45598522	356	-8.85E-08	2.79E-09	0.999

21	45603905	46174491	279	3.95E-06	3.37E-09	0.946
21	46174981	46740019	297	4.56E-05	2.55E-09	0.367
21	46741287	47491264	334	-2.41E-05	2.91E-09	0.655
21	47491975	48119635	293	-3.01E-05	2.21E-09	0.523
21	43343190	44632513	845	-2.03E-05	8.37E-09	0.824
22	17187276	17569017	168	-2.33E-05	1.70E-09	0.571
22	18333902	19138442	298	-1.34E-05	2.82E-09	0.800
22	17569439	18333467	393	5.86E-05	4.90E-09	0.402
22	19139368	20000428	424	-2.55E-05	4.63E-09	0.707
22	20001006	20952650	271	-1.03E-04	3.68E-09	0.090
22	20952972	21863717	295	-9.15E-06	2.33E-09	0.850
22	21866569	23015302	368	-5.91E-06	3.33E-09	0.918
22	23016570	23691564	208	2.00E-05	1.76E-09	0.634
22	23697301	24370194	306	1.27E-06	3.47E-09	0.983
22	24375019	25416292	358	4.76E-06	2.94E-09	0.930
22	25419524	26113333	226	3.38E-06	2.27E-09	0.943
22	26123092	26735929	447	-7.75E-05	4.76E-09	0.261
22	26736812	27294543	387	-1.89E-05	4.25E-09	0.772
22	27295234	27653424	353	-1.36E-05	4.53E-09	0.840
22	29456360	30604102	391	1.51E-05	2.85E-09	0.778
22	30605601	31534127	380	-2.46E-05	3.40E-09	0.673
22	31535872	32324140	238	-5.00E-06	1.49E-09	0.897
22	32324692	32856999	277	-1.78E-05	2.47E-09	0.720
22	27654838	29454477	731	-2.04E-04	7.81E-09	0.021
22	32857285	33511391	411	-3.73E-05	4.25E-09	0.567
22	33512106	33896495	178	6.29E-06	2.07E-09	0.890
22	35865218	36131498	134	-4.26E-05	1.59E-09	0.286
22	34983275	35862655	468	7.86E-06	3.26E-09	0.891
22	36132263	36922822	314	3.08E-05	3.85E-09	0.620
22	33898114	34982457	618	-5.74E-06	5.21E-09	0.937
22	36923144	37558356	398	2.38E-06	4.32E-09	0.971
22	38473398	39302783	269	1.65E-05	2.08E-09	0.717
22	37560592	38467784	418	-3.47E-05	3.87E-09	0.577
22	39304509	40092866	320	3.74E-05	2.88E-09	0.487
22	40095174	41405753	318	-3.88E-05	3.41E-09	0.507
22	43187900	43645335	232	-7.89E-05	1.76E-08	0.552
22	41406919	42870441	427	1.95E-05	4.12E-09	0.761

22	43646241	44317084	380	-1.41E-05	3.16E-09	0.803
22	44317416	44818986	414	1.28E-04	4.93E-09	0.069
22	45444397	45895495	269	3.58E-06	2.79E-09	0.946
22	44820796	45443845	418	-4.41E-05	5.41E-09	0.548
22	45895856	46512413	261	1.16E-05	3.10E-09	0.835
22	46513376	47106715	271	-6.70E-05	2.96E-09	0.218
22	47106800	47937655	434	-3.26E-06	3.92E-09	0.958
22	47937891	48479731	373	-4.88E-05	3.89E-09	0.434
22	49082979	49521080	313	-4.43E-05	4.37E-09	0.503
22	49522184	49751418	246	-1.82E-05	3.30E-09	0.752
22	48481280	49081753	463	1.09E-05	5.92E-09	0.887
22	49751512	50488153	335	3.34E-05	3.26E-09	0.558
22	50488995	51237713	281	3.49E-05	3.01E-09	0.525
**Prostate cancer and COVID-19 critical illness**						
1	1103150	2320702	355	-3.51E-05	7.04E-09	0.676
1	2321099	3065568	234	-4.75E-05	8.73E-09	0.611
1	3066761	3679461	289	-2.03E-04	1.32E-08	0.078
1	3679775	4749076	631	-1.59E-04	1.51E-08	0.196
1	4750013	5000086	168	4.29E-06	4.12E-09	0.947
1	5000970	5471517	301	-1.41E-05	7.28E-09	0.869
1	5472626	6133700	338	2.18E-05	7.47E-09	0.801
1	6134990	7245669	446	4.12E-05	8.17E-09	0.648
1	7245922	7604778	205	-3.49E-06	4.56E-09	0.959
1	7604831	9067142	512	1.09E-04	9.89E-09	0.272
1	9067673	9959051	318	1.77E-04	1.39E-08	0.134
1	9959748	11774956	583	9.76E-05	1.04E-08	0.339
1	11776196	12147753	192	-3.30E-05	3.35E-09	0.569
1	12150385	14625524	723	9.10E-05	1.75E-08	0.492
1	14626896	15423481	421	-7.63E-05	1.12E-08	0.470
1	15424441	17123862	613	-1.06E-04	1.32E-08	0.354
1	17176635	17872659	333	-1.59E-05	7.39E-09	0.853
1	17874843	18484945	340	-2.06E-05	7.48E-09	0.812
1	18486272	20022063	798	-1.27E-04	1.77E-08	0.340
1	20023833	20763899	370	3.68E-06	1.02E-08	0.971
1	20765245	22634131	796	-1.09E-04	1.62E-08	0.393
1	22636103	23082667	208	-1.15E-04	5.64E-09	0.125
1	23085227	24527501	489	-3.25E-05	8.46E-09	0.724

1	24528210	25305673	316	3.14E-05	7.23E-09	0.712
1	25305783	26976424	610	-7.80E-05	1.49E-08	0.523
1	26981479	28160199	204	1.88E-05	3.17E-09	0.738
1	28168872	29181525	242	-9.06E-05	3.51E-09	0.127
1	29184254	30792409	733	-8.75E-05	1.37E-08	0.454
1	30792984	32042421	610	2.71E-05	9.13E-09	0.776
1	32042540	34016219	485	-4.45E-06	9.10E-09	0.963
1	34017349	34797348	442	-4.89E-05	9.06E-09	0.607
1	34798014	35364965	274	-2.44E-05	6.81E-09	0.767
1	35366056	38196841	760	9.76E-05	1.38E-08	0.407
1	38459210	39535259	434	-6.89E-05	1.05E-08	0.501
1	39537291	40933221	445	2.87E-04	7.77E-09	0.001
1	40934284	42166782	487	-1.64E-05	8.57E-09	0.859
1	42168539	43365094	396	-7.93E-05	5.91E-09	0.302
1	43365565	44482312	463	-1.65E-04	6.90E-09	0.046
1	44483704	46895641	764	8.87E-05	9.53E-09	0.363
1	46897698	47999344	385	2.31E-05	5.70E-09	0.760
1	48000156	50640662	789	3.39E-05	1.20E-08	0.757
1	50641944	53116453	445	9.50E-05	6.58E-09	0.242
1	53117066	54197141	419	1.13E-05	7.19E-09	0.894
1	54197688	55012825	295	1.53E-05	6.59E-09	0.850
1	55012881	55479198	250	3.07E-05	5.70E-09	0.685
1	55479967	56413577	485	1.93E-04	1.43E-08	0.107
1	56414730	57585670	599	4.89E-05	1.09E-08	0.639
1	57585980	58535140	555	-6.22E-05	9.02E-09	0.513
1	58536417	59306823	412	6.41E-05	7.05E-09	0.445
1	59307290	59736866	187	9.84E-06	4.09E-09	0.878
1	59739179	61257170	537	-9.05E-05	9.69E-09	0.358
1	61258378	62107806	382	2.94E-04	1.22E-08	0.008
1	62108681	63376404	518	1.66E-04	9.33E-09	0.086
1	63379031	65008206	795	3.69E-05	1.54E-08	0.766
1	65010679	66773349	699	-7.61E-05	1.23E-08	0.493
1	66774350	67760291	536	4.87E-05	8.72E-09	0.602
1	67761365	69441414	724	-1.05E-04	1.43E-08	0.381
1	69441920	70486660	328	8.57E-05	5.96E-09	0.267
1	70490925	71818029	501	4.80E-05	8.91E-09	0.611
1	71822765	74326378	642	3.42E-05	9.23E-09	0.722

1	74326484	75992462	562	-3.52E-05	8.74E-09	0.706
1	75992778	76476360	183	-2.85E-05	2.50E-09	0.569
1	76477207	77139859	372	-6.84E-05	7.74E-09	0.436
1	77141039	79111862	685	2.08E-05	1.21E-08	0.850
1	79113455	80341320	482	3.12E-06	7.91E-09	0.972
1	80341732	81056946	241	-3.77E-06	4.16E-09	0.953
1	81058295	82883310	839	-2.41E-04	1.69E-08	0.064
1	82884721	83990906	440	1.92E-05	7.58E-09	0.826
1	83991748	84728665	328	-1.70E-05	5.14E-09	0.813
1	84729134	85940249	595	-1.27E-05	1.18E-08	0.907
1	85940875	86549498	268	1.27E-05	4.39E-09	0.848
1	86549569	87652502	416	-3.11E-05	7.34E-09	0.717
1	87654606	89447978	631	5.45E-06	1.59E-08	0.965
1	89451664	91866856	883	-1.06E-04	1.58E-08	0.397
1	91867046	93351477	560	1.50E-05	8.99E-09	0.875
1	93352491	94451007	318	-3.55E-05	4.38E-09	0.592
1	94452062	95199048	406	-5.63E-06	7.63E-09	0.949
1	95199771	96149941	435	5.99E-06	8.37E-09	0.948
1	96150318	97457893	467	-3.58E-05	7.26E-09	0.674
1	97458722	98562260	516	-1.00E-04	6.88E-09	0.226
1	98564736	100064599	575	1.56E-04	1.09E-08	0.136
1	100065413	101682573	614	1.78E-04	8.73E-09	0.056
1	101683165	102039858	221	-5.81E-05	4.35E-09	0.378
1	102040268	102893372	341	-2.09E-06	6.18E-09	0.979
1	102894567	104369301	287	5.86E-05	5.13E-09	0.413
1	104373157	105192916	269	-1.14E-05	5.00E-09	0.872
1	105193022	106641877	398	-2.36E-05	6.83E-09	0.775
1	106642705	107432265	291	-8.59E-05	4.97E-09	0.223
1	107432576	109075191	691	7.93E-05	1.44E-08	0.508
1	109075561	110301260	462	3.42E-05	7.95E-09	0.702
1	110302505	111738116	679	-3.84E-05	1.55E-08	0.758
1	111739749	112560278	566	-6.12E-05	1.17E-08	0.572
1	112561693	114451307	681	-7.28E-05	9.74E-09	0.461
1	114451386	115878242	636	-8.60E-05	1.15E-08	0.423
1	115879821	116884431	398	4.38E-05	7.35E-09	0.610
1	116884519	118837474	648	8.73E-05	1.20E-08	0.425
1	118837888	119792751	365	1.13E-05	5.86E-09	0.882

1	119793605	120347239	234	3.81E-05	5.82E-09	0.618
1	144998563	147347713	448	2.44E-05	7.46E-09	0.778
1	147348339	151830844	597	-3.95E-05	2.13E-08	0.787
1	151832901	152535021	216	6.94E-05	2.79E-09	0.189
1	152535586	153163377	278	1.91E-05	3.40E-09	0.744
1	153165450	154544651	506	1.18E-04	8.67E-09	0.205
1	154547292	156334116	549	6.58E-05	7.76E-08	0.813
1	156335111	157154186	313	2.02E-04	1.27E-08	0.073
1	157154324	158867538	819	3.07E-05	1.54E-08	0.805
1	158870085	160648549	764	-1.30E-04	1.64E-08	0.311
1	160649926	161973976	510	8.34E-05	9.30E-09	0.387
1	161974584	162995355	563	-2.87E-05	1.06E-08	0.780
1	163000394	164410254	676	-1.42E-04	1.08E-08	0.172
1	164412847	165190876	436	-5.64E-05	1.05E-08	0.582
1	165191702	166363258	604	-1.29E-05	1.21E-08	0.906
1	166364063	167158927	484	-1.35E-05	8.09E-09	0.880
1	167159608	168168773	462	9.84E-05	9.01E-09	0.300
1	168169452	169077127	489	-8.40E-05	1.08E-08	0.418
1	169078572	169521853	193	-4.94E-05	2.62E-09	0.335
1	169522554	170372503	446	-6.98E-05	6.94E-09	0.403
1	170375663	170914261	181	-1.86E-05	3.28E-09	0.745
1	170915133	172647523	864	8.87E-05	1.30E-08	0.437
1	172648956	175384090	908	-3.87E-07	1.26E-08	0.997
1	175385076	176416712	369	-5.47E-05	7.45E-09	0.526
1	176418578	177431255	438	-3.27E-05	7.14E-09	0.699
1	177432237	178940717	621	5.78E-05	1.04E-08	0.570
1	178943012	179676274	338	-8.57E-06	4.78E-09	0.901
1	179678188	180567596	397	-1.11E-04	8.70E-09	0.234
1	180568720	181311800	309	4.74E-06	4.99E-09	0.946
1	181313404	182293001	421	-2.23E-05	8.83E-09	0.812
1	182294372	183796074	577	1.18E-04	8.51E-09	0.202
1	183796618	185511423	737	-2.23E-04	1.15E-08	0.038
1	185512838	187847742	807	4.65E-05	1.27E-08	0.680
1	187848898	189756187	598	4.87E-05	7.93E-09	0.585
1	189758772	190107648	158	1.05E-05	2.35E-09	0.828
1	190107883	191432168	472	2.98E-05	6.71E-09	0.716
1	191433917	192444256	330	-6.83E-05	4.93E-09	0.330

1	192452631	193504005	346	3.02E-05	4.95E-09	0.667
1	193505535	194000243	197	-4.19E-06	2.95E-09	0.939
1	194000953	194733444	212	-1.54E-05	3.10E-09	0.782
1	194734415	195155161	176	-8.33E-06	2.53E-09	0.869
1	195155293	195995510	312	-5.20E-05	5.46E-09	0.482
1	195995948	197309603	342	8.94E-05	5.19E-09	0.215
1	197310096	198540028	417	-5.04E-05	6.19E-09	0.522
1	198540418	199578700	369	-3.19E-05	6.48E-09	0.692
1	199580285	201070422	658	-1.04E-04	1.17E-08	0.338
1	201070740	201365889	257	6.04E-05	5.15E-09	0.400
1	201366509	202820077	608	-5.46E-05	1.22E-08	0.621
1	202820815	203979519	487	1.18E-04	1.16E-08	0.271
1	203980019	205458538	791	-4.98E-04	3.47E-08	0.008
**1**	**205459511**	**206705609**	**334**	**5.36E-04**	**1.41E-08**	**0.000**
1	206706419	208158787	548	4.59E-05	9.52E-09	0.638
1	210741816	211763053	432	-3.07E-05	8.78E-09	0.743
1	208160220	210738604	1287	-1.16E-04	2.51E-08	0.464
1	211763754	213578006	640	1.90E-04	1.24E-08	0.088
1	213578592	215128675	755	1.22E-05	1.50E-08	0.921
1	215129087	216672805	623	3.81E-05	1.07E-08	0.712
1	216673326	217575665	684	-8.16E-06	1.40E-08	0.945
1	217577346	218860068	519	4.60E-05	1.09E-08	0.659
1	218860456	220068681	530	-9.21E-06	8.52E-09	0.920
1	220069681	221856899	640	-9.23E-07	1.11E-08	0.993
1	221857359	223630391	645	-2.31E-04	1.15E-08	0.031
1	223630619	224434769	280	-3.09E-05	5.18E-09	0.668
1	224435934	226143348	569	1.14E-06	9.60E-09	0.991
1	226146348	226808294	242	-1.73E-05	4.60E-09	0.799
1	226808697	228100891	557	1.26E-04	8.95E-09	0.181
1	228101119	229075210	320	-2.81E-04	1.05E-08	0.006
1	229076157	230477970	634	8.44E-05	1.19E-08	0.439
1	230479324	231318678	503	5.08E-05	1.10E-08	0.628
1	231321215	232382234	476	-4.50E-06	8.46E-09	0.961
1	232384356	232949914	349	3.67E-05	7.20E-09	0.665
1	232950888	233750725	429	2.94E-05	8.97E-09	0.757
1	233753991	234206593	224	-3.95E-06	5.00E-09	0.955
1	234209087	235772624	700	2.01E-04	1.56E-08	0.108

1	235773790	236536723	313	3.79E-05	6.29E-09	0.632
1	236537770	237493854	565	2.57E-05	1.05E-08	0.802
1	237495080	239297872	909	1.10E-04	1.94E-08	0.429
1	239299272	240562764	671	4.53E-05	1.45E-08	0.707
1	240563367	241178287	350	8.64E-06	9.97E-09	0.931
1	241178944	241766551	356	-4.08E-05	8.84E-09	0.664
1	241768617	242697031	496	-1.64E-05	1.19E-08	0.881
1	242700803	244105053	454	8.00E-05	6.91E-09	0.336
1	244106113	244927751	381	3.02E-05	9.20E-09	0.753
1	244929262	246606531	849	4.60E-05	1.84E-08	0.734
1	246606926	246989171	160	1.06E-05	3.31E-09	0.853
1	246991094	247838628	369	-7.00E-05	7.93E-09	0.432
1	247838870	248585259	279	-2.08E-05	4.58E-09	0.759
2	11320	1158981	538	-1.71E-04	1.05E-08	0.095
2	1159982	1681720	218	4.07E-05	5.12E-09	0.569
2	1683187	3003071	697	1.05E-04	1.76E-08	0.427
2	3003472	4148069	588	-2.82E-05	1.36E-08	0.808
2	4148784	5017172	422	-6.95E-05	9.24E-09	0.470
2	5118816	6361343	633	-3.92E-05	1.69E-08	0.763
2	6363082	7322437	567	-1.01E-04	1.31E-08	0.377
2	7323039	8980346	697	-6.48E-05	1.64E-08	0.612
2	8980472	10440987	657	-1.99E-04	1.86E-08	0.144
2	10442541	10879340	235	2.06E-04	7.74E-09	0.019
2	10881867	11652586	376	1.32E-04	1.15E-08	0.216
2	11652795	12720977	565	-5.93E-05	1.57E-08	0.636
2	12723749	14648908	706	6.21E-05	1.41E-08	0.601
2	14649571	15720775	452	-2.84E-05	7.63E-09	0.745
2	15721423	16549199	386	-1.07E-04	9.64E-09	0.277
2	16549538	18344242	767	1.40E-04	1.54E-08	0.258
2	18344821	18646822	144	-5.13E-06	2.93E-09	0.924
2	18647868	20059542	691	2.21E-05	1.44E-08	0.854
2	20062043	22427190	824	-6.85E-05	3.69E-08	0.721
2	22428680	23855977	652	-8.47E-05	1.38E-08	0.470
2	23857184	25485735	454	1.17E-04	7.12E-09	0.165
2	25486770	26894217	438	3.28E-05	8.46E-09	0.721
2	26894589	28597305	482	-9.66E-06	6.83E-09	0.907
2	28597624	29484922	455	5.81E-05	9.20E-09	0.545

2	29486078	29987735	338	-2.47E-05	6.71E-09	0.763
2	29988753	31009977	499	7.02E-05	1.06E-08	0.496
2	31010212	32489851	591	-2.13E-05	1.07E-08	0.837
2	32493210	33361425	336	1.75E-05	6.95E-09	0.834
2	33362643	34279720	532	-1.26E-04	1.16E-08	0.242
2	34280335	35607183	528	2.55E-05	1.01E-08	0.800
2	35608923	36121342	278	9.99E-05	5.04E-09	0.159
2	36122006	37004185	552	-1.08E-04	1.33E-08	0.349
2	37008066	38882278	873	-1.11E-04	1.94E-08	0.423
2	38882577	40077971	310	-8.07E-07	4.72E-09	0.991
2	40080343	40625847	349	-1.25E-04	6.89E-09	0.131
2	40626336	41381901	284	-7.77E-05	5.00E-09	0.272
2	41382889	42087411	359	-3.46E-05	6.46E-09	0.666
2	42088016	42947335	404	-4.55E-05	7.00E-09	0.586
2	42949616	43307776	218	-5.64E-05	8.88E-09	0.550
2	43309247	44048346	326	6.54E-06	2.10E-08	0.964
2	44049120	45194289	539	-7.56E-05	9.98E-09	0.449
2	45195022	45562814	268	-3.62E-05	8.13E-09	0.688
2	45563613	47052067	879	6.07E-05	2.07E-08	0.673
2	47052788	47782756	369	-2.61E-05	9.99E-09	0.794
2	47782836	49112501	506	-3.43E-05	1.13E-08	0.747
2	49113132	50757671	842	-1.78E-05	1.62E-08	0.889
2	50758657	51667413	400	1.18E-05	7.51E-09	0.892
2	51668142	53351493	834	2.02E-05	1.58E-08	0.872
2	53352175	54599930	553	-4.77E-05	1.09E-08	0.649
2	54600556	56158646	724	-9.55E-05	1.42E-08	0.423
2	56160647	56599354	240	-4.04E-05	6.28E-09	0.611
2	56600739	57938354	491	-8.30E-05	1.03E-08	0.413
2	57939866	60449069	1013	-7.84E-05	2.09E-08	0.587
2	60449710	62420656	657	1.26E-04	1.12E-08	0.233
2	62425639	64623935	687	1.38E-05	2.78E-08	0.934
2	64626895	65226463	256	-2.19E-05	6.06E-09	0.778
2	65228236	66374803	558	6.69E-05	1.10E-08	0.524
2	66375016	67978287	797	-5.78E-05	1.70E-08	0.657
2	67981487	68871573	395	-4.84E-05	7.43E-09	0.574
2	68871710	70753882	813	-9.68E-05	1.43E-08	0.418
2	70756057	71704491	467	-2.06E-05	1.03E-08	0.839

2	71704844	73172972	470	3.09E-05	9.25E-09	0.748
2	73174848	74121000	253	3.41E-05	3.58E-09	0.569
2	74121278	76615798	1069	-4.47E-05	2.13E-08	0.759
2	76616819	77384991	368	4.72E-05	7.43E-09	0.584
2	77386522	79007966	561	3.39E-05	1.18E-08	0.754
2	80804991	81588311	230	-1.23E-05	3.84E-09	0.842
2	79008155	80804284	1066	2.64E-05	2.32E-08	0.862
2	81588482	83604057	600	-2.26E-05	8.16E-09	0.803
2	83604796	85283713	481	5.70E-05	7.62E-09	0.514
2	85284353	86559358	571	-8.61E-06	1.45E-08	0.943
2	86562902	89135764	457	-7.39E-05	8.24E-09	0.416
2	92324281	98790763	459	3.58E-05	6.89E-09	0.666
2	98791433	100195879	407	-9.61E-05	5.84E-09	0.209
2	100197638	101338509	453	3.39E-05	7.30E-09	0.692
2	101338644	102688272	529	1.10E-04	1.10E-08	0.294
2	102688495	103262415	357	-5.33E-05	5.62E-09	0.477
2	103264434	104481488	294	6.99E-06	5.34E-09	0.924
2	104485456	105871610	462	3.18E-06	9.50E-09	0.974
2	105872982	107406122	722	6.76E-05	1.66E-08	0.600
2	107406885	108427822	407	1.29E-05	7.46E-09	0.881
2	108427959	110109210	583	4.33E-05	1.06E-08	0.674
2	110109222	112924907	583	6.35E-05	3.27E-08	0.725
2	112924973	114846282	716	-3.91E-05	1.30E-08	0.731
2	114848115	116770829	607	3.43E-05	1.16E-08	0.750
2	116772945	117727045	302	1.34E-05	4.51E-09	0.842
2	117728904	119581280	716	-4.94E-05	1.43E-08	0.679
2	119581439	121070811	505	-4.40E-05	9.46E-09	0.651
2	121073253	123241656	762	-4.64E-05	2.28E-08	0.758
2	123244233	124730999	532	-5.09E-06	9.06E-09	0.957
2	124733246	125735879	428	6.06E-05	8.64E-09	0.515
2	125741434	127372889	519	1.54E-05	8.47E-09	0.867
2	127374341	129310292	757	3.31E-05	1.52E-08	0.788
2	129312188	129864416	215	-7.16E-07	4.63E-09	0.992
2	129864730	131463038	407	-4.94E-05	7.59E-09	0.570
2	131466136	133030349	223	2.32E-05	4.76E-09	0.736
2	133034031	133950661	429	4.61E-05	9.47E-09	0.635
2	133951544	136822267	1073	-5.75E-05	1.87E-08	0.674

2	136825272	138619715	738	3.63E-05	1.48E-08	0.766
2	138620371	139412389	267	6.93E-05	4.55E-09	0.305
2	139413312	140902261	570	-1.17E-05	1.10E-08	0.911
2	140902383	141803496	400	-6.96E-05	7.79E-09	0.430
2	141803602	143254981	699	1.13E-04	1.44E-08	0.346
2	143255262	145301314	617	-5.75E-05	1.55E-08	0.644
2	145303576	147252762	524	-1.74E-05	9.55E-09	0.859
2	147255216	149268931	496	-3.89E-05	9.07E-09	0.683
2	149270710	151184680	609	-1.96E-04	1.19E-08	0.073
2	151191481	152490219	540	-1.01E-04	1.02E-08	0.315
2	152491269	153369892	310	1.13E-06	5.06E-09	0.987
2	153371042	154414456	449	-3.92E-06	9.89E-09	0.969
2	154415112	155208173	297	-1.24E-05	5.90E-09	0.872
2	155209736	156568446	482	3.37E-05	8.73E-09	0.718
2	156568806	158165818	441	1.52E-05	9.28E-09	0.875
2	158173710	159574883	463	-1.66E-05	9.81E-09	0.867
2	159575554	161077823	603	-4.13E-05	1.07E-08	0.690
2	161078891	163358537	677	-1.32E-05	1.04E-08	0.897
2	163359084	165200324	482	7.23E-07	9.73E-09	0.994
2	165202539	167120956	737	3.19E-04	1.43E-08	0.008
2	167123247	168427789	400	-1.91E-05	6.91E-09	0.818
2	168431583	169176766	322	-2.33E-05	7.22E-09	0.784
2	169178092	170973262	788	-6.08E-05	1.54E-08	0.624
2	170973461	172086127	479	-2.50E-05	1.05E-08	0.807
2	172088943	173586201	588	1.05E-04	3.66E-08	0.585
2	173588663	175585616	784	-3.73E-05	1.76E-08	0.779
2	175586510	177326682	628	1.41E-04	1.09E-08	0.177
2	177327202	178551958	557	6.72E-05	1.01E-08	0.503
2	178552788	180138313	563	-1.07E-04	1.01E-08	0.287
2	180139219	180661233	321	1.96E-05	9.36E-09	0.840
2	180662102	181627195	413	-3.23E-06	1.22E-08	0.977
2	181628139	182840114	361	-1.07E-04	6.56E-09	0.188
2	182845657	184442530	601	6.61E-05	1.07E-08	0.522
2	184443166	185277576	257	5.52E-05	4.56E-09	0.413
2	185279087	186731282	380	-1.60E-05	5.92E-09	0.835
2	186731309	188540877	478	4.46E-06	7.19E-09	0.958
2	188542361	190794689	527	2.32E-05	9.05E-09	0.807

2	190797341	191870624	348	-6.70E-05	8.36E-09	0.464
2	191873553	192695107	283	-3.22E-05	6.70E-09	0.694
2	192696255	195109845	440	-2.30E-05	7.02E-09	0.784
2	195117932	197307918	618	6.98E-05	1.10E-08	0.506
2	197308707	198140953	269	-3.82E-05	4.19E-09	0.555
2	198141746	200498970	618	9.24E-05	8.95E-09	0.329
2	200504209	201571624	452	9.74E-05	7.93E-09	0.274
2	201572564	202829668	381	-4.65E-05	7.68E-09	0.596
2	202830132	204814896	524	-5.49E-05	9.30E-09	0.569
2	204815902	206043539	517	-1.75E-05	1.15E-08	0.870
2	206045927	207721890	726	-2.21E-05	1.53E-08	0.858
2	207722425	208639154	388	-1.04E-04	1.23E-08	0.347
2	208641174	209940909	408	-5.85E-05	7.77E-09	0.507
2	209941332	210832981	269	-3.63E-06	4.37E-09	0.956
2	210838781	212377672	605	-2.26E-06	1.15E-08	0.983
2	212377759	212845716	216	-2.50E-05	5.42E-09	0.735
2	212845994	215005050	818	-1.00E-04	1.49E-08	0.411
2	215006557	216448007	604	-1.95E-05	1.18E-08	0.858
2	216449570	217184275	342	-4.12E-05	7.87E-09	0.643
2	217184715	218615634	619	-3.12E-05	1.30E-08	0.784
2	218616633	219671910	400	-2.77E-05	6.24E-09	0.726
2	219675067	220774229	409	4.27E-05	7.34E-09	0.618
2	220775971	221445218	359	-4.63E-05	6.89E-09	0.577
2	221445953	222985126	638	1.50E-05	1.37E-08	0.898
2	222985611	224002780	432	-8.37E-05	9.14E-09	0.382
2	224003069	225832409	735	8.02E-06	1.42E-08	0.946
2	225833758	227531180	618	-2.63E-05	1.07E-08	0.800
2	227532464	228456451	380	-2.14E-05	7.00E-09	0.798
2	228457117	229368530	432	-3.87E-05	9.35E-09	0.689
2	229368812	230282624	395	-8.61E-05	8.35E-09	0.346
2	230283310	231322125	471	1.31E-04	1.01E-08	0.191
2	231322831	232790491	543	-2.66E-05	1.18E-08	0.807
2	232791948	233964632	468	5.15E-05	9.11E-09	0.590
2	233968153	234699046	367	-1.49E-05	5.65E-09	0.843
2	234699366	235626316	630	5.37E-05	1.53E-08	0.665
2	235627087	236619385	515	-3.44E-06	1.44E-08	0.977
2	236619713	238209382	607	8.96E-05	1.29E-08	0.431

2	238209871	239122917	445	4.65E-05	1.03E-08	0.647
2	239123082	239801883	288	4.15E-05	5.98E-09	0.592
2	239832097	240325991	241	-6.83E-05	7.96E-09	0.444
2	240327154	240961852	336	-1.03E-04	9.00E-09	0.276
2	240962194	241907076	460	-1.72E-05	1.12E-08	0.871
2	241909927	243185680	318	8.60E-05	2.05E-08	0.547
3	60197	1128944	484	3.19E-05	1.41E-08	0.789
3	1129028	1527748	338	4.08E-05	1.06E-08	0.691
3	1528147	2423237	600	2.08E-06	1.59E-08	0.987
3	2423999	2769230	240	-8.39E-05	7.63E-09	0.337
3	2769392	3364279	476	7.03E-05	1.68E-08	0.587
3	3364442	4430024	629	-1.86E-05	1.61E-08	0.884
3	4430848	5564422	653	2.50E-06	2.04E-08	0.986
3	5565384	6421659	512	-4.11E-05	1.43E-08	0.731
3	6422814	7509288	573	1.14E-04	1.46E-08	0.345
3	7509715	7991133	371	-3.30E-06	9.10E-09	0.972
3	7991981	9194649	775	-2.06E-04	2.11E-08	0.155
3	9195331	10526438	649	-1.01E-05	1.67E-08	0.938
3	10526803	11220278	388	6.02E-05	1.23E-08	0.587
3	11221721	12858028	673	-1.16E-04	1.57E-08	0.356
3	12859004	13523338	331	-1.88E-04	9.31E-09	0.052
3	13524741	14309204	364	9.53E-06	1.02E-08	0.925
3	14309548	14816364	328	-3.44E-05	1.03E-08	0.735
3	14816745	16661587	812	-4.79E-05	1.96E-08	0.733
3	16662488	18454062	541	-8.85E-05	1.24E-08	0.426
3	18457304	18886128	166	1.53E-04	5.81E-09	0.044
3	18886158	20752492	602	1.12E-04	1.50E-08	0.359
3	20753368	21522703	362	1.19E-04	9.61E-09	0.224
3	21523116	22102707	446	-1.27E-05	1.21E-08	0.908
3	22103146	23122359	464	-5.29E-05	1.33E-08	0.647
3	23123318	23802647	259	2.27E-05	7.19E-09	0.789
3	23803541	25459497	887	4.85E-06	2.31E-08	0.975
3	25460109	26392563	317	4.71E-05	6.90E-09	0.571
3	26393305	27606695	465	-8.51E-05	1.02E-08	0.400
3	27609447	29409547	794	-6.24E-05	2.14E-08	0.670
3	29409589	30716602	619	8.80E-05	1.69E-08	0.498
3	30717661	31720982	438	1.19E-05	9.30E-09	0.902

3	31721774	33011510	531	-6.90E-05	1.63E-08	0.589
3	33012981	33986974	341	6.15E-05	7.44E-09	0.476
3	33988115	35241960	452	2.33E-05	1.03E-08	0.819
3	35243764	36481385	481	-6.48E-05	1.08E-08	0.533
3	36484375	38078255	626	1.49E-04	1.43E-08	0.213
3	38080101	38723967	263	-1.13E-05	6.08E-09	0.885
3	38724849	40649760	701	-2.67E-06	1.49E-08	0.983
3	40660283	42184173	434	-1.66E-05	9.15E-09	0.862
3	42184361	45162316	1057	-6.16E-05	2.32E-08	0.686
3	45165430	46657162	653	1.20E-04	7.05E-08	0.651
3	46657428	48525955	404	4.97E-05	8.03E-09	0.579
3	48531227	52214640	853	6.59E-05	1.68E-08	0.612
3	52215002	54078568	705	5.74E-05	1.45E-08	0.633
3	54080241	55731145	920	-5.21E-05	2.51E-08	0.742
3	55733390	58063819	872	-1.87E-04	1.84E-08	0.168
3	58067795	58700675	307	1.36E-05	6.66E-09	0.867
3	58700802	60347135	935	-1.74E-05	2.61E-08	0.914
3	60347701	61277928	613	1.65E-05	1.56E-08	0.895
3	61279726	62881664	854	-5.18E-05	2.48E-08	0.742
3	62882619	63504979	310	-9.94E-05	7.59E-09	0.254
3	63505191	64473972	483	-5.00E-05	1.30E-08	0.660
3	64475164	65337581	545	1.04E-04	1.50E-08	0.397
3	65340365	66169323	368	-4.89E-05	1.10E-08	0.640
3	66170253	67294968	419	-6.42E-05	1.05E-08	0.531
3	67299401	69197507	757	-8.68E-05	1.76E-08	0.513
3	69205190	70288126	413	4.20E-05	1.01E-08	0.676
3	70289919	71418327	409	-1.60E-04	1.83E-08	0.236
3	71421152	73238935	812	-8.59E-05	2.40E-08	0.579
3	73239448	75376291	876	-5.08E-05	2.23E-08	0.734
3	75377075	76854466	340	8.13E-05	8.02E-09	0.364
3	76855676	77507524	287	-1.68E-05	7.20E-09	0.843
3	77508835	79022673	488	5.90E-05	1.15E-08	0.583
3	79024541	81187867	525	-3.39E-05	1.04E-08	0.740
3	81190039	82446513	356	-6.24E-06	6.70E-09	0.939
3	82446746	83946780	383	-1.21E-05	7.97E-09	0.893
3	83947451	85090935	310	-2.30E-07	6.50E-09	0.998
3	85093629	86734415	434	1.33E-04	8.26E-09	0.143

3	86736368	87407921	210	2.65E-05	1.93E-08	0.848
3	87408634	88725583	376	-5.63E-05	7.65E-09	0.520
3	88725754	94202010	444	1.02E-04	9.27E-09	0.289
3	94202771	96056259	472	-1.01E-04	8.87E-09	0.284
3	96679063	98299365	463	-3.80E-05	9.60E-09	0.698
3	98300731	100591006	762	6.81E-05	1.52E-08	0.580
3	100591848	101808006	473	1.15E-04	9.82E-09	0.246
3	101808379	103025517	322	1.09E-05	6.95E-09	0.896
3	103025715	103698105	210	6.27E-05	4.53E-09	0.352
3	103700909	104579408	324	7.47E-05	7.29E-09	0.382
3	104580063	104878270	154	5.04E-05	3.44E-09	0.390
3	104879008	106330349	517	1.67E-05	1.25E-08	0.881
3	106332831	107729547	479	4.59E-05	1.61E-08	0.718
3	107730020	108635884	340	-6.45E-05	7.69E-09	0.462
3	108636466	109961316	402	8.65E-05	8.28E-09	0.342
3	109962369	111416310	404	2.57E-05	8.41E-09	0.779
3	111416451	112182995	420	5.11E-08	9.37E-09	1.000
3	112183360	113323793	431	-1.16E-05	1.55E-08	0.926
3	113324119	115280312	582	1.23E-04	1.44E-08	0.305
3	115282058	116450487	508	1.95E-05	1.23E-08	0.860
3	116452274	117998840	676	1.82E-04	1.81E-08	0.176
3	118000136	120520317	1012	-4.62E-05	2.25E-08	0.758
3	120521733	121335461	214	-7.31E-06	4.20E-09	0.910
3	121336367	122692039	596	1.37E-04	1.28E-08	0.225
3	122693533	123196819	219	-1.21E-05	5.10E-09	0.865
3	123200440	124677278	597	-3.71E-05	1.37E-08	0.751
3	124678230	125875831	441	-1.67E-04	1.06E-08	0.106
3	125876862	126213162	202	3.59E-05	5.33E-09	0.623
3	126213467	127471230	536	1.13E-05	1.26E-08	0.920
3	127471641	130243259	807	-2.74E-04	5.20E-08	0.230
3	130244120	131373405	333	-6.04E-05	6.52E-09	0.454
3	131373740	132115911	443	2.72E-05	9.96E-09	0.785
3	132117285	133128449	407	-7.53E-05	8.73E-09	0.420
3	133129272	134383242	550	1.61E-05	1.42E-08	0.893
3	137370076	138662229	384	1.16E-04	7.96E-09	0.194
3	134385789	137369181	1105	9.25E-05	2.38E-08	0.549
3	138665796	140275652	774	6.21E-05	1.87E-08	0.650

3	140276484	142863564	911	-5.76E-05	2.01E-08	0.684
3	142864473	143494546	387	-2.42E-05	1.20E-08	0.825
3	143495679	145663879	729	9.21E-05	1.57E-08	0.462
3	145664135	146722271	401	1.57E-04	9.20E-09	0.102
3	146722383	148316359	475	-5.97E-05	1.16E-08	0.580
3	148316766	149988630	749	1.74E-04	1.91E-08	0.208
3	149994553	151178708	481	-6.72E-05	1.19E-08	0.538
3	151178836	151904190	289	-5.30E-05	6.82E-09	0.521
3	151905386	153054901	368	3.00E-05	9.20E-09	0.755
3	153056839	154181836	468	6.04E-05	1.02E-08	0.550
3	154183562	156005200	508	-6.37E-06	1.21E-08	0.954
3	156005905	157311596	528	6.56E-05	1.20E-08	0.549
3	157312028	159272972	716	1.87E-05	1.50E-08	0.879
3	159274785	161523859	701	7.26E-05	1.46E-08	0.548
3	161524489	162065286	248	5.46E-06	6.81E-09	0.947
3	162067622	163250087	374	-2.10E-06	7.42E-09	0.981
3	163251056	164816939	493	-4.84E-05	9.25E-09	0.615
3	164820183	167116849	615	5.06E-05	1.34E-08	0.662
3	167117143	167912878	293	7.71E-06	6.24E-09	0.922
3	167913564	170158128	713	-6.81E-05	3.60E-08	0.720
3	170159134	171311936	486	-5.06E-05	1.38E-08	0.667
3	171312880	172965694	657	1.80E-05	1.79E-08	0.893
3	172966405	174274308	513	-1.93E-04	1.22E-08	0.081
3	174275336	175049294	365	-2.76E-05	9.52E-09	0.778
3	175050262	176268375	459	-1.80E-05	1.10E-08	0.864
3	176269091	177530606	501	-5.79E-05	1.31E-08	0.614
3	177531847	179359242	627	1.31E-04	1.48E-08	0.282
3	179366819	181426554	525	-7.34E-05	1.20E-08	0.503
3	181431571	182975975	457	5.54E-05	1.23E-08	0.618
3	182976382	183762614	363	-1.02E-04	7.63E-09	0.241
3	183762644	185262137	590	-2.52E-05	1.64E-08	0.844
3	185263467	186780913	684	-1.13E-04	2.05E-08	0.430
3	186782999	187220633	227	1.73E-04	6.07E-09	0.027
3	187220777	188201380	508	6.37E-05	1.48E-08	0.601
3	188202550	189531496	581	-1.76E-05	1.74E-08	0.893
3	189531911	190303805	414	8.21E-06	1.11E-08	0.938
3	190304172	190992002	285	5.26E-05	6.84E-09	0.524

3	190993386	191840443	361	-1.48E-05	8.49E-09	0.872
3	191840983	192753870	379	-1.26E-04	9.29E-09	0.193
3	192754177	193885973	511	7.67E-05	1.39E-08	0.515
3	193886289	194464791	285	1.08E-04	8.57E-09	0.243
3	194465233	194756339	146	4.48E-05	5.22E-09	0.536
3	194756597	195854787	319	3.17E-05	8.06E-09	0.724
3	195855401	196414159	209	-8.42E-05	6.14E-09	0.282
3	196415355	197962382	525	3.65E-05	1.20E-08	0.739
4	10642	685993	148	-1.52E-05	2.09E-09	0.739
4	686563	1422863	300	-1.17E-04	5.23E-09	0.104
4	3613164	4263579	146	9.09E-05	5.07E-09	0.202
4	1478711	3612028	636	-6.54E-05	1.03E-08	0.518
4	4264705	5044744	439	2.13E-05	9.19E-09	0.824
4	5047695	5971152	585	-2.64E-05	1.39E-08	0.822
4	5971634	7135912	601	-9.07E-05	1.62E-08	0.476
4	7136158	8054472	690	-3.06E-04	2.35E-08	0.045
4	8055323	9408136	367	3.96E-05	1.25E-08	0.723
4	9408158	10696229	605	8.85E-06	7.88E-09	0.921
4	10696913	12198579	733	8.07E-05	1.25E-08	0.470
4	12201002	13002341	347	-1.28E-05	4.98E-09	0.856
4	13002905	14394452	629	3.67E-06	1.24E-08	0.974
4	14394902	15849986	605	7.34E-07	1.02E-08	0.994
4	15850685	16520118	382	5.27E-05	7.35E-09	0.538
4	16521003	18752293	860	4.62E-05	1.41E-08	0.697
4	18752722	20084049	441	6.61E-05	7.46E-09	0.444
4	20084563	21292213	549	1.90E-04	9.71E-09	0.054
4	21292651	22393873	439	9.89E-06	7.13E-09	0.907
4	22395570	23563570	369	4.07E-05	6.66E-09	0.618
4	23563931	24992827	683	-2.64E-05	1.34E-08	0.820
4	24993167	26468997	677	1.70E-04	1.62E-08	0.182
4	26473220	27903383	509	-2.18E-05	9.64E-09	0.824
4	27904556	28871862	388	8.61E-05	6.07E-09	0.269
4	28872833	30453156	508	-9.55E-06	8.63E-09	0.918
4	30453619	31739254	480	2.65E-05	8.05E-09	0.768
4	31740009	32592949	294	3.35E-06	4.71E-09	0.961
4	32593453	33640100	204	-1.01E-04	2.89E-09	0.060
4	33640864	35150284	378	-2.22E-06	4.01E-09	0.972

4	35151237	36301997	434	1.55E-05	6.92E-09	0.853
4	36304377	37534798	577	-6.18E-06	1.09E-08	0.953
4	37536119	37886455	212	-8.25E-05	5.01E-09	0.244
4	37887735	38974458	602	5.86E-05	1.33E-08	0.611
4	38976533	40199852	438	-1.47E-04	8.10E-09	0.103
4	40200304	40869208	304	2.10E-05	8.37E-09	0.818
4	40870402	42662283	725	-9.71E-05	1.29E-08	0.392
4	42662718	43962569	451	-1.55E-05	6.80E-09	0.851
4	43963518	45187835	413	1.58E-05	5.84E-09	0.836
4	45188855	47543891	621	-9.87E-07	8.67E-09	0.992
4	47544012	48226267	277	2.02E-05	3.74E-09	0.741
4	48227642	53412129	408	7.42E-05	3.86E-09	0.232
4	53413674	55202613	629	-3.51E-05	9.22E-09	0.715
4	55202697	57418400	883	8.25E-05	1.56E-08	0.510
4	57420107	58696072	630	1.48E-05	1.24E-08	0.894
4	58697912	59358251	234	1.16E-05	3.94E-09	0.854
4	59358729	60145179	250	-1.97E-05	4.05E-09	0.757
4	60145181	61371992	375	-5.35E-05	5.78E-09	0.482
4	61372415	61872517	167	-1.31E-05	2.71E-09	0.801
4	61872656	64117718	644	-2.19E-05	1.11E-08	0.835
4	64118963	65300308	239	-5.00E-06	3.02E-09	0.927
4	65562213	66598615	369	2.87E-05	5.83E-09	0.707
4	66599449	68058793	478	-9.35E-05	7.58E-09	0.282
4	68059850	68455531	160	-6.79E-06	2.40E-09	0.890
4	68456074	69954151	269	-2.32E-05	4.14E-09	0.718
4	69954297	70517143	265	1.97E-05	3.14E-09	0.725
4	70517792	71516586	418	6.95E-05	5.60E-09	0.353
4	72416258	75196731	712	1.27E-04	1.22E-08	0.251
4	75555658	76283179	293	1.72E-05	5.56E-09	0.817
4	76284248	77128724	363	-3.16E-06	4.52E-09	0.963
4	77129568	78333381	606	-7.11E-05	1.01E-08	0.480
4	78333798	79469226	505	-3.11E-05	7.42E-09	0.718
4	79470550	80994124	436	-4.89E-06	7.30E-09	0.954
4	80995881	81978151	261	-1.26E-05	3.88E-09	0.840
4	81979993	83012967	403	-1.79E-05	5.88E-09	0.815
4	83017789	83994716	385	-3.81E-05	5.65E-09	0.612
4	83998014	85080933	430	-1.57E-05	7.49E-09	0.856

4	85081980	87980070	900	-1.03E-05	1.22E-08	0.926
4	87980748	89236855	568	2.67E-05	1.05E-08	0.794
4	89238369	90126190	323	-8.89E-05	5.72E-09	0.240
4	90126331	91453993	446	-1.08E-04	7.52E-09	0.214
4	91456722	94229509	751	1.73E-05	1.25E-08	0.877
4	94230511	95596188	496	-1.92E-04	4.01E-08	0.337
4	95596744	96181408	236	2.79E-05	4.06E-09	0.661
4	96182669	96464931	212	2.66E-05	4.56E-09	0.694
4	96466095	97911191	514	7.00E-05	7.83E-09	0.429
4	97911459	99422081	371	-6.53E-06	3.46E-09	0.912
4	99423280	100322786	399	1.65E-06	5.74E-09	0.983
4	100323029	101897750	598	2.49E-05	8.59E-09	0.788
4	101898276	103387944	552	-7.46E-05	8.14E-09	0.408
4	103388441	104802530	434	1.25E-05	4.88E-09	0.858
4	104803329	106479425	508	7.21E-04	7.92E-08	0.010
4	106482185	108468632	626	-1.30E-04	1.83E-08	0.337
4	108469756	109979131	574	2.02E-05	8.51E-09	0.827
4	109980374	112204254	780	1.70E-04	1.39E-08	0.151
4	112204259	114902479	1023	3.68E-05	1.62E-08	0.773
4	114904730	117275534	678	-8.51E-05	8.80E-09	0.364
4	117275542	118219755	308	-8.65E-05	5.02E-09	0.222
4	118220292	119945314	632	-2.64E-05	9.72E-09	0.789
4	119946706	121391843	518	3.06E-05	6.96E-09	0.713
4	121393020	121973864	244	1.99E-04	4.36E-09	0.003
4	121976226	123561869	550	-3.18E-05	8.68E-09	0.733
4	123563078	125863762	773	5.01E-05	1.27E-08	0.657
4	125863974	127236805	494	-1.45E-04	1.29E-08	0.201
4	127237363	129514427	625	-1.32E-05	7.99E-09	0.882
4	129514448	130364383	286	8.46E-05	4.79E-09	0.221
4	130364741	130949530	194	-4.31E-05	4.34E-09	0.513
4	130950008	131948861	379	4.53E-05	6.61E-09	0.578
4	131951466	133741365	442	-5.32E-05	6.35E-09	0.505
4	133741753	135744132	532	1.46E-05	7.29E-09	0.865
4	135749591	137247266	391	1.47E-05	7.08E-09	0.861
4	137247703	137987679	298	2.19E-05	6.10E-09	0.779
4	137988838	139114529	402	-1.45E-06	6.98E-09	0.986
4	139115093	139552965	239	7.32E-05	4.73E-09	0.287

4	139553359	140546206	342	-7.49E-05	5.62E-09	0.318
4	140548713	141751418	489	5.32E-05	1.43E-08	0.656
4	141752960	142807957	391	-7.42E-05	7.43E-09	0.389
4	142808503	145021868	626	-1.70E-05	7.30E-09	0.842
4	145024452	148047972	851	1.70E-04	1.31E-08	0.137
4	148053679	150632409	779	1.32E-04	1.18E-08	0.224
4	150634191	153226998	835	-3.78E-06	1.07E-08	0.971
4	153233476	154947363	614	4.17E-05	1.01E-08	0.678
4	154949091	156604003	633	-2.88E-05	1.20E-08	0.792
4	156604173	158112985	581	1.65E-05	1.07E-08	0.873
4	158114105	160356254	643	-2.99E-05	9.36E-09	0.758
4	160357877	160822830	147	-2.76E-05	2.02E-09	0.539
4	161056173	161796696	321	1.50E-05	5.59E-09	0.841
4	162151565	162629423	187	-5.07E-05	4.64E-09	0.456
4	162629512	163181531	215	2.32E-05	3.57E-09	0.698
4	163183341	163726178	186	-1.52E-05	3.66E-09	0.801
4	163728264	164910818	398	4.18E-05	6.81E-09	0.613
4	164913270	165817608	367	7.56E-06	6.68E-09	0.926
4	165820093	166609852	327	-3.69E-05	6.53E-09	0.648
4	166611193	167643023	297	3.36E-06	5.84E-09	0.965
4	167643601	169568082	733	7.26E-05	1.29E-08	0.522
4	169569516	170676328	420	-3.56E-05	6.61E-09	0.662
4	170678993	172735897	572	-1.85E-05	8.71E-09	0.843
4	172737462	173760563	310	2.27E-06	4.63E-09	0.973
4	173761916	174832248	301	-9.18E-06	7.22E-09	0.914
4	174833988	176193705	541	9.87E-06	1.23E-08	0.929
4	176199091	177290105	362	7.01E-06	7.45E-09	0.935
4	177290945	177972278	282	7.50E-06	4.95E-09	0.915
4	177974062	178758792	336	-5.12E-05	8.20E-09	0.572
4	178758930	180119509	494	2.46E-05	9.50E-09	0.801
4	180120693	181732608	833	2.63E-06	1.94E-08	0.985
4	181732746	182420390	387	2.30E-05	1.09E-08	0.826
4	184283411	185477454	593	-7.91E-05	1.67E-08	0.541
4	182420863	184283167	1001	-7.94E-05	2.60E-08	0.622
4	185477964	185932497	229	-4.12E-05	4.08E-09	0.519
4	185934003	187113041	594	-1.31E-04	1.42E-08	0.273
4	187114073	188687052	716	-7.44E-05	1.59E-08	0.556

4	188687155	189524815	363	-9.21E-05	1.10E-08	0.380
4	189525752	190088167	269	-5.75E-05	6.10E-09	0.462
4	190088768	191043878	198	-2.98E-05	4.50E-09	0.657
5	10056	1267356	414	7.79E-05	1.01E-08	0.438
5	1270983	1762678	241	1.84E-04	1.63E-08	0.149
5	1763566	2369337	388	5.53E-06	2.25E-08	0.971
5	2369710	2979000	434	8.66E-05	9.68E-09	0.379
5	2979710	3600334	481	9.27E-05	1.01E-08	0.357
5	3601924	4222111	305	5.76E-05	6.07E-09	0.460
5	4223577	5137474	431	5.06E-05	8.46E-09	0.582
5	5137788	5718773	374	-3.49E-05	7.84E-09	0.694
5	5719162	6858229	682	-5.39E-05	1.32E-08	0.639
5	6859297	7852978	494	8.04E-05	9.18E-09	0.402
5	7853304	9084076	627	2.32E-05	1.03E-08	0.819
5	9084701	10533767	856	5.01E-05	1.64E-08	0.696
5	10666804	11170506	324	-1.90E-05	5.47E-09	0.797
5	11170703	13603734	759	1.11E-04	1.10E-08	0.290
5	13603883	14987807	739	-7.50E-05	1.17E-08	0.488
5	14988284	16034608	298	-2.95E-06	3.26E-09	0.959
5	16035369	17161451	493	-4.38E-05	9.95E-09	0.661
5	17162704	18048683	258	2.13E-04	5.71E-09	0.005
5	18050107	18791978	251	-2.33E-05	4.72E-09	0.735
5	18792568	19486153	258	2.82E-05	4.08E-09	0.659
5	19487359	20414139	285	1.17E-05	3.35E-09	0.840
5	20415799	21573200	238	9.20E-06	3.82E-09	0.882
5	21574347	23150924	572	2.13E-05	6.88E-09	0.797
5	23153562	24794957	518	1.47E-06	7.76E-09	0.987
5	24796795	26183141	453	9.24E-05	8.06E-09	0.303
5	26185302	26849079	203	7.26E-05	5.13E-09	0.311
5	26849808	28303480	390	4.84E-05	6.27E-09	0.541
5	28310093	30129786	582	1.62E-04	9.60E-09	0.098
5	30130713	31748529	717	4.12E-05	1.35E-08	0.722
5	31748611	33944217	987	1.35E-05	2.02E-08	0.924
5	33945758	35798682	685	4.87E-05	1.18E-08	0.654
5	35799275	37888797	709	1.59E-05	1.12E-08	0.880
5	37889231	39438373	674	6.43E-05	1.19E-08	0.556
5	39438575	40954879	626	6.51E-05	7.82E-09	0.462

5	40955561	42620153	556	4.26E-05	6.37E-09	0.594
5	42623245	43859513	306	2.95E-05	3.02E-09	0.592
5	43862567	45286358	281	3.97E-05	2.77E-09	0.450
5	45286977	50161698	288	-4.90E-07	2.28E-09	0.992
5	50162186	52133670	655	-9.71E-05	9.60E-09	0.322
5	52134410	52661197	301	7.40E-05	4.54E-09	0.272
5	52662483	53513531	324	5.23E-05	5.70E-09	0.489
5	53515888	55412347	789	-6.94E-05	1.07E-08	0.502
5	55413961	55932471	298	-7.36E-05	8.18E-09	0.416
5	55933701	57337744	583	-2.78E-05	8.47E-09	0.762
5	57338357	58834736	805	1.19E-04	1.31E-08	0.297
5	58834852	60880207	583	8.08E-06	6.99E-09	0.923
5	60883078	62531300	657	4.65E-05	8.66E-09	0.618
5	62531952	63837838	373	8.94E-06	4.98E-09	0.899
5	63840208	65083148	458	8.02E-05	5.76E-09	0.290
5	65083209	65909616	333	-1.11E-04	6.12E-09	0.156
5	65910316	67096208	517	1.07E-04	8.52E-09	0.247
5	67096780	70682458	687	-5.89E-05	1.18E-08	0.587
5	70683839	72420748	628	-1.42E-04	7.40E-09	0.098
5	72420784	73507955	557	-7.55E-05	1.00E-08	0.451
5	73508509	75240469	728	-8.99E-05	1.08E-08	0.387
5	75242395	75740199	233	2.16E-05	4.04E-09	0.734
5	75743532	77203135	737	2.69E-04	1.36E-08	0.021
5	77203718	79068089	683	-2.47E-05	1.07E-08	0.811
5	79068300	80480761	702	5.04E-05	1.30E-08	0.659
5	80481996	81712715	433	-4.12E-05	6.79E-09	0.617
5	81714078	82658571	362	-1.17E-04	6.48E-09	0.144
5	82660901	84397685	585	6.52E-05	1.19E-08	0.551
5	84399219	85315500	310	-7.35E-06	3.87E-09	0.906
5	85316429	86490293	328	3.68E-06	5.36E-09	0.960
5	86492986	88065637	303	-1.04E-05	3.47E-09	0.859
5	88069593	90500398	713	-5.55E-05	1.10E-08	0.596
5	90504842	93602613	719	-9.13E-05	9.18E-09	0.341
5	93607443	95822590	794	-1.09E-04	1.25E-08	0.328
5	95822732	96867936	466	-2.44E-05	6.91E-09	0.769
5	96870861	97435808	143	4.06E-05	2.19E-09	0.385
5	97436504	99258325	556	-3.74E-05	7.51E-09	0.666

5	99259751	101101103	482	2.64E-06	5.72E-09	0.972
5	101101325	102681586	443	3.23E-05	4.56E-09	0.632
5	102686157	104542488	700	4.39E-05	1.22E-08	0.692
5	104543131	105885913	312	1.09E-06	4.60E-09	0.987
5	105887333	106800282	343	-1.31E-04	6.42E-09	0.101
5	108633347	109231597	246	-1.25E-05	2.94E-09	0.817
5	106800765	108633176	758	3.93E-05	1.36E-08	0.736
5	109232816	110819753	510	1.29E-04	7.43E-09	0.133
5	110821786	111962676	470	-1.10E-04	8.52E-09	0.234
5	111963025	112396750	155	7.78E-06	1.89E-09	0.858
5	112397011	113603768	561	2.17E-05	8.21E-09	0.811
5	113604579	114448326	332	1.12E-05	5.06E-09	0.875
5	114448597	115828291	681	-4.17E-05	1.08E-08	0.689
5	115829425	117003674	543	1.56E-04	9.59E-09	0.112
5	117004531	117712427	262	4.27E-05	4.48E-09	0.524
5	117714517	119668423	688	-6.27E-05	1.12E-08	0.553
5	119669457	120962383	495	2.88E-05	8.50E-09	0.755
5	120963248	121481183	148	3.59E-05	2.25E-09	0.449
5	121485609	122603725	451	-3.23E-05	6.80E-09	0.696
5	122604501	123236124	257	-3.03E-05	3.85E-09	0.625
5	123238611	123797013	229	3.48E-05	4.11E-09	0.587
5	123798436	124746813	465	4.52E-05	9.80E-09	0.648
5	124748624	126938920	873	-1.21E-04	1.43E-08	0.311
5	126940058	128727903	666	-4.36E-05	9.30E-09	0.651
5	128730275	130376511	412	6.94E-06	4.09E-09	0.914
5	130376936	131873073	450	2.30E-05	6.13E-09	0.769
5	131874022	132603137	223	-2.15E-05	3.35E-09	0.710
5	132609517	134321546	718	-1.56E-04	1.32E-08	0.174
5	134329463	135339464	501	-4.45E-05	8.06E-09	0.620
5	135340951	137948424	905	2.60E-05	1.11E-08	0.806
5	137950183	140644484	763	-3.61E-05	7.63E-09	0.679
5	140644957	141640050	474	-7.21E-05	9.10E-09	0.449
5	141640419	142230251	276	-8.57E-05	5.99E-09	0.268
5	142230949	144583138	961	1.29E-04	1.33E-08	0.263
5	144584265	145803004	431	-7.43E-06	4.97E-09	0.916
5	145803849	147651230	761	-4.80E-06	1.22E-08	0.965
5	150395351	150917796	368	4.58E-05	6.11E-09	0.558

5	150918158	152360494	593	1.90E-04	8.76E-09	0.042
5	147665223	150393107	1251	2.99E-05	1.90E-08	0.828
5	152361546	152864614	177	-4.53E-06	2.42E-09	0.927
5	152867348	153494463	316	2.06E-05	3.63E-09	0.733
5	153495193	154448863	413	-4.17E-05	7.15E-09	0.622
5	154449856	155290211	358	4.33E-05	5.36E-09	0.554
5	155290613	156628287	483	-3.04E-05	7.63E-09	0.728
5	156629754	157197141	306	-4.13E-05	4.52E-09	0.539
5	157199547	158491655	551	4.13E-07	7.99E-09	0.996
5	158492279	159135376	279	6.89E-05	5.16E-09	0.337
5	159136348	160908680	774	4.34E-05	1.36E-08	0.709
5	160910101	161688620	224	-2.14E-05	3.33E-09	0.711
5	161688913	162741298	358	7.35E-05	5.65E-09	0.328
5	162742614	163769136	431	1.20E-04	7.43E-09	0.163
5	163769738	164944949	399	5.39E-05	6.30E-09	0.497
5	164947504	165638940	255	1.06E-05	6.47E-09	0.895
5	165642395	166432731	347	-9.29E-05	7.51E-09	0.284
5	166433367	167760730	549	-2.89E-05	1.07E-08	0.780
5	167761922	169124478	717	1.84E-04	1.43E-08	0.123
5	169125063	170889081	951	-5.53E-05	1.65E-08	0.667
5	170891834	171772889	429	5.01E-06	8.53E-09	0.957
5	171775715	172376903	328	6.19E-06	7.48E-09	0.943
5	172377031	173611384	633	2.71E-05	1.27E-08	0.810
5	173613085	174468408	480	1.68E-04	1.71E-08	0.200
5	174468624	176128648	690	4.14E-05	1.47E-08	0.733
5	176129392	177991305	614	-1.20E-04	1.44E-08	0.316
5	177992368	178410668	199	4.52E-05	4.26E-09	0.489
5	178410839	179245749	390	-1.40E-06	8.01E-09	0.988
5	179245856	180007693	315	3.20E-05	6.83E-09	0.699
5	180008456	180790950	242	-2.53E-05	6.01E-09	0.744
6	63979	824529	327	4.67E-05	9.15E-09	0.626
6	826312	1582647	522	-7.00E-05	1.48E-08	0.565
6	1582926	2890836	717	1.07E-04	1.62E-08	0.402
6	2891016	4315210	770	-1.72E-04	1.84E-08	0.205
6	4317398	5056146	425	6.60E-05	9.03E-09	0.488
6	5057399	6246639	732	1.33E-04	1.69E-08	0.307
6	6246984	7328580	685	1.67E-04	1.95E-08	0.233

6	7329417	8791321	740	-4.12E-06	1.39E-08	0.972
6	8791421	9760323	373	-8.40E-06	9.51E-09	0.931
6	9764413	11234164	726	-1.57E-06	1.93E-08	0.991
6	11235682	12183952	542	8.98E-05	1.10E-08	0.392
6	12186102	13388480	617	-4.69E-05	1.20E-08	0.668
6	13389521	14618107	554	9.84E-05	1.14E-08	0.358
6	14619148	15998368	595	-5.75E-06	1.24E-08	0.959
6	15998406	17067160	545	1.84E-04	1.49E-08	0.132
6	17069966	19203108	878	6.59E-05	1.68E-08	0.611
6	19203782	20228741	524	1.53E-05	1.31E-08	0.893
6	20231516	22049758	823	-3.58E-06	2.13E-08	0.980
6	22052425	23495209	747	-1.01E-04	1.91E-08	0.466
6	23495260	23935020	233	6.56E-05	4.69E-09	0.339
6	23935352	24799440	436	-3.03E-05	1.11E-08	0.774
6	24803038	25483400	411	-1.72E-05	8.78E-09	0.855
6	25484295	26774948	727	-1.53E-04	1.47E-08	0.207
6	26778827	28669698	609	-7.61E-05	1.05E-08	0.457
6	32900018	33863002	400	-1.15E-04	1.36E-08	0.323
6	33863662	35451187	511	-4.87E-05	8.62E-09	0.600
6	35452346	36608267	395	6.58E-06	7.14E-09	0.938
6	38687185	39021712	205	-2.41E-05	4.14E-09	0.708
6	36611164	38686222	996	1.59E-04	2.12E-08	0.275
6	39022698	40344526	674	1.16E-04	1.37E-08	0.321
6	40345542	42105449	884	-1.77E-04	1.92E-08	0.201
6	42105555	43957473	632	2.43E-05	1.50E-08	0.843
6	43959116	45787468	700	3.33E-05	1.50E-08	0.785
6	45788111	46556325	327	-5.87E-05	5.84E-09	0.442
6	46560003	47885216	680	-6.58E-05	1.17E-08	0.543
6	47887063	49494241	468	-5.70E-05	7.06E-09	0.497
6	49494933	50406476	309	-8.50E-05	5.80E-09	0.264
6	50406575	51703012	476	-1.04E-05	8.54E-09	0.910
6	51704668	53402679	834	-1.03E-04	1.70E-08	0.431
6	53405127	54291407	455	-4.73E-05	7.85E-09	0.593
6	54293261	56103747	744	3.76E-05	1.27E-08	0.739
6	56104522	57082026	352	3.66E-05	5.59E-09	0.624
6	57085542	58084365	149	5.21E-06	2.67E-09	0.920
6	58139505	62691733	301	1.25E-05	4.55E-09	0.853

6	62692409	63712779	264	5.01E-06	3.61E-09	0.934
6	63713620	65760585	508	-3.13E-05	7.06E-09	0.710
6	65762553	67028107	491	-1.50E-05	8.41E-09	0.870
6	67029501	67556729	233	2.31E-05	4.59E-09	0.733
6	67557245	68546601	377	7.78E-05	7.00E-09	0.352
6	68546995	69295414	301	-1.55E-05	6.99E-09	0.853
6	69295526	70359936	399	-6.68E-06	6.72E-09	0.935
6	70360869	72448598	881	-1.50E-05	1.60E-08	0.906
6	72449421	73170712	296	2.89E-05	5.75E-09	0.703
6	73171594	75064563	682	2.56E-05	1.27E-08	0.821
6	75065279	75843360	172	-1.25E-05	3.42E-09	0.831
6	75843845	77257094	415	1.65E-05	6.18E-09	0.833
6	77257275	78957547	677	-2.36E-05	1.12E-08	0.823
6	78958134	79861169	296	-1.51E-05	4.72E-09	0.826
6	79862483	80556359	340	-1.36E-05	7.15E-09	0.872
6	80557170	82387384	657	8.53E-05	1.01E-08	0.397
6	82387920	83904869	519	7.32E-05	9.54E-09	0.454
6	83908232	86040334	810	2.82E-05	1.45E-08	0.815
6	86040497	87138115	340	-1.12E-05	5.40E-09	0.879
6	87140364	88532072	484	-1.79E-04	8.92E-09	0.058
6	88533324	89668547	467	1.16E-04	1.09E-08	0.268
6	89673861	91400644	815	9.10E-05	1.70E-08	0.485
6	91401234	93426186	759	4.63E-05	1.57E-08	0.711
6	93429797	94919277	573	8.71E-05	1.12E-08	0.411
6	94920611	97093037	660	3.95E-05	1.22E-08	0.720
6	97093295	98893182	513	-6.46E-05	8.94E-09	0.495
6	98893830	100286683	584	-2.87E-05	1.13E-08	0.787
6	100287245	101862923	464	-4.21E-05	7.98E-09	0.637
6	101863563	102887537	347	8.63E-06	7.24E-09	0.919
6	102888281	104211089	445	-8.92E-05	7.24E-09	0.295
6	104211475	105752435	548	-5.37E-05	9.86E-09	0.589
6	105754306	106921804	533	-1.09E-04	1.36E-08	0.351
6	106922281	108322638	653	-1.76E-05	1.50E-08	0.886
6	108325624	110654066	761	-1.05E-04	1.81E-08	0.434
6	110654605	111390344	273	6.26E-06	5.75E-09	0.934
6	111391537	114218733	1067	8.73E-05	1.95E-08	0.532
6	114220361	116130955	594	-9.34E-05	1.08E-08	0.368

6	116130983	117566682	495	-5.21E-05	1.00E-08	0.603
6	117567468	119071713	561	-1.26E-05	1.02E-08	0.900
6	119075856	120628075	540	-5.13E-05	1.03E-08	0.614
6	120628214	121673627	338	-1.13E-05	5.44E-09	0.878
6	121674337	123104703	522	-3.79E-06	8.96E-09	0.968
6	123105676	123889346	320	-5.91E-06	7.02E-09	0.944
6	123890282	125333797	656	4.57E-06	1.48E-08	0.970
6	125336799	128759251	899	-8.73E-05	1.59E-08	0.489
6	128759755	130538497	786	5.30E-05	1.68E-08	0.682
6	130540383	131972731	564	2.26E-04	1.09E-08	0.030
6	131973932	133867802	872	2.25E-05	1.89E-08	0.870
6	133869809	136186007	801	-4.71E-05	2.07E-08	0.744
6	136186368	137811295	507	3.30E-05	9.78E-09	0.738
6	137813095	139532424	747	6.99E-05	1.75E-08	0.597
6	139533572	142036032	706	-1.13E-04	1.31E-08	0.324
6	142036912	143719278	602	-7.35E-05	1.14E-08	0.491
6	146665424	148179283	590	4.95E-06	1.13E-08	0.963
6	143720467	146663272	1046	1.64E-05	1.89E-08	0.905
6	148180191	148917941	479	-6.91E-05	1.36E-08	0.553
6	148919158	150298842	637	5.82E-05	1.10E-08	0.579
6	150299299	151097980	538	-1.58E-04	1.43E-08	0.186
6	151100484	151929942	489	7.59E-05	1.29E-08	0.504
6	151930249	153550147	880	-2.93E-04	3.43E-08	0.114
6	153551177	155228511	744	-5.57E-05	1.72E-08	0.672
6	155229566	156756457	767	1.11E-04	1.82E-08	0.409
6	156761338	157398853	255	-9.81E-05	5.21E-09	0.174
6	157402041	158192951	273	1.61E-05	6.85E-09	0.846
6	158193244	159318774	547	-9.50E-05	9.51E-09	0.330
6	159319812	159792458	251	-5.68E-06	3.85E-09	0.927
6	159793334	161123451	690	-2.85E-04	6.82E-08	0.275
6	161123771	162298351	679	-1.03E-04	2.04E-08	0.472
6	162300151	163277414	507	4.30E-05	1.02E-08	0.670
6	163277858	164003180	378	1.24E-04	8.63E-09	0.180
6	164006012	165242243	674	-3.41E-06	1.44E-08	0.977
6	165246062	166191910	571	9.40E-05	1.35E-08	0.418
6	166193932	167177733	678	2.21E-05	1.64E-08	0.863
6	167178790	168548525	625	9.18E-05	1.26E-08	0.413

6	168549335	169231503	389	-1.72E-05	9.16E-09	0.857
6	169231937	171049558	872	-4.79E-05	1.70E-08	0.713
7	20608	1490775	409	-2.06E-05	8.89E-09	0.827
7	1493090	2479029	317	-1.67E-04	1.27E-08	0.140
7	2480729	2930941	207	4.08E-05	4.36E-09	0.537
7	2931676	3736942	499	5.10E-06	1.06E-08	0.961
7	3738087	4783224	511	2.04E-05	1.10E-08	0.846
7	4783723	5412932	213	4.07E-06	6.11E-09	0.958
7	5414602	6912527	397	1.77E-04	8.82E-09	0.059
7	7168493	7679885	293	-1.02E-04	6.08E-09	0.191
7	7680697	8475147	543	4.78E-05	1.39E-08	0.686
7	8476787	9223721	524	-5.19E-05	1.20E-08	0.635
7	9223762	9893744	340	2.63E-05	8.18E-09	0.771
7	9894421	11298815	623	-1.19E-05	1.20E-08	0.914
7	11299552	12687871	878	-1.78E-05	1.88E-08	0.897
7	12689322	13674908	507	-3.13E-05	1.06E-08	0.761
7	13675094	14941781	683	5.26E-05	1.52E-08	0.670
7	14942134	16480989	759	-1.85E-05	1.48E-08	0.879
7	16481863	17277084	484	-6.23E-05	1.27E-08	0.580
7	17279294	18861958	710	-1.03E-04	1.47E-08	0.393
7	18863387	19975992	510	-7.56E-05	9.47E-09	0.437
7	19978609	21712233	877	-2.18E-04	3.03E-08	0.210
7	21712615	22811384	707	2.77E-05	1.67E-08	0.830
7	22811921	24389035	702	1.64E-04	1.84E-08	0.227
7	24389147	24980408	322	-7.34E-07	7.06E-09	0.993
7	24982494	26300992	674	-2.45E-04	2.32E-08	0.107
7	26302668	28038375	768	5.28E-04	1.21E-07	0.129
7	28039590	28710596	382	-7.21E-05	1.46E-08	0.551
7	28710857	30171413	826	3.74E-05	1.75E-08	0.777
7	30172195	31608495	659	2.87E-05	1.39E-08	0.808
7	31609317	33150311	749	-2.86E-05	1.40E-08	0.808
7	33151181	34939884	766	9.63E-05	1.45E-08	0.424
7	34940039	35614036	299	-3.80E-05	5.45E-09	0.607
7	35615611	37353302	858	-1.12E-04	2.01E-08	0.430
7	37354091	38159855	465	-3.45E-05	9.41E-09	0.722
7	38160558	39534540	677	-1.14E-04	1.40E-08	0.335
7	39535570	40925323	371	2.35E-07	6.58E-09	0.998

7	40926906	42494824	653	6.17E-05	1.36E-08	0.596
7	42495555	44971837	905	1.42E-05	1.45E-08	0.906
7	44972152	46665658	694	7.42E-05	1.27E-08	0.511
7	46667873	47279128	336	-1.04E-05	6.36E-09	0.896
7	47281095	49130572	862	2.32E-05	1.77E-08	0.861
7	49131824	49821750	300	3.90E-05	5.28E-09	0.591
7	49822437	51674880	774	-4.17E-06	1.40E-08	0.972
7	51675218	52274523	293	-3.35E-05	6.60E-09	0.680
7	52275656	53175065	478	-7.19E-06	9.15E-09	0.940
7	53348777	54707110	519	8.62E-06	8.71E-09	0.926
7	54707337	55641707	450	-6.44E-05	9.06E-09	0.499
7	55641798	57382254	384	5.19E-05	6.69E-09	0.525
7	57382271	62556935	172	1.08E-05	2.34E-09	0.823
7	62557202	64193303	280	-1.09E-05	4.35E-09	0.869
7	64699988	65570171	150	7.78E-06	1.87E-09	0.857
7	65570310	66453460	218	2.65E-05	1.91E-09	0.545
7	66454071	67369810	338	-7.62E-06	6.65E-09	0.926
7	67370861	68125403	315	4.81E-05	7.44E-09	0.577
7	68126378	68561421	188	-4.42E-05	3.65E-09	0.464
7	68562932	69806895	289	1.56E-05	4.20E-09	0.810
7	69808153	71204524	593	-6.71E-05	1.47E-08	0.580
7	71205507	71873571	248	-4.29E-05	4.18E-09	0.507
7	71874997	73996533	396	1.79E-06	6.74E-09	0.983
7	73997700	76550299	360	-3.26E-04	9.31E-09	0.001
7	76551983	77101532	179	-3.97E-05	3.62E-09	0.509
7	77102731	77953248	410	2.25E-05	7.24E-09	0.792
7	77954163	78839317	553	-1.43E-04	1.19E-08	0.190
7	78843289	80546745	613	-2.53E-05	1.06E-08	0.807
7	80547047	82758529	882	4.18E-05	1.91E-08	0.763
7	82758644	83959105	533	8.55E-05	9.94E-09	0.391
7	83959751	85570789	448	-1.10E-05	6.58E-09	0.892
7	85571372	86341446	218	-3.02E-05	3.28E-09	0.598
7	88732470	89270556	217	-7.44E-05	3.90E-09	0.234
7	86345013	88732142	836	3.64E-05	1.26E-08	0.746
7	89271899	91173749	772	2.32E-05	1.34E-08	0.841
7	91174778	93339775	616	-1.82E-05	9.24E-09	0.850
7	93340137	95171731	630	1.31E-04	1.23E-08	0.238

7	95419256	96611864	459	-1.60E-05	7.86E-09	0.857
7	96613246	98151455	495	4.59E-05	9.78E-09	0.643
7	98152191	100555361	664	1.13E-04	1.32E-08	0.324
7	100606141	101730309	387	-3.90E-05	9.75E-09	0.693
7	101731567	103193331	317	-1.12E-05	5.49E-09	0.880
7	103193651	104157576	550	2.72E-05	1.12E-08	0.797
7	104158491	105425027	480	-3.78E-06	7.43E-09	0.965
7	105427565	106052506	320	6.24E-05	7.93E-09	0.483
7	106052873	107305328	456	6.21E-05	8.40E-09	0.498
7	107306421	108375939	543	-7.07E-05	9.08E-09	0.458
7	108376173	109584769	351	-1.38E-05	5.84E-09	0.857
7	109587777	111447153	592	-4.19E-05	9.87E-09	0.673
7	111450919	113336970	590	7.13E-06	1.13E-08	0.946
7	113337327	116315205	824	-4.93E-05	1.37E-08	0.673
7	116317043	118347634	568	8.57E-05	9.56E-09	0.381
7	118348359	121094386	572	1.69E-05	7.90E-09	0.849
7	121094460	122368663	412	4.56E-05	7.80E-09	0.606
7	122369853	124154451	639	-7.72E-05	9.49E-09	0.428
7	124155319	125386718	379	4.92E-05	5.19E-09	0.495
7	125387168	126516683	442	-6.39E-05	6.92E-09	0.442
7	126517818	127905983	588	3.50E-05	8.61E-09	0.706
7	127907054	128554075	209	-2.28E-05	4.11E-09	0.723
7	128555262	130074230	546	-5.64E-05	9.91E-09	0.571
7	130075370	130797123	249	-1.58E-05	6.19E-09	0.841
7	130797776	131856855	497	-1.95E-04	1.13E-08	0.067
7	131857419	134306672	895	1.17E-04	1.44E-08	0.328
7	134307114	135464701	503	1.22E-04	9.28E-09	0.205
7	135466022	137137511	701	-3.07E-06	1.34E-08	0.979
7	137138736	138742981	585	-3.44E-06	1.48E-08	0.977
7	138744100	141106552	843	1.89E-05	1.46E-08	0.876
7	141109797	142655113	558	1.09E-05	8.79E-09	0.907
7	142655717	144274735	430	-2.20E-05	7.32E-09	0.797
7	144276424	146018520	551	-1.95E-05	1.02E-08	0.846
7	146023023	147204086	503	9.33E-05	8.88E-09	0.322
7	147204549	148619685	683	8.25E-05	1.31E-08	0.471
7	148620581	150491084	651	6.41E-05	1.21E-08	0.560
7	150491851	151390799	462	5.32E-05	1.11E-08	0.613

7	151391044	152409155	286	2.17E-05	1.08E-08	0.834
7	152410248	153080056	287	-8.01E-05	7.70E-09	0.361
7	153081698	153934166	317	8.93E-06	7.40E-09	0.917
7	154964316	155379409	238	8.40E-05	8.78E-09	0.370
7	153934210	154963787	619	1.76E-04	1.39E-08	0.135
7	155380353	156814410	650	-3.32E-05	1.51E-08	0.787
7	156815867	158029340	505	1.70E-04	1.15E-08	0.113
7	158030463	158759939	285	-1.65E-05	4.71E-09	0.809
7	158760677	159127587	134	6.20E-06	4.19E-09	0.924
8	246934	1162157	487	3.49E-05	1.31E-08	0.760
8	1162454	1625424	272	-1.16E-05	7.53E-09	0.894
8	1625732	2042364	302	7.23E-05	9.88E-09	0.467
8	2043187	2680255	316	5.52E-05	9.59E-09	0.573
8	2681051	3358423	619	-1.11E-05	1.72E-08	0.933
8	3358433	3584832	296	-1.32E-05	7.40E-09	0.878
8	3585058	4071438	625	6.12E-05	1.50E-08	0.616
8	4071516	4274957	255	1.98E-05	6.38E-09	0.804
8	4275286	4479596	219	1.66E-05	7.51E-09	0.848
8	4479836	4754328	302	4.74E-05	7.12E-09	0.574
8	4755088	5146808	311	-6.52E-05	7.70E-09	0.457
8	5147734	5781592	595	-9.52E-05	1.43E-08	0.426
8	5781947	6017670	226	-1.03E-04	4.81E-09	0.137
8	6018292	6350873	272	-2.35E-05	5.73E-09	0.756
8	6352358	7793703	489	-1.36E-05	1.25E-08	0.903
8	7793837	8735213	411	-1.46E-06	8.82E-09	0.988
8	10117411	10535826	286	-1.04E-06	7.64E-09	0.991
8	8736386	10117293	799	3.21E-05	1.83E-08	0.813
8	10537940	11249261	408	-4.74E-05	7.99E-09	0.596
8	11250848	11836622	364	-5.28E-05	1.02E-08	0.601
8	11838355	12975295	284	1.19E-05	7.03E-09	0.888
8	12975578	13940652	686	-6.11E-05	1.56E-08	0.625
8	13942500	14876660	522	-1.02E-04	1.09E-08	0.332
8	14877288	15273777	320	-1.92E-05	6.24E-09	0.808
8	15274608	16087631	534	-3.86E-05	1.24E-08	0.728
8	16087729	17579518	802	-1.57E-05	1.92E-08	0.910
8	17581106	18670645	721	-1.66E-04	1.51E-08	0.178
8	18670908	19490180	560	-1.19E-04	1.34E-08	0.304

8	21022075	21402658	257	-3.49E-05	5.64E-09	0.643
8	19491428	21021401	888	1.65E-04	2.18E-08	0.265
8	21404381	22539316	482	6.92E-05	9.22E-09	0.471
8	22539641	23548146	588	-5.17E-05	5.44E-08	0.825
8	23548749	24456913	316	2.07E-05	8.04E-09	0.817
8	24457122	25461315	424	2.69E-05	7.44E-09	0.755
8	25461344	26681450	716	2.34E-05	2.01E-08	0.869
8	26681799	27404354	421	2.99E-05	8.46E-09	0.745
8	27405207	27759126	213	-1.56E-05	3.65E-09	0.797
8	27759839	29277590	622	-1.34E-04	1.25E-08	0.230
8	31947880	32448740	282	-6.26E-05	6.11E-09	0.424
8	29277652	31947675	952	-3.90E-05	1.95E-08	0.780
8	32449533	33980486	355	-3.35E-05	5.77E-09	0.659
8	33981742	35313077	399	2.45E-05	5.62E-09	0.744
8	35314837	37375458	435	-3.23E-05	7.59E-09	0.711
8	37376914	39779989	596	-1.55E-04	1.25E-08	0.165
8	39781444	40415799	338	1.29E-06	7.41E-09	0.988
8	40417433	41718356	592	-8.43E-05	1.32E-08	0.463
8	41719750	42501174	186	6.28E-05	3.25E-09	0.271
8	42501717	49028667	387	-6.24E-06	4.74E-09	0.928
8	49038732	52156842	813	1.29E-05	1.16E-08	0.905
8	52157415	54115712	718	-2.33E-05	1.27E-08	0.836
8	54116775	55914038	657	1.78E-05	1.34E-08	0.878
8	55914314	57653028	655	5.60E-05	1.30E-08	0.623
8	57655067	58674153	403	-8.10E-05	7.40E-09	0.347
8	58675131	59568410	383	1.12E-05	8.14E-09	0.901
8	59570176	61809929	767	-1.94E-04	1.45E-08	0.107
8	61810694	63710298	770	-1.20E-04	1.45E-08	0.319
8	63711348	65193342	460	-2.93E-05	8.56E-09	0.751
8	65195953	66024947	259	-2.83E-05	4.73E-09	0.681
8	66025862	67192642	386	-5.49E-05	6.93E-09	0.509
8	67193708	69108839	502	-1.32E-05	1.00E-08	0.895
8	69111244	70003448	401	-1.20E-05	9.41E-09	0.902
8	70003607	72013005	726	9.30E-05	1.45E-08	0.440
8	72014069	72749564	426	1.14E-04	1.06E-08	0.267
8	72751469	73423214	352	-1.33E-04	7.95E-09	0.136
8	73423786	74996061	687	2.31E-05	1.39E-08	0.845

8	74998868	76294909	508	6.11E-05	1.01E-08	0.543
8	76295292	78015335	544	-7.52E-06	9.76E-09	0.939
8	78015611	78632215	197	2.78E-06	3.34E-09	0.962
8	78632893	79831888	374	4.70E-06	5.85E-09	0.951
8	79832211	80654655	272	2.68E-05	5.49E-09	0.717
8	80656206	81330540	223	-7.12E-06	4.72E-09	0.918
8	81331077	83726996	809	-7.56E-05	1.62E-08	0.552
8	83727741	85093121	385	2.47E-05	7.58E-09	0.777
8	85094979	86979097	352	6.73E-05	5.23E-09	0.352
8	86990451	87562786	212	2.01E-05	3.23E-09	0.724
8	87563878	89083319	540	-3.87E-05	1.01E-08	0.700
8	89083979	90605445	456	2.84E-05	7.49E-09	0.743
8	90607207	92342283	521	-3.24E-05	7.95E-09	0.717
8	92344138	94269630	544	8.77E-08	1.13E-08	0.999
8	94272436	95810190	578	-5.84E-05	1.23E-08	0.598
8	95810772	96533604	390	-2.62E-06	8.53E-09	0.977
8	96534326	97318660	395	5.81E-06	8.92E-09	0.951
8	97320605	98867870	621	-7.55E-06	1.20E-08	0.945
8	98869940	101450242	829	2.03E-05	1.30E-08	0.859
8	101451169	102937063	655	1.46E-04	1.47E-08	0.228
8	102944509	103730879	375	-4.51E-06	9.46E-09	0.963
8	103970185	105389708	565	2.29E-06	9.93E-09	0.982
8	105391381	107409808	782	6.88E-05	1.53E-08	0.578
8	107410067	108108114	324	2.49E-05	5.96E-09	0.747
8	108115161	108646193	244	-2.90E-05	5.51E-09	0.696
8	108646968	110761074	742	-4.63E-05	1.25E-08	0.679
8	110761776	112611637	402	2.15E-05	6.62E-09	0.792
8	112612842	114393054	481	3.16E-06	6.93E-09	0.970
8	114394256	116095059	613	2.97E-05	1.01E-08	0.767
8	116095815	117130004	272	9.52E-05	6.59E-09	0.241
8	117131947	118622352	563	1.12E-04	1.17E-08	0.301
8	118623844	120216957	717	1.31E-04	1.50E-08	0.284
8	120217356	121767384	601	6.38E-05	1.02E-08	0.528
8	121770585	123545992	691	1.67E-04	1.51E-08	0.174
8	123547148	124483938	403	9.73E-05	9.56E-09	0.320
8	124486753	125247268	404	-3.53E-05	9.52E-09	0.717
8	125248576	126268046	461	-5.85E-05	1.06E-08	0.569

8	126269921	127434945	507	-1.64E-05	1.08E-08	0.875
8	127436003	128162974	337	4.21E-05	1.67E-07	0.918
8	128166556	128542444	193	-3.13E-05	1.59E-07	0.937
8	128543956	129297518	432	-2.31E-04	4.47E-08	0.275
8	129297807	130379330	412	-4.01E-05	7.95E-09	0.653
8	130380171	130812339	230	-6.14E-05	4.98E-09	0.384
8	130814803	131553214	284	-3.10E-05	5.36E-09	0.672
8	131553327	132358990	430	7.22E-05	9.23E-09	0.452
8	132359241	133454722	553	3.59E-05	1.07E-08	0.729
8	133455530	134566686	734	4.25E-05	1.53E-08	0.731
8	134567348	135269827	423	1.73E-05	1.09E-08	0.868
8	135270868	136209239	515	-1.06E-04	1.13E-08	0.319
8	136209325	137306730	483	-6.10E-05	8.15E-09	0.499
8	137307361	138223632	346	2.34E-07	6.32E-09	0.998
8	138224181	139194661	572	5.14E-05	1.12E-08	0.628
8	139195331	140093896	520	2.17E-05	1.30E-08	0.849
8	140094627	141355502	730	8.79E-05	1.60E-08	0.487
8	143750619	146303789	713	2.45E-05	1.89E-08	0.859
8	141358681	143750205	919	-8.02E-05	2.04E-08	0.574
9	342841	1006606	532	-3.69E-06	1.07E-08	0.971
9	1007923	1792147	679	2.73E-05	1.26E-08	0.807
9	1792277	2665240	685	-8.04E-05	1.57E-08	0.521
9	2666964	3348816	358	-3.79E-04	1.19E-08	0.001
9	4380096	4590305	142	-5.69E-06	3.41E-09	0.922
9	3349487	4378727	698	9.10E-06	1.66E-08	0.944
9	4591655	5273194	341	1.27E-04	5.53E-09	0.087
9	5273896	6144065	383	1.49E-06	6.11E-09	0.985
9	6144333	7232230	569	-5.10E-05	1.03E-08	0.616
9	7232510	7757891	465	-4.69E-05	9.14E-09	0.624
9	7758579	8235898	325	4.39E-05	6.16E-09	0.576
9	9375194	9793342	253	-3.67E-05	4.88E-09	0.599
9	8236122	9374664	803	1.49E-04	1.80E-08	0.265
9	9793846	10358286	350	-5.26E-05	6.70E-09	0.520
9	10358672	10879071	263	3.35E-05	5.67E-09	0.656
9	10879188	11616822	305	-2.57E-05	5.66E-09	0.733
9	11884425	12274220	180	-2.60E-05	3.03E-09	0.637
9	12274774	12778224	274	-1.76E-05	4.90E-09	0.801

9	12780295	13797055	477	-1.44E-05	9.32E-09	0.882
9	13797496	14712257	603	-1.98E-06	1.46E-08	0.987
9	14714681	16054406	644	-2.12E-04	1.06E-08	0.039
9	16056127	16833797	538	-5.03E-05	1.13E-08	0.636
9	16836011	17627988	363	1.47E-05	5.42E-09	0.842
9	17628726	18659920	604	-2.53E-05	1.05E-08	0.805
9	18660695	19129349	353	-1.36E-04	7.22E-09	0.111
9	19456098	20324812	481	2.01E-05	9.02E-09	0.833
9	20324848	21549638	502	9.13E-06	7.69E-09	0.917
9	21550959	22968575	596	-7.06E-05	9.87E-09	0.477
9	22968789	24154728	439	3.20E-05	1.03E-08	0.753
9	24155142	25425160	409	8.26E-05	8.62E-09	0.374
9	25425511	26317104	404	6.19E-05	9.52E-09	0.526
9	26317973	27335346	535	-6.11E-05	1.04E-08	0.549
9	27336207	28252383	496	3.56E-06	1.08E-08	0.973
9	28253887	29622939	659	2.07E-05	1.02E-08	0.838
9	29623053	30819576	414	5.01E-05	5.11E-09	0.483
9	30821676	31836697	373	-8.64E-05	5.50E-09	0.244
9	31837040	32249649	172	1.72E-06	2.33E-09	0.972
9	32250247	33186788	463	-9.45E-05	7.16E-09	0.264
9	33187897	34769004	521	3.85E-05	8.00E-09	0.667
9	36714252	37678932	456	2.30E-05	8.26E-09	0.800
9	34771534	36712124	645	1.05E-04	9.96E-09	0.291
9	37679713	42220384	538	1.50E-04	1.03E-08	0.140
9	69576527	71968825	444	-3.63E-05	7.49E-09	0.675
9	71969482	73398448	622	-1.34E-05	8.49E-09	0.884
9	73399750	73916072	309	6.19E-06	4.41E-09	0.926
9	73916583	74600739	231	6.08E-06	4.41E-09	0.927
9	74600822	75517582	356	-4.31E-05	4.74E-09	0.531
9	75519251	76785168	378	3.63E-05	5.76E-09	0.633
9	76786297	78214833	562	4.93E-05	9.77E-09	0.618
9	78215299	78900183	469	-2.29E-05	1.30E-08	0.841
9	81066078	81860861	384	-5.72E-05	7.12E-09	0.498
9	78901112	81064897	966	-4.69E-06	1.71E-08	0.971
9	81863722	82348771	246	-4.04E-05	4.01E-09	0.523
9	82349606	83041982	352	9.27E-06	7.00E-09	0.912
9	83043376	83455568	219	2.94E-05	3.68E-09	0.628

9	83455867	84388566	420	-3.16E-05	7.87E-09	0.722
9	84389168	86180165	683	9.03E-05	1.20E-08	0.409
9	87571959	87811640	150	1.10E-05	2.40E-09	0.822
9	86181461	87571563	628	-1.47E-04	9.30E-09	0.126
9	87813612	88867151	355	6.50E-05	5.36E-09	0.375
9	88869630	89948499	516	-1.32E-06	9.14E-09	0.989
9	89949778	90198251	198	2.21E-05	2.92E-09	0.682
9	90198375	90893610	336	-2.22E-05	6.29E-09	0.780
9	90893774	91608628	247	3.95E-06	4.27E-09	0.952
9	91610543	92342927	327	-1.92E-05	6.23E-09	0.808
9	92443570	93441674	461	1.60E-04	8.16E-09	0.076
9	93442205	94163515	361	1.14E-04	7.05E-09	0.176
9	94164373	95854872	626	1.72E-05	7.26E-09	0.840
9	95855100	97719592	729	5.31E-06	1.03E-08	0.958
9	97720818	98889276	456	2.09E-05	6.63E-09	0.798
9	98891743	100917602	625	5.29E-05	8.14E-09	0.558
9	100919318	101473404	376	-3.20E-05	7.52E-09	0.713
9	103544757	103969076	179	3.88E-06	1.86E-09	0.928
9	101474837	103543692	676	-5.52E-05	8.20E-09	0.542
9	103969339	104552711	293	4.20E-05	4.96E-09	0.551
9	104553938	105041375	303	-1.87E-05	5.22E-09	0.795
9	105041756	105743445	320	1.53E-05	4.83E-09	0.826
9	105744075	106487777	333	2.53E-05	5.67E-09	0.737
9	106488291	107545903	496	2.03E-05	5.51E-09	0.784
9	107546901	108751162	555	1.84E-05	8.98E-09	0.846
9	108754828	109972524	499	-4.36E-05	1.46E-08	0.718
9	109974206	111137001	673	1.19E-05	1.73E-08	0.928
9	111138232	111997222	549	6.66E-05	8.40E-09	0.467
9	112001473	112593531	326	1.34E-04	5.42E-09	0.070
9	112594858	113255402	400	-4.18E-05	8.60E-09	0.652
9	113255544	114077164	462	2.27E-05	8.09E-09	0.801
9	114078103	114828332	329	-1.92E-05	5.36E-09	0.793
9	114830330	115391053	230	1.51E-06	2.54E-09	0.976
9	115393806	116148630	399	-2.53E-05	7.06E-09	0.764
9	116149967	117306807	686	-1.44E-04	1.29E-08	0.205
9	117307880	118569429	682	3.80E-05	1.10E-08	0.717
9	118570426	120030166	743	4.82E-05	1.33E-08	0.676

9	121009165	121318886	151	1.83E-05	2.48E-09	0.713
9	120030269	121008844	499	9.80E-05	8.75E-09	0.295
9	121319662	122258204	434	-4.33E-05	6.85E-09	0.601
9	122258577	124870612	937	-6.92E-05	1.30E-08	0.543
9	124871322	125542861	333	-9.61E-05	6.30E-09	0.226
9	125545194	126926376	448	-4.05E-05	6.55E-09	0.616
9	126927204	128926989	624	-8.23E-05	6.91E-09	0.322
9	128927088	129477470	292	-5.11E-05	6.64E-09	0.530
9	129477886	131429495	582	-1.61E-05	7.42E-09	0.851
9	131430602	132891863	504	-3.69E-05	8.95E-09	0.696
9	132895131	133640777	304	-2.54E-05	7.97E-09	0.776
9	133642918	134639655	431	-5.85E-06	7.08E-09	0.945
9	134640928	135401279	345	5.04E-05	7.34E-09	0.556
9	135401721	136417159	500	1.32E-04	9.22E-09	0.168
9	136429816	136979539	317	-7.25E-05	7.80E-09	0.411
9	136979744	137928846	495	8.39E-05	1.28E-08	0.458
9	137929853	138817142	510	-9.90E-05	1.36E-08	0.396
9	138817178	141146684	716	-3.11E-05	1.25E-08	0.780
10	60969	747115	150	-5.03E-05	2.90E-09	0.350
10	747772	1330771	280	5.32E-05	5.24E-09	0.462
10	1331955	1609370	171	-2.09E-05	3.96E-09	0.741
10	1610839	2104472	389	-2.50E-05	8.32E-09	0.784
10	2106418	3143283	688	-5.19E-05	1.45E-08	0.666
10	3144251	3785605	441	5.73E-05	8.93E-09	0.545
10	5359010	6316910	614	6.70E-05	1.22E-08	0.544
10	3786476	5358542	954	2.05E-04	1.88E-08	0.136
10	6318458	7639684	881	1.07E-04	2.22E-08	0.471
10	9433090	10540465	440	2.43E-05	8.05E-09	0.787
10	7639932	9432277	1108	-5.10E-05	2.14E-08	0.727
10	10542217	12092029	827	-4.55E-05	1.74E-08	0.730
10	12092208	12798954	370	-3.97E-05	9.30E-09	0.681
10	12799286	13718384	597	-1.64E-04	1.29E-08	0.150
10	13719970	14411956	499	-4.10E-05	1.21E-08	0.709
10	14412084	15303527	488	2.56E-05	1.18E-08	0.814
10	15303983	16443442	476	1.31E-05	7.82E-09	0.883
10	16444693	17591626	663	-8.00E-05	1.33E-08	0.489
10	17591919	19202731	694	1.33E-05	1.20E-08	0.903

10	19203300	20131614	450	-1.18E-05	7.37E-09	0.890
10	20132516	21243558	679	-3.72E-05	1.27E-08	0.742
10	21244087	23696890	630	-4.67E-05	1.19E-08	0.669
10	23698033	25414215	686	-5.86E-05	1.12E-08	0.581
10	25416598	26631987	555	1.68E-05	8.83E-09	0.858
10	26633775	27667904	403	7.99E-05	6.63E-09	0.327
10	27668052	28734655	508	-1.14E-04	9.75E-09	0.246
10	28735045	29174963	234	-3.01E-05	3.86E-09	0.628
10	29175101	29961838	534	8.31E-05	1.16E-08	0.441
10	29962511	30653567	387	3.03E-06	8.16E-09	0.973
10	30654528	31479103	433	2.95E-07	7.86E-09	0.997
10	31479422	33656047	778	-9.52E-05	1.11E-08	0.366
10	33656119	36017592	829	-6.52E-05	1.24E-08	0.559
10	36020445	36884906	379	1.14E-05	7.36E-09	0.894
10	36885921	37845308	312	8.38E-06	5.11E-09	0.907
10	38367233	43220539	214	3.58E-07	2.55E-09	0.994
10	43222372	43605902	162	-1.28E-05	3.57E-09	0.830
10	43606687	45247184	692	4.27E-05	1.07E-08	0.680
10	45247418	46769302	372	1.33E-04	1.49E-08	0.275
10	47768664	49812917	342	-4.34E-05	6.83E-09	0.599
10	49816189	50839403	568	4.58E-05	9.74E-09	0.643
10	50840736	52086621	206	1.50E-04	2.28E-08	0.320
10	52086939	52707026	235	-1.61E-05	3.42E-09	0.784
10	52708822	53362540	343	9.29E-06	6.51E-09	0.908
10	53362932	54413554	512	5.81E-06	9.49E-09	0.952
10	54413909	55334166	522	-1.29E-04	9.82E-09	0.193
10	55334740	56204677	453	2.62E-05	6.33E-09	0.742
10	56207098	56883966	278	6.93E-05	6.78E-09	0.400
10	56885639	58806290	703	-3.91E-05	9.32E-09	0.686
10	59158617	59822195	312	5.94E-05	4.39E-09	0.370
10	59822974	60375613	205	2.98E-05	2.49E-09	0.550
10	62558842	64263322	625	-8.42E-05	1.05E-08	0.411
10	60375857	62558737	969	-1.53E-04	1.62E-08	0.230
10	64263402	65469133	435	1.42E-05	6.32E-09	0.858
10	65735028	66980708	393	-3.01E-05	6.04E-09	0.699
10	66980980	67545544	227	1.80E-05	3.96E-09	0.775
10	67546331	68429563	514	-4.89E-05	9.26E-09	0.611

10	68430236	69893580	549	-1.94E-05	9.84E-09	0.845
10	69896336	71052508	439	2.47E-05	7.61E-09	0.777
10	71052888	72479853	927	1.92E-05	2.29E-08	0.899
10	72480072	73612902	783	4.97E-05	1.87E-08	0.716
10	73614288	75696752	545	1.29E-05	4.67E-09	0.850
10	75700056	78638957	888	9.97E-06	1.15E-08	0.926
10	78642246	79740262	593	-2.79E-05	1.06E-08	0.787
10	79742617	80874092	594	8.68E-05	1.32E-08	0.451
10	80874523	81881954	306	2.96E-04	1.91E-08	0.033
10	81882370	82378624	276	2.10E-05	5.20E-09	0.771
10	82379403	82881996	242	6.19E-05	5.02E-09	0.382
10	82883949	83597042	269	-2.01E-05	4.38E-09	0.762
10	83597818	84383108	342	-1.69E-05	5.29E-09	0.816
10	84383277	85424102	486	9.56E-05	7.18E-09	0.259
10	85424493	86459792	528	3.84E-05	1.02E-08	0.704
10	86460169	87285511	356	3.72E-06	5.12E-09	0.959
10	87285907	88450872	496	-2.09E-05	9.84E-09	0.833
10	88451869	89845801	371	1.09E-05	6.60E-09	0.893
10	89846944	90734906	410	-9.56E-05	7.38E-09	0.266
10	90735832	91959588	592	-2.10E-05	9.83E-09	0.832
10	91960142	92682235	319	-3.08E-06	4.58E-09	0.964
10	92682369	94196248	469	3.77E-05	7.09E-09	0.655
10	94196690	95064828	355	2.86E-05	4.64E-09	0.674
10	95065655	95946938	409	-3.26E-05	7.78E-09	0.711
10	95949432	97984754	852	-9.43E-05	1.11E-08	0.370
10	97985690	99554180	665	-5.95E-05	1.09E-08	0.568
10	99554895	100240681	333	-2.10E-05	5.82E-09	0.783
10	100241204	102089663	714	3.59E-06	9.79E-09	0.971
10	102090924	104207799	687	-3.82E-05	9.49E-09	0.695
10	104208761	106139081	683	-9.82E-05	9.98E-09	0.325
10	106139294	107874146	733	-9.91E-05	1.08E-08	0.340
10	107875182	109577686	742	1.01E-04	1.33E-08	0.382
10	109578567	110311094	318	-2.18E-05	4.81E-09	0.753
10	110311425	112559414	764	-4.14E-05	1.13E-08	0.697
10	112560012	113839866	517	-7.69E-05	7.42E-09	0.372
10	113845990	115698315	909	-1.79E-04	1.93E-08	0.197
10	117705146	118416795	358	-6.52E-05	5.90E-09	0.396

10	115700005	117702645	679	-9.40E-05	1.02E-08	0.351
10	118417453	119697663	620	-6.76E-05	1.31E-08	0.555
10	119698700	120994671	529	-9.73E-05	1.03E-08	0.339
10	120997666	121874991	393	5.65E-05	7.32E-09	0.509
10	121875531	122805770	507	-1.02E-05	1.01E-08	0.920
10	122807254	123853223	520	1.78E-04	1.27E-08	0.114
10	123855124	124894743	543	-2.96E-05	1.01E-08	0.768
10	124894909	125868860	561	5.70E-05	1.28E-08	0.614
10	125919795	126739513	451	9.09E-05	1.17E-08	0.400
10	126739798	127820978	515	6.98E-05	9.06E-09	0.463
10	127821105	128409952	404	-1.11E-05	9.07E-09	0.907
10	128410393	129482709	632	-1.54E-04	1.40E-08	0.191
10	129483173	130282731	491	5.54E-05	1.11E-08	0.599
10	130282817	131192968	536	-2.88E-05	1.35E-08	0.804
10	131195759	132251820	595	-3.40E-05	1.21E-08	0.757
10	132252499	132964299	471	3.77E-05	1.06E-08	0.714
10	132966372	133355925	285	-3.41E-06	7.98E-09	0.970
10	133356417	134858046	637	-1.02E-04	1.30E-08	0.369
10	134858720	135524335	224	8.07E-06	3.74E-09	0.895
11	333347	1057948	319	-2.53E-05	5.45E-09	0.731
11	1058583	1861912	307	6.25E-05	1.18E-08	0.565
11	1862168	2422034	203	-1.93E-04	1.44E-08	0.107
11	2423545	3660412	521	-1.08E-04	1.32E-08	0.348
11	3661873	4745931	518	-6.35E-05	1.01E-08	0.528
11	4746391	5529152	652	-1.45E-05	1.07E-08	0.888
11	5532222	6010718	357	7.81E-05	6.60E-09	0.336
11	6011761	6917273	554	3.82E-06	1.02E-08	0.970
11	6917772	7715871	483	-5.45E-05	1.19E-08	0.617
11	7717884	9068716	661	7.17E-05	1.21E-08	0.515
11	9069046	10370675	439	-9.23E-05	6.95E-09	0.268
11	10371614	11361034	681	-4.56E-05	1.46E-08	0.706
11	11361458	12018942	492	7.00E-06	1.13E-08	0.947
11	12019010	13133902	717	-6.12E-05	1.66E-08	0.634
11	13134600	14934686	720	3.73E-05	1.06E-08	0.718
11	14936943	16789155	641	-5.29E-06	9.12E-09	0.956
11	16790799	18788126	916	2.14E-04	1.58E-08	0.088
11	18789820	20113591	791	1.36E-04	1.76E-08	0.304

11	20115277	21773251	1016	1.86E-05	2.27E-08	0.902
11	21773680	23111273	502	-9.63E-05	8.30E-09	0.290
11	24750113	25106557	162	-3.51E-06	2.71E-09	0.946
11	23112584	24749839	708	1.87E-05	1.41E-08	0.875
11	25109191	26046095	403	-3.40E-05	6.43E-09	0.671
11	26046627	27018778	418	-5.71E-05	6.67E-09	0.484
11	27019873	28741185	545	6.08E-05	7.96E-09	0.496
11	28742220	30056918	404	2.59E-05	6.98E-09	0.756
11	30057440	31853968	575	-1.06E-05	6.97E-09	0.899
11	31856654	33383448	566	-6.94E-05	9.25E-09	0.471
11	33384046	34321466	430	-3.57E-05	8.90E-09	0.705
11	34321876	35034307	445	-2.28E-04	2.20E-08	0.125
11	35036029	36117340	598	6.69E-05	1.25E-08	0.550
11	36118027	37838625	746	4.67E-05	1.44E-08	0.698
11	37839347	40357803	640	-9.99E-06	9.90E-09	0.920
11	40359663	40716222	154	-2.95E-05	2.51E-09	0.555
11	40716823	42243196	591	-1.22E-05	9.90E-09	0.902
11	42244050	42854352	256	2.26E-05	3.91E-09	0.717
11	42854878	43952796	470	-1.15E-05	8.13E-09	0.898
11	43953774	46245900	1080	3.20E-05	2.48E-08	0.839
11	46252956	47893120	417	-3.33E-05	8.10E-09	0.711
11	48351331	49659131	215	-2.07E-05	2.42E-09	0.674
11	49660004	55116367	287	-4.20E-05	3.00E-09	0.444
11	55116451	56160712	419	-3.10E-05	4.66E-09	0.650
11	56625775	58455947	713	-4.53E-05	8.96E-09	0.633
11	58457495	59615953	298	1.87E-05	3.50E-09	0.752
11	59620206	61870732	816	2.03E-05	1.45E-08	0.866
11	61873755	63153602	403	-1.04E-04	6.09E-09	0.181
11	63154309	66835194	1070	6.18E-05	1.51E-08	0.615
11	66835372	68515239	413	-2.61E-05	1.21E-08	0.813
11	68521072	69508567	391	-1.71E-04	6.81E-08	0.511
11	69509343	70613763	469	1.69E-04	1.34E-08	0.144
11	70616465	71842303	362	2.10E-05	5.58E-09	0.779
11	71842976	74158869	681	-5.50E-05	1.13E-08	0.605
11	74159542	76431100	890	1.23E-04	1.86E-08	0.368
11	76432156	77903706	543	1.46E-06	7.07E-09	0.986
11	79723675	80720242	465	1.42E-04	1.05E-08	0.164

11	77904339	79723318	879	-3.56E-05	1.51E-08	0.772
11	80721384	81653514	543	-1.18E-05	1.01E-08	0.907
11	81654617	82844918	481	-1.43E-05	8.30E-09	0.875
11	82847086	83575703	267	-1.24E-05	3.60E-09	0.836
11	83576460	84208616	304	3.97E-05	5.49E-09	0.592
11	84212522	84618654	169	1.73E-05	3.54E-09	0.771
11	84618788	86623913	828	4.48E-07	1.38E-08	0.997
11	86625137	87429545	370	-2.20E-05	5.74E-09	0.771
11	87431647	87827395	167	1.72E-05	3.57E-09	0.773
11	87827514	89208590	526	7.76E-05	9.58E-09	0.428
11	89441680	91115475	480	9.63E-07	6.47E-09	0.990
11	91118661	91676282	198	1.71E-05	3.56E-09	0.775
11	91677374	92626428	367	9.02E-06	4.97E-09	0.898
11	92627643	93270431	278	-9.71E-06	4.82E-09	0.889
11	93272186	94389331	421	3.46E-05	6.58E-09	0.670
11	94391845	95329108	415	7.58E-05	9.17E-09	0.429
11	95332310	96848965	669	7.00E-05	1.25E-08	0.531
11	96849530	98703542	778	3.07E-05	1.52E-08	0.803
11	98703733	99613901	507	4.11E-05	1.03E-08	0.686
11	101158517	101892922	344	1.59E-05	4.66E-09	0.816
11	99614560	101156501	812	-1.59E-04	1.58E-08	0.207
11	101894471	102668702	378	-7.16E-05	9.44E-09	0.461
11	102668882	103491310	383	-1.01E-05	6.47E-09	0.900
11	103491586	104871322	551	4.83E-05	8.36E-09	0.597
11	104872103	106251514	419	6.42E-05	5.65E-09	0.393
11	106252171	107489662	535	-6.38E-05	1.08E-08	0.539
11	107491606	108495975	326	-6.83E-05	4.75E-09	0.322
11	108496357	110701645	740	7.68E-05	1.11E-08	0.466
11	110702636	111981853	468	1.10E-04	6.86E-09	0.182
11	111985737	113103996	469	1.15E-05	7.58E-09	0.895
11	113105405	113958177	414	-1.30E-04	1.79E-08	0.332
11	113959061	114742322	447	-2.42E-05	8.89E-09	0.798
11	114743956	115627217	415	6.46E-05	8.16E-09	0.474
11	115652723	117097952	688	-1.73E-05	1.48E-08	0.887
11	118899218	120171371	573	1.80E-05	1.17E-08	0.868
11	117098436	118898319	897	1.75E-04	1.69E-08	0.178
11	120173354	121660636	712	-1.24E-04	1.25E-08	0.269

11	121661507	123289851	720	-6.49E-05	1.65E-08	0.613
11	123291301	124403522	616	4.29E-05	1.16E-08	0.690
11	124404297	125984562	770	1.46E-04	1.60E-08	0.249
11	125984970	126772347	528	-1.94E-05	1.12E-08	0.854
11	126772905	127715860	459	5.34E-05	8.95E-09	0.572
11	127716140	129186210	717	4.53E-05	1.44E-08	0.706
11	129187995	129838375	322	-1.06E-04	7.62E-09	0.225
11	129839528	130892029	436	-1.62E-04	9.21E-09	0.091
11	130894131	131870763	676	1.79E-05	1.54E-08	0.886
11	131871467	132386355	340	-5.78E-05	5.77E-09	0.446
11	132386834	133679495	782	-1.01E-04	1.94E-08	0.470
11	133679758	134946382	714	-1.84E-04	1.70E-08	0.158
12	68657	665200	216	-2.80E-05	4.71E-09	0.683
12	665655	1602602	326	6.09E-05	6.10E-09	0.436
12	1602652	2176046	281	2.81E-05	7.10E-09	0.739
12	2176265	2886299	324	5.92E-06	8.36E-09	0.948
12	2886946	3558488	354	9.32E-05	8.48E-09	0.312
12	3559429	4693778	654	-1.28E-04	1.70E-08	0.326
12	4695425	5877653	808	6.05E-05	1.71E-08	0.644
12	5878686	6879279	440	8.81E-05	1.17E-08	0.416
12	8658644	10477423	257	-7.30E-05	5.12E-09	0.308
12	10477712	11684725	445	-2.30E-05	6.61E-09	0.777
12	11687997	12530718	423	-2.58E-04	1.01E-08	0.010
12	12533494	13362446	379	-2.33E-05	8.38E-09	0.799
12	13362818	14658341	700	-3.44E-06	1.78E-08	0.979
12	14659441	16423287	588	-8.42E-05	9.52E-09	0.388
12	16423998	18466533	744	-6.40E-05	1.35E-08	0.582
12	18467618	19924288	581	-3.77E-05	1.23E-08	0.734
12	19925541	21568051	876	5.06E-06	1.42E-08	0.966
12	21569730	22325878	305	-3.20E-05	4.82E-09	0.645
12	22327634	22943718	204	4.21E-05	3.82E-09	0.496
12	22944771	23819079	359	-1.00E-04	1.02E-08	0.322
12	23819364	24545631	412	1.31E-04	1.03E-08	0.197
12	24545834	25991825	716	1.86E-05	1.32E-08	0.872
12	25992543	27529660	775	-4.31E-05	1.56E-08	0.730
12	27529712	29111136	699	9.17E-05	1.10E-08	0.381
12	29111480	30354932	708	-1.05E-04	1.13E-08	0.323

12	30355547	31067009	415	5.08E-05	6.14E-09	0.517
12	31067490	32081072	396	-4.82E-05	8.48E-09	0.600
12	32082695	32645755	311	-2.84E-05	6.49E-09	0.725
12	32645848	33435901	367	-1.23E-05	5.84E-09	0.873
12	33436298	38231063	325	-1.02E-05	4.00E-09	0.872
12	38233264	40017827	410	-1.11E-05	4.85E-09	0.873
12	40022029	41015211	475	-4.80E-06	7.45E-09	0.956
12	41015333	42346846	665	5.02E-05	1.03E-08	0.620
12	42347363	43074061	260	5.96E-05	3.85E-09	0.336
12	43074434	43982146	422	7.09E-06	6.25E-09	0.928
12	43982342	44922863	221	-6.09E-05	3.36E-09	0.293
12	44923334	46022099	342	-1.28E-05	5.17E-09	0.859
12	46023426	47504173	481	7.91E-05	7.15E-09	0.350
12	47505424	48267360	361	1.03E-05	8.31E-09	0.910
12	48267635	48965461	366	-4.60E-05	1.03E-08	0.650
12	48967935	50160195	313	1.82E-04	4.92E-09	0.009
12	50160662	51582048	414	-3.12E-05	7.26E-09	0.714
12	51582188	52913730	699	2.17E-05	1.11E-08	0.837
12	52914211	54379670	715	9.29E-06	2.99E-08	0.957
12	54380016	56792337	760	-1.61E-04	1.27E-08	0.155
12	56795415	57534912	235	1.08E-04	3.64E-09	0.074
12	57535266	58748272	384	1.49E-05	5.01E-09	0.833
12	58748846	60859665	698	6.56E-06	9.66E-09	0.947
12	60859809	61738896	266	2.37E-06	3.22E-09	0.967
12	61740561	63158569	602	1.09E-05	1.11E-08	0.918
12	63158712	63908647	359	-9.07E-05	6.76E-09	0.270
12	63910665	65383507	407	-1.69E-04	1.06E-08	0.100
12	65926411	67039427	475	-5.70E-05	7.32E-09	0.505
12	67039600	67817612	376	3.97E-05	7.48E-09	0.646
12	67818111	68740414	516	-4.53E-05	1.03E-08	0.655
12	68742112	69826093	484	-8.11E-05	1.12E-08	0.443
12	69826237	70815755	524	9.34E-05	8.57E-09	0.313
12	70817279	71688642	411	-5.33E-05	8.49E-09	0.563
12	71690933	72513196	315	5.00E-05	4.92E-09	0.476
12	72525849	74846997	634	-1.73E-05	9.57E-09	0.859
12	75193041	76058346	327	1.92E-05	5.14E-09	0.789
12	76060283	77098899	452	-9.42E-06	8.09E-09	0.917

12	77099081	78398357	623	3.27E-05	1.12E-08	0.757
12	78400884	78925445	243	2.20E-05	5.41E-09	0.765
12	78926240	80944201	508	3.31E-05	7.47E-09	0.702
12	80945774	83137872	721	-7.74E-05	1.28E-08	0.494
12	83143660	83854442	269	-1.35E-05	5.54E-09	0.856
12	83856540	85014953	417	5.95E-07	5.14E-09	0.993
12	85016898	87296535	569	-3.34E-05	7.22E-09	0.694
12	87297692	87905878	166	1.05E-05	2.50E-09	0.834
12	87909726	88848006	218	4.74E-05	3.21E-09	0.402
12	88851543	91827908	896	6.88E-05	1.51E-08	0.576
12	91827930	93792073	767	-9.58E-05	1.54E-08	0.441
12	93792655	95717265	1014	-7.75E-05	2.09E-08	0.592
12	99224060	99816442	203	-2.72E-05	3.63E-09	0.651
12	95717497	97439396	837	-1.41E-04	1.61E-08	0.266
12	97439589	99220284	910	-2.33E-06	1.82E-08	0.986
12	101857796	102961329	417	-1.83E-04	6.78E-09	0.027
12	99816842	101857521	802	1.30E-04	1.51E-08	0.291
12	102961450	104011319	432	-2.39E-06	7.84E-09	0.978
12	105817813	106155478	204	2.73E-05	4.63E-09	0.689
12	104012667	105816376	1163	6.56E-05	1.91E-08	0.635
12	106156001	107521770	612	4.04E-05	9.06E-09	0.671
12	107527013	108504440	424	8.91E-05	7.34E-09	0.298
12	108506696	110111462	699	2.85E-06	1.11E-08	0.978
12	110112932	113027651	567	-9.32E-05	6.67E-09	0.254
12	113030227	113877766	334	1.62E-04	5.40E-09	0.027
12	113885696	114882476	594	1.25E-04	1.57E-08	0.319
12	114883085	115326972	185	-5.35E-05	6.00E-09	0.490
12	117087697	117644288	184	4.59E-05	3.32E-09	0.426
12	115329869	117087376	969	7.95E-05	2.13E-08	0.586
12	117644436	118888131	674	3.91E-05	1.37E-08	0.738
12	118888731	119301273	203	-4.97E-06	4.57E-09	0.941
12	119303044	120007172	394	-1.64E-04	1.33E-08	0.155
12	120008093	121720040	643	3.56E-05	9.77E-09	0.719
12	121721831	124710880	878	-3.05E-05	1.12E-08	0.774
12	124710996	125416169	363	-2.32E-05	1.67E-08	0.858
12	125417493	126461057	519	-5.35E-05	1.18E-08	0.622
12	126463562	127382559	515	-1.09E-04	9.20E-09	0.256

12	127383648	128141516	401	-7.44E-05	1.04E-08	0.466
12	128141610	128810520	445	5.05E-05	1.06E-08	0.623
12	128814615	129688509	569	5.65E-05	1.71E-08	0.666
12	129689639	130157939	346	-9.02E-05	9.21E-09	0.347
12	130158997	130859973	457	7.60E-05	1.16E-08	0.481
12	130861322	131407836	344	5.10E-05	9.16E-09	0.594
12	131408716	132012266	369	1.21E-05	7.66E-09	0.890
12	132014206	132706778	276	-4.91E-05	5.39E-09	0.504
12	132807034	133841558	234	-2.37E-04	3.42E-08	0.200
13	19020095	20682304	388	-1.33E-05	4.65E-09	0.846
13	20684769	21688499	462	1.07E-04	7.60E-09	0.220
13	21688974	22209012	217	-5.61E-05	3.87E-09	0.367
13	23590702	23827019	193	-4.48E-05	5.82E-09	0.557
13	22211354	23590611	811	9.73E-05	2.08E-08	0.501
13	23827815	24477568	384	4.06E-05	6.88E-09	0.625
13	24477971	25210959	501	-1.64E-05	1.04E-08	0.872
13	25212359	25639070	222	-9.76E-05	3.98E-09	0.122
13	25640695	27282973	736	1.30E-05	1.19E-08	0.905
13	27283115	28283059	561	5.98E-05	1.15E-08	0.577
13	28284459	28876214	284	-7.26E-05	4.61E-09	0.285
13	28879332	29872965	448	-1.82E-04	1.09E-08	0.081
13	29873521	31379027	783	1.53E-04	1.60E-08	0.226
13	31384910	32983250	791	3.22E-05	1.35E-08	0.782
13	32984197	34681078	708	-7.07E-05	9.45E-09	0.467
13	34683262	36042839	439	-1.16E-05	8.30E-09	0.898
13	36043978	37027089	548	-1.14E-05	8.95E-09	0.904
13	37029009	38290705	619	-6.65E-05	8.84E-09	0.479
13	38291011	38877678	287	2.68E-06	4.09E-09	0.967
13	38887155	40179288	575	-3.66E-06	8.04E-09	0.967
13	40181792	41773356	639	7.21E-05	1.03E-08	0.478
13	41774489	43099692	546	-4.22E-05	7.99E-09	0.637
13	43099889	44637493	786	6.11E-05	1.19E-08	0.576
13	46493234	46866562	161	5.69E-06	2.17E-09	0.903
13	44638337	46491101	753	-4.92E-05	1.22E-08	0.656
13	48440893	49987145	462	-5.34E-07	4.99E-09	0.994
13	46869621	48439005	725	4.48E-05	1.12E-08	0.672
13	49987663	51254196	497	6.29E-05	1.58E-08	0.617

13	51254712	53336663	702	-4.16E-05	9.54E-09	0.670
13	53337952	54681048	610	6.09E-05	1.30E-08	0.593
13	54681363	56433642	544	1.90E-05	6.67E-09	0.816
13	56434161	58247964	411	-2.04E-05	4.24E-09	0.755
13	60087834	60854106	241	-1.05E-04	2.69E-09	0.043
13	58248124	60085069	714	5.10E-05	1.10E-08	0.628
13	60855021	61589772	347	2.89E-05	4.83E-09	0.678
13	61591949	62912481	486	-1.23E-05	6.69E-09	0.881
13	62913542	64201284	414	-2.55E-05	6.06E-09	0.744
13	64202212	65339575	366	7.28E-06	4.32E-09	0.912
13	65340421	66197786	242	4.02E-05	2.43E-09	0.415
13	66200191	67713633	592	-1.81E-05	8.05E-09	0.840
13	67714255	69561090	643	1.03E-05	9.34E-09	0.915
13	70643712	71095217	239	-3.25E-06	4.02E-09	0.959
13	69561894	70642326	429	-2.53E-06	7.16E-09	0.976
13	71095483	71759743	313	-4.78E-05	6.49E-09	0.553
13	71763633	72958993	471	3.27E-06	7.57E-09	0.970
13	72959394	73795534	346	2.57E-04	1.68E-08	0.047
13	73796728	75084163	678	-5.89E-05	1.35E-08	0.612
13	75084473	76504672	666	2.98E-04	1.17E-08	0.006
13	78032193	78807439	318	-3.47E-05	4.29E-09	0.596
13	76505282	78030962	671	5.07E-05	1.05E-08	0.620
13	78807836	80252742	553	6.13E-05	7.10E-09	0.467
13	80257200	82580321	856	1.55E-05	1.27E-08	0.890
13	82580994	84132765	493	1.67E-05	5.16E-09	0.816
13	84133082	85035423	269	7.59E-06	3.84E-09	0.903
13	85037313	85609664	262	4.09E-06	3.59E-09	0.946
13	85609760	87039195	528	2.99E-05	9.23E-09	0.756
13	87039360	88190520	314	6.21E-05	3.45E-09	0.290
13	88191656	89057861	239	-2.55E-05	3.25E-09	0.655
13	89059635	90177618	305	3.16E-05	3.17E-09	0.575
13	90179364	90867683	318	-2.88E-05	5.10E-09	0.687
13	90869873	91967960	404	-5.08E-05	6.15E-09	0.517
13	91970313	92912119	364	-2.58E-05	5.28E-09	0.722
13	92912423	93918144	412	-5.04E-05	6.15E-09	0.521
13	93918533	94562549	322	1.06E-04	4.23E-09	0.105
13	94563601	95540024	474	4.81E-05	9.19E-09	0.616

13	97521207	98589842	365	-1.42E-05	5.53E-09	0.849
13	95540709	97520182	918	2.41E-05	1.33E-08	0.835
13	98594975	100043997	776	-1.99E-06	1.20E-08	0.986
13	100044673	101392748	562	4.67E-05	9.35E-09	0.629
13	101394726	102299385	583	3.02E-05	9.12E-09	0.752
13	102299498	103339911	558	1.40E-04	8.82E-09	0.137
13	103340536	104065375	505	2.76E-05	8.81E-09	0.769
13	104065534	104842764	367	-7.64E-06	7.07E-09	0.928
13	104843186	105990071	584	4.48E-05	1.30E-08	0.694
13	105990812	106439396	245	-5.67E-05	4.71E-09	0.409
13	106442608	107785431	712	-1.03E-04	1.48E-08	0.394
13	107788025	108518444	439	3.12E-05	9.39E-09	0.747
13	108519987	109359792	437	9.84E-05	9.24E-09	0.306
13	109359834	110338482	539	9.08E-05	1.15E-08	0.397
13	110338913	111432821	728	1.38E-04	1.68E-08	0.287
13	111434814	113266084	864	3.54E-05	1.62E-08	0.781
13	113269127	115109853	666	1.20E-05	1.02E-08	0.906
14	20544512	20877114	188	1.01E-05	4.23E-09	0.876
14	20878890	21267077	308	4.75E-05	9.99E-09	0.635
14	21271573	22378835	520	-1.51E-05	1.26E-08	0.893
14	22379132	22738105	228	1.12E-04	5.36E-09	0.126
14	22738161	23607730	506	2.22E-04	1.24E-08	0.047
14	23607866	24904154	514	1.29E-04	1.62E-08	0.309
14	24904240	26134815	652	2.77E-05	1.11E-08	0.792
14	26135167	26679549	244	-1.62E-06	5.23E-09	0.982
14	26680794	28466225	588	-1.28E-05	1.06E-08	0.901
14	28466533	29196856	255	1.03E-05	4.53E-09	0.878
14	29198059	32014712	795	-3.66E-05	1.61E-08	0.773
14	32016504	33033695	420	5.51E-05	8.95E-09	0.561
14	36345767	36682357	143	1.74E-05	2.25E-09	0.714
14	34769883	36344816	496	8.36E-06	8.45E-09	0.928
14	33036081	34769821	1119	-1.83E-04	2.97E-08	0.287
14	36683516	38481516	689	2.53E-04	1.93E-08	0.069
14	38486187	39370633	360	-2.81E-05	6.21E-09	0.722
14	39370726	40336296	361	-5.13E-05	5.88E-09	0.504
14	40336990	41615299	437	-1.61E-05	6.38E-09	0.840
14	41615331	43065019	493	1.84E-05	7.29E-09	0.830

14	43068948	44541699	471	-6.68E-05	9.83E-09	0.501
14	44542368	45247021	304	2.07E-06	5.52E-09	0.978
14	45250418	46311209	220	1.49E-05	2.51E-09	0.765
14	46312678	47456738	355	-6.19E-06	4.82E-09	0.929
14	47457846	48351664	389	2.69E-05	6.69E-09	0.742
14	48352461	49001226	256	3.29E-06	5.26E-09	0.964
14	49004194	49955015	359	5.34E-05	5.82E-09	0.484
14	49955381	52077965	933	3.31E-05	1.79E-08	0.804
14	52078316	52761693	415	1.23E-06	8.49E-09	0.989
14	52762622	54162101	504	7.09E-05	1.27E-08	0.529
14	54162655	55043520	312	-4.64E-06	6.36E-09	0.954
14	55044905	56215082	474	-1.06E-04	7.65E-09	0.227
14	56215477	56949485	467	-7.76E-05	7.95E-09	0.384
14	58449526	59135770	213	9.78E-05	3.47E-09	0.097
14	56949644	58448681	677	-9.30E-05	1.36E-08	0.425
14	59135989	61649612	943	-4.61E-06	1.36E-08	0.968
14	63166807	63890839	304	-5.63E-05	4.98E-09	0.425
14	61651278	63164649	666	4.71E-05	1.37E-08	0.688
14	63897095	65218457	418	-2.32E-05	7.96E-09	0.795
14	65220775	66353557	440	4.14E-05	6.99E-09	0.621
14	66354477	67990648	525	3.60E-05	7.30E-09	0.674
14	67991028	69355606	630	-5.04E-05	1.92E-08	0.716
14	71132759	72213258	357	3.01E-05	4.79E-09	0.664
14	69356353	71131097	786	2.51E-05	1.78E-08	0.851
14	72214790	72888979	421	-4.30E-05	7.26E-09	0.614
14	72889376	74250730	549	-9.67E-05	1.32E-08	0.399
14	74252993	75747118	538	1.41E-04	7.73E-09	0.108
14	75747258	76583135	370	-9.45E-06	7.27E-09	0.912
14	76583775	77332160	385	-3.06E-05	9.01E-09	0.748
14	77332408	78421884	573	8.57E-06	1.04E-08	0.933
14	78422260	79026816	371	3.63E-05	7.01E-09	0.665
14	80999361	81787255	327	3.33E-05	5.37E-09	0.649
14	81787481	82459297	259	-1.80E-05	5.19E-09	0.803
14	79027504	80998547	757	7.34E-05	1.37E-08	0.531
14	82461794	84087895	606	-9.52E-05	1.22E-08	0.389
14	84087979	85035235	352	2.80E-05	5.94E-09	0.716
14	85037974	86051751	438	-4.33E-05	9.28E-09	0.653

14	86053777	87334624	501	-2.13E-05	8.81E-09	0.821
14	87335256	88641174	451	-5.82E-05	7.37E-09	0.498
14	88641335	90069965	638	-3.02E-05	1.17E-08	0.780
14	90075441	92098486	749	-4.25E-06	1.60E-08	0.973
14	92099719	93378838	646	3.48E-05	1.34E-08	0.764
14	93379151	94420996	456	2.33E-05	1.32E-08	0.839
14	94421488	95347359	703	8.06E-05	1.44E-08	0.502
14	96251418	97570260	662	3.05E-07	1.44E-08	0.998
14	95347761	96250900	641	-9.27E-05	1.46E-08	0.443
14	97571437	98987472	651	-4.64E-05	1.29E-08	0.683
14	98989483	99980087	542	-2.37E-05	1.36E-08	0.839
14	99981105	101244250	481	-1.07E-05	9.02E-09	0.910
14	101244293	102339561	445	1.49E-04	1.30E-08	0.192
14	104761778	105440784	241	-6.97E-05	5.39E-09	0.342
14	105443695	106552839	139	-4.18E-05	2.66E-09	0.418
14	102341650	104759919	713	4.83E-04	1.39E-08	0.000
15	22362658	23288776	142	-3.52E-05	4.09E-09	0.582
15	23292175	24320772	294	1.19E-05	7.47E-09	0.890
15	24321414	24976269	145	-1.21E-05	3.13E-09	0.828
15	25107905	25750941	208	-1.97E-05	4.35E-09	0.765
15	25752114	26185554	309	-9.21E-05	8.37E-09	0.314
15	26186113	26675188	259	1.98E-04	6.95E-09	0.018
15	26676258	27265824	242	-2.89E-05	6.26E-09	0.715
15	27266835	27819408	253	-8.78E-05	5.74E-09	0.247
15	27820352	28325439	264	5.25E-05	5.44E-09	0.476
15	28326536	29617158	224	1.16E-04	3.95E-09	0.064
15	29617517	31314317	409	3.63E-05	7.56E-09	0.676
15	31315570	32177326	353	2.81E-04	1.57E-08	0.025
15	32180607	33251727	347	1.10E-04	8.34E-09	0.227
15	33253484	33792116	378	-3.72E-05	1.25E-08	0.739
15	33793179	34273308	356	-5.01E-05	8.82E-09	0.594
15	34273346	34970673	223	1.67E-05	4.26E-09	0.798
15	34971983	35941864	367	2.96E-05	6.32E-09	0.710
15	35943377	36424581	299	-1.70E-04	5.92E-09	0.028
15	36425448	36968033	285	-7.13E-05	7.10E-09	0.397
15	36968291	37930544	441	4.09E-05	8.96E-09	0.666
15	37931874	38804104	335	-2.07E-04	6.52E-09	0.010

15	38805185	39518563	383	3.03E-05	7.86E-09	0.732
15	39519092	40382441	407	-8.54E-06	7.90E-09	0.923
15	40383779	41211827	274	-2.16E-04	6.89E-09	0.009
15	41212265	42222063	298	3.43E-05	3.94E-09	0.585
15	42222390	43473907	377	-6.87E-05	5.05E-09	0.333
15	43474655	44988610	261	2.94E-05	2.79E-09	0.578
15	44992475	46292382	423	4.82E-05	6.21E-09	0.541
15	46294385	47000090	290	-5.39E-06	4.51E-09	0.936
15	47000857	47977097	382	-2.47E-05	5.88E-09	0.747
15	47979127	49075918	359	-9.41E-06	5.25E-09	0.897
15	49078966	50004817	260	-1.52E-05	3.36E-09	0.794
15	50006184	51681073	716	-3.70E-05	1.03E-08	0.716
15	51682775	52589995	272	4.88E-05	3.05E-09	0.378
15	52590615	53014125	168	2.37E-06	2.33E-09	0.961
15	53015101	53766542	361	-8.54E-05	8.82E-09	0.364
15	53767876	54336662	313	-5.94E-05	5.74E-09	0.433
15	54337243	55134743	391	1.13E-05	7.27E-09	0.895
15	55135408	55469163	140	4.92E-05	2.28E-09	0.303
15	55470187	55877891	151	-7.82E-08	2.84E-09	0.999
15	55879453	56784345	319	-3.63E-05	5.19E-09	0.615
15	56784506	57637678	234	6.63E-06	2.92E-09	0.902
15	57638075	58438535	510	-4.22E-05	9.63E-09	0.667
15	58439935	59505191	501	5.63E-05	1.01E-08	0.574
15	59507391	60018662	244	8.84E-06	4.43E-09	0.894
15	60019136	60670149	284	1.08E-04	5.60E-09	0.151
15	61803892	62735671	460	8.11E-05	8.05E-09	0.366
15	60670287	61803306	669	2.59E-05	1.62E-08	0.839
15	62737452	64335240	693	-3.18E-05	1.23E-08	0.775
15	65104668	66358474	354	2.61E-05	4.54E-09	0.698
15	66359486	66948204	296	-1.08E-04	9.67E-09	0.271
15	66948951	68640917	759	4.16E-05	1.17E-08	0.701
15	68642047	69936117	406	-1.29E-04	8.18E-09	0.154
15	69938058	70772135	451	1.47E-04	1.10E-08	0.161
15	70772414	71919256	463	-3.19E-05	8.25E-09	0.725
15	71920065	74447996	707	7.14E-05	9.92E-09	0.473
15	74453873	75516403	321	-1.01E-04	5.07E-09	0.158
15	75520945	76397358	160	5.54E-07	2.33E-09	0.991

15	76397390	77910873	426	-1.62E-05	5.30E-09	0.824
15	77912408	78637877	276	-1.01E-04	6.15E-09	0.199
15	78640760	79259009	192	-4.62E-05	3.34E-09	0.424
15	79261077	79991408	436	6.46E-05	7.92E-09	0.468
15	79992629	80635045	302	5.59E-05	6.71E-09	0.495
15	80637749	81604871	477	5.43E-05	1.04E-08	0.594
15	84063245	85517022	506	-4.71E-05	9.51E-09	0.629
15	81607249	84062334	626	6.60E-05	1.07E-08	0.523
15	85518062	86341812	361	-1.25E-04	4.87E-09	0.074
15	86342119	86650301	159	3.05E-05	3.12E-09	0.585
15	86650331	87113409	248	-1.53E-05	4.74E-09	0.824
15	87114392	87693557	314	-2.52E-05	7.17E-09	0.766
15	87693885	88032107	160	7.83E-06	3.15E-09	0.889
15	88032736	88742960	322	-1.57E-05	6.16E-09	0.841
15	88743108	89561223	336	7.56E-05	7.83E-09	0.393
15	89562631	90452882	361	-8.05E-05	5.97E-09	0.298
15	90453183	91221307	299	-3.72E-06	5.17E-09	0.959
15	91226586	91745246	252	-6.65E-05	6.13E-09	0.396
15	91749626	92164207	203	3.37E-05	5.14E-09	0.638
15	92165068	92667377	253	3.70E-05	6.00E-09	0.633
15	92669184	93590376	566	8.13E-05	1.22E-08	0.462
15	93591893	93908402	232	2.40E-05	8.56E-09	0.795
15	93909052	94547933	367	3.32E-06	8.47E-09	0.971
15	94548924	95576904	643	-3.16E-05	1.64E-08	0.805
15	95577096	96497729	429	-9.16E-05	1.08E-08	0.378
15	96500358	97091511	333	-1.01E-04	8.22E-09	0.265
15	97092654	98496698	628	3.09E-06	1.71E-08	0.981
15	98497881	99128454	384	-7.79E-05	1.01E-08	0.439
15	100078196	100456641	150	-1.97E-05	2.98E-09	0.718
15	99128932	100077720	485	-5.90E-05	1.11E-08	0.576
15	100457692	101403655	488	-1.63E-04	1.16E-08	0.130
15	101408616	101918220	304	1.05E-04	6.61E-09	0.195
15	101918830	102516259	285	5.06E-05	6.94E-09	0.543
16	60291	596605	208	-8.74E-05	4.57E-09	0.196
16	946205	1547999	260	-3.20E-05	5.44E-09	0.665
16	1551082	2293296	333	8.59E-05	4.79E-09	0.215
16	2294890	3960458	485	-3.00E-05	1.22E-08	0.786

16	3961770	4689471	274	5.42E-05	8.36E-09	0.553
16	4690187	5114499	203	-1.06E-04	3.58E-09	0.078
16	5115228	5854496	425	-7.21E-05	9.14E-09	0.451
16	5855169	6264138	317	6.81E-05	8.44E-09	0.459
16	6264139	7045506	647	-1.55E-05	1.39E-08	0.895
16	7046430	7524343	442	-4.20E-05	9.34E-09	0.664
16	8237157	8572350	255	5.71E-05	6.65E-09	0.484
16	8572557	8960864	188	1.28E-05	4.95E-09	0.856
16	7525825	8236887	554	3.10E-05	1.20E-08	0.777
16	8962907	9658861	334	-1.62E-04	7.47E-09	0.062
16	9659584	9997178	212	4.73E-05	3.65E-09	0.434
16	10002419	10430046	324	1.90E-05	6.14E-09	0.809
16	10431221	11493266	531	4.91E-05	1.07E-08	0.635
16	11612436	12151685	190	9.19E-06	4.02E-09	0.885
16	12775897	13119544	141	-2.45E-05	3.12E-09	0.661
16	12151797	12775407	451	-1.01E-05	7.32E-09	0.906
16	13120367	13978116	506	-2.24E-05	9.66E-09	0.820
16	13979558	15885158	355	-4.89E-05	6.04E-09	0.529
16	15885866	16220858	240	2.82E-05	3.87E-09	0.650
16	16222763	17334499	270	2.21E-05	5.90E-09	0.773
16	17335780	18062164	300	3.31E-05	6.21E-09	0.674
16	18065944	20054371	500	2.14E-06	8.79E-09	0.982
16	20055884	21235343	433	2.50E-05	7.49E-09	0.773
16	21237217	23369433	478	2.07E-05	9.13E-09	0.829
16	23371110	24308825	377	-5.98E-05	5.88E-09	0.435
16	24309108	25589623	527	-1.07E-04	9.40E-09	0.271
16	26683251	26943239	137	-4.47E-05	3.99E-09	0.479
16	25590147	26681997	620	-1.44E-04	1.44E-08	0.231
16	26944850	27445360	274	1.64E-04	7.83E-09	0.064
16	27446054	29023966	442	2.42E-05	5.37E-09	0.741
16	29694822	31380596	353	-3.93E-05	4.25E-09	0.547
16	31384554	47981754	255	8.54E-06	3.76E-09	0.889
16	47982405	49006844	259	5.63E-05	3.23E-09	0.322
16	49007408	50694011	658	-5.94E-05	1.38E-08	0.613
16	50698071	51700822	458	-4.96E-05	9.74E-09	0.615
16	51703888	53845487	830	3.08E-05	1.35E-08	0.790
16	53848448	54671719	496	-1.57E-04	1.38E-08	0.182

16	54673195	55505040	496	2.73E-05	1.07E-08	0.791
16	55506137	56906006	601	9.00E-06	9.75E-09	0.927
16	56908045	57663112	346	-3.62E-05	8.76E-09	0.699
16	57663619	58508278	391	-1.97E-04	1.49E-08	0.106
16	58510034	59888170	534	3.23E-05	8.07E-09	0.719
16	59889269	60744157	301	6.99E-06	5.93E-09	0.928
16	60745208	61991704	352	-4.56E-05	5.57E-09	0.542
16	61993462	63395786	493	-8.75E-05	9.31E-09	0.364
16	63396006	64714759	477	-3.71E-06	7.51E-09	0.966
16	64716851	65538017	376	-5.87E-05	5.43E-09	0.426
16	68429894	70885818	618	-1.55E-05	7.74E-09	0.860
16	65538484	68429077	906	-1.86E-05	1.30E-08	0.871
16	72915753	73542956	354	-4.78E-05	9.69E-09	0.627
16	70887506	72915425	539	-5.29E-05	6.76E-09	0.520
16	73543220	74724933	470	-1.43E-05	8.42E-09	0.876
16	74730819	75517115	291	-6.68E-05	5.38E-09	0.363
16	75518129	76056374	169	-2.84E-05	2.81E-09	0.592
16	76058697	76987262	437	2.91E-05	7.04E-09	0.728
16	76988130	77455781	322	-1.89E-05	6.50E-09	0.815
16	77456244	77688760	199	-1.87E-05	3.71E-09	0.759
16	77689074	78446696	583	-8.10E-05	1.31E-08	0.479
16	78452348	78709939	217	-6.80E-05	3.97E-09	0.280
16	78710328	79368760	699	-2.29E-05	1.69E-08	0.861
16	79369779	80184442	562	-1.70E-04	1.69E-08	0.191
16	80184495	81209234	656	7.19E-05	1.13E-08	0.498
16	82482458	83078581	572	7.90E-06	1.36E-08	0.946
16	81209631	82481987	784	7.03E-05	1.80E-08	0.601
16	83972415	84645588	626	-1.65E-04	1.83E-08	0.224
16	83078959	83971446	825	-1.25E-04	1.86E-08	0.358
16	84646612	85146709	319	2.03E-04	8.33E-09	0.026
16	85146935	85663943	210	3.90E-05	8.19E-09	0.666
16	85664453	86221644	410	1.01E-04	1.12E-08	0.340
16	86222417	86745679	507	6.43E-05	1.50E-08	0.599
16	86745955	87173290	253	2.51E-06	8.17E-09	0.978
16	87175053	88389287	532	-1.43E-05	1.27E-08	0.899
16	88439429	88964934	242	-6.53E-05	6.87E-09	0.431
16	88966667	89604610	217	-2.82E-05	4.14E-09	0.660

16	89605495	90292767	238	4.44E-06	4.93E-09	0.950
17	828	296442	132	-8.61E-05	1.17E-08	0.427
17	396660	820493	178	2.09E-04	1.66E-08	0.104
17	820511	1480393	258	-3.62E-05	1.32E-08	0.753
17	1481897	2613925	468	6.11E-05	1.47E-08	0.614
17	2616352	3425788	386	3.83E-05	1.42E-08	0.748
17	3426133	4321755	383	-1.84E-05	1.20E-08	0.866
17	4322053	4650487	175	3.63E-05	7.94E-09	0.684
17	4652156	5573766	370	3.40E-05	1.08E-08	0.743
17	6572170	6975193	180	5.57E-05	7.13E-09	0.509
17	5574433	6570522	609	-3.18E-06	2.22E-08	0.983
17	6976284	7723513	317	-2.12E-04	1.07E-08	0.040
17	7725660	8704551	398	3.91E-05	1.76E-08	0.768
17	8705612	9229793	253	-1.07E-04	1.99E-08	0.449
17	9233113	9714549	223	-6.21E-05	7.82E-09	0.483
17	9714995	10573151	522	1.96E-05	1.81E-08	0.884
17	10573457	11667346	523	-1.26E-04	1.80E-08	0.347
17	11668104	13000883	588	-5.72E-06	2.64E-08	0.972
17	13001700	13625425	351	-5.60E-05	1.36E-08	0.632
17	13626102	14209988	321	1.36E-05	1.16E-08	0.900
17	14210015	14499605	184	2.52E-05	6.99E-09	0.763
17	14500054	14859339	269	1.71E-05	8.95E-09	0.857
17	14860784	16319512	495	2.27E-04	1.60E-08	0.073
17	16321959	17292491	305	4.15E-05	9.82E-09	0.675
17	17299591	18446092	382	9.08E-05	1.00E-08	0.364
17	18456679	19666001	255	-5.26E-06	7.45E-09	0.951
17	20679485	21284673	159	1.82E-05	4.66E-09	0.790
17	21285166	25677370	210	2.31E-05	5.75E-09	0.761
17	25679294	26272308	203	-6.00E-05	5.64E-09	0.424
17	26273607	27884779	445	-9.95E-05	1.15E-08	0.354
17	27885675	29259899	374	3.38E-06	9.80E-09	0.973
17	29262773	30878805	457	-2.25E-06	1.80E-08	0.987
17	30879433	31989867	615	6.42E-05	1.97E-08	0.647
17	31990385	32677709	409	1.27E-04	1.25E-08	0.254
17	33616080	34393028	274	-5.74E-05	1.19E-08	0.599
17	32677947	33614452	507	-7.46E-05	1.64E-08	0.560
17	34393296	35725997	358	-4.20E-05	1.09E-08	0.687

17	35731855	37341235	493	4.85E-05	1.25E-07	0.891
17	37344586	38649203	329	-2.58E-04	1.75E-08	0.051
17	38652220	39657179	453	-2.88E-05	1.16E-08	0.790
17	39657337	40231221	206	-1.73E-06	5.49E-09	0.981
17	40232810	41769732	392	5.03E-05	1.00E-08	0.616
17	41771040	43317574	551	-9.44E-05	1.54E-08	0.447
17	44863413	45874715	302	2.57E-05	8.28E-09	0.778
17	45876022	46828302	408	-5.58E-05	1.21E-08	0.613
17	46828412	48027295	402	-1.11E-04	2.42E-08	0.478
17	48027688	48832008	360	-5.47E-05	1.40E-08	0.644
17	48832437	49892805	427	-2.55E-05	1.20E-08	0.816
17	49894599	51282380	527	3.75E-05	1.32E-08	0.744
17	52863321	53338298	227	4.49E-05	6.79E-09	0.586
17	51284441	52861905	537	-1.34E-04	1.56E-08	0.282
17	53346718	54164464	383	-2.79E-05	1.09E-08	0.789
17	54164935	55278525	429	-2.70E-05	1.37E-08	0.818
17	57487538	59307733	300	5.57E-07	7.26E-09	0.995
17	60885287	62035655	261	-5.71E-05	6.51E-09	0.479
17	59309085	60883279	464	-4.94E-05	1.35E-08	0.671
17	55279508	57486793	879	-1.73E-05	2.83E-08	0.918
17	64434524	65013985	328	-5.11E-05	1.09E-08	0.625
17	62037685	64434272	752	-9.10E-07	2.22E-08	0.995
17	65016683	66315468	381	-1.88E-04	1.28E-08	0.096
17	66318466	66903293	310	5.77E-05	9.95E-09	0.563
17	66906217	68199133	459	4.27E-05	1.34E-08	0.712
17	68199783	68875761	303	-3.60E-05	9.24E-09	0.708
17	68877746	69242135	206	1.87E-04	2.88E-08	0.271
17	69242932	70147289	397	-1.43E-04	3.35E-08	0.434
17	70148198	70947580	476	8.19E-05	1.83E-08	0.546
17	70948535	71825523	479	1.94E-04	1.73E-08	0.139
17	71829519	72740027	435	9.60E-06	1.55E-08	0.938
17	72742484	74016834	403	-4.22E-05	1.16E-08	0.695
17	74018640	74909405	367	4.49E-05	1.09E-08	0.667
17	74910943	75471247	313	-4.46E-05	1.26E-08	0.690
17	75476450	75842792	220	-3.12E-06	9.60E-09	0.975
17	75844316	76445231	219	8.00E-05	8.94E-09	0.397
17	76446689	77093768	321	1.19E-05	1.19E-08	0.913

17	77094717	77468875	305	-2.48E-05	1.37E-08	0.832
17	77471383	78780080	508	1.97E-05	1.75E-08	0.882
17	78780686	79683249	362	-7.76E-05	1.27E-08	0.492
17	79683698	80969550	415	2.56E-05	1.16E-08	0.812
18	349293	906343	394	-5.51E-06	9.19E-09	0.954
18	906673	1940581	433	-5.56E-06	8.88E-09	0.953
18	1940863	2206099	166	-3.00E-05	4.76E-09	0.664
18	2206891	2889826	314	-3.86E-05	6.74E-09	0.638
18	2891847	3357120	218	-1.48E-04	5.77E-09	0.052
18	3359237	3861997	217	2.22E-05	7.60E-09	0.799
18	3862461	4597021	431	1.17E-04	1.11E-08	0.266
18	4601573	5197395	229	-2.76E-06	5.42E-09	0.970
18	5198562	5852964	326	-2.65E-05	7.43E-09	0.759
18	5855598	6630394	359	-2.24E-05	8.28E-09	0.806
18	6630840	7373590	413	-1.25E-05	9.65E-09	0.899
18	7374066	8440357	509	-4.73E-06	9.47E-09	0.961
18	8440558	9414901	547	2.79E-04	1.28E-08	0.014
18	9415389	10462537	664	7.24E-05	1.75E-08	0.584
18	10465005	11028008	321	4.69E-05	5.64E-09	0.532
18	11028129	11529011	226	3.94E-05	5.18E-09	0.584
18	11530996	12737037	465	6.49E-05	7.86E-09	0.464
18	12737582	14358413	604	1.10E-04	1.04E-08	0.279
18	18527782	21622994	744	2.77E-05	1.42E-08	0.816
18	21623416	22745505	572	9.84E-05	1.15E-08	0.359
18	22746547	24088696	488	-5.30E-05	8.46E-09	0.565
18	24088831	24971742	490	-1.81E-05	7.26E-09	0.831
18	26874132	28037427	397	9.64E-06	5.76E-09	0.899
18	24972728	26873513	697	-2.73E-05	1.08E-08	0.793
18	28037840	28560707	235	-2.14E-05	4.31E-09	0.745
18	28561634	29563172	480	3.98E-05	8.03E-09	0.657
18	31777336	32456417	204	-1.03E-05	2.75E-09	0.845
18	29564408	31774120	674	7.20E-05	9.03E-09	0.448
18	32457437	33791804	485	-4.96E-05	7.38E-09	0.564
18	33792671	35072855	572	4.31E-05	8.55E-09	0.641
18	35073616	35837704	322	-1.73E-06	4.91E-09	0.980
18	35838871	36364691	241	4.41E-05	4.33E-09	0.502
18	36365021	37682827	411	-2.10E-05	5.92E-09	0.785

18	37683967	38766853	525	6.77E-05	7.96E-09	0.448
18	38767938	39891215	366	1.02E-05	5.41E-09	0.890
18	42606091	43295247	412	9.98E-05	7.19E-09	0.239
18	43295347	43881224	239	4.93E-05	3.20E-09	0.384
18	39892281	42606024	953	8.55E-05	1.41E-08	0.472
18	45314528	46208355	424	-2.71E-05	8.82E-09	0.773
18	43881706	45313390	680	5.40E-05	1.07E-08	0.602
18	46209339	47262925	483	5.49E-05	8.63E-09	0.555
18	47263681	47814821	330	-3.44E-05	4.95E-09	0.625
18	47816777	48784027	395	1.80E-05	5.99E-09	0.816
18	48784953	50151479	574	4.15E-05	1.06E-08	0.687
18	50152229	51059341	375	-9.27E-05	5.38E-09	0.206
18	51059850	51952586	343	-5.30E-05	5.64E-09	0.481
18	51953945	52707502	198	-5.83E-05	3.22E-09	0.304
18	52709949	53763665	408	-4.12E-05	7.24E-09	0.628
18	53764157	55089715	500	-9.48E-06	7.72E-09	0.914
18	55090919	55808073	392	1.81E-04	9.96E-09	0.069
18	55808692	56752598	490	3.36E-05	1.64E-08	0.793
18	56754050	57504565	478	1.43E-04	9.22E-09	0.136
18	57505073	58816387	484	-2.78E-05	7.66E-09	0.751
18	58817565	59585320	377	5.64E-05	8.18E-09	0.533
18	60823554	61439643	275	-9.75E-06	4.42E-09	0.883
18	59585959	60822264	549	4.57E-06	9.13E-09	0.962
18	61441801	62075853	293	-3.08E-05	4.06E-09	0.629
18	62078305	63110788	403	-3.19E-05	6.76E-09	0.698
18	63111628	64250279	396	2.21E-05	5.79E-09	0.772
18	65830606	66404071	323	-1.92E-05	5.28E-09	0.792
18	64251305	65830031	733	4.85E-05	1.37E-08	0.679
18	66404118	67569547	516	-8.20E-05	1.13E-08	0.440
18	67574191	69038112	511	-5.43E-05	1.07E-08	0.600
18	69039235	70094707	484	-3.58E-05	9.98E-09	0.720
18	70094975	70717286	329	-4.46E-05	6.58E-09	0.583
18	70719886	71243399	284	5.94E-05	6.08E-09	0.446
18	71244692	71892202	389	-7.62E-05	1.42E-08	0.523
18	71892443	72519795	346	7.66E-05	6.20E-09	0.330
18	75029584	75473982	288	1.22E-04	6.72E-09	0.137
18	72521276	73897594	733	1.30E-04	1.73E-08	0.322

18	73898846	75028501	623	2.68E-04	1.73E-08	0.041
18	75474664	75975253	233	-1.11E-05	5.37E-09	0.880
18	75976567	77151520	501	3.09E-06	1.31E-08	0.978
18	77151867	78017074	333	-1.13E-05	6.35E-09	0.887
19	157892	757412	226	2.30E-05	9.94E-09	0.817
19	759261	1089241	159	-4.15E-05	6.91E-09	0.618
19	1090803	1920712	260	1.29E-04	1.09E-08	0.217
19	1922476	2815372	338	-1.00E-05	1.24E-08	0.928
19	2816339	3301333	213	6.16E-05	8.29E-09	0.499
19	3304779	4347573	345	1.17E-04	1.46E-08	0.332
19	4349416	5191547	333	-8.25E-05	2.26E-08	0.583
19	5192372	5766090	218	3.13E-06	9.54E-09	0.974
19	5767585	6612779	262	-5.01E-06	9.21E-09	0.958
19	6614637	7309688	315	9.39E-05	1.24E-08	0.398
19	7312727	8684707	486	7.52E-05	1.85E-08	0.581
19	9013231	10029966	389	-3.77E-05	1.02E-08	0.709
19	11280183	12497411	332	-1.67E-04	9.54E-09	0.087
19	12500149	13903388	403	-2.13E-05	1.29E-08	0.851
19	10030690	11279257	438	8.24E-05	4.16E-08	0.686
19	13906229	15549939	594	-3.54E-05	1.97E-08	0.801
19	15553878	16251518	443	-1.49E-06	1.22E-08	0.989
19	17944900	18506666	241	3.13E-05	8.30E-09	0.731
19	16252072	17942927	622	-2.44E-04	2.11E-08	0.093
19	19873640	20520392	140	-4.41E-05	3.97E-09	0.484
19	18506815	19873269	429	-6.17E-05	1.16E-08	0.567
19	21379657	22036399	158	6.57E-07	4.05E-09	0.992
19	22037866	22803640	244	-3.34E-05	6.38E-09	0.676
19	22805247	24059424	282	1.11E-05	6.72E-09	0.892
19	24059507	28555771	269	3.87E-06	7.12E-09	0.963
19	28556284	28917565	189	-3.15E-05	4.95E-09	0.654
19	30113991	30858084	257	3.00E-05	9.17E-09	0.754
19	29318443	30113252	464	-3.90E-05	1.34E-08	0.736
19	30858965	32136133	462	-2.69E-04	1.36E-08	0.021
19	33764103	34067891	139	7.08E-05	4.74E-09	0.304
19	32145233	33763475	600	2.68E-05	1.73E-08	0.839
19	34068435	35331436	448	2.35E-06	1.41E-08	0.984
19	35333176	36045772	363	1.70E-07	1.31E-08	0.999

19	39371014	39757572	147	6.68E-05	5.24E-09	0.356
19	36047098	37454119	410	-1.71E-05	1.12E-08	0.872
19	41442597	41841098	164	2.43E-05	4.44E-09	0.715
19	37455512	39370456	573	-2.68E-05	2.02E-08	0.850
19	39757926	41440863	611	-1.66E-04	1.79E-08	0.216
19	41843461	43954915	363	-2.02E-04	1.83E-08	0.136
19	44882157	45255679	171	-2.73E-05	6.83E-09	0.741
19	43956095	44880657	396	6.99E-05	1.08E-08	0.501
19	45259002	46433733	397	-1.05E-04	1.33E-08	0.362
19	46433891	47333204	327	8.56E-06	1.24E-08	0.939
19	47333497	48509893	369	-1.33E-04	1.14E-08	0.214
19	48697961	49485758	336	-2.85E-04	1.08E-08	0.006
19	49491035	49923166	159	2.07E-06	4.36E-09	0.975
19	49925208	50774141	253	9.28E-05	8.22E-09	0.306
19	50774581	51331589	225	2.60E-05	8.70E-09	0.780
19	52217405	52604625	221	-2.03E-05	6.41E-09	0.800
19	51332061	52216563	531	1.32E-06	6.47E-08	0.996
19	52606936	53107086	221	-7.33E-05	8.52E-09	0.427
19	53107937	53537923	153	5.49E-06	6.59E-09	0.946
19	53538505	53899797	196	-1.62E-05	7.11E-09	0.847
19	54099626	54702450	222	-1.14E-04	1.15E-08	0.288
19	54704266	55174498	204	5.26E-05	8.83E-09	0.575
19	55175009	55791380	259	-1.08E-04	1.01E-08	0.284
19	55794266	56364684	248	8.26E-05	1.39E-08	0.483
19	56365323	56898342	290	-1.51E-05	1.20E-08	0.890
19	57952035	58531715	259	-3.93E-05	6.35E-09	0.621
19	56898853	57950377	491	4.48E-05	1.69E-08	0.731
19	58532756	59118784	238	-7.75E-05	6.72E-09	0.344
20	246392	887800	376	-1.27E-04	1.43E-08	0.288
20	888523	1445821	368	-2.29E-05	1.04E-08	0.822
20	1446580	1897442	255	-1.13E-05	5.69E-09	0.881
20	3083151	3655085	235	6.69E-06	5.56E-09	0.929
20	3655943	4051325	176	-3.53E-05	5.95E-09	0.647
20	1899473	3081680	688	2.68E-05	1.47E-08	0.825
20	4053020	4701211	402	-1.11E-05	1.19E-08	0.919
20	4701706	5304961	360	7.32E-05	8.42E-09	0.425
20	6637234	7065163	285	-9.85E-05	8.46E-09	0.284

20	5305400	6636139	709	-2.56E-04	1.65E-08	0.046
20	8601114	8881586	198	4.09E-05	3.20E-09	0.470
20	7066818	8600105	763	-6.45E-05	1.39E-08	0.584
20	8884009	9745450	471	-1.37E-05	9.33E-09	0.887
20	9746345	11013570	694	2.28E-05	1.64E-08	0.859
20	11014704	11534388	290	4.62E-05	4.96E-09	0.512
20	11534488	12153352	274	1.21E-04	5.08E-09	0.090
20	13201177	13666198	193	3.26E-06	4.00E-09	0.959
20	12153396	13199755	619	-2.56E-04	1.27E-08	0.023
20	13670311	14929217	484	-5.00E-05	8.93E-09	0.597
20	17449713	17905102	299	-1.02E-04	7.76E-09	0.246
20	15148231	16150464	704	1.17E-04	1.60E-08	0.353
20	17906490	18896415	440	-2.91E-05	7.47E-09	0.736
20	16151566	17449361	877	2.58E-05	1.78E-08	0.847
20	18896895	19707457	462	-9.31E-05	8.94E-09	0.325
20	21679930	22779512	406	2.04E-06	5.55E-09	0.978
20	19708351	21676556	817	-1.06E-05	1.56E-08	0.932
20	23568495	24053655	218	3.96E-05	3.76E-09	0.519
20	22779865	23568490	399	-2.89E-06	6.71E-09	0.972
20	24054931	25019099	416	-1.05E-05	6.64E-09	0.897
20	25020047	26319418	306	-1.84E-05	4.03E-09	0.772
20	26319535	30570889	230	-4.79E-05	3.06E-09	0.386
20	30574455	31613985	334	-7.48E-05	5.02E-09	0.291
20	33885155	34891416	221	2.24E-05	2.71E-09	0.667
20	34892880	35892530	205	-4.34E-05	3.42E-09	0.458
20	31615242	33883015	704	-1.01E-04	9.50E-09	0.298
20	35899774	36904375	395	-1.12E-04	6.26E-09	0.158
20	36905894	37875045	354	-4.07E-06	5.85E-09	0.958
20	37875309	38431408	230	1.38E-05	3.26E-09	0.809
20	38435302	39453533	470	-4.19E-05	8.57E-09	0.651
20	39455778	40584825	395	-1.03E-04	7.26E-09	0.229
20	40585126	41194048	393	-3.02E-05	8.29E-09	0.740
20	41195111	42188284	488	9.63E-06	1.08E-08	0.926
20	42188914	43257406	508	5.50E-05	9.88E-09	0.580
20	44960997	45649369	359	3.74E-05	7.30E-09	0.661
20	43259306	44960236	710	-1.47E-04	1.18E-08	0.177
20	45650139	46540102	374	6.91E-05	9.06E-09	0.468

20	48081983	48655016	227	5.09E-06	6.73E-09	0.950
20	48658113	49385779	312	2.18E-05	6.85E-09	0.793
20	46540587	48080850	769	-2.54E-05	1.39E-08	0.829
20	49387520	49945761	256	-1.72E-04	1.31E-08	0.134
20	49948016	50633472	360	1.99E-05	8.37E-09	0.828
20	50635347	51389135	319	-2.80E-05	5.53E-09	0.706
20	51389670	51884594	263	-1.93E-06	6.25E-09	0.981
20	51885269	52525657	291	2.13E-06	1.60E-08	0.987
20	52526840	53406846	483	6.19E-05	1.08E-08	0.551
20	53408035	54321635	406	3.93E-05	7.62E-09	0.653
20	54321811	55431836	547	6.98E-05	1.12E-08	0.510
20	56216001	56679942	290	3.11E-05	7.61E-09	0.722
20	55434683	56214465	498	9.28E-05	1.35E-08	0.425
20	56679960	57319010	352	-6.30E-05	7.72E-09	0.473
20	57320545	58295563	478	1.71E-05	1.28E-08	0.880
20	58296350	59361064	553	3.47E-05	1.13E-08	0.744
20	59361382	59892000	354	1.16E-05	9.36E-09	0.905
20	59892231	61091395	656	-2.30E-04	3.66E-08	0.230
20	61301855	62119318	399	-9.88E-05	1.16E-08	0.358
20	62119875	62962870	340	-4.93E-06	1.61E-08	0.969
21	15451950	16139409	423	-1.52E-04	9.07E-09	0.109
21	18457828	19151566	246	-1.40E-04	7.14E-09	0.097
21	16140244	17301757	441	7.65E-05	9.01E-09	0.420
21	17305471	18457656	407	7.06E-06	1.01E-08	0.944
21	19151940	20198061	541	-7.08E-05	1.12E-08	0.504
21	20199390	21022168	395	-3.82E-05	8.06E-09	0.671
21	21022840	21905609	384	7.23E-05	9.14E-09	0.449
21	24695869	25083563	190	-3.67E-05	4.97E-09	0.603
21	23270321	24695405	541	-8.79E-05	1.09E-08	0.400
21	25090174	25898082	385	-6.72E-05	7.31E-09	0.432
21	25899516	26465774	241	-6.45E-05	4.96E-09	0.360
21	21905658	23268216	669	-3.27E-05	1.36E-08	0.779
21	27777181	28205789	287	-1.45E-05	6.45E-09	0.857
21	26467796	27776736	496	8.17E-05	9.82E-09	0.410
21	28206883	28705495	308	3.62E-05	7.30E-09	0.672
21	28705732	29515396	352	1.26E-05	7.97E-09	0.888
21	30845873	32211778	531	-2.59E-05	8.94E-09	0.784

21	29515884	30844467	549	-8.35E-05	8.38E-09	0.362
21	32213678	33270766	453	-5.59E-05	9.76E-09	0.571
21	33273162	33988973	358	-8.64E-05	7.29E-09	0.311
21	33991368	35219895	507	-2.16E-04	2.47E-08	0.169
21	35220001	36188046	410	-2.01E-04	3.93E-08	0.311
21	37870518	38429082	265	-8.28E-05	5.16E-09	0.249
21	37305159	37868817	300	7.62E-06	5.57E-09	0.919
21	36190040	37303320	549	-5.37E-05	1.10E-08	0.609
21	38429389	39248065	402	-6.75E-05	6.60E-09	0.406
21	39250321	40115483	422	2.55E-05	9.53E-09	0.794
21	41260363	41705756	344	-4.40E-05	7.87E-09	0.620
21	40116044	41095241	608	-1.57E-04	1.14E-08	0.142
21	41707453	42543663	490	3.60E-05	1.05E-08	0.725
21	44690856	45598522	250	5.86E-05	6.71E-09	0.475
21	45603905	46174491	233	-3.00E-05	5.94E-09	0.697
21	42545250	43342046	529	-3.10E-04	2.86E-08	0.066
21	46174981	46740019	297	2.00E-05	5.87E-09	0.794
21	46741287	47491264	334	-5.52E-05	6.68E-09	0.500
21	43343190	44632513	758	1.18E-04	1.75E-08	0.373
21	47491975	48119635	293	-4.36E-05	5.07E-09	0.540
22	17187276	17569017	166	-5.42E-05	4.23E-09	0.404
22	17569439	18333467	393	1.19E-04	1.20E-08	0.279
22	18333902	19138442	274	4.83E-05	6.27E-09	0.542
22	19139368	20000428	424	-9.39E-05	1.28E-08	0.407
22	20001006	20952650	270	-2.02E-04	1.31E-08	0.077
22	20952972	21863717	294	3.68E-06	6.24E-09	0.963
22	23016570	23691564	209	2.81E-05	4.39E-09	0.671
22	21866569	23015302	361	2.43E-05	8.08E-09	0.787
22	23697301	24370194	304	4.55E-05	8.51E-09	0.622
22	25419524	26113333	224	1.57E-05	5.57E-09	0.833
22	24375019	25416292	357	1.45E-05	7.49E-09	0.867
22	26123092	26735929	447	-1.37E-04	1.17E-08	0.205
22	26736812	27294543	386	1.07E-05	1.05E-08	0.917
22	27295234	27653424	353	4.31E-05	1.20E-08	0.694
22	29456360	30604102	391	-3.74E-06	7.21E-09	0.965
22	31535872	32324140	238	-2.80E-05	3.87E-09	0.652
22	30605601	31534127	380	-1.03E-05	8.50E-09	0.911

22	27654838	29454477	730	-2.06E-04	1.94E-08	0.140
22	32324692	32856999	277	-5.54E-05	6.10E-09	0.478
22	32857285	33511391	411	2.21E-05	1.05E-08	0.829
22	33512106	33896495	177	-1.20E-05	4.97E-09	0.865
22	35865218	36131498	134	-3.23E-05	3.88E-09	0.604
22	33898114	34982457	618	-7.32E-05	1.31E-08	0.522
22	36132263	36922822	314	5.07E-05	7.52E-09	0.559
22	34983275	35862655	468	6.57E-05	8.36E-09	0.473
22	36923144	37558356	395	3.96E-05	1.06E-08	0.700
22	37560592	38467784	415	-3.78E-05	9.32E-09	0.696
22	38473398	39302783	269	1.11E-05	5.27E-09	0.878
22	39304509	40092866	319	1.09E-04	7.14E-09	0.195
22	40095174	41405753	318	4.31E-06	1.50E-08	0.972
22	41406919	42870441	427	-3.74E-06	7.90E-09	0.966
22	43187900	43645335	232	9.23E-05	3.46E-08	0.620
22	43646241	44317084	380	-7.95E-07	7.99E-09	0.993
22	44317416	44818986	414	1.09E-05	1.21E-08	0.921
22	44820796	45443845	417	-9.34E-05	1.32E-08	0.417
22	45444397	45895495	269	-1.21E-05	9.56E-09	0.902
22	45895856	46512413	260	-3.15E-05	7.54E-09	0.717
22	46513376	47106715	271	-4.96E-05	7.38E-09	0.564
22	47106800	47937655	432	-1.09E-05	9.70E-09	0.912
22	47937891	48479731	373	-7.04E-05	9.57E-09	0.472
22	49082979	49521080	312	-3.97E-05	1.05E-08	0.698
22	48481280	49081753	463	1.41E-05	1.45E-08	0.907
22	49522184	49751418	245	-4.97E-05	7.98E-09	0.578
22	49751512	50488153	334	3.20E-05	8.16E-09	0.723
22	50488995	51237713	280	6.62E-05	7.46E-09	0.443

SNP: single nucleotide polymorphisms. A Bonferroni correction was applied to account for multiple testing ($P < \sim 0.05/2353$).

Supplementary Table 3. Pleiotropic loci between COVID-19 phenotypes and prostate cancer identified from cross-trait meta-analysis ($P_{CPASSOC}$ < 5×10^{-8}, $P_{single\text{-}trait}$ < 1×10^{-4}).

Index SNP	Effect allele	Other allele	COVID-19		Prostate cancer		$P_{CPASSOC}$	Chromosome: Position	Linear closest genes[#]
			Beta	*P*-value	Beta	*P*-value			
**Prostate cancer and SARS-CoV-2 infection**									
rs12821205	C	T	0.024	1.23E-07	-0.047	6.05E-09	1.20E-11	chr12:133046199..133165564	*FBRSL1*
rs2854005	G	A	-0.025	4.43E-07	-0.040	1.97E-06	6.12E-10	chr6:31235746..31413809	*HLA-B*
**Prostate cancer and COVID-19 hospitalization**									
rs2395045	T	C	-0.047	6.31E-05	-0.048	4.43E-07	3.83E-10	chr6:31228460..31717692	*PPIAP9*
rs2493732	C	A	0.050	1.31E-05	0.043	2.98E-05	5.05E-09	chr10:81318663..81780241	*NPAP1P2,CTSLP6*
rs2507976	A	C	0.047	3.24E-06	-0.045	8.78E-08	2.66E-12	chr6:31070078..31426619	*ZDHHC20P2,HLA-S*
rs2517723	T	C	-0.062	2.38E-09	0.038	2.47E-06	1.07E-13	chr6:29606761..30056476	*HLA-A*
rs2517985	C	T	0.048	2.94E-06	-0.064	1.48E-13	1.22E-17	chr6:31003436..31426967	*CCHCR1*
rs2854005	G	A	-0.052	4.22E-07	-0.040	1.97E-06	1.14E-11	chr6:31081940..31419905	*HLA-B*
rs4475994	G	C	0.062	8.72E-11	-0.047	6.07E-09	4.89E-18	chr12:133046199..133165564	*FBRSL1*
rs61036010*	A	G	-0.043	3.42E-05	-0.039	2.38E-05	1.05E-08	chr15:31611586..31647722	*KLF13*
rs6909636	A	G	-0.055	3.97E-08	0.035	1.58E-05	1.08E-11	chr6:31330411..31359548	*ZDHHC20P2,HLA-S,FGFR3P1*
rs72879614	T	C	0.052	1.84E-05	-0.056	2.22E-07	5.34E-11	chr6:33024738..33076268	*HLA-DPA1*
rs7766862	A	G	-0.045	1.31E-05	0.045	1.25E-07	1.73E-11	chr6:31893944..32177900	*TNXB*
rs800507*	T	G	0.046	8.11E-05	0.041	2.43E-05	2.34E-08	chr8:116657235..116840770	*TRPS1*
rs9271509	A	G	-0.059	1.42E-05	0.070	3.52E-12	1.29E-15	chr6:32212655..32710377	-
rs9501117	G	A	0.103	5.19E-07	-0.075	7.79E-06	5.68E-11	chr6:31137798..31428967	-
**Prostate cancer and COVID-19 critical illness**									
rs17201588	A	T	-0.059	9.93E-05	0.045	1.16E-07	6.88E-10	chr6:31851469..32179896	*TNXB*
rs2854005	G	A	-0.063	3.03E-05	-0.040	1.97E-06	1.03E-08	chr6:31313212..31426967	*HLA-B*
rs3868087	C	A	-0.072	2.66E-05	-0.048	3.85E-07	1.37E-09	chr6:31228460..31717692	*PPIAP9*
rs407238	G	C	-0.059	3.55E-05	0.047	8.12E-09	2.85E-11	chr6:29699784..29944314	-
rs6914052*	T	C	0.122	7.52E-06	-0.069	1.41E-05	4.96E-08	chr6:32844103..33017082	-
rs7295014	A	G	0.078	2.07E-07	-0.052	9.50E-10	9.69E-14	chr12:133038610..133165564	*FBRSL1*
rs7745040	C	T	-0.077	6.76E-08	0.054	3.69E-11	1.14E-15	chr6:32386554..32804798	-
rs9268000	C	A	-0.057	8.30E-05	0.050	1.84E-09	6.35E-12	chr6:32213210..32587013	*TSBP1-AS1*
rs9501117	G	A	0.147	1.47E-06	-0.075	7.79E-06	1.21E-08	chr6:31137798..31427594	-

SNP: single nucleotide polymorphisms; VEP: Ensembl Variant Effect Predictor. [*] represents novel shared SNP; [#] based on VEP.

Supplementary Table 4. Detailed annotation of genome-wide significant SNPs identified from cross-trait meta-analysis of COVID-19 and prostate cancer.

Index SNP	Consequence	SYMBOL	Gene	Feature	BIOTYPE
rs2493732	downstream_gene_variant	NPAP1P2	ENSG00000214695	ENST00000456716.1	processed_pseudogene
rs2493732	downstream_gene_variant	CTSLP6	ENSG00000204437	ENST00000458273.2	unprocessed_pseudogene
rs7295014	intron_variant	FBRSL1	ENSG00000112787	ENST00000434748.2	protein_coding
rs7295014	intron_variant	FBRSL1	ENSG00000112787	ENST00000650108.1	protein_coding
rs7295014	intron_variant	FBRSL1	ENSG00000112787	ENST00000680143.1	protein_coding
rs7295014	regulatory_region_variant	-	-	ENSR00001195094	promoter
rs12821205	intron_variant	FBRSL1	ENSG00000112787	ENST00000434748.2	protein_coding
rs12821205	intron_variant	FBRSL1	ENSG00000112787	ENST00000434748.2	protein_coding
rs12821205	upstream_gene_variant	FBRSL1	ENSG00000112787	ENST00000539264.5	protein_coding_CDS_not_defined
rs12821205	upstream_gene_variant	FBRSL1	ENSG00000112787	ENST00000539264.5	protein_coding_CDS_not_defined
rs12821205	upstream_gene_variant	FBRSL1	ENSG00000112787	ENST00000543360.1	protein_coding_CDS_not_defined
rs12821205	upstream_gene_variant	FBRSL1	ENSG00000112787	ENST00000543360.1	protein_coding_CDS_not_defined
rs12821205	downstream_gene_variant	-	ENSG00000280287	ENST00000624087.1	TEC
rs12821205	downstream_gene_variant	-	ENSG00000280287	ENST00000624087.1	TEC
rs12821205	intron_variant	FBRSL1	ENSG00000112787	ENST00000650108.1	protein_coding
rs12821205	intron_variant	FBRSL1	ENSG00000112787	ENST00000650108.1	protein_coding
rs12821205	intron_variant	FBRSL1	ENSG00000112787	ENST00000680143.1	protein_coding
rs12821205	intron_variant	FBRSL1	ENSG00000112787	ENST00000680143.1	protein_coding
rs4475994	intron_variant	FBRSL1	ENSG00000112787	ENST00000434748.2	protein_coding
rs4475994	intron_variant	FBRSL1	ENSG00000112787	ENST00000434748.2	protein_coding
rs4475994	upstream_gene_variant	FBRSL1	ENSG00000112787	ENST00000539264.5	protein_coding_CDS_not_defined
rs4475994	upstream_gene_variant	FBRSL1	ENSG00000112787	ENST00000539264.5	protein_coding_CDS_not_defined
rs4475994	upstream_gene_variant	FBRSL1	ENSG00000112787	ENST00000543360.1	protein_coding_CDS_not_defined
rs4475994	upstream_gene_variant	FBRSL1	ENSG00000112787	ENST00000543360.1	protein_coding_CDS_not_defined
rs4475994	downstream_gene_variant	-	ENSG00000280287	ENST00000624087.1	TEC
rs4475994	downstream_gene_variant	-	ENSG00000280287	ENST00000624087.1	TEC
rs4475994	intron_variant	FBRSL1	ENSG00000112787	ENST00000650108.1	protein_coding
rs4475994	intron_variant	FBRSL1	ENSG00000112787	ENST00000650108.1	protein_coding
rs4475994	intron_variant	FBRSL1	ENSG00000112787	ENST00000680143.1	protein_coding
rs4475994	intron_variant	FBRSL1	ENSG00000112787	ENST00000680143.1	protein_coding
rs61036010	intron_variant	KLF13	ENSG00000169926	ENST00000307145.4	protein_coding
rs61036010	intron_variant	KLF13	ENSG00000169926	ENST00000307145.4	protein_coding
rs61036010	intron_variant,non_coding_transcript_variant	KLF13	ENSG00000169926	ENST00000558921.1	protein_coding_CDS_not_defined
rs61036010	intron_variant,non_coding_transcript_variant	KLF13	ENSG00000169926	ENST00000558921.1	protein_coding_CDS_not_defined
rs61036010	regulatory_region_variant	-	-	ENSR00000074488	promoter

rs61036010	regulatory_region_variant	-	-	ENSR00000074488	promoter
rs407238	intergenic_variant	-	-	-	-
rs407238	intergenic_variant	-	-	-	-
rs2517723	downstream_gene_variant	HLA-A	ENSG00000206503	ENST00000376802.2	protein_coding
rs2517723	downstream_gene_variant	HLA-A	ENSG00000206503	ENST00000376802.2	protein_coding
rs2517723	downstream_gene_variant	HLA-A	ENSG00000206503	ENST00000376802.2	protein_coding
rs2517723	downstream_gene_variant	HLA-A	ENSG00000206503	ENST00000376806.9	protein_coding
rs2517723	downstream_gene_variant	HLA-A	ENSG00000206503	ENST00000376806.9	protein_coding
rs2517723	downstream_gene_variant	HLA-A	ENSG00000206503	ENST00000376806.9	protein_coding
rs2517723	downstream_gene_variant	HLA-A	ENSG00000206503	ENST00000376809.10	protein_coding
rs2517723	downstream_gene_variant	HLA-A	ENSG00000206503	ENST00000376809.10	protein_coding
rs2517723	downstream_gene_variant	HLA-A	ENSG00000206503	ENST00000376809.10	protein_coding
rs2517723	downstream_gene_variant	HLA-A	ENSG00000206503	ENST00000396634.5	protein_coding
rs2517723	downstream_gene_variant	HLA-A	ENSG00000206503	ENST00000396634.5	protein_coding
rs2517723	downstream_gene_variant	HLA-A	ENSG00000206503	ENST00000396634.5	protein_coding
rs2517723	upstream_gene_variant	-	ENSG00000227766	ENST00000429656.1	unprocessed_pseudogene
rs2517723	upstream_gene_variant	-	ENSG00000227766	ENST00000429656.1	unprocessed_pseudogene
rs2517723	upstream_gene_variant	-	ENSG00000227766	ENST00000429656.1	unprocessed_pseudogene
rs2517723	downstream_gene_variant	HLA-A	ENSG00000206503	ENST00000461903.1	retained_intron
rs2517723	downstream_gene_variant	HLA-A	ENSG00000206503	ENST00000461903.1	retained_intron
rs2517723	downstream_gene_variant	HLA-A	ENSG00000206503	ENST00000461903.1	retained_intron
rs2517723	downstream_gene_variant	HLA-A	ENSG00000206503	ENST00000479320.5	retained_intron
rs2517723	downstream_gene_variant	HLA-A	ENSG00000206503	ENST00000479320.5	retained_intron
rs2517723	downstream_gene_variant	HLA-A	ENSG00000206503	ENST00000479320.5	retained_intron
rs2517723	downstream_gene_variant	HLA-A	ENSG00000206503	ENST00000495183.5	retained_intron
rs2517723	downstream_gene_variant	HLA-A	ENSG00000206503	ENST00000495183.5	retained_intron
rs2517723	downstream_gene_variant	HLA-A	ENSG00000206503	ENST00000495183.5	retained_intron
rs2517723	downstream_gene_variant	HLA-A	ENSG00000206503	ENST00000496081.5	retained_intron
rs2517723	downstream_gene_variant	HLA-A	ENSG00000206503	ENST00000496081.5	retained_intron
rs2517723	downstream_gene_variant	HLA-A	ENSG00000206503	ENST00000496081.5	retained_intron
rs2517723	downstream_gene_variant	HLA-A	ENSG00000206503	ENST00000638375.1	protein_coding
rs2517723	downstream_gene_variant	HLA-A	ENSG00000206503	ENST00000638375.1	protein_coding
rs2517723	downstream_gene_variant	HLA-A	ENSG00000206503	ENST00000638375.1	protein_coding
rs2517985	intron_variant	CCHCR1	ENSG00000204536	ENST00000376266.9	protein_coding
rs2517985	intron_variant	CCHCR1	ENSG00000204536	ENST00000376266.9	protein_coding
rs2517985	intron_variant	CCHCR1	ENSG00000204536	ENST00000376266.9	protein_coding
rs2517985	intron_variant	CCHCR1	ENSG00000204536	ENST00000396263.6	protein_coding

rs2517985	intron_variant	CCHCR1	ENSG00000204536	ENST00000396263.6	protein_coding
rs2517985	intron_variant	CCHCR1	ENSG00000204536	ENST00000396263.6	protein_coding
rs2517985	intron_variant	CCHCR1	ENSG00000204536	ENST00000396268.8	protein_coding
rs2517985	intron_variant	CCHCR1	ENSG00000204536	ENST00000396268.8	protein_coding
rs2517985	intron_variant	CCHCR1	ENSG00000204536	ENST00000396268.8	protein_coding
rs2517985	downstream_gene_variant	CCHCR1	ENSG00000204536	ENST00000426967.5	protein_coding
rs2517985	downstream_gene_variant	CCHCR1	ENSG00000204536	ENST00000426967.5	protein_coding
rs2517985	downstream_gene_variant	CCHCR1	ENSG00000204536	ENST00000426967.5	protein_coding
rs2517985	downstream_gene_variant	CCHCR1	ENSG00000204536	ENST00000428174.1	protein_coding
rs2517985	downstream_gene_variant	CCHCR1	ENSG00000204536	ENST00000428174.1	protein_coding
rs2517985	downstream_gene_variant	CCHCR1	ENSG00000204536	ENST00000428174.1	protein_coding
rs2517985	intron_variant	CCHCR1	ENSG00000204536	ENST00000448141.6	protein_coding
rs2517985	intron_variant	CCHCR1	ENSG00000204536	ENST00000448141.6	protein_coding
rs2517985	intron_variant	CCHCR1	ENSG00000204536	ENST00000448141.6	protein_coding
rs2517985	intron_variant	CCHCR1	ENSG00000204536	ENST00000448162.6	protein_coding
rs2517985	intron_variant	CCHCR1	ENSG00000204536	ENST00000448162.6	protein_coding
rs2517985	intron_variant	CCHCR1	ENSG00000204536	ENST00000448162.6	protein_coding
rs2517985	intron_variant	CCHCR1	ENSG00000204536	ENST00000451521.6	protein_coding
rs2517985	intron_variant	CCHCR1	ENSG00000204536	ENST00000451521.6	protein_coding
rs2517985	intron_variant	CCHCR1	ENSG00000204536	ENST00000451521.6	protein_coding
rs2517985	downstream_gene_variant	CCHCR1	ENSG00000204536	ENST00000455279.6	protein_coding
rs2517985	downstream_gene_variant	CCHCR1	ENSG00000204536	ENST00000455279.6	protein_coding
rs2517985	downstream_gene_variant	CCHCR1	ENSG00000204536	ENST00000455279.6	protein_coding
rs2517985	intron_variant,non_coding_transcript_variant	CCHCR1	ENSG00000204536	ENST00000464012.1	retained_intron
rs2517985	intron_variant,non_coding_transcript_variant	CCHCR1	ENSG00000204536	ENST00000464012.1	retained_intron
rs2517985	intron_variant,non_coding_transcript_variant	CCHCR1	ENSG00000204536	ENST00000464012.1	retained_intron
rs2517985	intron_variant,non_coding_transcript_variant	CCHCR1	ENSG00000204536	ENST00000480060.5	protein_coding_CDS_not_defined
rs2517985	intron_variant,non_coding_transcript_variant	CCHCR1	ENSG00000204536	ENST00000480060.5	protein_coding_CDS_not_defined
rs2517985	intron_variant,non_coding_transcript_variant	CCHCR1	ENSG00000204536	ENST00000480060.5	protein_coding_CDS_not_defined
rs2517985	downstream_gene_variant	CCHCR1	ENSG00000204536	ENST00000502557.5	protein_coding
rs2517985	downstream_gene_variant	CCHCR1	ENSG00000204536	ENST00000502557.5	protein_coding
rs2517985	downstream_gene_variant	CCHCR1	ENSG00000204536	ENST00000502557.5	protein_coding
rs2517985	downstream_gene_variant	CCHCR1	ENSG00000204536	ENST00000503420.5	protein_coding
rs2517985	downstream_gene_variant	CCHCR1	ENSG00000204536	ENST00000503420.5	protein_coding
rs2517985	downstream_gene_variant	CCHCR1	ENSG00000204536	ENST00000503420.5	protein_coding
rs2517985	downstream_gene_variant	CCHCR1	ENSG00000204536	ENST00000503934.5	protein_coding
rs2517985	downstream_gene_variant	CCHCR1	ENSG00000204536	ENST00000503934.5	protein_coding

rs2517985	downstream_gene_variant	CCHCR1	ENSG00000204536	ENST00000503934.5	protein_coding
rs2517985	intron_variant,non_coding_transcript_variant	CCHCR1	ENSG00000204536	ENST00000505392.5	retained_intron
rs2517985	intron_variant,non_coding_transcript_variant	CCHCR1	ENSG00000204536	ENST00000505392.5	retained_intron
rs2517985	intron_variant,non_coding_transcript_variant	CCHCR1	ENSG00000204536	ENST00000505392.5	retained_intron
rs2517985	downstream_gene_variant	CCHCR1	ENSG00000204536	ENST00000507226.1	protein_coding
rs2517985	downstream_gene_variant	CCHCR1	ENSG00000204536	ENST00000507226.1	protein_coding
rs2517985	downstream_gene_variant	CCHCR1	ENSG00000204536	ENST00000507226.1	protein_coding
rs2517985	intron_variant,NMD_transcript_variant	CCHCR1	ENSG00000204536	ENST00000507459.5	nonsense_mediated_decay
rs2517985	intron_variant,NMD_transcript_variant	CCHCR1	ENSG00000204536	ENST00000507459.5	nonsense_mediated_decay
rs2517985	intron_variant,NMD_transcript_variant	CCHCR1	ENSG00000204536	ENST00000507459.5	nonsense_mediated_decay
rs2517985	downstream_gene_variant	CCHCR1	ENSG00000204536	ENST00000507751.5	protein_coding
rs2517985	downstream_gene_variant	CCHCR1	ENSG00000204536	ENST00000507751.5	protein_coding
rs2517985	downstream_gene_variant	CCHCR1	ENSG00000204536	ENST00000507751.5	protein_coding
rs2517985	downstream_gene_variant	CCHCR1	ENSG00000204536	ENST00000507829.5	protein_coding
rs2517985	downstream_gene_variant	CCHCR1	ENSG00000204536	ENST00000507829.5	protein_coding
rs2517985	downstream_gene_variant	CCHCR1	ENSG00000204536	ENST00000507829.5	protein_coding
rs2517985	intron_variant	CCHCR1	ENSG00000204536	ENST00000508683.5	protein_coding
rs2517985	intron_variant	CCHCR1	ENSG00000204536	ENST00000508683.5	protein_coding
rs2517985	intron_variant	CCHCR1	ENSG00000204536	ENST00000508683.5	protein_coding
rs2517985	intron_variant,NMD_transcript_variant	CCHCR1	ENSG00000204536	ENST00000508852.5	nonsense_mediated_decay
rs2517985	intron_variant,NMD_transcript_variant	CCHCR1	ENSG00000204536	ENST00000508852.5	nonsense_mediated_decay
rs2517985	intron_variant,NMD_transcript_variant	CCHCR1	ENSG00000204536	ENST00000508852.5	nonsense_mediated_decay
rs2517985	intron_variant,non_coding_transcript_variant	CCHCR1	ENSG00000204536	ENST00000509552.5	retained_intron
rs2517985	intron_variant,non_coding_transcript_variant	CCHCR1	ENSG00000204536	ENST00000509552.5	retained_intron
rs2517985	intron_variant,non_coding_transcript_variant	CCHCR1	ENSG00000204536	ENST00000509552.5	retained_intron
rs2517985	intron_variant,NMD_transcript_variant	CCHCR1	ENSG00000204536	ENST00000512418.5	nonsense_mediated_decay
rs2517985	intron_variant,NMD_transcript_variant	CCHCR1	ENSG00000204536	ENST00000512418.5	nonsense_mediated_decay
rs2517985	intron_variant,NMD_transcript_variant	CCHCR1	ENSG00000204536	ENST00000512418.5	nonsense_mediated_decay
rs2517985	downstream_gene_variant	CCHCR1	ENSG00000204536	ENST00000513222.1	protein_coding
rs2517985	downstream_gene_variant	CCHCR1	ENSG00000204536	ENST00000513222.1	protein_coding
rs2517985	downstream_gene_variant	CCHCR1	ENSG00000204536	ENST00000513222.1	protein_coding
rs2517985	intron_variant,NMD_transcript_variant	CCHCR1	ENSG00000204536	ENST00000652427.1	nonsense_mediated_decay
rs2517985	intron_variant,NMD_transcript_variant	CCHCR1	ENSG00000204536	ENST00000652427.1	nonsense_mediated_decay
rs2517985	intron_variant,NMD_transcript_variant	CCHCR1	ENSG00000204536	ENST00000652427.1	nonsense_mediated_decay
rs2517985	intron_variant,NMD_transcript_variant	CCHCR1	ENSG00000204536	ENST00000652535.1	nonsense_mediated_decay
rs2517985	intron_variant,NMD_transcript_variant	CCHCR1	ENSG00000204536	ENST00000652535.1	nonsense_mediated_decay
rs2517985	intron_variant,NMD_transcript_variant	CCHCR1	ENSG00000204536	ENST00000652535.1	nonsense_mediated_decay

rs2854005	intergenic_variant	-	-	-	-
rs2854005	intergenic_variant	-	-	-	-
rs6909636	upstream_gene_variant	ZDHHC20P2	ENSG00000223702	ENST00000424108.1	processed_pseudogene
rs6909636	downstream_gene_variant	HLA-S	ENSG00000225851	ENST00000425174.1	unprocessed_pseudogene
rs6909636	downstream_gene_variant	FGFR3P1	ENSG00000230994	ENST00000449999.1	processed_pseudogene
rs6909636	downstream_gene_variant	-	ENSG00000285647	ENST00000649421.1	lncRNA
rs2507976	downstream_gene_variant	ZDHHC20P2	ENSG00000223702	ENST00000424108.1	processed_pseudogene
rs2507976	downstream_gene_variant	ZDHHC20P2	ENSG00000223702	ENST00000424108.1	processed_pseudogene
rs2507976	upstream_gene_variant	HLA-S	ENSG00000225851	ENST00000425174.1	unprocessed_pseudogene
rs2507976	upstream_gene_variant	HLA-S	ENSG00000225851	ENST00000425174.1	unprocessed_pseudogene
rs9501117	intergenic_variant	-	-	-	-
rs2395045	downstream_gene_variant	PPIAP9	ENSG00000219797	ENST00000403866.2	processed_pseudogene
rs2395045	downstream_gene_variant	PPIAP9	ENSG00000219797	ENST00000403866.2	processed_pseudogene
rs2395045	downstream_gene_variant	PPIAP9	ENSG00000219797	ENST00000403866.2	processed_pseudogene
rs2395045	downstream_gene_variant	-	ENSG00000256851	ENST00000538358.1	unprocessed_pseudogene
rs2395045	downstream_gene_variant	-	ENSG00000256851	ENST00000538358.1	unprocessed_pseudogene
rs2395045	downstream_gene_variant	-	ENSG00000256851	ENST00000538358.1	unprocessed_pseudogene
rs3868087	downstream_gene_variant	PPIAP9	ENSG00000219797	ENST00000403866.2	processed_pseudogene
rs3868087	downstream_gene_variant	PPIAP9	ENSG00000219797	ENST00000403866.2	processed_pseudogene
rs3868087	downstream_gene_variant	-	ENSG00000256851	ENST00000538358.1	unprocessed_pseudogene
rs3868087	downstream_gene_variant	-	ENSG00000256851	ENST00000538358.1	unprocessed_pseudogene
rs7766862	intron_variant	TNXB	ENSG00000168477	ENST00000375244.7	protein_coding
rs7766862	intron_variant	TNXB	ENSG00000168477	ENST00000375244.7	protein_coding
rs7766862	upstream_gene_variant	TNXB	ENSG00000168477	ENST00000611016.2	protein_coding
rs7766862	upstream_gene_variant	TNXB	ENSG00000168477	ENST00000611016.2	protein_coding
rs7766862	intron_variant	TNXB	ENSG00000168477	ENST00000613214.4	protein_coding
rs7766862	intron_variant	TNXB	ENSG00000168477	ENST00000613214.4	protein_coding
rs7766862	intron_variant	TNXB	ENSG00000168477	ENST00000644971.2	protein_coding
rs7766862	intron_variant	TNXB	ENSG00000168477	ENST00000644971.2	protein_coding
rs7766862	intron_variant	TNXB	ENSG00000168477	ENST00000647633.1	protein_coding
rs7766862	intron_variant	TNXB	ENSG00000168477	ENST00000647633.1	protein_coding
rs17201588	intron_variant	TNXB	ENSG00000168477	ENST00000375244.7	protein_coding
rs17201588	downstream_gene_variant	TNXB	ENSG00000168477	ENST00000479795.1	protein_coding
rs17201588	intron_variant	TNXB	ENSG00000168477	ENST00000613214.4	protein_coding
rs17201588	intron_variant	TNXB	ENSG00000168477	ENST00000644971.2	protein_coding
rs17201588	intron_variant	TNXB	ENSG00000168477	ENST00000647633.1	protein_coding
rs9268000	non_coding_transcript_exon_variant	TSBP1-AS1	ENSG00000225914	ENST00000425033.1	lncRNA

rs9268000	intron_variant,non_coding_transcript_variant	TSBP1-AS1	ENSG00000225914	ENST00000611838.1	lncRNA
rs9268000	intron_variant,non_coding_transcript_variant	TSBP1-AS1	ENSG00000225914	ENST00000644884.2	lncRNA
rs9268000	intron_variant,non_coding_transcript_variant	TSBP1-AS1	ENSG00000225914	ENST00000645134.1	lncRNA
rs9268000	intron_variant,non_coding_transcript_variant	TSBP1-AS1	ENSG00000225914	ENST00000645167.1	lncRNA
rs9268000	intron_variant,non_coding_transcript_variant	TSBP1-AS1	ENSG00000225914	ENST00000645563.1	lncRNA
rs9268000	intron_variant,non_coding_transcript_variant	TSBP1-AS1	ENSG00000225914	ENST00000646176.1	lncRNA
rs9268000	non_coding_transcript_exon_variant	TSBP1-AS1	ENSG00000225914	ENST00000646550.1	lncRNA
rs9268000	intron_variant,non_coding_transcript_variant	TSBP1-AS1	ENSG00000225914	ENST00000646628.1	lncRNA
rs9268000	intron_variant,non_coding_transcript_variant	TSBP1-AS1	ENSG00000225914	ENST00000647036.1	lncRNA
rs9268000	intron_variant,non_coding_transcript_variant	TSBP1-AS1	ENSG00000225914	ENST00000653107.2	lncRNA
rs9268000	intron_variant,non_coding_transcript_variant	TSBP1-AS1	ENSG00000225914	ENST00000653523.2	lncRNA
rs9268000	intron_variant,non_coding_transcript_variant	TSBP1-AS1	ENSG00000225914	ENST00000653922.1	lncRNA
rs9268000	intron_variant,non_coding_transcript_variant	TSBP1-AS1	ENSG00000225914	ENST00000685706.1	lncRNA
rs9268000	intron_variant,non_coding_transcript_variant	TSBP1-AS1	ENSG00000225914	ENST00000690046.1	lncRNA
rs9268000	intron_variant,non_coding_transcript_variant	TSBP1-AS1	ENSG00000225914	ENST00000700993.1	lncRNA
rs9268000	regulatory_region_variant	-	-	ENSR00000195672	promoter
rs9268000	TF_binding_site_variant	-	-	ENSM00522754173	-
rs9268000	TF_binding_site_variant	-	-	ENSM00524181127	-
rs9271509	intergenic_variant	-	-	-	-
rs7745040	intergenic_variant	-	-	-	-
rs7745040	intergenic_variant	-	-	-	-
rs6914052	intergenic_variant	-	-	-	-
rs72879614	downstream_gene_variant	HLA-DPA1	ENSG00000231389	ENST00000417724.1	protein_coding
rs72879614	downstream_gene_variant	HLA-DPA1	ENSG00000231389	ENST00000417724.1	protein_coding
rs72879614	downstream_gene_variant	HLA-DPA1	ENSG00000231389	ENST00000417724.1	protein_coding
rs72879614	intron_variant	HLA-DPA1	ENSG00000231389	ENST00000419277.5	protein_coding
rs72879614	intron_variant	HLA-DPA1	ENSG00000231389	ENST00000419277.5	protein_coding
rs72879614	intron_variant	HLA-DPA1	ENSG00000231389	ENST00000419277.5	protein_coding
rs72879614	upstream_gene_variant	HLA-DPA1	ENSG00000231389	ENST00000437811.1	protein_coding
rs72879614	upstream_gene_variant	HLA-DPA1	ENSG00000231389	ENST00000437811.1	protein_coding
rs72879614	upstream_gene_variant	HLA-DPA1	ENSG00000231389	ENST00000437811.1	protein_coding
rs72879614	intron_variant	HLA-DPA1	ENSG00000231389	ENST00000453337.1	protein_coding
rs72879614	intron_variant	HLA-DPA1	ENSG00000231389	ENST00000453337.1	protein_coding
rs72879614	intron_variant	HLA-DPA1	ENSG00000231389	ENST00000453337.1	protein_coding
rs72879614	intron_variant,non_coding_transcript_variant	HLA-DPA1	ENSG00000231389	ENST00000463066.1	protein_coding_CDS_not_defined
rs72879614	intron_variant,non_coding_transcript_variant	HLA-DPA1	ENSG00000231389	ENST00000463066.1	protein_coding_CDS_not_defined
rs72879614	intron_variant,non_coding_transcript_variant	HLA-DPA1	ENSG00000231389	ENST00000463066.1	protein_coding_CDS_not_defined

rs72879614	intron_variant,non_coding_transcript_variant	HLA-DPA1	ENSG00000231389	ENST00000476642.5	protein_coding_CDS_not_defined
rs72879614	intron_variant,non_coding_transcript_variant	HLA-DPA1	ENSG00000231389	ENST00000476642.5	protein_coding_CDS_not_defined
rs72879614	intron_variant,non_coding_transcript_variant	HLA-DPA1	ENSG00000231389	ENST00000476642.5	protein_coding_CDS_not_defined
rs72879614	intron_variant,non_coding_transcript_variant	HLA-DPA1	ENSG00000231389	ENST00000479107.1	retained_intron
rs72879614	intron_variant,non_coding_transcript_variant	HLA-DPA1	ENSG00000231389	ENST00000479107.1	retained_intron
rs72879614	intron_variant,non_coding_transcript_variant	HLA-DPA1	ENSG00000231389	ENST00000479107.1	retained_intron
rs72879614	intron_variant	HLA-DPA1	ENSG00000231389	ENST00000692443.1	protein_coding
rs72879614	intron_variant	HLA-DPA1	ENSG00000231389	ENST00000692443.1	protein_coding
rs72879614	intron_variant	HLA-DPA1	ENSG00000231389	ENST00000692443.1	protein_coding
rs800507	intron_variant	TRPS1	ENSG00000104447	ENST00000422939.1	protein_coding
rs800507	intron_variant	TRPS1	ENSG00000104447	ENST00000422939.1	protein_coding
rs61036010	intron_variant	KLF13	ENSG00000275746	ENST00000616962.1	protein_coding
rs61036010	intron_variant	KLF13	ENSG00000275746	ENST00000616962.1	protein_coding
rs61036010	intron_variant	KLF13	ENSG00000275746	ENST00000621843.1	protein_coding
rs61036010	intron_variant	KLF13	ENSG00000275746	ENST00000621843.1	protein_coding
rs407238	intergenic_variant	-	-	-	-
rs407238	intergenic_variant	-	-	-	-
rs2517723	downstream_gene_variant	HLA-A	ENSG00000229215	ENST00000416096.5	protein_coding
rs2517723	downstream_gene_variant	HLA-A	ENSG00000229215	ENST00000416096.5	protein_coding
rs2517723	downstream_gene_variant	HLA-A	ENSG00000229215	ENST00000416096.5	protein_coding
rs2517723	upstream_gene_variant	-	ENSG00000234831	ENST00000417944.1	unprocessed_pseudogene
rs2517723	upstream_gene_variant	-	ENSG00000234831	ENST00000417944.1	unprocessed_pseudogene
rs2517723	upstream_gene_variant	-	ENSG00000234831	ENST00000417944.1	unprocessed_pseudogene
rs2517723	downstream_gene_variant	HLA-A	ENSG00000229215	ENST00000431930.5	protein_coding
rs2517723	downstream_gene_variant	HLA-A	ENSG00000229215	ENST00000431930.5	protein_coding
rs2517723	downstream_gene_variant	HLA-A	ENSG00000229215	ENST00000431930.5	protein_coding
rs2517723	downstream_gene_variant	HLA-A	ENSG00000229215	ENST00000444289.6	protein_coding
rs2517723	downstream_gene_variant	HLA-A	ENSG00000229215	ENST00000444289.6	protein_coding
rs2517723	downstream_gene_variant	HLA-A	ENSG00000229215	ENST00000444289.6	protein_coding
rs2517723	downstream_gene_variant	HLA-A	ENSG00000229215	ENST00000464610.5	retained_intron
rs2517723	downstream_gene_variant	HLA-A	ENSG00000229215	ENST00000464610.5	retained_intron
rs2517723	downstream_gene_variant	HLA-A	ENSG00000229215	ENST00000464610.5	retained_intron
rs2517723	downstream_gene_variant	HLA-A	ENSG00000229215	ENST00000465498.1	retained_intron
rs2517723	downstream_gene_variant	HLA-A	ENSG00000229215	ENST00000465498.1	retained_intron
rs2517723	downstream_gene_variant	HLA-A	ENSG00000229215	ENST00000465498.1	retained_intron
rs2517723	downstream_gene_variant	HLA-A	ENSG00000229215	ENST00000468995.5	retained_intron
rs2517723	downstream_gene_variant	HLA-A	ENSG00000229215	ENST00000468995.5	retained_intron

rs2517723	downstream_gene_variant	HLA-A	ENSG00000229215	ENST00000468995.5	retained_intron
rs2517723	downstream_gene_variant	HLA-A	ENSG00000229215	ENST00000485166.5	retained_intron
rs2517723	downstream_gene_variant	HLA-A	ENSG00000229215	ENST00000485166.5	retained_intron
rs2517723	downstream_gene_variant	HLA-A	ENSG00000229215	ENST00000485166.5	retained_intron
rs2517723	3_prime_UTR_variant	HLA-A	ENSG00000229215	ENST00000551120.4	protein_coding
rs2517723	3_prime_UTR_variant	HLA-A	ENSG00000229215	ENST00000551120.4	protein_coding
rs2517723	3_prime_UTR_variant	HLA-A	ENSG00000229215	ENST00000551120.4	protein_coding
rs2517723	downstream_gene_variant	HLA-A	ENSG00000229215	ENST00000552193.3	protein_coding
rs2517723	downstream_gene_variant	HLA-A	ENSG00000229215	ENST00000552193.3	protein_coding
rs2517723	downstream_gene_variant	HLA-A	ENSG00000229215	ENST00000552193.3	protein_coding
rs2517723	intron_variant	HLA-A	ENSG00000229215	ENST00000640267.1	protein_coding
rs2517723	intron_variant	HLA-A	ENSG00000229215	ENST00000640267.1	protein_coding
rs2517723	intron_variant	HLA-A	ENSG00000229215	ENST00000640267.1	protein_coding
rs2517723	intron_variant	HLA-A	ENSG00000229215	ENST00000640829.1	protein_coding
rs2517723	intron_variant	HLA-A	ENSG00000229215	ENST00000640829.1	protein_coding
rs2517723	intron_variant	HLA-A	ENSG00000229215	ENST00000640829.1	protein_coding
rs2395045	downstream_gene_variant	PPIAP9	ENSG00000230616	ENST00000440658.1	processed_pseudogene
rs2395045	downstream_gene_variant	PPIAP9	ENSG00000230616	ENST00000440658.1	processed_pseudogene
rs2395045	downstream_gene_variant	PPIAP9	ENSG00000230616	ENST00000440658.1	processed_pseudogene
rs3868087	downstream_gene_variant	PPIAP9	ENSG00000230616	ENST00000440658.1	processed_pseudogene
rs3868087	downstream_gene_variant	PPIAP9	ENSG00000230616	ENST00000440658.1	processed_pseudogene
rs6914052	intergenic_variant	-	-	-	-
rs72879614	downstream_gene_variant	HLA-DPA1	ENSG00000235844	ENST00000413109.1	protein_coding
rs72879614	downstream_gene_variant	HLA-DPA1	ENSG00000235844	ENST00000413109.1	protein_coding
rs72879614	downstream_gene_variant	HLA-DPA1	ENSG00000235844	ENST00000413109.1	protein_coding
rs72879614	upstream_gene_variant	HLA-DPA1	ENSG00000235844	ENST00000419452.1	protein_coding
rs72879614	upstream_gene_variant	HLA-DPA1	ENSG00000235844	ENST00000419452.1	protein_coding
rs72879614	upstream_gene_variant	HLA-DPA1	ENSG00000235844	ENST00000419452.1	protein_coding
rs72879614	intron_variant	HLA-DPA1	ENSG00000235844	ENST00000439571.1	protein_coding
rs72879614	intron_variant	HLA-DPA1	ENSG00000235844	ENST00000439571.1	protein_coding
rs72879614	intron_variant	HLA-DPA1	ENSG00000235844	ENST00000439571.1	protein_coding
rs72879614	intron_variant	HLA-DPA1	ENSG00000235844	ENST00000443117.5	protein_coding
rs72879614	intron_variant	HLA-DPA1	ENSG00000235844	ENST00000443117.5	protein_coding
rs72879614	intron_variant	HLA-DPA1	ENSG00000235844	ENST00000443117.5	protein_coding
rs72879614	intron_variant	HLA-DPA1	ENSG00000235844	ENST00000447657.1	protein_coding
rs72879614	intron_variant	HLA-DPA1	ENSG00000235844	ENST00000447657.1	protein_coding
rs72879614	intron_variant	HLA-DPA1	ENSG00000235844	ENST00000447657.1	protein_coding

rs72879614	intron_variant,non_coding_transcript_variant	HLA-DPA1	ENSG00000235844	ENST00000463243.5	protein_coding_CDS_not_defined
rs72879614	intron_variant,non_coding_transcript_variant	HLA-DPA1	ENSG00000235844	ENST00000463243.5	protein_coding_CDS_not_defined
rs72879614	intron_variant,non_coding_transcript_variant	HLA-DPA1	ENSG00000235844	ENST00000463243.5	protein_coding_CDS_not_defined
rs72879614	intron_variant,non_coding_transcript_variant	HLA-DPA1	ENSG00000235844	ENST00000480521.1	protein_coding_CDS_not_defined
rs72879614	intron_variant,non_coding_transcript_variant	HLA-DPA1	ENSG00000235844	ENST00000480521.1	protein_coding_CDS_not_defined
rs72879614	intron_variant,non_coding_transcript_variant	HLA-DPA1	ENSG00000235844	ENST00000480521.1	protein_coding_CDS_not_defined
rs72879614	intron_variant,non_coding_transcript_variant	HLA-DPA1	ENSG00000235844	ENST00000485095.1	retained_intron
rs72879614	intron_variant,non_coding_transcript_variant	HLA-DPA1	ENSG00000235844	ENST00000485095.1	retained_intron
rs72879614	intron_variant,non_coding_transcript_variant	HLA-DPA1	ENSG00000235844	ENST00000485095.1	retained_intron
rs407238	intergenic_variant	-	-	-	-
rs407238	intergenic_variant	-	-	-	-
rs2517723	downstream_gene_variant	HLA-A	ENSG00000224320	ENST00000417978.6	protein_coding
rs2517723	downstream_gene_variant	HLA-A	ENSG00000224320	ENST00000417978.6	protein_coding
rs2517723	downstream_gene_variant	HLA-A	ENSG00000224320	ENST00000417978.6	protein_coding
rs2517723	downstream_gene_variant	HLA-A	ENSG00000224320	ENST00000442939.5	protein_coding
rs2517723	downstream_gene_variant	HLA-A	ENSG00000224320	ENST00000442939.5	protein_coding
rs2517723	downstream_gene_variant	HLA-A	ENSG00000224320	ENST00000442939.5	protein_coding
rs2517723	downstream_gene_variant	HLA-A	ENSG00000224320	ENST00000443552.5	protein_coding
rs2517723	downstream_gene_variant	HLA-A	ENSG00000224320	ENST00000443552.5	protein_coding
rs2517723	downstream_gene_variant	HLA-A	ENSG00000224320	ENST00000443552.5	protein_coding
rs2517723	upstream_gene_variant	-	ENSG00000223708	ENST00000451967.1	unprocessed_pseudogene
rs2517723	upstream_gene_variant	-	ENSG00000223708	ENST00000451967.1	unprocessed_pseudogene
rs2517723	upstream_gene_variant	-	ENSG00000223708	ENST00000451967.1	unprocessed_pseudogene
rs2517723	downstream_gene_variant	HLA-A	ENSG00000224320	ENST00000479675.5	retained_intron
rs2517723	downstream_gene_variant	HLA-A	ENSG00000224320	ENST00000479675.5	retained_intron
rs2517723	downstream_gene_variant	HLA-A	ENSG00000224320	ENST00000479675.5	retained_intron
rs2517723	downstream_gene_variant	HLA-A	ENSG00000224320	ENST00000479969.1	retained_intron
rs2517723	downstream_gene_variant	HLA-A	ENSG00000224320	ENST00000479969.1	retained_intron
rs2517723	downstream_gene_variant	HLA-A	ENSG00000224320	ENST00000479969.1	retained_intron
rs2517723	downstream_gene_variant	HLA-A	ENSG00000224320	ENST00000484494.5	retained_intron
rs2517723	downstream_gene_variant	HLA-A	ENSG00000224320	ENST00000484494.5	retained_intron
rs2517723	downstream_gene_variant	HLA-A	ENSG00000224320	ENST00000484494.5	retained_intron
rs2517723	downstream_gene_variant	HLA-A	ENSG00000224320	ENST00000490454.5	retained_intron
rs2517723	downstream_gene_variant	HLA-A	ENSG00000224320	ENST00000490454.5	retained_intron
rs2517723	downstream_gene_variant	HLA-A	ENSG00000224320	ENST00000490454.5	retained_intron
rs2517723	downstream_gene_variant	HLA-A	ENSG00000224320	ENST00000549869.5	protein_coding
rs2517723	downstream_gene_variant	HLA-A	ENSG00000224320	ENST00000549869.5	protein_coding

rs2517723	downstream_gene_variant	HLA-A	ENSG00000224320	ENST00000549869.5	protein_coding
rs2517723	3_prime_UTR_variant	HLA-A	ENSG00000224320	ENST00000552498.2	protein_coding
rs2517723	3_prime_UTR_variant	HLA-A	ENSG00000224320	ENST00000552498.2	protein_coding
rs2517723	3_prime_UTR_variant	HLA-A	ENSG00000224320	ENST00000552498.2	protein_coding
rs2517723	intron_variant	HLA-A	ENSG00000224320	ENST00000638213.1	protein_coding
rs2517723	intron_variant	HLA-A	ENSG00000224320	ENST00000638213.1	protein_coding
rs2517723	intron_variant	HLA-A	ENSG00000224320	ENST00000638213.1	protein_coding
rs2517723	intron_variant	HLA-A	ENSG00000224320	ENST00000638761.1	protein_coding
rs2517723	intron_variant	HLA-A	ENSG00000224320	ENST00000638761.1	protein_coding
rs2517723	intron_variant	HLA-A	ENSG00000224320	ENST00000638761.1	protein_coding
rs2517985	intron_variant	CCHCR1	ENSG00000234114	ENST00000400351.5	protein_coding
rs2517985	intron_variant	CCHCR1	ENSG00000234114	ENST00000400351.5	protein_coding
rs2517985	intron_variant	CCHCR1	ENSG00000234114	ENST00000400351.5	protein_coding
rs2517985	intron_variant	CCHCR1	ENSG00000234114	ENST00000416163.6	protein_coding
rs2517985	intron_variant	CCHCR1	ENSG00000234114	ENST00000416163.6	protein_coding
rs2517985	intron_variant	CCHCR1	ENSG00000234114	ENST00000416163.6	protein_coding
rs2517985	intron_variant	CCHCR1	ENSG00000234114	ENST00000425620.6	protein_coding
rs2517985	intron_variant	CCHCR1	ENSG00000234114	ENST00000425620.6	protein_coding
rs2517985	intron_variant	CCHCR1	ENSG00000234114	ENST00000425620.6	protein_coding
rs2517985	intron_variant	CCHCR1	ENSG00000234114	ENST00000444737.5	protein_coding
rs2517985	intron_variant	CCHCR1	ENSG00000234114	ENST00000444737.5	protein_coding
rs2517985	intron_variant	CCHCR1	ENSG00000234114	ENST00000444737.5	protein_coding
rs2517985	downstream_gene_variant	CCHCR1	ENSG00000234114	ENST00000450631.5	protein_coding
rs2517985	downstream_gene_variant	CCHCR1	ENSG00000234114	ENST00000450631.5	protein_coding
rs2517985	downstream_gene_variant	CCHCR1	ENSG00000234114	ENST00000450631.5	protein_coding
rs2517985	downstream_gene_variant	CCHCR1	ENSG00000234114	ENST00000451337.5	protein_coding
rs2517985	downstream_gene_variant	CCHCR1	ENSG00000234114	ENST00000451337.5	protein_coding
rs2517985	downstream_gene_variant	CCHCR1	ENSG00000234114	ENST00000451337.5	protein_coding
rs2517985	downstream_gene_variant	CCHCR1	ENSG00000234114	ENST00000452419.1	protein_coding
rs2517985	downstream_gene_variant	CCHCR1	ENSG00000234114	ENST00000452419.1	protein_coding
rs2517985	downstream_gene_variant	CCHCR1	ENSG00000234114	ENST00000452419.1	protein_coding
rs2517985	intron_variant	CCHCR1	ENSG00000234114	ENST00000454570.5	protein_coding
rs2517985	intron_variant	CCHCR1	ENSG00000234114	ENST00000454570.5	protein_coding
rs2517985	intron_variant	CCHCR1	ENSG00000234114	ENST00000454570.5	protein_coding
rs2517985	downstream_gene_variant	CCHCR1	ENSG00000234114	ENST00000455669.5	protein_coding
rs2517985	downstream_gene_variant	CCHCR1	ENSG00000234114	ENST00000455669.5	protein_coding
rs2517985	downstream_gene_variant	CCHCR1	ENSG00000234114	ENST00000455669.5	protein_coding

rs2517985	intron_variant,non_coding_transcript_variant	CCHCR1	ENSG00000234114	ENST00000463211.1	retained_intron
rs2517985	intron_variant,non_coding_transcript_variant	CCHCR1	ENSG00000234114	ENST00000463211.1	retained_intron
rs2517985	intron_variant,non_coding_transcript_variant	CCHCR1	ENSG00000234114	ENST00000463211.1	retained_intron
rs2517985	intron_variant,non_coding_transcript_variant	CCHCR1	ENSG00000234114	ENST00000466904.5	protein_coding_CDS_not_defined
rs2517985	intron_variant,non_coding_transcript_variant	CCHCR1	ENSG00000234114	ENST00000466904.5	protein_coding_CDS_not_defined
rs2517985	intron_variant,non_coding_transcript_variant	CCHCR1	ENSG00000234114	ENST00000466904.5	protein_coding_CDS_not_defined
rs2517985	intron_variant	CCHCR1	ENSG00000234114	ENST00000610825.1	protein_coding
rs2517985	intron_variant	CCHCR1	ENSG00000234114	ENST00000610825.1	protein_coding
rs2517985	intron_variant	CCHCR1	ENSG00000234114	ENST00000610825.1	protein_coding
rs2854005	intron_variant	HLA-B	ENSG00000223532	ENST00000640223.1	protein_coding
rs2854005	intron_variant	HLA-B	ENSG00000223532	ENST00000640223.1	protein_coding
rs6909636	downstream_gene_variant	HLA-S	ENSG00000225145	ENST00000417754.1	unprocessed_pseudogene
rs6909636	downstream_gene_variant	-	ENSG00000223971	ENST00000434840.1	processed_pseudogene
rs6909636	upstream_gene_variant	ZDHHC20P2	ENSG00000232659	ENST00000449890.1	processed_pseudogene
rs2507976	upstream_gene_variant	HLA-S	ENSG00000225145	ENST00000417754.1	unprocessed_pseudogene
rs2507976	upstream_gene_variant	HLA-S	ENSG00000225145	ENST00000417754.1	unprocessed_pseudogene
rs2507976	downstream_gene_variant	ZDHHC20P2	ENSG00000232659	ENST00000449890.1	processed_pseudogene
rs2507976	downstream_gene_variant	ZDHHC20P2	ENSG00000232659	ENST00000449890.1	processed_pseudogene
rs9501117	intergenic_variant	-	-	-	-
rs2395045	downstream_gene_variant	PPIAP9	ENSG00000236415	ENST00000443635.1	processed_pseudogene
rs2395045	downstream_gene_variant	PPIAP9	ENSG00000236415	ENST00000443635.1	processed_pseudogene
rs2395045	downstream_gene_variant	PPIAP9	ENSG00000236415	ENST00000443635.1	processed_pseudogene
rs3868087	downstream_gene_variant	PPIAP9	ENSG00000236415	ENST00000443635.1	processed_pseudogene
rs3868087	downstream_gene_variant	PPIAP9	ENSG00000236415	ENST00000443635.1	processed_pseudogene
rs7766862	intron_variant	TNXB	ENSG00000233323	ENST00000383159.6	protein_coding
rs7766862	intron_variant	TNXB	ENSG00000233323	ENST00000383159.6	protein_coding
rs7766862	intron_variant	TNXB	ENSG00000233323	ENST00000549232.1	protein_coding
rs7766862	intron_variant	TNXB	ENSG00000233323	ENST00000549232.1	protein_coding
rs17201588	intron_variant	TNXB	ENSG00000233323	ENST00000383159.6	protein_coding
rs17201588	intron_variant	TNXB	ENSG00000233323	ENST00000549232.1	protein_coding
rs9268000	intergenic_variant	-	-	-	-
rs9271509	intergenic_variant	-	-	-	-
rs7745040	intergenic_variant	-	-	-	-
rs7745040	intergenic_variant	-	-	-	-
rs6914052	intergenic_variant	-	-	-	-
rs72879614	upstream_gene_variant	HLA-DPA1	ENSG00000224103	ENST00000414003.1	protein_coding
rs72879614	upstream_gene_variant	HLA-DPA1	ENSG00000224103	ENST00000414003.1	protein_coding

rs72879614	upstream_gene_variant	HLA-DPA1	ENSG00000224103	ENST00000414003.1	protein_coding
rs72879614	intron_variant	HLA-DPA1	ENSG00000224103	ENST00000415247.6	protein_coding
rs72879614	intron_variant	HLA-DPA1	ENSG00000224103	ENST00000415247.6	protein_coding
rs72879614	intron_variant	HLA-DPA1	ENSG00000224103	ENST00000415247.6	protein_coding
rs72879614	intron_variant	HLA-DPA1	ENSG00000224103	ENST00000425105.5	protein_coding
rs72879614	intron_variant	HLA-DPA1	ENSG00000224103	ENST00000425105.5	protein_coding
rs72879614	intron_variant	HLA-DPA1	ENSG00000224103	ENST00000425105.5	protein_coding
rs72879614	intron_variant	HLA-DPA1	ENSG00000224103	ENST00000439393.1	protein_coding
rs72879614	intron_variant	HLA-DPA1	ENSG00000224103	ENST00000439393.1	protein_coding
rs72879614	intron_variant	HLA-DPA1	ENSG00000224103	ENST00000439393.1	protein_coding
rs72879614	downstream_gene_variant	HLA-DPA1	ENSG00000224103	ENST00000448742.1	protein_coding
rs72879614	downstream_gene_variant	HLA-DPA1	ENSG00000224103	ENST00000448742.1	protein_coding
rs72879614	downstream_gene_variant	HLA-DPA1	ENSG00000224103	ENST00000448742.1	protein_coding
rs72879614	intron_variant,non_coding_transcript_variant	HLA-DPA1	ENSG00000224103	ENST00000484255.1	protein_coding_CDS_not_defined
rs72879614	intron_variant,non_coding_transcript_variant	HLA-DPA1	ENSG00000224103	ENST00000484255.1	protein_coding_CDS_not_defined
rs72879614	intron_variant,non_coding_transcript_variant	HLA-DPA1	ENSG00000224103	ENST00000484255.1	protein_coding_CDS_not_defined
rs72879614	intron_variant,non_coding_transcript_variant	HLA-DPA1	ENSG00000224103	ENST00000486369.1	retained_intron
rs72879614	intron_variant,non_coding_transcript_variant	HLA-DPA1	ENSG00000224103	ENST00000486369.1	retained_intron
rs72879614	intron_variant,non_coding_transcript_variant	HLA-DPA1	ENSG00000224103	ENST00000486369.1	retained_intron
rs72879614	intron_variant,non_coding_transcript_variant	HLA-DPA1	ENSG00000224103	ENST00000497834.5	protein_coding_CDS_not_defined
rs72879614	intron_variant,non_coding_transcript_variant	HLA-DPA1	ENSG00000224103	ENST00000497834.5	protein_coding_CDS_not_defined
rs72879614	intron_variant,non_coding_transcript_variant	HLA-DPA1	ENSG00000224103	ENST00000497834.5	protein_coding_CDS_not_defined
rs407238	intergenic_variant	-	-	-	-
rs407238	intergenic_variant	-	-	-	-
rs2517723	upstream_gene_variant	-	ENSG00000225114	ENST00000428199.1	unprocessed_pseudogene
rs2517723	upstream_gene_variant	-	ENSG00000225114	ENST00000428199.1	unprocessed_pseudogene
rs2517723	upstream_gene_variant	-	ENSG00000225114	ENST00000428199.1	unprocessed_pseudogene
rs2517723	downstream_gene_variant	HLA-A	ENSG00000235657	ENST00000438861.6	protein_coding
rs2517723	downstream_gene_variant	HLA-A	ENSG00000235657	ENST00000438861.6	protein_coding
rs2517723	downstream_gene_variant	HLA-A	ENSG00000235657	ENST00000438861.6	protein_coding
rs2517723	downstream_gene_variant	HLA-A	ENSG00000235657	ENST00000453975.5	protein_coding
rs2517723	downstream_gene_variant	HLA-A	ENSG00000235657	ENST00000453975.5	protein_coding
rs2517723	downstream_gene_variant	HLA-A	ENSG00000235657	ENST00000453975.5	protein_coding
rs2517723	downstream_gene_variant	HLA-A	ENSG00000235657	ENST00000457879.5	protein_coding
rs2517723	downstream_gene_variant	HLA-A	ENSG00000235657	ENST00000457879.5	protein_coding
rs2517723	downstream_gene_variant	HLA-A	ENSG00000235657	ENST00000457879.5	protein_coding
rs2517723	downstream_gene_variant	HLA-A	ENSG00000235657	ENST00000460542.5	retained_intron

rs2517723	downstream_gene_variant	HLA-A	ENSG00000235657	ENST00000460542.5	retained_intron
rs2517723	downstream_gene_variant	HLA-A	ENSG00000235657	ENST00000460542.5	retained_intron
rs2517723	downstream_gene_variant	HLA-A	ENSG00000235657	ENST00000468666.5	retained_intron
rs2517723	downstream_gene_variant	HLA-A	ENSG00000235657	ENST00000468666.5	retained_intron
rs2517723	downstream_gene_variant	HLA-A	ENSG00000235657	ENST00000468666.5	retained_intron
rs2517723	downstream_gene_variant	HLA-A	ENSG00000235657	ENST00000470534.1	retained_intron
rs2517723	downstream_gene_variant	HLA-A	ENSG00000235657	ENST00000470534.1	retained_intron
rs2517723	downstream_gene_variant	HLA-A	ENSG00000235657	ENST00000470534.1	retained_intron
rs2517723	downstream_gene_variant	HLA-A	ENSG00000235657	ENST00000487534.5	retained_intron
rs2517723	downstream_gene_variant	HLA-A	ENSG00000235657	ENST00000487534.5	retained_intron
rs2517723	downstream_gene_variant	HLA-A	ENSG00000235657	ENST00000487534.5	retained_intron
rs2517723	downstream_gene_variant	HLA-A	ENSG00000235657	ENST00000547271.2	protein_coding
rs2517723	downstream_gene_variant	HLA-A	ENSG00000235657	ENST00000547271.2	protein_coding
rs2517723	downstream_gene_variant	HLA-A	ENSG00000235657	ENST00000547271.2	protein_coding
rs2517723	intron_variant	HLA-A	ENSG00000235657	ENST00000638608.1	protein_coding
rs2517723	intron_variant	HLA-A	ENSG00000235657	ENST00000638608.1	protein_coding
rs2517723	intron_variant	HLA-A	ENSG00000235657	ENST00000638608.1	protein_coding
rs2517723	downstream_gene_variant	HLA-A	ENSG00000235657	ENST00000640080.1	protein_coding
rs2517723	downstream_gene_variant	HLA-A	ENSG00000235657	ENST00000640080.1	protein_coding
rs2517723	downstream_gene_variant	HLA-A	ENSG00000235657	ENST00000640080.1	protein_coding
rs2517985	intron_variant	CCHCR1	ENSG00000206355	ENST00000383341.8	protein_coding
rs2517985	intron_variant	CCHCR1	ENSG00000206355	ENST00000383341.8	protein_coding
rs2517985	intron_variant	CCHCR1	ENSG00000206355	ENST00000383341.8	protein_coding
rs2517985	intron_variant	CCHCR1	ENSG00000206355	ENST00000400352.7	protein_coding
rs2517985	intron_variant	CCHCR1	ENSG00000206355	ENST00000400352.7	protein_coding
rs2517985	intron_variant	CCHCR1	ENSG00000206355	ENST00000400352.7	protein_coding
rs2517985	intron_variant	CCHCR1	ENSG00000206355	ENST00000414614.5	protein_coding
rs2517985	intron_variant	CCHCR1	ENSG00000206355	ENST00000414614.5	protein_coding
rs2517985	intron_variant	CCHCR1	ENSG00000206355	ENST00000414614.5	protein_coding
rs2517985	downstream_gene_variant	CCHCR1	ENSG00000206355	ENST00000416552.5	protein_coding
rs2517985	downstream_gene_variant	CCHCR1	ENSG00000206355	ENST00000416552.5	protein_coding
rs2517985	downstream_gene_variant	CCHCR1	ENSG00000206355	ENST00000416552.5	protein_coding
rs2517985	downstream_gene_variant	CCHCR1	ENSG00000206355	ENST00000417501.5	protein_coding
rs2517985	downstream_gene_variant	CCHCR1	ENSG00000206355	ENST00000417501.5	protein_coding
rs2517985	downstream_gene_variant	CCHCR1	ENSG00000206355	ENST00000417501.5	protein_coding
rs2517985	downstream_gene_variant	CCHCR1	ENSG00000206355	ENST00000437918.5	protein_coding
rs2517985	downstream_gene_variant	CCHCR1	ENSG00000206355	ENST00000437918.5	protein_coding

rs2517985	downstream_gene_variant	CCHCR1	ENSG00000206355	ENST00000437918.5	protein_coding
rs2517985	intron_variant	CCHCR1	ENSG00000206355	ENST00000438812.5	protein_coding
rs2517985	intron_variant	CCHCR1	ENSG00000206355	ENST00000438812.5	protein_coding
rs2517985	intron_variant	CCHCR1	ENSG00000206355	ENST00000438812.5	protein_coding
rs2517985	downstream_gene_variant	CCHCR1	ENSG00000206355	ENST00000444855.5	protein_coding
rs2517985	downstream_gene_variant	CCHCR1	ENSG00000206355	ENST00000444855.5	protein_coding
rs2517985	downstream_gene_variant	CCHCR1	ENSG00000206355	ENST00000444855.5	protein_coding
rs2517985	intron_variant	CCHCR1	ENSG00000206355	ENST00000449364.5	protein_coding
rs2517985	intron_variant	CCHCR1	ENSG00000206355	ENST00000449364.5	protein_coding
rs2517985	intron_variant	CCHCR1	ENSG00000206355	ENST00000449364.5	protein_coding
rs2517985	intron_variant,non_coding_transcript_variant	CCHCR1	ENSG00000206355	ENST00000475620.1	retained_intron
rs2517985	intron_variant,non_coding_transcript_variant	CCHCR1	ENSG00000206355	ENST00000475620.1	retained_intron
rs2517985	intron_variant,non_coding_transcript_variant	CCHCR1	ENSG00000206355	ENST00000475620.1	retained_intron
rs2517985	intron_variant,non_coding_transcript_variant	CCHCR1	ENSG00000206355	ENST00000486976.1	protein_coding_CDS_not_defined
rs2517985	intron_variant,non_coding_transcript_variant	CCHCR1	ENSG00000206355	ENST00000486976.1	protein_coding_CDS_not_defined
rs2517985	intron_variant,non_coding_transcript_variant	CCHCR1	ENSG00000206355	ENST00000486976.1	protein_coding_CDS_not_defined
rs2517985	intron_variant	CCHCR1	ENSG00000206355	ENST00000613123.1	protein_coding
rs2517985	intron_variant	CCHCR1	ENSG00000206355	ENST00000613123.1	protein_coding
rs2517985	intron_variant	CCHCR1	ENSG00000206355	ENST00000613123.1	protein_coding
rs2854005	intergenic_variant	-	-	-	-
rs2854005	intergenic_variant	-	-	-	-
rs2395045	downstream_gene_variant	PPIAP9	ENSG00000231118	ENST00000447554.1	processed_pseudogene
rs2395045	downstream_gene_variant	PPIAP9	ENSG00000231118	ENST00000447554.1	processed_pseudogene
rs2395045	downstream_gene_variant	PPIAP9	ENSG00000231118	ENST00000447554.1	processed_pseudogene
rs3868087	downstream_gene_variant	PPIAP9	ENSG00000231118	ENST00000447554.1	processed_pseudogene
rs3868087	downstream_gene_variant	PPIAP9	ENSG00000231118	ENST00000447554.1	processed_pseudogene
rs7766862	intron_variant	TNXB	ENSG00000231608	ENST00000440248.5	protein_coding
rs7766862	intron_variant	TNXB	ENSG00000231608	ENST00000440248.5	protein_coding
rs7766862	intron_variant	TNXB	ENSG00000231608	ENST00000550212.1	protein_coding
rs7766862	intron_variant	TNXB	ENSG00000231608	ENST00000550212.1	protein_coding
rs17201588	intron_variant	TNXB	ENSG00000231608	ENST00000440248.5	protein_coding
rs17201588	intron_variant	TNXB	ENSG00000231608	ENST00000550212.1	protein_coding
rs9268000	intergenic_variant	-	-	-	-
rs9271509	intergenic_variant	-	-	-	-
rs7745040	intergenic_variant	-	-	-	-
rs7745040	intergenic_variant	-	-	-	-
rs6914052	intergenic_variant	-	-	-	-

rs72879614	downstream_gene_variant	HLA-DPA1	ENSG00000229685	ENST00000423630.1	protein_coding
rs72879614	downstream_gene_variant	HLA-DPA1	ENSG00000229685	ENST00000423630.1	protein_coding
rs72879614	downstream_gene_variant	HLA-DPA1	ENSG00000229685	ENST00000423630.1	protein_coding
rs72879614	intron_variant	HLA-DPA1	ENSG00000229685	ENST00000436183.1	protein_coding
rs72879614	intron_variant	HLA-DPA1	ENSG00000229685	ENST00000436183.1	protein_coding
rs72879614	intron_variant	HLA-DPA1	ENSG00000229685	ENST00000436183.1	protein_coding
rs72879614	intron_variant	HLA-DPA1	ENSG00000229685	ENST00000437676.1	protein_coding
rs72879614	intron_variant	HLA-DPA1	ENSG00000229685	ENST00000437676.1	protein_coding
rs72879614	intron_variant	HLA-DPA1	ENSG00000229685	ENST00000437676.1	protein_coding
rs72879614	upstream_gene_variant	HLA-DPA1	ENSG00000229685	ENST00000442259.1	protein_coding
rs72879614	upstream_gene_variant	HLA-DPA1	ENSG00000229685	ENST00000442259.1	protein_coding
rs72879614	upstream_gene_variant	HLA-DPA1	ENSG00000229685	ENST00000442259.1	protein_coding
rs72879614	intron_variant	HLA-DPA1	ENSG00000229685	ENST00000454805.5	protein_coding
rs72879614	intron_variant	HLA-DPA1	ENSG00000229685	ENST00000454805.5	protein_coding
rs72879614	intron_variant	HLA-DPA1	ENSG00000229685	ENST00000454805.5	protein_coding
rs72879614	intron_variant,non_coding_transcript_variant	HLA-DPA1	ENSG00000229685	ENST00000475500.1	protein_coding_CDS_not_defined
rs72879614	intron_variant,non_coding_transcript_variant	HLA-DPA1	ENSG00000229685	ENST00000475500.1	protein_coding_CDS_not_defined
rs72879614	intron_variant,non_coding_transcript_variant	HLA-DPA1	ENSG00000229685	ENST00000475500.1	protein_coding_CDS_not_defined
rs72879614	intron_variant,non_coding_transcript_variant	HLA-DPA1	ENSG00000229685	ENST00000484244.1	retained_intron
rs72879614	intron_variant,non_coding_transcript_variant	HLA-DPA1	ENSG00000229685	ENST00000484244.1	retained_intron
rs72879614	intron_variant,non_coding_transcript_variant	HLA-DPA1	ENSG00000229685	ENST00000484244.1	retained_intron
rs72879614	intron_variant,non_coding_transcript_variant	HLA-DPA1	ENSG00000229685	ENST00000486449.5	protein_coding_CDS_not_defined
rs72879614	intron_variant,non_coding_transcript_variant	HLA-DPA1	ENSG00000229685	ENST00000486449.5	protein_coding_CDS_not_defined
rs72879614	intron_variant,non_coding_transcript_variant	HLA-DPA1	ENSG00000229685	ENST00000486449.5	protein_coding_CDS_not_defined
rs407238	intergenic_variant	-	-	-	-
rs407238	intergenic_variant	-	-	-	-
rs2517723	upstream_gene_variant	-	ENSG00000226109	ENST00000442281.1	unprocessed_pseudogene
rs2517723	upstream_gene_variant	-	ENSG00000226109	ENST00000442281.1	unprocessed_pseudogene
rs2517723	upstream_gene_variant	-	ENSG00000226109	ENST00000442281.1	unprocessed_pseudogene
rs2517723	downstream_gene_variant	HLA-A	ENSG00000231834	ENST00000450342.5	protein_coding
rs2517723	downstream_gene_variant	HLA-A	ENSG00000231834	ENST00000450342.5	protein_coding
rs2517723	downstream_gene_variant	HLA-A	ENSG00000231834	ENST00000450342.5	protein_coding
rs2517723	downstream_gene_variant	HLA-A	ENSG00000231834	ENST00000456012.5	protein_coding
rs2517723	downstream_gene_variant	HLA-A	ENSG00000231834	ENST00000456012.5	protein_coding
rs2517723	downstream_gene_variant	HLA-A	ENSG00000231834	ENST00000456012.5	protein_coding
rs2517723	downstream_gene_variant	HLA-A	ENSG00000231834	ENST00000462184.1	retained_intron
rs2517723	downstream_gene_variant	HLA-A	ENSG00000231834	ENST00000462184.1	retained_intron

rs2517723	downstream_gene_variant	HLA-A	ENSG00000231834	ENST00000462184.1	retained_intron
rs2517723	downstream_gene_variant	HLA-A	ENSG00000231834	ENST00000470780.5	nonsense_mediated_decay
rs2517723	downstream_gene_variant	HLA-A	ENSG00000231834	ENST00000470780.5	nonsense_mediated_decay
rs2517723	downstream_gene_variant	HLA-A	ENSG00000231834	ENST00000470780.5	nonsense_mediated_decay
rs2517723	downstream_gene_variant	HLA-A	ENSG00000231834	ENST00000473059.5	retained_intron
rs2517723	downstream_gene_variant	HLA-A	ENSG00000231834	ENST00000473059.5	retained_intron
rs2517723	downstream_gene_variant	HLA-A	ENSG00000231834	ENST00000473059.5	retained_intron
rs2517723	downstream_gene_variant	HLA-A	ENSG00000231834	ENST00000481018.5	retained_intron
rs2517723	downstream_gene_variant	HLA-A	ENSG00000231834	ENST00000481018.5	retained_intron
rs2517723	downstream_gene_variant	HLA-A	ENSG00000231834	ENST00000481018.5	retained_intron
rs2517723	downstream_gene_variant	HLA-A	ENSG00000231834	ENST00000492759.5	retained_intron
rs2517723	downstream_gene_variant	HLA-A	ENSG00000231834	ENST00000492759.5	retained_intron
rs2517723	downstream_gene_variant	HLA-A	ENSG00000231834	ENST00000492759.5	retained_intron
rs2517723	downstream_gene_variant	HLA-A	ENSG00000231834	ENST00000550728.2	protein_coding
rs2517723	downstream_gene_variant	HLA-A	ENSG00000231834	ENST00000550728.2	protein_coding
rs2517723	downstream_gene_variant	HLA-A	ENSG00000231834	ENST00000550728.2	protein_coding
rs2517723	downstream_gene_variant	HLA-A	ENSG00000231834	ENST00000638576.1	protein_coding
rs2517723	downstream_gene_variant	HLA-A	ENSG00000231834	ENST00000638576.1	protein_coding
rs2517723	downstream_gene_variant	HLA-A	ENSG00000231834	ENST00000638576.1	protein_coding
rs2517985	intron_variant	CCHCR1	ENSG00000223533	ENST00000416392.6	protein_coding
rs2517985	intron_variant	CCHCR1	ENSG00000223533	ENST00000416392.6	protein_coding
rs2517985	intron_variant	CCHCR1	ENSG00000223533	ENST00000416392.6	protein_coding
rs2517985	downstream_gene_variant	CCHCR1	ENSG00000223533	ENST00000416850.5	protein_coding
rs2517985	downstream_gene_variant	CCHCR1	ENSG00000223533	ENST00000416850.5	protein_coding
rs2517985	downstream_gene_variant	CCHCR1	ENSG00000223533	ENST00000416850.5	protein_coding
rs2517985	downstream_gene_variant	CCHCR1	ENSG00000223533	ENST00000432736.5	protein_coding
rs2517985	downstream_gene_variant	CCHCR1	ENSG00000223533	ENST00000432736.5	protein_coding
rs2517985	downstream_gene_variant	CCHCR1	ENSG00000223533	ENST00000432736.5	protein_coding
rs2517985	intron_variant	CCHCR1	ENSG00000223533	ENST00000447252.6	protein_coding
rs2517985	intron_variant	CCHCR1	ENSG00000223533	ENST00000447252.6	protein_coding
rs2517985	intron_variant	CCHCR1	ENSG00000223533	ENST00000447252.6	protein_coding
rs2517985	intron_variant	CCHCR1	ENSG00000223533	ENST00000447874.5	protein_coding
rs2517985	intron_variant	CCHCR1	ENSG00000223533	ENST00000447874.5	protein_coding
rs2517985	intron_variant	CCHCR1	ENSG00000223533	ENST00000447874.5	protein_coding
rs2517985	intron_variant	CCHCR1	ENSG00000223533	ENST00000452333.5	protein_coding
rs2517985	intron_variant	CCHCR1	ENSG00000223533	ENST00000452333.5	protein_coding
rs2517985	intron_variant	CCHCR1	ENSG00000223533	ENST00000452333.5	protein_coding

rs2517985	downstream_gene_variant	CCHCR1	ENSG00000223533	ENST00000453360.5	protein_coding
rs2517985	downstream_gene_variant	CCHCR1	ENSG00000223533	ENST00000453360.5	protein_coding
rs2517985	downstream_gene_variant	CCHCR1	ENSG00000223533	ENST00000453360.5	protein_coding
rs2517985	intron_variant	CCHCR1	ENSG00000223533	ENST00000456365.5	protein_coding
rs2517985	intron_variant	CCHCR1	ENSG00000223533	ENST00000456365.5	protein_coding
rs2517985	intron_variant	CCHCR1	ENSG00000223533	ENST00000456365.5	protein_coding
rs2517985	downstream_gene_variant	CCHCR1	ENSG00000223533	ENST00000457929.5	protein_coding
rs2517985	downstream_gene_variant	CCHCR1	ENSG00000223533	ENST00000457929.5	protein_coding
rs2517985	downstream_gene_variant	CCHCR1	ENSG00000223533	ENST00000457929.5	protein_coding
rs2517985	intron_variant,non_coding_transcript_variant	CCHCR1	ENSG00000223533	ENST00000473623.1	retained_intron
rs2517985	intron_variant,non_coding_transcript_variant	CCHCR1	ENSG00000223533	ENST00000473623.1	retained_intron
rs2517985	intron_variant,non_coding_transcript_variant	CCHCR1	ENSG00000223533	ENST00000473623.1	retained_intron
rs2517985	intron_variant,non_coding_transcript_variant	CCHCR1	ENSG00000223533	ENST00000493009.1	protein_coding_CDS_not_defined
rs2517985	intron_variant,non_coding_transcript_variant	CCHCR1	ENSG00000223533	ENST00000493009.1	protein_coding_CDS_not_defined
rs2517985	intron_variant,non_coding_transcript_variant	CCHCR1	ENSG00000223533	ENST00000493009.1	protein_coding_CDS_not_defined
rs2517985	intron_variant	CCHCR1	ENSG00000223533	ENST00000622518.1	protein_coding
rs2517985	intron_variant	CCHCR1	ENSG00000223533	ENST00000622518.1	protein_coding
rs2517985	intron_variant	CCHCR1	ENSG00000223533	ENST00000622518.1	protein_coding
rs2854005	intron_variant	HLA-B	ENSG00000224608	ENST00000639633.1	protein_coding
rs2854005	intron_variant	HLA-B	ENSG00000224608	ENST00000639633.1	protein_coding
rs6909636	downstream_gene_variant	-	ENSG00000224131	ENST00000430251.1	processed_pseudogene
rs6909636	upstream_gene_variant	ZDHHC20P2	ENSG00000226732	ENST00000433773.1	processed_pseudogene
rs6909636	downstream_gene_variant	HLA-S	ENSG00000228179	ENST00000440712.1	unprocessed_pseudogene
rs2507976	downstream_gene_variant	ZDHHC20P2	ENSG00000226732	ENST00000433773.1	processed_pseudogene
rs2507976	downstream_gene_variant	ZDHHC20P2	ENSG00000226732	ENST00000433773.1	processed_pseudogene
rs2507976	upstream_gene_variant	HLA-S	ENSG00000228179	ENST00000440712.1	unprocessed_pseudogene
rs2507976	upstream_gene_variant	HLA-S	ENSG00000228179	ENST00000440712.1	unprocessed_pseudogene
rs9501117	intergenic_variant	-	-	-	-
rs2395045	downstream_gene_variant	PPIAP9	ENSG00000229216	ENST00000420447.1	processed_pseudogene
rs2395045	downstream_gene_variant	PPIAP9	ENSG00000229216	ENST00000420447.1	processed_pseudogene
rs2395045	downstream_gene_variant	PPIAP9	ENSG00000229216	ENST00000420447.1	processed_pseudogene
rs3868087	downstream_gene_variant	PPIAP9	ENSG00000229216	ENST00000420447.1	processed_pseudogene
rs3868087	downstream_gene_variant	PPIAP9	ENSG00000229216	ENST00000420447.1	processed_pseudogene
rs7766862	intron_variant	TNXB	ENSG00000236221	ENST00000451159.1	protein_coding
rs7766862	intron_variant	TNXB	ENSG00000236221	ENST00000451159.1	protein_coding
rs17201588	intron_variant	TNXB	ENSG00000236221	ENST00000451159.1	protein_coding
rs9268000	intergenic_variant	-	-	-	-

rs9271509	intergenic_variant	-	-	-	-
rs7745040	intergenic_variant	-	-	-	-
rs7745040	intergenic_variant	-	-	-	-
rs72879614	intron_variant	HLA-DPA1	ENSG00000228163	ENST00000422504.5	protein_coding
rs72879614	intron_variant	HLA-DPA1	ENSG00000228163	ENST00000422504.5	protein_coding
rs72879614	intron_variant	HLA-DPA1	ENSG00000228163	ENST00000422504.5	protein_coding
rs72879614	downstream_gene_variant	HLA-DPA1	ENSG00000228163	ENST00000427518.1	protein_coding
rs72879614	downstream_gene_variant	HLA-DPA1	ENSG00000228163	ENST00000427518.1	protein_coding
rs72879614	downstream_gene_variant	HLA-DPA1	ENSG00000228163	ENST00000427518.1	protein_coding
rs72879614	intron_variant	HLA-DPA1	ENSG00000228163	ENST00000452619.1	protein_coding
rs72879614	intron_variant	HLA-DPA1	ENSG00000228163	ENST00000452619.1	protein_coding
rs72879614	intron_variant	HLA-DPA1	ENSG00000228163	ENST00000452619.1	protein_coding
rs72879614	upstream_gene_variant	HLA-DPA1	ENSG00000228163	ENST00000455869.1	protein_coding
rs72879614	upstream_gene_variant	HLA-DPA1	ENSG00000228163	ENST00000455869.1	protein_coding
rs72879614	upstream_gene_variant	HLA-DPA1	ENSG00000228163	ENST00000455869.1	protein_coding
rs72879614	intron_variant	HLA-DPA1	ENSG00000228163	ENST00000456525.1	protein_coding
rs72879614	intron_variant	HLA-DPA1	ENSG00000228163	ENST00000456525.1	protein_coding
rs72879614	intron_variant	HLA-DPA1	ENSG00000228163	ENST00000456525.1	protein_coding
rs72879614	intron_variant,non_coding_transcript_variant	HLA-DPA1	ENSG00000228163	ENST00000472997.1	retained_intron
rs72879614	intron_variant,non_coding_transcript_variant	HLA-DPA1	ENSG00000228163	ENST00000472997.1	retained_intron
rs72879614	intron_variant,non_coding_transcript_variant	HLA-DPA1	ENSG00000228163	ENST00000472997.1	retained_intron
rs72879614	intron_variant,non_coding_transcript_variant	HLA-DPA1	ENSG00000228163	ENST00000494775.1	protein_coding_CDS_not_defined
rs72879614	intron_variant,non_coding_transcript_variant	HLA-DPA1	ENSG00000228163	ENST00000494775.1	protein_coding_CDS_not_defined
rs72879614	intron_variant,non_coding_transcript_variant	HLA-DPA1	ENSG00000228163	ENST00000494775.1	protein_coding_CDS_not_defined
rs72879614	intron_variant,non_coding_transcript_variant	HLA-DPA1	ENSG00000228163	ENST00000495074.5	protein_coding_CDS_not_defined
rs72879614	intron_variant,non_coding_transcript_variant	HLA-DPA1	ENSG00000228163	ENST00000495074.5	protein_coding_CDS_not_defined
rs72879614	intron_variant,non_coding_transcript_variant	HLA-DPA1	ENSG00000228163	ENST00000495074.5	protein_coding_CDS_not_defined
rs407238	intergenic_variant	-	-	-	-
rs407238	intergenic_variant	-	-	-	-
rs2517723	downstream_gene_variant	HLA-A	ENSG00000227715	ENST00000413609.5	protein_coding
rs2517723	downstream_gene_variant	HLA-A	ENSG00000227715	ENST00000413609.5	protein_coding
rs2517723	downstream_gene_variant	HLA-A	ENSG00000227715	ENST00000413609.5	protein_coding
rs2517723	upstream_gene_variant	-	ENSG00000228921	ENST00000445820.1	unprocessed_pseudogene
rs2517723	upstream_gene_variant	-	ENSG00000228921	ENST00000445820.1	unprocessed_pseudogene
rs2517723	upstream_gene_variant	-	ENSG00000228921	ENST00000445820.1	unprocessed_pseudogene
rs2517723	downstream_gene_variant	HLA-A	ENSG00000227715	ENST00000453057.5	protein_coding
rs2517723	downstream_gene_variant	HLA-A	ENSG00000227715	ENST00000453057.5	protein_coding

rs2517723	downstream_gene_variant	HLA-A	ENSG00000227715	ENST00000453057.5	protein_coding
rs2517723	downstream_gene_variant	HLA-A	ENSG00000227715	ENST00000455882.5	protein_coding
rs2517723	downstream_gene_variant	HLA-A	ENSG00000227715	ENST00000455882.5	protein_coding
rs2517723	downstream_gene_variant	HLA-A	ENSG00000227715	ENST00000455882.5	protein_coding
rs2517723	downstream_gene_variant	HLA-A	ENSG00000227715	ENST00000471291.5	retained_intron
rs2517723	downstream_gene_variant	HLA-A	ENSG00000227715	ENST00000471291.5	retained_intron
rs2517723	downstream_gene_variant	HLA-A	ENSG00000227715	ENST00000471291.5	retained_intron
rs2517723	downstream_gene_variant	HLA-A	ENSG00000227715	ENST00000476132.5	retained_intron
rs2517723	downstream_gene_variant	HLA-A	ENSG00000227715	ENST00000476132.5	retained_intron
rs2517723	downstream_gene_variant	HLA-A	ENSG00000227715	ENST00000476132.5	retained_intron
rs2517723	downstream_gene_variant	HLA-A	ENSG00000227715	ENST00000478186.1	retained_intron
rs2517723	downstream_gene_variant	HLA-A	ENSG00000227715	ENST00000478186.1	retained_intron
rs2517723	downstream_gene_variant	HLA-A	ENSG00000227715	ENST00000478186.1	retained_intron
rs2517723	downstream_gene_variant	HLA-A	ENSG00000227715	ENST00000479494.5	retained_intron
rs2517723	downstream_gene_variant	HLA-A	ENSG00000227715	ENST00000479494.5	retained_intron
rs2517723	downstream_gene_variant	HLA-A	ENSG00000227715	ENST00000479494.5	retained_intron
rs2517985	intron_variant	CCHCR1	ENSG00000224180	ENST00000419528.6	protein_coding
rs2517985	intron_variant	CCHCR1	ENSG00000224180	ENST00000419528.6	protein_coding
rs2517985	intron_variant	CCHCR1	ENSG00000224180	ENST00000419528.6	protein_coding
rs2517985	downstream_gene_variant	CCHCR1	ENSG00000224180	ENST00000423825.5	protein_coding
rs2517985	downstream_gene_variant	CCHCR1	ENSG00000224180	ENST00000423825.5	protein_coding
rs2517985	downstream_gene_variant	CCHCR1	ENSG00000224180	ENST00000423825.5	protein_coding
rs2517985	downstream_gene_variant	CCHCR1	ENSG00000224180	ENST00000425351.5	protein_coding
rs2517985	downstream_gene_variant	CCHCR1	ENSG00000224180	ENST00000425351.5	protein_coding
rs2517985	downstream_gene_variant	CCHCR1	ENSG00000224180	ENST00000425351.5	protein_coding
rs2517985	downstream_gene_variant	CCHCR1	ENSG00000224180	ENST00000427654.5	protein_coding
rs2517985	downstream_gene_variant	CCHCR1	ENSG00000224180	ENST00000427654.5	protein_coding
rs2517985	downstream_gene_variant	CCHCR1	ENSG00000224180	ENST00000427654.5	protein_coding
rs2517985	intron_variant	CCHCR1	ENSG00000224180	ENST00000428677.5	protein_coding
rs2517985	intron_variant	CCHCR1	ENSG00000224180	ENST00000428677.5	protein_coding
rs2517985	intron_variant	CCHCR1	ENSG00000224180	ENST00000428677.5	protein_coding
rs2517985	intron_variant	CCHCR1	ENSG00000224180	ENST00000433804.6	protein_coding
rs2517985	intron_variant	CCHCR1	ENSG00000224180	ENST00000433804.6	protein_coding
rs2517985	intron_variant	CCHCR1	ENSG00000224180	ENST00000433804.6	protein_coding
rs2517985	downstream_gene_variant	CCHCR1	ENSG00000224180	ENST00000438424.5	protein_coding
rs2517985	downstream_gene_variant	CCHCR1	ENSG00000224180	ENST00000438424.5	protein_coding
rs2517985	downstream_gene_variant	CCHCR1	ENSG00000224180	ENST00000438424.5	protein_coding

rs2517985	intron_variant	CCHCR1	ENSG00000224180	ENST00000438739.5	protein_coding
rs2517985	intron_variant	CCHCR1	ENSG00000224180	ENST00000438739.5	protein_coding
rs2517985	intron_variant	CCHCR1	ENSG00000224180	ENST00000438739.5	protein_coding
rs2517985	intron_variant	CCHCR1	ENSG00000224180	ENST00000439536.5	protein_coding
rs2517985	intron_variant	CCHCR1	ENSG00000224180	ENST00000439536.5	protein_coding
rs2517985	intron_variant	CCHCR1	ENSG00000224180	ENST00000439536.5	protein_coding
rs2517985	intron_variant,non_coding_transcript_variant	CCHCR1	ENSG00000224180	ENST00000471519.1	protein_coding_CDS_not_defined
rs2517985	intron_variant,non_coding_transcript_variant	CCHCR1	ENSG00000224180	ENST00000471519.1	protein_coding_CDS_not_defined
rs2517985	intron_variant,non_coding_transcript_variant	CCHCR1	ENSG00000224180	ENST00000471519.1	protein_coding_CDS_not_defined
rs2517985	intron_variant,non_coding_transcript_variant	CCHCR1	ENSG00000224180	ENST00000498184.1	retained_intron
rs2517985	intron_variant,non_coding_transcript_variant	CCHCR1	ENSG00000224180	ENST00000498184.1	retained_intron
rs2517985	intron_variant,non_coding_transcript_variant	CCHCR1	ENSG00000224180	ENST00000498184.1	retained_intron
rs2517985	intron_variant	CCHCR1	ENSG00000224180	ENST00000611796.1	protein_coding
rs2517985	intron_variant	CCHCR1	ENSG00000224180	ENST00000611796.1	protein_coding
rs2517985	intron_variant	CCHCR1	ENSG00000224180	ENST00000611796.1	protein_coding
rs2854005	intron_variant	HLA-B	ENSG00000232126	ENST00000639001.1	protein_coding
rs2854005	intron_variant	HLA-B	ENSG00000232126	ENST00000639001.1	protein_coding
rs2395045	downstream_gene_variant	PPIAP9	ENSG00000229706	ENST00000443254.1	processed_pseudogene
rs2395045	downstream_gene_variant	PPIAP9	ENSG00000229706	ENST00000443254.1	processed_pseudogene
rs2395045	downstream_gene_variant	PPIAP9	ENSG00000229706	ENST00000443254.1	processed_pseudogene
rs3868087	downstream_gene_variant	PPIAP9	ENSG00000229706	ENST00000443254.1	processed_pseudogene
rs3868087	downstream_gene_variant	PPIAP9	ENSG00000229706	ENST00000443254.1	processed_pseudogene
rs7766862	intron_variant	TNXB	ENSG00000206258	ENST00000424718.5	protein_coding
rs7766862	intron_variant	TNXB	ENSG00000206258	ENST00000424718.5	protein_coding
rs7766862	intron_variant	TNXB	ENSG00000206258	ENST00000548649.1	protein_coding
rs7766862	intron_variant	TNXB	ENSG00000206258	ENST00000548649.1	protein_coding
rs17201588	intron_variant	TNXB	ENSG00000206258	ENST00000424718.5	protein_coding
rs17201588	intron_variant	TNXB	ENSG00000206258	ENST00000548649.1	protein_coding
rs7745040	intergenic_variant	-	-	-	-
rs7745040	intergenic_variant	-	-	-	-
rs6914052	intergenic_variant	-	-	-	-
rs72879614	intron_variant	HLA-DPA1	ENSG00000168384	ENST00000374808.6	protein_coding
rs72879614	intron_variant	HLA-DPA1	ENSG00000168384	ENST00000374808.6	protein_coding
rs72879614	intron_variant	HLA-DPA1	ENSG00000168384	ENST00000374808.6	protein_coding
rs72879614	intron_variant	HLA-DPA1	ENSG00000168384	ENST00000414177.1	protein_coding
rs72879614	intron_variant	HLA-DPA1	ENSG00000168384	ENST00000414177.1	protein_coding
rs72879614	intron_variant	HLA-DPA1	ENSG00000168384	ENST00000414177.1	protein_coding

rs72879614	upstream_gene_variant	HLA-DPA1	ENSG00000168384	ENST00000429960.1	protein_coding
rs72879614	upstream_gene_variant	HLA-DPA1	ENSG00000168384	ENST00000429960.1	protein_coding
rs72879614	upstream_gene_variant	HLA-DPA1	ENSG00000168384	ENST00000429960.1	protein_coding
rs72879614	downstream_gene_variant	HLA-DPA1	ENSG00000168384	ENST00000432811.1	protein_coding
rs72879614	downstream_gene_variant	HLA-DPA1	ENSG00000168384	ENST00000432811.1	protein_coding
rs72879614	downstream_gene_variant	HLA-DPA1	ENSG00000168384	ENST00000432811.1	protein_coding
rs72879614	intron_variant	HLA-DPA1	ENSG00000168384	ENST00000441306.1	protein_coding
rs72879614	intron_variant	HLA-DPA1	ENSG00000168384	ENST00000441306.1	protein_coding
rs72879614	intron_variant	HLA-DPA1	ENSG00000168384	ENST00000441306.1	protein_coding
rs72879614	intron_variant,non_coding_transcript_variant	HLA-DPA1	ENSG00000168384	ENST00000480481.5	protein_coding_CDS_not_defined
rs72879614	intron_variant,non_coding_transcript_variant	HLA-DPA1	ENSG00000168384	ENST00000480481.5	protein_coding_CDS_not_defined
rs72879614	intron_variant,non_coding_transcript_variant	HLA-DPA1	ENSG00000168384	ENST00000480481.5	protein_coding_CDS_not_defined
rs72879614	intron_variant,non_coding_transcript_variant	HLA-DPA1	ENSG00000168384	ENST00000480689.1	retained_intron
rs72879614	intron_variant,non_coding_transcript_variant	HLA-DPA1	ENSG00000168384	ENST00000480689.1	retained_intron
rs72879614	intron_variant,non_coding_transcript_variant	HLA-DPA1	ENSG00000168384	ENST00000480689.1	retained_intron
rs72879614	intron_variant,non_coding_transcript_variant	HLA-DPA1	ENSG00000168384	ENST00000488565.1	protein_coding_CDS_not_defined
rs72879614	intron_variant,non_coding_transcript_variant	HLA-DPA1	ENSG00000168384	ENST00000488565.1	protein_coding_CDS_not_defined
rs72879614	intron_variant,non_coding_transcript_variant	HLA-DPA1	ENSG00000168384	ENST00000488565.1	protein_coding_CDS_not_defined
rs407238	intergenic_variant	-	-	-	-
rs407238	intergenic_variant	-	-	-	-
rs2517723	downstream_gene_variant	HLA-A	ENSG00000206505	ENST00000376822.6	protein_coding
rs2517723	downstream_gene_variant	HLA-A	ENSG00000206505	ENST00000376822.6	protein_coding
rs2517723	downstream_gene_variant	HLA-A	ENSG00000206505	ENST00000376822.6	protein_coding
rs2517723	downstream_gene_variant	HLA-A	ENSG00000206505	ENST00000383605.6	protein_coding
rs2517723	downstream_gene_variant	HLA-A	ENSG00000206505	ENST00000383605.6	protein_coding
rs2517723	downstream_gene_variant	HLA-A	ENSG00000206505	ENST00000383605.6	protein_coding
rs2517723	downstream_gene_variant	HLA-A	ENSG00000206505	ENST00000383619.7	protein_coding
rs2517723	downstream_gene_variant	HLA-A	ENSG00000206505	ENST00000383619.7	protein_coding
rs2517723	downstream_gene_variant	HLA-A	ENSG00000206505	ENST00000383619.7	protein_coding
rs2517723	upstream_gene_variant	-	ENSG00000235952	ENST00000453518.1	unprocessed_pseudogene
rs2517723	upstream_gene_variant	-	ENSG00000235952	ENST00000453518.1	unprocessed_pseudogene
rs2517723	upstream_gene_variant	-	ENSG00000235952	ENST00000453518.1	unprocessed_pseudogene
rs2517723	downstream_gene_variant	HLA-A	ENSG00000206505	ENST00000461996.5	retained_intron
rs2517723	downstream_gene_variant	HLA-A	ENSG00000206505	ENST00000461996.5	retained_intron
rs2517723	downstream_gene_variant	HLA-A	ENSG00000206505	ENST00000461996.5	retained_intron
rs2517723	downstream_gene_variant	HLA-A	ENSG00000206505	ENST00000462536.5	retained_intron
rs2517723	downstream_gene_variant	HLA-A	ENSG00000206505	ENST00000462536.5	retained_intron

rs2517723	downstream_gene_variant	HLA-A	ENSG00000206505	ENST00000462536.5	retained_intron
rs2517723	downstream_gene_variant	HLA-A	ENSG00000206505	ENST00000478740.5	retained_intron
rs2517723	downstream_gene_variant	HLA-A	ENSG00000206505	ENST00000478740.5	retained_intron
rs2517723	downstream_gene_variant	HLA-A	ENSG00000206505	ENST00000478740.5	retained_intron
rs2517723	downstream_gene_variant	HLA-A	ENSG00000206505	ENST00000492107.1	retained_intron
rs2517723	downstream_gene_variant	HLA-A	ENSG00000206505	ENST00000492107.1	retained_intron
rs2517723	downstream_gene_variant	HLA-A	ENSG00000206505	ENST00000492107.1	retained_intron
rs2517723	downstream_gene_variant	HLA-A	ENSG00000206505	ENST00000549224.3	protein_coding
rs2517723	downstream_gene_variant	HLA-A	ENSG00000206505	ENST00000549224.3	protein_coding
rs2517723	downstream_gene_variant	HLA-A	ENSG00000206505	ENST00000549224.3	protein_coding
rs2517723	3_prime_UTR_variant	HLA-A	ENSG00000206505	ENST00000616730.3	protein_coding
rs2517723	3_prime_UTR_variant	HLA-A	ENSG00000206505	ENST00000616730.3	protein_coding
rs2517723	3_prime_UTR_variant	HLA-A	ENSG00000206505	ENST00000616730.3	protein_coding
rs2517723	intron_variant	HLA-A	ENSG00000206505	ENST00000638354.1	protein_coding
rs2517723	intron_variant	HLA-A	ENSG00000206505	ENST00000638354.1	protein_coding
rs2517723	intron_variant	HLA-A	ENSG00000206505	ENST00000638354.1	protein_coding
rs2517723	downstream_gene_variant	HLA-A	ENSG00000206505	ENST00000639891.1	protein_coding
rs2517723	downstream_gene_variant	HLA-A	ENSG00000206505	ENST00000639891.1	protein_coding
rs2517723	downstream_gene_variant	HLA-A	ENSG00000206505	ENST00000639891.1	protein_coding
rs2517985	intron_variant	CCHCR1	ENSG00000206457	ENST00000383527.8	protein_coding
rs2517985	intron_variant	CCHCR1	ENSG00000206457	ENST00000383527.8	protein_coding
rs2517985	intron_variant	CCHCR1	ENSG00000206457	ENST00000383527.8	protein_coding
rs2517985	intron_variant	CCHCR1	ENSG00000206457	ENST00000400406.5	protein_coding
rs2517985	intron_variant	CCHCR1	ENSG00000206457	ENST00000400406.5	protein_coding
rs2517985	intron_variant	CCHCR1	ENSG00000206457	ENST00000400406.5	protein_coding
rs2517985	intron_variant	CCHCR1	ENSG00000206457	ENST00000400412.7	protein_coding
rs2517985	intron_variant	CCHCR1	ENSG00000206457	ENST00000400412.7	protein_coding
rs2517985	intron_variant	CCHCR1	ENSG00000206457	ENST00000400412.7	protein_coding
rs2517985	downstream_gene_variant	CCHCR1	ENSG00000206457	ENST00000412577.5	protein_coding
rs2517985	downstream_gene_variant	CCHCR1	ENSG00000206457	ENST00000412577.5	protein_coding
rs2517985	downstream_gene_variant	CCHCR1	ENSG00000206457	ENST00000412577.5	protein_coding
rs2517985	downstream_gene_variant	CCHCR1	ENSG00000206457	ENST00000425500.5	protein_coding
rs2517985	downstream_gene_variant	CCHCR1	ENSG00000206457	ENST00000425500.5	protein_coding
rs2517985	downstream_gene_variant	CCHCR1	ENSG00000206457	ENST00000425500.5	protein_coding
rs2517985	intron_variant	CCHCR1	ENSG00000206457	ENST00000429943.5	protein_coding
rs2517985	intron_variant	CCHCR1	ENSG00000206457	ENST00000429943.5	protein_coding
rs2517985	intron_variant	CCHCR1	ENSG00000206457	ENST00000429943.5	protein_coding

rs2517985	downstream_gene_variant	CCHCR1	ENSG00000206457	ENST00000437027.1	protein_coding
rs2517985	downstream_gene_variant	CCHCR1	ENSG00000206457	ENST00000437027.1	protein_coding
rs2517985	downstream_gene_variant	CCHCR1	ENSG00000206457	ENST00000437027.1	protein_coding
rs2517985	intron_variant	CCHCR1	ENSG00000206457	ENST00000439820.5	protein_coding
rs2517985	intron_variant	CCHCR1	ENSG00000206457	ENST00000439820.5	protein_coding
rs2517985	intron_variant	CCHCR1	ENSG00000206457	ENST00000439820.5	protein_coding
rs2517985	downstream_gene_variant	CCHCR1	ENSG00000206457	ENST00000456712.1	protein_coding
rs2517985	downstream_gene_variant	CCHCR1	ENSG00000206457	ENST00000456712.1	protein_coding
rs2517985	downstream_gene_variant	CCHCR1	ENSG00000206457	ENST00000456712.1	protein_coding
rs2517985	intron_variant,non_coding_transcript_variant	CCHCR1	ENSG00000206457	ENST00000465277.1	retained_intron
rs2517985	intron_variant,non_coding_transcript_variant	CCHCR1	ENSG00000206457	ENST00000465277.1	retained_intron
rs2517985	intron_variant,non_coding_transcript_variant	CCHCR1	ENSG00000206457	ENST00000465277.1	retained_intron
rs2517985	intron_variant,non_coding_transcript_variant	CCHCR1	ENSG00000206457	ENST00000482045.5	protein_coding_CDS_not_defined
rs2517985	intron_variant,non_coding_transcript_variant	CCHCR1	ENSG00000206457	ENST00000482045.5	protein_coding_CDS_not_defined
rs2517985	intron_variant,non_coding_transcript_variant	CCHCR1	ENSG00000206457	ENST00000482045.5	protein_coding_CDS_not_defined
rs2854005	intron_variant	HLA-B	ENSG00000206450	ENST00000639564.1	protein_coding
rs2854005	intron_variant	HLA-B	ENSG00000206450	ENST00000639564.1	protein_coding
rs6909636	downstream_gene_variant	HLA-S	ENSG00000237559	ENST00000419729.1	unprocessed_pseudogene
rs6909636	upstream_gene_variant	ZDHHC20P2	ENSG00000231773	ENST00000423129.1	processed_pseudogene
rs6909636	downstream_gene_variant	-	ENSG00000232276	ENST00000454711.1	processed_pseudogene
rs2507976	upstream_gene_variant	HLA-S	ENSG00000237559	ENST00000419729.1	unprocessed_pseudogene
rs2507976	upstream_gene_variant	HLA-S	ENSG00000237559	ENST00000419729.1	unprocessed_pseudogene
rs2507976	downstream_gene_variant	ZDHHC20P2	ENSG00000231773	ENST00000423129.1	processed_pseudogene
rs2507976	downstream_gene_variant	ZDHHC20P2	ENSG00000231773	ENST00000423129.1	processed_pseudogene
rs2395045	downstream_gene_variant	PPIAP9	ENSG00000225317	ENST00000418291.1	processed_pseudogene
rs2395045	downstream_gene_variant	PPIAP9	ENSG00000225317	ENST00000418291.1	processed_pseudogene
rs2395045	downstream_gene_variant	PPIAP9	ENSG00000225317	ENST00000418291.1	processed_pseudogene
rs3868087	downstream_gene_variant	PPIAP9	ENSG00000225317	ENST00000418291.1	processed_pseudogene
rs3868087	downstream_gene_variant	PPIAP9	ENSG00000225317	ENST00000418291.1	processed_pseudogene
rs7766862	intron_variant	TNXB	ENSG00000229353	ENST00000424713.5	protein_coding
rs7766862	intron_variant	TNXB	ENSG00000229353	ENST00000424713.5	protein_coding
rs9268000	intergenic_variant	-	-	-	-
rs9271509	intergenic_variant	-	-	-	-
rs7745040	intergenic_variant	-	-	-	-
rs7745040	intergenic_variant	-	-	-	-
rs6914052	intergenic_variant	-	-	-	-
rs72879614	intron_variant	HLA-DPA1	ENSG00000206291	ENST00000383224.6	protein_coding

rs72879614	intron_variant	HLA-DPA1	ENSG00000206291	ENST00000383224.6	protein_coding
rs72879614	intron_variant	HLA-DPA1	ENSG00000206291	ENST00000383224.6	protein_coding
rs72879614	upstream_gene_variant	HLA-DPA1	ENSG00000206291	ENST00000428180.1	protein_coding
rs72879614	upstream_gene_variant	HLA-DPA1	ENSG00000206291	ENST00000428180.1	protein_coding
rs72879614	upstream_gene_variant	HLA-DPA1	ENSG00000206291	ENST00000428180.1	protein_coding
rs72879614	intron_variant	HLA-DPA1	ENSG00000206291	ENST00000437123.1	protein_coding
rs72879614	intron_variant	HLA-DPA1	ENSG00000206291	ENST00000437123.1	protein_coding
rs72879614	intron_variant	HLA-DPA1	ENSG00000206291	ENST00000437123.1	protein_coding
rs72879614	downstream_gene_variant	HLA-DPA1	ENSG00000206291	ENST00000439867.1	protein_coding
rs72879614	downstream_gene_variant	HLA-DPA1	ENSG00000206291	ENST00000439867.1	protein_coding
rs72879614	downstream_gene_variant	HLA-DPA1	ENSG00000206291	ENST00000439867.1	protein_coding
rs72879614	intron_variant	HLA-DPA1	ENSG00000206291	ENST00000441483.1	protein_coding
rs72879614	intron_variant	HLA-DPA1	ENSG00000206291	ENST00000441483.1	protein_coding
rs72879614	intron_variant	HLA-DPA1	ENSG00000206291	ENST00000441483.1	protein_coding
rs72879614	intron_variant,non_coding_transcript_variant	HLA-DPA1	ENSG00000206291	ENST00000474244.1	protein_coding_CDS_not_defined
rs72879614	intron_variant,non_coding_transcript_variant	HLA-DPA1	ENSG00000206291	ENST00000474244.1	protein_coding_CDS_not_defined
rs72879614	intron_variant,non_coding_transcript_variant	HLA-DPA1	ENSG00000206291	ENST00000474244.1	protein_coding_CDS_not_defined
rs72879614	intron_variant,non_coding_transcript_variant	HLA-DPA1	ENSG00000206291	ENST00000481258.1	retained_intron
rs72879614	intron_variant,non_coding_transcript_variant	HLA-DPA1	ENSG00000206291	ENST00000481258.1	retained_intron
rs72879614	intron_variant,non_coding_transcript_variant	HLA-DPA1	ENSG00000206291	ENST00000481258.1	retained_intron
rs72879614	intron_variant,non_coding_transcript_variant	HLA-DPA1	ENSG00000206291	ENST00000483480.5	protein_coding_CDS_not_defined
rs72879614	intron_variant,non_coding_transcript_variant	HLA-DPA1	ENSG00000206291	ENST00000483480.5	protein_coding_CDS_not_defined
rs72879614	intron_variant,non_coding_transcript_variant	HLA-DPA1	ENSG00000206291	ENST00000483480.5	protein_coding_CDS_not_defined
rs407238	intergenic_variant	-	-	-	-
rs407238	intergenic_variant	-	-	-	-
rs2517723	downstream_gene_variant	HLA-A	ENSG00000223980	ENST00000414592.5	protein_coding
rs2517723	downstream_gene_variant	HLA-A	ENSG00000223980	ENST00000414592.5	protein_coding
rs2517723	downstream_gene_variant	HLA-A	ENSG00000223980	ENST00000414592.5	protein_coding
rs2517723	upstream_gene_variant	-	ENSG00000231067	ENST00000450479.1	unprocessed_pseudogene
rs2517723	upstream_gene_variant	-	ENSG00000231067	ENST00000450479.1	unprocessed_pseudogene
rs2517723	upstream_gene_variant	-	ENSG00000231067	ENST00000450479.1	unprocessed_pseudogene
rs2517723	downstream_gene_variant	HLA-A	ENSG00000223980	ENST00000454091.5	protein_coding
rs2517723	downstream_gene_variant	HLA-A	ENSG00000223980	ENST00000454091.5	protein_coding
rs2517723	downstream_gene_variant	HLA-A	ENSG00000223980	ENST00000454091.5	protein_coding
rs2517723	downstream_gene_variant	HLA-A	ENSG00000223980	ENST00000462986.1	retained_intron
rs2517723	downstream_gene_variant	HLA-A	ENSG00000223980	ENST00000462986.1	retained_intron
rs2517723	downstream_gene_variant	HLA-A	ENSG00000223980	ENST00000462986.1	retained_intron

rs2517723	downstream_gene_variant	HLA-A	ENSG00000223980	ENST00000463148.5	retained_intron
rs2517723	downstream_gene_variant	HLA-A	ENSG00000223980	ENST00000463148.5	retained_intron
rs2517723	downstream_gene_variant	HLA-A	ENSG00000223980	ENST00000463148.5	retained_intron
rs2517723	downstream_gene_variant	HLA-A	ENSG00000223980	ENST00000464713.5	retained_intron
rs2517723	downstream_gene_variant	HLA-A	ENSG00000223980	ENST00000464713.5	retained_intron
rs2517723	downstream_gene_variant	HLA-A	ENSG00000223980	ENST00000464713.5	retained_intron
rs2517723	downstream_gene_variant	HLA-A	ENSG00000223980	ENST00000478258.5	retained_intron
rs2517723	downstream_gene_variant	HLA-A	ENSG00000223980	ENST00000478258.5	retained_intron
rs2517723	downstream_gene_variant	HLA-A	ENSG00000223980	ENST00000478258.5	retained_intron
rs2517723	downstream_gene_variant	HLA-A	ENSG00000223980	ENST00000488889.5	protein_coding_CDS_not_defined
rs2517723	downstream_gene_variant	HLA-A	ENSG00000223980	ENST00000488889.5	protein_coding_CDS_not_defined
rs2517723	downstream_gene_variant	HLA-A	ENSG00000223980	ENST00000488889.5	protein_coding_CDS_not_defined
rs2517723	downstream_gene_variant	HLA-A	ENSG00000223980	ENST00000552493.4	protein_coding
rs2517723	downstream_gene_variant	HLA-A	ENSG00000223980	ENST00000552493.4	protein_coding
rs2517723	downstream_gene_variant	HLA-A	ENSG00000223980	ENST00000552493.4	protein_coding
rs2517723	downstream_gene_variant	HLA-A	ENSG00000223980	ENST00000612644.2	protein_coding
rs2517723	downstream_gene_variant	HLA-A	ENSG00000223980	ENST00000612644.2	protein_coding
rs2517723	downstream_gene_variant	HLA-A	ENSG00000223980	ENST00000612644.2	protein_coding
rs2517723	intron_variant	HLA-A	ENSG00000223980	ENST00000638335.1	protein_coding
rs2517723	intron_variant	HLA-A	ENSG00000223980	ENST00000638335.1	protein_coding
rs2517723	intron_variant	HLA-A	ENSG00000223980	ENST00000638335.1	protein_coding
rs2854005	intron_variant	HLA-B	ENSG00000228964	ENST00000640675.1	protein_coding
rs2854005	intron_variant	HLA-B	ENSG00000228964	ENST00000640675.1	protein_coding
rs6909636	downstream_gene_variant	HLA-S	ENSG00000226027	ENST00000416556.1	unprocessed_pseudogene
rs6909636	upstream_gene_variant	ZDHHC20P2	ENSG00000227405	ENST00000427014.1	processed_pseudogene
rs6909636	downstream_gene_variant	-	ENSG00000232244	ENST00000447153.1	processed_pseudogene
rs2507976	upstream_gene_variant	HLA-S	ENSG00000226027	ENST00000416556.1	unprocessed_pseudogene
rs2507976	upstream_gene_variant	HLA-S	ENSG00000226027	ENST00000416556.1	unprocessed_pseudogene
rs2507976	downstream_gene_variant	ZDHHC20P2	ENSG00000227405	ENST00000427014.1	processed_pseudogene
rs2507976	downstream_gene_variant	ZDHHC20P2	ENSG00000227405	ENST00000427014.1	processed_pseudogene
rs9501117	intergenic_variant	-	-	-	-
rs2395045	downstream_gene_variant	PPIAP9	ENSG00000235596	ENST00000442288.1	processed_pseudogene
rs2395045	downstream_gene_variant	PPIAP9	ENSG00000235596	ENST00000442288.1	processed_pseudogene
rs2395045	downstream_gene_variant	PPIAP9	ENSG00000235596	ENST00000442288.1	processed_pseudogene
rs3868087	downstream_gene_variant	PPIAP9	ENSG00000235596	ENST00000442288.1	processed_pseudogene
rs3868087	downstream_gene_variant	PPIAP9	ENSG00000235596	ENST00000442288.1	processed_pseudogene
rs7766862	intron_variant	TNXB	ENSG00000236236	ENST00000412618.5	protein_coding

rs7766862	intron_variant	TNXB	ENSG00000236236	ENST00000412618.5	protein_coding
rs9268000	intergenic_variant	-	-	-	-
rs9271509	intergenic_variant	-	-	-	-
rs7745040	intergenic_variant	-	-	-	-
rs7745040	intergenic_variant	-	-	-	-
rs6914052	intergenic_variant	-	-	-	-
rs72879614	downstream_gene_variant	HLA-DPA1	ENSG00000236177	ENST00000448154.1	protein_coding
rs72879614	downstream_gene_variant	HLA-DPA1	ENSG00000236177	ENST00000448154.1	protein_coding
rs72879614	downstream_gene_variant	HLA-DPA1	ENSG00000236177	ENST00000448154.1	protein_coding
rs72879614	upstream_gene_variant	HLA-DPA1	ENSG00000236177	ENST00000453314.1	protein_coding
rs72879614	upstream_gene_variant	HLA-DPA1	ENSG00000236177	ENST00000453314.1	protein_coding
rs72879614	upstream_gene_variant	HLA-DPA1	ENSG00000236177	ENST00000453314.1	protein_coding
rs72879614	intron_variant,non_coding_transcript_variant	HLA-DPA1	ENSG00000236177	ENST00000502285.1	protein_coding_CDS_not_defined
rs72879614	intron_variant,non_coding_transcript_variant	HLA-DPA1	ENSG00000236177	ENST00000502285.1	protein_coding_CDS_not_defined
rs72879614	intron_variant,non_coding_transcript_variant	HLA-DPA1	ENSG00000236177	ENST00000502285.1	protein_coding_CDS_not_defined
rs72879614	intron_variant,non_coding_transcript_variant	HLA-DPA1	ENSG00000236177	ENST00000511285.1	retained_intron
rs72879614	intron_variant,non_coding_transcript_variant	HLA-DPA1	ENSG00000236177	ENST00000511285.1	retained_intron
rs72879614	intron_variant,non_coding_transcript_variant	HLA-DPA1	ENSG00000236177	ENST00000511285.1	retained_intron
rs72879614	intron_variant	HLA-DPA1	ENSG00000236177	ENST00000512750.1	protein_coding
rs72879614	intron_variant	HLA-DPA1	ENSG00000236177	ENST00000512750.1	protein_coding
rs72879614	intron_variant	HLA-DPA1	ENSG00000236177	ENST00000512750.1	protein_coding
rs72879614	intron_variant,non_coding_transcript_variant	HLA-DPA1	ENSG00000236177	ENST00000514979.5	protein_coding_CDS_not_defined
rs72879614	intron_variant,non_coding_transcript_variant	HLA-DPA1	ENSG00000236177	ENST00000514979.5	protein_coding_CDS_not_defined
rs72879614	intron_variant,non_coding_transcript_variant	HLA-DPA1	ENSG00000236177	ENST00000514979.5	protein_coding_CDS_not_defined
rs72879614	intron_variant	HLA-DPA1	ENSG00000236177	ENST00000515317.5	protein_coding
rs72879614	intron_variant	HLA-DPA1	ENSG00000236177	ENST00000515317.5	protein_coding
rs72879614	intron_variant	HLA-DPA1	ENSG00000236177	ENST00000515317.5	protein_coding
rs72879614	intron_variant	HLA-DPA1	ENSG00000236177	ENST00000515346.1	protein_coding
rs72879614	intron_variant	HLA-DPA1	ENSG00000236177	ENST00000515346.1	protein_coding
rs72879614	intron_variant	HLA-DPA1	ENSG00000236177	ENST00000515346.1	protein_coding

SNP: single nucleotide polymorphism. The physical position is based on GRCh37. Index SNP refers to pleiotropic variants and SYMBOL indicates their nearest genes.

Supplementary Table 5. List of SNPs in the 99% credible set identified from fine-mapping analysis for each CPASSOC-identified locus shared between COVID-19 and prostate cancer.

Index SNP	99% credible-set SNPs	Chromosome	Position	CumSum
**Prostate cancer and infection**				
rs12821205	rs11146929	12	133135926	0.8491
	rs11146953	12	133155907	0.9727
	rs11610600	12	133138810	0.9884
	rs11614573	12	133136078	0.7308
	rs12579512	12	133138115	0.9862
	**rs12821205**	12	133133808	0.2949
	rs2178562	12	133132329	0.9439
	rs28435470	12	133067473	0.9772
	rs2875247	12	133129836	0.9025
	rs4475994	12	133135433	0.9675
	rs4883557	12	133120906	0.7938
	rs7295014	12	133067989	0.6625
	rs7301141	12	133138503	0.9838
	rs7973123	12	133136791	0.5424
	rs7973990	12	133137480	0.9903
	rs9971699	12	133137400	0.9813
rs2854005	rs1265067	6	31116142	0.6984
	rs1265069	6	31115700	0.8052
	rs1265076	6	31113083	0.2827
	rs1265078	6	31112602	0.4055
	rs1265079	6	31112108	0.6398
	rs1265087	6	31109810	0.7518
	rs1265092	6	31107317	0.2184
	rs1265111	6	31119380	0.5227
	rs1265112	6	31118019	0.4641
	rs1265114	6	31117188	0.5812
	rs1576	6	31110391	0.9513
	rs2517985	6	31118942	0.1124
	rs2523833	6	31109947	0.8539
	rs2523834	6	31109946	1.0000
	rs746646	6	31113940	0.3469
	rs746647	6	31114182	0.9026
**Prostate cancer and hospitalization**				
rs2395045	rs1065076	6	31477681	0.9393
	rs2286713	6	31473844	0.9856
	**rs2395045**	6	31484517	0.2142
	rs2523670	6	31437492	0.9785
	rs28729827	6	31475811	0.9592
	rs3093952	6	31475764	0.9554
	rs3093953	6	31474688	0.9771
	rs3093955	6	31469649	0.9868
	rs3093977	6	31500226	0.9711
	rs3093982	6	31497244	0.9475
	rs3093999	6	31484550	0.8847
	rs3094000	6	31484054	0.3393
	rs3094001	6	31484032	0.9062
	rs3094002	6	31471222	0.9845

rs2395045 (continued)	rs3094003	6	31469251	0.9822
	rs3095230	6	31470673	0.9901
	rs3095231	6	31470343	0.9879
	rs3095234	6	31464571	0.9798
	rs3095235	6	31463170	0.9743
	rs3130613	6	31481535	0.5850
	rs3130615	6	31475413	0.9660
	rs3130925	6	31463621	0.9757
	rs3130927	6	31464992	0.9810
	rs3131630	6	31485354	0.9728
	rs3131632	6	31483752	0.8541
	rs3131633	6	31480923	0.9304
	rs3131634	6	31477189	0.9626
	rs3131635	6	31476134	0.9434
	rs3131639	6	31473546	0.9834
	rs3132457	6	31484561	0.6806
	rs3132458	6	31481113	0.9248
	rs3132459	6	31481092	0.4621
	rs3132462	6	31480578	0.9351
	rs3132468	6	31475486	0.9516
	rs3134893	6	31476514	0.9694
	rs3134897	6	31466547	0.9890
	rs3868087	6	31484892	0.7696
rs2493732	rs12761299	10	81723884	0.9845
	rs2475735	10	81628355	0.9695
	**rs2493732**	10	81626286	0.9464
	rs3895291	10	81626662	0.9766
	rs56050597	10	81724536	0.9879
	rs61860415	10	81730579	0.9809
	rs7072378	10	81711067	0.9908
rs2507976	**rs2507976**	6	31351887	0.9612
	rs9265885	6	31313220	0.9982
rs2517723	rs2508037	6	29918436	0.9659
	rs2517713	6	29918099	0.9736
	rs2517714	6	29917881	0.9616
	rs2517722	6	29915330	0.7853
	**rs2517723**	6	29915268	0.3768
	rs2523954	6	29914034	0.9867
	rs2524037	6	29914950	0.5088
	rs2571390	6	29923554	0.9773
	rs2916806	6	29918684	0.9699
	rs2916807	6	29918770	0.9913
	rs366907	6	29907755	0.9810
	rs387850	6	29907696	0.9892
	rs393282	6	29942497	0.8734
	rs396379	6	29914500	0.6181
	rs407238	6	29806901	0.9561
	rs414215	6	29906772	0.7260
	rs9260036	6	29907494	0.9462
	rs9260038	6	29907544	0.9048
	rs9260072	6	29908838	0.9841
	rs9260115	6	29910027	0.9348

rs2517723 (continued)	rs9260403	6	29918861	0.8367
rs2517985	rs1265067	6	31116142	0.5271
	rs1265069	6	31115700	0.7006
	rs1265076	6	31113083	0.3833
	rs1265078	6	31112602	0.7504
	rs1265079	6	31112108	0.9794
	rs1265087	6	31109810	0.6471
	rs1265092	6	31107317	0.8842
	rs1265111	6	31119380	0.4555
	rs1265112	6	31118019	0.3085
	rs1265114	6	31117188	0.2248
	rs1265157	6	31142265	0.8600
	rs1576	6	31110391	0.8343
	rs2245738	6	31230221	0.9440
	**rs2517985**	6	31118942	0.1149
	rs2524107	6	31232590	0.9077
	rs2524112	6	31231780	0.9901
	rs2524117	6	31230869	0.9679
	rs2844624	6	31232247	0.9301
	rs2853961	6	31231989	0.5919
	rs746646	6	31113940	0.9562
	rs746647	6	31114182	0.7963
rs2854005	rs1265067	6	31116142	0.6432
	rs1265069	6	31115700	0.7759
	rs1265076	6	31113083	0.4677
	rs1265078	6	31112602	0.8366
	rs1265079	6	31112108	0.9975
	rs1265087	6	31109810	0.7105
	rs1265092	6	31107317	0.9686
	rs1265111	6	31119380	0.5559
	rs1265112	6	31118019	0.3764
	rs1265114	6	31117188	0.2743
	rs1576	6	31110391	0.9390
	rs2517985	6	31118942	0.1402
	rs746646	6	31113940	0.9834
	rs746647	6	31114182	0.8926
rs4475994	rs11146929	12	133135926	0.9155
	rs11614573	12	133136078	0.9879
	rs12821205	12	133133808	0.8298
	rs2178562	12	133132329	0.9959
	rs2875247	12	133129836	0.9556
	**rs4475994**	12	133135433	0.4316
rs61036010	rs11636034	15	31612041	0.8037
	rs11636159	15	31611586	0.6694
	rs1465778	15	31621321	0.5243
	rs34693358	15	31624505	0.9581
	rs4779861	15	31630352	0.9855
	rs4779862	15	31633161	0.9910
	rs58566807	15	31625332	0.9784
	**rs61036010**	15	31622974	0.3617
	rs72722828	15	31638632	0.9819
	rs74409869	15	31618659	0.9117

rs61036010 (continued)	rs80177425	15	31618590	0.8580
	rs8027539	15	31632806	0.9884
	rs9920754	15	31629298	0.9701
rs6909636	rs2844575	6	31334945	0.9993
	**rs6909636**	6	31346599	0.9694
rs72879614	rs10214910	6	33037675	0.4409
	rs1042177	6	33037557	0.8112
	rs1042178	6	33037522	0.6738
	rs1042190	6	33036999	0.8842
	rs1042308	6	33036853	0.8306
	rs1126769	6	33036435	0.9871
	rs112796164	6	33035088	0.9813
	rs115770992	6	33035288	0.9844
	rs116487412	6	33034931	0.9823
	rs13196639	6	33039484	0.9889
	rs13213149	6	33039686	0.9865
	rs13213165	6	33039729	0.9866
	rs13213265	6	33039755	0.9890
	rs1431400	6	33041176	0.4073
	rs146542010	6	33035095	0.9804
	rs17214526	6	33032206	0.9886
	rs17214573	6	33032668	0.9883
	rs17220829	6	33025386	0.9747
	rs17220885	6	33026185	0.9837
	rs199989840	6	33035285	0.9848
	rs200005596	6	33038834	0.9896
	rs201348127	6	33035286	0.9846
	rs201911418	6	33038833	0.9896
	rs2071351	6	33043930	0.2528
	rs2071352	6	33044188	0.9076
	rs2071353	6	33044257	0.2124
	rs2071354	6	33044388	0.8776
	rs2301220	6	33038766	0.4990
	rs2301221	6	33038597	0.6364
	rs2301222	6	33038548	0.6918
	rs2301224	6	33038369	0.3697
	rs2308911	6	33037580	0.9891
	rs2308912	6	33037579	0.9840
	rs2621322	6	32788712	0.9895
	rs28414332	6	33038862	0.9894
	rs28452918	6	33039295	0.9872
	rs34015624	6	33034723	0.9820
	rs34083733	6	33039446	0.1137
	rs34197320	6	33039503	0.9879
	rs34624643	6	33039376	0.9885
	rs34682523	6	33037304	0.8213
	rs34780193	6	33039719	0.9874
	rs34950776	6	33037104	0.8011
	rs35030344	6	33034653	0.9892
	rs35039691	6	33039405	0.9893
	rs35521473	6	33039606	0.9862
	rs35769133	6	33039848	0.9852

rs72879614 (continued)	rs35877170	6	33034702	0.9835
	rs36043556	6	33039837	0.9858
	rs36050357	6	33034699	0.9817
	rs36081724	6	33037265	0.8488
	rs3830066	6	33037187	0.7549
	rs4247257	6	33041226	0.3313
	rs4604307	6	33025671	0.9281
	rs4640928	6	33040596	0.4719
	rs552153841	6	33035097	0.9799
	rs55752341	6	33025044	0.9773
	rs55763051	6	33029937	0.9898
	rs55950816	6	33029827	0.9023
	rs56301606	6	33024908	0.9534
	rs563060972	6	33035091	0.9809
	rs57389774	6	33038579	0.7256
	rs57753382	6	33039180	0.5968
	rs57896011	6	33038947	0.9863
	rs58063871	6	33040478	0.9829
	rs58083473	6	33024972	0.9647
	rs59342333	6	33029599	0.9794
	rs59977910	6	33038979	0.9884
	rs60349783	6	33042492	0.9232
	rs60461436	6	33029639	0.9181
	rs66720176	6	33028988	0.9900
	rs66799959	6	33037690	0.8397
	rs66977180	6	33039196	0.9495
	rs67022713	6	33029276	0.8636
	rs67325643	6	33037991	0.7906
	rs67463587	6	33038322	0.7090
	rs67475104	6	33029409	0.9901
	rs67542233	6	33038802	0.9369
	rs67643877	6	33024823	0.8563
	rs67984906	6	33037768	0.7675
	rs6914348	6	33040654	0.9413
	rs6914849	6	33040715	0.6557
	rs6914995	6	33040744	0.5258
	rs6919972	6	33034177	0.9887
	rs6923131	6	33029729	0.7797
	rs72500560	6	33039927	0.9875
	rs72500562	6	33039980	0.9876
	rs72867695	6	33034354	0.9869
	rs72870105	6	33035134	0.9854
	rs72873927	6	33029255	0.8709
	rs72873929	6	33029326	0.8907
	**rs72879614**	6	33037891	0.0602
	rs72879616	6	33037957	0.8969
	rs72879617	6	33038117	0.7416
	rs72879690	6	33039855	0.9850
	rs73400034	6	33035272	0.9832
	rs73400085	6	33038830	0.9897
	rs73415247	6	33029205	0.9868
	rs73738013	6	33029137	0.9899

rs72879614 (continued)	rs73739639	6	33035306	0.9842
	rs73739674	6	33037865	0.9856
	rs73739675	6	33037866	0.9860
	rs73739677	6	33037878	0.9880
	rs74818705	6	33035158	0.9826
	rs7757860	6	33025740	0.9718
	rs7775838	6	33025712	0.9325
	rs79229871	6	33035143	0.9881
	rs9348904	6	33040835	0.5514
	rs9380335	6	33025174	0.9455
	rs9380336	6	33040736	0.6167
	rs9380337	6	33040781	0.2922
	rs9380338	6	33040798	0.1644
	rs9380339	6	33040877	0.5747
	rs9469344	6	33042368	0.9573
	rs9469345	6	33042406	0.9610
	rs9469347	6	33044258	0.9129
	rs987870	6	33042880	0.9683
	rs9885608	6	33029148	0.9878
rs7766862	rs17201588	6	32057972	0.3951
	rs17207951	6	32050653	0.2735
	rs17421624	6	32066177	0.9721
	rs2071293	6	32062687	0.8772
	rs2071295	6	32038700	0.8298
	rs2077116	6	32021838	0.5046
	rs2228628	6	32088854	0.9453
	rs2239689	6	32030284	0.5995
	rs28361052	6	32032205	0.6921
	rs2857009	6	32019746	0.7661
	rs4713505	6	32105001	0.9178
	**rs7766862**	6	32033007	0.1371
	rs9267802	6	32099817	0.9899
	rs9267803	6	32101762	0.9963
rs800507	rs1014180	8	116839713	0.9807
	rs1040333	8	116825349	0.9113
	rs10505262	8	116697852	0.9717
	rs1065805	8	116803130	0.6981
	rs1065806	8	116803082	0.4135
	rs1089116	8	116804458	0.9695
	rs1089117	8	116805292	0.8025
	rs12682033	8	116701130	0.9342
	rs1405296	8	116747751	0.9774
	rs1405297	8	116747501	0.9849
	rs1474517	8	116825542	0.7297
	rs1474518	8	116825690	0.7597
	rs179456	8	116695112	0.9221
	rs1830141	8	116747737	0.9791
	rs1880814	8	116746477	0.9821
	rs1880815	8	116746285	0.9862
	rs2205265	8	116746746	0.9828
	rs2205266	8	116746899	0.9855
	rs2254401	8	116824700	0.6466

rs800507 (continued)	rs2254555	8	116753600	0.9902
	rs2694033	8	116810032	0.8421
	rs2694035	8	116794593	0.8842
	rs2694036	8	116794276	0.2049
	rs2694037	8	116794231	0.2591
	rs2694041	8	116827273	0.9727
	rs2694045	8	116824201	0.8297
	rs2694047	8	116750548	0.9747
	rs2736202	8	116792426	0.7883
	rs2736203	8	116793567	0.2858
	rs2736207	8	116809655	0.9625
	rs2736208	8	116826244	0.9494
	rs2736209	8	116826356	0.4374
	rs2736212	8	116827228	0.5276
	rs2736215	8	116828721	0.9312
	rs2736222	8	116831768	0.9737
	rs2736233	8	116747364	0.9842
	rs2736234	8	116747824	0.9783
	rs2737240	8	116657235	0.9814
	rs2960148	8	116747789	0.9799
	rs2960151	8	116718620	0.9593
	rs2960152	8	116840670	0.9252
	rs2960155	8	116831832	0.9757
	rs2960156	8	116795877	0.3118
	rs2960157	8	116795541	0.8771
	rs2975506	8	116831946	0.9896
	rs2975508	8	116840770	0.9868
	rs4876612	8	116699039	0.9150
	rs702771	8	116819866	0.5056
	rs702772	8	116806917	0.9186
	rs702773	8	116806658	0.8616
	rs702774	8	116806651	0.6643
	rs702775	8	116806504	0.3634
	rs720928	8	116697876	0.9558
	rs7834801	8	116743801	0.9886
	**rs800507**	8	116796539	0.0800
	rs800508	8	116796912	0.7449
	rs800509	8	116797530	0.7141
	rs800510	8	116798227	0.1770
	rs800511	8	116799168	0.1486
	rs800512	8	116799593	0.1200
	rs800514	8	116800884	0.3376
	rs800516	8	116801367	0.2324
	rs800517	8	116801489	0.6815
	rs800518	8	116801780	0.8535
	rs800519	8	116806029	0.9022
	rs800521	8	116744508	0.9577
	rs800537	8	116721984	0.9835
	rs800538	8	116722990	0.9538
	rs800539	8	116725199	0.9656
	rs800540	8	116791749	0.4833
	rs800541	8	116730138	0.9517

rs800507 (continued)	rs800542	8	116731444	0.9641
	rs800544	8	116823610	0.9283
	rs800545	8	116822646	0.9073
	rs800546	8	116821186	0.8964
	rs800547	8	116820174	0.6102
	rs800548	8	116819887	0.9683
	rs800550	8	116819629	0.9398
	rs800551	8	116819267	0.8906
	rs800552	8	116816756	0.5703
	rs800553	8	116816592	0.5911
	rs800554	8	116816165	0.8165
	rs800557	8	116835859	0.9766
	rs800560	8	116837887	0.9891
	rs800562	8	116709020	0.9472
	rs800566	8	116709834	0.9706
	rs800572	8	116718438	0.9423
	rs800574	8	116717451	0.9609
	rs800585	8	116813923	0.6287
	rs800586	8	116813905	0.9448
	rs800587	8	116813278	0.4605
	rs800588	8	116813143	0.7741
	rs800589	8	116812703	0.3886
	rs800590	8	116812578	0.9670
	rs800591	8	116811386	0.5492
	rs808982	8	116800251	0.9370
	rs813889	8	116798852	0.8695
	rs9297543	8	116704980	0.9880
	rs985161	8	116754143	0.9874
rs9271509	rs3104371	6	32602137	0.8132
	rs3104413	6	32582650	0.9863
	rs3129769	6	32597022	0.9766
	rs3997872	6	32580617	0.9621
	rs510205	6	32584693	0.9914
	rs521539	6	32581973	0.9890
	rs679242	6	32570958	0.9202
	rs6931277	6	32583357	0.8708
	**rs9271509**	6	32589415	0.6545
rs9501117	rs111748703	6	31149228	0.9491
	rs112420503	6	31320097	0.9653
	rs112434986	6	31154814	0.9610
	rs113379855	6	31153548	0.5980
	rs114300451	6	31234537	0.9848
	rs142031128	6	31164689	0.5286
	rs150765547	6	31159581	0.9678
	rs17193150	6	31337092	0.9702
	rs17197686	6	31178540	0.1211
	rs17199474	6	31336960	0.9763
	rs28577989	6	31255762	0.6657
	rs28611443	6	31341411	0.9819
	rs34023488	6	31270295	0.9885
	rs34196875	6	31275603	0.9834
	rs35446580	6	31250796	0.9906

rs9501117 (continued)	rs35624930	6	31272251	0.9861
	rs35734933	6	31257247	0.9896
	rs35795116	6	31298123	0.9744
	rs72863810	6	31179121	0.7327
	rs72863820	6	31182682	0.2315
	rs72863826	6	31187338	0.3381
	rs72863830	6	31189541	0.9550
	rs7450458	6	31418616	0.9185
	rs75430636	6	31347630	0.9801
	rs76188215	6	31347583	0.9782
	rs7765011	6	31427590	0.8639
	rs7765013	6	31427594	0.7997
	rs79245272	6	31427189	0.9426
	rs79493037	6	31405825	0.9873
	rs9295987	6	31349844	0.8917
	rs9295988	6	31373718	0.9723
	**rs9501117**	6	31398591	0.4381
**Prostate cancer and critical illness**				
rs17201588	**rs17201588**	6	32057972	0.1107
	rs17207951	6	32050653	0.3223
	rs17421624	6	32066177	0.9097
	rs2071293	6	32062687	0.8586
	rs2071295	6	32038700	0.7982
	rs2077116	6	32021838	0.7093
	rs2228628	6	32088854	0.9709
	rs2239689	6	32030284	0.5197
	rs28361052	6	32032205	0.6171
	rs2857009	6	32019746	0.4217
	rs4713505	6	32105001	0.9472
	rs4713506	6	32113980	0.9918
	rs7766862	6	32033007	0.2211
	rs9267802	6	32099817	0.9838
	rs9267803	6	32101762	0.9886
rs2854005	rs1265067	6	31116142	0.5368
	rs1265069	6	31115700	0.8114
	rs1265076	6	31113083	0.4049
	rs1265078	6	31112602	0.6536
	rs1265079	6	31112108	0.8626
	rs1265087	6	31109810	0.3213
	rs1265092	6	31107317	0.1254
	rs1265111	6	31119380	0.5988
	rs1265112	6	31118019	0.7067
	rs1265114	6	31117188	0.4713
	rs1576	6	31110391	0.9551
	rs2517985	6	31118942	0.2313
	rs2523833	6	31109947	0.9977
	rs746646	6	31113940	0.7594
	rs746647	6	31114182	0.9112
rs3868087	rs2286713	6	31473844	0.5714
	rs2395045	6	31484517	0.1988
	rs3093953	6	31474688	0.4584
	rs3093955	6	31469649	0.8481

rs3868087 (continued)	rs3093999	6	31484550	0.4172
	rs3094000	6	31484054	0.2598
	rs3094001	6	31484032	0.9902
	rs3094002	6	31471222	0.7163
	rs3094003	6	31469251	0.6085
	rs3095230	6	31470673	0.6817
	rs3095231	6	31470343	0.8164
	rs3095234	6	31464571	0.6455
	rs3095235	6	31463170	0.9788
	rs3130613	6	31481535	0.1342
	rs3130925	6	31463621	0.7504
	rs3130926	6	31463680	0.9275
	rs3130927	6	31464992	0.7836
	rs3131630	6	31485354	0.9829
	rs3131632	6	31483752	0.4965
	rs3131638	6	31475127	0.9419
	rs3131639	6	31473546	0.5342
	rs3132457	6	31484561	0.3745
	rs3132458	6	31481113	0.9866
	rs3132459	6	31481092	0.3198
	rs3132463	6	31479645	0.9745
	rs3134891	6	31476265	0.9647
	rs3134892	6	31476340	0.9538
	rs3134897	6	31466547	0.9096
	rs3134899	6	31473286	0.8797
	**rs3868087**	6	31484892	0.0677
rs407238	rs1610682	6	29801324	0.9797
	rs1611133	6	29809382	0.9683
	rs1611136	6	29811131	0.9593
	rs1611182	6	29781049	0.9755
	rs1961135	6	29914706	0.9908
	rs2252963	6	29932007	0.9396
	rs2508037	6	29918436	0.8458
	rs2517681	6	29932330	0.9350
	rs2517684	6	29931066	0.9052
	rs2517690	6	29929073	0.9479
	rs2517710	6	29918296	0.9891
	rs2517713	6	29918099	0.8869
	rs2517714	6	29917881	0.8570
	rs2517716	6	29917260	0.9816
	rs2517722	6	29915330	0.6527
	rs2517723	6	29915268	0.5583
	rs2517878	6	29813380	0.9709
	rs2523753	6	29734727	0.9304
	rs2523754	6	29734477	0.9190
	rs2523954	6	29914034	0.9253
	rs2524037	6	29914950	0.4305
	rs2571383	6	29914315	0.8965
	rs2571390	6	29923554	0.7621
	rs2571402	6	29932380	0.9626
	rs2735046	6	29734098	0.9519
	rs2844810	6	29923580	0.9900

rs407238 (continued)	rs2916806	6	29918684	0.8227
	rs2916807	6	29918770	0.8770
	rs2916809	6	29920549	0.9859
	rs2975042	6	29920536	0.9847
	rs366907	6	29907755	0.8345
	rs387850	6	29907696	0.9127
	rs396379	6	29914500	0.5104
	**rs407238**	6	29806901	0.2568
	rs414215	6	29906772	0.3477
	rs428081	6	29906512	0.8671
	rs437464	6	29928270	0.9439
	rs4628142	6	29931799	0.9656
	rs9260029	6	29907243	0.9870
	rs9260036	6	29907494	0.7802
	rs9260038	6	29907544	0.7365
	rs9260046	6	29908044	0.9881
	rs9260072	6	29908838	0.8104
	rs9260078	6	29909033	0.9732
	rs9260081	6	29909153	0.9558
	rs9260104	6	29909616	0.9835
	rs9260115	6	29910027	0.6057
	rs9260321	6	29917028	0.9776
	rs9260403	6	29918861	0.6987
	rs9404952	6	29804165	0.7958
rs6914052	rs114132738	6	32996999	0.4862
	rs114545731	6	32990195	0.6779
	rs1431397	6	33005335	0.9753
	rs1431398	6	33005338	0.8656
	rs194676	6	32896330	0.8087
	rs2061952	6	32785714	0.9878
	rs2071475	6	32782387	0.9925
	rs2621344	6	32773278	0.9826
	**rs6914052**	6	33003119	0.2584
	rs9277038	6	33001355	0.9101
	rs9277054	6	33006410	0.9437
rs7295014	rs10870546	12	133054609	0.5758
	rs11146929	12	133135926	0.9184
	rs11614573	12	133136078	0.9465
	rs12821205	12	133133808	0.7399
	rs2178562	12	133132329	0.9776
	rs2875247	12	133129836	0.9692
	rs4400876	12	133097398	0.9892
	rs4475994	12	133135433	0.8674
	rs4883557	12	133120906	0.9878
	rs4883583	12	133056570	0.9907
	**rs7295014**	12	133067989	0.3870
	rs7973123	12	133136791	0.9854
rs7745040	rs6457619	6	32663986	0.9186
	rs6457622	6	32664163	0.6236
	**rs7745040**	6	32664332	0.3187
	rs9275160	6	32652620	0.9795
	rs9275273	6	32662559	0.9932

rs9268000	rs2022533	6	32307260	0.9863
	rs2143463	6	32318729	0.9898
	rs2395145	6	32311871	0.9894
	rs28361060	6	32303848	0.9792
	rs471081	6	32293598	0.9845
	rs4959093	6	32313097	0.9896
	rs504203	6	32294843	0.9892
	rs505274	6	32294992	0.9895
	rs510181	6	32293434	0.9884
	rs522254	6	32273060	0.9833
	rs524578	6	32295357	0.9867
	rs525607	6	32292084	0.9873
	rs531094	6	32286102	0.9891
	rs540639	6	32288564	0.9875
	rs542525	6	32288805	0.9878
	rs547077	6	32289318	0.9870
	rs557539	6	32286680	0.9881
	rs560324	6	32286984	0.9889
	rs574710	6	32288190	0.9887
	rs6910071	6	32282854	0.9825
	rs6913664	6	32295874	0.9777
	rs6929776	6	32303511	0.9860
	**rs9268000**	6	32223531	0.9111
	rs9268145	6	32257284	0.9816
	rs9268301	6	32319637	0.9734
	rs9268362	6	32333341	0.9757
	rs9268363	6	32333410	0.9901
	rs9348880	6	32296780	0.9837
	rs9348881	6	32296842	0.9849
	rs9348882	6	32301322	0.9897
	rs9357140	6	32301514	0.9899
	rs9366793	6	32301289	0.9853
	rs9368714	6	32297341	0.9806
	rs9380290	6	32301452	0.9856
	rs9394087	6	32300848	0.9841
rs9501117	rs11967243	6	31246266	0.4278
	rs17192274	6	31248033	0.4865
	rs17198616	6	31247964	0.9517
	rs2074488	6	31240431	0.8038
	rs2074490	6	31239937	0.9013
	rs34023488	6	31270295	0.9164
	rs34090104	6	31241353	0.7305
	rs34131062	6	31236410	0.8342
	rs34196875	6	31275603	0.1991
	rs34255701	6	31241600	0.7721
	rs34314881	6	31233937	0.8855
	rs34622008	6	31240814	0.9877
	rs34634498	6	31236400	0.8639
	rs34950876	6	31247416	0.5398
	rs35028179	6	31241721	0.6405
	rs35303934	6	31234222	0.9738
	rs35438642	6	31247439	0.6874

rs9501117 (continued)	rs35446580	6	31250796	0.9300
	rs35624930	6	31272251	0.2893
	rs35647108	6	31274449	0.9836
	rs35734933	6	31257247	0.3645
	rs35795116	6	31298123	0.5906
	rs35857242	6	31253121	0.9424
	rs35976302	6	31241077	0.9789
	rs7450458	6	31418616	0.9684
	rs79245272	6	31427189	0.9908
	**rs9501117**	6	31398591	0.9602

SNP: single nucleotide polymorphism; CumSum: cumulative summation of probability of SNP in this credible set.

Supplementary Table 6. Results from colocalization analysis for each pleiotropic locus identified from CPASSOC.

Index SNP	COVID-19		Prostate cancer		$P_{CPASSOC}$	Chromosome: Position	PPH4
	Beta	*P*-value	Beta	*P*-value			
**Prostate cancer and infection**							
**rs12821205**	0.024	1.23E-07	-0.047	6.05E-09	1.20E-11	chr12:133046199..133165564	**0.960**
rs2854005	-0.025	4.43E-07	-0.040	1.97E-06	6.12E-10	chr6:31235746..31413809	0.001
**Prostate cancer and hospitalization**							
rs2395045	-0.047	6.31E-05	-0.048	4.43E-07	3.83E-10	chr6:31228460..31717692	0.000
rs2493732	0.050	1.31E-05	0.043	2.98E-05	5.05E-09	chr10:81318663..81780241	0.054
rs2507976	0.047	3.24E-06	-0.045	8.78E-08	2.66E-12	chr6:31070078..31426619	0.000
rs2517723	-0.062	2.38E-09	0.038	2.47E-06	1.07E-13	chr6:29606761..30056476	0.000
rs2517985	0.048	2.94E-06	-0.064	1.48E-13	1.22E-17	chr6:31003436..31426967	0.000
rs2854005	-0.052	4.22E-07	-0.040	1.97E-06	1.14E-11	chr6:31081940..31419905	0.000
**rs4475994**	0.062	8.72E-11	-0.047	6.07E-09	4.89E-18	chr12:133046199..133165564	**0.766**
rs61036010*	-0.043	3.42E-05	-0.039	2.38E-05	1.05E-08	chr15:31611586..31647722	0.389
rs6909636	-0.055	3.97E-08	0.035	1.58E-05	1.08E-11	chr6:31330411..31359548	0.000
rs72879614	0.052	1.84E-05	-0.056	2.22E-07	5.34E-11	chr6:33024738..33076268	0.246
rs7766862	-0.045	1.31E-05	0.045	1.25E-07	1.73E-11	chr6:31893944..32177900	0.028
**rs800507***	0.046	8.11E-05	0.041	2.43E-05	2.34E-08	chr8:116657235..116840770	**0.709**
rs9271509	-0.059	1.42E-05	0.070	3.52E-12	1.29E-15	chr6:32212655..32710377	0.000
rs9501117	0.103	5.19E-07	-0.075	7.79E-06	5.68E-11	chr6:31137798..31428967	0.000
**Prostate cancer and critical illness**							
rs17201588	-0.059	9.93E-05	0.045	1.16E-07	6.88E-10	chr6:31851469..32179896	0.042
rs2854005	-0.063	3.03E-05	-0.040	1.97E-06	1.03E-08	chr6:31313212..31426967	0.000
rs3868087	-0.072	2.66E-05	-0.048	3.85E-07	1.37E-09	chr6:31228460..31717692	0.000
rs407238	-0.059	3.55E-05	0.047	8.12E-09	2.85E-11	chr6:29699784..29944314	0.152
**rs6914052***	0.122	7.52E-06	-0.069	1.41E-05	4.96E-08	chr6:32844103..33017082	**0.536**
rs7295014	0.078	2.07E-07	-0.052	9.50E-10	9.69E-14	chr12:133038610..133165564	0.010
rs7745040	-0.077	6.76E-08	0.054	3.69E-11	1.14E-15	chr6:32386554..32804798	0.027
rs9268000	-0.057	8.30E-05	0.050	1.84E-09	6.35E-12	chr6:32213210..32587013	0.143
rs9501117	0.147	1.47E-06	-0.075	7.79E-06	1.21E-08	chr6:31137798..31427594	0.000

SNP: single nucleotide polymorphisms; * represents novel shared SNP; PPH4: posterior probability for hypothesis 4 (a joint causal variant). The bolded index SNP probably associates with both traits and shares a single causal variant (PPH4 > 0.5).

Supplementary Table 7. Details of instrumental variables selected for COVID-19 phenotypes.

SNP	CHR	Position	Effect allele	Other allele	Effect allele frequency	Beta	Standard error	*P*-value	Pleiotropic traits	Combined R^2	*F*-statistic
**Infection**											
rs11264339	1	155140648	T	C	0.4947	-0.0350	0.0046	1.62E-14	PR interval	0.001	105.06
rs1123573	2	60707588	G	A	0.3789	-0.0264	0.0048	3.16E-08			
rs73062389	3	45835417	A	G	0.0543	0.2004	0.0099	7.61E-92			
rs35044562	3	45909024	G	A	0.0776	0.1280	0.0085	1.64E-51			
rs2290859	3	101525625	T	C	0.3510	-0.0508	0.0049	1.32E-25			
rs2260685	3	195497743	C	T	0.4744	0.0334	0.0047	1.01E-12			
rs9264740	6	31244331	T	C	0.7321	-0.0305	0.0053	6.87E-09			
rs554833	9	136147160	T	C	0.3484	0.0908	0.0048	5.87E-81			
rs7118388	11	34454147	G	A	0.5057	0.0266	0.0045	4.71E-09			
rs10774673	12	113361158	T	C	0.6754	0.0289	0.0048	2.14E-09			
rs184781326	14	29459234	G	A	0.0472	-0.0690	0.0117	3.42E-09			
rs12610495	19	4717672	G	A	0.3032	0.0495	0.0051	1.80E-22	Interstitial lung disease		
rs142770866	19	10525372	A	G	0.0787	0.0468	0.0084	2.91E-08			
rs679574	19	49206108	G	C	0.4469	-0.0360	0.0046	4.37E-15	LDL cholesterol,Blood protein levels,Chronic inflammatory diseases (ankylosing spondylitis, Crohn's disease, psoriasis, primary sclerosing cholangitis, ulcerative colitis) (pleiotropy)		
rs676314	19	50865535	G	A	0.3256	0.0276	0.0048	1.02E-08			
rs2834158	21	34617213	C	T	0.6611	-0.0405	0.0049	8.51E-17			
**Hospitalization**											
rs3014983	1	65460691	T	C	0.7360	-0.0695	0.0120	6.99E-09		0.001	73.84
rs1498399	1	77947239	G	A	0.3863	0.0688	0.0101	8.24E-12			
rs139589338	1	154826289	G	A	0.0191	0.2090	0.0357	4.97E-09			
rs41264915	1	155167786	G	A	0.0970	-0.1430	0.0154	1.43E-20	Lung function (FEV1/FVC)		
rs1123573	2	60707588	G	A	0.3773	-0.0688	0.0104	4.13E-11			

rs17279437	3	45814094	A	G	0.1067	-0.1137	0.0168	1.37E-11	Blood metabolite ratios,Blood metabolite levels,Macular thickness,Urinary metabolites
rs67959919	3	45871908	A	G	0.0774	0.4916	0.0175	2.36E-173	
rs17412601	3	101499275	C	T	0.3513	-0.0683	0.0103	3.69E-11	
rs34712979	4	106819053	A	G	0.2499	-0.0643	0.0115	2.17E-08	Lung function (FEV1/FVC),Chronic obstructive pulmonary disease,Respiratory diseases,Lung function (FEV1),Lung function (FVC),Peak expiratory flow
rs4475253	5	131776506	G	A	0.3241	0.0582	0.0101	7.07E-09	
rs2517723	6	29915268	T	C	0.6093	-0.0620	0.0104	2.38E-09	
rs1634761	6	31274027	T	C	0.4938	-0.0677	0.0095	1.02E-12	
rs17219281	6	32675645	A	G	0.0797	-0.1245	0.0184	1.16E-11	
rs41435745	6	41490382	C	G	0.0291	0.2131	0.0352	1.45E-09	
rs2897075	7	99630342	T	C	0.3740	0.0585	0.0099	2.88E-09	Chronic obstructive pulmonary disease
rs2326562	8	61428200	T	C	0.3814	0.0531	0.0097	4.81E-08	
rs149533170	9	21172825	A	G	0.0078	0.2839	0.0511	2.78E-08	
rs657152	9	136139265	A	C	0.3864	0.1001	0.0098	1.18E-24	
rs17885848	10	81316456	T	C	0.3374	0.0611	0.0109	1.93E-08	
rs35705950	11	1241221	T	G	0.1073	-0.0989	0.0155	1.90E-10	Idiopathic pulmonary fibrosis
rs61882275	11	34504292	A	G	0.3424	-0.0920	0.0099	2.30E-20	
rs4767025	12	113358794	T	C	0.6762	0.0755	0.0101	7.13E-14	
rs5023077	12	133141973	C	T	0.4910	-0.0661	0.0095	3.79E-12	
rs12585036	13	113535741	T	C	0.2163	0.0967	0.0116	9.39E-17	
rs2102497	16	54255222	C	T	0.7339	0.0644	0.0117	4.21E-08	
rs61078946	16	75640517	T	A	0.1112	-0.0896	0.0162	3.12E-08	Educational attainment (MTAG)
rs117169628	16	89262657	A	G	0.1440	0.1011	0.0137	1.27E-13	
rs63750417	17	44060775	T	C	0.1835	-0.0918	0.0115	1.37E-15	
rs3848456	17	47940623	A	C	0.0316	0.2186	0.0289	3.62E-14	
rs12610495	19	4717672	G	A	0.3045	0.1483	0.0110	4.21E-41	Interstitial lung disease

rs34536443	19	10463118	C	G	0.0413	0.2346	0.0255	3.83E-20	Multiple sclerosis,Systemic lupus erythematosus,Sarcoidosis,Psoriasis vulgaris,Rheumatoid arthritis (ACPA-positive),Autoimmune traits,Rheumatoid arthritis,Juvenile idiopathic arthritis (oligoarticular or rheumatoid factor-negative polyarticular),Platelet count,Hypothyroidism,Type 1 diabetes,Psoriasis,Primary biliary cirrhosis		
rs142770866	19	10525372	A	G	0.0787	0.1323	0.0176	5.73E-14			
rs492602	19	49206417	G	A	0.4729	-0.0550	0.0095	7.81E-09	Blood protein levels,Cholesterol (total),Alcohol use disorder (total score),Vitamin B levels in ischemic stroke,Vitamin B12 levels,Triglycerides,Psoriasis,Low density lipoprotein cholesterol levels,Lung function (FEV1/FVC)		
rs676314	19	50865535	G	A	0.3246	0.0780	0.0101	8.95E-15			
rs9636867	21	34609944	G	A	0.3357	0.1282	0.0102	2.05E-36			
rs78314212	21	35312916	T	C	0.0849	0.1220	0.0172	1.22E-12			
rs915823	21	42851454	C	A	0.2375	-0.0685	0.0125	4.33E-08			
rs548403132	3	46167911	GA	G	0.5990	0.0888	0.0158	1.75E-08			
**Critical illness**											
rs11208559	1	65435283	G	C	0.2912	0.1025	0.0171	2.17E-09		0.003	77.49
rs41264915	1	155167786	G	A	0.0944	-0.2063	0.0229	2.09E-19	Lung function (FEV1/FVC)		
rs568035	1	156110167	T	C	0.0635	0.1431	0.0260	3.60E-08			
rs12614007	2	57316503	A	G	0.7494	0.0941	0.0169	2.46E-08			
rs1123573	2	60707588	G	A	0.3702	-0.1062	0.0152	2.80E-12			

rs17279437	3	45814094	A	G	0.1005	-0.1721	0.0251	7.29E-12	Blood metabolite ratios,Blood metabolite levels,Macular thickness,Urinary metabolites,
rs17713054	3	45859651	A	G	0.0746	0.7558	0.0260	1.09E-185	
rs79152783	3	46913179	G	A	0.0177	0.3801	0.0606	3.47E-10	
rs343320	3	146234909	A	G	0.0700	0.1542	0.0275	2.06E-08	
rs34712979	4	106819053	A	G	0.2540	-0.1095	0.0170	1.21E-10	Lung function (FEV1/FVC),Chronic obstructive pulmonary disease,Respiratory diseases,Lung function (FEV1),Lung function (FVC),Peak expiratory flow
rs10066378	5	131776967	C	T	0.1159	0.1180	0.0210	1.96E-08	
rs396379	6	29914500	T	G	0.5974	-0.0882	0.0143	7.27E-10	
rs1128175	6	31150435	G	A	0.7531	-0.1255	0.0171	2.25E-13	
rs17219281	6	32675645	A	G	0.0818	-0.1768	0.0270	5.88E-11	
rs12534422	7	75263792	T	C	0.3028	0.0856	0.0151	1.34E-08	
rs2897075	7	99630342	T	C	0.3757	0.0880	0.0144	9.32E-10	Chronic obstructive pulmonary disease
rs28368148	9	21206605	G	C	0.0234	0.4488	0.0655	7.26E-12	
rs550057	9	136146597	T	C	0.2739	0.1170	0.0159	2.12E-13	
rs17885848	10	81316456	T	C	0.3385	0.0902	0.0156	7.78E-09	
rs35705950	11	1241221	T	G	0.1085	-0.1636	0.0228	6.95E-13	Idiopathic pulmonary fibrosis
rs61882275	11	34504292	A	G	0.3432	-0.1261	0.0147	8.08E-18	
rs10850097	12	113361117	T	C	0.6710	0.0952	0.0149	1.71E-10	
rs11614702	12	133058157	A	G	0.5069	0.1014	0.0138	2.33E-13	
rs12585036	13	113535741	T	C	0.2133	0.1414	0.0172	2.32E-16	
rs117169628	16	89262657	A	G	0.1370	0.1575	0.0201	4.36E-15	
rs62056905	17	43782693	G	A	0.1838	-0.1285	0.0168	2.18E-14	
rs199512	17	44857352	C	T	0.8203	0.1297	0.0173	5.68E-14	
rs77534576	17	47940666	T	C	0.0339	0.3109	0.0424	2.33E-13	
rs12610495	19	4717672	G	A	0.3130	0.2417	0.0160	3.05E-51	Interstitial lung disease

rs34536443	19	10463118	C	G	0.0401	0.3813	0.0367	3.03E-25	Multiple sclerosis,Systemic lupus erythematosus,Sarcoidosis,Psoriasis vulgaris,Rheumatoid arthritis (ACPA-positive),Autoimmune traits,Rheumatoid arthritis,Juvenile idiopathic arthritis (oligoarticular or rheumatoid factor-negative polyarticular),Platelet count,Hypothyroidism,Type 1 diabetes,Psoriasis,Primary biliary cirrhosis
rs142770866	19	10525372	A	G	0.0826	0.2069	0.0257	7.61E-16	
rs368565	19	49201217	T	C	0.4643	0.1056	0.0149	1.41E-12	
rs60132559	19	50867106	T	C	0.3205	0.0915	0.0150	1.11E-09	
rs9636867	21	34609944	G	A	0.3312	0.1839	0.0151	3.47E-34	
rs2236645	21	35342238	T	C	0.0848	0.1794	0.0250	6.46E-13	
rs9305744	21	42842988	A	G	0.2679	-0.0993	0.0172	8.27E-09	
rs548403132	3	46167911	GA	G	0.7438	0.1374	0.0227	1.34E-09	

SNP: single nucleotide polymorphisms. CHR: chromosome. *F*-statistic was calculated using the following formulas: *F*=R^2 (n-k-1)/k(1-R^2) and R^2=2×β^2×MAF×(1-MAF)/(2×β^2×MAF×(1-MAF)+2×se(β)^2×n×MAF×(1-MAF)), where *F* represents *F*-statistic, R^2 represents the phenotypic variance explained by a genetic instrument, n is the sample size, k is the number of IVs, β is the estimated genetic association of SNP with the exposure, se(β) is the standard error of effect size, MAF is the minor allele frequency.

Supplementary Table 8. Causal relationships of COVID-19 phenotypes and prostate cancer.

Outcome	MR approach	SRAS-CoV-2 infection			COVID-19 hospitalization			COVID-19 critical illness		
		No. of IVs	OR (95%CI)	*P*-value	No. of IVs	OR (95%CI)	*P*-value	No. of IVs	OR (95%CI)	*P*-value
Prostate cancer	IVW	16	0.991 (0.912-1.076)	0.83	37	0.985 (0.927-1.046)	0.61	36	1.007 (0.967-1.050)	0.73
	MR-Egger	16	1.041 (0.880-1.230)	0.65	37	1.075 (0.964-1.200)	0.20	36	1.049 (0.974-1.130)	0.22
	MR-Egger intercept			0.51			0.07			0.21
	Weighted median	16	1.021 (0.898-1.160)	0.75	37	1.005 (0.952-1.061)	0.86	36	1.008 (0.974-1.044)	0.64
	MRPRESSO	16	0.991 (0.912-1.076)	0.83	34	1.002 (0.958-1.049)	0.92	33	1.019 (0.986-1.052)	0.27
	(IVW)Without pleiotropic SNPs	13	1.011 (0.922-1.109)	0.82	28	0.965 (0.903-1.031)	0.29	29	0.996 (0.952-1.041)	0.85
	(IVW)Without palindromic SNPs	15	1.001 (0.921-1.088)	0.98	34	0.983 (0.922-1.047)	0.59	33	1.004 (0.961-1.049)	0.85

MR: Mendelian randomization; IVW: inverse-variance weighted; MRPRESSO: MR-Pleiotropy Residual Sum and Outlier; IVs: instrumental variables; No. of IVs: number of IVs; OR: odds ratio; CI: confidence interval.

Supplementary Table 9. Causal relationships of COVID-19 phenotypes with prostate cancer by leave-one-out method.

Exposure	Outcome	SNP leaved out	Beta	Standard error	*P*-value
Infection	Prostate cancer	rs10774673	-0.0110	0.0461	0.8117
Infection	Prostate cancer	rs1123573	-0.0081	0.0461	0.8603
Infection	Prostate cancer	rs11264339	-0.0057	0.0466	0.9033
Infection	Prostate cancer	rs12610495	-0.0044	0.0468	0.9242
Infection	Prostate cancer	rs142770866	-0.0213	0.0461	0.6442
Infection	Prostate cancer	rs184781326	-0.0070	0.0461	0.8801
Infection	Prostate cancer	rs2260685	0.0009	0.0464	0.9844
Infection	Prostate cancer	rs2290859	-0.0275	0.0475	0.5625
Infection	Prostate cancer	rs2834158	-0.0019	0.0468	0.9673
Infection	Prostate cancer	rs35044562	-0.0188	0.0494	0.7041
Infection	Prostate cancer	rs554833	-0.0373	0.0525	0.4770
Infection	Prostate cancer	rs676314	-0.0106	0.0461	0.8185
Infection	Prostate cancer	rs679574	0.0009	0.0466	0.9847
Infection	Prostate cancer	rs7118388	-0.0004	0.0461	0.9927
Infection	Prostate cancer	rs73062389	0.0128	0.0503	0.7985
Infection	Prostate cancer	rs9264740	-0.0122	0.0462	0.7912
Infection	Prostate cancer	All	-0.0092	0.0456	0.8403
Hospitalization	Prostate cancer	rs1123573	-0.0154	0.0316	0.6244
Hospitalization	Prostate cancer	rs117169628	-0.0162	0.0316	0.6082
Hospitalization	Prostate cancer	rs12585036	-0.0178	0.0316	0.5727
Hospitalization	Prostate cancer	rs12610495	-0.0145	0.0322	0.6521
Hospitalization	Prostate cancer	rs139589338	-0.0159	0.0314	0.6121
Hospitalization	Prostate cancer	rs142770866	-0.0202	0.0311	0.5168
Hospitalization	Prostate cancer	rs149533170	-0.0176	0.0312	0.5721
Hospitalization	Prostate cancer	rs1498399	-0.0187	0.0313	0.5507
Hospitalization	Prostate cancer	rs1634761	-0.0054	0.0291	0.8520
Hospitalization	Prostate cancer	rs17219281	-0.0110	0.0311	0.7235
Hospitalization	Prostate cancer	rs17279437	-0.0165	0.0315	0.6019
Hospitalization	Prostate cancer	rs17412601	-0.0187	0.0313	0.5509
Hospitalization	Prostate cancer	rs17885848	-0.0185	0.0311	0.5521
Hospitalization	Prostate cancer	rs2102497	-0.0153	0.0314	0.6276
Hospitalization	Prostate cancer	rs2326562	-0.0117	0.0308	0.7048
Hospitalization	Prostate cancer	rs2517723	-0.0061	0.0288	0.8313
Hospitalization	Prostate cancer	rs2897075	-0.0155	0.0315	0.6214

Hospitalization	Prostate cancer	rs3014983	-0.0135	0.0314	0.6672
Hospitalization	Prostate cancer	rs34536443	-0.0213	0.0313	0.4964
Hospitalization	Prostate cancer	rs34712979	-0.0124	0.0310	0.6889
Hospitalization	Prostate cancer	rs35705950	-0.0211	0.0303	0.4860
Hospitalization	Prostate cancer	rs3848456	-0.0128	0.0315	0.6838
Hospitalization	Prostate cancer	rs41264915	-0.0271	0.0297	0.3618
Hospitalization	Prostate cancer	rs41435745	-0.0122	0.0312	0.6963
Hospitalization	Prostate cancer	rs4475253	-0.0129	0.0312	0.6797
Hospitalization	Prostate cancer	rs4767025	-0.0163	0.0316	0.6053
Hospitalization	Prostate cancer	rs492602	-0.0138	0.0313	0.6595
Hospitalization	Prostate cancer	rs5023077	-0.0078	0.0300	0.7942
Hospitalization	Prostate cancer	rs61078946	-0.0151	0.0315	0.6307
Hospitalization	Prostate cancer	rs61882275	-0.0129	0.0316	0.6836
Hospitalization	Prostate cancer	rs63750417	-0.0225	0.0307	0.4627
Hospitalization	Prostate cancer	rs657152	-0.0194	0.0317	0.5409
Hospitalization	Prostate cancer	rs676314	-0.0164	0.0316	0.6048
Hospitalization	Prostate cancer	rs67959919	-0.0263	0.0368	0.4741
Hospitalization	Prostate cancer	rs78314212	-0.0121	0.0312	0.6978
Hospitalization	Prostate cancer	rs915823	-0.0152	0.0315	0.6281
Hospitalization	Prostate cancer	rs9636867	-0.0136	0.0322	0.6719
Hospitalization	Prostate cancer	All	-0.0156	0.0308	0.6132
Critical illness	Prostate cancer	rs10066378	0.0062	0.0214	0.7714
Critical illness	Prostate cancer	rs10850097	0.0069	0.0215	0.7471
Critical illness	Prostate cancer	rs11208559	0.0088	0.0214	0.6808
Critical illness	Prostate cancer	rs1123573	0.0078	0.0215	0.7170
Critical illness	Prostate cancer	rs1128175	0.0059	0.0215	0.7834
Critical illness	Prostate cancer	rs11614702	0.0137	0.0201	0.4945
Critical illness	Prostate cancer	rs117169628	0.0073	0.0216	0.7343
Critical illness	Prostate cancer	rs12534422	0.0079	0.0214	0.7143
Critical illness	Prostate cancer	rs12585036	0.0063	0.0216	0.7693
Critical illness	Prostate cancer	rs12610495	0.0094	0.0220	0.6704
Critical illness	Prostate cancer	rs12614007	0.0079	0.0214	0.7109
Critical illness	Prostate cancer	rs142770866	0.0045	0.0213	0.8331
Critical illness	Prostate cancer	rs17219281	0.0107	0.0211	0.6135
Critical illness	Prostate cancer	rs17279437	0.0071	0.0215	0.7413
Critical illness	Prostate cancer	rs17713054	0.0069	0.0256	0.7878
Critical illness	Prostate cancer	rs17885848	0.0056	0.0213	0.7919

Critical illness	Prostate cancer	rs199512	0.0039	0.0211	0.8544
Critical illness	Prostate cancer	rs2236645	0.0101	0.0212	0.6349
Critical illness	Prostate cancer	rs28368148	0.0066	0.0214	0.7597
Critical illness	Prostate cancer	rs2897075	0.0076	0.0215	0.7223
Critical illness	Prostate cancer	rs343320	0.0064	0.0214	0.7651
Critical illness	Prostate cancer	rs34536443	0.0038	0.0215	0.8605
Critical illness	Prostate cancer	rs34712979	0.0101	0.0211	0.6335
Critical illness	Prostate cancer	rs35705950	0.0035	0.0208	0.8675
Critical illness	Prostate cancer	rs368565	0.0099	0.0213	0.6427
Critical illness	Prostate cancer	rs396379	0.0138	0.0194	0.4783
Critical illness	Prostate cancer	rs41264915	0.0004	0.0205	0.9861
Critical illness	Prostate cancer	rs550057	0.0062	0.0215	0.7740
Critical illness	Prostate cancer	rs568035	0.0041	0.0207	0.8442
Critical illness	Prostate cancer	rs60132559	0.0070	0.0215	0.7454
Critical illness	Prostate cancer	rs61882275	0.0095	0.0215	0.6577
Critical illness	Prostate cancer	rs62056905	0.0033	0.0210	0.8752
Critical illness	Prostate cancer	rs77534576	0.0100	0.0213	0.6404
Critical illness	Prostate cancer	rs79152783	0.0053	0.0214	0.8058
Critical illness	Prostate cancer	rs9305744	0.0114	0.0207	0.5807
Critical illness	Prostate cancer	rs9636867	0.0097	0.0219	0.6579
Critical illness	Prostate cancer	All	0.0074	0.0210	0.7261

SNP: single nucleotide polymorphisms.

Supplementary Table 10. Details of instrumental variables selected for prostate cancer.

SNP	CHR	Position	Effect allele	Other allele	Effect allele frequency	Beta	Standard error	*P*-value	Pleiotropic traits	Combined R^2	*F*-statistic
rs56391074	1	88210715	A	AT	0.6298	-0.0466	0.0082	1.66E-08		0.101	91.18
rs34579442	1	153899900	CT	C	0.6637	-0.0655	0.0087	4.48E-14			
rs62106670	2	8597123	T	C	0.3791	0.0524	0.0090	7.11E-09			
rs74702681	2	66652885	T	C	0.0222	0.1586	0.0264	1.96E-09			
rs34925593	2	174234547	T	C	0.5183	-0.0466	0.0084	2.82E-08			
rs59308963	2	202123479	T	TATTCTGTC	0.7291	0.0505	0.0090	2.41E-08			
rs1283104	3	106962521	C	G	0.6212	-0.0470	0.0082	8.81E-09			
rs182314334	3	152004202	T	C	0.8953	0.0887	0.0134	4.06E-11			
rs142436749	3	169093100	A	G	0.9879	-0.2212	0.0378	4.70E-09			
rs12665339	6	30601232	A	G	0.8326	-0.0615	0.0106	5.56E-09			
rs9296068	6	32988695	T	G	0.6513	0.0477	0.0084	1.34E-08			
rs9469899	6	34793124	A	G	0.3570	0.0484	0.0083	5.27E-09	Medication use (thyroid preparations)		
rs4711748	6	43694598	T	C	0.2254	0.0521	0.0094	3.36E-08			
rs11452686	7	20414110	T	TA	0.5580	0.0497	0.0086	7.81E-09			
rs1048169	9	19055965	T	C	0.6210	-0.0609	0.0081	6.53E-14			
rs10122495	9	34049779	A	T	0.7040	-0.0501	0.0088	1.34E-08			
rs1182	9	132576060	A	C	0.2198	0.0581	0.0095	1.10E-09			
rs141536087	10	854691	G	GCGCA	0.8500	-0.0805	0.0113	8.99E-13			
rs1881502	11	1507512	T	C	0.1903	0.0581	0.0101	7.42E-09			
rs2277283	11	61908440	T	C	0.6868	-0.0558	0.0089	3.03E-10			
rs12785905	11	66951965	C	G	0.0466	0.1157	0.0200	7.82E-09			
rs11290954	11	76260543	A	AC	0.3239	-0.0609	0.0085	7.38E-13			
rs1800057	11	108143456	C	G	0.9766	-0.1500	0.0260	8.15E-09	Renal cell carcinoma		
rs138466039	11	125054793	T	C	0.0089	0.2806	0.0418	2.01E-11			
rs878987	11	134266372	A	G	0.8541	-0.0639	0.0117	4.77E-08			
rs1004030	14	23305649	T	C	0.5844	0.0462	0.0082	1.55E-08			
rs4924487	15	40922915	C	G	0.8359	0.0622	0.0109	1.32E-08			
rs112293876	15	66764641	CA	C	0.7115	-0.0573	0.0091	3.52E-10			
rs142444269	17	30098749	T	C	0.2181	-0.0666	0.0106	3.19E-10			
rs8093601	18	51772473	C	G	0.4421	0.0451	0.0081	2.31E-08			
rs12956892	18	56746315	T	G	0.3021	0.0498	0.0086	7.68E-09			

rs10460109	18	73036165	T	C	0.4143	0.0441	0.0080	3.48E-08	
rs11666569	19	17214073	T	C	0.2867	-0.0516	0.0090	8.17E-09	
rs118005503	19	32167803	C	G	0.0889	-0.0902	0.0156	7.31E-09	
rs11480453	20	31347512	CA	C	0.3984	-0.0463	0.0084	3.19E-08	
rs9625483	22	28888939	A	G	0.0288	0.1338	0.0240	2.43E-08	
rs11691517	2	111893096	T	G	0.7411	0.0635	0.0091	3.51E-12	
rs10793821	5	133836209	T	C	0.5795	0.0527	0.0080	5.43E-11	
rs76551843	5	169172133	A	G	0.9913	0.2705	0.0480	1.70E-08	
rs4976790	5	177968915	T	G	0.1131	0.0737	0.0127	6.73E-09	
rs527510716	7	1944537	C	G	0.2408	0.0585	0.0107	4.94E-08	
rs17621345	7	40875192	A	C	0.7413	0.0715	0.0095	6.72E-14	
rs1935581	10	90195149	T	C	0.3728	-0.0477	0.0082	6.55E-09	
rs7094871	10	114712154	C	G	0.4597	-0.0435	0.0080	4.84E-08	
rs2066827	12	12871099	T	G	0.7551	0.0564	0.0094	2.31E-09	
rs10845938	12	14416918	A	G	0.4459	-0.0572	0.0080	9.80E-13	
rs7968403	12	65012824	T	C	0.6425	0.0589	0.0085	3.38E-12	
rs5799921	12	90160530	G	GA	0.3032	-0.0608	0.0089	7.05E-12	
rs7295014	12	133067989	A	G	0.6578	-0.0516	0.0084	9.50E-10	
rs11629412	14	37138294	C	G	0.5821	0.0573	0.0082	2.34E-12	
rs33984059	15	56385868	A	G	0.9775	0.1761	0.0308	1.10E-08	
rs11863709	16	57654576	T	C	0.0403	-0.1481	0.0220	1.78E-11	
rs201158093	16	82178893	TA	TAA	0.5648	-0.0487	0.0085	9.07E-09	
rs28441558	17	7803118	T	C	0.9452	-0.1507	0.0182	1.02E-16	Smoking initiation (ever regular vs never regular)
rs2680708	17	56456120	A	G	0.3939	-0.0462	0.0082	1.58E-08	
rs28607662	18	53230859	T	C	0.9033	-0.0746	0.0134	2.85E-08	
rs61088131	19	42700947	T	C	0.8229	0.0624	0.0108	8.83E-09	
rs6091758	20	52455205	A	G	0.5349	-0.0719	0.0083	6.42E-18	
rs61890184	11	7547587	A	G	0.1237	0.0706	0.0122	6.56E-09	
rs547171081	11	47421962	CGG	C	0.4679	0.0468	0.0085	3.37E-08	
rs533722308	18	60961193	CT	C	0.4172	0.0520	0.0091	1.21E-08	
rs17321482	23	11482634	T	C	0.1330	-0.0671	0.0091	2.05E-13	
rs4245739	1	204518842	A	C	0.7379	0.0924	0.0091	3.17E-24	Systolic blood pressure
rs9306895	2	20878153	T	C	0.6366	-0.0773	0.0082	3.07E-21	

rs1465618	2	43553949	T	C	0.2141	0.0829	0.0095	2.08E-18	
rs721048	2	63131731	A	G	0.1822	0.0971	0.0101	5.00E-22	
rs10187424	2	85794297	T	C	0.5738	0.0744	0.0080	1.27E-20	
rs12621278	2	173311553	A	G	0.9414	0.2423	0.0178	2.22E-42	
rs2292884	2	238443226	A	G	0.7587	-0.0606	0.0092	3.69E-11	
rs10936632	3	170130102	A	C	0.5074	0.0972	0.0080	3.36E-34	
rs7679673	4	106061534	A	C	0.4078	-0.1201	0.0081	1.16E-49	Cancer (pleiotropy)
rs4713266	6	11219030	T	C	0.4831	-0.0514	0.0079	9.52E-11	
rs7767188	6	30073776	A	G	0.2102	0.0544	0.0097	2.02E-08	
rs3096702	6	32192331	A	G	0.3771	0.0559	0.0082	9.55E-12	
rs3129859	6	32400939	C	G	0.3302	-0.0602	0.0085	1.30E-12	Acute-on-chronic liver failure in hepatitis B
rs2273669	6	109285189	A	G	0.8538	-0.0694	0.0110	3.25E-10	
rs1933488	6	153441079	A	G	0.5788	0.0760	0.0081	3.98E-21	
rs56232506	7	47437244	A	G	0.4507	0.0540	0.0080	1.28E-11	White blood cell count
rs6465657	7	97816327	T	C	0.5365	-0.1005	0.0079	6.97E-37	
rs2928679	8	23438975	A	G	0.4375	0.0534	0.0080	1.86E-11	
rs12543663	8	127924659	A	C	0.7053	-0.1114	0.0086	2.57E-38	
rs10086908	8	128011937	T	C	0.6964	0.1255	0.0087	1.81E-47	
rs183373024	8	128104117	A	G	0.9931	-1.0680	0.0396	3.70E-160	
rs16901979	8	128124916	A	C	0.0319	0.4450	0.0204	1.42E-105	
rs620861	8	128335673	A	G	0.3691	-0.1386	0.0083	4.94E-63	Waist-hip ratio
rs6983267	8	128413305	T	G	0.4892	-0.2004	0.0079	2.81E-141	Colorectal cancer
rs1447295	8	128485038	A	C	0.1073	0.3450	0.0121	1.17E-179	
rs17694493	9	22041998	C	G	0.8643	-0.0726	0.0114	2.26E-10	Adolescent idiopathic scoliosis
rs7931342	11	68994497	T	G	0.4959	-0.1565	0.0079	9.74E-87	Cancer (pleiotropy)
rs11568818	11	102401661	T	C	0.5500	0.0742	0.0082	1.67E-19	
rs11649743	17	36074979	A	G	0.1945	-0.1216	0.0102	8.96E-33	
rs4430796	17	36098040	A	G	0.5253	0.1973	0.0081	1.26E-132	Endometrial cancer
rs11650494	17	47345186	A	G	0.0779	0.0992	0.0145	9.12E-12	
rs7241993	18	76773973	T	C	0.3051	-0.0761	0.0087	2.29E-18	
rs8102476	19	38735613	T	C	0.4607	-0.0902	0.0080	8.83E-30	
rs17599629	1	150658287	A	G	0.7819	-0.0654	0.0096	9.87E-12	

rs1218582	1	154834183	A	G	0.5533	-0.0457	0.0080	9.58E-09	
rs9287719	2	10710730	T	C	0.5336	-0.0663	0.0079	6.00E-17	
rs2660753	3	87110674	T	C	0.1028	0.1198	0.0128	6.30E-21	
rs7611694	3	113275624	A	C	0.5790	0.0831	0.0080	4.52E-25	
rs10934853	3	128038373	A	C	0.2770	0.0989	0.0087	1.06E-29	
rs10009409	4	73855253	T	C	0.3108	0.0555	0.0089	4.29E-10	
rs1894292	4	74349158	A	G	0.4848	-0.0620	0.0079	5.21E-15	
rs12500426	4	95514609	A	C	0.4632	0.0685	0.0079	5.11E-18	
rs17021918	4	95562877	T	C	0.3493	-0.0852	0.0083	1.37E-24	
rs2242652	5	1280028	A	G	0.2054	-0.1598	0.0105	3.46E-52	Breast cancer
rs2121875	5	44365545	A	C	0.6700	-0.0481	0.0085	1.43E-08	
rs9364554	6	160833664	T	C	0.2826	0.1037	0.0087	4.58E-33	
rs12155172	7	20994491	A	G	0.2200	0.0925	0.0094	9.12E-23	
rs10486567	7	27976563	A	G	0.2373	-0.1335	0.0094	2.04E-45	Prostate-specific antigen levels
rs11135910	8	25892142	T	C	0.1529	0.0782	0.0110	9.19E-13	
rs76934034	10	46082985	T	C	0.9174	0.1151	0.0151	2.05E-14	
rs10993994	10	51549496	T	C	0.3830	0.2075	0.0080	2.29E-147	Eczema
rs3850699	10	104414221	A	G	0.6995	0.0704	0.0087	6.02E-16	
rs4962416	10	126696872	T	C	0.7332	-0.0593	0.0089	2.39E-11	
rs7127900	11	2233574	A	G	0.1985	0.1704	0.0098	4.94E-68	
rs11214775	11	113807181	A	G	0.2915	-0.0710	0.0090	3.93E-15	
rs80130819	12	48419618	A	C	0.9084	0.0957	0.0142	1.89E-11	
rs10875943	12	49676010	T	C	0.7128	-0.0688	0.0087	2.35E-15	
rs902774	12	53273904	A	G	0.1526	0.1258	0.0108	1.70E-31	Cerebrospinal fluid t-tau:AB1-42 ratio
rs1270884	12	114685571	A	G	0.4825	0.0697	0.0079	1.22E-18	
rs8008270	14	53372330	T	C	0.1861	-0.0832	0.0103	6.37E-16	
rs7141529	14	69126744	T	C	0.5010	-0.0505	0.0079	1.62E-10	
rs8014671	14	71092256	A	G	0.4198	-0.0466	0.0083	1.67E-08	
rs684232	17	618965	T	C	0.6466	-0.0832	0.0082	4.34E-24	
rs1859962	17	69108753	T	G	0.5187	-0.1606	0.0079	1.13E-91	
rs11672691	19	41985587	A	G	0.2632	-0.0916	0.0092	1.67E-23	
rs2735839	19	51364623	A	G	0.1473	-0.1666	0.0115	3.34E-47	Elevated serum prostate-specific antigen levels in healthy men

rs2427345	20	61015611	T	C	0.3795	-0.0452	0.0082	3.29E-08	Post bronchodilator FEV1/FVC ratio
rs6062509	20	62362563	T	G	0.6983	0.0775	0.0089	3.10E-18	
rs58133635	22	40471188	T	C	0.1972	0.0681	0.0099	4.82E-12	
rs5759167	22	43500212	T	G	0.4979	-0.1423	0.0080	5.55E-71	
rs3771570	2	242382864	T	C	0.1495	0.0841	0.0109	1.19E-14	
rs138213197	17	46805705	T	C	0.0022	1.3475	0.0806	9.17E-63	
rs2405942	23	9814135	A	G	0.7833	0.0486	0.0069	1.65E-12	
rs5945619	23	51241672	T	C	0.6360	-0.1043	0.0057	9.67E-74	
rs2807031	23	52896949	T	C	0.8193	-0.0581	0.0072	7.47E-16	
rs5919432	23	67021550	T	C	0.8008	0.0429	0.0071	1.43E-09	
rs4844289	23	70407983	A	G	0.6163	-0.0408	0.0058	2.90E-12	

SNP: single nucleotide polymorphisms. CHR: chromosome. *F*-statistic was calculated using the following formulas: *F*=R^2 (n-k-1)⁄k(1-R^2) and R^2=2×β^2×MAF×(1-MAF)/(2×β^2×MAF×(1-MAF)+2×se(β)^2×n×MAF×(1-MAF)), where *F* represents *F*-statistic, R^2 represents the phenotypic variance explained by a genetic instrument, n is the sample size, k is the number of IVs, β is the estimated genetic association of SNP with the exposure, se(β) is the standard error of effect size, MAF is the minor allele frequency.

Supplementary Table 11. Causal relationships of prostate cancer and COVID-19 phenotypes.

Exposure	MR approach	SRAS-CoV-2 infection			COVID-19 hospitalization			COVID-19 critical illness		
		No. of IVs	OR (95%CI)	*P*-value	No. of IVs	OR (95%CI)	*P*-value	No. of IVs	OR (95%CI)	*P*-value
Prostate cancer	IVW	134	0.997 (0.986-1.009)	0.66	125	0.986 (0.958-1.015)	0.34	123	0.982 (0.941-1.024)	0.39
	MR-Egger	134	0.994 (0.971-1.017)	0.59	125	1.019 (0.963-1.079)	0.51	123	1.049 (0.962-1.143)	0.28
	MR-Egger intercept			0.71			0.19			0.09
	Weighted median	134	1.019 (0.986-1.052)	0.31	125	1.008 (0.973-1.044)	0.66	123	0.996 (0.944-1.051)	0.88
	MRPRESSO	137	0.995 (0.982-1.007)	0.41	138	0.975 (0.945-1.005)	0.11	138	0.981 (0.940-1.025)	0.39
	(IVW)Without pleiotropic SNPs	116	0.990 (0.976-1.004)	0.15	107	0.970 (0.938-1.003)	0.07	105	0.974 (0.929-1.021)	0.28
	(IVW)Without palindromic SNPs	123	0.996 (0.984-1.008)	0.50	115	0.990 (0.962-1.018)	0.47	113	0.988 (0.947-1.031)	0.58

MR: Mendelian randomization; IVW: inverse-variance weighted; MRPRESSO: MR-Pleiotropy Residual Sum and Outlier; IVs: instrumental variables; No. of IVs: number of IVs; OR: odds ratio; CI: confidence interval.

Supplementary Table 12. Causal relationships of prostate cancer with COVID-19 phenotypes by leave-one-out method.

Exposure	Outcome	SNP leaved out	Beta	Standard error	*P*-value
Prostate cancer	Infection	rs10009409	-0.0028	0.0061	0.6490
Prostate cancer	Infection	rs1004030	-0.0027	0.0061	0.6591
Prostate cancer	Infection	rs10086908	-0.0023	0.0061	0.7105
Prostate cancer	Infection	rs10122495	-0.0026	0.0061	0.6690
Prostate cancer	Infection	rs10187424	-0.0029	0.0061	0.6335
Prostate cancer	Infection	rs10460109	-0.0025	0.0061	0.6854
Prostate cancer	Infection	rs1048169	-0.0025	0.0061	0.6816
Prostate cancer	Infection	rs10486567	-0.0033	0.0061	0.5882
Prostate cancer	Infection	rs10793821	-0.0023	0.0061	0.7014
Prostate cancer	Infection	rs10845938	-0.0028	0.0061	0.6500
Prostate cancer	Infection	rs10875943	-0.0026	0.0061	0.6680
Prostate cancer	Infection	rs10934853	-0.0014	0.0060	0.8197
Prostate cancer	Infection	rs10936632	-0.0030	0.0061	0.6254
Prostate cancer	Infection	rs10993994	-0.0031	0.0062	0.6196
Prostate cancer	Infection	rs11135910	-0.0021	0.0060	0.7312
Prostate cancer	Infection	rs11214775	-0.0021	0.0060	0.7224
Prostate cancer	Infection	rs11290954	-0.0024	0.0061	0.6919
Prostate cancer	Infection	rs11452686	-0.0025	0.0061	0.6805
Prostate cancer	Infection	rs11480453	-0.0025	0.0061	0.6796
Prostate cancer	Infection	rs11568818	-0.0024	0.0061	0.6881
Prostate cancer	Infection	rs11629412	-0.0032	0.0060	0.5970
Prostate cancer	Infection	rs11649743	-0.0035	0.0061	0.5679
Prostate cancer	Infection	rs11650494	-0.0028	0.0061	0.6455
Prostate cancer	Infection	rs11666569	-0.0027	0.0061	0.6571
Prostate cancer	Infection	rs11691517	-0.0026	0.0061	0.6693
Prostate cancer	Infection	rs118005503	-0.0027	0.0061	0.6516
Prostate cancer	Infection	rs1182	-0.0029	0.0061	0.6347
Prostate cancer	Infection	rs11863709	-0.0025	0.0061	0.6768
Prostate cancer	Infection	rs12155172	-0.0023	0.0061	0.6993
Prostate cancer	Infection	rs1218582	-0.0029	0.0061	0.6327
Prostate cancer	Infection	rs12500426	-0.0023	0.0061	0.7106
Prostate cancer	Infection	rs12543663	-0.0026	0.0061	0.6735
Prostate cancer	Infection	rs12621278	-0.0026	0.0061	0.6747
Prostate cancer	Infection	rs12665339	-0.0024	0.0061	0.6866

Prostate cancer	Infection	rs1270884	-0.0026	0.0061	0.6723
Prostate cancer	Infection	rs12785905	-0.0025	0.0061	0.6797
Prostate cancer	Infection	rs1283104	-0.0033	0.0060	0.5864
Prostate cancer	Infection	rs12956892	-0.0028	0.0061	0.6408
Prostate cancer	Infection	rs138213197	-0.0031	0.0061	0.6101
Prostate cancer	Infection	rs138466039	-0.0026	0.0061	0.6721
Prostate cancer	Infection	rs141536087	-0.0027	0.0061	0.6526
Prostate cancer	Infection	rs142436749	-0.0028	0.0061	0.6425
Prostate cancer	Infection	rs142444269	-0.0027	0.0061	0.6504
Prostate cancer	Infection	rs1447295	-0.0011	0.0062	0.8553
Prostate cancer	Infection	rs1465618	-0.0023	0.0061	0.7103
Prostate cancer	Infection	rs16901979	-0.0017	0.0062	0.7770
Prostate cancer	Infection	rs17021918	-0.0024	0.0061	0.6888
Prostate cancer	Infection	rs17321482	-0.0031	0.0060	0.6116
Prostate cancer	Infection	rs17599629	-0.0028	0.0061	0.6399
Prostate cancer	Infection	rs17621345	-0.0026	0.0061	0.6696
Prostate cancer	Infection	rs17694493	-0.0030	0.0060	0.6199
Prostate cancer	Infection	rs1800057	-0.0024	0.0061	0.6882
Prostate cancer	Infection	rs182314334	-0.0025	0.0060	0.6833
Prostate cancer	Infection	rs183373024	-0.0013	0.0061	0.8285
Prostate cancer	Infection	rs1859962	-0.0019	0.0062	0.7534
Prostate cancer	Infection	rs1881502	-0.0026	0.0061	0.6675
Prostate cancer	Infection	rs1894292	-0.0027	0.0061	0.6598
Prostate cancer	Infection	rs1933488	-0.0031	0.0061	0.6127
Prostate cancer	Infection	rs1935581	-0.0023	0.0060	0.6997
Prostate cancer	Infection	rs2066827	-0.0025	0.0061	0.6794
Prostate cancer	Infection	rs2121875	-0.0023	0.0060	0.7090
Prostate cancer	Infection	rs2242652	-0.0032	0.0061	0.6003
Prostate cancer	Infection	rs2273669	-0.0022	0.0060	0.7207
Prostate cancer	Infection	rs2277283	-0.0026	0.0061	0.6673
Prostate cancer	Infection	rs2292884	-0.0026	0.0061	0.6686
Prostate cancer	Infection	rs2405942	-0.0028	0.0061	0.6424
Prostate cancer	Infection	rs2427345	-0.0024	0.0061	0.6861
Prostate cancer	Infection	rs2660753	-0.0032	0.0061	0.6027
Prostate cancer	Infection	rs2680708	-0.0031	0.0060	0.6106
Prostate cancer	Infection	rs2735839	-0.0032	0.0061	0.6050
Prostate cancer	Infection	rs2807031	-0.0027	0.0061	0.6605

Prostate cancer	Infection	rs28441558	-0.0035	0.0060	0.5577
Prostate cancer	Infection	rs28607662	-0.0028	0.0061	0.6431
Prostate cancer	Infection	rs2928679	-0.0019	0.0060	0.7501
Prostate cancer	Infection	rs3096702	-0.0024	0.0061	0.6894
Prostate cancer	Infection	rs3129859	-0.0030	0.0061	0.6181
Prostate cancer	Infection	rs33984059	-0.0025	0.0061	0.6800
Prostate cancer	Infection	rs34579442	-0.0024	0.0060	0.6921
Prostate cancer	Infection	rs34925593	-0.0034	0.0059	0.5683
Prostate cancer	Infection	rs3771570	-0.0024	0.0061	0.6954
Prostate cancer	Infection	rs3850699	-0.0027	0.0061	0.6562
Prostate cancer	Infection	rs4245739	-0.0019	0.0060	0.7513
Prostate cancer	Infection	rs4430796	-0.0019	0.0062	0.7543
Prostate cancer	Infection	rs4711748	-0.0025	0.0061	0.6790
Prostate cancer	Infection	rs4713266	-0.0027	0.0061	0.6608
Prostate cancer	Infection	rs4844289	-0.0025	0.0061	0.6854
Prostate cancer	Infection	rs4924487	-0.0025	0.0061	0.6791
Prostate cancer	Infection	rs4962416	-0.0033	0.0060	0.5858
Prostate cancer	Infection	rs4976790	-0.0029	0.0061	0.6333
Prostate cancer	Infection	rs547171081/rs59111863	-0.0027	0.0061	0.6581
Prostate cancer	Infection	rs56232506	-0.0030	0.0060	0.6151
Prostate cancer	Infection	rs56391074	-0.0028	0.0061	0.6440
Prostate cancer	Infection	rs5759167	-0.0020	0.0061	0.7465
Prostate cancer	Infection	rs5799921	-0.0026	0.0061	0.6718
Prostate cancer	Infection	rs58133635	-0.0028	0.0061	0.6421
Prostate cancer	Infection	rs5919432	-0.0026	0.0061	0.6732
Prostate cancer	Infection	rs59308963	-0.0025	0.0061	0.6765
Prostate cancer	Infection	rs5945619	-0.0030	0.0061	0.6186
Prostate cancer	Infection	rs6062509	-0.0026	0.0061	0.6723
Prostate cancer	Infection	rs6091758	-0.0026	0.0061	0.6726
Prostate cancer	Infection	rs61088131	-0.0025	0.0061	0.6788
Prostate cancer	Infection	rs61890184	-0.0029	0.0061	0.6360
Prostate cancer	Infection	rs620861	-0.0021	0.0061	0.7362
Prostate cancer	Infection	rs62106670	-0.0026	0.0061	0.6739
Prostate cancer	Infection	rs6465657	-0.0005	0.0059	0.9324
Prostate cancer	Infection	rs684232	-0.0033	0.0061	0.5858
Prostate cancer	Infection	rs6983267	-0.0027	0.0062	0.6647
Prostate cancer	Infection	rs7094871	-0.0024	0.0061	0.6865

Prostate cancer	Infection	rs7127900	-0.0036	0.0061	0.5519
Prostate cancer	Infection	rs7141529	-0.0029	0.0061	0.6335
Prostate cancer	Infection	rs721048	-0.0025	0.0061	0.6820
Prostate cancer	Infection	rs7241993	-0.0027	0.0061	0.6629
Prostate cancer	Infection	rs7295014	-0.0012	0.0056	0.8323
Prostate cancer	Infection	rs74702681	-0.0031	0.0060	0.6095
Prostate cancer	Infection	rs7611694	-0.0031	0.0061	0.6082
Prostate cancer	Infection	rs76551843	-0.0022	0.0060	0.7180
Prostate cancer	Infection	rs7679673	-0.0036	0.0061	0.5549
Prostate cancer	Infection	rs76934034	-0.0029	0.0061	0.6371
Prostate cancer	Infection	rs7767188	-0.0030	0.0060	0.6186
Prostate cancer	Infection	rs7931342	-0.0041	0.0061	0.4998
Prostate cancer	Infection	rs7968403	-0.0021	0.0060	0.7222
Prostate cancer	Infection	rs8008270	-0.0029	0.0061	0.6269
Prostate cancer	Infection	rs80130819	-0.0027	0.0061	0.6589
Prostate cancer	Infection	rs8014671	-0.0027	0.0061	0.6619
Prostate cancer	Infection	rs8093601	-0.0028	0.0061	0.6393
Prostate cancer	Infection	rs8102476	-0.0034	0.0061	0.5769
Prostate cancer	Infection	rs878987	-0.0033	0.0060	0.5830
Prostate cancer	Infection	rs902774	-0.0032	0.0061	0.5932
Prostate cancer	Infection	rs9287719	-0.0031	0.0061	0.6103
Prostate cancer	Infection	rs9296068	-0.0018	0.0059	0.7566
Prostate cancer	Infection	rs9306895	-0.0024	0.0061	0.6953
Prostate cancer	Infection	rs9364554	-0.0025	0.0061	0.6774
Prostate cancer	Infection	rs9469899	-0.0031	0.0060	0.6018
Prostate cancer	Infection	rs9625483	-0.0027	0.0061	0.6504
Prostate cancer	Infection	All	-0.0026	0.0060	0.6624
Prostate cancer	Hospitalization	rs10009409	-0.0150	0.0147	0.3091
Prostate cancer	Hospitalization	rs1004030	-0.0146	0.0147	0.3228
Prostate cancer	Hospitalization	rs10086908	-0.0142	0.0149	0.3381
Prostate cancer	Hospitalization	rs10122495	-0.0133	0.0147	0.3632
Prostate cancer	Hospitalization	rs10187424	-0.0148	0.0148	0.3174
Prostate cancer	Hospitalization	rs10460109	-0.0143	0.0148	0.3342
Prostate cancer	Hospitalization	rs1048169	-0.0133	0.0147	0.3655
Prostate cancer	Hospitalization	rs10486567	-0.0153	0.0148	0.3019
Prostate cancer	Hospitalization	rs10793821	-0.0129	0.0146	0.3785
Prostate cancer	Hospitalization	rs10845938	-0.0142	0.0148	0.3361

Prostate cancer	Hospitalization	rs10875943	-0.0149	0.0148	0.3133
Prostate cancer	Hospitalization	rs10934853	-0.0131	0.0148	0.3749
Prostate cancer	Hospitalization	rs10936632	-0.0119	0.0147	0.4192
Prostate cancer	Hospitalization	rs10993994	-0.0167	0.0150	0.2638
Prostate cancer	Hospitalization	rs11135910	-0.0138	0.0148	0.3502
Prostate cancer	Hospitalization	rs11214775	-0.0129	0.0147	0.3791
Prostate cancer	Hospitalization	rs11290954	-0.0150	0.0147	0.3062
Prostate cancer	Hospitalization	rs11568818	-0.0143	0.0148	0.3344
Prostate cancer	Hospitalization	rs11629412	-0.0157	0.0145	0.2809
Prostate cancer	Hospitalization	rs11649743	-0.0152	0.0148	0.3029
Prostate cancer	Hospitalization	rs11650494	-0.0140	0.0148	0.3437
Prostate cancer	Hospitalization	rs11666569	-0.0138	0.0148	0.3496
Prostate cancer	Hospitalization	rs11691517	-0.0132	0.0147	0.3704
Prostate cancer	Hospitalization	rs1182	-0.0144	0.0148	0.3310
Prostate cancer	Hospitalization	rs11863709	-0.0142	0.0148	0.3364
Prostate cancer	Hospitalization	rs12155172	-0.0140	0.0148	0.3440
Prostate cancer	Hospitalization	rs1218582	-0.0132	0.0147	0.3688
Prostate cancer	Hospitalization	rs12500426	-0.0118	0.0146	0.4193
Prostate cancer	Hospitalization	rs12543663	-0.0144	0.0148	0.3327
Prostate cancer	Hospitalization	rs12621278	-0.0151	0.0149	0.3106
Prostate cancer	Hospitalization	rs12665339	-0.0134	0.0147	0.3612
Prostate cancer	Hospitalization	rs1270884	-0.0155	0.0147	0.2919
Prostate cancer	Hospitalization	rs12785905	-0.0138	0.0148	0.3507
Prostate cancer	Hospitalization	rs1283104	-0.0145	0.0147	0.3255
Prostate cancer	Hospitalization	rs12956892	-0.0150	0.0147	0.3079
Prostate cancer	Hospitalization	rs138213197	-0.0139	0.0148	0.3461
Prostate cancer	Hospitalization	rs138466039	-0.0142	0.0148	0.3378
Prostate cancer	Hospitalization	rs142436749	-0.0153	0.0146	0.2968
Prostate cancer	Hospitalization	rs1447295	-0.0111	0.0150	0.4594
Prostate cancer	Hospitalization	rs1465618	-0.0135	0.0148	0.3609
Prostate cancer	Hospitalization	rs16901979	-0.0126	0.0150	0.3996
Prostate cancer	Hospitalization	rs17021918	-0.0120	0.0146	0.4125
Prostate cancer	Hospitalization	rs17321482	-0.0151	0.0147	0.3043
Prostate cancer	Hospitalization	rs17599629	-0.0154	0.0147	0.2933
Prostate cancer	Hospitalization	rs17621345	-0.0143	0.0148	0.3322
Prostate cancer	Hospitalization	rs17694493	-0.0137	0.0148	0.3531
Prostate cancer	Hospitalization	rs1800057	-0.0128	0.0146	0.3804

Prostate cancer	Hospitalization	rs183373024	-0.0157	0.0149	0.2893
Prostate cancer	Hospitalization	rs1859962	-0.0162	0.0150	0.2792
Prostate cancer	Hospitalization	rs1881502	-0.0140	0.0148	0.3444
Prostate cancer	Hospitalization	rs1894292	-0.0152	0.0147	0.3020
Prostate cancer	Hospitalization	rs1933488	-0.0136	0.0148	0.3590
Prostate cancer	Hospitalization	rs1935581	-0.0139	0.0148	0.3465
Prostate cancer	Hospitalization	rs2066827	-0.0129	0.0146	0.3792
Prostate cancer	Hospitalization	rs2121875	-0.0140	0.0148	0.3421
Prostate cancer	Hospitalization	rs2242652	-0.0161	0.0149	0.2788
Prostate cancer	Hospitalization	rs2273669	-0.0130	0.0147	0.3766
Prostate cancer	Hospitalization	rs2277283	-0.0136	0.0147	0.3558
Prostate cancer	Hospitalization	rs2292884	-0.0137	0.0148	0.3535
Prostate cancer	Hospitalization	rs2405942	-0.0143	0.0147	0.3319
Prostate cancer	Hospitalization	rs2427345	-0.0123	0.0144	0.3939
Prostate cancer	Hospitalization	rs2660753	-0.0149	0.0148	0.3133
Prostate cancer	Hospitalization	rs2680708	-0.0145	0.0147	0.3270
Prostate cancer	Hospitalization	rs2735839	-0.0153	0.0149	0.3044
Prostate cancer	Hospitalization	rs2807031	-0.0147	0.0148	0.3211
Prostate cancer	Hospitalization	rs28441558	-0.0141	0.0148	0.3408
Prostate cancer	Hospitalization	rs28607662	-0.0135	0.0147	0.3582
Prostate cancer	Hospitalization	rs2928679	-0.0135	0.0147	0.3595
Prostate cancer	Hospitalization	rs3096702	-0.0132	0.0147	0.3678
Prostate cancer	Hospitalization	rs3129859	-0.0127	0.0146	0.3867
Prostate cancer	Hospitalization	rs33984059	-0.0132	0.0147	0.3695
Prostate cancer	Hospitalization	rs34925593	-0.0154	0.0146	0.2936
Prostate cancer	Hospitalization	rs3771570	-0.0141	0.0148	0.3408
Prostate cancer	Hospitalization	rs3850699	-0.0142	0.0148	0.3352
Prostate cancer	Hospitalization	rs4245739	-0.0114	0.0146	0.4330
Prostate cancer	Hospitalization	rs4430796	-0.0167	0.0151	0.2708
Prostate cancer	Hospitalization	rs4711748	-0.0141	0.0148	0.3393
Prostate cancer	Hospitalization	rs4713266	-0.0153	0.0147	0.2954
Prostate cancer	Hospitalization	rs4844289	-0.0132	0.0147	0.3697
Prostate cancer	Hospitalization	rs4924487	-0.0124	0.0145	0.3909
Prostate cancer	Hospitalization	rs4962416	-0.0149	0.0147	0.3100
Prostate cancer	Hospitalization	rs4976790	-0.0136	0.0147	0.3551
Prostate cancer	Hospitalization	rs56232506	-0.0148	0.0147	0.3152
Prostate cancer	Hospitalization	rs56391074	-0.0139	0.0147	0.3467

Prostate cancer	Hospitalization	rs5759167	-0.0154	0.0149	0.3025
Prostate cancer	Hospitalization	rs5799921	-0.0143	0.0148	0.3321
Prostate cancer	Hospitalization	rs58133635	-0.0137	0.0147	0.3511
Prostate cancer	Hospitalization	rs5919432	-0.0144	0.0147	0.3271
Prostate cancer	Hospitalization	rs59308963	-0.0147	0.0147	0.3172
Prostate cancer	Hospitalization	rs5945619	-0.0130	0.0148	0.3783
Prostate cancer	Hospitalization	rs6062509	-0.0135	0.0148	0.3616
Prostate cancer	Hospitalization	rs61088131	-0.0141	0.0148	0.3389
Prostate cancer	Hospitalization	rs61890184	-0.0142	0.0148	0.3345
Prostate cancer	Hospitalization	rs620861	-0.0142	0.0149	0.3418
Prostate cancer	Hospitalization	rs62106670	-0.0142	0.0148	0.3360
Prostate cancer	Hospitalization	rs6465657	-0.0127	0.0148	0.3917
Prostate cancer	Hospitalization	rs684232	-0.0157	0.0147	0.2867
Prostate cancer	Hospitalization	rs6983267	-0.0151	0.0152	0.3189
Prostate cancer	Hospitalization	rs7094871	-0.0147	0.0147	0.3159
Prostate cancer	Hospitalization	rs7127900	-0.0136	0.0149	0.3624
Prostate cancer	Hospitalization	rs7141529	-0.0148	0.0147	0.3165
Prostate cancer	Hospitalization	rs721048	-0.0135	0.0148	0.3621
Prostate cancer	Hospitalization	rs7241993	-0.0143	0.0148	0.3324
Prostate cancer	Hospitalization	rs7295014	-0.0109	0.0138	0.4313
Prostate cancer	Hospitalization	rs74702681	-0.0144	0.0148	0.3304
Prostate cancer	Hospitalization	rs7611694	-0.0145	0.0148	0.3276
Prostate cancer	Hospitalization	rs76551843	-0.0128	0.0145	0.3780
Prostate cancer	Hospitalization	rs7679673	-0.0177	0.0147	0.2278
Prostate cancer	Hospitalization	rs76934034	-0.0149	0.0147	0.3121
Prostate cancer	Hospitalization	rs7767188	-0.0128	0.0146	0.3783
Prostate cancer	Hospitalization	rs7931342	-0.0153	0.0150	0.3066
Prostate cancer	Hospitalization	rs7968403	-0.0142	0.0148	0.3365
Prostate cancer	Hospitalization	rs8008270	-0.0138	0.0148	0.3516
Prostate cancer	Hospitalization	rs80130819	-0.0149	0.0147	0.3104
Prostate cancer	Hospitalization	rs8014671	-0.0145	0.0148	0.3268
Prostate cancer	Hospitalization	rs8093601	-0.0141	0.0148	0.3410
Prostate cancer	Hospitalization	rs8102476	-0.0155	0.0148	0.2947
Prostate cancer	Hospitalization	rs878987	-0.0141	0.0148	0.3382
Prostate cancer	Hospitalization	rs902774	-0.0159	0.0147	0.2820
Prostate cancer	Hospitalization	rs9287719	-0.0150	0.0147	0.3086
Prostate cancer	Hospitalization	rs9296068	-0.0129	0.0146	0.3791

Prostate cancer	Hospitalization	rs9306895	-0.0130	0.0147	0.3766
Prostate cancer	Hospitalization	rs9364554	-0.0133	0.0148	0.3709
Prostate cancer	Hospitalization	rs9469899	-0.0157	0.0145	0.2818
Prostate cancer	Hospitalization	rs9625483	-0.0137	0.0147	0.3523
Prostate cancer	Hospitalization	All	-0.0141	0.0147	0.3362
Prostate cancer	Critical illness	rs10009409	-0.0198	0.0216	0.3597
Prostate cancer	Critical illness	rs1004030	-0.0196	0.0215	0.3633
Prostate cancer	Critical illness	rs10086908	-0.0174	0.0218	0.4260
Prostate cancer	Critical illness	rs10122495	-0.0189	0.0216	0.3825
Prostate cancer	Critical illness	rs10187424	-0.0180	0.0217	0.4066
Prostate cancer	Critical illness	rs10460109	-0.0201	0.0215	0.3502
Prostate cancer	Critical illness	rs1048169	-0.0174	0.0217	0.4229
Prostate cancer	Critical illness	rs10486567	-0.0239	0.0215	0.2682
Prostate cancer	Critical illness	rs10793821	-0.0179	0.0217	0.4090
Prostate cancer	Critical illness	rs10845938	-0.0184	0.0217	0.3974
Prostate cancer	Critical illness	rs10875943	-0.0185	0.0217	0.3940
Prostate cancer	Critical illness	rs10934853	-0.0179	0.0218	0.4111
Prostate cancer	Critical illness	rs10936632	-0.0164	0.0217	0.4497
Prostate cancer	Critical illness	rs10993994	-0.0234	0.0220	0.2878
Prostate cancer	Critical illness	rs11135910	-0.0191	0.0217	0.3787
Prostate cancer	Critical illness	rs11214775	-0.0179	0.0217	0.4104
Prostate cancer	Critical illness	rs11290954	-0.0208	0.0214	0.3330
Prostate cancer	Critical illness	rs11568818	-0.0176	0.0217	0.4167
Prostate cancer	Critical illness	rs11629412	-0.0198	0.0215	0.3564
Prostate cancer	Critical illness	rs11649743	-0.0198	0.0218	0.3624
Prostate cancer	Critical illness	rs11650494	-0.0185	0.0217	0.3949
Prostate cancer	Critical illness	rs11666569	-0.0178	0.0217	0.4107
Prostate cancer	Critical illness	rs11691517	-0.0155	0.0213	0.4677
Prostate cancer	Critical illness	rs1182	-0.0187	0.0217	0.3887
Prostate cancer	Critical illness	rs11863709	-0.0198	0.0216	0.3596
Prostate cancer	Critical illness	rs12155172	-0.0182	0.0217	0.4020
Prostate cancer	Critical illness	rs1218582	-0.0177	0.0216	0.4145
Prostate cancer	Critical illness	rs12500426	-0.0154	0.0215	0.4740
Prostate cancer	Critical illness	rs12543663	-0.0174	0.0218	0.4235
Prostate cancer	Critical illness	rs12621278	-0.0184	0.0218	0.3994
Prostate cancer	Critical illness	rs12665339	-0.0178	0.0216	0.4108
Prostate cancer	Critical illness	rs1270884	-0.0200	0.0217	0.3562

Prostate cancer	Critical illness	rs12785905	-0.0187	0.0217	0.3900
Prostate cancer	Critical illness	rs1283104	-0.0180	0.0217	0.4062
Prostate cancer	Critical illness	rs12956892	-0.0204	0.0214	0.3412
Prostate cancer	Critical illness	rs138466039	-0.0185	0.0217	0.3936
Prostate cancer	Critical illness	rs142436749	-0.0198	0.0216	0.3591
Prostate cancer	Critical illness	rs1447295	-0.0183	0.0220	0.4048
Prostate cancer	Critical illness	rs1465618	-0.0188	0.0217	0.3856
Prostate cancer	Critical illness	rs16901979	-0.0192	0.0221	0.3835
Prostate cancer	Critical illness	rs17021918	-0.0179	0.0217	0.4111
Prostate cancer	Critical illness	rs17321482	-0.0201	0.0215	0.3505
Prostate cancer	Critical illness	rs17599629	-0.0193	0.0217	0.3725
Prostate cancer	Critical illness	rs17621345	-0.0182	0.0217	0.4028
Prostate cancer	Critical illness	rs17694493	-0.0182	0.0217	0.4011
Prostate cancer	Critical illness	rs1800057	-0.0170	0.0215	0.4300
Prostate cancer	Critical illness	rs183373024	-0.0176	0.0218	0.4183
Prostate cancer	Critical illness	rs1859962	-0.0195	0.0221	0.3765
Prostate cancer	Critical illness	rs1881502	-0.0184	0.0217	0.3970
Prostate cancer	Critical illness	rs1894292	-0.0197	0.0217	0.3630
Prostate cancer	Critical illness	rs1933488	-0.0166	0.0217	0.4427
Prostate cancer	Critical illness	rs1935581	-0.0174	0.0216	0.4204
Prostate cancer	Critical illness	rs2066827	-0.0178	0.0217	0.4124
Prostate cancer	Critical illness	rs2121875	-0.0195	0.0216	0.3658
Prostate cancer	Critical illness	rs2242652	-0.0240	0.0217	0.2673
Prostate cancer	Critical illness	rs2273669	-0.0165	0.0215	0.4432
Prostate cancer	Critical illness	rs2277283	-0.0177	0.0217	0.4140
Prostate cancer	Critical illness	rs2292884	-0.0188	0.0217	0.3851
Prostate cancer	Critical illness	rs2405942	-0.0192	0.0216	0.3731
Prostate cancer	Critical illness	rs2427345	-0.0154	0.0210	0.4633
Prostate cancer	Critical illness	rs2660753	-0.0200	0.0217	0.3556
Prostate cancer	Critical illness	rs2680708	-0.0186	0.0217	0.3920
Prostate cancer	Critical illness	rs2735839	-0.0210	0.0218	0.3361
Prostate cancer	Critical illness	rs2807031	-0.0213	0.0216	0.3238
Prostate cancer	Critical illness	rs28441558	-0.0189	0.0217	0.3833
Prostate cancer	Critical illness	rs28607662	-0.0191	0.0217	0.3772
Prostate cancer	Critical illness	rs2928679	-0.0169	0.0216	0.4335
Prostate cancer	Critical illness	rs3096702	-0.0182	0.0217	0.4021
Prostate cancer	Critical illness	rs3129859	-0.0148	0.0211	0.4818

Prostate cancer	Critical illness	rs33984059	-0.0185	0.0217	0.3939
Prostate cancer	Critical illness	rs34925593	-0.0193	0.0217	0.3733
Prostate cancer	Critical illness	rs3771570	-0.0174	0.0217	0.4213
Prostate cancer	Critical illness	rs3850699	-0.0185	0.0217	0.3941
Prostate cancer	Critical illness	rs4245739	-0.0139	0.0213	0.5160
Prostate cancer	Critical illness	rs4430796	-0.0133	0.0222	0.5492
Prostate cancer	Critical illness	rs4711748	-0.0182	0.0217	0.4012
Prostate cancer	Critical illness	rs4713266	-0.0188	0.0217	0.3874
Prostate cancer	Critical illness	rs4844289	-0.0179	0.0217	0.4100
Prostate cancer	Critical illness	rs4924487	-0.0165	0.0214	0.4410
Prostate cancer	Critical illness	rs4962416	-0.0202	0.0216	0.3479
Prostate cancer	Critical illness	rs4976790	-0.0180	0.0217	0.4061
Prostate cancer	Critical illness	rs56232506	-0.0179	0.0217	0.4101
Prostate cancer	Critical illness	rs56391074	-0.0194	0.0215	0.3677
Prostate cancer	Critical illness	rs5759167	-0.0209	0.0219	0.3410
Prostate cancer	Critical illness	rs5799921	-0.0191	0.0217	0.3783
Prostate cancer	Critical illness	rs5919432	-0.0186	0.0217	0.3904
Prostate cancer	Critical illness	rs59308963	-0.0196	0.0216	0.3645
Prostate cancer	Critical illness	rs5945619	-0.0192	0.0218	0.3772
Prostate cancer	Critical illness	rs6062509	-0.0188	0.0217	0.3883
Prostate cancer	Critical illness	rs61088131	-0.0187	0.0217	0.3884
Prostate cancer	Critical illness	rs61890184	-0.0188	0.0217	0.3861
Prostate cancer	Critical illness	rs620861	-0.0174	0.0219	0.4272
Prostate cancer	Critical illness	rs62106670	-0.0187	0.0217	0.3887
Prostate cancer	Critical illness	rs6465657	-0.0193	0.0218	0.3765
Prostate cancer	Critical illness	rs684232	-0.0202	0.0217	0.3509
Prostate cancer	Critical illness	rs6983267	-0.0192	0.0223	0.3902
Prostate cancer	Critical illness	rs7094871	-0.0182	0.0217	0.4017
Prostate cancer	Critical illness	rs7127900	-0.0192	0.0219	0.3801
Prostate cancer	Critical illness	rs7141529	-0.0183	0.0217	0.3993
Prostate cancer	Critical illness	rs721048	-0.0193	0.0217	0.3734
Prostate cancer	Critical illness	rs7241993	-0.0176	0.0217	0.4183
Prostate cancer	Critical illness	rs7295014	-0.0136	0.0202	0.5025
Prostate cancer	Critical illness	rs74702681	-0.0192	0.0217	0.3744
Prostate cancer	Critical illness	rs7611694	-0.0190	0.0217	0.3827
Prostate cancer	Critical illness	rs76551843	-0.0173	0.0214	0.4205
Prostate cancer	Critical illness	rs7679673	-0.0251	0.0215	0.2417

Prostate cancer	Critical illness	rs76934034	-0.0187	0.0217	0.3881
Prostate cancer	Critical illness	rs7767188	-0.0172	0.0215	0.4257
Prostate cancer	Critical illness	rs7931342	-0.0169	0.0220	0.4445
Prostate cancer	Critical illness	rs7968403	-0.0171	0.0216	0.4277
Prostate cancer	Critical illness	rs8008270	-0.0186	0.0217	0.3902
Prostate cancer	Critical illness	rs80130819	-0.0190	0.0217	0.3807
Prostate cancer	Critical illness	rs8014671	-0.0182	0.0217	0.4025
Prostate cancer	Critical illness	rs8093601	-0.0186	0.0217	0.3916
Prostate cancer	Critical illness	rs8102476	-0.0195	0.0218	0.3717
Prostate cancer	Critical illness	rs878987	-0.0179	0.0217	0.4091
Prostate cancer	Critical illness	rs902774	-0.0198	0.0217	0.3617
Prostate cancer	Critical illness	rs9287719	-0.0206	0.0216	0.3400
Prostate cancer	Critical illness	rs9296068	-0.0156	0.0211	0.4605
Prostate cancer	Critical illness	rs9306895	-0.0157	0.0215	0.4651
Prostate cancer	Critical illness	rs9364554	-0.0167	0.0217	0.4429
Prostate cancer	Critical illness	rs9469899	-0.0187	0.0217	0.3894
Prostate cancer	Critical illness	rs9625483	-0.0189	0.0216	0.3812
Prostate cancer	Critical illness	All	-0.0185	0.0216	0.3907

SNP: single nucleotide polymorphisms.
